# The technological, organizational and environmental determinants of adoption of mobile health applications (m-health) by hospitals in Kenya

**DOI:** 10.1371/journal.pone.0225167

**Published:** 2019-12-13

**Authors:** Bahati Prince Ngongo, Phares Ochola, Joyce Ndegwa, Paul Katuse

**Affiliations:** Chandaria School of Business, United States International University- Africa, Nairobi, Nairobi, Kenya; National University Singapore Saw Swee Hock School of Public Health, SINGAPORE

## Abstract

**Introduction:**

Sub-Saharan Africa lags in adoption of mobile health (m-health) applications and in leveraging m-health for sustainable development goals. There is a need for a comprehensive investigation of determinants of hospitals’ adoption of m-health in Sub-Saharan Africa to inform policies, practices and investments.

**Methods:**

This investigation used a logit regression model to analyze the determinants of m-health adoption in Kenyan hospitals applying the Technological, Organizational and Environmental (TOE) framework and the Diffusion of Innovation (DOI) theory. A representative sample of 211 executives of Level 4–6 hospitals in 24 counties provided primary data on Patient-Centered (PC) and Facility-Centered (FC) m-health applications.

**Results:**

Both PC and FC m-health adoption were predicted by competition for patients (PC p = 0.041, FC p = 0.021), IT human resource capacity (PC p = 0.048, FC p = 0.037), and hospital pursuit of market growth through technological leadership (PC p = 0.010, FC p = 0.020). Further determinants of PC m-health adoption included hospital access to slack financial resources (p = 0.006), acquisition strategy (p = 0.011), compatibility with the hospital systems (p = 0.015), trialability (p = 0.019), medical insurance company support (p = 0.025),patient pressure (p = 0.036), and perceived effect of global medical tourism (p = 0.039). FC m-health adoption was predicted by hospital size (p = 0.008), ICT infrastructure capacity (p = 0.041), and government support (p = 0.013).

**Conclusion:**

A differentiated approach is required to scale up m-health adoption. PC m-health requires emphasis on establishing national and regional compatibility and interoperability, developing trialability processes and validation mechanisms, incentivizing patient competition and mobility, establishing innovative and cost-effective acquisition strategies, and ensuring integration of digital services within national insurance schemes and policies. These policies require support from patients and communities to drive demand and spur investment in adequate IT human resources to maintain reliability. Pilot PC m-health projects should prioritize hospitals with slack financial resources, while FC m-health should target large facility size. FC m-health applications are more complex and costly than PC, requiring government incentives to trigger hospital investments and national investment in ICT infrastructure. Investors and hospital managers should integrate m-health into market growth strategies for sustainable m-health scale-up in Kenya and beyond.

## 1. Introduction

The sustainable development goals (SDG) acknowledge the transformational impact that digital health technologies such as mobile health (m-health) will have in a context of continued global population growth, inequitable access to health, increased healthcare costs, and the limited number of health care workers [1,2]. Accordingly, global investment in m-health innovations has risen exponentially in the last five years to respond to the market needs and potential. Gagnon [3] estimated that there were an estimated 40,000 m-health applications launched in 2012 and projected that the number will double by 2017. Akter & Ray [4] projected that the m-health market will reach US $23 billion by 2017. However, while m-health adoption by hospitals is expanding in high income countries, the adoption of m-health applications in Low- and Middle-Income Countries (LMICs) in general and particularly in Sub-Saharan Africa, remains negligible with limited understanding of factors affecting this disparity [5]. According to WHO [6], despite Africa’s leadership in mobile phone penetration and mobile financing (m-Financing), the region lags North America, Europe, South America, and Southeast Asia in health system adoption of m-health. Africa also registers the highest rate of failure of m-health projects [4,7].

WHO defines m-health as the use of any wireless technology or portable device by health providers to enable communication between patients and health services, for consultation between health care professionals, for health monitoring and surveillance, for access to information for health care professionals at point of care [6]. It identified twelve general applications of m-health that could be further categorized as patient-centered (PC) and facility-centered (FC) (see Table 1). PC m-health applications aim to facilitate communications and data between patients and health providers while FC m-health applications mostly facilitate communications and data between healthcare providers.

**Table 1:** m-health applications categories and re-categorization as per WHO.

m-Health Intervention Taxonomy	m-Health intervention sub-grouping
Patient-Centered (PC)	Health call centers/telephone help line
	Emergency toll-free telephone services
	Treatment compliance
	Appointment reminders
	Community mobilization
	Awareness raising over health issues
	Mobile surveys (surveys by mobile phone)
	Surveillance
	Patient monitoring
Facility-Centered (FC)	Mobile telemedicine
	Information and decision support systems
	Patient records

According to the Kenya Health sector market survey [8], Kenya is amongst African countries that lag in adoption of m-health despite its success in ICT connectivity (99% of internet subscribers accessing the internet through their mobile phone in 2016), high mobile phone penetration (88.1% of penetration of smart mobile phone in 2016 [9]), high mobile financing system and a progressive digital health policy. There is limited investigation of determinants of adoption of m-health in Sub-Saharan Africa and in Kenya in particular. Most studies on adoption of m-health in Sub-Saharan Africa are qualitative in nature, limited in scope and focus on either patient or health care providers acceptability of a specific m-health technology [3]. This has led to what has been identified as a techno-optimistic view of adoption of m-health that does not lead to scalable organizational adoption [3,4,7,10]. To the best of available knowledge, this study is amongst the first to comprehensively assess determinants of all the 12 WHO applications, using a comprehensive analysis of technological, organizational and environmental determinants with the top executive decision makers of organizational adoption of m-health in Kenya. It is also the first to investigate the differential effect of PC and FC m-health adoption. This study does not focus on any single specific technology as such approaches have less predictive power of adoption than the general m-health applications used by WHO [4,7,11–13]. It also defines adoption of innovation using Rogers [14] definition as a binary process of the hospital formally accepting and currently using m-health applications or rejecting (i.e. currently not using) the use of m-health at the time of the study.

Because of the focus on hospital adoption, this study used the most commonly used framework of Technology, Organizational and Environmental (TOE) established by Tornatzky and Fleischer's [13,15] to investigate the determinants of adoption or non-adoption of m-health by hospitals in Kenya. These constructs have been used in multiple studies with significant degree of reliability and predictability of adoption [16–19]. However, due to the limitations of the TOE to provide specificities in the technological determinants related to innovations in Information and Communication and Technology, this study integrated the theory of the Diffusion of Innovation (DOI) by Rogers [14] to analyze the technological attributes of m-health adoption. This integrated approach of TOE and DOI has been used by multiple studies and proved higher degree of reliability and validation [20–22].

The technological constructs in this study were adopted from Rogers’ DOI [14]. Rogers’ DOI theory posits that adoption of an innovation is a function of the characteristics of the innovation and include five important traits. These include relative advantage, the degree to which the innovation is perceived to be superior than its predecessor; compatibility, the degree to which the innovation is perceived to be consistent with current values, past experience and need of potential users; complexity, the perceived degree of difficulty to understand and use the innovation; and trialability, the degree and possibility to experiment with the innovation on a limited basis. He hypothesizes that there is a negative relationship between complexity and adoption of innovations and a positive relationship between adoption of innovations with relative advantage, compatibility, and trialability. Rye and Kimberley [23] in their empirical review of adoption of health innovations identified the need to include the impact of technology acquisition strategies in future studies. Innovations in technologies, especially high-cost technologies, is fundamentally one of the greatest contributing factors in cost of health care and has led to differentiated modes of acquisitions of technologies ranging from lease, rental and joint venture with complex contractual matrices. However, there is limited evidence on how acquisition strategies (either by hospital strategic decision or imposed by vendors with integrated switching costs) affect adoption of health innovation in general and m-health.

The organizational constructs are determined by the five commonly used constructs in empirical studies on IT innovations [13,15]. These include 1) the decision making structure of the organization on adoption of m-health innovations 2) the size of the organization (number of staff and number of patients); 3) human IT competencies (number and quality of staff that could implement m-health), 4) availability of ICT infrastructure (pre-existence of IT departments or IT platforms to support m-health without excessive additional cost); 5) financial resources or organizational slack. Recent studies on strategic management of innovations [24] suggest inclusion of effect of 6) market growth through technological leadership. Most studies investigating adoption of health innovations assumed the silent role of hospitals’ vision, and strategies for market growth [23]. The TOE framework thus hypothesizes that hospitals with less complex, decentralized organizational structures and decision-making processes are more likely to adopt m-health than those that have complex and centralized structures. Hospitals with higher number of staff and patients, with human resources that have advanced IT knowledge, a fully-fledged IT department and IT platform will be more likely to adopt m-health applications. In addition, this study added Porter [24] hypothesis that hospitals that pursue market growth through technological leadership will be more likely to adopt m-health than those that don’t.

The nature and level of competition in the industry, government regulations, consumer readiness and pressure are the most commonly investigated determinants on adoption of IT innovations in the TOE framework [13,25]. However, the nature of decision making on technology adoption in healthcare industry requires inclusion of pushes and pulls from different stakeholders with divergent needs such as professional medical associations, health insurance companies and regulators in an environment that is increasingly affected by global medical tourism [26,27]. This study thus investigated the following 5 constructs as part of environmental determinants of m-health adoption: 1) level of competition and rivalry at country level, effect of global medical tourism 2) government pressure or incentives 3) patient pressure 4) support or resistance from medical professional associations, and 5) support or resistance from health insurance companies.

This study hypothesized that executives that perceive higher level of competition for patients and rivalry in the health care industry will likely adopt m-health. Executives that perceive the threat and opportunities of global medical tourism and borderless healthcare will be more likely to adopt m-health than those that don’t. Positive perceptions of government regulations and incentives will lead to adoption of m-health than negative perceptions. Perceptions of patients’ readiness and pressure to use m-health will lead to more adoption. Similarly support of m-health by professional associations (doctors, nurses and community health workers) and health insurance companies will lead to m-health adoption.

**Fig 1:**
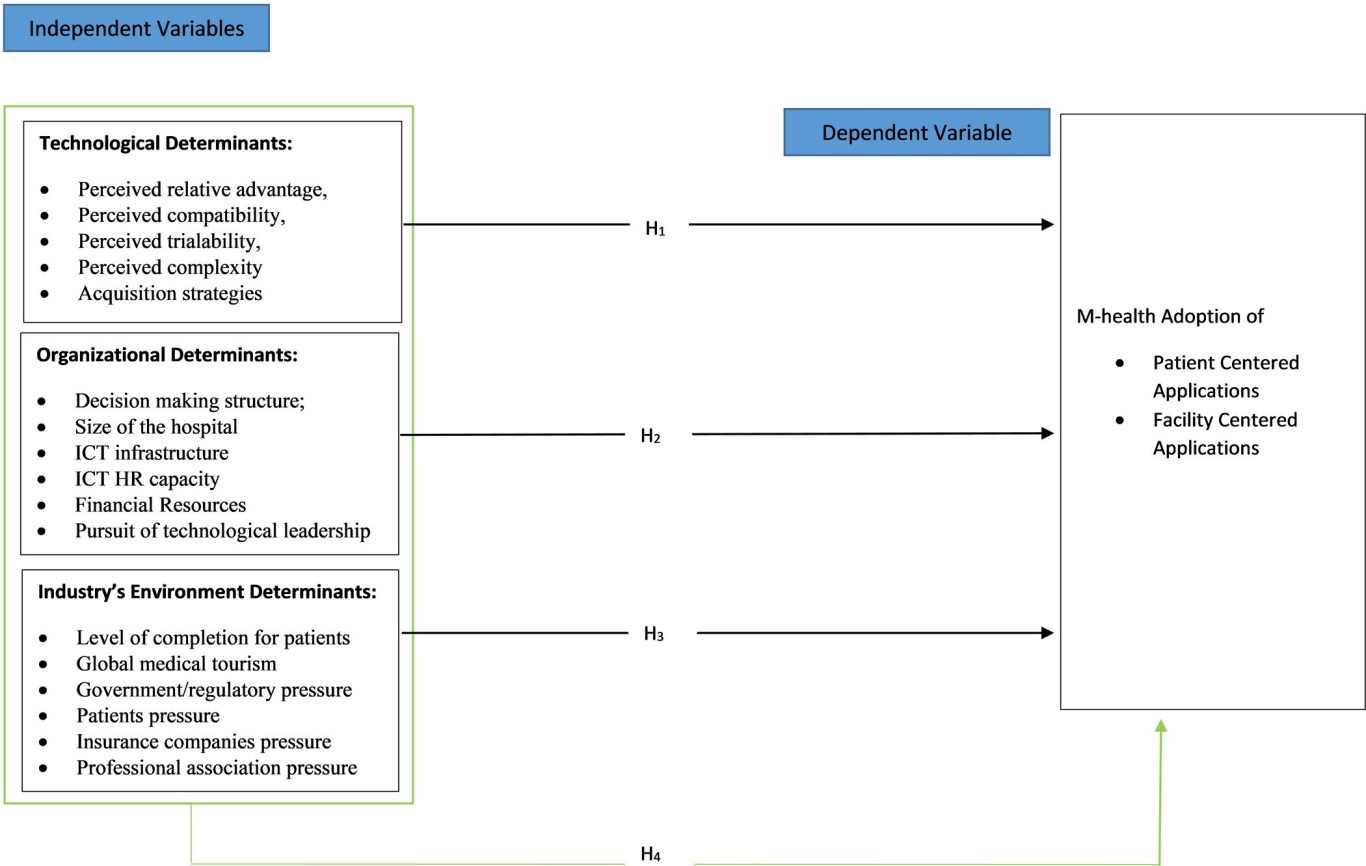
Conceptual framework of TOE determinants and m-health adoption.

Fig 1 below summarizes the summarizes the conceptual framework of m-health adoption and the 17 constructs used in this study.

It also summarizes the four null hypotheses under investigation in this study:

H_o1_ Technological determinants have no statistical significance on m-health adoption by hospitals in Kenya.
Ho2 Organizational determinants have no statistical significance on m-health adoption by hospitals in Kenya.
H_o3_ Environmental determinants have no statistical significance on m-health adoption by hospitals in Kenya
H_o4_ The interaction between all TOE determinants and m-health adoption by hospitals in Kenya have no statistical significance on m-health adoption by hospitals in Kenya.

### Methods

This study is a non-experimental quantitative research with correlational design. Creswell [28] defines correlational design as studies that investigators use correlational statistics to describe, explain or measure the degree or relationships between one or more variables. The most commonly used approach in applied quantitative research is the survey design as it provides a numeric or quantified description of trends or association of variables within a population by studying a sample of that population [28]. The choice of a quantitative correlational survey design is, therefore, appropriate for this study as it is aligned with the aim of testing the statistical significance and relationship of TOE determinants (independent variables) on the adoption of m-health (dependent variable). The general target population of this study are the 507 (N) Top Executives (TEs) or managers of hospitals from levels 4, 5 and 6 registered in the Kenya Master Health Facility data base. This study defines TE as the most senior officer, manager or executive in charge of strategic leadership and management of the hospital. It targeted one top executive per hospital. The choice of top executives as respondents for this study is justified by the fact that they possess both the decision-making power on adoption of health innovations and the accountability for understanding TOE determinants and their implications on hospital’s strategic vision and performances. This study targeted executives of levels 4, 5 and 6 public, private, and Faith Based Organizations (FBO) and/or Non-Governmenal Organizations (NGO) hospitals because they form the main structure of Kenya’s MoH introduction and scale up of policies and innovations. Levels 3 (Health centers), 2 (dispensaries and clinics) and 1 (community levels) are excluded from this analysis as their relevance for m-health and other innovations are often reliant on the adoption of levels 4 to 6 hospitals that they refer their patients to. The ethical approval for the study was obtained through United States International University—Africa and the research approval was given by the National Council on Science, Technology and Innovations (NACOSTI). In view of the nature and scope of the study, the researcher requested also authorizations and recommendation from the MoH at national level and from county health departments. Table 2 below shows the population size of the level 4–6 hospitals with each hospital representing one top executive.

**Table 2:** National distribution of hospitals by categories of ownership and classification of levels.

Types of Hospitals	Public	Private For Profit	FBOs/NGOs	Total
Tertiary Hospitals (level 6)	4			4 (1%)
Secondary Hospitals (level 5)	14	3	1	18 (4%)
Primary hospitals (District or sub-district level 4)	278	139	68	485 (95%)
Total	296 (58%)	142 (28%)	69 (14%)	507

Source: Kenya MoH Master list of hospitals (2017)

Selected facilities were spread across all 47 counties and registered in the MoH database. This study used the census method for the 22 level 5 and 6 hospitals. It used the Slovin formula to select a stratified simple random sampling method for the 485 level 4 hospitals to select a sample of size 219 facilities proportionately distributed across the 47 counties and ownership to derive a total of 126 public health facilities from 278 registered hospitals; 63 private for-profit hospitals from 139 registered facilities, and 30 from FBOs/NGOs.

It used the Slovin Formula with n=N(1+Ne2) to derive the sample size at the margin error of e = 0.05 or a confidence level of 95%. Where n is the sample size and N, the total population. Since N = 485, then n=485(1+485*0.052)=219 facilities.

Table 3 below summarizes the representative sample distribution of level 4 hospitals based on ownership categories.

**Table 3:** Distribution of level 4 hospitals by types of providers/ownership (N = 485).

Types of Hospitals	Population	Sample Size
Public	278	126
Private For-Profit	139	63
FBOs/NGOs	68	30
Total	485	219

The questionnaire was pilot tested and validated in 2 counties (Kiambu and Nairobi) with 20 TEs of public, private and FBO/NGOs hospitals. This study used the Cronbach’s alpha scores to assess the constructs reliability of the instruments in the pilot phase. The outcome of the reliability test was within the recommended range by Field and Miles [29] of alpha between 0.65 and 0.8 as summarized in Table 4 below with results of reliability tests.

**Table 4:** Summary of reliability tests.

Case Processing Summary	N	%	Cronbach’s Alpha	N of Items
Cases Valid	20	100		
Excluded*	0	0		
Total	20	100		
			0.748	76

* Listwise deletion based on all variables in the procedure

Because it adapted the questionnaire used in other studies using TOE/DOI framework, it focuses mostly on content validity as guided by Foxcroft, Paterson, le Roux and Herbst [30]. A team of 15 experienced research assistants based in the selected counties was hired to ensure accuracy of respondents and higher response rate due to their local knowledge of the county systems. The use of emails or e-surveys was not considered as a reliable option for this study due to the low rate of response observed in other studies that targeted executives of hospitals by emails and the potential challenges of excluding rural hospitals that may have limited ICT infrastructures. Data collection was initiated from April to July 2018.

The data analysis was conducted using SPSS 21 to derive Pearson Chi-Square, Likelihood Ratio and Linear-by-Linear Association at 5% of significance level. It collected n explanatory variables that result into two specific outcomes Y = 1 adoption of m-health, Y = 0 rejection of m-health. The logit model used is based on cumulative logistic probability functions, is computational and has the advantage of predicting the probability of the adoption. A Logit Regression Model (LRM) was estimated against the 17 independent variables on the status of adoption or non-adoption of each 12 m-health applications grouped under Patient-Centered or Facility-Centered group (see Table 1). Data was first collected on the 3 FC and 9 PC m-health individual applications thereafter the results were transformed to generate the PC and FC variables while maintaining the adoption measurement scale. The response scale for PC and FC was based on the arithmetic mean of the responses for each variable under each category. The responses recorded are discrete (mutually exclusive and exhaustive) and therefore, adopts univariate logit model to analyze adoption or non-adoption decisions by hospitals through the perspectives of the TEs.

The LRM is thus defined by a latent variable y* which is presented by the relationship in Eq 1 below:

$${\text{y}}^{*}=\beta^{/}{\text{x}}_{\text{ki}}+\xi_{\text{ki}}$$ Eq 1

Where ${\text{x}}_{\text{ki}}$ and $\xi_{\text{ki}}$ are normally distributed with mean and common variance.

From Eq 1 the present study employed a LRM to determine factors influencing adoption of PC and FC m-health applications. The LRM is a probabilistic model that explains the likelihood that one will select to adopt a specific or a combination of m-health applications and takes the following form:

P(Yi=1|xki)=π(Yi) Eq 2

where π (Y) is a nonlinear function of the best combination of the explanatory variables. From Eq 1 above,

Let

$$Z=\beta_{0}+\sum_{i=1}^{n}\beta_{i}X_{i}+\varepsilon$$ Eq 3

where Z is defined as follows:

$$\text{Z}=\text{In}(\frac{\text{p}}{\left(1-\text{p}\right)})$$ Eq 4

Hence from Eqs 3 and 4,

$$\text{In}\left(\frac{\text{p}}{1-\text{p}}\right)=\beta_{0}+\Sigma \;\beta_{i}X_{i=}$$

$$\left(\frac{\text{p}}{1-\text{p}}\right)={\text{e}}^{\beta_{0}+\Sigma \;\beta_{i}X_{ki}}$$

$$\pi \left(Y\right)=P\left[Y=1\right]=\frac{e^{\beta_{0}+\sum_{1}^{18}\beta_{i}X_{i}}}{\left(1+e^{\beta_{0}}{}^{+\sum \;\beta_{i}X_{ki}}\right)}$$ Eq 5

Because the theoretical and mathematical reasoning is based on a linear model of natural logarithm of the odds (the log. odds) in favor of Yi = 1, then the log of the ratio of adoption and non-adoption of m-health applications in this study is shown below as:-

$$\text{In}\left[\frac{\text{p}}{1-\text{p}}\right]=\beta_{0}+\beta_{1}X_{\text{t}.1}+\beta_{2}X_{\text{t}.2}+\beta_{3}X_{\text{t}.3}+\beta_{4}X_{\text{t}.4}+\beta_{5}X_{\text{t}.5}+\beta_{6}X_{\text{o}.1}+\beta_{7}X_{\text{o}.2}+\beta_{8}X_{\text{0}.3}+\beta_{9}X_{\text{e}.1}+\beta_{10}X_{\text{e}.2}$$ Eq 6

To determine the estimated LRM of the TOE determinants, this study conducted first the Omnibus Tests of model coefficients to check if the new models with TOE variables is an improvement of the constant model without TOE variables or determinants at 5% of significance level. It then used the enter method of model fitting which involves the entering of all TOE determinants or variables at the same step for each of the two categories (PC and FC) of m-health innovations and regressed them on the TOE determinants. It also tested the goodness of fit of the two models (PC and FC) using the Cox & Snell square and the Nagelkerke R Square. It used the Hosmer-Lemeshow test to analyze whether the predicted probabilities are the same as the observed probabilities. It finally gave an interpretation of the two models using the coefficients of the TOE determinants at 5% of significance level.

## 2. Results, discussion and conclusion

The total number of questionnaires that met the requirements for the study was 211 out of 241 (219 level 4 hospitals and 22 level 5 and 6 hospitals) distributed questionnaires across 24 counties.This represents 87.5% response rate which complies with recommendations by Fincham [31] that a response rate of 80% and above is needed for generalizability of results of surveys. Four questionnaires were discarded because they were filled by non-top executive staff and 3 questionnaires were discarded because the hospitals self-categorized as level 3 hospitals despite being registered as level 4 in the Kenya Ministry of Health database. The overall distribution of respondents by hospitals ownership was 48% for public, 36% for private and 16% for FBOs/NGOs. The distribution by levels of hospitals was 80% level 4 hospitals, 16% level 5 hospitals and 4% level 6. The geographical distribution of hospitals was 36% urban, 38% semi-urban and 26% rural.

The majority (75%) of TEs who responded were male. Most respondents (61%) were below 40 years of age and the rest were above 40 years of age. Most TEs (53%) have been in their current leadership position at the hospital for between 2 and 4 years, while 21% had served between 5 and10 years, 17% had served less than one year, and 9% had served for more than 10 years. Eleven percent of respondents had attained the diploma level of education, 46% had a first degree at the university level and the remaining 42% had attained post graduate levels.

**Fig 2:**
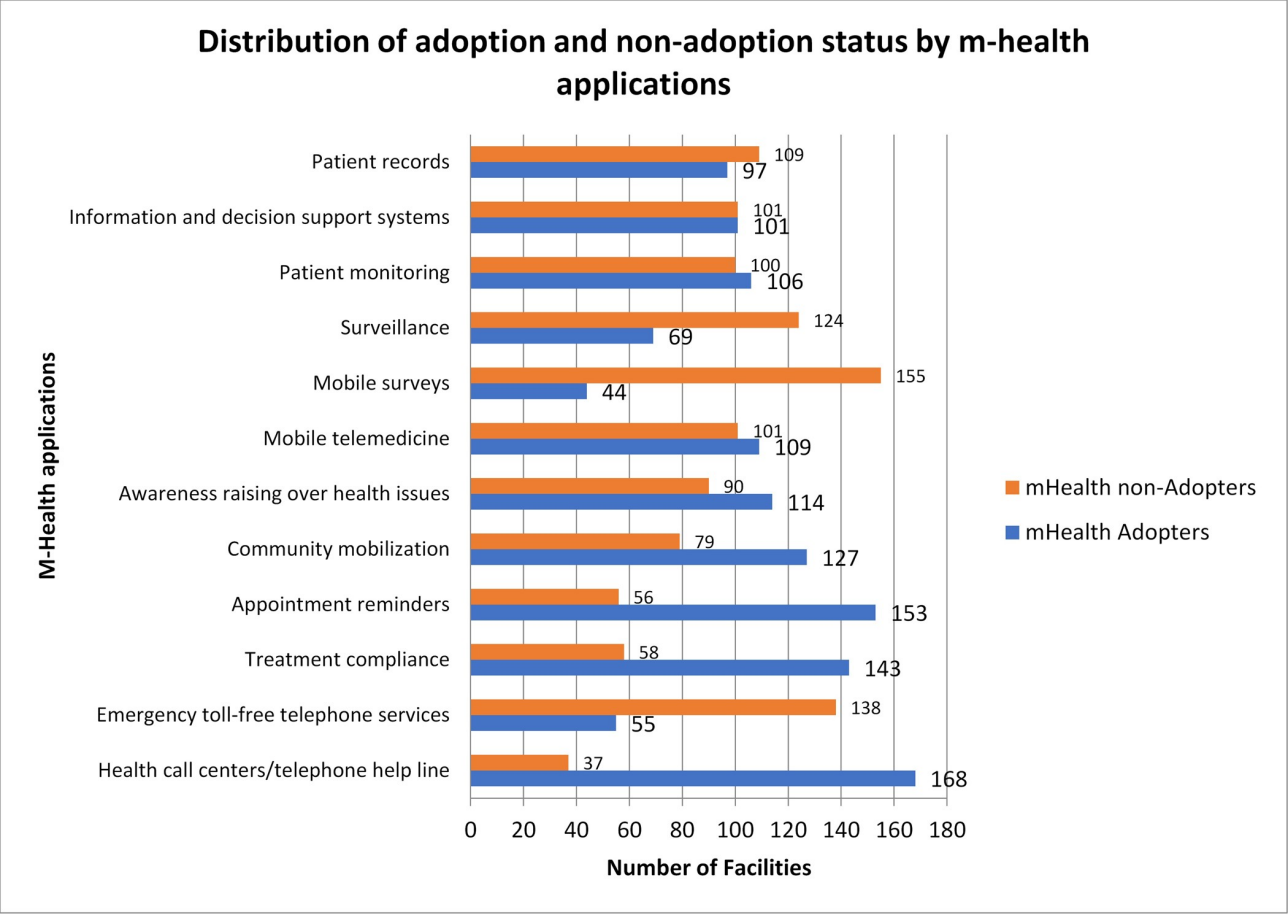
Distribution of adoption of the 12 WHO classified m-health applications. Number of non-adopters of each m-health application designated with an orange square; Number of adopters of each m-health application designated with a blue square.

The distribution of adoption or non-adoption individual m-health applications is shown in Fig 2 below.

The top three adopted applications by majority of the facilities were health call centers/telephone help line, appointment reminders and treatment compliance. The three least used applications out of the twelve were mobile surveys, emergency toll-free telephone services and patient records.

A summary of results based on the two main categories of PC and FC m-health applications, by levels of hospitals, types of ownership and facility is shown in Table 5 below. There are more adopters of PC than FC m-health applications at level 4–6 hospitals. Private and Public hospitals have similar proportion of both PC and FC m-health applications adoption while FBO/NGOs have the highest proportion of PC m-health applications. Overall there is higher proportion of adopters of PC m-health than FC m-health applications. There is a higher percentage of adoption of PC and FC m-health in rural areas than in urban and peri-urban areas.

**Table 5:** Distribution of adoption status by level of hospital, ownership and geographical locations.

		Patient Centered		Facility Centered	
Facility attributes		Non Adopters	Adopters	Non Adopters	Adopters
Hospital Classification	Level IV	54 (42%)	75 (58%)	88 (54%)	75 (46%)
	Level V	7 (28%)	18 (72%)	12 (41%)	17 (59%)
	Level VI	2 (67%)	1(33%)	3 (50%)	3 (50%)
Facility ownership	Public	35 (44%)	45 (56%)	57 (59%)	39 (41%)
	Private	22 (41%)	32 (59%)	31 (44%)	39 (56%)
	FBO/NGO	6 (26%)	17 (74%)	15 (46%)	17 (54%)
Facility Location	Urban	25 (44%)	32 (56%)	38 (54%)	33 (46%)
	Peri-urban	25 (43%)	34 (57%)	40 (53%)	36 (47%)
	Rural	13 (32%)	28 (68%)	25 (49%)	26 (51%)

The results of the four hypotheses tested are presented below.

H_o1_ Technological determinants have no statistical significance on m-health adoption by hospitals in Kenya.

The study used the LRM for Technological determinants below

$$\text{In}\left[\frac{{\text{p}}_{\text{k}}}{1-{\text{p}}_{\text{k}}}\right]=\alpha_{0}+\alpha_{1}{\text{X}}_{1}+\alpha_{2}{\text{X}}_{2}+\alpha_{3}{\text{X}}_{3}+\alpha_{4}{\text{X}}_{4}+\alpha_{5}{\text{X}}_{5}$$ Eq 7

Where for i = 1,2,3,4,5) α_i_ are coefficients of the technological determinants Xi measured as categorical variables and defined as follows: X1 = Relative Advantage, X2 = Compatibility, X3 = Complexity, X4 = Trialability, X5 = Acquisition strategy.

Table 6 below presents the results of the omnibus tests for model coefficients, the goodness of fit summary and the Hosmer-Lemeshow tests for technological determinants for PC and FC m-health applications at 5% level of significance.

**Table 6:** Results of omnibus tests, goodness of fit summary and hosmer-lemeshow test for technological determinants.

m-health models	Omnibus Tests of Model Coefficients			Goodness of Fit Summary			Hosmer-Lemeshow Test		
	Chi-Square	df	Sig(p-value)	-2Log likelihood	Cox&Snell R square	Nagelkerke R square	Chi-Square	df	Sig(P-value)
PC	16.445	5	0.006	162.556	0.115	0.156	1.796	6	0.937
FC	1.156	5	0.949	219.108	0.007	0.010	2.317	4	0.678

The Omnibus results indicate that the PC m-health adoption model with all the predictors added is significantly better than the constant only model [Chi-Square = 16.445, df = 5 and p = 0.006 (<0.05)]. However, the FC model with all independent variables included is not significantly better than its corresponding constant only model [Chi-Square = 1.158, df = 5 and p = 0.949 (>0.05). The results imply that the addition of all the technological determinants/predictors improves the predictive power of the PC adoption model. However, the addition of all technological determinants/predictors does not improve the predictive power of the FC model, implying that the FC model predictive power may be caused by other determinants than technological determinants.

This study used the Nagelkerke R Square for goodness of fit test as it fits the binary models of adoption and because the Cox & Snell test does not scale up to 1. The results of the Nagelkerke R Square PC model show that 15.6% of the variation in the PC adoption model is accounted for by the five technological determinants of relative advantage, compatibility, and complexity, trialability and acquisition strategy. The FC adoption model shows that only 10% of variability is accounted for by the technological determinants. The -2 Log likelihood (goodness of fit test) value for the predictors inclusive of PC model and FC models are 162.556 and 219.108 respectively, which are compared to their respective null models.

The Hosmer-Lemeshow test determines if the predicted probabilities are the same as the observed probabilities. An overall goodness of fit of the model is indicated by p values > 0.05 (Hosmer and Lemeshow, 2000). Since p-values (Sig.) in Table 6 are greater than 0.05 (at the 5% level of significance) for each of the m-health models (PC and FC) and their related adoption decisions, the goodness of fit assumption is confirmed for each model.

Table 7 below, summarizes the significance of predictive model of PC adoption against the 5 technological determinants identified in H1.

**Table 7:** PC m-health application adoption and technological determinants.

Variables in the Equation								
Variables	B	S.E.	Wald	df	Sig.	Exp(B)	95% C.I.for EXP(B)	
							Lower	Upper
Relative Advantage	.065	.625	.011	1	.917	1.068	.314	3.635
Compatibility	.982	.402	5.976	1	.015	2.670	1.215	5.869
Complexity	-.130	.391	.111	1	.739	.878	.408	1.889
Trialability	-2.220	.946	5.511	1	.019	.109	.017	.693
Acquisition strategy	2.182	.860	6.436	1	.011	8.861	1.642	47.802
Constant	-.055	.918	.004	1	.952	.946		

a. Variable(s) entered on step 1: Relative Advantage, Compatibility, Complexity, Trialability, Acquisition strategy.

Therefore, the LRM equation below on technological determinants shows the estimated coefficients for relative advantage, compatibility, complexity, trialability, acquisition strategy.

$$\text{In}\left[\frac{p_{1}}{1-p_{1}}\right]=-0.055+0.065*\text{Relative advantage}+0.982*\text{Compatibility}-0.130*\text{Complexity}-2.220*\text{Trialability}+2.182*\text{Acquisition strategy}$$ Eq 8

Determinants with p values (Sig) less than 0.005 are statistically significant predictors of adoption of m-health applications by hospitals in Kenya. The Wald chi-square statistic tests the unique contribution of each determinant in the context of other predictor variables, to test the conventional 0.05 standard for statistical significance. The results show that the determinants of compatibility, trialability and acquisition strategy with p values (Sig) of 0.015, 0.019 and 0.011 respectively (which are less than 0.005) are significant predictors of adoption of PC m-health applications at the 5% level. However, the determinants of relative advantage (p value of 0.917) and complexity (p value of 0.739) with their p values greater than 0.05 are not significant predictors of PC m-health adoption at the 5% level of significance. The negative value of determinants of trialability (-2.220) and complexity (-0.130) in Table 7, column B above indicate that the executives who agreed that m-health innovations were complex and needed to be tried before adoption were 0.878 and 0.109 respectively less likely to adopt PC m-health applications. The binary logit regression model relating to the likelihood of technological determinants affecting adoption of PC m-health indicates that compatibility, trialability and acquisition strategy are statistically significant predictors of adoption (Chi-Square = 16.445, df = 5 and p = 0.006 (<0.05)). Compatibility (Wald = 5.976, p = 0.015 (<0.05)); trailability (Wald = 5.511, p = 0.019 (<0.05)) and acquisition strategy (Wald = 6.436, p = 0.011 (<0.05)) are significant at the 5% level. The odds ratio (OR) for compatibility is 2.670 (95% CI 1.215–5.869), for trialability is 0.109 (95% CI: 0.017–0.693) and for acquisition strategy is 8.861 (95% CI 1.642–47.802). However, the other two predictors of complexity and relative advantage are not statistically significant.

Table 8 below, summarizes the significance of predictive model of FC adoption against the 5 technological determinants identified in H1. It shows that none of the estimated coefficients are statistically significant (p value > 0.05) with respect to their predictive power on the likelihood of FC m-health adoption. This implies that any changes in the likelihood of adoption may be attributable to other factors.

**Table 8:** FC m-health application adoption and technological determinants.

Variables in the Equation								
Variables	B	S.E.	Wald	df	Sig.	Exp(B)	95% C.I.for EXP(B)	
							Lower	Upper
Relative Advantage	.251	.530	.225	1	.635	1.286	.455	3.632
Compatibility	.108	.351	.095	1	.758	1.114	.560	2.218
Complexity	-.002	.325	.000	1	.995	.998	.528	1.885
Trialability	.213	.590	.131	1	.718	1.238	.390	3.932
Acquisition strategy	.235	.586	.161	1	.688	1.265	.401	3.988
Constant	-.760	.739	1.056	1	.304	.468		

a. Variable(s) entered on step 1: Relative Advantage, Compatibility, Complexity, Trialability, Acquisition strategy.

$$\text{In}\left[\frac{p_{2}}{1-p_{2}}\right]=-0.760+0.251*\text{Relative advantage}+0.108*\text{Compatibility}-0.002*\text{Complexity}+0.213*\text{Trailability}+0.235*\text{Acquisition strategy}$$ Eq 9

In conclusion, this study accepts the null hypothesis that all five technological determinants (perceived relative advantage, perceived compatibility, perceived complexity, perceived trialability and acquisition strategy) have no statistical significance on the likelihood of FC m-health adoption by hospitals in Kenya. However, it rejects the null hypothesis that technological determinants (especially, compatibility, trialability and acquisition strategy with p values of 0.015, 0.019 and 0.011 respectively) have no statistical significance on the likelihood of PC m-health adoption by hospitals in Kenya.

Table 9 below therefore summarizes the model result of hypothesis H1 on technological determinants of m-health adoption by hospitals in Kenya.

**Table 9:** Model result of hypothesis on technological determinants.

Hypothesis	PC m-health applications	FC m-health applications
H01.1 Perceived relative advantage (superiority, efficiency and cost reduction) of m-health has no statistical significance on the likelihood of PC m-health adoption by hospitals in Kenya	Fail to reject	Fail to reject
H01.2 Perceived compatibility (with health information system, required security and confidentiality, HR) of m-health has no statistical significance on the likelihood of PC m-health adoption by hospitals in Kenya	Rejected	Fail to reject
H01.3 Perceived complexity of m-health (difficulty of understanding and use, cost on infrastructure and HR) has no statistical significance on the likelihood of PC m-health adoption by hospitals in Kenya	Fail to reject	Fail to reject
H01.4 Perceived trialability of m-health (trialability for superiority, security to patients and operations) has no statistical significance on the likelihood of PC m-health adoption by hospitals in Kenya.	Rejected	Fail to reject
H01.5 Acquisition strategies of m-health (lease, full ownership) have no statistical significance on the likelihood of PC m-health adoption by hospitals in Kenya	rejected	Fail to reject

Ho2 Organizational determinants have no statistical significance on m-health adoption by hospitals in Kenya.

The study used the LRM below

$$\text{In}\left[\frac{{\text{p}}_{\text{k}}}{1-{\text{p}}_{\text{k}}}\right]=\beta_{0}+\beta_{1}{\text{X}}_{1}+\beta_{2}{\text{X}}_{2}+\beta_{3}{\text{X}}_{3}+\beta_{4}{\text{X}}_{4}+\beta_{5}{\text{X}}_{5}+\beta_{6}{\text{X}}_{6}+\beta_{7}{\text{X}}_{7}$$ Eq 10

Whereas X1 = hospital decision making structure, X2 = size of hospital, X3 = hospital’s ICT infrastructure capacity, X4 = hospital’ ICT HR capacity, X5 = hospital market focus, X 6 = hospital’s slack financial resources, X7 = hospital’s pursuit of market growth through technology leadership,

Table 10 below presents the results of the omnibus tests for model coefficients, the goodness of fit summary and the Hosmer-Lemeshow tests for organizations determinants for PC and FC m-health applications at 5% level of significance.

**Table 10:** Results of omnibus tests, goodness of fit summary and hosmer-lemeshow test for organizational determinants.

m-health models	Omnibus Tests of Model Coefficients			Goodness of Fit Summary			Hosmer-Lemeshow Test		
	Chi-Square	df	Sig(p-value)	-2Log likelihood	Cox&Snell R square	Nagelkerke R square	Chi-Square	df	Sig(P-value)
PC	5.665	7	0.579	204.791	0.27	0.38	2.375	7	0.936
FC	1.736	7	0.973	269.467	0.31	0.52	4.619	8	0.797

The results of Omnibus test show that for both the PC and FC models with all the determinants or predictors added was not significantly better than the constant only model [Chi-Square = 5.665, df = 7 and p = .579 (>0.05) and [Chi-Square = 1.736, df = 7 and p = 0.973 (>0.05) respectively. The results imply that the addition of all organizational determinants does not improve the predictive power of both PC and FC models of adoption of m-health.

The results of goodness of fit models with -2Log likelihood of 204.791 and 269.467 for PC and FC respectively indicate that the model adequately fits the research data. Furthermore, the Nagelkerke R Squared of 0.38 and 0.52 implies that 38% and 52% of the respective changes in adoption of the two interventions are explained by the organizational determinants identified.

The results of the Hosmer and Lemeshow chi-square test for PC model (Chi: 2.375, df = 7, p = 0.936 (>0.05) and FC model (Chi: 4.619, df = 8, p = 0.797 (>0.05) is non-significant indicating that the data fit the model well. Because for both PC (p-value of 0.93) and FC (p-value of 0.79) have p-values greater than 0.05, therefore, the goodness of fit assumption is confirmed for both PC and FC models.

Table 11 below, summarizes the significance of predictive model of PC adoption against the 7 organizational determinants identified in H2.

**Table 11:** PC m-health applications adoption and organizational determinants.

Variables in the Equation								
Variables	B	S.E.	Wald	Df	Sig.	Exp(B)	95% C.I.for EXP(B)	
							Lower	Upper
Decision making structure.	.361	.382	.897	1	.344	1.435	.679	3.033
Size of hospital—patients and staff	.692	.455	2.318	1	.128	1.998	.820	4.870
ICT capacity and infrastructure	-.356	.493	.522	1	.470	.701	.267	1.840
ICT HR Capacity	-.036	.406	.008	1	.048*	.964	.435	2.138
Scope of the Market focus.	-.139	.461	.091	1	.763	.870	.352	2.148
Slack/Financial Resources	-.019	.418	.002	1	.006*	.981	.432	2.227
Technology leadership	-.485	.389	1.552	1	.010*	.616	.287	1.321
Constant	.501	.318	2.485	1	.115	1.650		

*Significant at 5% level of significance

$$\begin{array}{l} \text{In}\left[\frac{p_{1}}{1-p_{1}}\right]=0.501+0.361*\text{Decision making structure}.\;+0.692*\text{Size of hospital}\\ \;\;\;-0.356*\text{ICT capacity and infrastructure}-0.036*\text{ICT HR Capacity}\\ \;\;\;-0.139*\text{Market focus scope}\;-0.019*\text{Slack/financial resources}\\ \;\;\;-0.485*\text{Technolohy leadership} \end{array}$$ Eq 11

The results show that the following organizational determinants are statistically significant predictors of PC m-health adoption: ICT staff knowledge and adequacy (p = 0.048), Slack/Financial Resources (p = 0.006) and pursuit of market grown through technology leadership (p = 0.010). The rest of organizational determinants such as decision-making structure (p = 0.344), size of hospital—(p = 0.128), ICT capacity and infrastructure (p = 0.470) and scope of market focus (p = 0.763) were not statistically significant predictors of PC m-health adoption.

Table 12 below, summarizes the significance of predictive model of FC adoption against the 7 organizational determinants identified in H2.

**Table 12:** FC m-health applications adoption and organizational determinants.

Variables in the Equation								
Variables	B	S.E.	Wald	df	Sig.	Exp(B)	95% C.I.for EXP(B)	
							Lower	Upper
Decision making structure.	.136	.326	.175	1	.676	1.146	.605	2.171
Size of hospital—patients and staff	-.048	.371	.017	1	.008*	.953	.460	1.974
ICT capacity and infrastructure	-.067	.428	.025	1	.041*	.935	.404	2.164
ICT HR capacity	.139	.345	.162	1	.037*	1.149	.584	2.261
Scope of the Market focus.	.176	.395	.198	1	.656	1.192	.550	2.585
Slack/Financial Resources	-.198	.369	.287	1	.592	.821	.398	1.691
Technology leadership	-.364	.330	1.217	1	.020*	.695	.364	1.326
Constant	.030	.271	.012	1	.912	1.030		

$$\begin{array}{l} \text{In}\left[\frac{p_{2}}{1-p_{2}}\right]=0.030+0.136*\text{Decision making structure}.-0.048*\text{Size of hospital}\\ \;\;\;\;-0.067*\text{ICT capacity and infrastructure }+0.13\text{9*ICT HR capacity}\\ \;\;\;\;+0.176*\text{Market focus scope}\;-0.198*\text{Slack/financial resources}\\ \;\;\;\;-364*\text{Technolohy leadership} \end{array}$$ Eq 12

The results of FC m-health adoption indicate that the size of a hospital (p = 0.008), its ICT capacity and infrastructure (p = 0.041), its ICT HR capacity (p = 0.037) and its pursuit of market growth through technology leadership (p = 0.020), were statistically significant predictors of FC m-health adoption. On the other hand, decision making structure (p = 0.676), market focus (p = 0.656), slack/financial resources (p = 0.592) showed statistically non-significant outcomes.

In conclusion, for PC model, ICT HR capacity (p = 0.048), slack financial resources (p = 0.006) and pursuit of market growth through technology leadership (p = 0.010) are statistically significant organizational determinants of PC m-health adoption. For the FC model, size of hospital (p = 0.008), ICT capacity and infrastructure (p = 0.041), ICT HR capacity (p = 0.037) and hospital’s pursuit of market growth through technology leadership (p value of 0.020), were statistically significant predictors of FC m-health adoption.

Table 13 below, therefore, summarizes the model result of hypothesis H2 on organizational determinants of m-health adoption by hospitals in Kenya.

**Table 13:** Model result of the hypothesis on organizational determinants.

	PC in health applications	FC in health applications
H_**02.1**_ Decision making structure on adoption of innovations in hospitals have no statistical significance on PC m-health adoption by hospitals in Kenya	Fail to reject.	Fail to reject.
H_**02.2**_ The size of hospitals (number of staff and patient volume) have no statistical significance on PC m-health adoption by hospitals in Kenya.	Fail to reject.	Rejected
H_**02.3**_ The level of ICT capacity and infrastructure of hospitals have no statistical significance on PC m-health adoption by hospitals in Kenya	Fail to reject.	Rejected
H_**02.4**_ The level of ICT HR capacity (staff knowledgeable and adequacy) of hospitals have no statistical significance on PC m-health adoption by hospitals in Kenya	Fail to reject.	Rejected
H_**02.5**_ The scope of market of the hospital (national, regional and global) have no statistical significance on PC m-health adoption by hospitals in Kenya	Fail to reject.	Fail to reject.
H_**02.6**_ The level of slack resources of hospitals have no statistical significance on PC m-health adoption by hospitals in Kenya	Rejected	Fail to reject.
H_**02.7**_ The pursuit of market growth through technology leadership by hospitals have no statistical significance on PC m-health adoption by hospitals in Kenya.	Rejected	Rejected

H_o3_ Environmental determinants have no statistical significance on m-health adoption by hospitals in Kenya

The study used the LRM below

$$\text{In}\left[\frac{{\text{p}}_{\text{k}}}{1-{\text{p}}_{\text{k}}}\right]=\gamma_{0}+\gamma_{1}{\text{X}}_{1}\gamma_{2}{\text{X}}_{2}+\gamma_{3}{\text{X}}_{3}{+\gamma }_{4}{\text{X}}_{4}+\gamma_{5}{\text{X}}_{5}{+\gamma }_{6}{\text{X}}_{6}$$ Eq 13

Whereas X_1_ = Industry competition for patients, X_2_ = Effect of Global Medical Tourism, X_3_ = Government support, X_4_ = Patients pressure for m-health services, X_5_ = Professional associations support for m-Health, X_6_ = Support from medical health insurance firms.

Table 14 below presents the results of the omnibus tests for model coefficients, the goodness of fit summary and the Hosmer-Lemeshow tests for environmental determinants for PC and FC m-health applications at 5% level of significance.

**Table 14:** Results of omnibus tests, goodness of fit summary and hosmer-lemeshow test for environmental determinants.

m-health categories	Omnibus Tests of Model Coefficients			Goodness of Fit Summary			Hosmer-Lemeshow Test		
	Chi-Square	df	Sig(p-value)	-2Log likelihood	Cox&Snell R square	Nagelkerke R square	Chi-Square	df	Sig(P-value)
PC	11.608	6	0.46	207.848	0.170	0.222	5.693	7	0.576
FC	13.297	6	0.30	265.314	0.170	0.320	1.118	8	0.997

The Omnibus tests results indicate that both the PC model [Chi-Square = 11.608, df = 6 and p = 0.046 (<0.05)] and FC model [Chi-Square = 13.297, df = 6 and p = 0.030 (<0.05)] and the environmental determinants are significantly better than the constant only model.

The results of the Nagelkerke R Square model show that between17% and 22.2% and 17% and 32% of the variation in the PC and FC adoption models respectively are accounted for by the six industry’s environmental determinants. The respective -2 Log likelihood (goodness of fit test) value for the predictors inclusive of the two models were 207.848 and 265.314 indicating that they were significantly lower than the constant only model, which compared well with their respective null models.

The Hosmer-Lemeshow test results for PC model (Chi:5.693, df = 7, p = 0.576) and FC model (Chi:1.118, df = 8, p = 0.997) indicate an overall goodness of fit of the data and the model at 5% level of significance.

Table 15 below, summarizes the significance of predictive model of PC adoption against the 6 organizational determinants identified in H3.

**Table 15:** PC m-health applications adoption and environmental determinants.

Variables in the Equation								
Variables	B	S.E.	Wald	df	Sig.	Exp(B)	95% C.I.for EXP(B)	
							Lower	Upper
Industry competition for patients	-.098	.336	.084	1	.041	.907	.469	1.753
Global Medical Tourism	.447	.677	.436	1	.039	1.563	.415	5.886
Government support/ incentives	.184	.517	.127	1	.957	1.202	.437	3.311
Patients pressure for m-health services	.259	.347	.555	1	.036	1.295	.656	2.559
Professional associations support for m-health as an accepted standard.	.016	.455	.001	1	.997	1.016	.417	2.478
Support from medical health insurance firms	-.490	.417	1.383	1	.240	.613	.271	1.386
Constant	.067	.656	.011	1	.918	1.070		

$$\begin{array}{l} \text{In}\left[\frac{p_{1}}{1-p_{1}}\right]=0.067-0.98*\text{Competion in Industry for patient}+0.447*\text{Global Medical Tourism}\\ \;\;\;\text{by hospitals}+0.185*\text{Government support}+0.259*\text{Patient pressure}\\ \;\;\;+0.016*\text{Professional associations' support}-0.490*\text{Medical health insurance firms' support} \end{array}$$ Eq 14

The results show that majority of the predictors such as industry competition for patients (p = 0.041), global medical tourism by hospitals (p = 0.039) and patients’ pressure for m-health services (p = 0.036) are statistically significant determinants for adoption of PC m-health innovations. On the other hand, government support: in terms of incentives (p = 0.957), support of m-health by professional associations (p = 0.997), and support from medical health insurance firms (p = 0.613) were not statically significant predictors of PC m-health applications.

Table 16 below, summarizes the significance of predictive model of FC adoption against the 6 environmental determinants identified in H2.

**Table 16:** FC m-health applications adoption and environmental determinants.

Variables in the Equation								
Variables	B	S.E.	Wald	df	Sig.	Exp(B)	95% C.I.for EXP(B)	
							Lower	Upper
Industry competition for patients.	.050	.298	.028	1	.021*	1.051	.586	1.885
Global Medical Tourism.	.015	.549	.001	1	.979	1.015	.346	2.974
Government support: in terms of incentives	.521	.438	1.414	1	.013	1.683	.713	3.971
Patients pressure for M-health services	-.239	.307	.606	1	.436	.787	.431	1.438
Professional associations support for m-Health as an accepted standard.	-.431	.422	1.043	1	.307	.650	.284	1.486
Support from medical health insurance firms	.233	.372	.392	1	.025	1.262	.608	2.620
Constant	-.238	.569	.175	1	.676	.788		

* Significant at 5% level of significance

$$\begin{array}{l} \text{In}\left[\frac{p_{2}}{1-p_{2}}\right]=-0.238+0.050*\text{Competion in Industry for patient}+0.015*\text{Global Medical Tourism}\\ \;\;\;\text{by hospitals}+0.521*\text{Government support}-0.239*\text{Patient pressure}\\ \;\;\;-0.431*\text{Professional associatio in support}+0.233*\text{Medical health insurance firms support} \end{array}$$ Eq 15

For FC, industry competition for patients (p = 0.021), government support (p = 0.013) and support from medical insurance (p = 0.025) were statistically significant predictors of adoption of FC m-health application. Furthermore, the results show that the odds of adopting FC m-health applications will increase with the increased level of industry competition for patients ((odds ratio of 1.051 (CI: 0.586–1.885) with a positive beta of 0.050 and a p = 0.021); with government support (odds ratio of 1.683 (CI:0.713–3.971) with a positive beta of 0.521 and a p = 0.013); and with support from medical insurance companies ((odds ratio of 1.262 (CI:0.608–2.620) with a positive beta of 0.233 and p = 0.025).

Table 17 below, therefore, summarizes the model result of hypothesis H3 on environmental determinants of m-health adoption by hospitals in Kenya.

**Table 17:** Model result of hypothesis on industry’s environmental determinants.

	PC m-health applications	FC m-health applications
H03.1 Perception of level of industry’s competition for patients has no statistical significance on PC m-health adoption by hospitals in Kenya.	Rejected.	Rejected.
H03.2 Perception of level of impact of global medical tourism (borderless health care services) on competition has no statistical significance on m-health adoption by hospitals in Kenya.	Rejected	Fail to reject
H03.3 Perception of level of government and counties’ support for m-health has no statistical significance on m-health adoption by hospitals in Kenya.	Fail to reject	Rejected.
H03.4 Perception of level of pressure from patients for m-health services has no statistical significance on PC m-health adoption by hospitals in Kenya.	Rejected.	Fail to reject.
H03.5 Perception of the level of support for m-health by medical professional associations has no statistical significance on PC m-health adoption by hospitals in Kenya.	Fail to reject.	Fail to reject.
H03.6 Perception of the level of support for m-health by medical insurance companies has no statistical significance on PC m-health adoption by hospitals in Kenya.	Fail to reject	Reject

H_o4_ The interaction between all TOE determinants and m-health adoption by hospitals in Kenya have no statistical significance on m-health adoption by hospitals in Kenya.

The LRM below was conducted to assess the impact of interaction of the TOE:

$$\text{In}\left[\frac{{\text{p}}_{\text{k}}}{1-{\text{p}}_{\text{k}}}\right]=\omega_{0}+\omega_{1}{\text{X}}_{1}+\omega_{2}{\text{X}}_{2}+\omega_{3}{\text{X}}_{3}+\omega_{4}{\text{X}}_{4}$$ Eq 16

Where for i = 1,2,3…‥6) $\gamma_{\text{i}}$are coefficients of the combined TOE determinants X_i_ measured as categorical variables and defined as follows: X_1_ = Technological, X_2_ = Organizational, X_3_ = Environmental, and X_4_ = Technological* Organizational* Industry’s Environment.

P_k_ = is the likelihood of adopting the k^th^ (for k = 1,2) m-health technological category 1 = PC m-health innovations and 2 = FC m-health innovations.

Tables 18 and 19 below show the corresponding results for PC and FC m-health applications.

**Table 18:** PC m-health applications on adoption and combined TOE effect variables.

Variables in the Equation								
Variables	B	S.E.	Wald	df	Sig.	Exp(B)	95% C.I.for EXP(B)	
							Lower	Upper
Technology	1.588	1.091	2.116	1	.146	4.893	.576	41.546
Organization	-.204	.384	.283	1	.595	.815	.384	1.730
Environment	.690	.840	.676	1	.411	1.994	.385	10.337
Environment by Organization by Technology	-3.711	1.784	4.327	1	.038	.024	.001	.807
Constant	.539	.306	3.091	1	.079	1.714		

$$\begin{array}{l} \text{In}\left[\frac{p_{2}}{1-p_{2}}\right]=0.539+1.588*\text{Technology}-0.204*\text{Organization}+0.690*\text{Environment}-3.711\\ \;\;\;\;\;\;*\left(\text{Technology}*\text{Organization}*\text{Environmental Determinants}\right) \end{array}$$ Eq 17

The results of the PC model show that the combined TOE with p = 0.038 is statistically significant predictor of adoption of PC m-health. While it shows that the odds of adopting the PC m-health innovations will increase (column B) with improvement of technological and industry’ environmental determinants, albeit not in a statistically significant way by multiples of 4.893 and 1.994 respectively (coefficient EXP(B)), the odds will decrease by 0.815 and 0.024 (coefficient EXP(B)) with changes in the organizational and interaction effect of TOE.

**Table 19:** FC m-health applications on adoption and combined TOE effect variables.

Variables in the Equation								
Variables	B	S.E.	Wald	df	Sig.	Exp(B)	95% C.I.for EXP(B)	
							Lower	Upper
Technology	-.106	.634	.028	1	.867	.899	.260	3.114
Organization	.168	.344	.240	1	.625	1.183	.603	2.322
Environment	.502	.670	.562	1	.453	1.652	.445	6.137
Environment by Organization by Technology	-1.449	1.466	.976	1	.323	.235	.013	4.159
Constant	-.214	.280	.582	1	.445	.808		

$$\begin{array}{l} \text{In}\left[\frac{p_{2}}{1-p_{2}}\right]=-0.214-106*\text{Technology}+0.168*\text{Organization}+0.502*\text{Environment}-1.449*\\ \left(\text{Technology}*\text{Organization}*\text{Environmental Determinants}\right) \end{array}$$ Eq 18

The findings indicate that none of the coefficient’s predictors are statistically significant for FC m-health as can be seen from the p-values (>0.05). This suggests that for the FC model, the interaction of all TOEs would not have a significant contribution to the adoption or non-adoption of FC m-health applications. Table 20 below summarizes the outcome of the hypothesis on combined TOE determinants.

**Table 20:** Summary of the TOE model results of the hypotheses.

	PC m-health applications	FC m-health applications
H_05.1_ The interaction between TOE determinants and the likelihood of PC m-health adoption by hospitals in Kenya are not statistically significant	Reject.	Fail to reject.

The overall result of all the hypotheses is thus presented in the Table 21 below.

**Table 21:** A summary of the TOE model results of the hypotheses.

Hypotheses	Results for PC m-health applications	Results for FC m-health applications
H_**o1**_ Technological determinants have no statistical significance on m-health adoption by hospitals in Kenya	Rejected	Failed to Reject
H_o2_ Organizational determinants have no statistical significance on the likelihood of m-health adoption by hospitals in Kenya.	Rejected	Rejected
H_o3_ Industry’s environmental determinants have no statistical significance on m-health adoption by hospitals in Kenya	Rejected	Rejected
H_o4_ The interaction between TOE determinants and the likelihood of m-health adoption by hospitals in Kenya are not statistical significant.	Rejected	Failed to Reject

This study found that technological determinants had no statistical significance on the likelihood of FC m-health adoption by hospitals in Kenya while it found that compatibility, trialability and acquisition strategy have statistical significance on the likelihood of PC m-health adoption by hospitals in Kenya. There is thus a significant difference on the effect of technological determinants based on the target of m-health innovations. Adoption of PC m-health innovations are better explained by technological determinants while adoption of FC m-health innovation is explained by other determinants than technological. This study thus concurs with the findings byLee et al [32] and Rogers [14] on technological determinants that also demonstrated a significant positive relationship between trialability and adoption of IT innovation. It also concurs with the findings by Buonanno et. al. [33] that found insignificant effect of complexity on adoption of IT innovations. It also supports the findings of Hoti [13] that found significant effect of compatibility in the adoption of IT innovations and the study of adoption of e-commerce in Kenya by Ochola [22] which found a positive and significant relationship between e-commerce and the attributes of relative advantage, compatibility, trialability and observability in Kenya. However, this study differs partially with findings from Hoti’s [13] review of empirical studies on adoption of digital innovations (such as e-commerce) that found that the top three statistically significant technological determinants for adoption of IT innovations to be relative advantage, compatibility and complexity. Except for compatibility, this study found trialability and acquisition strategies to be statistically significant in adoption of m-health by hospitals in Kenya. This could be explained by the uniqueness of the health sector where trialability of health innovation is critical to minimize harm to patients and where costly acquisition of health care innovations and patient confidentiality require a differentiated approach to innovation acquisitions. To increase adoption and minimize failure rates of PC m-health innovations, governments and vendors of m-health innovations should ensure increased compatibility of products with existing national health information systems, provide a scope for cost-free trial phases by hospitals, and introduce differentiated modes of acquisition strategies of technologies that provide more ownership of technologies and data to hospitals and minimize operational costs.

This study also found that out of the 6 organizational determinants for PC model, only ICT HR capacity, slack financial resources and pursuit of market growth through technology leadership were statistically significant for adoption. It also found that for the FC model, size of facility, ICT capacity and infrastructure, ICT HR capacity and market growth through technology leadership, were statistically significant as predictors of m-health adoption. This is consistent with other studies that found a significant effect of slack resources on adoption of IT innovations [21,32,34–36]. It also supports studies that found the significance of HR capacity and IT infrastructure in adoption of e-commerce [22,37]. However, these findings differ from other studies in North America and Asia that did not find any significant association between ICT infrastructure and ICT HR capacity with adoption of IT innovations [38,39]. These countries maybe characterized by hospitals with enough slack financial resources, adequate and qualified IT specialist in-house or outsourced which is not the case for most hospitals in Kenya and in Sub-Saharan Africa in general. The combination of lack of adequate ICT capacity and HR ICT capacity significantly explain the reluctance of adoption of both PC and FC m-health innovations. The perception of how m-health could contribute to market growth was also positively correlated with adoption but has not been explored in other studies. Therefore, sustainable scale up of m-health in Kenya and other Sub-Saharan African countries is dependent on not only establishing compatible, trialable and cost-effective acquisition strategies but also with significant investments in ICT infrastructure, adequate ICT human resources and integration of market growth strategies alongside impetus for adoption of m-health. The approach for scale up and segmentation of FC m-health applications (which may be more resource intensive) should prioritize amongst other things the facility size, slack resources, ICT infrastructure, ICT human resources and vision of technology leadership of executives of hospitals.

This study found that for PC model, environmental determinants that are statistically significant in adoption of PC m-health included: industry competition for patients, effect of global medical tourism by hospitals and pressure from patients for m-health services. For FC model, environmental determinants that are statistically significant in adoption of FC m-health included: industry competition for patients, government support and support from medical insurance companies. This study corroborates findings from other studies that identified a positive correlation between competition and innovation adoption [35,36,40,41]. It also concurs with findings by Hwangand and Christensen [11] and Herlzinger [42] that found that lack of support by health insurance companies had an impact on adoption of health innovations. However, it differs with studies that did not find any association between the level of competition and adoption of ICT innovations [34]. It also concurs with other studies on e-commerce that found a positive and significant correlation with government support and incentives [32,35,36,38–40,43]. The study also concurs with Rye and Kimberley [23] that found that pressure from patients to be significant in adoption of health innovations. However, this study differs from other studies that found that resistance from medical associations significantly contributed to the slow spread of health innovations [44,45].

In summary, the findings of this study demonstrate that different TOE determinants affect PC and FC m-health adoption by hospitals in Kenya. The study, therefore, proposes a differentiated approach to policies and practices to effectively scale up adoption of m-health and reduce failure rates of m-health projects in Kenya and in other Sub-Saharan Africa countries.

For PC m-health applications, the policy priorities should be on establishing compatible and interoperable mechanisms of m-health at national and regional levels; developing processes and validation mechanisms for trialability of m-health applications; incentivize mobility of and competition for patients, establishing innovative and cost-effective acquisition strategies of m-health technologies by hospitals; and ensuring integration of m-health and other digital services with national insurance schemes and policies. Such policies should be supported by a strong community and patients’ education to drive patients demand for m-health services and by enough investment in adequate IT human resources to maintain the quality and reliability of m-health services. Sustainable scale up of m-health by hospitals will be ensured when hospitals integrate m-health as part of their market growth strategies and when pilot projects prioritize hospitals with slack financial resources. To increase adoption of FC m-health applications that are more resources intensive and complex than PC, policy priorities should be on ensuring the appropriate level of government incentives to trigger hospitals investments; national investment in more reliable ICT infrastructure, policies to improve mobility of or level of competition for patients. Unlike for PC, the size of hospitals (number of staff and number of patients), should be prioritized in pilot projects and scale up programs.

This study also provided unique contributions to the theoretical and conceptual frameworks of adoption of health innovations in the context of LMICs. It provides strong evidence for inclusion of determinants of acquisition strategies of health innovations, the pursuit of market growth through technology leadership, the level of competition for patients and pressure from patients/clients in future studies and conceptual frameworks on digital health adoption. It also shows the uniqueness of the health sector by demonstrating the significance of support by medical health insurance companies and the effect of global medical tourism on adoption of m-health.

While these findings and recommendations contribute to the knowledge gap on determinants of adoption of m-health, they also represent limitations that are important to consider. For instance, this study did not approach a longitudinal view of adoption of innovations in view of the nascent nature of m-health adoption in Kenya. It also did not separate the different processes and stages of decision making on adoption (e.g. considering adoption or adopted then rejected) but instead chose a binary approach (adopted or not adopted) to analyze the current status of adoption and related determinants. Furthermore, this study did not evaluate the effect of network externalities (adoption of m-health by one hospital because others within the network or geographical locations have adopted) and did not explore the effect of sources of funding of m-health applications (self-funding vs. donor-funding and government funding). It also did not explore the effect of prior competence on adoption of other digital health and/or mobile financing innovations on adoption of m-health innovations. Further research to investigate these effects in the evolving context of digital health adoption and uniqueness of the health care setting in Sub-Saharan Africa are urgently needed.

In conclusion, a differentiated approach is required to scale up adoption of m-health by hospitals. To effectively scale up PC m-health applications, policy makers should develop and enforce policy frameworks that not only tackle compatibility and interoperability at national level and regional level, but also that develop mechanisms for trialability and validation of m-health applications within the national and regional health systems. Policy makers, health practitioners, digital health investors and health insurance companies should collaborate to co-create a sustainable digital health economy that stimulates both the supply and demand sides of the PC digital health system. To develop a sustainable demand for PC requires significant investment in education and in policy changes that incentivize patient and community demand for digital health services; patient access to quality digital health services across hospitals and national borders; and integration of m-health and other digital health services into public and private insurance schemes. However, to ensure that PC digital health applications such as m-health become catalytic in achieving the sustainable development goals in Sub-Saharan Africa, demand for m-health services and other digital health services will need more than effective policy framework and demand creation. Adequate investment is required to support hospitals acquire or share ICT technical support to maintain reliability and confidence of patients and communities and to improve likelihood of TEs to adopt m-health and other digital technologies.

To increase adoption and scale of FC m-health or other digital health applications, policy makers, practitioners and investors should prioritize hospitals with large volume of patients and staff to minimize failure rate. For FC m-health applications to become catalytic in achieving sustainable development goals, priority should be given on creating tax incentives for hospitals and investors that invest in costly FC m-health or other FC digital health technologies and in establishing adequate national and regional ICT infrastructure. However, considerations should be given on the unequal distribution of ICT infrastructure, HR resources and the unique characteristics of hospitals operating in different markets (e.g. rural, semi-urban and urban) to ensure equitable but cost-effective scale up of FC m-health.

## Supporting information

S1 File Questionnaire. (DOCX)

S2 File Data set. (DOCX)

S3 File Raw SPSS data. (DOCX)
