# Supplementary material for: The technological, organizational and environmental determinants of adoption of mobile health applications (m-health) by hospitals in Kenya
Source: PLoS One. 2019 Dec 13;14(12):e0225167. doi: 10.1371/journal.pone.0225167 (PMC6910672; doi:10.1371/journal.pone.0225167)
Supplement: S1 File — (DOCX) [file pone.0225167.s001.docx]

# APPENDIX 1: QUESTIONNAIRE

**QUESTIONNAIRE ON DETERMINANTS OF ADOPTION OF MOBILE HEALTH (M-Health) INNOVATIONS IN KENYA**

**Goal:** This study investigates the Technological, Organizational and Industry’s Environment determinants of M-Health adoption in Kenya through the lenses of Executives of Hospitals in Kenya.

**M-Health -** The Global Observatory for eHealth defined mobile health as medical and public health practice supported by mobile devices, such as mobile phones, patient monitoring devices, personal digital assistants (PDAs), and other wireless devices. This study focuses on the categories of m-health applications as defined by the World Health Organization (2011).

**Ethical approval**: This study has received ethical approval by NACOSTI and is in compliance of doctorate in Business Administration at United States International University – Africa (USIU-A). Your answers will be confidential and will be used only for the purpose of this study. Any errors or misunderstanding therefore shall be the responsibility of the researcher and not yours or those of your organization.

**Instructions** for filling the questionnaire: Please, answer all questions as honestly and candidly as possible. Please, tick the provided box in the section of options provided. When questions require explanations, kindly provide concise answers related to the question or use the section on other comments.

**Time estimates:** It is estimated that filling this questionnaire will take 30 to 45 minutes.

**Part I: Executives Characteristics**

1. **What is your position in the hospital?**

🗆 Chief Executive Officer or equivalent 🗆 Medical Superintendent

🗆 Chief Finance Officer or equivalent 🗆 Chief Marketing officer or equivalent
🗆 Other (please specify)

1. **For how long have you been in this position?**

🗆 Less than a year 🗆 More than a year **🗆** More than five years **🗆** More than 10 years

1. **Sex** 🗆 Male 🗆 Female
2. **Age** 🗆 21-30 🗆 31-40 🗆 41-50 🗆 51-60 🗆 Over 60 years
3. **What is your highest level of education attained?**

🗆 Undergraduate 🗆 Post-graduate/Masters 🗆 Doctorate/PhD 🗆 Other (please, specify)

1. **How would you rate your own knowledge of M-Health?**

🗆 High 🗆 Medium 🗆 Low 🗆 None 🗆 Other, specify

**Part II. Organizational Characteristics**

1. **What is the age of your hospital?**

🗆 Less than 10 years 🗆 10-20 years 🗆 20-30 years 🗆 30-40 years 🗆 More than 40 years

1. **Which level is your hospital classified under in the Government of Kenya (GoK) classification?**

🗆 Level 4 🗆 Level 5 🗆 Level 6 🗆 Other (please specify)

1. **Which category is your hospital classified under in the GoK classification?**

🗆 Public Hospital 🗆 Private Hospital 🗆 Faith-Based Hospital 🗆 NGO 🗆 Other (please specify)

1. **Which geographical category is your hospital classified under in the GOK classification?**

🗆 Urban 🗆 Semi-Urban 🗆 Rural

1. **What is the current number of staff in the hospital?**

🗆 Less than 100 🗆 100-200 🗆 201-300 🗆 301-500 🗆 501 and above

1. **What is the current annual number of patients (outpatients and in-patients) seen in the hospital?**

🗆 Less than 10,000 🗆 10,000-20,000 🗆 20,001-30,000 🗆 30,001-40,000 🗆 40,001-50,000
🗆 50,001 and above

1. **Please indicate the average medical consultation fee that your hospital charges for outpatient services**

🗆 Less than 200 🗆 200-500 🗆 501-1,000 🗆 1,001-1,500 🗆 1,501-2,000 🗆 2,001 and above

1. **Please indicate the current turnover of the hospital per annum**

🗆 Less than 100,000,000 🗆 100,000,000-200,000,000 🗆 200,000,001-400,000,000
🗆 400,000,001-600,000,000 🗆 600,000,001-800,000,000 🗆 800,000,000 and above

1. **Please indicate your current target market and focus (tick the top 3 target markets that the hospital is currently actively pursuing)**

🗆 County 🗆 Country (Kenya) 🗆 Eastern Africa 🗆 Continental Africa 🗆 Global

1. **Please, indicate the extent to which your hospital pursues market growth through technology leadership (as indicated in its vision and mission)**

🗆 High 🗆 Medium 🗆 Low 🗆 None

1. **What is the level of Information Communication and Technology (ICT) infrastructure in the hospital that can/could accommodate implementation of M-health innovations?**

🗆 High 🗆 Medium 🗆 Low 🗆 None

1. **What is the level of ICT human capacity in the hospital as it relates to effective implementation of M-Health?**

🗆 High 🗆 Medium 🗆 Low 🗆 None

1. **What kind of Mobile Financing has the hospital adopted? Please, tick all that applies.**

🗆 Mobile payment (e.g. Mpesa, Airtel Money, etc.) 🗆 Online or Phone banking payment

🗆 Mobile healthcare financing 🗆 Pre-paid Mobile phone saving accounts

🗆 Others, please specify

**Part III Adoption Status**

1. **Which of the following areas of M-Health applications is your hospital currently using?**

| **Categories** | **Applications** | **Yes** | **No** |
| --- | --- | --- | --- |
| *Use of mobile phones or devices for communication between individuals and health services*. | *Health call centers/telephone help line* |  |  |
|  | *Emergency toll-free telephone services* |  |  |
| *Use of mobile phones or devices for communication between health services and individuals.* | *Treatment compliance* |  |  |
|  | *Appointment reminders* |  |  |
|  | *Community mobilization* |  |  |
|  | *Awareness raising over health issues* |  |  |
| *Use of mobile phones or devices for consultation between health care professionals* | *Mobile telemedicine* |  |  |
| *Use of mobile phones or devices for health monitoring and surveillance* | *Mobile surveys (surveys by mobile phone)* |  |  |
|  | *Surveillance* |  |  |
|  | *Patient monitoring* |  |  |
| *Use of mobile phones or devices for access to information for health care professionals at point of care* | *Information and decision support systems* |  |  |
|  | *Patient records* |  |  |

1. **If you are using any M-health technologies or platforms, please, indicate the mode of acquisition that the hospital used:**

🗆 Leased 🗆 Fully bought and owned 🗆 Not Applicable 🗆 Others, please specify

1. **If you are not using any M-Health technology or platforms, please, indicate which mode of acquisition would be conducive for your decision to adopt M-health**

🗆 Leased 🗆 Fully bought and owned 🗆 Others 🗆 Not Applicable

**Part IV Technological Determinants**

1. **Rate the extent to which you agree with the following statements relating the effect of the technological determinants listed below on adoption or non-adoption of M-Health using 1 = Strongly Disagree, 2=Disagree, 3=Agree and 4=Strongly Agree.**

|  | **RATING** | | | |
| --- | --- | --- | --- | --- |
| **Determinants** | **1** | **2** | **3** | **4** |
| **M-Health Characteristics** |  |  |  |  |
| **Relative advantage:** |  |  |  |  |
| Use of M-health is superior and more advantageous than current manual practices. |  |  |  |  |
| M-Health improves efficiency of hospital’s operations |  |  |  |  |
| M-health will reduce the cost of health care to patients |  |  |  |  |
| **Compatibility:** |  |  |  |  |
| M-Health is compatible with current hospital’s health information system and consistent with its values and needs. |  |  |  |  |
| M-Health can be trusted in terms of its security and confidentiality of patient information |  |  |  |  |
| M-Health will make many key staff redundant |  |  |  |  |
| **Complexity:** |  |  |  |  |
| M-Health is difficult to understand, use and integrate in the hospital systems and operations |  |  |  |  |
| Adoption of M-Health innovations requires exorbitant cost in infrastructure and human resources development |  |  |  |  |
| **Trialability:** |  |  |  |  |
| M-Health needs to be piloted first in order to demonstrate that it is better than using current manual systems |  |  |  |  |
| M-Health can be piloted without serious negative impact to patients and hospital operations |  |  |  |  |
| **M-Health Acquisition strategies** |  |  |  |  |
| It is more strategic to adopt M-Health when the technology is leased to the hospital |  |  |  |  |
| It is more strategic to adopt M-Health when the hospital fully owned the technology |  |  |  |  |

**Part V: Organizational Determinant of M-Health Adoption**

1. **Rate the extent to which you agree with the following statements relating the effect of the organizational determinants listed below on adoption of M-Health using 1 = Strongly Disagree, 2=Disagree, 3=Agree and 4=Strongly Agree.**

|  | **RATING** | | | |
| --- | --- | --- | --- | --- |
| **Determinants** | **1** | **2** | **3** | **4** |
| **Organizational Characteristics** |  |  |  |  |
| **Decision making structure:** decision to adopt or not to adopt M-Health adoption is the prerogative of the hospital’s top management only. |  |  |  |  |
| **Size:** M-Health adoption is appropriate only when the hospital has substantial volume of patients and staff to justify its adoption |  |  |  |  |
| **ICT capacity:** M-Health adoption is appropriate when hospitals have a very complex ICT infrastructure |  |  |  |  |
| **ICT staff :** M-Health adoption is appropriate when hospitals have very knowledgeable and adequate number of staff in ICT |  |  |  |  |
| **Scope of the Market**: M-Health adoption is appropriate for hospitals with larger market scopes such as national, regional or global markets. |  |  |  |  |
| **Slack/Financial Resources:** M-health adoption is appropriate for hospitals that have excess budgets to invest in new IT technologies (soft and hard ware) such as M-Health |  |  |  |  |
| **Technology leadership:** M-Health is appropriate for hospitals that pursue market growth through technology leadership |  |  |  |  |

**Part VI. Industry’s Environment Determinants of M-Health Adoption**

1. **Rate the extent to which you agree with the following statements relating the effect of the industry’s environment determinants listed below on adoption of M-Health using 1 = Strongly Disagree, 2=Disagree, 3=Agree and 4=Strongly Agree.**

|  | **RATING** | | | |
| --- | --- | --- | --- | --- |
| **Determinants** | **1** | **2** | **3** | **4** |
| **Industry’s Environment determinants** |  |  |  |  |
| **Industry competition:** Decision to adopt M-Health is appropriate when the hospital is facing high level of competition for patients. |  |  |  |  |
| **Global Medical Tourism**: The increased pace of medical tourism and borderless health care services requires strategic adoption of M-Health by hospitals. |  |  |  |  |
| **Government support:** Government and counties’ incentives (such as tax cuts) is important for adoption of M-Health. |  |  |  |  |
| **Patients pressure:** Decision to adopt M-Health was (or will be) driven by patients’ demand for M-health services |  |  |  |  |
| **Support from professional associations:** Decision to adopt M-health was (will be) dependent on support from medical professional associations as an accepted standard. |  |  |  |  |
| **Support from medical health insurance:** Adoption of M-Health was (will be) dependent on health insurance companies approval and payment of claims for services rendered through M-Health |  |  |  |  |

1. **Indicate the extent to which you agree with the following statements using 1=strongly disagree.....4= strongly agree.**

| **Options** | **1** | **2** | **3** | **4** |
| --- | --- | --- | --- | --- |
| M-Health will disrupt the way healthcare is delivered in Kenya within the next 10 years |  |  |  |  |
| M-health will increase competition among Kenya hospitals and with international hospitals |  |  |  |  |
| M-health has the potential to reduce the cost of health care to the patient |  |  |  |  |
| Kenya is well-positioned to become the leader in M-health as it did with Mobile Financing |  |  |  |  |
| M-health will reduce the ability of hospitals to keep their customer-bases and to insulate themselves against the competition |  |  |  |  |
| M-Health innovations present more risks than they offer opportunities |  |  |  |  |

**PART VII: LEADERSHIP STYLE OF HOSPITAL MANAGEMENT BODIES**

1. **This section provides statements that best describe the leadership style used by boards or oversight committees of your hospital as it moderates adoption of m-health. Please rate the extent to which your board or similar oversight bodies exhibit the following leadership** **styles/characteristics using a 7 point likert scale (1=never to 7= always)**

|  | Rating | | | | | | |
| --- | --- | --- | --- | --- | --- | --- | --- |
| LEADERSHIP STYLES | 1 | 2 | 3 | 4 | 5 | 6 | 7 |
| The leadership of my board fosters trust, involvement and cooperation that is necessary for adoption of m-health |  |  |  |  |  |  |  |
| The leadership of my board instills pride and respect in others that is necessary for adoption of m-health |  |  |  |  |  |  |  |
| The leadership of my board practices what it preaches that is necessary for adoption of m-health |  |  |  |  |  |  |  |
| The leadership of my board encourages thinking about problems in new ways and questions assumptions that is necessary for adoption of m-health |  |  |  |  |  |  |  |
| The leadership of my board treats me as an individual, supports and encourages my development that is necessary for adoption of m-health |  |  |  |  |  |  |  |
| The leadership of my board is clear about organizational values and communicates them to me and staff that is necessary for adoption of m-health |  |  |  |  |  |  |  |
| The leadership of my board gives me encouragement and recognition that is necessary for adoption of m-health |  |  |  |  |  |  |  |
| The leadership of my board communicates a clear and positive vision of the future that is necessary for adoption of m-health |  |  |  |  |  |  |  |
| The leadership of my board inspires by being highly competent that is necessary for adoption of m-health |  |  |  |  |  |  |  |

1. **What are your recommendations for ensuring adoption of m-health innovations in Kenya? (Please, be as specific and concise as possible)**

**Thank you for taking your time and contribution in this study**
