# Supplementary material for: The technological, organizational and environmental determinants of adoption of mobile health applications (m-health) by hospitals in Kenya
Source: PLoS One. 2019 Dec 13;14(12):e0225167. doi: 10.1371/journal.pone.0225167 (PMC6910672; doi:10.1371/journal.pone.0225167)
Supplement: S2 File — (DOCX) [file pone.0225167.s002.docx]

1. **What is your position in the hospital?**

| **Position of respondent in the hospital * Category of hospital classification Crosstabulation** | | | | | |
| --- | --- | --- | --- | --- | --- |
| Count | | | | | |
|  | | Category of hospital classification | | | Total |
|  |  | Public Hospital | Private Hospital | Faith-Based Hospital/NGO |  |
| Position iof respondent n the hospital | CEO or equivalent | 5 | 29 | 6 | 40 |
|  | Medical Superitendant | 62 | 5 | 6 | 73 |
|  | Medical or Clinical officer in Charge | 5 | 5 | 2 | 12 |
|  | Nurse in Charge | 11 | 4 | 3 | 18 |
|  | Chief Finance Officer | 3 | 13 | 9 | 25 |
|  | Chief HR officer | 0 | 5 | 1 | 6 |
|  | Executives in Charge of operations | 15 | 15 | 6 | 36 |
| Total | | 101 | 76 | 33 | 210 |

1. **For how long have you been in this position?**

| **Period of stay in current position * Category of hospital classification Crosstabulation** | | | | | |
| --- | --- | --- | --- | --- | --- |
| Count | | | | | |
|  | | Category of hospital classification | | | Total |
|  |  | Public Hospital | Private Hospital | Faith-Based Hospital/NGO |  |
| Period of stay in current position | Less than a year | 23 | 9 | 3 | 35 |
|  | 1-5 years | 57 | 37 | 16 | 110 |
|  | 5-10 years | 18 | 14 | 12 | 44 |
|  | 10 and above | 3 | 13 | 2 | 18 |
| Total | | 101 | 73 | 33 | 207 |

| **Period of stay in current position * Category of hospital classification Crosstabulation** | | | | | |
| --- | --- | --- | --- | --- | --- |
| Count | | | | | |
|  | | Category of hospital classification | | | Total |
|  |  | Public Hospital | Private Hospital | Faith-Based Hospital/NGO |  |
| Period of stay in current position | Less than a year | 23 | 9 | 3 | 35 |
|  | 1-5 years | 57 | 37 | 16 | 110 |
|  | 5-10 years | 18 | 14 | 12 | 44 |
|  | 10 and above | 3 | 13 | 2 | 18 |
| Total | | 101 | 73 | 33 | 207 |

1. **Sex** 🗆 Male 🗆 Female of respondent

| **Gender of respondent * Category of hospital classification Crosstabulation** | | | | | |
| --- | --- | --- | --- | --- | --- |
| Count | | | | | |
|  | | Category of hospital classification | | | Total |
|  |  | Public Hospital | Private Hospital | Faith-Based Hospital/NGO |  |
| Gender of respondent | Female | 24 | 20 | 8 | 52 |
|  | Male | 77 | 56 | 25 | 158 |
| Total | | 101 | 76 | 33 | 210 |

1. **Age** 🗆 21-30 🗆 31-40 🗆 41-50 🗆 51-60 🗆 Over 60 years

| **Age of respondent * Category of hospital classification Crosstabulation** | | | | | |
| --- | --- | --- | --- | --- | --- |
| Count | | | | | |
|  | | Category of hospital classification | | | Total |
|  |  | Public Hospital | Private Hospital | Faith-Based Hospital/NGO |  |
| Age of respondent | 21-30 | 18 | 13 | 8 | 39 |
|  | 31-40 | 45 | 33 | 12 | 90 |
|  | 41-50 | 28 | 14 | 8 | 50 |
|  | 51-60 | 10 | 13 | 4 | 27 |
|  | Above 60 | 0 | 3 | 1 | 4 |
| Total | | 101 | 76 | 33 | 210 |

| **What is your highest level of education attained? highest level of education attained * Category of hospital classification Crosstabulation** | | | | | |
| --- | --- | --- | --- | --- | --- |
| Count | | | | | |
|  | | Category of hospital classification | | | Total |
|  |  | Public Hospital | Private Hospital | Faith-Based Hospital/NGO |  |
| Highest level of education attained | Undergraduate | 55 | 29 | 13 | 97 |
|  | Post-graduate/Masters | 35 | 35 | 11 | 81 |
|  | Doctorate/PhD | 1 | 5 | 0 | 6 |
|  | Diploma | 10 | 7 | 5 | 22 |
|  | 5 | 1 | 0 | 0 | 1 |
| Total | | 102 | 76 | 29 | 207 |

| **Highest level of education attained * Category of hospital classification Crosstabulation** | | | | | |
| --- | --- | --- | --- | --- | --- |
| Count | | | | | |
|  | | Category of hospital classification | | | Total |
|  |  | Public Hospital | Private Hospital | Faith-Based Hospital/NGO |  |
| Highest level of education attained | Undergraduate | 55 | 29 | 13 | 97 |
|  | Post-graduate/Masters | 35 | 35 | 11 | 81 |
|  | Doctorate/PhD | 1 | 5 | 0 | 6 |
|  | Diploma | 11 | 7 | 5 | 23 |
| Total | | 102 | 76 | 29 | 207 |

1. **How would you rate your own knowledge of M-Health?**

| **Extent of your knowledge of M-Health * Category of hospital classification Crosstabulation** | | | | | |
| --- | --- | --- | --- | --- | --- |
| Count | | | | | |
|  | | Category of hospital classification | | | Total |
|  |  | Public Hospital | Private Hospital | Faith-Based Hospital/NGO |  |
| Extent of your knowledge of M-Health | High | 9 | 14 | 4 | 27 |
|  | Medium | 63 | 49 | 24 | 136 |
|  | Low | 23 | 9 | 5 | 37 |
|  | None | 5 | 4 | 0 | 9 |
| Total | | 100 | 76 | 33 | 209 |

1. **What is the age of your hospital?**

| **Age of hospital * Category of hospital classification Crosstabulation** | | | | | |
| --- | --- | --- | --- | --- | --- |
| Count | | | | | |
|  | | Category of hospital classification | | | Total |
|  |  | Public Hospital | Private Hospital | Faith-Based Hospital/NGO |  |
| Age of hospital | less than 10 years | 7 | 27 | 1 | 35 |
|  | 10-20 | 13 | 22 | 7 | 42 |
|  | 21-30 | 13 | 16 | 5 | 34 |
|  | 31 - 40 | 19 | 1 | 8 | 28 |
|  | Above 40 | 49 | 10 | 12 | 71 |
| Total | | 101 | 76 | 33 | 210 |

1. **Which level is your hospital classified under in the Government of Kenya (GoK) classification?**

| **Classification level of facility * Category of hospital classification Crosstabulation** | | | | | |
| --- | --- | --- | --- | --- | --- |
| Count | | | | | |
|  | | Category of hospital classification | | | Total |
|  |  | Public Hospital | Private Hospital | Faith-Based Hospital/NGO |  |
| Classification level of facility | Level IV | 86 | 61 | 23 | 170 |
|  | Level V | 14 | 12 | 7 | 33 |
|  | Level VI | 2 | 3 | 3 | 8 |
| Total | | 102 | 76 | 33 | 211 |

1. **Which geographical category is your hospital classified under in the GOK classification?**

| **Geographical category of facility classification * Category of hospital classification Crosstabulation** | | | | | |
| --- | --- | --- | --- | --- | --- |
| Count | | | | | |
|  | | Category of hospital classification | | | Total |
|  |  | Public Hospital | Private Hospital | Faith-Based Hospital/NGO |  |
| Geographical category of facility classification | Urban | 24 | 46 | 7 | 77 |
|  | Semi-urban | 44 | 24 | 12 | 80 |
|  | Rural | 34 | 6 | 14 | 54 |
| Total | | 102 | 76 | 33 | 211 |

1. **What is the current number of staff in the hospital?**

| **Number of staff * Category of hospital classification Crosstabulation** | | | | | |
| --- | --- | --- | --- | --- | --- |
| Count | | | | | |
|  | | Category of hospital classification | | | Total |
|  |  | Public Hospital | Private Hospital | Faith-Based Hospital/NGO |  |
| Number of staff | Less than 100 | 53 | 52 | 20 | 125 |
|  | 100-200 | 28 | 14 | 9 | 51 |
|  | 201-300 | 9 | 3 | 1 | 13 |
|  | 301-500 | 10 | 1 | 1 | 12 |
|  | 501 and above | 2 | 6 | 2 | 10 |
| Total | | 102 | 76 | 33 | 211 |

| **Chi-Square Tests** | | | |
| --- | --- | --- | --- |
|  | Value | df | Asymptotic Significance (2-sided) |
| Pearson Chi-Square | 15.315^a^ | 8 | .053 |
| Likelihood Ratio | 16.342 | 8 | .038 |
| N of Valid Cases | 211 |  |  |
| a. 7 cells (46.7%) have expected count less than 5. The minimum expected count is 1.56. | | | |

\Staff by Level

| **Number of staff * Classification level of facility Crosstabulation** | | | | | |
| --- | --- | --- | --- | --- | --- |
| Count | | | | | |
|  | | Classification level of facility | | | Total |
|  |  | Level IV | Level V | Level VI |  |
| Number of staff | Less than 100 | 119 | 6 | 0 | 125 |
|  | 100-200 | 38 | 13 | 0 | 51 |
|  | 201-300 | 9 | 3 | 1 | 13 |
|  | 301-500 | 3 | 7 | 2 | 12 |
|  | 501 and above | 1 | 4 | 5 | 10 |
| Total | | 170 | 33 | 8 | 211 |

| **Chi-Square Tests** | | | |
| --- | --- | --- | --- |
|  | Value | df | Asymptotic Significance (2-sided) |
| Pearson Chi-Square | 114.094^a^ | 8 | .000 |
| Likelihood Ratio | 79.780 | 8 | .000 |
| N of Valid Cases | 211 |  |  |
| a. 8 cells (53.3%) have expected count less than 5. The minimum expected count is .38. | | | |

Staff by hospital location

| **Number of staff * Geographical category of facility classification Crosstabulation** | | | | | |
| --- | --- | --- | --- | --- | --- |
| Count | | | | | |
|  | | Geographical category of facility classification | | | Total |
|  |  | Urban | Semi-urban | Rural |  |
| Number of staff | Less than 100 | 34 | 48 | 43 | 125 |
|  | 100-200 | 22 | 22 | 7 | 51 |
|  | 201-300 | 7 | 5 | 1 | 13 |
|  | 301-500 | 7 | 4 | 1 | 12 |
|  | 501 and above | 7 | 1 | 2 | 10 |
| Total | | 77 | 80 | 54 | 211 |

| **Chi-Square Tests** | | | |
| --- | --- | --- | --- |
|  | Value | df | Asymptotic Significance (2-sided) |
| Pearson Chi-Square | 21.532^a^ | 8 | .006 |
| Likelihood Ratio | 22.932 | 8 | .003 |
| N of Valid Cases | 211 |  |  |
| a. 9 cells (60.0%) have expected count less than 5. The minimum expected count is 2.56. | | | |

1. **What is the current annual number of patients (outpatients and in-patients) seen in the hospital?**

| **Current annual number of patients (outpatients and in-patients) in thousands * Category of hospital classification Crosstabulation** | | | | | |
| --- | --- | --- | --- | --- | --- |
| Count | | | | | |
|  | | Category of hospital classification | | | Total |
|  |  | Public Hospital | Private Hospital | Faith-Based Hospital/NGO |  |
| Current annual number of patients (outpatients and in-patients) | Less than 200 | 14 | 23 | 12 | 49 |
|  | 200-500 | 24 | 23 | 9 | 56 |
|  | 501-1000 | 13 | 9 | 2 | 24 |
|  | 1001-1500 | 3 | 9 | 1 | 13 |
|  | 1501-2000 | 8 | 3 | 4 | 15 |
|  | 2001 and above | 36 | 9 | 5 | 50 |
| Total | | 98 | 76 | 33 | 207 |

| **Number of staff * Geographical category of facility classification Crosstabulation** | | | | | |
| --- | --- | --- | --- | --- | --- |
| Count | | | | | |
|  | | Geographical category of facility classification | | | Total |
|  |  | Urban | Semi-urban | Rural |  |
| Number of staff | Less than 100 | 34 | 48 | 43 | 125 |
|  | 100-200 | 22 | 22 | 7 | 51 |
|  | 201-300 | 7 | 5 | 1 | 13 |
|  | 301-500 | 7 | 4 | 1 | 12 |
|  | 501 and above | 7 | 1 | 2 | 10 |
| Total | | 77 | 80 | 54 | 211 |

| **Chi-Square Tests** | | | |
| --- | --- | --- | --- |
|  | Value | df | Asymptotic Significance (2-sided) |
| Pearson Chi-Square | 21.532^a^ | 8 | .006 |
| Likelihood Ratio | 22.932 | 8 | .003 |
| N of Valid Cases | 211 |  |  |
| a. 9 cells (60.0%) have expected count less than 5. The minimum expected count is 2.56. | | | |

**Current annual number of patients (outpatients and in-patients) in thousands * Classification level of facility**

| **Crosstab** | | | | | |
| --- | --- | --- | --- | --- | --- |
| Count | | | | | |
|  | | Classification level of facility | | | Total |
|  |  | Level IV | Level V | Level VI |  |
| Current annual number of patients (outpatients and in-patients) | Less than 200 | 46 | 3 | 0 | 49 |
|  | 200-500 | 51 | 5 | 0 | 56 |
|  | 501-1000 | 16 | 8 | 0 | 24 |
|  | 1001-1500 | 11 | 2 | 0 | 13 |
|  | 1501-2000 | 13 | 1 | 1 | 15 |
|  | 2001 and above | 29 | 14 | 7 | 50 |
| Total | | 166 | 33 | 8 | 207 |

| **Chi-Square Tests** | | | |
| --- | --- | --- | --- |
|  | Value | df | Asymptotic Significance (2-sided) |
| Pearson Chi-Square | 39.399^a^ | 10 | .000 |
| Likelihood Ratio | 39.224 | 10 | .000 |
| N of Valid Cases | 207 |  |  |
| a. 9 cells (50.0%) have expected count less than 5. The minimum expected count is .50. | | | |

**Current annual number of patients (outpatients and in-patients) in thousands * Category of hospital classification**

| **Crosstab** | | | | | |
| --- | --- | --- | --- | --- | --- |
| Count | | | | | |
|  | | Category of hospital classification | | | Total |
|  |  | Public Hospital | Private Hospital | Faith-Based Hospital/NGO |  |
| Current annual number of patients (outpatients and in-patients) | Less than 200 | 14 | 23 | 12 | 49 |
|  | 200-500 | 24 | 23 | 9 | 56 |
|  | 501-1000 | 13 | 9 | 2 | 24 |
|  | 1001-1500 | 3 | 9 | 1 | 13 |
|  | 1501-2000 | 8 | 3 | 4 | 15 |
|  | 2001 and above | 36 | 9 | 5 | 50 |
| Total | | 98 | 76 | 33 | 207 |

| **Chi-Square Tests** | | | |
| --- | --- | --- | --- |
|  | Value | df | Asymptotic Significance (2-sided) |
| Pearson Chi-Square | 29.478^a^ | 10 | .001 |
| Likelihood Ratio | 29.992 | 10 | .001 |
| N of Valid Cases | 207 |  |  |
| a. 4 cells (22.2%) have expected count less than 5. The minimum expected count is 2.07. | | | |

**Current annual number of patients (outpatients and in-patients) in thousands * Geographical category of facility classification**

| **Crosstab** | | | | | |
| --- | --- | --- | --- | --- | --- |
| Count | | | | | |
|  | | Geographical category of facility classification | | | Total |
|  |  | Urban | Semi-urban | Rural |  |
| Current annual number of patients (outpatients and in-patients) | Less than 200 | 13 | 20 | 16 | 49 |
|  | 200-500 | 20 | 20 | 16 | 56 |
|  | 501-1000 | 10 | 10 | 4 | 24 |
|  | 1001-1500 | 7 | 5 | 1 | 13 |
|  | 1501-2000 | 4 | 8 | 3 | 15 |
|  | 2001 and above | 22 | 17 | 11 | 50 |
| Total | | 76 | 80 | 51 | 207 |

| **Chi-Square Tests** | | | |
| --- | --- | --- | --- |
|  | Value | df | Asymptotic Significance (2-sided) |
| Pearson Chi-Square | 9.127^a^ | 10 | .520 |
| Likelihood Ratio | 9.582 | 10 | .478 |
| N of Valid Cases | 207 |  |  |
| a. 3 cells (16.7%) have expected count less than 5. The minimum expected count is 3.20. | | | |

1. **Please indicate the average medical consultation fee that your hospital charges for outpatient services**

| **Average medical consultation outpatient fee charged * Category of hospital classification Crosstabulation** | | | | | |
| --- | --- | --- | --- | --- | --- |
| Count | | | | | |
|  | | Category of hospital classification | | | Total |
|  |  | Public Hospital | Private Hospital | Faith-Based Hospital/NGO |  |
| Average medical consultation outpatient fee chargedf | Less than 200 | 79 | 3 | 12 | 94 |
|  | 200-500 | 12 | 28 | 14 | 54 |
|  | 501-1,000 | 2 | 19 | 3 | 24 |
|  | 1,001-2,1500 | 1 | 10 | 4 | 15 |
|  | 1501- 2000 | 0 | 6 | 0 | 6 |
|  | 2,001 & Above | 0 | 9 | 0 | 9 |
|  | None | 6 | 1 | 0 | 7 |
| Total | | 100 | 76 | 33 | 209 |

**Average medical consultation outpatient fee charged * Classification level of facility**

| **Crosstab** | | | | | |
| --- | --- | --- | --- | --- | --- |
| Count | | | | | |
|  | | Classification level of facility | | | Total |
|  |  | Level IV | Level V | Level VI |  |
| Average medical consultation outpatient fee chargedf | Less than 200 | 80 | 13 | 1 | 94 |
|  | 200-500 | 44 | 6 | 4 | 54 |
|  | 501-1,000 | 21 | 3 | 0 | 24 |
|  | 1,001-2,1500 | 7 | 8 | 0 | 15 |
|  | 1501- 2000 | 3 | 1 | 2 | 6 |
|  | 2,001 & Above | 6 | 2 | 1 | 9 |
|  | None | 7 | 0 | 0 | 7 |
| Total | | 168 | 33 | 8 | 209 |

| **Chi-Square Tests** | | | |
| --- | --- | --- | --- |
|  | Value | df | Asymptotic Significance (2-sided) |
| Pearson Chi-Square | 39.991^a^ | 12 | .000 |
| Likelihood Ratio | 29.641 | 12 | .003 |
| N of Valid Cases | 209 |  |  |
| a. 13 cells (61.9%) have expected count less than 5. The minimum expected count is .23. | | | |

**Average medical consultation outpatient fee chargedf * Category of hospital classification**

| **Crosstab** | | | | | |
| --- | --- | --- | --- | --- | --- |
| Count | | | | | |
|  | | Category of hospital classification | | | Total |
|  |  | Public Hospital | Private Hospital | Faith-Based Hospital/NGO |  |
| Average medical consultation outpatient fee chargedf | Less than 200 | 79 | 3 | 12 | 94 |
|  | 200-500 | 12 | 28 | 14 | 54 |
|  | 501-1,000 | 2 | 19 | 3 | 24 |
|  | 1,001-2,1500 | 1 | 10 | 4 | 15 |
|  | 1501- 2000 | 0 | 6 | 0 | 6 |
|  | 2,001 & Above | 0 | 9 | 0 | 9 |
|  | None | 6 | 1 | 0 | 7 |
| Total | | 100 | 76 | 33 | 209 |

| **Chi-Square Tests** | | | |
| --- | --- | --- | --- |
|  | Value | df | Asymptotic Significance (2-sided) |
| Pearson Chi-Square | 129.923^a^ | 12 | .000 |
| Likelihood Ratio | 153.673 | 12 | .000 |
| N of Valid Cases | 209 |  |  |
| a. 11 cells (52.4%) have expected count less than 5. The minimum expected count is .95. | | | |

**Average medical consultation outpatient fee (in Ksh) charged * Geographical category of facility classification**

| **Crosstab** | | | | | |
| --- | --- | --- | --- | --- | --- |
| Count | | | | | |
|  | | Geographical category of facility classification | | | Total |
|  |  | Urban | Semi-urban | Rural |  |
| Average medical consultation outpatient fee chargedf | Less than 200 | 22 | 34 | 38 | 94 |
|  | 200-500 | 19 | 24 | 11 | 54 |
|  | 501-1,000 | 12 | 11 | 1 | 24 |
|  | 1,001-2,1500 | 10 | 3 | 2 | 15 |
|  | 1501- 2000 | 5 | 1 | 0 | 6 |
|  | 2,001 & Above | 9 | 0 | 0 | 9 |
|  | None | 0 | 5 | 2 | 7 |
| Total | | 77 | 78 | 54 | 209 |

| **Chi-Square Tests** | | | |
| --- | --- | --- | --- |
|  | Value | Df | Asymptotic Significance (2-sided) |
| Pearson Chi-Square | 51.439^a^ | 12 | .000 |
| Likelihood Ratio | 58.424 | 12 | .000 |
| N of Valid Cases | 209 |  |  |
| a. 10 cells (47.6%) have expected count less than 5. The minimum expected count is 1.55. | | | |

1. **Please indicate the current turnover of the hospital per annum**

| **Current turnover (in Ksh. millions) of the hospital per annum* Category of hospital classification Crosstabulation** | | | | | |
| --- | --- | --- | --- | --- | --- |
| Count | | | | | |
|  | | Category of hospital classification | | | Total |
|  |  | Public Hospital | Private Hospital | Faith-Based Hospital/NGO |  |
| Current turnover of the hospital per annum | Less than 100 | 82 | 48 | 24 | 154 |
|  | 100-200 | 9 | 11 | 3 | 23 |
|  | 201-400 | 3 | 5 | 1 | 9 |
|  | 401-600 | 1 | 5 | 1 | 7 |
|  | 601-800 | 0 | 2 | 0 | 2 |
|  | 801 and above | 3 | 3 | 4 | 10 |
| Total | | 98 | 74 | 33 | 205 |

**Current turnover (in Ksh. millions) of the hospital per annum * Classification level of facility**

| **Crosstab** | | | | | |
| --- | --- | --- | --- | --- | --- |
| Count | | | | | |
|  | | Classification level of facility | | | Total |
|  |  | Level IV | Level V | Level VI |  |
| Current turnover of the hospital per annum | Less than 100 | 138 | 15 | 1 | 154 |
|  | 100-200 | 15 | 7 | 1 | 23 |
|  | 201-400 | 1 | 8 | 0 | 9 |
|  | 401-600 | 7 | 0 | 0 | 7 |
|  | 601-800 | 0 | 2 | 0 | 2 |
|  | 801 and above | 5 | 0 | 5 | 10 |
|  |  |  |  |  |  |
| Total | | 166 | 32 | 7 | 205 |

| **Chi-Square Tests** | | | |
| --- | --- | --- | --- |
|  | Value | df | Asymptotic Significance (2-sided) |
| Pearson Chi-Square | 136.197^a^ | 12 | .000 |
| Likelihood Ratio | 71.594 | 12 | .000 |
| N of Valid Cases | 205 |  |  |
| a. 14 cells (66.7%) have expected count less than 5. The minimum expected count is .03. | | | |

**Current turnover (in Ksh. millions) of the hospital per annum * Category of hospital classification**

| **Crosstab** | | | | | |
| --- | --- | --- | --- | --- | --- |
| Count | | | | | |
|  | | Category of hospital classification | | | Total |
|  |  | Public Hospital | Private Hospital | Faith-Based Hospital/NGO |  |
| Current turnover of the hospital per annum | Less than 100 | 82 | 48 | 24 | 154 |
|  | 100-200 | 9 | 11 | 3 | 23 |
|  | 201-400 | 3 | 5 | 1 | 9 |
|  | 401-600 | 1 | 5 | 1 | 7 |
|  | 601-800 | 0 | 2 | 0 | 2 |
|  | 801 and above | 3 | 3 | 4 | 10 |
|  |  |  |  |  |  |
| Total | | 98 | 74 | 33 | 205 |

| **Chi-Square Tests** | | | |
| --- | --- | --- | --- |
|  | Value | df | Asymptotic Significance (2-sided) |
| Pearson Chi-Square | 19.772^a^ | 12 | .072 |
| Likelihood Ratio | 18.387 | 12 | .104 |
| N of Valid Cases | 205 |  |  |
| a. 16 cells (76.2%) have expected count less than 5. The minimum expected count is .16. | | | |

**Current turnover (in ksh. Millions) of the hospital per annum * Geographical category of facility classification**

| **Crosstab** | | | | | |
| --- | --- | --- | --- | --- | --- |
| Count | | | | | |
|  | | Geographical category of facility classification | | | Total |
|  |  | Urban | Semi-urban | Rural |  |
| Current turnover of the hospital per annum | Less than 100 | 46 | 61 | 47 | 154 |
|  | 100-200 | 12 | 10 | 1 | 23 |
|  | 201-400 | 7 | 1 | 1 | 9 |
|  | 401-600 | 4 | 2 | 1 | 7 |
|  | 601-800 | 2 | 0 | 0 | 2 |
|  | 801 and above | 4 | 3 | 3 | 10 |
|  |  |  |  |  |  |
| Total | | 75 | 77 | 53 | 205 |

| **Chi-Square Tests** | | | |
| --- | --- | --- | --- |
|  | Value | df | Asymptotic Significance (2-sided) |
| Pearson Chi-Square | 23.728^a^ | 12 | .022 |
| Likelihood Ratio | 25.982 | 12 | .011 |
| N of Valid Cases | 205 |  |  |
| a. 15 cells (71.4%) have expected count less than 5. The minimum expected count is .26. | | | |

**Chi-square test between fees and turnover**

| **Current annual number of patients (outpatients and in-patients) * Current turnover of the hospital per annum Crosstabulation** | | | | | | | | | |
| --- | --- | --- | --- | --- | --- | --- | --- | --- | --- |
| Count | | | | | | | | | |
|  | | Current turnover of the hospital per annum | | | | | | | Total |
|  |  | Less than 100 | 100-200 | 201-400 | 401-600 | 601-800 | 801 and above | 7 |  |
| Current annual number of patients (outpatients and in-patients) | Less than 200 | 44 | 1 | 1 | 1 | 0 | 2 | 0 | 49 |
|  | 200-500 | 46 | 7 | 1 | 1 | 0 | 0 | 0 | 55 |
|  | 501-1000 | 17 | 3 | 2 | 0 | 0 | 1 | 0 | 23 |
|  | 1001-1500 | 9 | 2 | 1 | 0 | 0 | 0 | 0 | 12 |
|  | 1501-2000 | 9 | 2 | 1 | 1 | 0 | 1 | 0 | 14 |
|  | 2001 and above | 26 | 8 | 3 | 4 | 2 | 5 | 1 | 49 |
| Total | | 151 | 23 | 9 | 7 | 2 | 9 | 1 | 202 |

| **Chi-Square Tests** | | | |
| --- | --- | --- | --- |
|  | Value | df | Asymptotic Significance (2-sided) |
| Pearson Chi-Square | 35.897^a^ | 30 | .211 |
| Likelihood Ratio | 39.445 | 30 | .116 |
| N of Valid Cases | 202 |  |  |
| a. 33 cells (78.6%) have expected count less than 5. The minimum expected count is .06. | | | |

1. **Please indicate your current target market and focus (tick the top 3 target markets that the hospital is currently actively pursuing)**

| **County only Market Focus * Category of hospital classification Crosstabulation** | | | | | |
| --- | --- | --- | --- | --- | --- |
| Count | | | | | |
|  | | Category of hospital classification | | | Total |
|  |  | Public Hospital | Private Hospital | Faith-Based Hospital/NGO |  |
| County only Market Focus | No | 11 | 22 | 3 | 36 |
|  | Yes | 91 | 54 | 30 | 175 |
| Total | | 102 | 76 | 33 | 211 |

**Classification level of facility * County Only market focus**

| **Crosstab** | | | | |
| --- | --- | --- | --- | --- |
| Count | | | | |
|  | | County | | Total |
|  |  | No | Yes |  |
| Classification level of facility | Level IV | 26 | 144 | 170 |
|  | Level V | 6 | 27 | 33 |
|  | Level VI | 4 | 4 | 8 |
| Total | | 36 | 175 | 211 |

| **Chi-Square Tests** | | | |
| --- | --- | --- | --- |
|  | Value | df | Asymptotic Significance (2-sided) |
| Pearson Chi-Square | 6.538^a^ | 2 | .038 |
| Likelihood Ratio | 4.968 | 2 | .083 |
| N of Valid Cases | 211 |  |  |
| a. 1 cells (16.7%) have expected count less than 5. The minimum expected count is 1.36. | | | |

**Ownership/Category of hospital classification * County Only Market Focus**

| **Crosstab** | | | | |
| --- | --- | --- | --- | --- |
| Count | | | | |
|  | | County | | Total |
|  |  | No | Yes |  |
| Category of hospital classification | Public Hospital | 11 | 91 | 102 |
|  | Private Hospital | 22 | 54 | 76 |
|  | Faith-Based Hospital/NGO | 3 | 30 | 33 |
| Total | | 36 | 175 | 211 |

| **Chi-Square Tests** | | | |
| --- | --- | --- | --- |
|  | Value | df | Asymptotic Significance (2-sided) |
| Pearson Chi-Square | 11.909^a^ | 2 | .003 |
| Likelihood Ratio | 11.470 | 2 | .003 |
| N of Valid Cases | 211 |  |  |
| a. 0 cells (0.0%) have expected count less than 5. The minimum expected count is 5.63. | | | |

**Geographical category of facility classification * County Only Market Focus**

| **Crosstab** | | | | |
| --- | --- | --- | --- | --- |
| Count | | | | |
|  | | County | | Total |
|  |  | No | Yes |  |
| Geographical category of facility classification | Urban | 23 | 54 | 77 |
|  | Semi-urban | 9 | 71 | 80 |
|  | Rural | 4 | 50 | 54 |
| Total | | 36 | 175 | 211 |

| **Chi-Square Tests** | | | |
| --- | --- | --- | --- |
|  | Value | df | Asymptotic Significance (2-sided) |
| Pearson Chi-Square | 14.393^a^ | 2 | .001 |
| Likelihood Ratio | 14.101 | 2 | .001 |
| N of Valid Cases | 211 |  |  |
| a. 0 cells (0.0%) have expected count less than 5. The minimum expected count is 9.21. | | | |

| **Country - Wide market * Category of hospital classification Crosstabulation** | | | | | |
| --- | --- | --- | --- | --- | --- |
| Count | | | | | |
|  | | Category of hospital classification | | | Total |
|  |  | Public Hospital | Private Hospital | Faith-Based Hospital/NGO |  |
| Country - Wide market | No | 39 | 18 | 4 | 61 |
|  | Yes | 63 | 58 | 29 | 150 |
| Total | | 102 | 76 | 33 | 211 |

**Classification level of facility * Country wide market focus**

| **Crosstab** | | | | | |
| --- | --- | --- | --- | --- | --- |
| Count | | | | | |
|  | | Country | | | Total |
|  |  | No | Yes | 2 |  |
| Classification level of facility | Level IV | 49 | 120 | 1 | 170 |
|  | Level V | 9 | 24 | 0 | 33 |
|  | Level VI | 3 | 5 | 0 | 8 |
| Total | | 61 | 149 | 1 | 211 |

| **Chi-Square Tests** | | | |
| --- | --- | --- | --- |
|  | Value | df | Asymptotic Significance (2-sided) |
| Pearson Chi-Square | .572^a^ | 4 | .966 |
| Likelihood Ratio | .749 | 4 | .945 |
| N of Valid Cases | 211 |  |  |
| a. 4 cells (44.4%) have expected count less than 5. The minimum expected count is .04. | | | |

**Category of hospital classification * Country wide Market Focus**

| **Crosstab** | | | | | |
| --- | --- | --- | --- | --- | --- |
| Count | | | | | |
|  | | Country | | | Total |
|  |  | No | Yes | 2 |  |
| Category of hospital classification | Public Hospital | 39 | 63 | 0 | 102 |
|  | Private Hospital | 18 | 58 | 0 | 76 |
|  | Faith-Based Hospital/NGO | 4 | 28 | 1 | 33 |
| Total | | 61 | 149 | 1 | 211 |

| **Chi-Square Tests** | | | |
| --- | --- | --- | --- |
|  | Value | df | Asymptotic Significance (2-sided) |
| Pearson Chi-Square | 14.825^a^ | 4 | .005 |
| Likelihood Ratio | 13.797 | 4 | .008 |
| N of Valid Cases | 211 |  |  |
| a. 3 cells (33.3%) have expected count less than 5. The minimum expected count is .16. | | | |

**Geographical category of facility classification * Country wide Market Focus**

| **Crosstab** | | | | | |
| --- | --- | --- | --- | --- | --- |
| Count | | | | | |
|  | | Country | | | Total |
|  |  | No | Yes | 2 |  |
| Geographical category of facility classification | Urban | 22 | 55 | 0 | 77 |
|  | Semi-urban | 23 | 57 | 0 | 80 |
|  | Rural | 16 | 37 | 1 | 54 |
| Total | | 61 | 149 | 1 | 211 |

| **Chi-Square Tests** | | | |
| --- | --- | --- | --- |
|  | Value | df | Asymptotic Significance (2-sided) |
| Pearson Chi-Square | 2.966^a^ | 4 | .563 |
| Likelihood Ratio | 2.785 | 4 | .594 |
| N of Valid Cases | 211 |  |  |
| a. 3 cells (33.3%) have expected count less than 5. The minimum expected count is .26. | | | |

| **Eastern Africa Market focus * Category of hospital classification Crosstabulation** | | | | | |
| --- | --- | --- | --- | --- | --- |
| Count | | | | | |
|  | | Category of hospital classification | | | Total |
|  |  | Public Hospital | Private Hospital | Faith-Based Hospital/NGO |  |
| Eastern Africa Market focus | No | 72 | 39 | 19 | 130 |
|  | Yes | 30 | 37 | 14 | 81 |
| Total | | 102 | 76 | 33 | 211 |

**Classification level of facility * East African market focus**

| **Crosstab** | | | | | |
| --- | --- | --- | --- | --- | --- |
| Count | | | | | |
|  | | Eastern Africa | | | Total |
|  |  | No | Yes | 2 |  |
| Classification level of facility | Level IV | 109 | 59 | 2 | 170 |
|  | Level V | 17 | 16 | 0 | 33 |
|  | Level VI | 4 | 4 | 0 | 8 |
| Total | | 130 | 79 | 2 | 211 |

| **Chi-Square Tests** | | | |
| --- | --- | --- | --- |
|  | Value | df | Asymptotic Significance (2-sided) |
| Pearson Chi-Square | 3.128^a^ | 4 | .537 |
| Likelihood Ratio | 3.436 | 4 | .488 |
| N of Valid Cases | 211 |  |  |
| a. 5 cells (55.6%) have expected count less than 5. The minimum expected count is .08. | | | |

**Ownership Category of hospital classification * Eastern Africa**

| **Crosstab** | | | | | |
| --- | --- | --- | --- | --- | --- |
| Count | | | | | |
|  | | Eastern Africa | | | Total |
|  |  | No | Yes | 2 |  |
| Category of hospital classification | Public Hospital | 72 | 30 | 0 | 102 |
|  | Private Hospital | 39 | 36 | 1 | 76 |
|  | Faith-Based Hospital/NGO | 19 | 13 | 1 | 33 |
| Total | | 130 | 79 | 2 | 211 |

| **Chi-Square Tests** | | | |
| --- | --- | --- | --- |
|  | Value | df | Asymptotic Significance (2-sided) |
| Pearson Chi-Square | 9.104^a^ | 4 | .059 |
| Likelihood Ratio | 9.505 | 4 | .050 |
| N of Valid Cases | 211 |  |  |
| a. 3 cells (33.3%) have expected count less than 5. The minimum expected count is .31. | | | |

**Geographical category of facility classification * Eastern Africa**

| **Crosstab** | | | | | |
| --- | --- | --- | --- | --- | --- |
| Count | | | | | |
|  | | Eastern Africa | | | Total |
|  |  | No | Yes | 2 |  |
| Geographical category of facility classification | Urban | 42 | 35 | 0 | 77 |
|  | Semi-urban | 53 | 27 | 0 | 80 |
|  | Rural | 35 | 17 | 2 | 54 |
| Total | | 130 | 79 | 2 | 211 |

| **Chi-Square Tests** | | | |
| --- | --- | --- | --- |
|  | Value | df | Asymptotic Significance (2-sided) |
| Pearson Chi-Square | 8.932^a^ | 4 | .063 |
| Likelihood Ratio | 8.540 | 4 | .074 |
| N of Valid Cases | 211 |  |  |
| a. 3 cells (33.3%) have expected count less than 5. The minimum expected count is .51. | | | |

| **Africa Wide Market focus * Category of hospital classification Crosstabulation** | | | | | |
| --- | --- | --- | --- | --- | --- |
| Count | | | | | |
|  | | Category of hospital classification | | | Total |
|  |  | Public Hospital | Private Hospital | Faith-Based Hospital/NGO |  |
| Africa Wide Market focus | No | 102 | 74 | 32 | 208 |
|  | Yes | 0 | 2 | 1 | 3 |
| Total | | 102 | 76 | 33 | 211 |

**Classification level of facility * Africa market focus**

| **Crosstab** | | | | | |
| --- | --- | --- | --- | --- | --- |
| Count | | | | | |
|  | | Africa Wide | | | Total |
|  |  | No | Yes | 2 |  |
| Classification level of facility | Level IV | 168 | 0 | 2 | 170 |
|  | Level V | 33 | 0 | 0 | 33 |
|  | Level VI | 7 | 1 | 0 | 8 |
| Total | | 208 | 1 | 2 | 211 |

| **Chi-Square Tests** | | | |
| --- | --- | --- | --- |
|  | Value | df | Asymptotic Significance (2-sided) |
| Pearson Chi-Square | 25.965^a^ | 4 | .000 |
| Likelihood Ratio | 7.520 | 4 | .111 |
| N of Valid Cases | 211 |  |  |
| a. 6 cells (66.7%) have expected count less than 5. The minimum expected count is .04. | | | |

**Ownership Category of hospital classification * Africa Wide**

| **Crosstab** | | | | | |
| --- | --- | --- | --- | --- | --- |
| Count | | | | | |
|  | | Africa Wide | | | Total |
|  |  | No | Yes | 2 |  |
| Category of hospital classification | Public Hospital | 102 | 0 | 0 | 102 |
|  | Private Hospital | 74 | 1 | 1 | 76 |
|  | Faith-Based Hospital/NGO | 32 | 0 | 1 | 33 |
| Total | | 208 | 1 | 2 | 211 |

| **Chi-Square Tests** | | | |
| --- | --- | --- | --- |
|  | Value | df | Asymptotic Significance (2-sided) |
| Pearson Chi-Square | 4.402^a^ | 4 | .354 |
| Likelihood Ratio | 5.063 | 4 | .281 |
| N of Valid Cases | 211 |  |  |
| a. 6 cells (66.7%) have expected count less than 5. The minimum expected count is .16. | | | |

**Geographical category of facility classification * Africa Wide**

| **Crosstab** | | | | | |
| --- | --- | --- | --- | --- | --- |
| Count | | | | | |
|  | | Africa Wide | | | Total |
|  |  | No | Yes | 2 |  |
| Geographical category of facility classification | Urban | 76 | 1 | 0 | 77 |
|  | Semi-urban | 80 | 0 | 0 | 80 |
|  | Rural | 52 | 0 | 2 | 54 |
| Total | | 208 | 1 | 2 | 211 |

| **Chi-Square Tests** | | | |
| --- | --- | --- | --- |
|  | Value | df | Asymptotic Significance (2-sided) |
| Pearson Chi-Square | 7.600^a^ | 4 | .107 |
| Likelihood Ratio | 7.513 | 4 | .111 |
| N of Valid Cases | 211 |  |  |
| a. 6 cells (66.7%) have expected count less than 5. The minimum expected count is .26. | | | |

| **Global Market focus * Category of hospital classification Crosstabulation** | | | | | |
| --- | --- | --- | --- | --- | --- |
| Count | | | | | |
|  | | Category of hospital classification | | | Total |
|  |  | Public Hospital | Private Hospital | Faith-Based Hospital/NGO |  |
| Global Market focus | No | 101 | 72 | 33 | 206 |
|  | Yes | 1 | 4 | 0 | 5 |
| Total | | 102 | 76 | 33 | 211 |

**Classification level of facility * Global market focus**

| **Crosstab** | | | | | |
| --- | --- | --- | --- | --- | --- |
| Count | | | | | |
|  | | Global | | | Total |
|  |  | No | Yes | 2 |  |
| Classification level of facility | Level IV | 166 | 2 | 2 | 170 |
|  | Level V | 32 | 1 | 0 | 33 |
|  | Level VI | 6 | 2 | 0 | 8 |
| Total | | 204 | 5 | 2 | 211 |

| **Chi-Square Tests** | | | |
| --- | --- | --- | --- |
|  | Value | df | Asymptotic Significance (2-sided) |
| Pearson Chi-Square | 19.260^a^ | 4 | .001 |
| Likelihood Ratio | 8.418 | 4 | .077 |
| N of Valid Cases | 211 |  |  |
| a. 6 cells (66.7%) have expected count less than 5. The minimum expected count is .08. | | | |

**Ownership Category of hospital classification * Global**

| **Crosstab** | | | | | |
| --- | --- | --- | --- | --- | --- |
| Count | | | | | |
|  | | Global | | | Total |
|  |  | No | Yes | 2 |  |
| Category of hospital classification | Public Hospital | 101 | 1 | 0 | 102 |
|  | Private Hospital | 71 | 4 | 1 | 76 |
|  | Faith-Based Hospital/NGO | 32 | 0 | 1 | 33 |
| Total | | 204 | 5 | 2 | 211 |

| **Chi-Square Tests** | | | |
| --- | --- | --- | --- |
|  | Value | df | Asymptotic Significance (2-sided) |
| Pearson Chi-Square | 7.025^a^ | 4 | .135 |
| Likelihood Ratio | 7.741 | 4 | .102 |
| N of Valid Cases | 211 |  |  |
| a. 6 cells (66.7%) have expected count less than 5. The minimum expected count is .31. | | | |

**Geographical category of facility classification * Global**

| **Crosstab** | | | | | |
| --- | --- | --- | --- | --- | --- |
| Count | | | | | |
|  | | Global | | | Total |
|  |  | No | Yes | 2 |  |
| Geographical category of facility classification | Urban | 75 | 2 | 0 | 77 |
|  | Semi-urban | 78 | 2 | 0 | 80 |
|  | Rural | 51 | 1 | 2 | 54 |
| Total | | 204 | 5 | 2 | 211 |

| **Chi-Square Tests** | | | |
| --- | --- | --- | --- |
|  | Value | df | Asymptotic Significance (2-sided) |
| Pearson Chi-Square | 5.936^a^ | 4 | .204 |
| Likelihood Ratio | 5.577 | 4 | .233 |
| N of Valid Cases | 211 |  |  |
| a. 6 cells (66.7%) have expected count less than 5. The minimum expected count is .51. | | | |

1. **Please, indicate the extent to which your hospital pursues market growth through technology leadership (as indicated in its vision and mission)**

| **Extent to which hospital pursues market growth through technology * Category of hospital classification Crosstabulation** | | | | | |
| --- | --- | --- | --- | --- | --- |
| Count | | | | | |
|  | | Category of hospital classification | | | Total |
|  |  | Public Hospital | Private Hospital | Faith-Based Hospital/NGO |  |
| Extent to which hospital pursues market growth through technology | High | 10 | 29 | 8 | 47 |
|  | Medium | 47 | 39 | 18 | 104 |
|  | Low | 34 | 4 | 7 | 45 |
|  | None | 11 | 4 | 0 | 15 |
| Total | | 102 | 76 | 33 | 211 |

**Category of hospital classification * Extent to which hospital pursues market growth through technology**

| **Crosstab** | | | | | | |
| --- | --- | --- | --- | --- | --- | --- |
| Count | | | | | | |
|  | | Extent to which hospital pursues market growth through technology | | | | Total |
|  |  | High | Medium | Low | None |  |
| Category of hospital classification | Public Hospital | 10 | 47 | 34 | 11 | 102 |
|  | Private Hospital | 29 | 39 | 4 | 4 | 76 |
|  | Faith-Based Hospital/NGO | 8 | 18 | 7 | 0 | 33 |
| Total | | 47 | 104 | 45 | 15 | 211 |

| **Chi-Square Tests** | | | |
| --- | --- | --- | --- |
|  | Value | df | Asymptotic Significance (2-sided) |
| Pearson Chi-Square | 36.986^a^ | 6 | .000 |
| Likelihood Ratio | 42.481 | 6 | .000 |
| N of Valid Cases | 211 |  |  |
| a. 1 cells (8.3%) have expected count less than 5. The minimum expected count is 2.35. | | | |

1. **What is the level of Information Communication and Technology (ICT) infrastructure in the hospital that can/could accommodate implementation of M-health innovations?**

| **Level of facility ICT infrastructure * Category of hospital classification Crosstabulation** | | | | | |
| --- | --- | --- | --- | --- | --- |
| Count | | | | | |
|  | | Category of hospital classification | | | Total |
|  |  | Public Hospital | Private Hospital | Faith-Based Hospital/NGO |  |
| Level of facility ICT infrastructure | High | 1 | 23 | 6 | 30 |
|  | Medium | 56 | 43 | 23 | 122 |
|  | Low | 43 | 8 | 3 | 54 |
|  | None | 2 | 2 | 0 | 4 |
| Total | | 102 | 76 | 32 | 210 |

1. **What is the level of ICT human capacity in the hospital as it relates to effective implementation of M-Health?**

| **Level of ICT human resource capicity * Category of hospital classification Crosstabulation** | | | | | |
| --- | --- | --- | --- | --- | --- |
| Count | | | | | |
|  | | Category of hospital classification | | | Total |
|  |  | Public Hospital | Private Hospital | Faith-Based Hospital/NGO |  |
| Level of ICT human resource capicity | High | 2 | 10 | 5 | 17 |
|  | Medium | 41 | 50 | 20 | 111 |
|  | Low | 50 | 13 | 8 | 71 |
|  | None | 9 | 2 | 0 | 11 |
| Total | | 102 | 75 | 33 | 210 |

1. **What kind of Mobile Financing has the hospital adopted? Please, tick all that applies.**

| **Crosstab** | | | | | | |
| --- | --- | --- | --- | --- | --- | --- |
| Count | | | | | | |
|  | | Level of facility ICT infrastructure | | | | Total |
|  |  | High | Medium | Low | None |  |
| Classification level of facility | Level IV | 18 | 100 | 47 | 4 | 169 |
|  | Level V | 9 | 18 | 6 | 0 | 33 |
|  | Level VI | 3 | 4 | 1 | 0 | 8 |
| Total | | 30 | 122 | 54 | 4 | 210 |

| **Chi-Square Tests** | | | |
| --- | --- | --- | --- |
|  | Value | df | Asymptotic Significance (2-sided) |
| Pearson Chi-Square | 11.203^a^ | 6 | .082 |
| Likelihood Ratio | 10.563 | 6 | .103 |
| N of Valid Cases | 210 |  |  |
| a. 7 cells (58.3%) have expected count less than 5. The minimum expected count is .15. | | | |

**Category of hospital classification * Level of facility ICT infrastructure**

| **Crosstab** | | | | | | |
| --- | --- | --- | --- | --- | --- | --- |
| Count | | | | | | |
|  | | Level of facility ICT infrastructure | | | | Total |
|  |  | High | Medium | Low | None |  |
| Category of hospital classification | Public Hospital | 1 | 56 | 43 | 2 | 102 |
|  | Private Hospital | 23 | 43 | 8 | 2 | 76 |
|  | Faith-Based Hospital/NGO | 6 | 23 | 3 | 0 | 32 |
| Total | | 30 | 122 | 54 | 4 | 210 |

| **Chi-Square Tests** | | | |
| --- | --- | --- | --- |
|  | Value | df | Asymptotic Significance (2-sided) |
| Pearson Chi-Square | 49.609^a^ | 6 | .000 |
| Likelihood Ratio | 57.199 | 6 | .000 |
| N of Valid Cases | 210 |  |  |
| a. 4 cells (33.3%) have expected count less than 5. The minimum expected count is .61. | | | |

**Geographical category of facility classification * Level of facility ICT infrastructure**

| **Crosstab** | | | | | | |
| --- | --- | --- | --- | --- | --- | --- |
| Count | | | | | | |
|  | | Level of facility ICT infrastructure | | | | Total |
|  |  | High | Medium | Low | None |  |
| Geographical category of facility classification | Urban | 16 | 42 | 17 | 2 | 77 |
|  | Semi-urban | 10 | 50 | 19 | 0 | 79 |
|  | Rural | 4 | 30 | 18 | 2 | 54 |
| Total | | 30 | 122 | 54 | 4 | 210 |

| **Chi-Square Tests** | | | |
| --- | --- | --- | --- |
|  | Value | df | Asymptotic Significance (2-sided) |
| Pearson Chi-Square | 9.118^a^ | 6 | .167 |
| Likelihood Ratio | 10.422 | 6 | .108 |
| N of Valid Cases | 210 |  |  |
| a. 3 cells (25.0%) have expected count less than 5. The minimum expected count is 1.03. | | | |

| **Mobile Payment (MPESA, AIRTEL Money) system * Category of hospital classification Crosstabulation** | | | | | |
| --- | --- | --- | --- | --- | --- |
| Count | | | | | |
|  | | Category of hospital classification | | | Total |
|  |  | Public Hospital | Private Hospital | Faith-Based Hospital/NGO |  |
| Mobile Payment (MPESA, AITEl Money) system | No | 65 | 4 | 4 | 73 |
|  | Yes | 37 | 72 | 29 | 138 |
| Total | | 102 | 76 | 33 | 211 |

| **Online or phone banking system * Category of hospital classification Crosstabulation** | | | | | |
| --- | --- | --- | --- | --- | --- |
| Count | | | | | |
|  | | Category of hospital classification | | | Total |
|  |  | Public Hospital | Private Hospital | Faith-Based Hospital/NGO |  |
| Online or phone banking system | No | 96 | 43 | 18 | 157 |
|  | Yes | 6 | 33 | 15 | 54 |
| Total | | 102 | 76 | 33 | 211 |

| **Mobile healthcare financing * Category of hospital classification Crosstabulation** | | | | | |
| --- | --- | --- | --- | --- | --- |
| Count | | | | | |
|  | | Category of hospital classification | | | Total |
|  |  | Public Hospital | Private Hospital | Faith-Based Hospital/NGO |  |
| Mobile healthcare financing | No | 100 | 68 | 30 | 198 |
|  | Yes | 2 | 8 | 3 | 13 |
| Total | | 102 | 76 | 33 | 211 |

| **Pre-paid mobile phone savings account * Category of hospital classification Crosstabulation** | | | | | |
| --- | --- | --- | --- | --- | --- |
| Count | | | | | |
|  | | Category of hospital classification | | | Total |
|  |  | Public Hospital | Private Hospital | Faith-Based Hospital/NGO |  |
| Pre-paid mobile phone savings account | No | 97 | 67 | 30 | 194 |
|  | Yes | 5 | 9 | 3 | 17 |
| Total | | 102 | 76 | 33 | 211 |

1. **Which of the following areas of M-Health applications is your hospital currently using?**

| **Health call centers/telephone help line * Category of hospital classification Crosstabulation** | | | | | |
| --- | --- | --- | --- | --- | --- |
| Count | | | | | |
|  | | Category of hospital classification | | | Total |
|  |  | Public Hospital | Private Hospital | Faith-Based Hospital/NGO |  |
| Health call centers/telephone help line | Not adopted | 24 | 10 | 3 | 37 |
|  | Adopted | 76 | 63 | 29 | 168 |
| Total | | 100 | 73 | 32 | 205 |

| **Crosstab** | | | | |
| --- | --- | --- | --- | --- |
| Count | | | | |
|  | | Health call centers/telephone help line | | Total |
|  |  | Not adopted | Adopted |  |
| Classification level of facility | Level IV | 34 | 130 | 164 |
|  | Level V | 2 | 31 | 33 |
|  | Level VI | 1 | 7 | 8 |
| Total | | 37 | 168 | 205 |

| **Chi-Square Tests** | | | |
| --- | --- | --- | --- |
|  | Value | df | Asymptotic Significance (2-sided) |
| Pearson Chi-Square | 4.171^a^ | 2 | .124 |
| Likelihood Ratio | 5.052 | 2 | .080 |
| N of Valid Cases | 205 |  |  |
| a. 1 cells (16.7%) have expected count less than 5. The minimum expected count is 1.44. | | | |

**Category of hospital classification * Health call centers/telephone help line**

| **Crosstab** | | | | |
| --- | --- | --- | --- | --- |
| Count | | | | |
|  | | Health call centers/telephone help line | | Total |
|  |  | Not adopted | Adopted |  |
| Category of hospital classification | Public Hospital | 24 | 76 | 100 |
|  | Private Hospital | 10 | 63 | 73 |
|  | Faith-Based Hospital/NGO | 3 | 29 | 32 |
| Total | | 37 | 168 | 205 |

| **Chi-Square Tests** | | | |
| --- | --- | --- | --- |
|  | Value | df | Asymptotic Significance (2-sided) |
| Pearson Chi-Square | 4.956^a^ | 2 | .084 |
| Likelihood Ratio | 5.126 | 2 | .077 |
| N of Valid Cases | 205 |  |  |
| a. 0 cells (0.0%) have expected count less than 5. The minimum expected count is 5.78. | | | |

**Geographical category of facility classification * Health call centers/telephone help line**

| **Crosstab** | | | | |
| --- | --- | --- | --- | --- |
| Count | | | | |
|  | | Health call centers/telephone help line | | Total |
|  |  | Not adopted | Adopted |  |
| Geographical category of facility classification | Urban | 11 | 64 | 75 |
|  | Semi-urban | 15 | 62 | 77 |
|  | Rural | 11 | 42 | 53 |
| Total | | 37 | 168 | 205 |

| **Chi-Square Tests** | | | |
| --- | --- | --- | --- |
|  | Value | df | Asymptotic Significance (2-sided) |
| Pearson Chi-Square | .949^a^ | 2 | .622 |
| Likelihood Ratio | .969 | 2 | .616 |
| N of Valid Cases | 205 |  |  |
| a. 0 cells (0.0%) have expected count less than 5. The minimum expected count is 9.57. | | | |

| **Emergency toll-free telephone services * Category of hospital classification Crosstabulation** | | | | | |
| --- | --- | --- | --- | --- | --- |
| Count | | | | | |
|  | | Category of hospital classification | | | Total |
|  |  | Public Hospital | Private Hospital | Faith-Based Hospital/NGO |  |
| Emergency toll-free telephone services | Not adopted | 74 | 45 | 19 | 138 |
|  | Adopted | 23 | 20 | 12 | 55 |
| Total | | 97 | 65 | 31 | 193 |

| **Crosstab** | | | | |
| --- | --- | --- | --- | --- |
| Count | | | | |
|  | | Emergency toll-free telephone services | | Total |
|  |  | Not adopted | Adopted |  |
| Classification level of facility | Level IV | 115 | 42 | 157 |
|  | Level V | 17 | 12 | 29 |
|  | Level VI | 6 | 1 | 7 |
| Total | | 138 | 55 | 193 |

| **Chi-Square Tests** | | | |
| --- | --- | --- | --- |
|  | Value | df | Asymptotic Significance (2-sided) |
| Pearson Chi-Square | 3.290^a^ | 2 | .193 |
| Likelihood Ratio | 3.229 | 2 | .199 |
| N of Valid Cases | 193 |  |  |
| a. 1 cells (16.7%) have expected count less than 5. The minimum expected count is 1.99. | | | |

**Category of hospital classification * Emergency toll-free telephone services**

| **Crosstab** | | | | |
| --- | --- | --- | --- | --- |
| Count | | | | |
|  | | Emergency toll-free telephone services | | Total |
|  |  | Not adopted | Adopted |  |
| Category of hospital classification | Public Hospital | 74 | 23 | 97 |
|  | Private Hospital | 45 | 20 | 65 |
|  | Faith-Based Hospital/NGO | 19 | 12 | 31 |
| Total | | 138 | 55 | 193 |

| **Chi-Square Tests** | | | |
| --- | --- | --- | --- |
|  | Value | df | Asymptotic Significance (2-sided) |
| Pearson Chi-Square | 2.842^a^ | 2 | .242 |
| Likelihood Ratio | 2.788 | 2 | .248 |
| N of Valid Cases | 193 |  |  |
| a. 0 cells (0.0%) have expected count less than 5. The minimum expected count is 8.83. | | | |

**Geographical category of facility classification * Emergency toll-free telephone services**

| **Crosstab** | | | | |
| --- | --- | --- | --- | --- |
| Count | | | | |
|  | | Emergency toll-free telephone services | | Total |
|  |  | Not adopted | Adopted |  |
| Geographical category of facility classification | Urban | 39 | 31 | 70 |
|  | Semi-urban | 60 | 11 | 71 |
|  | Rural | 39 | 13 | 52 |
| Total | | 138 | 55 | 193 |

| **Chi-Square Tests** | | | |
| --- | --- | --- | --- |
|  | Value | df | Asymptotic Significance (2-sided) |
| Pearson Chi-Square | 14.768^a^ | 2 | .001 |
| Likelihood Ratio | 14.837 | 2 | .001 |
| N of Valid Cases | 193 |  |  |
| a. 0 cells (0.0%) have expected count less than 5. The minimum expected count is 14.82. | | | |

| **Treatment compliance * Category of hospital classification Crosstabulation** | | | | | |
| --- | --- | --- | --- | --- | --- |
| Count | | | | | |
|  | | Category of hospital classification | | | Total |
|  |  | Public Hospital | Private Hospital | Faith-Based Hospital/NGO |  |
| Treatment compliance | Not adopted | 30 | 22 | 6 | 58 |
|  | Adopted | 68 | 49 | 26 | 143 |
| Total | | 98 | 71 | 32 | 201 |

| **Crosstab** | | | | |
| --- | --- | --- | --- | --- |
| Count | | | | |
|  | | Treatment compliance | | Total |
|  |  | Not adopted | Adopted |  |
| Classification level of facility | Level IV | 49 | 115 | 164 |
|  | Level V | 8 | 22 | 30 |
|  | Level VI | 1 | 6 | 7 |
| Total | | 58 | 143 | 201 |

| **Chi-Square Tests** | | | |
| --- | --- | --- | --- |
|  | Value | df | Asymptotic Significance (2-sided) |
| Pearson Chi-Square | .877^a^ | 2 | .645 |
| Likelihood Ratio | .984 | 2 | .612 |
| N of Valid Cases | 201 |  |  |
| a. 2 cells (33.3%) have expected count less than 5. The minimum expected count is 2.02. | | | |

**Category of hospital classification * Treatment compliance**

| **Crosstab** | | | | |
| --- | --- | --- | --- | --- |
| Count | | | | |
|  | | Treatment compliance | | Total |
|  |  | Not adopted | Adopted |  |
| Category of hospital classification | Public Hospital | 30 | 68 | 98 |
|  | Private Hospital | 22 | 49 | 71 |
|  | Faith-Based Hospital/NGO | 6 | 26 | 32 |
| Total | | 58 | 143 | 201 |

| **Chi-Square Tests** | | | |
| --- | --- | --- | --- |
|  | Value | df | Asymptotic Significance (2-sided) |
| Pearson Chi-Square | 1.896^a^ | 2 | .387 |
| Likelihood Ratio | 2.034 | 2 | .362 |
| N of Valid Cases | 201 |  |  |
| a. 0 cells (0.0%) have expected count less than 5. The minimum expected count is 9.23. | | | |

**Geographical category of facility classification * Treatment compliance**

| **Crosstab** | | | | |
| --- | --- | --- | --- | --- |
| Count | | | | |
|  | | Treatment compliance | | Total |
|  |  | Not adopted | Adopted |  |
| Geographical category of facility classification | Urban | 23 | 50 | 73 |
|  | Semi-urban | 26 | 50 | 76 |
|  | Rural | 9 | 43 | 52 |
| Total | | 58 | 143 | 201 |

| **Chi-Square Tests** | | | |
| --- | --- | --- | --- |
|  | Value | df | Asymptotic Significance (2-sided) |
| Pearson Chi-Square | 4.689^a^ | 2 | .096 |
| Likelihood Ratio | 5.007 | 2 | .082 |
| N of Valid Cases | 201 |  |  |
| a. 0 cells (0.0%) have expected count less than 5. The minimum expected count is 15.00. | | | |

| **Appointment reminders * Category of hospital classification Crosstabulation** | | | | | |
| --- | --- | --- | --- | --- | --- |
| Count | | | | | |
|  | | Category of hospital classification | | | Total |
|  |  | Public Hospital | Private Hospital | Faith-Based Hospital/NGO |  |
| Appointment reminders | Not adopted | 35 | 17 | 4 | 56 |
|  | Adopted | 66 | 59 | 28 | 153 |
| Total | | 101 | 76 | 32 | 209 |

| **Crosstab** | | | | |
| --- | --- | --- | --- | --- |
| Count | | | | |
|  | | Appointment reminders | | Total |
|  |  | Not adopted | Adopted |  |
| Classification level of facility | Level IV | 47 | 121 | 168 |
|  | Level V | 8 | 25 | 33 |
|  | Level VI | 1 | 7 | 8 |
| Total | | 56 | 153 | 209 |

| **Chi-Square Tests** | | | |
| --- | --- | --- | --- |
|  | Value | df | Asymptotic Significance (2-sided) |
| Pearson Chi-Square | 1.063^a^ | 2 | .588 |
| Likelihood Ratio | 1.203 | 2 | .548 |
| N of Valid Cases | 209 |  |  |
| a. 1 cells (16.7%) have expected count less than 5. The minimum expected count is 2.14. | | | |

**Category of hospital classification * Appointment reminders**

| **Crosstab** | | | | |
| --- | --- | --- | --- | --- |
| Count | | | | |
|  | | Appointment reminders | | Total |
|  |  | Not adopted | Adopted |  |
| Category of hospital classification | Public Hospital | 35 | 66 | 101 |
|  | Private Hospital | 17 | 59 | 76 |
|  | Faith-Based Hospital/NGO | 4 | 28 | 32 |
| Total | | 56 | 153 | 209 |

| **Chi-Square Tests** | | | |
| --- | --- | --- | --- |
|  | Value | df | Asymptotic Significance (2-sided) |
| Pearson Chi-Square | 7.273^a^ | 2 | .026 |
| Likelihood Ratio | 7.691 | 2 | .021 |
| N of Valid Cases | 209 |  |  |
| a. 0 cells (0.0%) have expected count less than 5. The minimum expected count is 8.57. | | | |

**Geographical category of facility classification * Appointment reminders**

| **Crosstab** | | | | |
| --- | --- | --- | --- | --- |
| Count | | | | |
|  | | Appointment reminders | | Total |
|  |  | Not adopted | Adopted |  |
| Geographical category of facility classification | Urban | 24 | 52 | 76 |
|  | Semi-urban | 17 | 63 | 80 |
|  | Rural | 15 | 38 | 53 |
| Total | | 56 | 153 | 209 |

| **Chi-Square Tests** | | | |
| --- | --- | --- | --- |
|  | Value | Df | Asymptotic Significance (2-sided) |
| Pearson Chi-Square | 2.202^a^ | 2 | .333 |
| Likelihood Ratio | 2.234 | 2 | .327 |
| N of Valid Cases | 209 |  |  |
| a. 0 cells (0.0%) have expected count less than 5. The minimum expected count is 14.20. | | | |

| **Community mobilization * Category of hospital classification Crosstabulation** | | | | | |
| --- | --- | --- | --- | --- | --- |
| Count | | | | | |
|  | | Category of hospital classification | | | Total |
|  |  | Public Hospital | Private Hospital | Faith-Based Hospital/NGO |  |
| Community mobilization | Not adopted | 39 | 33 | 7 | 79 |
|  | Adopted | 62 | 41 | 24 | 127 |
| Total | | 101 | 74 | 31 | 206 |

| **Crosstab** | | | | |
| --- | --- | --- | --- | --- |
| Count | | | | |
|  | | Community mobilization | | Total |
|  |  | Not adopted | Adopted |  |
| Classification level of facility | Level IV | 65 | 103 | 168 |
|  | Level V | 9 | 21 | 30 |
|  | Level VI | 5 | 3 | 8 |
| Total | | 79 | 127 | 206 |

| **Chi-Square Tests** | | | |
| --- | --- | --- | --- |
|  | Value | df | Asymptotic Significance (2-sided) |
| Pearson Chi-Square | 2.866^a^ | 2 | .239 |
| Likelihood Ratio | 2.824 | 2 | .244 |
| N of Valid Cases | 206 |  |  |
| a. 2 cells (33.3%) have expected count less than 5. The minimum expected count is 3.07. | | | |

**Category of hospital classification * Community mobilization**

| **Crosstab** | | | | |
| --- | --- | --- | --- | --- |
| Count | | | | |
|  | | Community mobilization | | Total |
|  |  | Not adopted | Adopted |  |
| Category of hospital classification | Public Hospital | 39 | 62 | 101 |
|  | Private Hospital | 33 | 41 | 74 |
|  | Faith-Based Hospital/NGO | 7 | 24 | 31 |
| Total | | 79 | 127 | 206 |

| **Chi-Square Tests** | | | |
| --- | --- | --- | --- |
|  | Value | df | Asymptotic Significance (2-sided) |
| Pearson Chi-Square | 4.484^a^ | 2 | .106 |
| Likelihood Ratio | 4.720 | 2 | .094 |
| N of Valid Cases | 206 |  |  |
| a. 0 cells (0.0%) have expected count less than 5. The minimum expected count is 11.89. | | | |

**Geographical category of facility classification * Community mobilization**

| **Crosstab** | | | | |
| --- | --- | --- | --- | --- |
| Count | | | | |
|  | | Community mobilization | | Total |
|  |  | Not adopted | Adopted |  |
| Geographical category of facility classification | Urban | 37 | 37 | 74 |
|  | Semi-urban | 24 | 54 | 78 |
|  | Rural | 18 | 36 | 54 |
| Total | | 79 | 127 | 206 |

| **Chi-Square Tests** | | | |
| --- | --- | --- | --- |
|  | Value | df | Asymptotic Significance (2-sided) |
| Pearson Chi-Square | 6.719^a^ | 2 | .035 |
| Likelihood Ratio | 6.670 | 2 | .036 |
| N of Valid Cases | 206 |  |  |
| a. 0 cells (0.0%) have expected count less than 5. The minimum expected count is 20.71. | | | |

| **Awareness raising over health issues * Category of hospital classification Crosstabulation** | | | | | |
| --- | --- | --- | --- | --- | --- |
| Count | | | | | |
|  | | Category of hospital classification | | | Total |
|  |  | Public Hospital | Private Hospital | Faith-Based Hospital/NGO |  |
| Awareness raising over health issues | Not adopted | 43 | 31 | 16 | 90 |
|  | Adopted | 55 | 44 | 15 | 114 |
| Total | | 98 | 75 | 31 | 204 |

| **Crosstab** | | | | |
| --- | --- | --- | --- | --- |
| Count | | | | |
|  | | Awareness raising over health issues | | Total |
|  |  | Not adopted | Adopted |  |
| Classification level of facility | Level IV | 74 | 91 | 165 |
|  | Level V | 11 | 21 | 32 |
|  | Level VI | 5 | 2 | 7 |
| Total | | 90 | 114 | 204 |

| **Chi-Square Tests** | | | |
| --- | --- | --- | --- |
|  | Value | df | Asymptotic Significance (2-sided) |
| Pearson Chi-Square | 3.386^a^ | 2 | .184 |
| Likelihood Ratio | 3.431 | 2 | .180 |
| N of Valid Cases | 204 |  |  |
| a. 2 cells (33.3%) have expected count less than 5. The minimum expected count is 3.09. | | | |

**Category of hospital classification * Awareness raising over health issues**

| **Crosstab** | | | | |
| --- | --- | --- | --- | --- |
| Count | | | | |
|  | | Awareness raising over health issues | | Total |
|  |  | Not adopted | Adopted |  |
| Category of hospital classification | Public Hospital | 43 | 55 | 98 |
|  | Private Hospital | 31 | 44 | 75 |
|  | Faith-Based Hospital/NGO | 16 | 15 | 31 |
| Total | | 90 | 114 | 204 |

| **Chi-Square Tests** | | | |
| --- | --- | --- | --- |
|  | Value | df | Asymptotic Significance (2-sided) |
| Pearson Chi-Square | .945^a^ | 2 | .624 |
| Likelihood Ratio | .940 | 2 | .625 |
| N of Valid Cases | 204 |  |  |
| a. 0 cells (0.0%) have expected count less than 5. The minimum expected count is 13.68. | | | |

**Geographical category of facility classification * Awareness raising over health issues**

| **Crosstab** | | | | |
| --- | --- | --- | --- | --- |
| Count | | | | |
|  | | Awareness raising over health issues | | Total |
|  |  | Not adopted | Adopted |  |
| Geographical category of facility classification | Urban | 36 | 39 | 75 |
|  | Semi-urban | 32 | 44 | 76 |
|  | Rural | 22 | 31 | 53 |
| Total | | 90 | 114 | 204 |

| **Chi-Square Tests** | | | |
| --- | --- | --- | --- |
|  | Value | df | Asymptotic Significance (2-sided) |
| Pearson Chi-Square | .730^a^ | 2 | .694 |
| Likelihood Ratio | .728 | 2 | .695 |
| N of Valid Cases | 204 |  |  |
| a. 0 cells (0.0%) have expected count less than 5. The minimum expected count is 23.38. | | | |

| **Mobile telemedicine * Category of hospital classification Crosstabulation** | | | | | |
| --- | --- | --- | --- | --- | --- |
| Count | | | | | |
|  | | Category of hospital classification | | | Total |
|  |  | Public Hospital | Private Hospital | Faith-Based Hospital/NGO |  |
| Mobile telemedicine | Not adopted | 50 | 33 | 18 | 101 |
|  | Adopted | 51 | 43 | 15 | 109 |
| Total | | 101 | 76 | 33 | 210 |

| **Crosstab** | | | | |
| --- | --- | --- | --- | --- |
| Count | | | | |
|  | | Mobile telemedicine | | Total |
|  |  | Not adopted | Adopted |  |
| Classification level of facility | Level IV | 84 | 85 | 169 |
|  | Level V | 14 | 19 | 33 |
|  | Level VI | 3 | 5 | 8 |
| Total | | 101 | 109 | 210 |

| **Chi-Square Tests** | | | |
| --- | --- | --- | --- |
|  | Value | df | Asymptotic Significance (2-sided) |
| Pearson Chi-Square | .960^a^ | 2 | .619 |
| Likelihood Ratio | .967 | 2 | .617 |
| N of Valid Cases | 210 |  |  |
| a. 2 cells (33.3%) have expected count less than 5. The minimum expected count is 3.85. | | | |

**Category of hospital classification * Mobile telemedicine**

| **Crosstab** | | | | |
| --- | --- | --- | --- | --- |
| Count | | | | |
|  | | Mobile telemedicine | | Total |
|  |  | Not adopted | Adopted |  |
| Category of hospital classification | Public Hospital | 50 | 51 | 101 |
|  | Private Hospital | 33 | 43 | 76 |
|  | Faith-Based Hospital/NGO | 18 | 15 | 33 |
| Total | | 101 | 109 | 210 |

| **Chi-Square Tests** | | | |
| --- | --- | --- | --- |
|  | Value | df | Asymptotic Significance (2-sided) |
| Pearson Chi-Square | 1.296^a^ | 2 | .523 |
| Likelihood Ratio | 1.298 | 2 | .523 |
| N of Valid Cases | 210 |  |  |
| a. 0 cells (0.0%) have expected count less than 5. The minimum expected count is 15.87. | | | |

**Geographical category of facility classification * Mobile telemedicine**

| **Crosstab** | | | | |
| --- | --- | --- | --- | --- |
| Count | | | | |
|  | | Mobile telemedicine | | Total |
|  |  | Not adopted | Adopted |  |
| Geographical category of facility classification | Urban | 41 | 36 | 77 |
|  | Semi-urban | 38 | 42 | 80 |
|  | Rural | 22 | 31 | 53 |
| Total | | 101 | 109 | 210 |

| **Chi-Square Tests** | | | |
| --- | --- | --- | --- |
|  | Value | df | Asymptotic Significance (2-sided) |
| Pearson Chi-Square | 1.751^a^ | 2 | .417 |
| Likelihood Ratio | 1.756 | 2 | .416 |
| N of Valid Cases | 210 |  |  |
| a. 0 cells (0.0%) have expected count less than 5. The minimum expected count is 25.49. | | | |

| **Mobile surveys (surveys by mobile phone) * Category of hospital classification Crosstabulation** | | | | | |
| --- | --- | --- | --- | --- | --- |
| Count | | | | | |
|  | | Category of hospital classification | | | Total |
|  |  | Public Hospital | Private Hospital | Faith-Based Hospital/NGO |  |
| Mobile surveys (surveys by mobile phone) | Not adopted | 74 | 59 | 22 | 155 |
|  | Adopted | 27 | 10 | 7 | 44 |
| Total | | 101 | 69 | 29 | 199 |

| **Crosstab** | | | | |
| --- | --- | --- | --- | --- |
| Count | | | | |
|  | | Mobile surveys (surveys by mobile phone) | | Total |
|  |  | Not adopted | Adopted |  |
| Classification level of facility | Level IV | 131 | 31 | 162 |
|  | Level V | 19 | 13 | 32 |
|  | Level VI | 5 | 0 | 5 |
| Total | | 155 | 44 | 199 |

| **Chi-Square Tests** | | | |
| --- | --- | --- | --- |
|  | Value | df | Asymptotic Significance (2-sided) |
| Pearson Chi-Square | 8.621^a^ | 2 | .013 |
| Likelihood Ratio | 8.863 | 2 | .012 |
| N of Valid Cases | 199 |  |  |
| a. 2 cells (33.3%) have expected count less than 5. The minimum expected count is 1.11. | | | |

**Category of hospital classification * Mobile surveys (surveys by mobile phone)**

| **Crosstab** | | | | |
| --- | --- | --- | --- | --- |
| Count | | | | |
|  | | Mobile surveys (surveys by mobile phone) | | Total |
|  |  | Not adopted | Adopted |  |
| Category of hospital classification | Public Hospital | 74 | 27 | 101 |
|  | Private Hospital | 59 | 10 | 69 |
|  | Faith-Based Hospital/NGO | 22 | 7 | 29 |
| Total | | 155 | 44 | 199 |

| **Chi-Square Tests** | | | |
| --- | --- | --- | --- |
|  | Value | df | Asymptotic Significance (2-sided) |
| Pearson Chi-Square | 3.647^a^ | 2 | .161 |
| Likelihood Ratio | 3.827 | 2 | .148 |
| N of Valid Cases | 199 |  |  |
| a. 0 cells (0.0%) have expected count less than 5. The minimum expected count is 6.41. | | | |

**Geographical category of facility classification * Mobile surveys (surveys by mobile phone)**

| **Crosstab** | | | | |
| --- | --- | --- | --- | --- |
| Count | | | | |
|  | | Mobile surveys (surveys by mobile phone) | | Total |
|  |  | Not adopted | Adopted |  |
| Geographical category of facility classification | Urban | 54 | 19 | 73 |
|  | Semi-urban | 58 | 17 | 75 |
|  | Rural | 43 | 8 | 51 |
| Total | | 155 | 44 | 199 |

| **Chi-Square Tests** | | | |
| --- | --- | --- | --- |
|  | Value | df | Asymptotic Significance (2-sided) |
| Pearson Chi-Square | 1.886^a^ | 2 | .389 |
| Likelihood Ratio | 1.962 | 2 | .375 |
| N of Valid Cases | 199 |  |  |
| a. 0 cells (0.0%) have expected count less than 5. The minimum expected count is 11.28. | | | |

| **Surveillance * Category of hospital classification Crosstabulation** | | | | | |
| --- | --- | --- | --- | --- | --- |
| Count | | | | | |
|  | | Category of hospital classification | | | Total |
|  |  | Public Hospital | Private Hospital | Faith-Based Hospital/NGO |  |
| Surveillance | Not adopted | 54 | 52 | 18 | 124 |
|  | Adopted | 41 | 16 | 12 | 69 |
| Total | | 95 | 68 | 30 | 193 |

| **Crosstab** | | | | |
| --- | --- | --- | --- | --- |
| Count | | | | |
|  | | Surveillance | | Total |
|  |  | Not adopted | Adopted |  |
| Classification level of facility | Level IV | 104 | 53 | 157 |
|  | Level V | 15 | 14 | 29 |
|  | Level VI | 5 | 2 | 7 |
| Total | | 124 | 69 | 193 |

| **Chi-Square Tests** | | | |
| --- | --- | --- | --- |
|  | Value | df | Asymptotic Significance (2-sided) |
| Pearson Chi-Square | 2.409^a^ | 2 | .300 |
| Likelihood Ratio | 2.341 | 2 | .310 |
| N of Valid Cases | 193 |  |  |
| a. 2 cells (33.3%) have expected count less than 5. The minimum expected count is 2.50. | | | |

**Category of hospital classification * Surveillance**

| **Crosstab** | | | | |
| --- | --- | --- | --- | --- |
| Count | | | | |
|  | | Surveillance | | Total |
|  |  | Not adopted | Adopted |  |
| Category of hospital classification | Public Hospital | 54 | 41 | 95 |
|  | Private Hospital | 52 | 16 | 68 |
|  | Faith-Based Hospital/NGO | 18 | 12 | 30 |
| Total | | 124 | 69 | 193 |

| **Chi-Square Tests** | | | |
| --- | --- | --- | --- |
|  | Value | df | Asymptotic Significance (2-sided) |
| Pearson Chi-Square | 6.927^a^ | 2 | .031 |
| Likelihood Ratio | 7.167 | 2 | .028 |
| N of Valid Cases | 193 |  |  |
| a. 0 cells (0.0%) have expected count less than 5. The minimum expected count is 10.73. | | | |

**Geographical category of facility classification * Surveillance**

| **Crosstab** | | | | |
| --- | --- | --- | --- | --- |
| Count | | | | |
|  | | Surveillance | | Total |
|  |  | Not adopted | Adopted |  |
| Geographical category of facility classification | Urban | 45 | 23 | 68 |
|  | Semi-urban | 50 | 26 | 76 |
|  | Rural | 29 | 20 | 49 |
| Total | | 124 | 69 | 193 |

| **Chi-Square Tests** | | | |
| --- | --- | --- | --- |
|  | Value | df | Asymptotic Significance (2-sided) |
| Pearson Chi-Square | .736^a^ | 2 | .692 |
| Likelihood Ratio | .727 | 2 | .695 |
| N of Valid Cases | 193 |  |  |
| a. 0 cells (0.0%) have expected count less than 5. The minimum expected count is 17.52. | | | |

| **Patient monitoring * Category of hospital classification Crosstabulation** | | | | | |
| --- | --- | --- | --- | --- | --- |
| Count | | | | | |
|  | | Category of hospital classification | | | Total |
|  |  | Public Hospital | Private Hospital | Faith-Based Hospital/NGO |  |
| Patient monitoring | Not adopted | 55 | 33 | 12 | 100 |
|  | Adopted | 44 | 41 | 21 | 106 |
| Total | | 99 | 74 | 33 | 206 |

| **Crosstab** | | | | |
| --- | --- | --- | --- | --- |
| Count | | | | |
|  | | Patient monitoring | | Total |
|  |  | Not adopted | Adopted |  |
| Classification level of facility | Level IV | 84 | 82 | 166 |
|  | Level V | 12 | 20 | 32 |
|  | Level VI | 4 | 4 | 8 |
| Total | | 100 | 106 | 206 |

| **Chi-Square Tests** | | | |
| --- | --- | --- | --- |
|  | Value | df | Asymptotic Significance (2-sided) |
| Pearson Chi-Square | 1.851^a^ | 2 | .396 |
| Likelihood Ratio | 1.871 | 2 | .392 |
| N of Valid Cases | 206 |  |  |
| a. 2 cells (33.3%) have expected count less than 5. The minimum expected count is 3.88. | | | |

**Category of hospital classification * Patient monitoring**

| **Crosstab** | | | | |
| --- | --- | --- | --- | --- |
| Count | | | | |
|  | | Patient monitoring | | Total |
|  |  | Not adopted | Adopted |  |
| Category of hospital classification | Public Hospital | 55 | 44 | 99 |
|  | Private Hospital | 33 | 41 | 74 |
|  | Faith-Based Hospital/NGO | 12 | 21 | 33 |
| Total | | 100 | 106 | 206 |

| **Chi-Square Tests** | | | |
| --- | --- | --- | --- |
|  | Value | df | Asymptotic Significance (2-sided) |
| Pearson Chi-Square | 4.371^a^ | 2 | .112 |
| Likelihood Ratio | 4.402 | 2 | .111 |
| N of Valid Cases | 206 |  |  |
| a. 0 cells (0.0%) have expected count less than 5. The minimum expected count is 16.02. | | | |

**Geographical category of facility classification * Patient monitoring**

| **Crosstab** | | | | |
| --- | --- | --- | --- | --- |
| Count | | | | |
|  | | Patient monitoring | | Total |
|  |  | Not adopted | Adopted |  |
| Geographical category of facility classification | Urban | 39 | 35 | 74 |
|  | Semi-urban | 41 | 38 | 79 |
|  | Rural | 20 | 33 | 53 |
| Total | | 100 | 106 | 206 |

| **Chi-Square Tests** | | | |
| --- | --- | --- | --- |
|  | Value | df | Asymptotic Significance (2-sided) |
| Pearson Chi-Square | 3.347^a^ | 2 | .188 |
| Likelihood Ratio | 3.377 | 2 | .185 |
| N of Valid Cases | 206 |  |  |
| a. 0 cells (0.0%) have expected count less than 5. The minimum expected count is 25.73. | | | |

| **Information and decision support systems * Category of hospital classification Crosstabulation** | | | | | |
| --- | --- | --- | --- | --- | --- |
| Count | | | | | |
|  | | Category of hospital classification | | | Total |
|  |  | Public Hospital | Private Hospital | Faith-Based Hospital/NGO |  |
| Information and decision support systems | Not adopted | 56 | 29 | 16 | 101 |
|  | Adopted | 43 | 42 | 16 | 101 |
| Total | | 99 | 71 | 32 | 202 |

| **Crosstab** | | | | |
| --- | --- | --- | --- | --- |
| Count | | | | |
|  | | Information and decision support systems | | Total |
|  |  | Not adopted | Adopted |  |
| Classification level of facility | Level IV | 85 | 81 | 166 |
|  | Level V | 12 | 18 | 30 |
|  | Level VI | 4 | 2 | 6 |
| Total | | 101 | 101 | 202 |

| **Chi-Square Tests** | | | |
| --- | --- | --- | --- |
|  | Value | df | Asymptotic Significance (2-sided) |
| Pearson Chi-Square | 1.963^a^ | 2 | .375 |
| Likelihood Ratio | 1.984 | 2 | .371 |
| N of Valid Cases | 202 |  |  |
| a. 2 cells (33.3%) have expected count less than 5. The minimum expected count is 3.00. | | | |

**Category of hospital classification * Information and decision support systems**

| **Crosstab** | | | | |
| --- | --- | --- | --- | --- |
| Count | | | | |
|  | | Information and decision support systems | | Total |
|  |  | Not adopted | Adopted |  |
| Category of hospital classification | Public Hospital | 56 | 43 | 99 |
|  | Private Hospital | 29 | 42 | 71 |
|  | Faith-Based Hospital/NGO | 16 | 16 | 32 |
| Total | | 101 | 101 | 202 |

| **Chi-Square Tests** | | | |
| --- | --- | --- | --- |
|  | Value | df | Asymptotic Significance (2-sided) |
| Pearson Chi-Square | 4.087^a^ | 2 | .130 |
| Likelihood Ratio | 4.106 | 2 | .128 |
| N of Valid Cases | 202 |  |  |
| a. 0 cells (0.0%) have expected count less than 5. The minimum expected count is 16.00. | | | |

**Geographical category of facility classification * Information and decision support systems**

| **Crosstab** | | | | |
| --- | --- | --- | --- | --- |
| Count | | | | |
|  | | Information and decision support systems | | Total |
|  |  | Not adopted | Adopted |  |
| Geographical category of facility classification | Urban | 37 | 36 | 73 |
|  | Semi-urban | 37 | 40 | 77 |
|  | Rural | 27 | 25 | 52 |
| Total | | 101 | 101 | 202 |

| **Chi-Square Tests** | | | |
| --- | --- | --- | --- |
|  | Value | df | Asymptotic Significance (2-sided) |
| Pearson Chi-Square | .208^a^ | 2 | .901 |
| Likelihood Ratio | .208 | 2 | .901 |
| N of Valid Cases | 202 |  |  |
| a. 0 cells (0.0%) have expected count less than 5. The minimum expected count is 26.00. | | | |

| **Patient records * Category of hospital classification Crosstabulation** | | | | | |
| --- | --- | --- | --- | --- | --- |
| Count | | | | | |
|  | | Category of hospital classification | | | Total |
|  |  | Public Hospital | Private Hospital | Faith-Based Hospital/NGO |  |
| Patient records | Not adopted | 60 | 32 | 17 | 109 |
|  | Adopted | 40 | 41 | 16 | 97 |
| Total | | 100 | 73 | 33 | 206 |

| **Crosstab** | | | | |
| --- | --- | --- | --- | --- |
| Count | | | | |
|  | | Patient records | | Total |
|  |  | Not adopted | Adopted |  |
| Classification level of facility | Level IV | 92 | 76 | 168 |
|  | Level V | 14 | 17 | 31 |
|  | Level VI | 3 | 4 | 7 |
| Total | | 109 | 97 | 206 |

| **Chi-Square Tests** | | | |
| --- | --- | --- | --- |
|  | Value | df | Asymptotic Significance (2-sided) |
| Pearson Chi-Square | 1.262^a^ | 2 | .532 |
| Likelihood Ratio | 1.261 | 2 | .532 |
| N of Valid Cases | 206 |  |  |
| a. 2 cells (33.3%) have expected count less than 5. The minimum expected count is 3.30. | | | |

**Category of hospital classification * Patient records**

| **Crosstab** | | | | |
| --- | --- | --- | --- | --- |
| Count | | | | |
|  | | Patient records | | Total |
|  |  | Not adopted | Adopted |  |
| Category of hospital classification | Public Hospital | 60 | 40 | 100 |
|  | Private Hospital | 32 | 41 | 73 |
|  | Faith-Based Hospital/NGO | 17 | 16 | 33 |
| Total | | 109 | 97 | 206 |

| **Chi-Square Tests** | | | |
| --- | --- | --- | --- |
|  | Value | df | Asymptotic Significance (2-sided) |
| Pearson Chi-Square | 4.456^a^ | 2 | .108 |
| Likelihood Ratio | 4.470 | 2 | .107 |
| N of Valid Cases | 206 |  |  |
| a. 0 cells (0.0%) have expected count less than 5. The minimum expected count is 15.54. | | | |

**Geographical category of facility classification * Patient records**

| **Crosstab** | | | | |
| --- | --- | --- | --- | --- |
| Count | | | | |
|  | | Patient records | | Total |
|  |  | Not adopted | Adopted |  |
| Geographical category of facility classification | Urban | 41 | 32 | 73 |
|  | Semi-urban | 41 | 38 | 79 |
|  | Rural | 27 | 27 | 54 |
| Total | | 109 | 97 | 206 |

| **Chi-Square Tests** | | | |
| --- | --- | --- | --- |
|  | Value | df | Asymptotic Significance (2-sided) |
| Pearson Chi-Square | .526^a^ | 2 | .769 |
| Likelihood Ratio | .527 | 2 | .768 |
| N of Valid Cases | 206 |  |  |
| a. 0 cells (0.0%) have expected count less than 5. The minimum expected count is 25.43. | | | |

1. **If you are using any M-health technologies or platforms, please, indicate the mode of acquisition that the hospital used:**

| **Lease * Category of hospital classification Crosstabulation** | | | | | |
| --- | --- | --- | --- | --- | --- |
| Count | | | | | |
|  | | Category of hospital classification | | | Total |
|  |  | Public Hospital | Private Hospital | Faith-Based Hospital/NGO |  |
| Lease | No | 99 | 62 | 29 | 190 |
|  | Yes | 3 | 13 | 4 | 20 |
|  | Missing variable | 0 | 1 | 0 | 1 |
| Total | | 102 | 76 | 33 | 211 |

| **Crosstab** | | | | | |
| --- | --- | --- | --- | --- | --- |
| Count | | | | | |
|  | | Lease | | | Total |
|  |  | No | Yes | 99 |  |
| Classification level of facility | Level IV | 154 | 15 | 1 | 170 |
|  | Level V | 31 | 2 | 0 | 33 |
|  | Level VI | 5 | 3 | 0 | 8 |
| Total | | 190 | 20 | 1 | 211 |

| **Chi-Square Tests** | | | |
| --- | --- | --- | --- |
|  | Value | df | Asymptotic Significance (2-sided) |
| Pearson Chi-Square | 8.087^a^ | 4 | .088 |
| Likelihood Ratio | 5.562 | 4 | .234 |
| N of Valid Cases | 211 |  |  |
| a. 5 cells (55.6%) have expected count less than 5. The minimum expected count is .04. | | | |

**Category of hospital classification * Lease**

| **Crosstab** | | | | | |
| --- | --- | --- | --- | --- | --- |
| Count | | | | | |
|  | | Lease | | | Total |
|  |  | No | Yes | 99 |  |
| Category of hospital classification | Public Hospital | 99 | 3 | 0 | 102 |
|  | Private Hospital | 62 | 13 | 1 | 76 |
|  | Faith-Based Hospital/NGO | 29 | 4 | 0 | 33 |
| Total | | 190 | 20 | 1 | 211 |

| **Chi-Square Tests** | | | |
| --- | --- | --- | --- |
|  | Value | df | Asymptotic Significance (2-sided) |
| Pearson Chi-Square | 12.462^a^ | 4 | .014 |
| Likelihood Ratio | 13.523 | 4 | .009 |
| N of Valid Cases | 211 |  |  |
| a. 4 cells (44.4%) have expected count less than 5. The minimum expected count is .16. | | | |

**Geographical category of facility classification * Lease**

| **Crosstab** | | | | | |
| --- | --- | --- | --- | --- | --- |
| Count | | | | | |
|  | | Lease | | | Total |
|  |  | No | Yes | 99 |  |
| Geographical category of facility classification | Urban | 66 | 10 | 1 | 77 |
|  | Semi-urban | 73 | 7 | 0 | 80 |
|  | Rural | 51 | 3 | 0 | 54 |
| Total | | 190 | 20 | 1 | 211 |

| **Chi-Square Tests** | | | |
| --- | --- | --- | --- |
|  | Value | Df | Asymptotic Significance (2-sided) |
| Pearson Chi-Square | 3.951^a^ | 4 | .413 |
| Likelihood Ratio | 4.279 | 4 | .370 |
| N of Valid Cases | 211 |  |  |
| a. 3 cells (33.3%) have expected count less than 5. The minimum expected count is .26. | | | |

**Current turnover (in Ksh. Millions) of the hospital per annum * Lease**

| **Crosstab** | | | | | |
| --- | --- | --- | --- | --- | --- |
| Count | | | | | |
|  | | Lease | | | Total |
|  |  | No | Yes | 99 |  |
| Current turnover of the hospital per annum | Less than 100 | 140 | 13 | 1 | 154 |
|  | 100-200 | 22 | 1 | 0 | 23 |
|  | 201-400 | 9 | 0 | 0 | 9 |
|  | 401-600 | 5 | 2 | 0 | 7 |
|  | 601-800 | 1 | 1 | 0 | 2 |
|  | 801 and above | 8 | 2 | 0 | 10 |
|  |  |  |  |  |  |
| Total | | 185 | 19 | 1 | 205 |

| **Chi-Square Tests** | | | |
| --- | --- | --- | --- |
|  | Value | Df | Asymptotic Significance (2-sided) |
| Pearson Chi-Square | 10.972^a^ | 12 | .531 |
| Likelihood Ratio | 9.071 | 12 | .697 |
| N of Valid Cases | 205 |  |  |
| a. 15 cells (71.4%) have expected count less than 5. The minimum expected count is .00. | | | |

| **Fully bought and owned * Category of hospital classification Crosstabulation** | | | | | |
| --- | --- | --- | --- | --- | --- |
| Count | | | | | |
|  | | Category of hospital classification | | | Total |
|  |  | Public Hospital | Private Hospital | Faith-Based Hospital/NGO |  |
| Fully bought and owned | No | 74 | 34 | 17 | 125 |
|  | Yes | 28 | 41 | 16 | 85 |
|  | Missing variable | 0 | 1 | 0 | 1 |
| Total | | 102 | 76 | 33 | 211 |

| **Crosstab** | | | | | |
| --- | --- | --- | --- | --- | --- |
| Count | | | | | |
|  | | Fully bought and owned | | | Total |
|  |  | No | Yes | 99 |  |
| Classification level of facility | Level IV | 104 | 65 | 1 | 170 |
|  | Level V | 15 | 18 | 0 | 33 |
|  | Level VI | 6 | 2 | 0 | 8 |
| Total | | 125 | 85 | 1 | 211 |

| **Chi-Square Tests** | | | |
| --- | --- | --- | --- |
|  | Value | Df | Asymptotic Significance (2-sided) |
| Pearson Chi-Square | 4.050^a^ | 4 | .399 |
| Likelihood Ratio | 4.215 | 4 | .378 |
| N of Valid Cases | 211 |  |  |
| a. 5 cells (55.6%) have expected count less than 5. The minimum expected count is .04. | | | |

**Category of hospital classification * Fully bought and owned**

| **Crosstab** | | | | | |
| --- | --- | --- | --- | --- | --- |
| Count | | | | | |
|  | | Fully bought and owned | | | Total |
|  |  | No | Yes | 99 |  |
| Category of hospital classification | Public Hospital | 74 | 28 | 0 | 102 |
|  | Private Hospital | 34 | 41 | 1 | 76 |
|  | Faith-Based Hospital/NGO | 17 | 16 | 0 | 33 |
| Total | | 125 | 85 | 1 | 211 |

| **Chi-Square Tests** | | | |
| --- | --- | --- | --- |
|  | Value | Df | Asymptotic Significance (2-sided) |
| Pearson Chi-Square | 16.100^a^ | 4 | .003 |
| Likelihood Ratio | 16.582 | 4 | .002 |
| N of Valid Cases | 211 |  |  |
| a. 3 cells (33.3%) have expected count less than 5. The minimum expected count is .16. | | | |

**Geographical category of facility classification * Fully bought and owned**

| **Crosstab** | | | | | |
| --- | --- | --- | --- | --- | --- |
| Count | | | | | |
|  | | Fully bought and owned | | | Total |
|  |  | No | Yes | 99 |  |
| Geographical category of facility classification | Urban | 41 | 35 | 1 | 77 |
|  | Semi-urban | 45 | 35 | 0 | 80 |
|  | Rural | 39 | 15 | 0 | 54 |
| Total | | 125 | 85 | 1 | 211 |

| **Chi-Square Tests** | | | |
| --- | --- | --- | --- |
|  | Value | Df | Asymptotic Significance (2-sided) |
| Pearson Chi-Square | 6.710^a^ | 4 | .152 |
| Likelihood Ratio | 7.135 | 4 | .129 |
| N of Valid Cases | 211 |  |  |
| a. 3 cells (33.3%) have expected count less than 5. The minimum expected count is .26. | | | |

**Current turnover (in Ksh. Millions) of the hospital per annum * Fully bought and owned**

| **Crosstab** | | | | | |
| --- | --- | --- | --- | --- | --- |
| Count | | | | | |
|  | | Fully bought and owned | | | Total |
|  |  | No | Yes | 99 |  |
| Current turnover of the hospital per annum | Less than 100 | 101 | 52 | 1 | 154 |
|  | 100-200 | 7 | 16 | 0 | 23 |
|  | 201-400 | 3 | 6 | 0 | 9 |
|  | 401-600 | 3 | 4 | 0 | 7 |
|  | 601-800 | 0 | 2 | 0 | 2 |
|  | 801 and above | 7 | 3 | 0 | 10 |
|  |  |  |  |  |  |
| Total | | 121 | 83 | 1 | 205 |

| **Chi-Square Tests** | | | |
| --- | --- | --- | --- |
|  | Value | df | Asymptotic Significance (2-sided) |
| Pearson Chi-Square | 18.302^a^ | 12 | .107 |
| Likelihood Ratio | 19.385 | 12 | .080 |
| N of Valid Cases | 205 |  |  |
| a. 15 cells (71.4%) have expected count less than 5. The minimum expected count is .00. | | | |

| **Not applicable * Category of hospital classification Crosstabulation** | | | | | |
| --- | --- | --- | --- | --- | --- |
| Count | | | | | |
|  | | Category of hospital classification | | | Total |
|  |  | Public Hospital | Private Hospital | Faith-Based Hospital/NGO |  |
| Not applicable | No | 50 | 53 | 19 | 122 |
|  | Yes | 52 | 22 | 14 | 88 |
|  | Missing variable | 0 | 1 | 0 | 1 |
| Total | | 102 | 76 | 33 | 211 |

| **Crosstab** | | | | | |
| --- | --- | --- | --- | --- | --- |
| Count | | | | | |
|  | | Not applicable | | | Total |
|  |  | No | Yes | 99 |  |
| Classification level of facility | Level IV | 97 | 72 | 1 | 170 |
|  | Level V | 20 | 13 | 0 | 33 |
|  | Level VI | 5 | 3 | 0 | 8 |
| Total | | 122 | 88 | 1 | 211 |

| **Chi-Square Tests** | | | |
| --- | --- | --- | --- |
|  | Value | df | Asymptotic Significance (2-sided) |
| Pearson Chi-Square | .426^a^ | 4 | .980 |
| Likelihood Ratio | .618 | 4 | .961 |
| N of Valid Cases | 211 |  |  |
| a. 5 cells (55.6%) have expected count less than 5. The minimum expected count is .04. | | | |

**Category of hospital classification * Not applicable**

| **Crosstab** | | | | | |
| --- | --- | --- | --- | --- | --- |
| Count | | | | | |
|  | | Not applicable | | | Total |
|  |  | No | Yes | 99 |  |
| Category of hospital classification | Public Hospital | 50 | 52 | 0 | 102 |
|  | Private Hospital | 53 | 22 | 1 | 76 |
|  | Faith-Based Hospital/NGO | 19 | 14 | 0 | 33 |
| Total | | 122 | 88 | 1 | 211 |

| **Chi-Square Tests** | | | |
| --- | --- | --- | --- |
|  | Value | df | Asymptotic Significance (2-sided) |
| Pearson Chi-Square | 10.084^a^ | 4 | .039 |
| Likelihood Ratio | 10.527 | 4 | .032 |
| N of Valid Cases | 211 |  |  |
| a. 3 cells (33.3%) have expected count less than 5. The minimum expected count is .16. | | | |

**Geographical category of facility classification * Not applicable**

| **Crosstab** | | | | | |
| --- | --- | --- | --- | --- | --- |
| Count | | | | | |
|  | | Not applicable | | | Total |
|  |  | No | Yes | 99 |  |
| Geographical category of facility classification | Urban | 49 | 27 | 1 | 77 |
|  | Semi-urban | 44 | 36 | 0 | 80 |
|  | Rural | 29 | 25 | 0 | 54 |
| Total | | 122 | 88 | 1 | 211 |

| **Chi-Square Tests** | | | |
| --- | --- | --- | --- |
|  | Value | df | Asymptotic Significance (2-sided) |
| Pearson Chi-Square | 3.754^a^ | 4 | .440 |
| Likelihood Ratio | 4.054 | 4 | .399 |
| N of Valid Cases | 211 |  |  |
| a. 3 cells (33.3%) have expected count less than 5. The minimum expected count is .26. | | | |

**Current turnover (in Ksh. Millions) of the hospital per annum * Not applicable**

| **Crosstab** | | | | | |
| --- | --- | --- | --- | --- | --- |
| Count | | | | | |
|  | | Not applicable | | | Total |
|  |  | No | Yes | 99 |  |
| Current turnover of the hospital per annum | Less than 100 | 78 | 75 | 1 | 154 |
|  | 100-200 | 19 | 4 | 0 | 23 |
|  | 201-400 | 6 | 3 | 0 | 9 |
|  | 401-600 | 6 | 1 | 0 | 7 |
|  | 601-800 | 2 | 0 | 0 | 2 |
|  | 801 and above | 6 | 4 | 0 | 10 |
|  |  |  |  |  |  |
| Total | | 117 | 87 | 1 | 205 |

| **Chi-Square Tests** | | | |
| --- | --- | --- | --- |
|  | Value | df | Asymptotic Significance (2-sided) |
| Pearson Chi-Square | 14.697^a^ | 12 | .258 |
| Likelihood Ratio | 16.996 | 12 | .150 |
| N of Valid Cases | 205 |  |  |
| a. 15 cells (71.4%) have expected count less than 5. The minimum expected count is .00. | | | |

| **Self developed * Category of hospital classification Crosstabulation** | | | | | |
| --- | --- | --- | --- | --- | --- |
| Count | | | | | |
|  | | Category of hospital classification | | | Total |
|  |  | Public Hospital | Private Hospital | Faith-Based Hospital/NGO |  |
| Self developed | No | 98 | 74 | 33 | 205 |
|  | Yes | 4 | 1 | 0 | 5 |
| Total | | 102 | 75 | 33 | 210 |

| **Crosstab** | | | | |
| --- | --- | --- | --- | --- |
| Count | | | | |
|  | | Self developed | | Total |
|  |  | No | Yes |  |
| Classification level of facility | Level IV | 165 | 4 | 169 |
|  | Level V | 32 | 1 | 33 |
|  | Level VI | 8 | 0 | 8 |
| Total | | 205 | 5 | 210 |

| **Chi-Square Tests** | | | |
| --- | --- | --- | --- |
|  | Value | df | Asymptotic Significance (2-sided) |
| Pearson Chi-Square | .255^a^ | 2 | .880 |
| Likelihood Ratio | .441 | 2 | .802 |
| N of Valid Cases | 210 |  |  |
| a. 3 cells (50.0%) have expected count less than 5. The minimum expected count is .19. | | | |

**Category of hospital classification * Self developed**

| **Crosstab** | | | | |
| --- | --- | --- | --- | --- |
| Count | | | | |
|  | | Self developed | | Total |
|  |  | No | Yes |  |
| Category of hospital classification | Public Hospital | 98 | 4 | 102 |
|  | Private Hospital | 74 | 1 | 75 |
|  | Faith-Based Hospital/NGO | 33 | 0 | 33 |
| Total | | 205 | 5 | 210 |

| **Chi-Square Tests** | | | |
| --- | --- | --- | --- |
|  | Value | df | Asymptotic Significance (2-sided) |
| Pearson Chi-Square | 2.201^a^ | 2 | .333 |
| Likelihood Ratio | 2.885 | 2 | .236 |
| N of Valid Cases | 210 |  |  |
| a. 3 cells (50.0%) have expected count less than 5. The minimum expected count is .79. | | | |

**Geographical category of facility classification * Self developed**

| **Crosstab** | | | | |
| --- | --- | --- | --- | --- |
| Count | | | | |
|  | | Self developed | | Total |
|  |  | No | Yes |  |
| Geographical category of facility classification | Urban | 75 | 1 | 76 |
|  | Semi-urban | 76 | 4 | 80 |
|  | Rural | 54 | 0 | 54 |
| Total | | 205 | 5 | 210 |

| **Chi-Square Tests** | | | |
| --- | --- | --- | --- |
|  | Value | df | Asymptotic Significance (2-sided) |
| Pearson Chi-Square | 4.049^a^ | 2 | .132 |
| Likelihood Ratio | 4.846 | 2 | .089 |
| N of Valid Cases | 210 |  |  |
| a. 3 cells (50.0%) have expected count less than 5. The minimum expected count is 1.29. | | | |

**Current turnover (in Ksh. Millions) of the hospital per annum * Self developed**

| **Crosstab** | | | | |
| --- | --- | --- | --- | --- |
| Count | | | | |
|  | | Self developed | | Total |
|  |  | No | Yes |  |
| Current turnover of the hospital per annum | Less than 100 | 151 | 2 | 153 |
|  | 100-200 | 22 | 1 | 23 |
|  | 201-400 | 9 | 0 | 9 |
|  | 401-600 | 7 | 0 | 7 |
|  | 601-800 | 1 | 1 | 2 |
|  | 801 and above | 10 | 0 | 10 |
|  |  |  |  |  |
| Total | | 200 | 4 | 204 |

| **Chi-Square Tests** | | | |
| --- | --- | --- | --- |
|  | Value | df | Asymptotic Significance (2-sided) |
| Pearson Chi-Square | 25.552^a^ | 6 | .000 |
| Likelihood Ratio | 7.053 | 6 | .316 |
| N of Valid Cases | 204 |  |  |
| a. 9 cells (64.3%) have expected count less than 5. The minimum expected count is .02. | | | |

1. **If you are not using any M-Health technology or platforms, please, indicate which mode of acquisition would be conducive for your decision to adopt M-health**

| **Mode of acquisition * Category of hospital classification Crosstabulation** | | | | | |
| --- | --- | --- | --- | --- | --- |
| Count | | | | | |
|  | | Category of hospital classification | | | Total |
|  |  | Public Hospital | Private Hospital | Faith-Based Hospital/NGO |  |
| Mode of acquisition | Leased | 11 | 12 | 4 | 27 |
|  | Fully bought and owned | 55 | 25 | 15 | 95 |
|  | No applicable | 31 | 35 | 13 | 79 |
|  | Missing variable | 5 | 4 | 1 | 10 |
| Total | | 102 | 76 | 33 | 211 |

| **Crosstab** | | | | | | |
| --- | --- | --- | --- | --- | --- | --- |
| Count | | | | | | |
|  | | Mode of acquisition | | | | Total |
|  |  | Leased | Full bought and owned | No applicable | 99 |  |
| Classification level of facility | Level IV | 25 | 73 | 62 | 10 | 170 |
|  | Level V | 2 | 18 | 13 | 0 | 33 |
|  | Level VI | 0 | 4 | 4 | 0 | 8 |
| Total | | 27 | 95 | 79 | 10 | 211 |

| **Chi-Square Tests** | | | |
| --- | --- | --- | --- |
|  | Value | df | Asymptotic Significance (2-sided) |
| Pearson Chi-Square | 6.376^a^ | 6 | .382 |
| Likelihood Ratio | 9.498 | 6 | .147 |
| N of Valid Cases | 211 |  |  |
| a. 6 cells (50.0%) have expected count less than 5. The minimum expected count is .38. | | | |

**Category of hospital classification * Mode of acquisition**

| **Crosstab** | | | | | | |
| --- | --- | --- | --- | --- | --- | --- |
| Count | | | | | | |
|  | | Mode of acquisition | | | | Total |
|  |  | Leased | Full bought and owned | No applicable | 99 |  |
| Category of hospital classification | Public Hospital | 11 | 55 | 31 | 5 | 102 |
|  | Private Hospital | 12 | 25 | 35 | 4 | 76 |
|  | Faith-Based Hospital/NGO | 4 | 15 | 13 | 1 | 33 |
| Total | | 27 | 95 | 79 | 10 | 211 |

| **Chi-Square Tests** | | | |
| --- | --- | --- | --- |
|  | Value | df | Asymptotic Significance (2-sided) |
| Pearson Chi-Square | 8.290^a^ | 6 | .218 |
| Likelihood Ratio | 8.441 | 6 | .208 |
| N of Valid Cases | 211 |  |  |
| a. 4 cells (33.3%) have expected count less than 5. The minimum expected count is 1.56. | | | |

**Geographical category of facility classification * Mode of acquisition**

| **Crosstab** | | | | | | |
| --- | --- | --- | --- | --- | --- | --- |
| Count | | | | | | |
|  | | Mode of acquisition | | | | Total |
|  |  | Leased | Full bought and owned | No applicable | 99 |  |
| Geographical category of facility classification | Urban | 11 | 33 | 30 | 3 | 77 |
|  | Semi-urban | 12 | 37 | 25 | 6 | 80 |
|  | Rural | 4 | 25 | 24 | 1 | 54 |
| Total | | 27 | 95 | 79 | 10 | 211 |

| **Chi-Square Tests** | | | |
| --- | --- | --- | --- |
|  | Value | df | Asymptotic Significance (2-sided) |
| Pearson Chi-Square | 5.715^a^ | 6 | .456 |
| Likelihood Ratio | 6.025 | 6 | .420 |
| N of Valid Cases | 211 |  |  |
| a. 3 cells (25.0%) have expected count less than 5. The minimum expected count is 2.56. | | | |

**Current turnover of the hospital per annum * Mode of acquisition**

| **Crosstab** | | | | | | |
| --- | --- | --- | --- | --- | --- | --- |
| Count | | | | | | |
|  | | Mode of acquisition | | | | Total |
|  |  | Leased | Full bought and owned | No applicable | 99 |  |
| Current turnover of the hospital per annum | Less than 100 | 22 | 77 | 50 | 5 | 154 |
|  | 100-200 | 3 | 7 | 12 | 1 | 23 |
|  | 201-400 | 0 | 4 | 5 | 0 | 9 |
|  | 401-600 | 1 | 0 | 5 | 1 | 7 |
|  | 601-800 | 0 | 1 | 1 | 0 | 2 |
|  | 801 and above | 1 | 5 | 3 | 1 | 10 |
| Total | | 27 | 94 | 76 | 8 | 205 |

| **Chi-Square Tests** | | | |
| --- | --- | --- | --- |
|  | Value | df | Asymptotic Significance (2-sided) |
| Pearson Chi-Square | 15.782^a^ | 15 | .397 |
| Likelihood Ratio | 19.232 | 15 | .203 |
| N of Valid Cases | 205 |  |  |
| a. 18 cells (75.0%) have expected count less than 5. The minimum expected count is .08. | | | |

TECHNOLOGICAL DETERMINANTS

1. **Rate the extent to which you agree with the following statements relating the effect of the technological determinants listed below on adoption or non-adoption of M-Health using 1 = Strongly Disagree, 2=Disagree, 3=Agree and 4=Strongly Agree.**

Relative Advantage

| **Use of M-health is superior and more advantageous than current manual practices. * Category of hospital classification Crosstabulation** | | | | | |
| --- | --- | --- | --- | --- | --- |
| Count | | | | | |
|  | | Category of hospital classification | | | Total |
|  |  | Public Hospital | Private Hospital | Faith-Based Hospital/NGO |  |
| Use of M-health is superior and more advantageous than current manual practices. | Strongly disagree | 8 | 2 | 1 | 11 |
|  | Disagree | 11 | 4 | 1 | 16 |
|  | Agree | 44 | 29 | 13 | 86 |
|  | Strongly Agree | 38 | 40 | 17 | 95 |
| Total | | 101 | 75 | 32 | 208 |

| **Crosstab** | | | | | | |
| --- | --- | --- | --- | --- | --- | --- |
| Count | | | | | | |
|  | | Use of M-health is superior and more advantageous than current manual practices. | | | | Total |
|  |  | Strongly disgree | Disagree | Agree | Strongly Agree |  |
| Classification level of facility | Level IV | 9 | 14 | 67 | 78 | 168 |
|  | Level V | 2 | 1 | 14 | 15 | 32 |
|  | Level VI | 0 | 1 | 5 | 2 | 8 |
| Total | | 11 | 16 | 86 | 95 | 208 |

| **Chi-Square Tests** | | | |
| --- | --- | --- | --- |
|  | Value | df | Asymptotic Significance (2-sided) |
| Pearson Chi-Square | 3.456^a^ | 6 | .750 |
| Likelihood Ratio | 4.119 | 6 | .661 |
| Linear-by-Linear Association | .037 | 1 | .847 |
| N of Valid Cases | 208 |  |  |
| a. 6 cells (50.0%) have expected count less than 5. The minimum expected count is .42. | | | |

**Category of hospital classification * Use of M-health is superior and more advantageous than current manual practices.**

| **Crosstab** | | | | | | |
| --- | --- | --- | --- | --- | --- | --- |
| Count | | | | | | |
|  | | Use of M-health is superior and more advantageous than current manual practices. | | | | Total |
|  |  | Strongly disgree | Disagree | Agree | Strongly Agree |  |
| Category of hospital classification | Public Hospital | 8 | 11 | 44 | 38 | 101 |
|  | Private Hospital | 2 | 4 | 29 | 40 | 75 |
|  | Faith-Based Hospital/NGO | 1 | 1 | 13 | 17 | 32 |
| Total | | 11 | 16 | 86 | 95 | 208 |

| **Chi-Square Tests** | | | |
| --- | --- | --- | --- |
|  | Value | df | Asymptotic Significance (2-sided) |
| Pearson Chi-Square | 8.375^a^ | 6 | .212 |
| Likelihood Ratio | 8.655 | 6 | .194 |
| Linear-by-Linear Association | 6.474 | 1 | .011 |
| N of Valid Cases | 208 |  |  |
| a. 3 cells (25.0%) have expected count less than 5. The minimum expected count is 1.69. | | | |

**Geographical category of facility classification * Use of M-health is superior and more advantageous than current manual practices.**

| **Crosstab** | | | | | | |
| --- | --- | --- | --- | --- | --- | --- |
| Count | | | | | | |
|  | | Use of M-health is superior and more advantageous than current manual practices. | | | | Total |
|  |  | Strongly disgree | Disagree | Agree | Strongly Agree |  |
| Geographical category of facility classification | Urban | 3 | 4 | 31 | 37 | 75 |
|  | Semi-urban | 6 | 5 | 33 | 36 | 80 |
|  | Rural | 2 | 7 | 22 | 22 | 53 |
| Total | | 11 | 16 | 86 | 95 | 208 |

| **Chi-Square Tests** | | | |
| --- | --- | --- | --- |
|  | Value | df | Asymptotic Significance (2-sided) |
| Pearson Chi-Square | 4.490^a^ | 6 | .611 |
| Likelihood Ratio | 4.159 | 6 | .655 |
| N of Valid Cases | 208 |  |  |
| a. 4 cells (33.3%) have expected count less than 5. The minimum expected count is 2.80. | | | |

**Current annual number of patients (outpatients and in-patients) * Use of M-health is superior and more advantageous than current manual practices.**

| **Crosstab** | | | | | | |
| --- | --- | --- | --- | --- | --- | --- |
| Count | | | | | | |
|  | | Use of M-health is superior and more advantageous than current manual practices. | | | | Total |
|  |  | Strongly disgree | Disagree | Agree | Strongly Agree |  |
| Current annual number of patients (outpatients and in-patients) | Less than 200 | 3 | 5 | 19 | 22 | 49 |
|  | 200-500 | 4 | 4 | 27 | 19 | 54 |
|  | 501-1000 | 0 | 1 | 9 | 14 | 24 |
|  | 1001-1500 | 3 | 0 | 1 | 9 | 13 |
|  | 1501-2000 | 0 | 2 | 7 | 6 | 15 |
|  | 2001 and above | 1 | 4 | 20 | 24 | 49 |
| Total | | 11 | 16 | 83 | 94 | 204 |

| **Chi-Square Tests** | | | |
| --- | --- | --- | --- |
|  | Value | df | Asymptotic Significance (2-sided) |
| Pearson Chi-Square | 22.272^a^ | 15 | .101 |
| Likelihood Ratio | 23.759 | 15 | .069 |
| Linear-by-Linear Association | 1.265 | 1 | .261 |
| N of Valid Cases | 204 |  |  |
| a. 12 cells (50.0%) have expected count less than 5. The minimum expected count is .70. | | | |

**Number of staff * Use of M-health is superior and more advantageous than current manual practices.**

| **Crosstab** | | | | | | |
| --- | --- | --- | --- | --- | --- | --- |
| Count | | | | | | |
|  | | Use of M-health is superior and more advantageous than current manual practices. | | | | Total |
|  |  | Strongly disgree | Disagree | Agree | Strongly Agree |  |
| Number of staff | Less than 100 | 8 | 10 | 52 | 54 | 124 |
|  | 100-200 | 2 | 4 | 16 | 27 | 49 |
|  | 201-300 | 0 | 0 | 5 | 8 | 13 |
|  | 301-500 | 0 | 1 | 6 | 5 | 12 |
|  | 501 and above | 1 | 1 | 7 | 1 | 10 |
| Total | | 11 | 16 | 86 | 95 | 208 |

| **Chi-Square Tests** | | | |
| --- | --- | --- | --- |
|  | Value | df | Asymptotic Significance (2-sided) |
| Pearson Chi-Square | 11.063^a^ | 12 | .524 |
| Likelihood Ratio | 14.176 | 12 | .290 |
| Linear-by-Linear Association | .032 | 1 | .857 |
| N of Valid Cases | 208 |  |  |
| a. 11 cells (55.0%) have expected count less than 5. The minimum expected count is .53. | | | |

**County * Use of M-health is superior and more advantageous than current manual practices.**

| **Crosstab** | | | | | | |
| --- | --- | --- | --- | --- | --- | --- |
| Count | | | | | | |
|  | | Use of M-health is superior and more advantageous than current manual practices. | | | | Total |
|  |  | Strongly disgree | Disagree | Agree | Strongly Agree |  |
| County | No | 3 | 2 | 16 | 14 | 35 |
|  | Yes | 8 | 14 | 70 | 81 | 173 |
| Total | | 11 | 16 | 86 | 95 | 208 |

| **Chi-Square Tests** | | | |
| --- | --- | --- | --- |
|  | Value | df | Asymptotic Significance (2-sided) |
| Pearson Chi-Square | 1.562^a^ | 3 | .668 |
| Likelihood Ratio | 1.477 | 3 | .688 |
| N of Valid Cases | 208 |  |  |
| a. 2 cells (25.0%) have expected count less than 5. The minimum expected count is 1.85. | | | |

**Country * Use of M-health is superior and more advantageous than current manual practices.**

| **Crosstab** | | | | | | |
| --- | --- | --- | --- | --- | --- | --- |
| Count | | | | | | |
|  | | Use of M-health is superior and more advantageous than current manual practices. | | | | Total |
|  |  | Strongly disgree | Disagree | Agree | Strongly Agree |  |
| Country | No | 3 | 6 | 26 | 25 | 60 |
|  | Yes | 8 | 10 | 60 | 69 | 147 |
|  | 2 | 0 | 0 | 0 | 1 | 1 |
| Total | | 11 | 16 | 86 | 95 | 208 |

| **Chi-Square Tests** | | | |
| --- | --- | --- | --- |
|  | Value | df | Asymptotic Significance (2-sided) |
| Pearson Chi-Square | 2.102^a^ | 6 | .910 |
| Likelihood Ratio | 2.455 | 6 | .873 |
| N of Valid Cases | 208 |  |  |
| a. 6 cells (50.0%) have expected count less than 5. The minimum expected count is .05. | | | |

**Eastern Africa * Use of M-health is superior and more advantageous than current manual practices.**

| **Crosstab** | | | | | | |
| --- | --- | --- | --- | --- | --- | --- |
| Count | | | | | | |
|  | | Use of M-health is superior and more advantageous than current manual practices. | | | | Total |
|  |  | Strongly disgree | Disagree | Agree | Strongly Agree |  |
| Eastern Africa | No | 7 | 9 | 56 | 56 | 128 |
|  | Yes | 4 | 7 | 29 | 38 | 78 |
|  | 2 | 0 | 0 | 1 | 1 | 2 |
| Total | | 11 | 16 | 86 | 95 | 208 |

| **Chi-Square Tests** | | | |
| --- | --- | --- | --- |
|  | Value | df | Asymptotic Significance (2-sided) |
| Pearson Chi-Square | 1.323^a^ | 6 | .970 |
| Likelihood Ratio | 1.580 | 6 | .954 |
| N of Valid Cases | 208 |  |  |
| a. 5 cells (41.7%) have expected count less than 5. The minimum expected count is .11. | | | |

| **M-Health improves efficiency of hospital’s operations * Category of hospital classification Crosstabulation** | | | | | |
| --- | --- | --- | --- | --- | --- |
| Count | | | | | |
|  | | Category of hospital classification | | | Total |
|  |  | Public Hospital | Private Hospital | Faith-Based Hospital/NGO |  |
| M-Health improves efficiency of hospital’s operations | Strongly disagree | 4 | 2 | 0 | 6 |
|  | Disagree | 4 | 6 | 4 | 14 |
|  | Agree | 43 | 25 | 11 | 79 |
|  | Strongly Agree | 50 | 43 | 18 | 111 |
| Total | | 101 | 76 | 33 | 210 |

| **Crosstab** | | | | | | |
| --- | --- | --- | --- | --- | --- | --- |
| Count | | | | | | |
|  | | M-Health improves efficiency of hospital’s operations | | | | Total |
|  |  | Strongly disgree | Disagree | Agree | Strongly Agree |  |
| Classification level of facility | Level IV | 5 | 10 | 62 | 92 | 169 |
|  | Level V | 1 | 3 | 15 | 14 | 33 |
|  | Level VI | 0 | 1 | 2 | 5 | 8 |
| Total | | 6 | 14 | 79 | 111 | 210 |

| **Chi-Square Tests** | | | |
| --- | --- | --- | --- |
|  | Value | df | Asymptotic Significance (2-sided) |
| Pearson Chi-Square | 2.896^a^ | 6 | .822 |
| Likelihood Ratio | 3.069 | 6 | .800 |
| Linear-by-Linear Association | .273 | 1 | .601 |
| N of Valid Cases | 210 |  |  |
| a. 7 cells (58.3%) have expected count less than 5. The minimum expected count is .23. | | | |

**Category of hospital classification * M-Health improves efficiency of hospital’s operations**

| **Crosstab** | | | | | | |
| --- | --- | --- | --- | --- | --- | --- |
| Count | | | | | | |
|  | | M-Health improves efficiency of hospital’s operations | | | | Total |
|  |  | Strongly disgree | Disagree | Agree | Strongly Agree |  |
| Category of hospital classification | Public Hospital | 4 | 4 | 43 | 50 | 101 |
|  | Private Hospital | 2 | 6 | 25 | 43 | 76 |
|  | Faith-Based Hospital/NGO | 0 | 4 | 11 | 18 | 33 |
| Total | | 6 | 14 | 79 | 111 | 210 |

| **Chi-Square Tests** | | | |
| --- | --- | --- | --- |
|  | Value | df | Asymptotic Significance (2-sided) |
| Pearson Chi-Square | 5.844^a^ | 6 | .441 |
| Likelihood Ratio | 6.631 | 6 | .356 |
| Linear-by-Linear Association | .202 | 1 | .653 |
| N of Valid Cases | 210 |  |  |
| a. 4 cells (33.3%) have expected count less than 5. The minimum expected count is .94. | | | |

**Geographical category of facility classification * M-Health improves efficiency of hospital’s operations**

| **Crosstab** | | | | | | |
| --- | --- | --- | --- | --- | --- | --- |
| Count | | | | | | |
|  | | M-Health improves efficiency of hospital’s operations | | | | Total |
|  |  | Strongly disgree | Disagree | Agree | Strongly Agree |  |
| Geographical category of facility classification | Urban | 3 | 6 | 33 | 35 | 77 |
|  | Semi-urban | 3 | 4 | 27 | 46 | 80 |
|  | Rural | 0 | 4 | 19 | 30 | 53 |
| Total | | 6 | 14 | 79 | 111 | 210 |

| **Chi-Square Tests** | | | |
| --- | --- | --- | --- |
|  | Value | df | Asymptotic Significance (2-sided) |
| Pearson Chi-Square | 4.759^a^ | 6 | .575 |
| Likelihood Ratio | 6.260 | 6 | .395 |
| N of Valid Cases | 210 |  |  |
| a. 4 cells (33.3%) have expected count less than 5. The minimum expected count is 1.51. | | | |

**Current annual number of patients (outpatients and in-patients) * M-Health improves efficiency of hospital’s operations**

| **Crosstab** | | | | | | |
| --- | --- | --- | --- | --- | --- | --- |
| Count | | | | | | |
|  | | M-Health improves efficiency of hospital’s operations | | | | Total |
|  |  | Strongly disgree | Disagree | Agree | Strongly Agree |  |
| Current annual number of patients (outpatients and in-patients) | Less than 200 | 0 | 3 | 18 | 28 | 49 |
|  | 200-500 | 3 | 5 | 22 | 25 | 55 |
|  | 501-1000 | 0 | 2 | 7 | 15 | 24 |
|  | 1001-1500 | 2 | 1 | 4 | 6 | 13 |
|  | 1501-2000 | 0 | 2 | 4 | 9 | 15 |
|  | 2001 and above | 1 | 1 | 21 | 27 | 50 |
| Total | | 6 | 14 | 76 | 110 | 206 |

| **Chi-Square Tests** | | | |
| --- | --- | --- | --- |
|  | Value | df | Asymptotic Significance (2-sided) |
| Pearson Chi-Square | 16.910^a^ | 15 | .324 |
| Likelihood Ratio | 16.192 | 15 | .369 |
| Linear-by-Linear Association | .105 | 1 | .746 |
| N of Valid Cases | 206 |  |  |
| a. 13 cells (54.2%) have expected count less than 5. The minimum expected count is .38. | | | |

**Number of staff * M-Health improves efficiency of hospital’s operations**

| **Crosstab** | | | | | | |
| --- | --- | --- | --- | --- | --- | --- |
| Count | | | | | | |
|  | | M-Health improves efficiency of hospital’s operations | | | | Total |
|  |  | Strongly disgree | Disagree | Agree | Strongly Agree |  |
| Number of staff | Less than 100 | 5 | 8 | 47 | 64 | 124 |
|  | 100-200 | 0 | 5 | 19 | 27 | 51 |
|  | 201-300 | 0 | 0 | 3 | 10 | 13 |
|  | 301-500 | 0 | 0 | 5 | 7 | 12 |
|  | 501 and above | 1 | 1 | 5 | 3 | 10 |
| Total | | 6 | 14 | 79 | 111 | 210 |

| **Chi-Square Tests** | | | |
| --- | --- | --- | --- |
|  | Value | df | Asymptotic Significance (2-sided) |
| Pearson Chi-Square | 10.864^a^ | 12 | .541 |
| Likelihood Ratio | 13.889 | 12 | .308 |
| Linear-by-Linear Association | .008 | 1 | .928 |
| N of Valid Cases | 210 |  |  |
| a. 12 cells (60.0%) have expected count less than 5. The minimum expected count is .29. | | | |

**County * M-Health improves efficiency of hospital’s operations**

| **Crosstab** | | | | | | |
| --- | --- | --- | --- | --- | --- | --- |
| Count | | | | | | |
|  | | M-Health improves efficiency of hospital’s operations | | | | Total |
|  |  | Strongly disgree | Disagree | Agree | Strongly Agree |  |
| County | No | 3 | 6 | 11 | 16 | 36 |
|  | Yes | 3 | 8 | 68 | 95 | 174 |
| Total | | 6 | 14 | 79 | 111 | 210 |

| **Chi-Square Tests** | | | |
| --- | --- | --- | --- |
|  | Value | df | Asymptotic Significance (2-sided) |
| Pearson Chi-Square | 12.236^a^ | 3 | .007 |
| Likelihood Ratio | 9.659 | 3 | .022 |
| N of Valid Cases | 210 |  |  |
| a. 3 cells (37.5%) have expected count less than 5. The minimum expected count is 1.03. | | | |

**Country * M-Health improves efficiency of hospital’s operations**

| **Crosstab** | | | | | | |
| --- | --- | --- | --- | --- | --- | --- |
| Count | | | | | | |
|  | | M-Health improves efficiency of hospital’s operations | | | | Total |
|  |  | Strongly disgree | Disagree | Agree | Strongly Agree |  |
| Country | No | 3 | 2 | 27 | 29 | 61 |
|  | Yes | 3 | 12 | 52 | 81 | 148 |
|  | 2 | 0 | 0 | 0 | 1 | 1 |
| Total | | 6 | 14 | 79 | 111 | 210 |

| **Chi-Square Tests** | | | |
| --- | --- | --- | --- |
|  | Value | df | Asymptotic Significance (2-sided) |
| Pearson Chi-Square | 5.050^a^ | 6 | .537 |
| Likelihood Ratio | 5.503 | 6 | .481 |
| N of Valid Cases | 210 |  |  |
| a. 7 cells (58.3%) have expected count less than 5. The minimum expected count is .03. | | | |

**Eastern Africa * M-Health improves efficiency of hospital’s operations**

| **Crosstab** | | | | | | |
| --- | --- | --- | --- | --- | --- | --- |
| Count | | | | | | |
|  | | M-Health improves efficiency of hospital’s operations | | | | Total |
|  |  | Strongly disgree | Disagree | Agree | Strongly Agree |  |
| Eastern Africa | No | 6 | 9 | 48 | 66 | 129 |
|  | Yes | 0 | 5 | 30 | 44 | 79 |
|  | 2 | 0 | 0 | 1 | 1 | 2 |
| Total | | 6 | 14 | 79 | 111 | 210 |

| **Chi-Square Tests** | | | |
| --- | --- | --- | --- |
|  | Value | df | Asymptotic Significance (2-sided) |
| Pearson Chi-Square | 4.216^a^ | 6 | .647 |
| Likelihood Ratio | 6.421 | 6 | .378 |
| N of Valid Cases | 210 |  |  |
| a. 6 cells (50.0%) have expected count less than 5. The minimum expected count is .06. | | | |

| **M-health will reduce the cost of health care to patients * Category of hospital classification Crosstabulation** | | | | | |
| --- | --- | --- | --- | --- | --- |
| Count | | | | | |
|  | | Category of hospital classification | | | Total |
|  |  | Public Hospital | Private Hospital | Faith-Based Hospital/NGO |  |
| M-health will reduce the cost of health care to patients | Strongly disagree | 4 | 3 | 1 | 8 |
|  | Disagree | 14 | 13 | 5 | 32 |
|  | Agree | 36 | 26 | 9 | 71 |
|  | Strongly Agree | 47 | 29 | 16 | 92 |
| Total | | 101 | 71 | 31 | 203 |

| **Crosstab** | | | | | | |
| --- | --- | --- | --- | --- | --- | --- |
| Count | | | | | | |
|  | | M-health will reduce the cost of health care to patients | | | | Total |
|  |  | Strongly disgree | Disagree | Agree | Strongly Agree |  |
| Classification level of facility | Level IV | 5 | 23 | 59 | 77 | 164 |
|  | Level V | 3 | 7 | 9 | 12 | 31 |
|  | Level VI | 0 | 2 | 3 | 3 | 8 |
| Total | | 8 | 32 | 71 | 92 | 203 |

| **Chi-Square Tests** | | | |
| --- | --- | --- | --- |
|  | Value | df | Asymptotic Significance (2-sided) |
| Pearson Chi-Square | 5.774^a^ | 6 | .449 |
| Likelihood Ratio | 5.240 | 6 | .513 |
| Linear-by-Linear Association | 2.234 | 1 | .135 |
| N of Valid Cases | 203 |  |  |
| a. 6 cells (50.0%) have expected count less than 5. The minimum expected count is .32. | | | |

**Category of hospital classification * M-health will reduce the cost of health care to patients**

| **Crosstab** | | | | | | |
| --- | --- | --- | --- | --- | --- | --- |
| Count | | | | | | |
|  | | M-health will reduce the cost of health care to patients | | | | Total |
|  |  | Strongly disgree | Disagree | Agree | Strongly Agree |  |
| Category of hospital classification | Public Hospital | 4 | 14 | 36 | 47 | 101 |
|  | Private Hospital | 3 | 13 | 26 | 29 | 71 |
|  | Faith-Based Hospital/NGO | 1 | 5 | 9 | 16 | 31 |
| Total | | 8 | 32 | 71 | 92 | 203 |

| **Chi-Square Tests** | | | |
| --- | --- | --- | --- |
|  | Value | df | Asymptotic Significance (2-sided) |
| Pearson Chi-Square | 1.580^a^ | 6 | .954 |
| Likelihood Ratio | 1.595 | 6 | .953 |
| Linear-by-Linear Association | .009 | 1 | .925 |
| N of Valid Cases | 203 |  |  |
| a. 4 cells (33.3%) have expected count less than 5. The minimum expected count is 1.22. | | | |

**Geographical category of facility classification * M-health will reduce the cost of health care to patients**

| **Crosstab** | | | | | | |
| --- | --- | --- | --- | --- | --- | --- |
| Count | | | | | | |
|  | | M-health will reduce the cost of health care to patients | | | | Total |
|  |  | Strongly disgree | Disagree | Agree | Strongly Agree |  |
| Geographical category of facility classification | Urban | 4 | 12 | 26 | 30 | 72 |
|  | Semi-urban | 3 | 15 | 25 | 34 | 77 |
|  | Rural | 1 | 5 | 20 | 28 | 54 |
| Total | | 8 | 32 | 71 | 92 | 203 |

| **Chi-Square Tests** | | | |
| --- | --- | --- | --- |
|  | Value | df | Asymptotic Significance (2-sided) |
| Pearson Chi-Square | 4.210^a^ | 6 | .648 |
| Likelihood Ratio | 4.495 | 6 | .610 |
| N of Valid Cases | 203 |  |  |
| a. 3 cells (25.0%) have expected count less than 5. The minimum expected count is 2.13. | | | |

**Current annual number of patients (outpatients and in-patients) * M-health will reduce the cost of health care to patients**

| **Crosstab** | | | | | | |
| --- | --- | --- | --- | --- | --- | --- |
| Count | | | | | | |
|  | | M-health will reduce the cost of health care to patients | | | | Total |
|  |  | Strongly disgree | Disagree | Agree | Strongly Agree |  |
| Current annual number of patients (outpatients and in-patients) | Less than 200 | 1 | 8 | 19 | 18 | 46 |
|  | 200-500 | 2 | 13 | 12 | 27 | 54 |
|  | 501-1000 | 0 | 2 | 11 | 11 | 24 |
|  | 1001-1500 | 2 | 0 | 4 | 4 | 10 |
|  | 1501-2000 | 1 | 2 | 9 | 3 | 15 |
|  | 2001 and above | 2 | 7 | 13 | 28 | 50 |
| Total | | 8 | 32 | 68 | 91 | 199 |

| **Chi-Square Tests** | | | |
| --- | --- | --- | --- |
|  | Value | df | Asymptotic Significance (2-sided) |
| Pearson Chi-Square | 24.837^a^ | 15 | .052 |
| Likelihood Ratio | 24.483 | 15 | .057 |
| Linear-by-Linear Association | .331 | 1 | .565 |
| N of Valid Cases | 199 |  |  |
| a. 11 cells (45.8%) have expected count less than 5. The minimum expected count is .40. | | | |

**Number of staff * M-health will reduce the cost of health care to patients**

| **Crosstab** | | | | | | |
| --- | --- | --- | --- | --- | --- | --- |
| Count | | | | | | |
|  | | M-health will reduce the cost of health care to patients | | | | Total |
|  |  | Strongly disgree | Disagree | Agree | Strongly Agree |  |
| Number of staff | Less than 100 | 5 | 18 | 49 | 49 | 121 |
|  | 100-200 | 2 | 8 | 11 | 26 | 47 |
|  | 201-300 | 0 | 2 | 3 | 8 | 13 |
|  | 301-500 | 0 | 1 | 4 | 7 | 12 |
|  | 501 and above | 1 | 3 | 4 | 2 | 10 |
| Total | | 8 | 32 | 71 | 92 | 203 |

| **Chi-Square Tests** | | | |
| --- | --- | --- | --- |
|  | Value | df | Asymptotic Significance (2-sided) |
| Pearson Chi-Square | 11.492^a^ | 12 | .487 |
| Likelihood Ratio | 12.486 | 12 | .407 |
| Linear-by-Linear Association | .000 | 1 | .983 |
| N of Valid Cases | 203 |  |  |
| a. 12 cells (60.0%) have expected count less than 5. The minimum expected count is .39. | | | |

**County * M-health will reduce the cost of health care to patients**

| **Crosstab** | | | | | | |
| --- | --- | --- | --- | --- | --- | --- |
| Count | | | | | | |
|  | | M-health will reduce the cost of health care to patients | | | | Total |
|  |  | Strongly disgree | Disagree | Agree | Strongly Agree |  |
| County | No | 3 | 6 | 10 | 12 | 31 |
|  | Yes | 5 | 26 | 61 | 80 | 172 |
| Total | | 8 | 32 | 71 | 92 | 203 |

| **Chi-Square Tests** | | | |
| --- | --- | --- | --- |
|  | Value | df | Asymptotic Significance (2-sided) |
| Pearson Chi-Square | 3.785^a^ | 3 | .286 |
| Likelihood Ratio | 3.077 | 3 | .380 |
| N of Valid Cases | 203 |  |  |
| a. 2 cells (25.0%) have expected count less than 5. The minimum expected count is 1.22. | | | |

**Country * M-health will reduce the cost of health care to patients**

| **Crosstab** | | | | | | |
| --- | --- | --- | --- | --- | --- | --- |
| Count | | | | | | |
|  | | M-health will reduce the cost of health care to patients | | | | Total |
|  |  | Strongly disgree | Disagree | Agree | Strongly Agree |  |
| Country | No | 3 | 12 | 20 | 25 | 60 |
|  | Yes | 5 | 20 | 51 | 66 | 142 |
|  | 2 | 0 | 0 | 0 | 1 | 1 |
| Total | | 8 | 32 | 71 | 92 | 203 |

| **Chi-Square Tests** | | | |
| --- | --- | --- | --- |
|  | Value | df | Asymptotic Significance (2-sided) |
| Pearson Chi-Square | 2.679^a^ | 6 | .848 |
| Likelihood Ratio | 3.004 | 6 | .808 |
| N of Valid Cases | 203 |  |  |
| a. 5 cells (41.7%) have expected count less than 5. The minimum expected count is .04. | | | |

**Eastern Africa * M-health will reduce the cost of health care to patients**

| **Crosstab** | | | | | | |
| --- | --- | --- | --- | --- | --- | --- |
| Count | | | | | | |
|  | | M-health will reduce the cost of health care to patients | | | | Total |
|  |  | Strongly disgree | Disagree | Agree | Strongly Agree |  |
| Eastern Africa | No | 5 | 21 | 42 | 55 | 123 |
|  | Yes | 3 | 11 | 28 | 36 | 78 |
|  | 2 | 0 | 0 | 1 | 1 | 2 |
| Total | | 8 | 32 | 71 | 92 | 203 |

| **Chi-Square Tests** | | | |
| --- | --- | --- | --- |
|  | Value | df | Asymptotic Significance (2-sided) |
| Pearson Chi-Square | .875^a^ | 6 | .990 |
| Likelihood Ratio | 1.255 | 6 | .974 |
| N of Valid Cases | 203 |  |  |
| a. 6 cells (50.0%) have expected count less than 5. The minimum expected count is .08. | | | |

Compatibility

| **M-Health is compatible with current hospital’s health information system and consistent with its values and needs. * Category of hospital classification Crosstabulation** | | | | | |
| --- | --- | --- | --- | --- | --- |
| Count | | | | | |
|  | | Category of hospital classification | | | Total |
|  |  | Public Hospital | Private Hospital | Faith-Based Hospital/NGO |  |
| M-Health is compatible with current hospital’s health information system and consistent with its values and needs. | Strongly disagree | 12 | 3 | 3 | 18 |
|  | Disagree | 21 | 15 | 3 | 39 |
|  | Agree | 42 | 31 | 12 | 85 |
|  | Strongly Agree | 24 | 26 | 14 | 64 |
| Total | | 99 | 75 | 32 | 206 |

| **Crosstab** | | | | | | |
| --- | --- | --- | --- | --- | --- | --- |
| Count | | | | | | |
|  | | M-Health is compatible with current hospital’s health information system and consistent with its values and needs. | | | | Total |
|  |  | Strongly disgree | Disagree | Agree | Strongly Agree |  |
| Classification level of facility | Level IV | 14 | 28 | 71 | 52 | 165 |
|  | Level V | 3 | 9 | 11 | 10 | 33 |
|  | Level VI | 1 | 2 | 3 | 2 | 8 |
| Total | | 18 | 39 | 85 | 64 | 206 |

| **Chi-Square Tests** | | | |
| --- | --- | --- | --- |
|  | Value | df | Asymptotic Significance (2-sided) |
| Pearson Chi-Square | 2.617^a^ | 6 | .855 |
| Likelihood Ratio | 2.494 | 6 | .869 |
| Linear-by-Linear Association | .898 | 1 | .343 |
| N of Valid Cases | 206 |  |  |
| a. 5 cells (41.7%) have expected count less than 5. The minimum expected count is .70. | | | |

**Category of hospital classification * M-Health is compatible with current hospital’s health information system and consistent with its values and needs.**

| **Crosstab** | | | | | | |
| --- | --- | --- | --- | --- | --- | --- |
| Count | | | | | | |
|  | | M-Health is compatible with current hospital’s health information system and consistent with its values and needs. | | | | Total |
|  |  | Strongly disgree | Disagree | Agree | Strongly Agree |  |
| Category of hospital classification | Public Hospital | 12 | 21 | 42 | 24 | 99 |
|  | Private Hospital | 3 | 15 | 31 | 26 | 75 |
|  | Faith-Based Hospital/NGO | 3 | 3 | 12 | 14 | 32 |
| Total | | 18 | 39 | 85 | 64 | 206 |

| **Chi-Square Tests** | | | |
| --- | --- | --- | --- |
|  | Value | df | Asymptotic Significance (2-sided) |
| Pearson Chi-Square | 8.695^a^ | 6 | .191 |
| Likelihood Ratio | 9.317 | 6 | .157 |
| Linear-by-Linear Association | 5.411 | 1 | .020 |
| N of Valid Cases | 206 |  |  |
| a. 1 cells (8.3%) have expected count less than 5. The minimum expected count is 2.80. | | | |

**Geographical category of facility classification * M-Health is compatible with current hospital’s health information system and consistent with its values and needs.**

| **Crosstab** | | | | | | |
| --- | --- | --- | --- | --- | --- | --- |
| Count | | | | | | |
|  | | M-Health is compatible with current hospital’s health information system and consistent with its values and needs. | | | | Total |
|  |  | Strongly disgree | Disagree | Agree | Strongly Agree |  |
| Geographical category of facility classification | Urban | 4 | 14 | 31 | 25 | 74 |
|  | Semi-urban | 6 | 13 | 34 | 26 | 79 |
|  | Rural | 8 | 12 | 20 | 13 | 53 |
| Total | | 18 | 39 | 85 | 64 | 206 |

| **Chi-Square Tests** | | | |
| --- | --- | --- | --- |
|  | Value | df | Asymptotic Significance (2-sided) |
| Pearson Chi-Square | 5.369^a^ | 6 | .497 |
| Likelihood Ratio | 5.126 | 6 | .528 |
| N of Valid Cases | 206 |  |  |
| a. 1 cells (8.3%) have expected count less than 5. The minimum expected count is 4.63. | | | |

**Current annual number of patients (outpatients and in-patients) * M-Health is compatible with current hospital’s health information system and consistent with its values and needs.**

| **Crosstab** | | | | | | |
| --- | --- | --- | --- | --- | --- | --- |
| Count | | | | | | |
|  | | M-Health is compatible with current hospital’s health information system and consistent with its values and needs. | | | | Total |
|  |  | Strongly disgree | Disagree | Agree | Strongly Agree |  |
| Current annual number of patients (outpatients and in-patients) | Less than 200 | 3 | 6 | 25 | 15 | 49 |
|  | 200-500 | 6 | 10 | 17 | 20 | 53 |
|  | 501-1000 | 3 | 5 | 8 | 8 | 24 |
|  | 1001-1500 | 1 | 3 | 6 | 3 | 13 |
|  | 1501-2000 | 1 | 2 | 6 | 5 | 14 |
|  | 2001 and above | 4 | 12 | 20 | 13 | 49 |
| Total | | 18 | 38 | 82 | 64 | 202 |

| **Chi-Square Tests** | | | |
| --- | --- | --- | --- |
|  | Value | df | Asymptotic Significance (2-sided) |
| Pearson Chi-Square | 7.641^a^ | 15 | .937 |
| Likelihood Ratio | 7.731 | 15 | .934 |
| Linear-by-Linear Association | .827 | 1 | .363 |
| N of Valid Cases | 202 |  |  |
| a. 11 cells (45.8%) have expected count less than 5. The minimum expected count is 1.16. | | | |

**Number of staff * M-Health is compatible with current hospital’s health information system and consistent with its values and needs.**

| **Crosstab** | | | | | | |
| --- | --- | --- | --- | --- | --- | --- |
| Count | | | | | | |
|  | | M-Health is compatible with current hospital’s health information system and consistent with its values and needs. | | | | Total |
|  |  | Strongly disgree | Disagree | Agree | Strongly Agree |  |
| Number of staff | Less than 100 | 12 | 19 | 55 | 36 | 122 |
|  | 100-200 | 3 | 16 | 16 | 16 | 51 |
|  | 201-300 | 0 | 0 | 4 | 8 | 12 |
|  | 301-500 | 2 | 0 | 5 | 4 | 11 |
|  | 501 and above | 1 | 4 | 5 | 0 | 10 |
| Total | | 18 | 39 | 85 | 64 | 206 |

| **Chi-Square Tests** | | | |
| --- | --- | --- | --- |
|  | Value | df | Asymptotic Significance (2-sided) |
| Pearson Chi-Square | 24.682^a^ | 12 | .016 |
| Likelihood Ratio | 30.626 | 12 | .002 |
| Linear-by-Linear Association | .174 | 1 | .677 |
| N of Valid Cases | 206 |  |  |
| a. 13 cells (65.0%) have expected count less than 5. The minimum expected count is .87. | | | |

**County * M-Health is compatible with current hospital’s health information system and consistent with its values and needs.**

| **Crosstab** | | | | | | |
| --- | --- | --- | --- | --- | --- | --- |
| Count | | | | | | |
|  | | M-Health is compatible with current hospital’s health information system and consistent with its values and needs. | | | | Total |
|  |  | Strongly disgree | Disagree | Agree | Strongly Agree |  |
| County | No | 2 | 7 | 16 | 9 | 34 |
|  | Yes | 16 | 32 | 69 | 55 | 172 |
| Total | | 18 | 39 | 85 | 64 | 206 |

| **Chi-Square Tests** | | | |
| --- | --- | --- | --- |
|  | Value | df | Asymptotic Significance (2-sided) |
| Pearson Chi-Square | 1.048^a^ | 3 | .790 |
| Likelihood Ratio | 1.087 | 3 | .780 |
| N of Valid Cases | 206 |  |  |
| a. 1 cells (12.5%) have expected count less than 5. The minimum expected count is 2.97. | | | |

**Country * M-Health is compatible with current hospital’s health information system and consistent with its values and needs.**

| **Crosstab** | | | | | | |
| --- | --- | --- | --- | --- | --- | --- |
| Count | | | | | | |
|  | | M-Health is compatible with current hospital’s health information system and consistent with its values and needs. | | | | Total |
|  |  | Strongly disgree | Disagree | Agree | Strongly Agree |  |
| Country | No | 7 | 17 | 21 | 12 | 57 |
|  | Yes | 11 | 22 | 64 | 51 | 148 |
|  | 2 | 0 | 0 | 0 | 1 | 1 |
| Total | | 18 | 39 | 85 | 64 | 206 |

| **Chi-Square Tests** | | | |
| --- | --- | --- | --- |
|  | Value | df | Asymptotic Significance (2-sided) |
| Pearson Chi-Square | 10.990^a^ | 6 | .089 |
| Likelihood Ratio | 10.826 | 6 | .094 |
| N of Valid Cases | 206 |  |  |
| a. 5 cells (41.7%) have expected count less than 5. The minimum expected count is .09. | | | |

**Eastern Africa * M-Health is compatible with current hospital’s health information system and consistent with its values and needs.**

| **Crosstab** | | | | | | |
| --- | --- | --- | --- | --- | --- | --- |
| Count | | | | | | |
|  | | M-Health is compatible with current hospital’s health information system and consistent with its values and needs. | | | | Total |
|  |  | Strongly disgree | Disagree | Agree | Strongly Agree |  |
| Eastern Africa | No | 13 | 27 | 54 | 32 | 126 |
|  | Yes | 5 | 12 | 31 | 31 | 79 |
|  |  |  |  |  |  |  |
| Total | | 18 | 39 | 85 | 64 | 206 |

| **Chi-Square Tests** | | | |
| --- | --- | --- | --- |
|  | Value | df | Asymptotic Significance (2-sided) |
| Pearson Chi-Square | 11.103^a^ | 6 | .085 |
| Likelihood Ratio | 10.348 | 6 | .111 |
| N of Valid Cases | 206 |  |  |
| a. 4 cells (33.3%) have expected count less than 5. The minimum expected count is .17. | | | |

| **M-Health can be trusted in terms of its security and confidentiality of patient information * Category of hospital classification Crosstabulation** | | | | | |
| --- | --- | --- | --- | --- | --- |
| Count | | | | | |
|  | | Category of hospital classification | | | Total |
|  |  | Public Hospital | Private Hospital | Faith-Based Hospital/NGO |  |
| M-Health can be trusted in terms of its security and confidentiality of patient information | Strongly disagree | 8 | 4 | 1 | 13 |
|  | Disagree | 16 | 17 | 7 | 40 |
|  | Agree | 51 | 37 | 16 | 104 |
|  | Strongly Agree | 26 | 18 | 8 | 52 |
| Total | | 101 | 76 | 32 | 209 |

| **Crosstab** | | | | | | |
| --- | --- | --- | --- | --- | --- | --- |
| Count | | | | | | |
|  | | M-Health can be trusted in terms of its security and confidentiality of patient information | | | | Total |
|  |  | Strongly disgree | Disagree | Agree | Strongly Agree |  |
| Classification level of facility | Level IV | 9 | 29 | 88 | 42 | 168 |
|  | Level V | 2 | 11 | 12 | 8 | 33 |
|  | Level VI | 2 | 0 | 4 | 2 | 8 |
| Total | | 13 | 40 | 104 | 52 | 209 |

| **Chi-Square Tests** | | | |
| --- | --- | --- | --- |
|  | Value | df | Asymptotic Significance (2-sided) |
| Pearson Chi-Square | 11.482^a^ | 6 | .075 |
| Likelihood Ratio | 10.541 | 6 | .104 |
| Linear-by-Linear Association | 1.618 | 1 | .203 |
| N of Valid Cases | 209 |  |  |
| a. 5 cells (41.7%) have expected count less than 5. The minimum expected count is .50. | | | |

**Category of hospital classification * M-Health can be trusted in terms of its security and confidentiality of patient information**

| **Crosstab** | | | | | | |
| --- | --- | --- | --- | --- | --- | --- |
| Count | | | | | | |
|  | | M-Health can be trusted in terms of its security and confidentiality of patient information | | | | Total |
|  |  | Strongly disgree | Disagree | Agree | Strongly Agree |  |
| Category of hospital classification | Public Hospital | 8 | 16 | 51 | 26 | 101 |
|  | Private Hospital | 4 | 17 | 37 | 18 | 76 |
|  | Faith-Based Hospital/NGO | 1 | 7 | 16 | 8 | 32 |
| Total | | 13 | 40 | 104 | 52 | 209 |

| **Chi-Square Tests** | | | |
| --- | --- | --- | --- |
|  | Value | df | Asymptotic Significance (2-sided) |
| Pearson Chi-Square | 2.291^a^ | 6 | .891 |
| Likelihood Ratio | 2.380 | 6 | .882 |
| Linear-by-Linear Association | .002 | 1 | .965 |
| N of Valid Cases | 209 |  |  |
| a. 2 cells (16.7%) have expected count less than 5. The minimum expected count is 1.99. | | | |

**Geographical category of facility classification * M-Health can be trusted in terms of its security and confidentiality of patient information**

| **Crosstab** | | | | | | |
| --- | --- | --- | --- | --- | --- | --- |
| Count | | | | | | |
|  | | M-Health can be trusted in terms of its security and confidentiality of patient information | | | | Total |
|  |  | Strongly disgree | Disagree | Agree | Strongly Agree |  |
| Geographical category of facility classification | Urban | 4 | 20 | 35 | 18 | 77 |
|  | Semi-urban | 6 | 12 | 40 | 21 | 79 |
|  | Rural | 3 | 8 | 29 | 13 | 53 |
| Total | | 13 | 40 | 104 | 52 | 209 |

| **Chi-Square Tests** | | | |
| --- | --- | --- | --- |
|  | Value | df | Asymptotic Significance (2-sided) |
| Pearson Chi-Square | 4.098^a^ | 6 | .663 |
| Likelihood Ratio | 3.985 | 6 | .679 |
| N of Valid Cases | 209 |  |  |
| a. 3 cells (25.0%) have expected count less than 5. The minimum expected count is 3.30. | | | |

**Current annual number of patients (outpatients and in-patients) * M-Health can be trusted in terms of its security and confidentiality of patient information**

| **Crosstab** | | | | | | |
| --- | --- | --- | --- | --- | --- | --- |
| Count | | | | | | |
|  | | M-Health can be trusted in terms of its security and confidentiality of patient information | | | | Total |
|  |  | Strongly disgree | Disagree | Agree | Strongly Agree |  |
| Current annual number of patients (outpatients and in-patients) | Less than 200 | 1 | 6 | 25 | 16 | 48 |
|  | 200-500 | 6 | 14 | 25 | 11 | 56 |
|  | 501-1000 | 1 | 5 | 9 | 9 | 24 |
|  | 1001-1500 | 1 | 2 | 8 | 2 | 13 |
|  | 1501-2000 | 1 | 2 | 8 | 3 | 14 |
|  | 2001 and above | 3 | 11 | 26 | 10 | 50 |
| Total | | 13 | 40 | 101 | 51 | 205 |

| **Chi-Square Tests** | | | |
| --- | --- | --- | --- |
|  | Value | df | Asymptotic Significance (2-sided) |
| Pearson Chi-Square | 12.058^a^ | 15 | .675 |
| Likelihood Ratio | 12.253 | 15 | .660 |
| Linear-by-Linear Association | 1.014 | 1 | .314 |
| N of Valid Cases | 205 |  |  |
| a. 11 cells (45.8%) have expected count less than 5. The minimum expected count is .82. | | | |

**Number of staff * M-Health can be trusted in terms of its security and confidentiality of patient information**

| **Crosstab** | | | | | | |
| --- | --- | --- | --- | --- | --- | --- |
| Count | | | | | | |
|  | | M-Health can be trusted in terms of its security and confidentiality of patient information | | | | Total |
|  |  | Strongly disgree | Disagree | Agree | Strongly Agree |  |
| Number of staff | Less than 100 | 7 | 19 | 67 | 30 | 123 |
|  | 100-200 | 3 | 15 | 23 | 10 | 51 |
|  | 201-300 | 0 | 2 | 3 | 8 | 13 |
|  | 301-500 | 1 | 0 | 7 | 4 | 12 |
|  | 501 and above | 2 | 4 | 4 | 0 | 10 |
| Total | | 13 | 40 | 104 | 52 | 209 |

| **Chi-Square Tests** | | | |
| --- | --- | --- | --- |
|  | Value | df | Asymptotic Significance (2-sided) |
| Pearson Chi-Square | 25.803^a^ | 12 | .011 |
| Likelihood Ratio | 27.784 | 12 | .006 |
| Linear-by-Linear Association | 1.222 | 1 | .269 |
| N of Valid Cases | 209 |  |  |
| a. 11 cells (55.0%) have expected count less than 5. The minimum expected count is .62. | | | |

**County * M-Health can be trusted in terms of its security and confidentiality of patient information**

| **Crosstab** | | | | | | |
| --- | --- | --- | --- | --- | --- | --- |
| Count | | | | | | |
|  | | M-Health can be trusted in terms of its security and confidentiality of patient information | | | | Total |
|  |  | Strongly disgree | Disagree | Agree | Strongly Agree |  |
| County | No | 5 | 8 | 15 | 8 | 36 |
|  | Yes | 8 | 32 | 89 | 44 | 173 |
| Total | | 13 | 40 | 104 | 52 | 209 |

| **Chi-Square Tests** | | | |
| --- | --- | --- | --- |
|  | Value | df | Asymptotic Significance (2-sided) |
| Pearson Chi-Square | 5.024^a^ | 3 | .170 |
| Likelihood Ratio | 4.224 | 3 | .238 |
| N of Valid Cases | 209 |  |  |
| a. 1 cells (12.5%) have expected count less than 5. The minimum expected count is 2.24. | | | |

**Country * M-Health can be trusted in terms of its security and confidentiality of patient information**

| **Crosstab** | | | | | | |
| --- | --- | --- | --- | --- | --- | --- |
| Count | | | | | | |
|  | | M-Health can be trusted in terms of its security and confidentiality of patient information | | | | Total |
|  |  | Strongly disgree | Disagree | Agree | Strongly Agree |  |
| Country | No | 8 | 11 | 29 | 12 | 60 |
|  | Yes | 5 | 29 | 75 | 40 | 149 |
|  |  |  |  |  |  |  |
| Total | | 13 | 40 | 104 | 52 | 209 |

| **Chi-Square Tests** | | | |
| --- | --- | --- | --- |
|  | Value | df | Asymptotic Significance (2-sided) |
| Pearson Chi-Square | 8.723^a^ | 6 | .190 |
| Likelihood Ratio | 8.313 | 6 | .216 |
| N of Valid Cases | 209 |  |  |
| a. 5 cells (41.7%) have expected count less than 5. The minimum expected count is .06. | | | |

**Eastern Africa * M-Health can be trusted in terms of its security and confidentiality of patient information**

| **Crosstab** | | | | | | |
| --- | --- | --- | --- | --- | --- | --- |
| Count | | | | | | |
|  | | M-Health can be trusted in terms of its security and confidentiality of patient information | | | | Total |
|  |  | Strongly disgree | Disagree | Agree | Strongly Agree |  |
| Eastern Africa | No | 11 | 30 | 57 | 30 | 128 |
|  | Yes | 2 | 10 | 47 | 22 | 79 |
|  |  |  |  |  |  |  |
| Total | | 13 | 40 | 104 | 52 | 209 |

| **Chi-Square Tests** | | | |
| --- | --- | --- | --- |
|  | Value | df | Asymptotic Significance (2-sided) |
| Pearson Chi-Square | 9.790^a^ | 6 | .134 |
| Likelihood Ratio | 11.077 | 6 | .086 |
| N of Valid Cases | 209 |  |  |
| a. 5 cells (41.7%) have expected count less than 5. The minimum expected count is .12. | | | |

| **M-Health will make many key staff redundant * Category of hospital classification Crosstabulation** | | | | | |
| --- | --- | --- | --- | --- | --- |
| Count | | | | | |
|  | | Category of hospital classification | | | Total |
|  |  | Public Hospital | Private Hospital | Faith-Based Hospital/NGO |  |
| M-Health will make many key staff redundant | Strongly disagree | 28 | 21 | 7 | 56 |
|  | Disagree | 46 | 34 | 6 | 86 |
|  | Agree | 20 | 14 | 12 | 46 |
|  | Strongly Agree | 7 | 7 | 7 | 21 |
| Total | | 101 | 76 | 32 | 209 |

| **Crosstab** | | | | | | |
| --- | --- | --- | --- | --- | --- | --- |
| Count | | | | | | |
|  | | M-Health will make many key staff redundant | | | | Total |
|  |  | Strongly disgree | Disagree | Agree | Strongly Agree |  |
| Classification level of facility | Level IV | 43 | 72 | 38 | 15 | 168 |
|  | Level V | 8 | 13 | 7 | 5 | 33 |
|  | Level VI | 5 | 1 | 1 | 1 | 8 |
| Total | | 56 | 86 | 46 | 21 | 209 |

| **Chi-Square Tests** | | | |
| --- | --- | --- | --- |
|  | Value | df | Asymptotic Significance (2-sided) |
| Pearson Chi-Square | 7.196^a^ | 6 | .303 |
| Likelihood Ratio | 6.704 | 6 | .349 |
| Linear-by-Linear Association | .134 | 1 | .714 |
| N of Valid Cases | 209 |  |  |
| a. 5 cells (41.7%) have expected count less than 5. The minimum expected count is .80. | | | |

**Category of hospital classification * M-Health will make many key staff redundant**

| **Crosstab** | | | | | | |
| --- | --- | --- | --- | --- | --- | --- |
| Count | | | | | | |
|  | | M-Health will make many key staff redundant | | | | Total |
|  |  | Strongly disgree | Disagree | Agree | Strongly Agree |  |
| Category of hospital classification | Public Hospital | 28 | 46 | 20 | 7 | 101 |
|  | Private Hospital | 21 | 34 | 14 | 7 | 76 |
|  | Faith-Based Hospital/NGO | 7 | 6 | 12 | 7 | 32 |
| Total | | 56 | 86 | 46 | 21 | 209 |

| **Chi-Square Tests** | | | |
| --- | --- | --- | --- |
|  | Value | df | Asymptotic Significance (2-sided) |
| Pearson Chi-Square | 14.597^a^ | 6 | .024 |
| Likelihood Ratio | 14.004 | 6 | .030 |
| Linear-by-Linear Association | 5.786 | 1 | .016 |
| N of Valid Cases | 209 |  |  |
| a. 1 cells (8.3%) have expected count less than 5. The minimum expected count is 3.22. | | | |

**Geographical category of facility classification * M-Health will make many key staff redundant**

| **Crosstab** | | | | | | |
| --- | --- | --- | --- | --- | --- | --- |
| Count | | | | | | |
|  | | M-Health will make many key staff redundant | | | | Total |
|  |  | Strongly disgree | Disagree | Agree | Strongly Agree |  |
| Geographical category of facility classification | Urban | 18 | 31 | 19 | 8 | 76 |
|  | Semi-urban | 28 | 30 | 14 | 7 | 79 |
|  | Rural | 10 | 25 | 13 | 6 | 54 |
| Total | | 56 | 86 | 46 | 21 | 209 |

| **Chi-Square Tests** | | | |
| --- | --- | --- | --- |
|  | Value | df | Asymptotic Significance (2-sided) |
| Pearson Chi-Square | 5.666^a^ | 6 | .462 |
| Likelihood Ratio | 5.666 | 6 | .462 |
| N of Valid Cases | 209 |  |  |
| a. 0 cells (0.0%) have expected count less than 5. The minimum expected count is 5.43. | | | |

**Current annual number of patients (outpatients and in-patients) * M-Health will make many key staff redundant**

| **Crosstab** | | | | | | |
| --- | --- | --- | --- | --- | --- | --- |
| Count | | | | | | |
|  | | M-Health will make many key staff redundant | | | | Total |
|  |  | Strongly disgree | Disagree | Agree | Strongly Agree |  |
| Current annual number of patients (outpatients and in-patients) | Less than 200 | 11 | 16 | 16 | 6 | 49 |
|  | 200-500 | 11 | 19 | 14 | 10 | 54 |
|  | 501-1000 | 5 | 15 | 3 | 1 | 24 |
|  | 1001-1500 | 3 | 6 | 2 | 2 | 13 |
|  | 1501-2000 | 5 | 8 | 2 | 0 | 15 |
|  | 2001 and above | 19 | 21 | 8 | 2 | 50 |
| Total | | 54 | 85 | 45 | 21 | 205 |

| **Chi-Square Tests** | | | |
| --- | --- | --- | --- |
|  | Value | df | Asymptotic Significance (2-sided) |
| Pearson Chi-Square | 22.707^a^ | 15 | .091 |
| Likelihood Ratio | 23.810 | 15 | .068 |
| Linear-by-Linear Association | 11.519 | 1 | .001 |
| N of Valid Cases | 205 |  |  |
| a. 7 cells (29.2%) have expected count less than 5. The minimum expected count is 1.33. | | | |

**Number of staff * M-Health will make many key staff redundant**

| **Crosstab** | | | | | | |
| --- | --- | --- | --- | --- | --- | --- |
| Count | | | | | | |
|  | | M-Health will make many key staff redundant | | | | Total |
|  |  | Strongly disgree | Disagree | Agree | Strongly Agree |  |
| Number of staff | Less than 100 | 28 | 52 | 31 | 13 | 124 |
|  | 100-200 | 16 | 22 | 7 | 6 | 51 |
|  | 201-300 | 3 | 6 | 3 | 0 | 12 |
|  | 301-500 | 3 | 5 | 2 | 2 | 12 |
|  | 501 and above | 6 | 1 | 3 | 0 | 10 |
| Total | | 56 | 86 | 46 | 21 | 209 |

| **Chi-Square Tests** | | | |
| --- | --- | --- | --- |
|  | Value | df | Asymptotic Significance (2-sided) |
| Pearson Chi-Square | 13.515^a^ | 12 | .333 |
| Likelihood Ratio | 15.810 | 12 | .200 |
| Linear-by-Linear Association | 2.380 | 1 | .123 |
| N of Valid Cases | 209 |  |  |
| a. 12 cells (60.0%) have expected count less than 5. The minimum expected count is 1.00. | | | |

**County * M-Health will make many key staff redundant**

| **Crosstab** | | | | | | |
| --- | --- | --- | --- | --- | --- | --- |
| Count | | | | | | |
|  | | M-Health will make many key staff redundant | | | | Total |
|  |  | Strongly disgree | Disagree | Agree | Strongly Agree |  |
| County | No | 6 | 16 | 9 | 4 | 35 |
|  | Yes | 50 | 70 | 37 | 17 | 174 |
| Total | | 56 | 86 | 46 | 21 | 209 |

| **Chi-Square Tests** | | | |
| --- | --- | --- | --- |
|  | Value | df | Asymptotic Significance (2-sided) |
| Pearson Chi-Square | 2.016^a^ | 3 | .569 |
| Likelihood Ratio | 2.171 | 3 | .538 |
| N of Valid Cases | 209 |  |  |
| a. 1 cells (12.5%) have expected count less than 5. The minimum expected count is 3.52. | | | |

**Country * M-Health will make many key staff redundant**

| **Crosstab** | | | | | | |
| --- | --- | --- | --- | --- | --- | --- |
| Count | | | | | | |
|  | | M-Health will make many key staff redundant | | | | Total |
|  |  | Strongly disgree | Disagree | Agree | Strongly Agree |  |
| Country | No | 15 | 25 | 12 | 8 | 60 |
|  | Yes | 41 | 61 | 33 | 13 | 148 |
|  | 2 | 0 | 0 | 1 | 0 | 1 |
| Total | | 56 | 86 | 46 | 21 | 209 |

| **Chi-Square Tests** | | | |
| --- | --- | --- | --- |
|  | Value | df | Asymptotic Significance (2-sided) |
| Pearson Chi-Square | 4.661^a^ | 6 | .588 |
| Likelihood Ratio | 4.099 | 6 | .663 |
| N of Valid Cases | 209 |  |  |
| a. 4 cells (33.3%) have expected count less than 5. The minimum expected count is .10. | | | |

**Eastern Africa * M-Health will make many key staff redundant**

| **Crosstab** | | | | | | |
| --- | --- | --- | --- | --- | --- | --- |
| Count | | | | | | |
|  | | M-Health will make many key staff redundant | | | | Total |
|  |  | Strongly disgree | Disagree | Agree | Strongly Agree |  |
| Eastern Africa | No | 30 | 59 | 24 | 17 | 130 |
|  | Yes | 26 | 27 | 22 | 4 | 79 |
| Total | | 56 | 86 | 46 | 21 | 209 |

| **Chi-Square Tests** | | | |
| --- | --- | --- | --- |
|  | Value | df | Asymptotic Significance (2-sided) |
| Pearson Chi-Square | 8.381^a^ | 3 | .039 |
| Likelihood Ratio | 8.661 | 3 | .034 |
| N of Valid Cases | 209 |  |  |
| a. 0 cells (0.0%) have expected count less than 5. The minimum expected count is 7.94. | | | |

Complexity

| **M-Health is difficult to understand, use and integrate in the hospital systems and operations * Category of hospital classification Crosstabulation** | | | | | |
| --- | --- | --- | --- | --- | --- |
| Count | | | | | |
|  | | Category of hospital classification | | | Total |
|  |  | Public Hospital | Private Hospital | Faith-Based Hospital/NGO |  |
| M-Health is difficult to understand, use and integrate in the hospital systems and operations | Strongly disagree | 28 | 23 | 12 | 63 |
|  | Disagree | 50 | 30 | 14 | 94 |
|  | Agree | 21 | 16 | 4 | 41 |
|  | Strongly Agree | 3 | 5 | 1 | 9 |
| Total | | 102 | 74 | 31 | 207 |

| **Crosstab** | | | | | | |
| --- | --- | --- | --- | --- | --- | --- |
| Count | | | | | | |
|  | | M-Health is difficult to understand, use and integrate in the hospital systems and operations | | | | Total |
|  |  | Strongly disgree | Disagree | Agree | Strongly Agree |  |
| Classification level of facility | Level IV | 49 | 77 | 34 | 8 | 168 |
|  | Level V | 11 | 14 | 6 | 1 | 32 |
|  | Level VI | 3 | 3 | 1 | 0 | 7 |
| Total | | 63 | 94 | 41 | 9 | 207 |

| **Chi-Square Tests** | | | |
| --- | --- | --- | --- |
|  | Value | df | Asymptotic Significance (2-sided) |
| Pearson Chi-Square | 1.265^a^ | 6 | .974 |
| Likelihood Ratio | 1.546 | 6 | .956 |
| Linear-by-Linear Association | 1.105 | 1 | .293 |
| N of Valid Cases | 207 |  |  |
| a. 5 cells (41.7%) have expected count less than 5. The minimum expected count is .30. | | | |

**Category of hospital classification * M-Health is difficult to understand, use and integrate in the hospital systems and operations**

| **Crosstab** | | | | | | |
| --- | --- | --- | --- | --- | --- | --- |
| Count | | | | | | |
|  | | M-Health is difficult to understand, use and integrate in the hospital systems and operations | | | | Total |
|  |  | Strongly disgree | Disagree | Agree | Strongly Agree |  |
| Category of hospital classification | Public Hospital | 28 | 50 | 21 | 3 | 102 |
|  | Private Hospital | 23 | 30 | 16 | 5 | 74 |
|  | Faith-Based Hospital/NGO | 12 | 14 | 4 | 1 | 31 |
| Total | | 63 | 94 | 41 | 9 | 207 |

| **Chi-Square Tests** | | | |
| --- | --- | --- | --- |
|  | Value | df | Asymptotic Significance (2-sided) |
| Pearson Chi-Square | 4.128^a^ | 6 | .659 |
| Likelihood Ratio | 4.116 | 6 | .661 |
| Linear-by-Linear Association | .550 | 1 | .458 |
| N of Valid Cases | 207 |  |  |
| a. 3 cells (25.0%) have expected count less than 5. The minimum expected count is 1.35. | | | |

**Geographical category of facility classification * M-Health is difficult to understand, use and integrate in the hospital systems and operations**

| **Crosstab** | | | | | | |
| --- | --- | --- | --- | --- | --- | --- |
| Count | | | | | | |
|  | | M-Health is difficult to understand, use and integrate in the hospital systems and operations | | | | Total |
|  |  | Strongly disgree | Disagree | Agree | Strongly Agree |  |
| Geographical category of facility classification | Urban | 21 | 34 | 15 | 6 | 76 |
|  | Semi-urban | 24 | 34 | 17 | 3 | 78 |
|  | Rural | 18 | 26 | 9 | 0 | 53 |
| Total | | 63 | 94 | 41 | 9 | 207 |

| **Chi-Square Tests** | | | |
| --- | --- | --- | --- |
|  | Value | df | Asymptotic Significance (2-sided) |
| Pearson Chi-Square | 5.554^a^ | 6 | .475 |
| Likelihood Ratio | 7.429 | 6 | .283 |
| N of Valid Cases | 207 |  |  |
| a. 3 cells (25.0%) have expected count less than 5. The minimum expected count is 2.30. | | | |

**Current annual number of patients (outpatients and in-patients) * M-Health is difficult to understand, use and integrate in the hospital systems and operations**

| **Crosstab** | | | | | | |
| --- | --- | --- | --- | --- | --- | --- |
| Count | | | | | | |
|  | | M-Health is difficult to understand, use and integrate in the hospital systems and operations | | | | Total |
|  |  | Strongly disgree | Disagree | Agree | Strongly Agree |  |
| Current annual number of patients (outpatients and in-patients) | Less than 200 | 15 | 25 | 7 | 2 | 49 |
|  | 200-500 | 20 | 16 | 15 | 4 | 55 |
|  | 501-1000 | 5 | 14 | 5 | 0 | 24 |
|  | 1001-1500 | 0 | 5 | 5 | 1 | 11 |
|  | 1501-2000 | 5 | 7 | 3 | 0 | 15 |
|  | 2001 and above | 18 | 24 | 5 | 2 | 49 |
| Total | | 63 | 91 | 40 | 9 | 203 |

| **Chi-Square Tests** | | | |
| --- | --- | --- | --- |
|  | Value | df | Asymptotic Significance (2-sided) |
| Pearson Chi-Square | 21.484^a^ | 15 | .122 |
| Likelihood Ratio | 26.096 | 15 | .037 |
| Linear-by-Linear Association | .632 | 1 | .427 |
| N of Valid Cases | 203 |  |  |
| a. 12 cells (50.0%) have expected count less than 5. The minimum expected count is .49. | | | |

**Number of staff * M-Health is difficult to understand, use and integrate in the hospital systems and operations**

| **Crosstab** | | | | | | |
| --- | --- | --- | --- | --- | --- | --- |
| Count | | | | | | |
|  | | M-Health is difficult to understand, use and integrate in the hospital systems and operations | | | | Total |
|  |  | Strongly disgree | Disagree | Agree | Strongly Agree |  |
| Number of staff | Less than 100 | 35 | 59 | 24 | 5 | 123 |
|  | 100-200 | 18 | 18 | 12 | 2 | 50 |
|  | 201-300 | 5 | 4 | 3 | 1 | 13 |
|  | 301-500 | 2 | 8 | 2 | 0 | 12 |
|  | 501 and above | 3 | 5 | 0 | 1 | 9 |
| Total | | 63 | 94 | 41 | 9 | 207 |

| **Chi-Square Tests** | | | |
| --- | --- | --- | --- |
|  | Value | df | Asymptotic Significance (2-sided) |
| Pearson Chi-Square | 9.080^a^ | 12 | .696 |
| Likelihood Ratio | 11.093 | 12 | .521 |
| Linear-by-Linear Association | .058 | 1 | .809 |
| N of Valid Cases | 207 |  |  |
| a. 11 cells (55.0%) have expected count less than 5. The minimum expected count is .39. | | | |

**County * M-Health is difficult to understand, use and integrate in the hospital systems and operations**

| **Crosstab** | | | | | | |
| --- | --- | --- | --- | --- | --- | --- |
| Count | | | | | | |
|  | | M-Health is difficult to understand, use and integrate in the hospital systems and operations | | | | Total |
|  |  | Strongly disgree | Disagree | Agree | Strongly Agree |  |
| County | No | 8 | 17 | 6 | 4 | 35 |
|  | Yes | 55 | 77 | 35 | 5 | 172 |
| Total | | 63 | 94 | 41 | 9 | 207 |

| **Chi-Square Tests** | | | |
| --- | --- | --- | --- |
|  | Value | df | Asymptotic Significance (2-sided) |
| Pearson Chi-Square | 5.896^a^ | 3 | .117 |
| Likelihood Ratio | 4.809 | 3 | .186 |
| N of Valid Cases | 207 |  |  |
| a. 1 cells (12.5%) have expected count less than 5. The minimum expected count is 1.52. | | | |

**Country * M-Health is difficult to understand, use and integrate in the hospital systems and operations**

| **Crosstab** | | | | | | |
| --- | --- | --- | --- | --- | --- | --- |
| Count | | | | | | |
|  | | M-Health is difficult to understand, use and integrate in the hospital systems and operations | | | | Total |
|  |  | Strongly disgree | Disagree | Agree | Strongly Agree |  |
| Country | No | 15 | 29 | 14 | 2 | 60 |
|  | Yes | 47 | 65 | 27 | 7 | 146 |
|  | 2 | 1 | 0 | 0 | 0 | 1 |
| Total | | 63 | 94 | 41 | 9 | 207 |

| **Chi-Square Tests** | | | |
| --- | --- | --- | --- |
|  | Value | df | Asymptotic Significance (2-sided) |
| Pearson Chi-Square | 3.867^a^ | 6 | .695 |
| Likelihood Ratio | 3.987 | 6 | .678 |
| N of Valid Cases | 207 |  |  |
| a. 5 cells (41.7%) have expected count less than 5. The minimum expected count is .04. | | | |

**Eastern Africa * M-Health is difficult to understand, use and integrate in the hospital systems and operations**

| **Crosstab** | | | | | | |
| --- | --- | --- | --- | --- | --- | --- |
| Count | | | | | | |
|  | | M-Health is difficult to understand, use and integrate in the hospital systems and operations | | | | Total |
|  |  | Strongly disgree | Disagree | Agree | Strongly Agree |  |
| Eastern Africa | No | 34 | 61 | 28 | 6 | 129 |
|  | Yes | 29 | 33 | 13 | 3 | 78 |
| Total | | 63 | 94 | 41 | 9 | 207 |

| **Chi-Square Tests** | | | |
| --- | --- | --- | --- |
|  | Value | df | Asymptotic Significance (2-sided) |
| Pearson Chi-Square | 2.832^a^ | 3 | .418 |
| Likelihood Ratio | 2.807 | 3 | .422 |
| N of Valid Cases | 207 |  |  |
| a. 1 cells (12.5%) have expected count less than 5. The minimum expected count is 3.39. | | | |

| **Adoption of M-Health innovations requires exorbitant cost in infrastructure and human resources development * Category of hospital classification Crosstabulation** | | | | | |
| --- | --- | --- | --- | --- | --- |
| Count | | | | | |
|  | | Category of hospital classification | | | Total |
|  |  | Public Hospital | Private Hospital | Faith-Based Hospital/NGO |  |
| Adoption of M-Health innovations requires exorbitant cost in infrastructure and human resources development | Strongly disagree | 10 | 16 | 2 | 28 |
|  | Disagree | 37 | 24 | 12 | 73 |
|  | Agree | 37 | 25 | 12 | 74 |
|  | Strongly Agree | 16 | 9 | 7 | 32 |
| Total | | 100 | 74 | 33 | 207 |

| **Crosstab** | | | | | | |
| --- | --- | --- | --- | --- | --- | --- |
| Count | | | | | | |
|  | | Adoption of M-Health innovations requires exorbitant cost in infrastructure and human resources development | | | | Total |
|  |  | Strongly disgree | Disagree | Agree | Strongly Agree |  |
| Classification level of facility | Level IV | 19 | 55 | 65 | 28 | 167 |
|  | Level V | 6 | 14 | 8 | 4 | 32 |
|  | Level VI | 3 | 4 | 1 | 0 | 8 |
| Total | | 28 | 73 | 74 | 32 | 207 |

| **Chi-Square Tests** | | | |
| --- | --- | --- | --- |
|  | Value | df | Asymptotic Significance (2-sided) |
| Pearson Chi-Square | 10.335^a^ | 6 | .111 |
| Likelihood Ratio | 10.846 | 6 | .093 |
| Linear-by-Linear Association | 8.643 | 1 | .003 |
| N of Valid Cases | 207 |  |  |
| a. 6 cells (50.0%) have expected count less than 5. The minimum expected count is 1.08. | | | |

**Category of hospital classification * Adoption of M-Health innovations requires exorbitant cost in infrastructure and human resources development**

| **Crosstab** | | | | | | |
| --- | --- | --- | --- | --- | --- | --- |
| Count | | | | | | |
|  | | Adoption of M-Health innovations requires exorbitant cost in infrastructure and human resources development | | | | Total |
|  |  | Strongly disgree | Disagree | Agree | Strongly Agree |  |
| Category of hospital classification | Public Hospital | 10 | 37 | 37 | 16 | 100 |
|  | Private Hospital | 16 | 24 | 25 | 9 | 74 |
|  | Faith-Based Hospital/NGO | 2 | 12 | 12 | 7 | 33 |
| Total | | 28 | 73 | 74 | 32 | 207 |

| **Chi-Square Tests** | | | |
| --- | --- | --- | --- |
|  | Value | df | Asymptotic Significance (2-sided) |
| Pearson Chi-Square | 7.502^a^ | 6 | .277 |
| Likelihood Ratio | 7.366 | 6 | .288 |
| Linear-by-Linear Association | .004 | 1 | .950 |
| N of Valid Cases | 207 |  |  |
| a. 1 cells (8.3%) have expected count less than 5. The minimum expected count is 4.46. | | | |

**Geographical category of facility classification * Adoption of M-Health innovations requires exorbitant cost in infrastructure and human resources development**

| **Crosstab** | | | | | | |
| --- | --- | --- | --- | --- | --- | --- |
| Count | | | | | | |
|  | | Adoption of M-Health innovations requires exorbitant cost in infrastructure and human resources development | | | | Total |
|  |  | Strongly disgree | Disagree | Agree | Strongly Agree |  |
| Geographical category of facility classification | Urban | 15 | 28 | 22 | 10 | 75 |
|  | Semi-urban | 9 | 26 | 28 | 15 | 78 |
|  | Rural | 4 | 19 | 24 | 7 | 54 |
| Total | | 28 | 73 | 74 | 32 | 207 |

| **Chi-Square Tests** | | | |
| --- | --- | --- | --- |
|  | Value | df | Asymptotic Significance (2-sided) |
| Pearson Chi-Square | 7.381^a^ | 6 | .287 |
| Likelihood Ratio | 7.333 | 6 | .291 |
| N of Valid Cases | 207 |  |  |
| a. 0 cells (0.0%) have expected count less than 5. The minimum expected count is 7.30. | | | |

**Current annual number of patients (outpatients and in-patients) * Adoption of M-Health innovations requires exorbitant cost in infrastructure and human resources development**

| **Crosstab** | | | | | | |
| --- | --- | --- | --- | --- | --- | --- |
| Count | | | | | | |
|  | | Adoption of M-Health innovations requires exorbitant cost in infrastructure and human resources development | | | | Total |
|  |  | Strongly disgree | Disagree | Agree | Strongly Agree |  |
| Current annual number of patients (outpatients and in-patients) | Less than 200 | 3 | 16 | 26 | 4 | 49 |
|  | 200-500 | 9 | 18 | 15 | 13 | 55 |
|  | 501-1000 | 4 | 6 | 9 | 3 | 22 |
|  | 1001-1500 | 1 | 5 | 2 | 5 | 13 |
|  | 1501-2000 | 2 | 7 | 5 | 0 | 14 |
|  | 2001 and above | 8 | 19 | 16 | 7 | 50 |
| Total | | 27 | 71 | 73 | 32 | 203 |

| **Chi-Square Tests** | | | |
| --- | --- | --- | --- |
|  | Value | df | Asymptotic Significance (2-sided) |
| Pearson Chi-Square | 22.493^a^ | 15 | .096 |
| Likelihood Ratio | 23.965 | 15 | .066 |
| Linear-by-Linear Association | 1.569 | 1 | .210 |
| N of Valid Cases | 203 |  |  |
| a. 9 cells (37.5%) have expected count less than 5. The minimum expected count is 1.73. | | | |

**Number of staff * Adoption of M-Health innovations requires exorbitant cost in infrastructure and human resources development**

| **Crosstab** | | | | | | |
| --- | --- | --- | --- | --- | --- | --- |
| Count | | | | | | |
|  | | Adoption of M-Health innovations requires exorbitant cost in infrastructure and human resources development | | | | Total |
|  |  | Strongly disgree | Disagree | Agree | Strongly Agree |  |
| Number of staff | Less than 100 | 16 | 40 | 45 | 22 | 123 |
|  | 100-200 | 6 | 16 | 22 | 7 | 51 |
|  | 201-300 | 3 | 3 | 3 | 2 | 11 |
|  | 301-500 | 1 | 7 | 3 | 1 | 12 |
|  | 501 and above | 2 | 7 | 1 | 0 | 10 |
| Total | | 28 | 73 | 74 | 32 | 207 |

| **Chi-Square Tests** | | | |
| --- | --- | --- | --- |
|  | Value | df | Asymptotic Significance (2-sided) |
| Pearson Chi-Square | 13.967^a^ | 12 | .303 |
| Likelihood Ratio | 14.948 | 12 | .244 |
| Linear-by-Linear Association | 5.169 | 1 | .023 |
| N of Valid Cases | 207 |  |  |
| a. 12 cells (60.0%) have expected count less than 5. The minimum expected count is 1.35. | | | |

**County * Adoption of M-Health innovations requires exorbitant cost in infrastructure and human resources development**

| **Crosstab** | | | | | | |
| --- | --- | --- | --- | --- | --- | --- |
| Count | | | | | | |
|  | | Adoption of M-Health innovations requires exorbitant cost in infrastructure and human resources development | | | | Total |
|  |  | Strongly disgree | Disagree | Agree | Strongly Agree |  |
| County | No | 4 | 12 | 13 | 6 | 35 |
|  | Yes | 24 | 61 | 61 | 26 | 172 |
| Total | | 28 | 73 | 74 | 32 | 207 |

| **Chi-Square Tests** | | | |
| --- | --- | --- | --- |
|  | Value | df | Asymptotic Significance (2-sided) |
| Pearson Chi-Square | .249^a^ | 3 | .969 |
| Likelihood Ratio | .253 | 3 | .969 |
| N of Valid Cases | 207 |  |  |
| a. 1 cells (12.5%) have expected count less than 5. The minimum expected count is 4.73. | | | |

**Country * Adoption of M-Health innovations requires exorbitant cost in infrastructure and human resources development**

| **Crosstab** | | | | | | |
| --- | --- | --- | --- | --- | --- | --- |
| Count | | | | | | |
|  | | Adoption of M-Health innovations requires exorbitant cost in infrastructure and human resources development | | | | Total |
|  |  | Strongly disgree | Disagree | Agree | Strongly Agree |  |
| Country | No | 8 | 22 | 20 | 8 | 58 |
|  | Yes | 20 | 51 | 54 | 23 | 148 |
|  | 2 | 0 | 0 | 0 | 1 | 1 |
| Total | | 28 | 73 | 74 | 32 | 207 |

| **Chi-Square Tests** | | | |
| --- | --- | --- | --- |
|  | Value | df | Asymptotic Significance (2-sided) |
| Pearson Chi-Square | 5.769^a^ | 6 | .450 |
| Likelihood Ratio | 4.036 | 6 | .672 |
| N of Valid Cases | 207 |  |  |
| a. 4 cells (33.3%) have expected count less than 5. The minimum expected count is .14. | | | |

**Eastern Africa * Adoption of M-Health innovations requires exorbitant cost in infrastructure and human resources development**

| **Crosstab** | | | | | | |
| --- | --- | --- | --- | --- | --- | --- |
| Count | | | | | | |
|  | | Adoption of M-Health innovations requires exorbitant cost in infrastructure and human resources development | | | | Total |
|  |  | Strongly disgree | Disagree | Agree | Strongly Agree |  |
| Eastern Africa | No | 11 | 47 | 48 | 23 | 129 |
|  | Yes | 17 | 26 | 26 | 9 | 78 |
| Total | | 28 | 73 | 74 | 32 | 207 |

| **Chi-Square Tests** | | | |
| --- | --- | --- | --- |
|  | Value | df | Asymptotic Significance (2-sided) |
| Pearson Chi-Square | 7.907^a^ | 3 | .048 |
| Likelihood Ratio | 7.705 | 3 | .053 |
| N of Valid Cases | 207 |  |  |
| a. 0 cells (0.0%) have expected count less than 5. The minimum expected count is 10.55. | | | |

Trialability

| **M-Health needs to be piloted first in order to demonstrate that it is better than using current manual systems * Category of hospital classification Crosstabulation** | | | | | |
| --- | --- | --- | --- | --- | --- |
| Count | | | | | |
|  | | Category of hospital classification | | | Total |
|  |  | Public Hospital | Private Hospital | Faith-Based Hospital/NGO |  |
| M-Health needs to be piloted first in order to demonstrate that it is better than using current manual systems | Strongly disagree | 13 | 2 | 2 | 17 |
|  | Disagree | 8 | 9 | 4 | 21 |
|  | Agree | 34 | 30 | 8 | 72 |
|  | Strongly Agree | 47 | 35 | 19 | 101 |
| Total | | 102 | 76 | 33 | 211 |

| **Crosstab** | | | | | | |
| --- | --- | --- | --- | --- | --- | --- |
| Count | | | | | | |
|  | | M-Health needs to be piloted first in order to demonstrate that it is better than using current manual systems | | | | Total |
|  |  | Strongly disgree | Disagree | Agree | Strongly Agree |  |
| Classification level of facility | Level IV | 14 | 16 | 61 | 79 | 170 |
|  | Level V | 3 | 4 | 8 | 18 | 33 |
|  | Level VI | 0 | 1 | 3 | 4 | 8 |
| Total | | 17 | 21 | 72 | 101 | 211 |

| **Chi-Square Tests** | | | |
| --- | --- | --- | --- |
|  | Value | df | Asymptotic Significance (2-sided) |
| Pearson Chi-Square | 2.463^a^ | 6 | .873 |
| Likelihood Ratio | 3.183 | 6 | .786 |
| Linear-by-Linear Association | .240 | 1 | .625 |
| N of Valid Cases | 211 |  |  |
| a. 6 cells (50.0%) have expected count less than 5. The minimum expected count is .64. | | | |

**Category of hospital classification * M-Health needs to be piloted first in order to demonstrate that it is better than using current manual systems**

| **Crosstab** | | | | | | |
| --- | --- | --- | --- | --- | --- | --- |
| Count | | | | | | |
|  | | M-Health needs to be piloted first in order to demonstrate that it is better than using current manual systems | | | | Total |
|  |  | Strongly disgree | Disagree | Agree | Strongly Agree |  |
| Category of hospital classification | Public Hospital | 13 | 8 | 34 | 47 | 102 |
|  | Private Hospital | 2 | 9 | 30 | 35 | 76 |
|  | Faith-Based Hospital/NGO | 2 | 4 | 8 | 19 | 33 |
| Total | | 17 | 21 | 72 | 101 | 211 |

| **Chi-Square Tests** | | | |
| --- | --- | --- | --- |
|  | Value | df | Asymptotic Significance (2-sided) |
| Pearson Chi-Square | 8.978^a^ | 6 | .175 |
| Likelihood Ratio | 9.613 | 6 | .142 |
| Linear-by-Linear Association | 1.765 | 1 | .184 |
| N of Valid Cases | 211 |  |  |
| a. 2 cells (16.7%) have expected count less than 5. The minimum expected count is 2.66. | | | |

**Geographical category of facility classification * M-Health needs to be piloted first in order to demonstrate that it is better than using current manual systems**

| **Crosstab** | | | | | | |
| --- | --- | --- | --- | --- | --- | --- |
| Count | | | | | | |
|  | | M-Health needs to be piloted first in order to demonstrate that it is better than using current manual systems | | | | Total |
|  |  | Strongly disgree | Disagree | Agree | Strongly Agree |  |
| Geographical category of facility classification | Urban | 5 | 9 | 26 | 37 | 77 |
|  | Semi-urban | 5 | 8 | 28 | 39 | 80 |
|  | Rural | 7 | 4 | 18 | 25 | 54 |
| Total | | 17 | 21 | 72 | 101 | 211 |

| **Chi-Square Tests** | | | |
| --- | --- | --- | --- |
|  | Value | df | Asymptotic Significance (2-sided) |
| Pearson Chi-Square | 2.828^a^ | 6 | .830 |
| Likelihood Ratio | 2.640 | 6 | .852 |
| N of Valid Cases | 211 |  |  |
| a. 1 cells (8.3%) have expected count less than 5. The minimum expected count is 4.35. | | | |

**Current annual number of patients (outpatients and in-patients) * M-Health needs to be piloted first in order to demonstrate that it is better than using current manual systems**

| **Crosstab** | | | | | | |
| --- | --- | --- | --- | --- | --- | --- |
| Count | | | | | | |
|  | | M-Health needs to be piloted first in order to demonstrate that it is better than using current manual systems | | | | Total |
|  |  | Strongly disgree | Disagree | Agree | Strongly Agree |  |
| Current annual number of patients (outpatients and in-patients) | Less than 200 | 4 | 5 | 16 | 24 | 49 |
|  | 200-500 | 3 | 6 | 19 | 28 | 56 |
|  | 501-1000 | 0 | 2 | 10 | 12 | 24 |
|  | 1001-1500 | 1 | 2 | 4 | 6 | 13 |
|  | 1501-2000 | 0 | 3 | 5 | 7 | 15 |
|  | 2001 and above | 9 | 3 | 15 | 23 | 50 |
| Total | | 17 | 21 | 69 | 100 | 207 |

| **Chi-Square Tests** | | | |
| --- | --- | --- | --- |
|  | Value | df | Asymptotic Significance (2-sided) |
| Pearson Chi-Square | 13.159^a^ | 15 | .590 |
| Likelihood Ratio | 14.699 | 15 | .473 |
| Linear-by-Linear Association | 1.391 | 1 | .238 |
| N of Valid Cases | 207 |  |  |
| a. 11 cells (45.8%) have expected count less than 5. The minimum expected count is 1.07. | | | |

**Number of staff * M-Health needs to be piloted first in order to demonstrate that it is better than using current manual systems**

| **Crosstab** | | | | | | |
| --- | --- | --- | --- | --- | --- | --- |
| Count | | | | | | |
|  | | M-Health needs to be piloted first in order to demonstrate that it is better than using current manual systems | | | | Total |
|  |  | Strongly disgree | Disagree | Agree | Strongly Agree |  |
| Number of staff | Less than 100 | 8 | 13 | 48 | 56 | 125 |
|  | 100-200 | 5 | 5 | 12 | 29 | 51 |
|  | 201-300 | 1 | 0 | 7 | 5 | 13 |
|  | 301-500 | 2 | 1 | 4 | 5 | 12 |
|  | 501 and above | 1 | 2 | 1 | 6 | 10 |
| Total | | 17 | 21 | 72 | 101 | 211 |

| **Chi-Square Tests** | | | |
| --- | --- | --- | --- |
|  | Value | df | Asymptotic Significance (2-sided) |
| Pearson Chi-Square | 11.427^a^ | 12 | .493 |
| Likelihood Ratio | 12.820 | 12 | .382 |
| Linear-by-Linear Association | .129 | 1 | .720 |
| N of Valid Cases | 211 |  |  |
| a. 11 cells (55.0%) have expected count less than 5. The minimum expected count is .81. | | | |

**County * M-Health needs to be piloted first in order to demonstrate that it is better than using current manual systems**

| **Crosstab** | | | | | | |
| --- | --- | --- | --- | --- | --- | --- |
| Count | | | | | | |
|  | | M-Health needs to be piloted first in order to demonstrate that it is better than using current manual systems | | | | Total |
|  |  | Strongly disgree | Disagree | Agree | Strongly Agree |  |
| County | No | 3 | 3 | 14 | 16 | 36 |
|  | Yes | 14 | 18 | 58 | 85 | 175 |
| Total | | 17 | 21 | 72 | 101 | 211 |

| **Chi-Square Tests** | | | |
| --- | --- | --- | --- |
|  | Value | df | Asymptotic Significance (2-sided) |
| Pearson Chi-Square | .514^a^ | 3 | .916 |
| Likelihood Ratio | .511 | 3 | .916 |
| N of Valid Cases | 211 |  |  |
| a. 2 cells (25.0%) have expected count less than 5. The minimum expected count is 2.90. | | | |

**Country * M-Health needs to be piloted first in order to demonstrate that it is better than using current manual systems**

| **Crosstab** | | | | | | |
| --- | --- | --- | --- | --- | --- | --- |
| Count | | | | | | |
|  | | M-Health needs to be piloted first in order to demonstrate that it is better than using current manual systems | | | | Total |
|  |  | Strongly disgree | Disagree | Agree | Strongly Agree |  |
| Country | No | 4 | 7 | 18 | 32 | 61 |
|  | Yes | 13 | 13 | 54 | 69 | 149 |
|  | 2 | 0 | 1 | 0 | 0 | 1 |
| Total | | 17 | 21 | 72 | 101 | 211 |

| **Chi-Square Tests** | | | |
| --- | --- | --- | --- |
|  | Value | df | Asymptotic Significance (2-sided) |
| Pearson Chi-Square | 10.589^a^ | 6 | .102 |
| Likelihood Ratio | 6.176 | 6 | .404 |
| N of Valid Cases | 211 |  |  |
| a. 5 cells (41.7%) have expected count less than 5. The minimum expected count is .08. | | | |

**Eastern Africa * M-Health needs to be piloted first in order to demonstrate that it is better than using current manual systems**

| **Crosstab** | | | | | | |
| --- | --- | --- | --- | --- | --- | --- |
| Count | | | | | | |
|  | | M-Health needs to be piloted first in order to demonstrate that it is better than using current manual systems | | | | Total |
|  |  | Strongly disgree | Disagree | Agree | Strongly Agree |  |
| Eastern Africa | No | 12 | 13 | 44 | 63 | 132 |
|  | Yes | 5 | 8 | 28 | 38 | 79 |
| Total | | 17 | 21 | 72 | 101 | 211 |

| **Chi-Square Tests** | | | |
| --- | --- | --- | --- |
|  | Value | df | Asymptotic Significance (2-sided) |
| Pearson Chi-Square | .538^a^ | 3 | .911 |
| Likelihood Ratio | .554 | 3 | .907 |
| N of Valid Cases | 211 |  |  |
| a. 0 cells (0.0%) have expected count less than 5. The minimum expected count is 6.36. | | | |

| **M-Health can be piloted without serious negative impact to patients and hospital operations * Category of hospital classification Crosstabulation** | | | | | |
| --- | --- | --- | --- | --- | --- |
| Count | | | | | |
|  | | Category of hospital classification | | | Total |
|  |  | Public Hospital | Private Hospital | Faith-Based Hospital/NGO |  |
| M-Health can be piloted without serious negative impact to patients and hospital operations | Strongly disagree | 2 | 4 | 0 | 6 |
|  | Disagree | 18 | 6 | 7 | 31 |
|  | Agree | 58 | 35 | 15 | 108 |
|  | Strongly Agree | 21 | 28 | 10 | 59 |
| Total | | 99 | 73 | 32 | 204 |

| **Crosstab** | | | | | | |
| --- | --- | --- | --- | --- | --- | --- |
| Count | | | | | | |
|  | | M-Health can be piloted without serious negative impact to patients and hospital operations | | | | Total |
|  |  | Strongly disgree | Disagree | Agree | Strongly Agree |  |
| Classification level of facility | Level IV | 5 | 26 | 89 | 44 | 164 |
|  | Level V | 1 | 4 | 18 | 9 | 32 |
|  | Level VI | 0 | 1 | 1 | 6 | 8 |
| Total | | 6 | 31 | 108 | 59 | 204 |

| **Chi-Square Tests** | | | |
| --- | --- | --- | --- |
|  | Value | df | Asymptotic Significance (2-sided) |
| Pearson Chi-Square | 9.204^a^ | 6 | .162 |
| Likelihood Ratio | 8.757 | 6 | .188 |
| Linear-by-Linear Association | 2.914 | 1 | .088 |
| N of Valid Cases | 204 |  |  |
| a. 7 cells (58.3%) have expected count less than 5. The minimum expected count is .24. | | | |

**Category of hospital classification * M-Health can be piloted without serious negative impact to patients and hospital operations**

| **Crosstab** | | | | | | |
| --- | --- | --- | --- | --- | --- | --- |
| Count | | | | | | |
|  | | M-Health can be piloted without serious negative impact to patients and hospital operations | | | | Total |
|  |  | Strongly disgree | Disagree | Agree | Strongly Agree |  |
| Category of hospital classification | Public Hospital | 2 | 18 | 58 | 21 | 99 |
|  | Private Hospital | 4 | 6 | 35 | 28 | 73 |
|  | Faith-Based Hospital/NGO | 0 | 7 | 15 | 10 | 32 |
| Total | | 6 | 31 | 108 | 59 | 204 |

| **Chi-Square Tests** | | | |
| --- | --- | --- | --- |
|  | Value | df | Asymptotic Significance (2-sided) |
| Pearson Chi-Square | 12.188^a^ | 6 | .058 |
| Likelihood Ratio | 13.160 | 6 | .041 |
| Linear-by-Linear Association | 1.415 | 1 | .234 |
| N of Valid Cases | 204 |  |  |
| a. 4 cells (33.3%) have expected count less than 5. The minimum expected count is .94. | | | |

**Geographical category of facility classification * M-Health can be piloted without serious negative impact to patients and hospital operations**

| **Crosstab** | | | | | | |
| --- | --- | --- | --- | --- | --- | --- |
| Count | | | | | | |
|  | | M-Health can be piloted without serious negative impact to patients and hospital operations | | | | Total |
|  |  | Strongly disgree | Disagree | Agree | Strongly Agree |  |
| Geographical category of facility classification | Urban | 2 | 10 | 37 | 23 | 72 |
|  | Semi-urban | 4 | 10 | 40 | 25 | 79 |
|  | Rural | 0 | 11 | 31 | 11 | 53 |
| Total | | 6 | 31 | 108 | 59 | 204 |

| **Chi-Square Tests** | | | |
| --- | --- | --- | --- |
|  | Value | df | Asymptotic Significance (2-sided) |
| Pearson Chi-Square | 6.341^a^ | 6 | .386 |
| Likelihood Ratio | 7.707 | 6 | .260 |
| N of Valid Cases | 204 |  |  |
| a. 3 cells (25.0%) have expected count less than 5. The minimum expected count is 1.56. | | | |

**Current annual number of patients (outpatients and in-patients) * M-Health can be piloted without serious negative impact to patients and hospital operations**

| **Crosstab** | | | | | | |
| --- | --- | --- | --- | --- | --- | --- |
| Count | | | | | | |
|  | | M-Health can be piloted without serious negative impact to patients and hospital operations | | | | Total |
|  |  | Strongly disgree | Disagree | Agree | Strongly Agree |  |
| Current annual number of patients (outpatients and in-patients) | Less than 200 | 1 | 8 | 22 | 17 | 48 |
|  | 200-500 | 3 | 7 | 32 | 11 | 53 |
|  | 501-1000 | 0 | 7 | 12 | 5 | 24 |
|  | 1001-1500 | 0 | 1 | 7 | 5 | 13 |
|  | 1501-2000 | 1 | 2 | 8 | 3 | 14 |
|  | 2001 and above | 1 | 6 | 24 | 17 | 48 |
| Total | | 6 | 31 | 105 | 58 | 200 |

| **Chi-Square Tests** | | | |
| --- | --- | --- | --- |
|  | Value | df | Asymptotic Significance (2-sided) |
| Pearson Chi-Square | 12.363^a^ | 15 | .651 |
| Likelihood Ratio | 12.651 | 15 | .629 |
| Linear-by-Linear Association | .492 | 1 | .483 |
| N of Valid Cases | 200 |  |  |
| a. 11 cells (45.8%) have expected count less than 5. The minimum expected count is .39. | | | |

**Number of staff * M-Health can be piloted without serious negative impact to patients and hospital operations**

| **Crosstab** | | | | | | |
| --- | --- | --- | --- | --- | --- | --- |
| Count | | | | | | |
|  | | M-Health can be piloted without serious negative impact to patients and hospital operations | | | | Total |
|  |  | Strongly disgree | Disagree | Agree | Strongly Agree |  |
| Number of staff | Less than 100 | 3 | 18 | 65 | 34 | 120 |
|  | 100-200 | 2 | 7 | 29 | 12 | 50 |
|  | 201-300 | 0 | 4 | 5 | 4 | 13 |
|  | 301-500 | 0 | 1 | 8 | 3 | 12 |
|  | 501 and above | 1 | 1 | 1 | 6 | 9 |
| Total | | 6 | 31 | 108 | 59 | 204 |

| **Chi-Square Tests** | | | |
| --- | --- | --- | --- |
|  | Value | df | Asymptotic Significance (2-sided) |
| Pearson Chi-Square | 14.781^a^ | 12 | .254 |
| Likelihood Ratio | 14.521 | 12 | .269 |
| Linear-by-Linear Association | .399 | 1 | .528 |
| N of Valid Cases | 204 |  |  |
| a. 12 cells (60.0%) have expected count less than 5. The minimum expected count is .26. | | | |

**County * M-Health can be piloted without serious negative impact to patients and hospital operations**

| **Crosstab** | | | | | | |
| --- | --- | --- | --- | --- | --- | --- |
| Count | | | | | | |
|  | | M-Health can be piloted without serious negative impact to patients and hospital operations | | | | Total |
|  |  | Strongly disgree | Disagree | Agree | Strongly Agree |  |
| County | No | 1 | 6 | 14 | 13 | 34 |
|  | Yes | 5 | 25 | 94 | 46 | 170 |
| Total | | 6 | 31 | 108 | 59 | 204 |

| **Chi-Square Tests** | | | |
| --- | --- | --- | --- |
|  | Value | df | Asymptotic Significance (2-sided) |
| Pearson Chi-Square | 2.452^a^ | 3 | .484 |
| Likelihood Ratio | 2.427 | 3 | .489 |
| N of Valid Cases | 204 |  |  |
| a. 1 cells (12.5%) have expected count less than 5. The minimum expected count is 1.00. | | | |

**Country * M-Health can be piloted without serious negative impact to patients and hospital operations**

| **Crosstab** | | | | | | |
| --- | --- | --- | --- | --- | --- | --- |
| Count | | | | | | |
|  | | M-Health can be piloted without serious negative impact to patients and hospital operations | | | | Total |
|  |  | Strongly disgree | Disagree | Agree | Strongly Agree |  |
| Country | No | 1 | 10 | 30 | 16 | 57 |
|  | Yes | 5 | 20 | 78 | 43 | 146 |
|  | 2 | 0 | 1 | 0 | 0 | 1 |
| Total | | 6 | 31 | 108 | 59 | 204 |

| **Chi-Square Tests** | | | |
| --- | --- | --- | --- |
|  | Value | df | Asymptotic Significance (2-sided) |
| Pearson Chi-Square | 6.428^a^ | 6 | .377 |
| Likelihood Ratio | 4.654 | 6 | .589 |
| N of Valid Cases | 204 |  |  |
| a. 6 cells (50.0%) have expected count less than 5. The minimum expected count is .03. | | | |

**Eastern Africa * M-Health can be piloted without serious negative impact to patients and hospital operations**

| **Crosstab** | | | | | | |
| --- | --- | --- | --- | --- | --- | --- |
| Count | | | | | | |
|  | | M-Health can be piloted without serious negative impact to patients and hospital operations | | | | Total |
|  |  | Strongly disgree | Disagree | Agree | Strongly Agree |  |
| Eastern Africa | No | 3 | 24 | 63 | 37 | 127 |
|  | Yes | 3 | 7 | 45 | 22 | 77 |
| Total | | 6 | 31 | 108 | 59 | 204 |

| **Chi-Square Tests** | | | |
| --- | --- | --- | --- |
|  | Value | df | Asymptotic Significance (2-sided) |
| Pearson Chi-Square | 4.129^a^ | 3 | .248 |
| Likelihood Ratio | 4.346 | 3 | .226 |
| N of Valid Cases | 204 |  |  |
| a. 2 cells (25.0%) have expected count less than 5. The minimum expected count is 2.26. | | | |

Acquisition strategies

| **It is more strategic to adopt M-Health when the technology is leased to the hospital * Category of hospital classification Crosstabulation** | | | | | |
| --- | --- | --- | --- | --- | --- |
| Count | | | | | |
|  | | Category of hospital classification | | | Total |
|  |  | Public Hospital | Private Hospital | Faith-Based Hospital/NGO |  |
| It is more strategic to adopt M-Health when the technology is leased to the hospital | Strongly disagree | 18 | 16 | 7 | 41 |
|  | Disagree | 41 | 27 | 7 | 75 |
|  | Agree | 21 | 18 | 11 | 50 |
|  | Strongly Agree | 16 | 13 | 4 | 33 |
| Total | | 96 | 74 | 29 | 199 |

| **Crosstab** | | | | | | |
| --- | --- | --- | --- | --- | --- | --- |
| Count | | | | | | |
|  | | It is more strategic to adopt M-Health when the technology is leased to the hospital | | | | Total |
|  |  | Strongly disgree | Disagree | Agree | Strongly Agree |  |
| Classification level of facility | Level IV | 34 | 64 | 37 | 25 | 160 |
|  | Level V | 6 | 10 | 11 | 6 | 33 |
|  | Level VI | 1 | 1 | 2 | 2 | 6 |
| Total | | 41 | 75 | 50 | 33 | 199 |

| **Chi-Square Tests** | | | |
| --- | --- | --- | --- |
|  | Value | df | Asymptotic Significance (2-sided) |
| Pearson Chi-Square | 4.035^a^ | 6 | .672 |
| Likelihood Ratio | 3.935 | 6 | .685 |
| Linear-by-Linear Association | 2.189 | 1 | .139 |
| N of Valid Cases | 199 |  |  |
| a. 4 cells (33.3%) have expected count less than 5. The minimum expected count is .99. | | | |

**Category of hospital classification * It is more strategic to adopt M-Health when the technology is leased to the hospital**

| **Crosstab** | | | | | | |
| --- | --- | --- | --- | --- | --- | --- |
| Count | | | | | | |
|  | | It is more strategic to adopt M-Health when the technology is leased to the hospital | | | | Total |
|  |  | Strongly disgree | Disagree | Agree | Strongly Agree |  |
| Category of hospital classification | Public Hospital | 18 | 41 | 21 | 16 | 96 |
|  | Private Hospital | 16 | 27 | 18 | 13 | 74 |
|  | Faith-Based Hospital/NGO | 7 | 7 | 11 | 4 | 29 |
| Total | | 41 | 75 | 50 | 33 | 199 |

| **Chi-Square Tests** | | | |
| --- | --- | --- | --- |
|  | Value | df | Asymptotic Significance (2-sided) |
| Pearson Chi-Square | 4.951^a^ | 6 | .550 |
| Likelihood Ratio | 4.896 | 6 | .557 |
| Linear-by-Linear Association | .050 | 1 | .823 |
| N of Valid Cases | 199 |  |  |
| a. 1 cells (8.3%) have expected count less than 5. The minimum expected count is 4.81. | | | |

**Geographical category of facility classification * It is more strategic to adopt M-Health when the technology is leased to the hospital**

| **Crosstab** | | | | | | |
| --- | --- | --- | --- | --- | --- | --- |
| Count | | | | | | |
|  | | It is more strategic to adopt M-Health when the technology is leased to the hospital | | | | Total |
|  |  | Strongly disgree | Disagree | Agree | Strongly Agree |  |
| Geographical category of facility classification | Urban | 13 | 22 | 23 | 17 | 75 |
|  | Semi-urban | 16 | 34 | 17 | 9 | 76 |
|  | Rural | 12 | 19 | 10 | 7 | 48 |
| Total | | 41 | 75 | 50 | 33 | 199 |

| **Chi-Square Tests** | | | |
| --- | --- | --- | --- |
|  | Value | df | Asymptotic Significance (2-sided) |
| Pearson Chi-Square | 7.602^a^ | 6 | .269 |
| Likelihood Ratio | 7.569 | 6 | .271 |
| N of Valid Cases | 199 |  |  |
| a. 0 cells (0.0%) have expected count less than 5. The minimum expected count is 7.96. | | | |

**Current annual number of patients (outpatients and in-patients) * It is more strategic to adopt M-Health when the technology is leased to the hospital**

| **Crosstab** | | | | | | |
| --- | --- | --- | --- | --- | --- | --- |
| Count | | | | | | |
|  | | It is more strategic to adopt M-Health when the technology is leased to the hospital | | | | Total |
|  |  | Strongly disgree | Disagree | Agree | Strongly Agree |  |
| Current annual number of patients (outpatients and in-patients) | Less than 200 | 10 | 22 | 5 | 6 | 43 |
|  | 200-500 | 15 | 13 | 14 | 11 | 53 |
|  | 501-1000 | 4 | 8 | 7 | 4 | 23 |
|  | 1001-1500 | 3 | 5 | 2 | 3 | 13 |
|  | 1501-2000 | 5 | 6 | 4 | 0 | 15 |
|  | 2001 and above | 4 | 18 | 17 | 9 | 48 |
| Total | | 41 | 72 | 49 | 33 | 195 |

| **Chi-Square Tests** | | | |
| --- | --- | --- | --- |
|  | Value | df | Asymptotic Significance (2-sided) |
| Pearson Chi-Square | 20.561^a^ | 15 | .151 |
| Likelihood Ratio | 24.521 | 15 | .057 |
| Linear-by-Linear Association | 2.550 | 1 | .110 |
| N of Valid Cases | 195 |  |  |
| a. 9 cells (37.5%) have expected count less than 5. The minimum expected count is 2.20. | | | |

**Number of staff * It is more strategic to adopt M-Health when the technology is leased to the hospital**

| **Crosstab** | | | | | | |
| --- | --- | --- | --- | --- | --- | --- |
| Count | | | | | | |
|  | | It is more strategic to adopt M-Health when the technology is leased to the hospital | | | | Total |
|  |  | Strongly disgree | Disagree | Agree | Strongly Agree |  |
| Number of staff | Less than 100 | 26 | 50 | 21 | 20 | 117 |
|  | 100-200 | 12 | 13 | 18 | 7 | 50 |
|  | 201-300 | 2 | 3 | 7 | 0 | 12 |
|  | 301-500 | 0 | 6 | 2 | 4 | 12 |
|  | 501 and above | 1 | 3 | 2 | 2 | 8 |
| Total | | 41 | 75 | 50 | 33 | 199 |

| **Chi-Square Tests** | | | |
| --- | --- | --- | --- |
|  | Value | df | Asymptotic Significance (2-sided) |
| Pearson Chi-Square | 21.787^a^ | 12 | .040 |
| Likelihood Ratio | 24.583 | 12 | .017 |
| Linear-by-Linear Association | 3.065 | 1 | .080 |
| N of Valid Cases | 199 |  |  |
| a. 12 cells (60.0%) have expected count less than 5. The minimum expected count is 1.33. | | | |

**County * It is more strategic to adopt M-Health when the technology is leased to the hospital**

| **Crosstab** | | | | | | |
| --- | --- | --- | --- | --- | --- | --- |
| Count | | | | | | |
|  | | It is more strategic to adopt M-Health when the technology is leased to the hospital | | | | Total |
|  |  | Strongly disgree | Disagree | Agree | Strongly Agree |  |
| County | No | 6 | 12 | 10 | 6 | 34 |
|  | Yes | 35 | 63 | 40 | 27 | 165 |
| Total | | 41 | 75 | 50 | 33 | 199 |

| **Chi-Square Tests** | | | |
| --- | --- | --- | --- |
|  | Value | df | Asymptotic Significance (2-sided) |
| Pearson Chi-Square | .564^a^ | 3 | .905 |
| Likelihood Ratio | .559 | 3 | .906 |
| N of Valid Cases | 199 |  |  |
| a. 0 cells (0.0%) have expected count less than 5. The minimum expected count is 5.64. | | | |

**Country * It is more strategic to adopt M-Health when the technology is leased to the hospital**

| **Crosstab** | | | | | | |
| --- | --- | --- | --- | --- | --- | --- |
| Count | | | | | | |
|  | | It is more strategic to adopt M-Health when the technology is leased to the hospital | | | | Total |
|  |  | Strongly disgree | Disagree | Agree | Strongly Agree |  |
| Country | No | 11 | 24 | 14 | 7 | 56 |
|  | Yes | 30 | 50 | 36 | 26 | 142 |
|  | 2 | 0 | 1 | 0 | 0 | 1 |
| Total | | 41 | 75 | 50 | 33 | 199 |

| **Chi-Square Tests** | | | |
| --- | --- | --- | --- |
|  | Value | df | Asymptotic Significance (2-sided) |
| Pearson Chi-Square | 3.147^a^ | 6 | .790 |
| Likelihood Ratio | 3.478 | 6 | .747 |
| N of Valid Cases | 199 |  |  |
| a. 4 cells (33.3%) have expected count less than 5. The minimum expected count is .17. | | | |

**Eastern Africa * It is more strategic to adopt M-Health when the technology is leased to the hospital**

| **Crosstab** | | | | | | |
| --- | --- | --- | --- | --- | --- | --- |
| Count | | | | | | |
|  | | It is more strategic to adopt M-Health when the technology is leased to the hospital | | | | Total |
|  |  | Strongly disgree | Disagree | Agree | Strongly Agree |  |
| Eastern Africa | No | 25 | 49 | 36 | 13 | 123 |
|  | Yes | 16 | 26 | 14 | 20 | 76 |
| Total | | 41 | 75 | 50 | 33 | 199 |

| **Chi-Square Tests** | | | |
| --- | --- | --- | --- |
|  | Value | df | Asymptotic Significance (2-sided) |
| Pearson Chi-Square | 9.630^a^ | 3 | .022 |
| Likelihood Ratio | 9.470 | 3 | .024 |
| N of Valid Cases | 199 |  |  |
| a. 0 cells (0.0%) have expected count less than 5. The minimum expected count is 12.60. | | | |

| **It is more strategic to adopt M-Health when the hospital fully owned the technology * Category of hospital classification Crosstabulation** | | | | | |
| --- | --- | --- | --- | --- | --- |
| Count | | | | | |
|  | | Category of hospital classification | | | Total |
|  |  | Public Hospital | Private Hospital | Faith-Based Hospital/NGO |  |
| It is more strategic to adopt M-Health when the hospital fully owned the technology | Strongly disagree | 5 | 8 | 5 | 18 |
|  | Disagree | 17 | 9 | 0 | 26 |
|  | Agree | 33 | 36 | 9 | 78 |
|  | Strongly Agree | 45 | 20 | 18 | 83 |
| Total | | 100 | 73 | 32 | 205 |

| **Crosstab** | | | | | | |
| --- | --- | --- | --- | --- | --- | --- |
| Count | | | | | | |
|  | | It is more strategic to adopt M-Health when the hospital fully owned the technology | | | | Total |
|  |  | Strongly disgree | Disagree | Agree | Strongly Agree |  |
| Classification level of facility | Level IV | 11 | 23 | 68 | 62 | 164 |
|  | Level V | 2 | 2 | 10 | 19 | 33 |
|  | Level VI | 5 | 1 | 0 | 2 | 8 |
| Total | | 18 | 26 | 78 | 83 | 205 |

| **Chi-Square Tests** | | | |
| --- | --- | --- | --- |
|  | Value | df | Asymptotic Significance (2-sided) |
| Pearson Chi-Square | 35.960^a^ | 6 | .000 |
| Likelihood Ratio | 23.945 | 6 | .001 |
| Linear-by-Linear Association | 2.149 | 1 | .143 |
| N of Valid Cases | 205 |  |  |
| a. 6 cells (50.0%) have expected count less than 5. The minimum expected count is .70. | | | |

**Category of hospital classification * It is more strategic to adopt M-Health when the hospital fully owned the technology**

| **Crosstab** | | | | | | |
| --- | --- | --- | --- | --- | --- | --- |
| Count | | | | | | |
|  | | It is more strategic to adopt M-Health when the hospital fully owned the technology | | | | Total |
|  |  | Strongly disgree | Disagree | Agree | Strongly Agree |  |
| Category of hospital classification | Public Hospital | 5 | 17 | 33 | 45 | 100 |
|  | Private Hospital | 8 | 9 | 36 | 20 | 73 |
|  | Faith-Based Hospital/NGO | 5 | 0 | 9 | 18 | 32 |
| Total | | 18 | 26 | 78 | 83 | 205 |

| **Chi-Square Tests** | | | |
| --- | --- | --- | --- |
|  | Value | df | Asymptotic Significance (2-sided) |
| Pearson Chi-Square | 18.754^a^ | 6 | .005 |
| Likelihood Ratio | 22.692 | 6 | .001 |
| Linear-by-Linear Association | .096 | 1 | .757 |
| N of Valid Cases | 205 |  |  |
| a. 2 cells (16.7%) have expected count less than 5. The minimum expected count is 2.81. | | | |

**Geographical category of facility classification * It is more strategic to adopt M-Health when the hospital fully owned the technology**

| **Crosstab** | | | | | | |
| --- | --- | --- | --- | --- | --- | --- |
| Count | | | | | | |
|  | | It is more strategic to adopt M-Health when the hospital fully owned the technology | | | | Total |
|  |  | Strongly disgree | Disagree | Agree | Strongly Agree |  |
| Geographical category of facility classification | Urban | 8 | 8 | 31 | 27 | 74 |
|  | Semi-urban | 6 | 10 | 31 | 33 | 80 |
|  | Rural | 4 | 8 | 16 | 23 | 51 |
| Total | | 18 | 26 | 78 | 83 | 205 |

| **Chi-Square Tests** | | | |
| --- | --- | --- | --- |
|  | Value | df | Asymptotic Significance (2-sided) |
| Pearson Chi-Square | 2.584^a^ | 6 | .859 |
| Likelihood Ratio | 2.585 | 6 | .859 |
| N of Valid Cases | 205 |  |  |
| a. 1 cells (8.3%) have expected count less than 5. The minimum expected count is 4.48. | | | |

**Current annual number of patients (outpatients and in-patients) * It is more strategic to adopt M-Health when the hospital fully owned the technology**

| **Crosstab** | | | | | | |
| --- | --- | --- | --- | --- | --- | --- |
| Count | | | | | | |
|  | | It is more strategic to adopt M-Health when the hospital fully owned the technology | | | | Total |
|  |  | Strongly disgree | Disagree | Agree | Strongly Agree |  |
| Current annual number of patients (outpatients and in-patients) | Less than 200 | 4 | 7 | 17 | 18 | 46 |
|  | 200-500 | 6 | 4 | 23 | 21 | 54 |
|  | 501-1000 | 0 | 4 | 9 | 11 | 24 |
|  | 1001-1500 | 2 | 0 | 5 | 5 | 12 |
|  | 1501-2000 | 1 | 1 | 7 | 6 | 15 |
|  | 2001 and above | 5 | 10 | 14 | 21 | 50 |
| Total | | 18 | 26 | 75 | 82 | 201 |

| **Chi-Square Tests** | | | |
| --- | --- | --- | --- |
|  | Value | df | Asymptotic Significance (2-sided) |
| Pearson Chi-Square | 11.266^a^ | 15 | .734 |
| Likelihood Ratio | 14.874 | 15 | .461 |
| Linear-by-Linear Association | .047 | 1 | .829 |
| N of Valid Cases | 201 |  |  |
| a. 11 cells (45.8%) have expected count less than 5. The minimum expected count is 1.07. | | | |

**Number of staff * It is more strategic to adopt M-Health when the hospital fully owned the technology**

| **Crosstab** | | | | | | |
| --- | --- | --- | --- | --- | --- | --- |
| Count | | | | | | |
|  | | It is more strategic to adopt M-Health when the hospital fully owned the technology | | | | Total |
|  |  | Strongly disgree | Disagree | Agree | Strongly Agree |  |
| Number of staff | Less than 100 | 11 | 11 | 53 | 44 | 119 |
|  | 100-200 | 1 | 12 | 15 | 23 | 51 |
|  | 201-300 | 0 | 1 | 5 | 7 | 13 |
|  | 301-500 | 1 | 2 | 3 | 6 | 12 |
|  | 501 and above | 5 | 0 | 2 | 3 | 10 |
| Total | | 18 | 26 | 78 | 83 | 205 |

| **Chi-Square Tests** | | | |
| --- | --- | --- | --- |
|  | Value | df | Asymptotic Significance (2-sided) |
| Pearson Chi-Square | 36.193^a^ | 12 | .000 |
| Likelihood Ratio | 29.070 | 12 | .004 |
| Linear-by-Linear Association | 1.235 | 1 | .266 |
| N of Valid Cases | 205 |  |  |
| a. 12 cells (60.0%) have expected count less than 5. The minimum expected count is .88. | | | |

**County * It is more strategic to adopt M-Health when the hospital fully owned the technology**

| **Crosstab** | | | | | | |
| --- | --- | --- | --- | --- | --- | --- |
| Count | | | | | | |
|  | | It is more strategic to adopt M-Health when the hospital fully owned the technology | | | | Total |
|  |  | Strongly disgree | Disagree | Agree | Strongly Agree |  |
| County | No | 5 | 4 | 15 | 9 | 33 |
|  | Yes | 13 | 22 | 63 | 74 | 172 |
| Total | | 18 | 26 | 78 | 83 | 205 |

| **Chi-Square Tests** | | | |
| --- | --- | --- | --- |
|  | Value | df | Asymptotic Significance (2-sided) |
| Pearson Chi-Square | 4.091^a^ | 3 | .252 |
| Likelihood Ratio | 3.985 | 3 | .263 |
| N of Valid Cases | 205 |  |  |
| a. 2 cells (25.0%) have expected count less than 5. The minimum expected count is 2.90. | | | |

**Country * It is more strategic to adopt M-Health when the hospital fully owned the technology**

| **Crosstab** | | | | | | |
| --- | --- | --- | --- | --- | --- | --- |
| Count | | | | | | |
|  | | It is more strategic to adopt M-Health when the hospital fully owned the technology | | | | Total |
|  |  | Strongly disgree | Disagree | Agree | Strongly Agree |  |
| Country | No | 5 | 9 | 21 | 25 | 60 |
|  | Yes | 13 | 17 | 57 | 57 | 144 |
|  | 2 | 0 | 0 | 0 | 1 | 1 |
| Total | | 18 | 26 | 78 | 83 | 205 |

| **Chi-Square Tests** | | | |
| --- | --- | --- | --- |
|  | Value | df | Asymptotic Significance (2-sided) |
| Pearson Chi-Square | 2.120^a^ | 6 | .908 |
| Likelihood Ratio | 2.450 | 6 | .874 |
| N of Valid Cases | 205 |  |  |
| a. 4 cells (33.3%) have expected count less than 5. The minimum expected count is .09. | | | |

**Eastern Africa * It is more strategic to adopt M-Health when the hospital fully owned the technology**

| **Crosstab** | | | | | | |
| --- | --- | --- | --- | --- | --- | --- |
| Count | | | | | | |
|  | | It is more strategic to adopt M-Health when the hospital fully owned the technology | | | | Total |
|  |  | Strongly disgree | Disagree | Agree | Strongly Agree |  |
| Eastern Africa | No | 12 | 17 | 48 | 49 | 126 |
|  | Yes | 6 | 9 | 30 | 34 | 79 |
| Total | | 18 | 26 | 78 | 83 | 205 |

| **Chi-Square Tests** | | | |
| --- | --- | --- | --- |
|  | Value | df | Asymptotic Significance (2-sided) |
| Pearson Chi-Square | .581^a^ | 3 | .901 |
| Likelihood Ratio | .586 | 3 | .900 |
| N of Valid Cases | 205 |  |  |
| a. 0 cells (0.0%) have expected count less than 5. The minimum expected count is 6.94. | | | |

1. **Rate the extent to which you agree with the following statements relating the effect of the organizational determinants listed below on adoption of M-Health using 1 = Strongly Disagree, 2=Disagree, 3=Agree and 4=Strongly Agree.**

ORGANIZATIONAL DETERMINANTS

| **Decision making structure: decision to adopt or not to adopt M-Health adoption is the prerogative of the hospital’s top management only. * Category of hospital classification Crosstabulation** | | | | | |
| --- | --- | --- | --- | --- | --- |
| Count | | | | | |
|  | | Category of hospital classification | | | Total |
|  |  | Public Hospital | Private Hospital | Faith-Based Hospital/NGO |  |
| Decision making structure: decision to adopt or not to adopt M-Health adoption is the prerogative of the hospital’s top management only. | Strongly disagree | 37 | 16 | 5 | 58 |
|  | Disagree | 37 | 27 | 13 | 77 |
|  | Agree | 17 | 18 | 9 | 44 |
|  | Strongly Agree | 11 | 15 | 6 | 32 |
| Total | | 102 | 76 | 33 | 211 |

| **Crosstab** | | | | | | |
| --- | --- | --- | --- | --- | --- | --- |
| Count | | | | | | |
|  | | Decision making structure: decision to adopt or not to adopt M-Health adoption is the prerogative of the hospital’s top management only. | | | | Total |
|  |  | Strongly disgree | Disagree | Agree | Strongly Agree |  |
| Classification level of facility | Level IV | 46 | 63 | 38 | 23 | 170 |
|  | Level V | 10 | 8 | 6 | 9 | 33 |
|  | Level VI | 2 | 6 | 0 | 0 | 8 |
| Total | | 58 | 77 | 44 | 32 | 211 |

| **Chi-Square Tests** | | | |
| --- | --- | --- | --- |
|  | Value | df | Asymptotic Significance (2-sided) |
| Pearson Chi-Square | 11.415^a^ | 6 | .076 |
| Likelihood Ratio | 13.106 | 6 | .041 |
| Linear-by-Linear Association | .047 | 1 | .829 |
| N of Valid Cases | 211 |  |  |
| a. 4 cells (33.3%) have expected count less than 5. The minimum expected count is 1.21. | | | |

**Category of hospital classification * Decision making structure: decision to adopt or not to adopt M-Health adoption is the prerogative of the hospital’s top management only.**

| **Crosstab** | | | | | | |
| --- | --- | --- | --- | --- | --- | --- |
| Count | | | | | | |
|  | | Decision making structure: decision to adopt or not to adopt M-Health adoption is the prerogative of the hospital’s top management only. | | | | Total |
|  |  | Strongly disgree | Disagree | Agree | Strongly Agree |  |
| Category of hospital classification | Public Hospital | 37 | 37 | 17 | 11 | 102 |
|  | Private Hospital | 16 | 27 | 18 | 15 | 76 |
|  | Faith-Based Hospital/NGO | 5 | 13 | 9 | 6 | 33 |
| Total | | 58 | 77 | 44 | 32 | 211 |

| **Chi-Square Tests** | | | |
| --- | --- | --- | --- |
|  | Value | df | Asymptotic Significance (2-sided) |
| Pearson Chi-Square | 10.271^a^ | 6 | .114 |
| Likelihood Ratio | 10.501 | 6 | .105 |
| Linear-by-Linear Association | 7.887 | 1 | .005 |
| N of Valid Cases | 211 |  |  |
| a. 0 cells (0.0%) have expected count less than 5. The minimum expected count is 5.00. | | | |

**Geographical category of facility classification * Decision making structure: decision to adopt or not to adopt M-Health adoption is the prerogative of the hospital’s top management only.**

| **Crosstab** | | | | | | |
| --- | --- | --- | --- | --- | --- | --- |
| Count | | | | | | |
|  | | Decision making structure: decision to adopt or not to adopt M-Health adoption is the prerogative of the hospital’s top management only. | | | | Total |
|  |  | Strongly disgree | Disagree | Agree | Strongly Agree |  |
| Geographical category of facility classification | Urban | 20 | 28 | 16 | 13 | 77 |
|  | Semi-urban | 20 | 29 | 18 | 13 | 80 |
|  | Rural | 18 | 20 | 10 | 6 | 54 |
| Total | | 58 | 77 | 44 | 32 | 211 |

| **Chi-Square Tests** | | | |
| --- | --- | --- | --- |
|  | Value | df | Asymptotic Significance (2-sided) |
| Pearson Chi-Square | 1.964^a^ | 6 | .923 |
| Likelihood Ratio | 1.985 | 6 | .921 |
| N of Valid Cases | 211 |  |  |
| a. 0 cells (0.0%) have expected count less than 5. The minimum expected count is 8.19. | | | |

**Current annual number of patients (outpatients and in-patients) * Decision making structure: decision to adopt or not to adopt M-Health adoption is the prerogative of the hospital’s top management only.**

| **Crosstab** | | | | | | |
| --- | --- | --- | --- | --- | --- | --- |
| Count | | | | | | |
|  | | Decision making structure: decision to adopt or not to adopt M-Health adoption is the prerogative of the hospital’s top management only. | | | | Total |
|  |  | Strongly disgree | Disagree | Agree | Strongly Agree |  |
| Current annual number of patients (outpatients and in-patients) | Less than 200 | 12 | 18 | 14 | 5 | 49 |
|  | 200-500 | 13 | 23 | 9 | 11 | 56 |
|  | 501-1000 | 4 | 6 | 10 | 4 | 24 |
|  | 1001-1500 | 2 | 3 | 2 | 6 | 13 |
|  | 1501-2000 | 4 | 6 | 4 | 1 | 15 |
|  | 2001 and above | 22 | 19 | 4 | 5 | 50 |
| Total | | 57 | 75 | 43 | 32 | 207 |

| **Chi-Square Tests** | | | |
| --- | --- | --- | --- |
|  | Value | df | Asymptotic Significance (2-sided) |
| Pearson Chi-Square | 31.724^a^ | 15 | .007 |
| Likelihood Ratio | 29.064 | 15 | .016 |
| Linear-by-Linear Association | 4.388 | 1 | .036 |
| N of Valid Cases | 207 |  |  |
| a. 9 cells (37.5%) have expected count less than 5. The minimum expected count is 2.01. | | | |

**Number of staff * Decision making structure: decision to adopt or not to adopt M-Health adoption is the prerogative of the hospital’s top management only.**

| **Crosstab** | | | | | | |
| --- | --- | --- | --- | --- | --- | --- |
| Count | | | | | | |
|  | | Decision making structure: decision to adopt or not to adopt M-Health adoption is the prerogative of the hospital’s top management only. | | | | Total |
|  |  | Strongly disgree | Disagree | Agree | Strongly Agree |  |
| Number of staff | Less than 100 | 28 | 46 | 29 | 22 | 125 |
|  | 100-200 | 19 | 19 | 9 | 4 | 51 |
|  | 201-300 | 5 | 3 | 3 | 2 | 13 |
|  | 301-500 | 3 | 4 | 2 | 3 | 12 |
|  | 501 and above | 3 | 5 | 1 | 1 | 10 |
| Total | | 58 | 77 | 44 | 32 | 211 |

| **Chi-Square Tests** | | | |
| --- | --- | --- | --- |
|  | Value | df | Asymptotic Significance (2-sided) |
| Pearson Chi-Square | 9.263^a^ | 12 | .680 |
| Likelihood Ratio | 9.556 | 12 | .655 |
| Linear-by-Linear Association | 1.373 | 1 | .241 |
| N of Valid Cases | 211 |  |  |
| a. 12 cells (60.0%) have expected count less than 5. The minimum expected count is 1.52. | | | |

**County * Decision making structure: decision to adopt or not to adopt M-Health adoption is the prerogative of the hospital’s top management only.**

| **Crosstab** | | | | | | |
| --- | --- | --- | --- | --- | --- | --- |
| Count | | | | | | |
|  | | Decision making structure: decision to adopt or not to adopt M-Health adoption is the prerogative of the hospital’s top management only. | | | | Total |
|  |  | Strongly disgree | Disagree | Agree | Strongly Agree |  |
| County | No | 8 | 12 | 10 | 6 | 36 |
|  | Yes | 50 | 65 | 34 | 26 | 175 |
| Total | | 58 | 77 | 44 | 32 | 211 |

| **Chi-Square Tests** | | | |
| --- | --- | --- | --- |
|  | Value | df | Asymptotic Significance (2-sided) |
| Pearson Chi-Square | 1.619^a^ | 3 | .655 |
| Likelihood Ratio | 1.570 | 3 | .666 |
| N of Valid Cases | 211 |  |  |
| a. 0 cells (0.0%) have expected count less than 5. The minimum expected count is 5.46. | | | |

**Country * Decision making structure: decision to adopt or not to adopt M-Health adoption is the prerogative of the hospital’s top management only.**

| **Crosstab** | | | | | | |
| --- | --- | --- | --- | --- | --- | --- |
| Count | | | | | | |
|  | | Decision making structure: decision to adopt or not to adopt M-Health adoption is the prerogative of the hospital’s top management only. | | | | Total |
|  |  | Strongly disgree | Disagree | Agree | Strongly Agree |  |
| Country | No | 18 | 30 | 6 | 7 | 61 |
|  | Yes | 40 | 46 | 38 | 25 | 149 |
|  | 2 | 0 | 1 | 0 | 0 | 1 |
| Total | | 58 | 77 | 44 | 32 | 211 |

| **Chi-Square Tests** | | | |
| --- | --- | --- | --- |
|  | Value | df | Asymptotic Significance (2-sided) |
| Pearson Chi-Square | 11.733^a^ | 6 | .068 |
| Likelihood Ratio | 12.623 | 6 | .049 |
| N of Valid Cases | 211 |  |  |
| a. 4 cells (33.3%) have expected count less than 5. The minimum expected count is .15. | | | |

**Eastern Africa * Decision making structure: decision to adopt or not to adopt M-Health adoption is the prerogative of the hospital’s top management only.**

| **Crosstab** | | | | | | |
| --- | --- | --- | --- | --- | --- | --- |
| Count | | | | | | |
|  | | Decision making structure: decision to adopt or not to adopt M-Health adoption is the prerogative of the hospital’s top management only. | | | | Total |
|  |  | Strongly disgree | Disagree | Agree | Strongly Agree |  |
| Eastern Africa | No | 39 | 49 | 26 | 18 | 132 |
|  | Yes | 19 | 28 | 18 | 14 | 79 |
| Total | | 58 | 77 | 44 | 32 | 211 |

| **Chi-Square Tests** | | | |
| --- | --- | --- | --- |
|  | Value | df | Asymptotic Significance (2-sided) |
| Pearson Chi-Square | 1.351^a^ | 3 | .717 |
| Likelihood Ratio | 1.348 | 3 | .718 |
| N of Valid Cases | 211 |  |  |
| a. 0 cells (0.0%) have expected count less than 5. The minimum expected count is 11.98. | | | |

| **Size: M-Health adoption is appropriate only when the hospital has substantial volume of patients and staff to justify its adoption * Category of hospital classification Crosstabulation** | | | | | |
| --- | --- | --- | --- | --- | --- |
| Count | | | | | |
|  | | Category of hospital classification | | | Total |
|  |  | Public Hospital | Private Hospital | Faith-Based Hospital/NGO |  |
| Size: M-Health adoption is appropriate only when the hospital has substantial volume of patients and staff to justify its adoption | Strongly disagree | 33 | 18 | 8 | 59 |
|  | Disagree | 42 | 29 | 12 | 83 |
|  | Agree | 20 | 19 | 10 | 49 |
|  | Strongly Agree | 7 | 10 | 3 | 20 |
| Total | | 102 | 76 | 33 | 211 |

| **Crosstab** | | | | | | |
| --- | --- | --- | --- | --- | --- | --- |
| Count | | | | | | |
|  | | Size: M-Health adoption is appropriate only when the hospital has substantial volume of patients and staff to justify its adoption | | | | Total |
|  |  | Strongly disgree | Disagree | Agree | Strongly Agree |  |
| Classification level of facility | Level IV | 48 | 65 | 41 | 16 | 170 |
|  | Level V | 10 | 12 | 7 | 4 | 33 |
|  | Level VI | 1 | 6 | 1 | 0 | 8 |
| Total | | 59 | 83 | 49 | 20 | 211 |

| **Chi-Square Tests** | | | |
| --- | --- | --- | --- |
|  | Value | df | Asymptotic Significance (2-sided) |
| Pearson Chi-Square | 4.981^a^ | 6 | .546 |
| Likelihood Ratio | 5.433 | 6 | .490 |
| Linear-by-Linear Association | .082 | 1 | .774 |
| N of Valid Cases | 211 |  |  |
| a. 5 cells (41.7%) have expected count less than 5. The minimum expected count is .76. | | | |

**Category of hospital classification * Size: M-Health adoption is appropriate only when the hospital has substantial volume of patients and staff to justify its adoption**

| **Crosstab** | | | | | | |
| --- | --- | --- | --- | --- | --- | --- |
| Count | | | | | | |
|  | | Size: M-Health adoption is appropriate only when the hospital has substantial volume of patients and staff to justify its adoption | | | | Total |
|  |  | Strongly disgree | Disagree | Agree | Strongly Agree |  |
| Category of hospital classification | Public Hospital | 33 | 42 | 20 | 7 | 102 |
|  | Private Hospital | 18 | 29 | 19 | 10 | 76 |
|  | Faith-Based Hospital/NGO | 8 | 12 | 10 | 3 | 33 |
| Total | | 59 | 83 | 49 | 20 | 211 |

| **Chi-Square Tests** | | | |
| --- | --- | --- | --- |
|  | Value | df | Asymptotic Significance (2-sided) |
| Pearson Chi-Square | 4.769^a^ | 6 | .574 |
| Likelihood Ratio | 4.699 | 6 | .583 |
| Linear-by-Linear Association | 2.873 | 1 | .090 |
| N of Valid Cases | 211 |  |  |
| a. 1 cells (8.3%) have expected count less than 5. The minimum expected count is 3.13. | | | |

**Geographical category of facility classification * Size: M-Health adoption is appropriate only when the hospital has substantial volume of patients and staff to justify its adoption**

| **Crosstab** | | | | | | |
| --- | --- | --- | --- | --- | --- | --- |
| Count | | | | | | |
|  | | Size: M-Health adoption is appropriate only when the hospital has substantial volume of patients and staff to justify its adoption | | | | Total |
|  |  | Strongly disgree | Disagree | Agree | Strongly Agree |  |
| Geographical category of facility classification | Urban | 17 | 31 | 22 | 7 | 77 |
|  | Semi-urban | 23 | 34 | 14 | 9 | 80 |
|  | Rural | 19 | 18 | 13 | 4 | 54 |
| Total | | 59 | 83 | 49 | 20 | 211 |

| **Chi-Square Tests** | | | |
| --- | --- | --- | --- |
|  | Value | df | Asymptotic Significance (2-sided) |
| Pearson Chi-Square | 5.309^a^ | 6 | .505 |
| Likelihood Ratio | 5.374 | 6 | .497 |
| N of Valid Cases | 211 |  |  |
| a. 0 cells (0.0%) have expected count less than 5. The minimum expected count is 5.12. | | | |

**Current annual number of patients (outpatients and in-patients) * Size: M-Health adoption is appropriate only when the hospital has substantial volume of patients and staff to justify its adoption**

| **Crosstab** | | | | | | |
| --- | --- | --- | --- | --- | --- | --- |
| Count | | | | | | |
|  | | Size: M-Health adoption is appropriate only when the hospital has substantial volume of patients and staff to justify its adoption | | | | Total |
|  |  | Strongly disgree | Disagree | Agree | Strongly Agree |  |
| Current annual number of patients (outpatients and in-patients) | Less than 200 | 14 | 18 | 14 | 3 | 49 |
|  | 200-500 | 18 | 18 | 12 | 8 | 56 |
|  | 501-1000 | 5 | 7 | 8 | 4 | 24 |
|  | 1001-1500 | 1 | 7 | 3 | 2 | 13 |
|  | 1501-2000 | 4 | 7 | 3 | 1 | 15 |
|  | 2001 and above | 17 | 23 | 8 | 2 | 50 |
| Total | | 59 | 80 | 48 | 20 | 207 |

| **Chi-Square Tests** | | | |
| --- | --- | --- | --- |
|  | Value | df | Asymptotic Significance (2-sided) |
| Pearson Chi-Square | 14.487^a^ | 15 | .489 |
| Likelihood Ratio | 15.374 | 15 | .425 |
| Linear-by-Linear Association | 1.742 | 1 | .187 |
| N of Valid Cases | 207 |  |  |
| a. 9 cells (37.5%) have expected count less than 5. The minimum expected count is 1.26. | | | |

**Number of staff * Size: M-Health adoption is appropriate only when the hospital has substantial volume of patients and staff to justify its adoption**

| **Crosstab** | | | | | | |
| --- | --- | --- | --- | --- | --- | --- |
| Count | | | | | | |
|  | | Size: M-Health adoption is appropriate only when the hospital has substantial volume of patients and staff to justify its adoption | | | | Total |
|  |  | Strongly disgree | Disagree | Agree | Strongly Agree |  |
| Number of staff | Less than 100 | 33 | 48 | 29 | 15 | 125 |
|  | 100-200 | 19 | 18 | 12 | 2 | 51 |
|  | 201-300 | 3 | 7 | 2 | 1 | 13 |
|  | 301-500 | 2 | 5 | 4 | 1 | 12 |
|  | 501 and above | 2 | 5 | 2 | 1 | 10 |
| Total | | 59 | 83 | 49 | 20 | 211 |

| **Chi-Square Tests** | | | |
| --- | --- | --- | --- |
|  | Value | df | Asymptotic Significance (2-sided) |
| Pearson Chi-Square | 7.293^a^ | 12 | .838 |
| Likelihood Ratio | 7.582 | 12 | .817 |
| Linear-by-Linear Association | .033 | 1 | .855 |
| N of Valid Cases | 211 |  |  |
| a. 12 cells (60.0%) have expected count less than 5. The minimum expected count is .95. | | | |

**County * Size: M-Health adoption is appropriate only when the hospital has substantial volume of patients and staff to justify its adoption**

| **Crosstab** | | | | | | |
| --- | --- | --- | --- | --- | --- | --- |
| Count | | | | | | |
|  | | Size: M-Health adoption is appropriate only when the hospital has substantial volume of patients and staff to justify its adoption | | | | Total |
|  |  | Strongly disgree | Disagree | Agree | Strongly Agree |  |
| County | No | 8 | 17 | 7 | 4 | 36 |
|  | Yes | 51 | 66 | 42 | 16 | 175 |
| Total | | 59 | 83 | 49 | 20 | 211 |

| **Chi-Square Tests** | | | |
| --- | --- | --- | --- |
|  | Value | df | Asymptotic Significance (2-sided) |
| Pearson Chi-Square | 1.586^a^ | 3 | .662 |
| Likelihood Ratio | 1.592 | 3 | .661 |
| N of Valid Cases | 211 |  |  |
| a. 1 cells (12.5%) have expected count less than 5. The minimum expected count is 3.41. | | | |

**Country * Size: M-Health adoption is appropriate only when the hospital has substantial volume of patients and staff to justify its adoption**

| **Crosstab** | | | | | | |
| --- | --- | --- | --- | --- | --- | --- |
| Count | | | | | | |
|  | | Size: M-Health adoption is appropriate only when the hospital has substantial volume of patients and staff to justify its adoption | | | | Total |
|  |  | Strongly disgree | Disagree | Agree | Strongly Agree |  |
| Country | No | 15 | 24 | 16 | 6 | 61 |
|  | Yes | 44 | 58 | 33 | 14 | 149 |
|  | 2 | 0 | 1 | 0 | 0 | 1 |
| Total | | 59 | 83 | 49 | 20 | 211 |

| **Chi-Square Tests** | | | |
| --- | --- | --- | --- |
|  | Value | df | Asymptotic Significance (2-sided) |
| Pearson Chi-Square | 2.249^a^ | 6 | .895 |
| Likelihood Ratio | 2.572 | 6 | .860 |
| N of Valid Cases | 211 |  |  |
| a. 4 cells (33.3%) have expected count less than 5. The minimum expected count is .09. | | | |

**Eastern Africa * Size: M-Health adoption is appropriate only when the hospital has substantial volume of patients and staff to justify its adoption**

| **Crosstab** | | | | | | |
| --- | --- | --- | --- | --- | --- | --- |
| Count | | | | | | |
|  | | Size: M-Health adoption is appropriate only when the hospital has substantial volume of patients and staff to justify its adoption | | | | Total |
|  |  | Strongly disgree | Disagree | Agree | Strongly Agree |  |
| Eastern Africa | No | 31 | 57 | 32 | 12 | 132 |
|  | Yes | 28 | 26 | 17 | 8 | 79 |
| Total | | 59 | 83 | 49 | 20 | 211 |

| **Chi-Square Tests** | | | |
| --- | --- | --- | --- |
|  | Value | df | Asymptotic Significance (2-sided) |
| Pearson Chi-Square | 4.066^a^ | 3 | .254 |
| Likelihood Ratio | 4.032 | 3 | .258 |
| N of Valid Cases | 211 |  |  |
| a. 0 cells (0.0%) have expected count less than 5. The minimum expected count is 7.49. | | | |

| **ICT capacity: M-Health adoption is appropriate when hospitals have a very complex ICT infrastructure * Category of hospital classification Crosstabulation** | | | | | |
| --- | --- | --- | --- | --- | --- |
| Count | | | | | |
|  | | Category of hospital classification | | | Total |
|  |  | Public Hospital | Private Hospital | Faith-Based Hospital/NGO |  |
| ICT capacity: M-Health adoption is appropriate when hospitals have a very complex ICT infrastructure | Strongly disagree | 30 | 21 | 14 | 65 |
|  | Disagree | 53 | 30 | 11 | 94 |
|  | Agree | 13 | 11 | 3 | 27 |
|  | Strongly Agree | 6 | 14 | 5 | 25 |
| Total | | 102 | 76 | 33 | 211 |

| **Crosstab** | | | | | | |
| --- | --- | --- | --- | --- | --- | --- |
| Count | | | | | | |
|  | | ICT capacity: M-Health adoption is appropriate when hospitals have a very complex ICT infrastructure | | | | Total |
|  |  | Strongly disgree | Disagree | Agree | Strongly Agree |  |
| Classification level of facility | Level IV | 52 | 75 | 24 | 19 | 170 |
|  | Level V | 9 | 16 | 2 | 6 | 33 |
|  | Level VI | 4 | 3 | 1 | 0 | 8 |
| Total | | 65 | 94 | 27 | 25 | 211 |

| **Chi-Square Tests** | | | |
| --- | --- | --- | --- |
|  | Value | df | Asymptotic Significance (2-sided) |
| Pearson Chi-Square | 4.837^a^ | 6 | .565 |
| Likelihood Ratio | 5.760 | 6 | .451 |
| Linear-by-Linear Association | .294 | 1 | .588 |
| N of Valid Cases | 211 |  |  |
| a. 6 cells (50.0%) have expected count less than 5. The minimum expected count is .95. | | | |

**Category of hospital classification * ICT capacity: M-Health adoption is appropriate when hospitals have a very complex ICT infrastructure**

| **Crosstab** | | | | | | |
| --- | --- | --- | --- | --- | --- | --- |
| Count | | | | | | |
|  | | ICT capacity: M-Health adoption is appropriate when hospitals have a very complex ICT infrastructure | | | | Total |
|  |  | Strongly disgree | Disagree | Agree | Strongly Agree |  |
| Category of hospital classification | Public Hospital | 30 | 53 | 13 | 6 | 102 |
|  | Private Hospital | 21 | 30 | 11 | 14 | 76 |
|  | Faith-Based Hospital/NGO | 14 | 11 | 3 | 5 | 33 |
| Total | | 65 | 94 | 27 | 25 | 211 |

| **Chi-Square Tests** | | | |
| --- | --- | --- | --- |
|  | Value | df | Asymptotic Significance (2-sided) |
| Pearson Chi-Square | 11.048^a^ | 6 | .087 |
| Likelihood Ratio | 11.255 | 6 | .081 |
| Linear-by-Linear Association | .613 | 1 | .434 |
| N of Valid Cases | 211 |  |  |
| a. 2 cells (16.7%) have expected count less than 5. The minimum expected count is 3.91. | | | |

**Geographical category of facility classification * ICT capacity: M-Health adoption is appropriate when hospitals have a very complex ICT infrastructure**

| **Crosstab** | | | | | | |
| --- | --- | --- | --- | --- | --- | --- |
| Count | | | | | | |
|  | | ICT capacity: M-Health adoption is appropriate when hospitals have a very complex ICT infrastructure | | | | Total |
|  |  | Strongly disgree | Disagree | Agree | Strongly Agree |  |
| Geographical category of facility classification | Urban | 24 | 30 | 13 | 10 | 77 |
|  | Semi-urban | 23 | 40 | 8 | 9 | 80 |
|  | Rural | 18 | 24 | 6 | 6 | 54 |
| Total | | 65 | 94 | 27 | 25 | 211 |

| **Chi-Square Tests** | | | |
| --- | --- | --- | --- |
|  | Value | df | Asymptotic Significance (2-sided) |
| Pearson Chi-Square | 3.045^a^ | 6 | .803 |
| Likelihood Ratio | 3.000 | 6 | .809 |
| N of Valid Cases | 211 |  |  |
| a. 0 cells (0.0%) have expected count less than 5. The minimum expected count is 6.40. | | | |

**Current annual number of patients (outpatients and in-patients) * ICT capacity: M-Health adoption is appropriate when hospitals have a very complex ICT infrastructure**

| **Crosstab** | | | | | | |
| --- | --- | --- | --- | --- | --- | --- |
| Count | | | | | | |
|  | | ICT capacity: M-Health adoption is appropriate when hospitals have a very complex ICT infrastructure | | | | Total |
|  |  | Strongly disgree | Disagree | Agree | Strongly Agree |  |
| Current annual number of patients (outpatients and in-patients) | Less than 200 | 17 | 21 | 6 | 5 | 49 |
|  | 200-500 | 16 | 18 | 10 | 12 | 56 |
|  | 501-1000 | 3 | 15 | 3 | 3 | 24 |
|  | 1001-1500 | 4 | 7 | 1 | 1 | 13 |
|  | 1501-2000 | 3 | 7 | 4 | 1 | 15 |
|  | 2001 and above | 22 | 23 | 2 | 3 | 50 |
| Total | | 65 | 91 | 26 | 25 | 207 |

| **Chi-Square Tests** | | | |
| --- | --- | --- | --- |
|  | Value | df | Asymptotic Significance (2-sided) |
| Pearson Chi-Square | 23.317^a^ | 15 | .078 |
| Likelihood Ratio | 23.955 | 15 | .066 |
| Linear-by-Linear Association | 4.274 | 1 | .039 |
| N of Valid Cases | 207 |  |  |
| a. 8 cells (33.3%) have expected count less than 5. The minimum expected count is 1.57. | | | |

**Number of staff * ICT capacity: M-Health adoption is appropriate when hospitals have a very complex ICT infrastructure**

| **Crosstab** | | | | | | |
| --- | --- | --- | --- | --- | --- | --- |
| Count | | | | | | |
|  | | ICT capacity: M-Health adoption is appropriate when hospitals have a very complex ICT infrastructure | | | | Total |
|  |  | Strongly disgree | Disagree | Agree | Strongly Agree |  |
| Number of staff | Less than 100 | 34 | 54 | 19 | 18 | 125 |
|  | 100-200 | 21 | 22 | 5 | 3 | 51 |
|  | 201-300 | 5 | 6 | 2 | 0 | 13 |
|  | 301-500 | 2 | 8 | 0 | 2 | 12 |
|  | 501 and above | 3 | 4 | 1 | 2 | 10 |
| Total | | 65 | 94 | 27 | 25 | 211 |

| **Chi-Square Tests** | | | |
| --- | --- | --- | --- |
|  | Value | df | Asymptotic Significance (2-sided) |
| Pearson Chi-Square | 11.923^a^ | 12 | .452 |
| Likelihood Ratio | 14.998 | 12 | .242 |
| Linear-by-Linear Association | .483 | 1 | .487 |
| N of Valid Cases | 211 |  |  |
| a. 10 cells (50.0%) have expected count less than 5. The minimum expected count is 1.18. | | | |

**County * ICT capacity: M-Health adoption is appropriate when hospitals have a very complex ICT infrastructure**

| **Crosstab** | | | | | | |
| --- | --- | --- | --- | --- | --- | --- |
| Count | | | | | | |
|  | | ICT capacity: M-Health adoption is appropriate when hospitals have a very complex ICT infrastructure | | | | Total |
|  |  | Strongly disgree | Disagree | Agree | Strongly Agree |  |
| County | No | 13 | 11 | 7 | 5 | 36 |
|  | Yes | 52 | 83 | 20 | 20 | 175 |
| Total | | 65 | 94 | 27 | 25 | 211 |

| **Chi-Square Tests** | | | |
| --- | --- | --- | --- |
|  | Value | df | Asymptotic Significance (2-sided) |
| Pearson Chi-Square | 3.956^a^ | 3 | .266 |
| Likelihood Ratio | 3.962 | 3 | .266 |
| N of Valid Cases | 211 |  |  |
| a. 2 cells (25.0%) have expected count less than 5. The minimum expected count is 4.27. | | | |

**Country * ICT capacity: M-Health adoption is appropriate when hospitals have a very complex ICT infrastructure**

| **Crosstab** | | | | | | |
| --- | --- | --- | --- | --- | --- | --- |
| Count | | | | | | |
|  | | ICT capacity: M-Health adoption is appropriate when hospitals have a very complex ICT infrastructure | | | | Total |
|  |  | Strongly disgree | Disagree | Agree | Strongly Agree |  |
| Country | No | 19 | 27 | 7 | 8 | 61 |
|  | Yes | 45 | 67 | 20 | 17 | 149 |
|  | 2 | 1 | 0 | 0 | 0 | 1 |
| Total | | 65 | 94 | 27 | 25 | 211 |

| **Chi-Square Tests** | | | |
| --- | --- | --- | --- |
|  | Value | df | Asymptotic Significance (2-sided) |
| Pearson Chi-Square | 2.509^a^ | 6 | .867 |
| Likelihood Ratio | 2.617 | 6 | .855 |
| N of Valid Cases | 211 |  |  |
| a. 4 cells (33.3%) have expected count less than 5. The minimum expected count is .12. | | | |

**Eastern Africa * ICT capacity: M-Health adoption is appropriate when hospitals have a very complex ICT infrastructure**

| **Crosstab** | | | | | | |
| --- | --- | --- | --- | --- | --- | --- |
| Count | | | | | | |
|  | | ICT capacity: M-Health adoption is appropriate when hospitals have a very complex ICT infrastructure | | | | Total |
|  |  | Strongly disgree | Disagree | Agree | Strongly Agree |  |
| Eastern Africa | No | 38 | 60 | 19 | 15 | 132 |
|  | Yes | 27 | 34 | 8 | 10 | 79 |
| Total | | 65 | 94 | 27 | 25 | 211 |

| **Chi-Square Tests** | | | |
| --- | --- | --- | --- |
|  | Value | df | Asymptotic Significance (2-sided) |
| Pearson Chi-Square | 1.304^a^ | 3 | .728 |
| Likelihood Ratio | 1.321 | 3 | .724 |
| N of Valid Cases | 211 |  |  |
| a. 0 cells (0.0%) have expected count less than 5. The minimum expected count is 9.36. | | | |

| **ICT staff : M-Health adoption is appropriate when hospitals have very knowledgeable and adequate number of staff in ICT * Category of hospital classification Crosstabulation** | | | | | |
| --- | --- | --- | --- | --- | --- |
| Count | | | | | |
|  | | Category of hospital classification | | | Total |
|  |  | Public Hospital | Private Hospital | Faith-Based Hospital/NGO |  |
| ICT staff : M-Health adoption is appropriate when hospitals have very knowledgeable and adequate number of staff in ICT | Strongly disagree | 13 | 13 | 1 | 27 |
|  | Disagree | 32 | 24 | 12 | 68 |
|  | Agree | 41 | 22 | 13 | 76 |
|  | Strongly Agree | 16 | 17 | 7 | 40 |
| Total | | 102 | 76 | 33 | 211 |

| **Crosstab** | | | | | | |
| --- | --- | --- | --- | --- | --- | --- |
| Count | | | | | | |
|  | | ICT staff : M-Health adoption is appropriate when hospitals have very knowledgeable and adequate number of staff in ICT | | | | Total |
|  |  | Strongly disgree | Disagree | Agree | Strongly Agree |  |
| Classification level of facility | Level IV | 22 | 55 | 61 | 32 | 170 |
|  | Level V | 5 | 10 | 11 | 7 | 33 |
|  | Level VI | 0 | 3 | 4 | 1 | 8 |
| Total | | 27 | 68 | 76 | 40 | 211 |

| **Chi-Square Tests** | | | |
| --- | --- | --- | --- |
|  | Value | df | Asymptotic Significance (2-sided) |
| Pearson Chi-Square | 2.044^a^ | 6 | .916 |
| Likelihood Ratio | 3.033 | 6 | .805 |
| Linear-by-Linear Association | .089 | 1 | .766 |
| N of Valid Cases | 211 |  |  |
| a. 5 cells (41.7%) have expected count less than 5. The minimum expected count is 1.02. | | | |

**Category of hospital classification * ICT staff : M-Health adoption is appropriate when hospitals have very knowledgeable and adequate number of staff in ICT**

| **Crosstab** | | | | | | |
| --- | --- | --- | --- | --- | --- | --- |
| Count | | | | | | |
|  | | ICT staff : M-Health adoption is appropriate when hospitals have very knowledgeable and adequate number of staff in ICT | | | | Total |
|  |  | Strongly disgree | Disagree | Agree | Strongly Agree |  |
| Category of hospital classification | Public Hospital | 13 | 32 | 41 | 16 | 102 |
|  | Private Hospital | 13 | 24 | 22 | 17 | 76 |
|  | Faith-Based Hospital/NGO | 1 | 12 | 13 | 7 | 33 |
| Total | | 27 | 68 | 76 | 40 | 211 |

| **Chi-Square Tests** | | | |
| --- | --- | --- | --- |
|  | Value | df | Asymptotic Significance (2-sided) |
| Pearson Chi-Square | 6.555^a^ | 6 | .364 |
| Likelihood Ratio | 7.610 | 6 | .268 |
| Linear-by-Linear Association | .680 | 1 | .410 |
| N of Valid Cases | 211 |  |  |
| a. 1 cells (8.3%) have expected count less than 5. The minimum expected count is 4.22. | | | |

**Geographical category of facility classification * ICT staff : M-Health adoption is appropriate when hospitals have very knowledgeable and adequate number of staff in ICT**

| **Crosstab** | | | | | | |
| --- | --- | --- | --- | --- | --- | --- |
| Count | | | | | | |
|  | | ICT staff : M-Health adoption is appropriate when hospitals have very knowledgeable and adequate number of staff in ICT | | | | Total |
|  |  | Strongly disgree | Disagree | Agree | Strongly Agree |  |
| Geographical category of facility classification | Urban | 11 | 28 | 22 | 16 | 77 |
|  | Semi-urban | 9 | 25 | 34 | 12 | 80 |
|  | Rural | 7 | 15 | 20 | 12 | 54 |
| Total | | 27 | 68 | 76 | 40 | 211 |

| **Chi-Square Tests** | | | |
| --- | --- | --- | --- |
|  | Value | df | Asymptotic Significance (2-sided) |
| Pearson Chi-Square | 4.282^a^ | 6 | .639 |
| Likelihood Ratio | 4.355 | 6 | .629 |
| N of Valid Cases | 211 |  |  |
| a. 0 cells (0.0%) have expected count less than 5. The minimum expected count is 6.91. | | | |

**Current annual number of patients (outpatients and in-patients) * ICT staff : M-Health adoption is appropriate when hospitals have very knowledgeable and adequate number of staff in ICT**

| **Crosstab** | | | | | | |
| --- | --- | --- | --- | --- | --- | --- |
| Count | | | | | | |
|  | | ICT staff : M-Health adoption is appropriate when hospitals have very knowledgeable and adequate number of staff in ICT | | | | Total |
|  |  | Strongly disgree | Disagree | Agree | Strongly Agree |  |
| Current annual number of patients (outpatients and in-patients) | Less than 200 | 9 | 19 | 15 | 6 | 49 |
|  | 200-500 | 7 | 12 | 18 | 19 | 56 |
|  | 501-1000 | 2 | 11 | 7 | 4 | 24 |
|  | 1001-1500 | 2 | 4 | 5 | 2 | 13 |
|  | 1501-2000 | 1 | 2 | 8 | 4 | 15 |
|  | 2001 and above | 6 | 18 | 21 | 5 | 50 |
| Total | | 27 | 66 | 74 | 40 | 207 |

| **Chi-Square Tests** | | | |
| --- | --- | --- | --- |
|  | Value | df | Asymptotic Significance (2-sided) |
| Pearson Chi-Square | 21.093^a^ | 15 | .134 |
| Likelihood Ratio | 20.953 | 15 | .138 |
| Linear-by-Linear Association | .014 | 1 | .906 |
| N of Valid Cases | 207 |  |  |
| a. 9 cells (37.5%) have expected count less than 5. The minimum expected count is 1.70. | | | |

**Number of staff * ICT staff : M-Health adoption is appropriate when hospitals have very knowledgeable and adequate number of staff in ICT**

| **Crosstab** | | | | | | |
| --- | --- | --- | --- | --- | --- | --- |
| Count | | | | | | |
|  | | ICT staff : M-Health adoption is appropriate when hospitals have very knowledgeable and adequate number of staff in ICT | | | | Total |
|  |  | Strongly disgree | Disagree | Agree | Strongly Agree |  |
| Number of staff | Less than 100 | 14 | 44 | 43 | 24 | 125 |
|  | 100-200 | 7 | 16 | 16 | 12 | 51 |
|  | 201-300 | 4 | 5 | 4 | 0 | 13 |
|  | 301-500 | 1 | 1 | 9 | 1 | 12 |
|  | 501 and above | 1 | 2 | 4 | 3 | 10 |
| Total | | 27 | 68 | 76 | 40 | 211 |

| **Chi-Square Tests** | | | |
| --- | --- | --- | --- |
|  | Value | df | Asymptotic Significance (2-sided) |
| Pearson Chi-Square | 16.905^a^ | 12 | .153 |
| Likelihood Ratio | 18.289 | 12 | .107 |
| Linear-by-Linear Association | .131 | 1 | .718 |
| N of Valid Cases | 211 |  |  |
| a. 12 cells (60.0%) have expected count less than 5. The minimum expected count is 1.28. | | | |

**County * ICT staff : M-Health adoption is appropriate when hospitals have very knowledgeable and adequate number of staff in ICT**

| **Crosstab** | | | | | | |
| --- | --- | --- | --- | --- | --- | --- |
| Count | | | | | | |
|  | | ICT staff : M-Health adoption is appropriate when hospitals have very knowledgeable and adequate number of staff in ICT | | | | Total |
|  |  | Strongly disgree | Disagree | Agree | Strongly Agree |  |
| County | No | 6 | 13 | 9 | 8 | 36 |
|  | Yes | 21 | 55 | 67 | 32 | 175 |
| Total | | 27 | 68 | 76 | 40 | 211 |

| **Chi-Square Tests** | | | |
| --- | --- | --- | --- |
|  | Value | df | Asymptotic Significance (2-sided) |
| Pearson Chi-Square | 2.419^a^ | 3 | .490 |
| Likelihood Ratio | 2.509 | 3 | .474 |
| N of Valid Cases | 211 |  |  |
| a. 1 cells (12.5%) have expected count less than 5. The minimum expected count is 4.61. | | | |

**Country * ICT staff : M-Health adoption is appropriate when hospitals have very knowledgeable and adequate number of staff in ICT**

| **Crosstab** | | | | | | |
| --- | --- | --- | --- | --- | --- | --- |
| Count | | | | | | |
|  | | ICT staff : M-Health adoption is appropriate when hospitals have very knowledgeable and adequate number of staff in ICT | | | | Total |
|  |  | Strongly disgree | Disagree | Agree | Strongly Agree |  |
| Country | No | 9 | 13 | 25 | 14 | 61 |
|  | Yes | 18 | 55 | 50 | 26 | 149 |
|  | 2 | 0 | 0 | 1 | 0 | 1 |
| Total | | 27 | 68 | 76 | 40 | 211 |

| **Chi-Square Tests** | | | |
| --- | --- | --- | --- |
|  | Value | df | Asymptotic Significance (2-sided) |
| Pearson Chi-Square | 6.649^a^ | 6 | .355 |
| Likelihood Ratio | 7.131 | 6 | .309 |
| N of Valid Cases | 211 |  |  |
| a. 4 cells (33.3%) have expected count less than 5. The minimum expected count is .13. | | | |

**Eastern Africa * ICT staff : M-Health adoption is appropriate when hospitals have very knowledgeable and adequate number of staff in ICT**

| **Crosstab** | | | | | | |
| --- | --- | --- | --- | --- | --- | --- |
| Count | | | | | | |
|  | | ICT staff : M-Health adoption is appropriate when hospitals have very knowledgeable and adequate number of staff in ICT | | | | Total |
|  |  | Strongly disgree | Disagree | Agree | Strongly Agree |  |
| Eastern Africa | No | 15 | 42 | 48 | 27 | 132 |
|  | Yes | 12 | 26 | 28 | 13 | 79 |
| Total | | 27 | 68 | 76 | 40 | 211 |

| **Chi-Square Tests** | | | |
| --- | --- | --- | --- |
|  | Value | df | Asymptotic Significance (2-sided) |
| Pearson Chi-Square | 1.012^a^ | 3 | .798 |
| Likelihood Ratio | 1.009 | 3 | .799 |
| N of Valid Cases | 211 |  |  |
| a. 0 cells (0.0%) have expected count less than 5. The minimum expected count is 10.11. | | | |

| **Scope of the Market: M-Health adoption is appropriate for hospitals with larger market scopes such as national, regional or global markets. * Category of hospital classification Crosstabulation** | | | | | |
| --- | --- | --- | --- | --- | --- |
| Count | | | | | |
|  | | Category of hospital classification | | | Total |
|  |  | Public Hospital | Private Hospital | Faith-Based Hospital/NGO |  |
| Scope of the Market: M-Health adoption is appropriate for hospitals with larger market scopes such as national, regional or global markets. | Strongly disagree | 30 | 21 | 5 | 56 |
|  | Disagree | 46 | 26 | 17 | 89 |
|  | Agree | 21 | 15 | 2 | 38 |
|  | Strongly Agree | 5 | 12 | 9 | 26 |
| Total | | 102 | 74 | 33 | 209 |

| **Crosstab** | | | | | | |
| --- | --- | --- | --- | --- | --- | --- |
| Count | | | | | | |
|  | | Scope of the Market: M-Health adoption is appropriate for hospitals with larger market scopes such as national, regional or global markets. | | | | Total |
|  |  | Strongly disgree | Disagree | Agree | Strongly Agree |  |
| Classification level of facility | Level IV | 48 | 74 | 28 | 19 | 169 |
|  | Level V | 6 | 12 | 9 | 5 | 32 |
|  | Level VI | 2 | 3 | 1 | 2 | 8 |
| Total | | 56 | 89 | 38 | 26 | 209 |

| **Chi-Square Tests** | | | |
| --- | --- | --- | --- |
|  | Value | df | Asymptotic Significance (2-sided) |
| Pearson Chi-Square | 4.840^a^ | 6 | .564 |
| Likelihood Ratio | 4.467 | 6 | .614 |
| Linear-by-Linear Association | 2.555 | 1 | .110 |
| N of Valid Cases | 209 |  |  |
| a. 5 cells (41.7%) have expected count less than 5. The minimum expected count is 1.00. | | | |

**Category of hospital classification * Scope of the Market: M-Health adoption is appropriate for hospitals with larger market scopes such as national, regional or global markets.**

| **Crosstab** | | | | | | |
| --- | --- | --- | --- | --- | --- | --- |
| Count | | | | | | |
|  | | Scope of the Market: M-Health adoption is appropriate for hospitals with larger market scopes such as national, regional or global markets. | | | | Total |
|  |  | Strongly disgree | Disagree | Agree | Strongly Agree |  |
| Category of hospital classification | Public Hospital | 30 | 46 | 21 | 5 | 102 |
|  | Private Hospital | 21 | 26 | 15 | 12 | 74 |
|  | Faith-Based Hospital/NGO | 5 | 17 | 2 | 9 | 33 |
| Total | | 56 | 89 | 38 | 26 | 209 |

| **Chi-Square Tests** | | | |
| --- | --- | --- | --- |
|  | Value | df | Asymptotic Significance (2-sided) |
| Pearson Chi-Square | 18.246^a^ | 6 | .006 |
| Likelihood Ratio | 19.324 | 6 | .004 |
| Linear-by-Linear Association | 6.128 | 1 | .013 |
| N of Valid Cases | 209 |  |  |
| a. 1 cells (8.3%) have expected count less than 5. The minimum expected count is 4.11. | | | |

**Geographical category of facility classification * Scope of the Market: M-Health adoption is appropriate for hospitals with larger market scopes such as national, regional or global markets.**

| **Crosstab** | | | | | | |
| --- | --- | --- | --- | --- | --- | --- |
| Count | | | | | | |
|  | | Scope of the Market: M-Health adoption is appropriate for hospitals with larger market scopes such as national, regional or global markets. | | | | Total |
|  |  | Strongly disgree | Disagree | Agree | Strongly Agree |  |
| Geographical category of facility classification | Urban | 17 | 29 | 17 | 12 | 75 |
|  | Semi-urban | 25 | 37 | 12 | 6 | 80 |
|  | Rural | 14 | 23 | 9 | 8 | 54 |
| Total | | 56 | 89 | 38 | 26 | 209 |

| **Chi-Square Tests** | | | |
| --- | --- | --- | --- |
|  | Value | df | Asymptotic Significance (2-sided) |
| Pearson Chi-Square | 5.529^a^ | 6 | .478 |
| Likelihood Ratio | 5.681 | 6 | .460 |
| N of Valid Cases | 209 |  |  |
| a. 0 cells (0.0%) have expected count less than 5. The minimum expected count is 6.72. | | | |

**Current annual number of patients (outpatients and in-patients) * Scope of the Market: M-Health adoption is appropriate for hospitals with larger market scopes such as national, regional or global markets.**

| **Crosstab** | | | | | | |
| --- | --- | --- | --- | --- | --- | --- |
| Count | | | | | | |
|  | | Scope of the Market: M-Health adoption is appropriate for hospitals with larger market scopes such as national, regional or global markets. | | | | Total |
|  |  | Strongly disgree | Disagree | Agree | Strongly Agree |  |
| Current annual number of patients (outpatients and in-patients) | Less than 200 | 13 | 24 | 7 | 4 | 48 |
|  | 200-500 | 14 | 20 | 11 | 11 | 56 |
|  | 501-1000 | 6 | 12 | 4 | 1 | 23 |
|  | 1001-1500 | 3 | 6 | 4 | 0 | 13 |
|  | 1501-2000 | 4 | 7 | 1 | 3 | 15 |
|  | 2001 and above | 16 | 17 | 10 | 7 | 50 |
| Total | | 56 | 86 | 37 | 26 | 205 |

| **Chi-Square Tests** | | | |
| --- | --- | --- | --- |
|  | Value | df | Asymptotic Significance (2-sided) |
| Pearson Chi-Square | 12.551^a^ | 15 | .637 |
| Likelihood Ratio | 14.460 | 15 | .491 |
| Linear-by-Linear Association | .003 | 1 | .958 |
| N of Valid Cases | 205 |  |  |
| a. 8 cells (33.3%) have expected count less than 5. The minimum expected count is 1.65. | | | |

**Number of staff * Scope of the Market: M-Health adoption is appropriate for hospitals with larger market scopes such as national, regional or global markets.**

| **Crosstab** | | | | | | |
| --- | --- | --- | --- | --- | --- | --- |
| Count | | | | | | |
|  | | Scope of the Market: M-Health adoption is appropriate for hospitals with larger market scopes such as national, regional or global markets. | | | | Total |
|  |  | Strongly disgree | Disagree | Agree | Strongly Agree |  |
| Number of staff | Less than 100 | 28 | 58 | 23 | 15 | 124 |
|  | 100-200 | 22 | 17 | 8 | 4 | 51 |
|  | 201-300 | 3 | 7 | 1 | 2 | 13 |
|  | 301-500 | 1 | 4 | 5 | 1 | 11 |
|  | 501 and above | 2 | 3 | 1 | 4 | 10 |
| Total | | 56 | 89 | 38 | 26 | 209 |

| **Chi-Square Tests** | | | |
| --- | --- | --- | --- |
|  | Value | df | Asymptotic Significance (2-sided) |
| Pearson Chi-Square | 22.839^a^ | 12 | .029 |
| Likelihood Ratio | 19.463 | 12 | .078 |
| Linear-by-Linear Association | 1.632 | 1 | .201 |
| N of Valid Cases | 209 |  |  |
| a. 11 cells (55.0%) have expected count less than 5. The minimum expected count is 1.24. | | | |

**County * Scope of the Market: M-Health adoption is appropriate for hospitals with larger market scopes such as national, regional or global markets.**

| **Crosstab** | | | | | | |
| --- | --- | --- | --- | --- | --- | --- |
| Count | | | | | | |
|  | | Scope of the Market: M-Health adoption is appropriate for hospitals with larger market scopes such as national, regional or global markets. | | | | Total |
|  |  | Strongly disgree | Disagree | Agree | Strongly Agree |  |
| County | No | 8 | 15 | 6 | 5 | 34 |
|  | Yes | 48 | 74 | 32 | 21 | 175 |
| Total | | 56 | 89 | 38 | 26 | 209 |

| **Chi-Square Tests** | | | |
| --- | --- | --- | --- |
|  | Value | df | Asymptotic Significance (2-sided) |
| Pearson Chi-Square | .358^a^ | 3 | .949 |
| Likelihood Ratio | .356 | 3 | .949 |
| N of Valid Cases | 209 |  |  |
| a. 1 cells (12.5%) have expected count less than 5. The minimum expected count is 4.23. | | | |

**Country * Scope of the Market: M-Health adoption is appropriate for hospitals with larger market scopes such as national, regional or global markets.**

| **Crosstab** | | | | | | |
| --- | --- | --- | --- | --- | --- | --- |
| Count | | | | | | |
|  | | Scope of the Market: M-Health adoption is appropriate for hospitals with larger market scopes such as national, regional or global markets. | | | | Total |
|  |  | Strongly disgree | Disagree | Agree | Strongly Agree |  |
| Country | No | 17 | 27 | 6 | 9 | 59 |
|  | Yes | 39 | 61 | 32 | 17 | 149 |
|  | 2 | 0 | 1 | 0 | 0 | 1 |
| Total | | 56 | 89 | 38 | 26 | 209 |

| **Chi-Square Tests** | | | |
| --- | --- | --- | --- |
|  | Value | df | Asymptotic Significance (2-sided) |
| Pearson Chi-Square | 5.170^a^ | 6 | .522 |
| Likelihood Ratio | 5.852 | 6 | .440 |
| N of Valid Cases | 209 |  |  |
| a. 4 cells (33.3%) have expected count less than 5. The minimum expected count is .12. | | | |

**Eastern Africa * Scope of the Market: M-Health adoption is appropriate for hospitals with larger market scopes such as national, regional or global markets.**

| **Crosstab** | | | | | | |
| --- | --- | --- | --- | --- | --- | --- |
| Count | | | | | | |
|  | | Scope of the Market: M-Health adoption is appropriate for hospitals with larger market scopes such as national, regional or global markets. | | | | Total |
|  |  | Strongly disgree | Disagree | Agree | Strongly Agree |  |
| Eastern Africa | No | 32 | 66 | 17 | 16 | 131 |
|  | Yes | 24 | 23 | 21 | 10 | 78 |
| Total | | 56 | 89 | 38 | 26 | 209 |

| **Chi-Square Tests** | | | |
| --- | --- | --- | --- |
|  | Value | df | Asymptotic Significance (2-sided) |
| Pearson Chi-Square | 10.990^a^ | 3 | .012 |
| Likelihood Ratio | 11.048 | 3 | .011 |
| N of Valid Cases | 209 |  |  |
| a. 0 cells (0.0%) have expected count less than 5. The minimum expected count is 9.70. | | | |

| **Slack/Financial Resources: M-health adoption is appropriate for hospitals that have excess budgets to invest in new IT technologies (soft and hard ware) such as M-Health * Category of hospital classification Crosstabulation** | | | | | |
| --- | --- | --- | --- | --- | --- |
| Count | | | | | |
|  | | Category of hospital classification | | | Total |
|  |  | Public Hospital | Private Hospital | Faith-Based Hospital/NGO |  |
| Slack/Financial Resources: M-health adoption is appropriate for hospitals that have excess budgets to invest in new IT technologies (soft and hard ware) such as M-Health | Strongly disagree | 36 | 28 | 12 | 76 |
|  | Disagree | 41 | 23 | 12 | 76 |
|  | Agree | 19 | 18 | 7 | 44 |
|  | Strongly Agree | 6 | 7 | 2 | 15 |
| Total | | 102 | 76 | 33 | 211 |

| **Crosstab** | | | | | | |
| --- | --- | --- | --- | --- | --- | --- |
| Count | | | | | | |
|  | | Slack/Financial Resources: M-health adoption is appropriate for hospitals that have excess budgets to invest in new IT technologies (soft and hard ware) such as M-Health | | | | Total |
|  |  | Strongly disgree | Disagree | Agree | Strongly Agree |  |
| Classification level of facility | Level IV | 59 | 65 | 34 | 12 | 170 |
|  | Level V | 12 | 8 | 10 | 3 | 33 |
|  | Level VI | 5 | 3 | 0 | 0 | 8 |
| Total | | 76 | 76 | 44 | 15 | 211 |

| **Chi-Square Tests** | | | |
| --- | --- | --- | --- |
|  | Value | df | Asymptotic Significance (2-sided) |
| Pearson Chi-Square | 7.040^a^ | 6 | .317 |
| Likelihood Ratio | 8.967 | 6 | .175 |
| Linear-by-Linear Association | .667 | 1 | .414 |
| N of Valid Cases | 211 |  |  |
| a. 5 cells (41.7%) have expected count less than 5. The minimum expected count is .57. | | | |

**Category of hospital classification * Slack/Financial Resources: M-health adoption is appropriate for hospitals that have excess budgets to invest in new IT technologies (soft and hard ware) such as M-Health**

| **Crosstab** | | | | | | |
| --- | --- | --- | --- | --- | --- | --- |
| Count | | | | | | |
|  | | Slack/Financial Resources: M-health adoption is appropriate for hospitals that have excess budgets to invest in new IT technologies (soft and hard ware) such as M-Health | | | | Total |
|  |  | Strongly disgree | Disagree | Agree | Strongly Agree |  |
| Category of hospital classification | Public Hospital | 36 | 41 | 19 | 6 | 102 |
|  | Private Hospital | 28 | 23 | 18 | 7 | 76 |
|  | Faith-Based Hospital/NGO | 12 | 12 | 7 | 2 | 33 |
| Total | | 76 | 76 | 44 | 15 | 211 |

| **Chi-Square Tests** | | | |
| --- | --- | --- | --- |
|  | Value | df | Asymptotic Significance (2-sided) |
| Pearson Chi-Square | 2.500^a^ | 6 | .868 |
| Likelihood Ratio | 2.495 | 6 | .869 |
| Linear-by-Linear Association | .116 | 1 | .733 |
| N of Valid Cases | 211 |  |  |
| a. 1 cells (8.3%) have expected count less than 5. The minimum expected count is 2.35. | | | |

**Geographical category of facility classification * Slack/Financial Resources: M-health adoption is appropriate for hospitals that have excess budgets to invest in new IT technologies (soft and hard ware) such as M-Health**

| **Crosstab** | | | | | | |
| --- | --- | --- | --- | --- | --- | --- |
| Count | | | | | | |
|  | | Slack/Financial Resources: M-health adoption is appropriate for hospitals that have excess budgets to invest in new IT technologies (soft and hard ware) such as M-Health | | | | Total |
|  |  | Strongly disgree | Disagree | Agree | Strongly Agree |  |
| Geographical category of facility classification | Urban | 24 | 30 | 13 | 10 | 77 |
|  | Semi-urban | 34 | 27 | 16 | 3 | 80 |
|  | Rural | 18 | 19 | 15 | 2 | 54 |
| Total | | 76 | 76 | 44 | 15 | 211 |

| **Chi-Square Tests** | | | |
| --- | --- | --- | --- |
|  | Value | df | Asymptotic Significance (2-sided) |
| Pearson Chi-Square | 9.598^a^ | 6 | .143 |
| Likelihood Ratio | 9.208 | 6 | .162 |
| N of Valid Cases | 211 |  |  |
| a. 1 cells (8.3%) have expected count less than 5. The minimum expected count is 3.84. | | | |

**Current annual number of patients (outpatients and in-patients) * Slack/Financial Resources: M-health adoption is appropriate for hospitals that have excess budgets to invest in new IT technologies (soft and hard ware) such as M-Health**

| **Crosstab** | | | | | | |
| --- | --- | --- | --- | --- | --- | --- |
| Count | | | | | | |
|  | | Slack/Financial Resources: M-health adoption is appropriate for hospitals that have excess budgets to invest in new IT technologies (soft and hard ware) such as M-Health | | | | Total |
|  |  | Strongly disgree | Disagree | Agree | Strongly Agree |  |
| Current annual number of patients (outpatients and in-patients) | Less than 200 | 19 | 21 | 6 | 3 | 49 |
|  | 200-500 | 17 | 17 | 17 | 5 | 56 |
|  | 501-1000 | 5 | 11 | 6 | 2 | 24 |
|  | 1001-1500 | 5 | 4 | 3 | 1 | 13 |
|  | 1501-2000 | 3 | 8 | 3 | 1 | 15 |
|  | 2001 and above | 27 | 13 | 7 | 3 | 50 |
| Total | | 76 | 74 | 42 | 15 | 207 |

| **Chi-Square Tests** | | | |
| --- | --- | --- | --- |
|  | Value | df | Asymptotic Significance (2-sided) |
| Pearson Chi-Square | 18.200^a^ | 15 | .252 |
| Likelihood Ratio | 18.134 | 15 | .256 |
| Linear-by-Linear Association | 1.667 | 1 | .197 |
| N of Valid Cases | 207 |  |  |
| a. 11 cells (45.8%) have expected count less than 5. The minimum expected count is .94. | | | |

**Number of staff * Slack/Financial Resources: M-health adoption is appropriate for hospitals that have excess budgets to invest in new IT technologies (soft and hard ware) such as M-Health**

| **Crosstab** | | | | | | |
| --- | --- | --- | --- | --- | --- | --- |
| Count | | | | | | |
|  | | Slack/Financial Resources: M-health adoption is appropriate for hospitals that have excess budgets to invest in new IT technologies (soft and hard ware) such as M-Health | | | | Total |
|  |  | Strongly disgree | Disagree | Agree | Strongly Agree |  |
| Number of staff | Less than 100 | 39 | 43 | 32 | 11 | 125 |
|  | 100-200 | 25 | 17 | 8 | 1 | 51 |
|  | 201-300 | 6 | 7 | 0 | 0 | 13 |
|  | 301-500 | 2 | 6 | 2 | 2 | 12 |
|  | 501 and above | 4 | 3 | 2 | 1 | 10 |
| Total | | 76 | 76 | 44 | 15 | 211 |

| **Chi-Square Tests** | | | |
| --- | --- | --- | --- |
|  | Value | df | Asymptotic Significance (2-sided) |
| Pearson Chi-Square | 16.760^a^ | 12 | .159 |
| Likelihood Ratio | 20.531 | 12 | .058 |
| Linear-by-Linear Association | .824 | 1 | .364 |
| N of Valid Cases | 211 |  |  |
| a. 13 cells (65.0%) have expected count less than 5. The minimum expected count is .71. | | | |

**County * Slack/Financial Resources: M-health adoption is appropriate for hospitals that have excess budgets to invest in new IT technologies (soft and hard ware) such as M-Health**

| **Crosstab** | | | | | | |
| --- | --- | --- | --- | --- | --- | --- |
| Count | | | | | | |
|  | | Slack/Financial Resources: M-health adoption is appropriate for hospitals that have excess budgets to invest in new IT technologies (soft and hard ware) such as M-Health | | | | Total |
|  |  | Strongly disgree | Disagree | Agree | Strongly Agree |  |
| County | No | 10 | 18 | 5 | 3 | 36 |
|  | Yes | 66 | 58 | 39 | 12 | 175 |
| Total | | 76 | 76 | 44 | 15 | 211 |

| **Chi-Square Tests** | | | |
| --- | --- | --- | --- |
|  | Value | df | Asymptotic Significance (2-sided) |
| Pearson Chi-Square | 4.275^a^ | 3 | .233 |
| Likelihood Ratio | 4.235 | 3 | .237 |
| N of Valid Cases | 211 |  |  |
| a. 1 cells (12.5%) have expected count less than 5. The minimum expected count is 2.56. | | | |

**Country * Slack/Financial Resources: M-health adoption is appropriate for hospitals that have excess budgets to invest in new IT technologies (soft and hard ware) such as M-Health**

| **Crosstab** | | | | | | |
| --- | --- | --- | --- | --- | --- | --- |
| Count | | | | | | |
|  | | Slack/Financial Resources: M-health adoption is appropriate for hospitals that have excess budgets to invest in new IT technologies (soft and hard ware) such as M-Health | | | | Total |
|  |  | Strongly disgree | Disagree | Agree | Strongly Agree |  |
| Country | No | 22 | 22 | 12 | 5 | 61 |
|  | Yes | 54 | 53 | 32 | 10 | 149 |
|  | 2 | 0 | 1 | 0 | 0 | 1 |
| Total | | 76 | 76 | 44 | 15 | 211 |

| **Chi-Square Tests** | | | |
| --- | --- | --- | --- |
|  | Value | df | Asymptotic Significance (2-sided) |
| Pearson Chi-Square | 1.990^a^ | 6 | .921 |
| Likelihood Ratio | 2.252 | 6 | .895 |
| N of Valid Cases | 211 |  |  |
| a. 5 cells (41.7%) have expected count less than 5. The minimum expected count is .07. | | | |

**Eastern Africa * Slack/Financial Resources: M-health adoption is appropriate for hospitals that have excess budgets to invest in new IT technologies (soft and hard ware) such as M-Health**

| **Crosstab** | | | | | | |
| --- | --- | --- | --- | --- | --- | --- |
| Count | | | | | | |
|  | | Slack/Financial Resources: M-health adoption is appropriate for hospitals that have excess budgets to invest in new IT technologies (soft and hard ware) such as M-Health | | | | Total |
|  |  | Strongly disgree | Disagree | Agree | Strongly Agree |  |
| Eastern Africa | No | 48 | 48 | 26 | 10 | 132 |
|  | Yes | 28 | 28 | 18 | 5 | 79 |
| Total | | 76 | 76 | 44 | 15 | 211 |

| **Chi-Square Tests** | | | |
| --- | --- | --- | --- |
|  | Value | df | Asymptotic Significance (2-sided) |
| Pearson Chi-Square | .357^a^ | 3 | .949 |
| Likelihood Ratio | .357 | 3 | .949 |
| N of Valid Cases | 211 |  |  |
| a. 0 cells (0.0%) have expected count less than 5. The minimum expected count is 5.62. | | | |

| **Technology leadership: M-Health is appropriate for hospitals that pursue market growth through technology leadership * Category of hospital classification Crosstabulation** | | | | | |
| --- | --- | --- | --- | --- | --- |
| Count | | | | | |
|  | | Category of hospital classification | | | Total |
|  |  | Public Hospital | Private Hospital | Faith-Based Hospital/NGO |  |
| Technology leadership: M-Health is appropriate for hospitals that pursue market growth through technology leadership | Strongly disgree | 15 | 11 | 4 | 30 |
|  | Disagree | 29 | 13 | 7 | 49 |
|  | Agree | 41 | 31 | 6 | 78 |
|  | Strongly Agree | 17 | 21 | 16 | 54 |
| Total | | 102 | 76 | 33 | 211 |

| **Crosstab** | | | | | | |
| --- | --- | --- | --- | --- | --- | --- |
| Count | | | | | | |
|  | | Technology leadership: M-Health is appropriate for hospitals that pursue market growth through technology leadership | | | | Total |
|  |  | Strongly disgree | Disagree | Agree | Strongly Agree |  |
| Classification level of facility | Level IV | 25 | 40 | 63 | 42 | 170 |
|  | Level V | 5 | 6 | 14 | 8 | 33 |
|  | Level VI | 0 | 3 | 1 | 4 | 8 |
| Total | | 30 | 49 | 78 | 54 | 211 |

| **Chi-Square Tests** | | | |
| --- | --- | --- | --- |
|  | Value | df | Asymptotic Significance (2-sided) |
| Pearson Chi-Square | 5.756^a^ | 6 | .451 |
| Likelihood Ratio | 6.841 | 6 | .336 |
| Linear-by-Linear Association | .861 | 1 | .353 |
| N of Valid Cases | 211 |  |  |
| a. 5 cells (41.7%) have expected count less than 5. The minimum expected count is 1.14. | | | |

**Category of hospital classification * Technology leadership: M-Health is appropriate for hospitals that pursue market growth through technology leadership**

| **Crosstab** | | | | | | |
| --- | --- | --- | --- | --- | --- | --- |
| Count | | | | | | |
|  | | Technology leadership: M-Health is appropriate for hospitals that pursue market growth through technology leadership | | | | Total |
|  |  | Strongly disgree | Disagree | Agree | Strongly Agree |  |
| Category of hospital classification | Public Hospital | 15 | 29 | 41 | 17 | 102 |
|  | Private Hospital | 11 | 13 | 31 | 21 | 76 |
|  | Faith-Based Hospital/NGO | 4 | 7 | 6 | 16 | 33 |
| Total | | 30 | 49 | 78 | 54 | 211 |

| **Chi-Square Tests** | | | |
| --- | --- | --- | --- |
|  | Value | df | Asymptotic Significance (2-sided) |
| Pearson Chi-Square | 16.391^a^ | 6 | .012 |
| Likelihood Ratio | 16.185 | 6 | .013 |
| Linear-by-Linear Association | 5.601 | 1 | .018 |
| N of Valid Cases | 211 |  |  |
| a. 1 cells (8.3%) have expected count less than 5. The minimum expected count is 4.69. | | | |

**Geographical category of facility classification * Technology leadership: M-Health is appropriate for hospitals that pursue market growth through technology leadership**

| **Crosstab** | | | | | | |
| --- | --- | --- | --- | --- | --- | --- |
| Count | | | | | | |
|  | | Technology leadership: M-Health is appropriate for hospitals that pursue market growth through technology leadership | | | | Total |
|  |  | Strongly disgree | Disagree | Agree | Strongly Agree |  |
| Geographical category of facility classification | Urban | 10 | 15 | 31 | 21 | 77 |
|  | Semi-urban | 13 | 19 | 30 | 18 | 80 |
|  | Rural | 7 | 15 | 17 | 15 | 54 |
| Total | | 30 | 49 | 78 | 54 | 211 |

| **Chi-Square Tests** | | | |
| --- | --- | --- | --- |
|  | Value | df | Asymptotic Significance (2-sided) |
| Pearson Chi-Square | 2.487^a^ | 6 | .870 |
| Likelihood Ratio | 2.504 | 6 | .868 |
| N of Valid Cases | 211 |  |  |
| a. 0 cells (0.0%) have expected count less than 5. The minimum expected count is 7.68. | | | |

**Current annual number of patients (outpatients and in-patients) * Technology leadership: M-Health is appropriate for hospitals that pursue market growth through technology leadership**

| **Crosstab** | | | | | | |
| --- | --- | --- | --- | --- | --- | --- |
| Count | | | | | | |
|  | | Technology leadership: M-Health is appropriate for hospitals that pursue market growth through technology leadership | | | | Total |
|  |  | Strongly disgree | Disagree | Agree | Strongly Agree |  |
| Current annual number of patients (outpatients and in-patients) | Less than 200 | 8 | 13 | 19 | 9 | 49 |
|  | 200-500 | 8 | 7 | 22 | 19 | 56 |
|  | 501-1000 | 2 | 5 | 8 | 9 | 24 |
|  | 1001-1500 | 4 | 4 | 3 | 2 | 13 |
|  | 1501-2000 | 1 | 4 | 7 | 3 | 15 |
|  | 2001 and above | 6 | 16 | 16 | 12 | 50 |
| Total | | 29 | 49 | 75 | 54 | 207 |

| **Chi-Square Tests** | | | |
| --- | --- | --- | --- |
|  | Value | df | Asymptotic Significance (2-sided) |
| Pearson Chi-Square | 15.184^a^ | 15 | .438 |
| Likelihood Ratio | 15.243 | 15 | .434 |
| Linear-by-Linear Association | .131 | 1 | .717 |
| N of Valid Cases | 207 |  |  |
| a. 8 cells (33.3%) have expected count less than 5. The minimum expected count is 1.82. | | | |

**Number of staff * Technology leadership: M-Health is appropriate for hospitals that pursue market growth through technology leadership**

| **Crosstab** | | | | | | |
| --- | --- | --- | --- | --- | --- | --- |
| Count | | | | | | |
|  | | Technology leadership: M-Health is appropriate for hospitals that pursue market growth through technology leadership | | | | Total |
|  |  | Strongly disgree | Disagree | Agree | Strongly Agree |  |
| Number of staff | Less than 100 | 17 | 25 | 51 | 32 | 125 |
|  | 100-200 | 8 | 17 | 15 | 11 | 51 |
|  | 201-300 | 3 | 3 | 4 | 3 | 13 |
|  | 301-500 | 0 | 3 | 6 | 3 | 12 |
|  | 501 and above | 2 | 1 | 2 | 5 | 10 |
| Total | | 30 | 49 | 78 | 54 | 211 |

| **Chi-Square Tests** | | | |
| --- | --- | --- | --- |
|  | Value | df | Asymptotic Significance (2-sided) |
| Pearson Chi-Square | 11.777^a^ | 12 | .464 |
| Likelihood Ratio | 12.966 | 12 | .372 |
| Linear-by-Linear Association | .134 | 1 | .714 |
| N of Valid Cases | 211 |  |  |
| a. 12 cells (60.0%) have expected count less than 5. The minimum expected count is 1.42. | | | |

**County * Technology leadership: M-Health is appropriate for hospitals that pursue market growth through technology leadership**

| **Crosstab** | | | | | | |
| --- | --- | --- | --- | --- | --- | --- |
| Count | | | | | | |
|  | | Technology leadership: M-Health is appropriate for hospitals that pursue market growth through technology leadership | | | | Total |
|  |  | Strongly disgree | Disagree | Agree | Strongly Agree |  |
| County | No | 8 | 8 | 12 | 8 | 36 |
|  | Yes | 22 | 41 | 66 | 46 | 175 |
| Total | | 30 | 49 | 78 | 54 | 211 |

| **Chi-Square Tests** | | | |
| --- | --- | --- | --- |
|  | Value | df | Asymptotic Significance (2-sided) |
| Pearson Chi-Square | 2.322^a^ | 3 | .508 |
| Likelihood Ratio | 2.108 | 3 | .550 |
| N of Valid Cases | 211 |  |  |
| a. 0 cells (0.0%) have expected count less than 5. The minimum expected count is 5.12. | | | |

**Country * Technology leadership: M-Health is appropriate for hospitals that pursue market growth through technology leadership**

| **Crosstab** | | | | | | |
| --- | --- | --- | --- | --- | --- | --- |
| Count | | | | | | |
|  | | Technology leadership: M-Health is appropriate for hospitals that pursue market growth through technology leadership | | | | Total |
|  |  | Strongly disgree | Disagree | Agree | Strongly Agree |  |
| Country | No | 10 | 13 | 22 | 16 | 61 |
|  | Yes | 20 | 36 | 56 | 37 | 149 |
|  | 2 | 0 | 0 | 0 | 1 | 1 |
| Total | | 30 | 49 | 78 | 54 | 211 |

| **Chi-Square Tests** | | | |
| --- | --- | --- | --- |
|  | Value | df | Asymptotic Significance (2-sided) |
| Pearson Chi-Square | 3.401^a^ | 6 | .757 |
| Likelihood Ratio | 3.213 | 6 | .782 |
| N of Valid Cases | 211 |  |  |
| a. 4 cells (33.3%) have expected count less than 5. The minimum expected count is .14. | | | |

**Eastern Africa * Technology leadership: M-Health is appropriate for hospitals that pursue market growth through technology leadership**

| **Crosstab** | | | | | | |
| --- | --- | --- | --- | --- | --- | --- |
| Count | | | | | | |
|  | | Technology leadership: M-Health is appropriate for hospitals that pursue market growth through technology leadership | | | | Total |
|  |  | Strongly disgree | Disagree | Agree | Strongly Agree |  |
| Eastern Africa | No | 19 | 34 | 48 | 31 | 132 |
|  | Yes | 11 | 15 | 30 | 23 | 79 |
| Total | | 30 | 49 | 78 | 54 | 211 |

| **Chi-Square Tests** | | | |
| --- | --- | --- | --- |
|  | Value | df | Asymptotic Significance (2-sided) |
| Pearson Chi-Square | 1.630^a^ | 3 | .653 |
| Likelihood Ratio | 1.648 | 3 | .649 |
| N of Valid Cases | 211 |  |  |
| a. 0 cells (0.0%) have expected count less than 5. The minimum expected count is 11.23. | | | |

INDUSTRY’S ENVIRONMENT

1. **Rate the extent to which you agree with the following statements relating the effect of the industry’s environment determinants listed below on adoption of M-Health using 1 = Strongly Disagree, 2=Disagree, 3=Agree and 4=Strongly Agree.**

| **Industry competition: Decision to adopt M-Health is appropriate when the hospital is facing high level of competition for patients. * Category of hospital classification Crosstabulation** | | | | | |
| --- | --- | --- | --- | --- | --- |
| Count | | | | | |
|  | | Category of hospital classification | | | Total |
|  |  | Public Hospital | Private Hospital | Faith-Based Hospital/NGO |  |
| Industry competition: Decision to adopt M-Health is appropriate when the hospital is facing high level of competition for patients. | Strongly disagree | 20 | 14 | 5 | 39 |
|  | Disagree | 44 | 29 | 11 | 84 |
|  | Agree | 33 | 22 | 6 | 61 |
|  | Strongly Agree | 5 | 11 | 11 | 27 |
| Total | | 102 | 76 | 33 | 211 |

| **Crosstab** | | | | | | |
| --- | --- | --- | --- | --- | --- | --- |
| Count | | | | | | |
|  | | Industry competition: Decision to adopt M-Health is appropriate when the hospital is facing high level of competition for patients. | | | | Total |
|  |  | Strongly disgree | Disagree | Agree | Strongly Agree |  |
| Classification level of facility | Level IV | 33 | 71 | 44 | 22 | 170 |
|  | Level V | 5 | 10 | 15 | 3 | 33 |
|  | Level VI | 1 | 3 | 2 | 2 | 8 |
| Total | | 39 | 84 | 61 | 27 | 211 |

| **Chi-Square Tests** | | | |
| --- | --- | --- | --- |
|  | Value | df | Asymptotic Significance (2-sided) |
| Pearson Chi-Square | 6.349^a^ | 6 | .385 |
| Likelihood Ratio | 5.836 | 6 | .442 |
| Linear-by-Linear Association | 1.512 | 1 | .219 |
| N of Valid Cases | 211 |  |  |
| a. 5 cells (41.7%) have expected count less than 5. The minimum expected count is 1.02. | | | |

**Category of hospital classification * Industry competition: Decision to adopt M-Health is appropriate when the hospital is facing high level of competition for patients.**

| **Crosstab** | | | | | | |
| --- | --- | --- | --- | --- | --- | --- |
| Count | | | | | | |
|  | | Industry competition: Decision to adopt M-Health is appropriate when the hospital is facing high level of competition for patients. | | | | Total |
|  |  | Strongly disgree | Disagree | Agree | Strongly Agree |  |
| Category of hospital classification | Public Hospital | 20 | 44 | 33 | 5 | 102 |
|  | Private Hospital | 14 | 29 | 22 | 11 | 76 |
|  | Faith-Based Hospital/NGO | 5 | 11 | 6 | 11 | 33 |
| Total | | 39 | 84 | 61 | 27 | 211 |

| **Chi-Square Tests** | | | |
| --- | --- | --- | --- |
|  | Value | df | Asymptotic Significance (2-sided) |
| Pearson Chi-Square | 18.695^a^ | 6 | .005 |
| Likelihood Ratio | 17.073 | 6 | .009 |
| Linear-by-Linear Association | 6.387 | 1 | .011 |
| N of Valid Cases | 211 |  |  |
| a. 1 cells (8.3%) have expected count less than 5. The minimum expected count is 4.22. | | | |

**Geographical category of facility classification * Industry competition: Decision to adopt M-Health is appropriate when the hospital is facing high level of competition for patients.**

| **Crosstab** | | | | | | |
| --- | --- | --- | --- | --- | --- | --- |
| Count | | | | | | |
|  | | Industry competition: Decision to adopt M-Health is appropriate when the hospital is facing high level of competition for patients. | | | | Total |
|  |  | Strongly disgree | Disagree | Agree | Strongly Agree |  |
| Geographical category of facility classification | Urban | 13 | 29 | 27 | 8 | 77 |
|  | Semi-urban | 16 | 32 | 22 | 10 | 80 |
|  | Rural | 10 | 23 | 12 | 9 | 54 |
| Total | | 39 | 84 | 61 | 27 | 211 |

| **Chi-Square Tests** | | | |
| --- | --- | --- | --- |
|  | Value | df | Asymptotic Significance (2-sided) |
| Pearson Chi-Square | 3.287^a^ | 6 | .772 |
| Likelihood Ratio | 3.265 | 6 | .775 |
| N of Valid Cases | 211 |  |  |
| a. 0 cells (0.0%) have expected count less than 5. The minimum expected count is 6.91. | | | |

**Current annual number of patients (outpatients and in-patients) * Industry competition: Decision to adopt M-Health is appropriate when the hospital is facing high level of competition for patients.**

| **Crosstab** | | | | | | |
| --- | --- | --- | --- | --- | --- | --- |
| Count | | | | | | |
|  | | Industry competition: Decision to adopt M-Health is appropriate when the hospital is facing high level of competition for patients. | | | | Total |
|  |  | Strongly disgree | Disagree | Agree | Strongly Agree |  |
| Current annual number of patients (outpatients and in-patients) | Less than 200 | 12 | 19 | 11 | 7 | 49 |
|  | 200-500 | 10 | 24 | 13 | 9 | 56 |
|  | 501-1000 | 3 | 11 | 7 | 3 | 24 |
|  | 1001-1500 | 4 | 5 | 3 | 1 | 13 |
|  | 1501-2000 | 3 | 7 | 3 | 2 | 15 |
|  | 2001 and above | 7 | 14 | 24 | 5 | 50 |
| Total | | 39 | 80 | 61 | 27 | 207 |

| **Chi-Square Tests** | | | |
| --- | --- | --- | --- |
|  | Value | df | Asymptotic Significance (2-sided) |
| Pearson Chi-Square | 14.405^a^ | 15 | .495 |
| Likelihood Ratio | 13.737 | 15 | .546 |
| Linear-by-Linear Association | 1.226 | 1 | .268 |
| N of Valid Cases | 207 |  |  |
| a. 8 cells (33.3%) have expected count less than 5. The minimum expected count is 1.70. | | | |

**Number of staff * Industry competition: Decision to adopt M-Health is appropriate when the hospital is facing high level of competition for patients.**

| **Crosstab** | | | | | | |
| --- | --- | --- | --- | --- | --- | --- |
| Count | | | | | | |
|  | | Industry competition: Decision to adopt M-Health is appropriate when the hospital is facing high level of competition for patients. | | | | Total |
|  |  | Strongly disgree | Disagree | Agree | Strongly Agree |  |
| Number of staff | Less than 100 | 25 | 53 | 31 | 16 | 125 |
|  | 100-200 | 11 | 18 | 18 | 4 | 51 |
|  | 201-300 | 2 | 7 | 3 | 1 | 13 |
|  | 301-500 | 0 | 5 | 6 | 1 | 12 |
|  | 501 and above | 1 | 1 | 3 | 5 | 10 |
| Total | | 39 | 84 | 61 | 27 | 211 |

| **Chi-Square Tests** | | | |
| --- | --- | --- | --- |
|  | Value | df | Asymptotic Significance (2-sided) |
| Pearson Chi-Square | 22.149^a^ | 12 | .036 |
| Likelihood Ratio | 20.609 | 12 | .056 |
| Linear-by-Linear Association | 6.419 | 1 | .011 |
| N of Valid Cases | 211 |  |  |
| a. 11 cells (55.0%) have expected count less than 5. The minimum expected count is 1.28. | | | |

**County * Industry competition: Decision to adopt M-Health is appropriate when the hospital is facing high level of competition for patients.**

| **Crosstab** | | | | | | |
| --- | --- | --- | --- | --- | --- | --- |
| Count | | | | | | |
|  | | Industry competition: Decision to adopt M-Health is appropriate when the hospital is facing high level of competition for patients. | | | | Total |
|  |  | Strongly disgree | Disagree | Agree | Strongly Agree |  |
| County | No | 6 | 16 | 9 | 5 | 36 |
|  | Yes | 33 | 68 | 52 | 22 | 175 |
| Total | | 39 | 84 | 61 | 27 | 211 |

| **Chi-Square Tests** | | | |
| --- | --- | --- | --- |
|  | Value | df | Asymptotic Significance (2-sided) |
| Pearson Chi-Square | .582^a^ | 3 | .901 |
| Likelihood Ratio | .585 | 3 | .900 |
| N of Valid Cases | 211 |  |  |
| a. 1 cells (12.5%) have expected count less than 5. The minimum expected count is 4.61. | | | |

**Country * Industry competition: Decision to adopt M-Health is appropriate when the hospital is facing high level of competition for patients.**

| **Crosstab** | | | | | | |
| --- | --- | --- | --- | --- | --- | --- |
| Count | | | | | | |
|  | | Industry competition: Decision to adopt M-Health is appropriate when the hospital is facing high level of competition for patients. | | | | Total |
|  |  | Strongly disgree | Disagree | Agree | Strongly Agree |  |
| Country | No | 8 | 29 | 18 | 6 | 61 |
|  | Yes | 31 | 54 | 43 | 21 | 149 |
|  | 2 | 0 | 1 | 0 | 0 | 1 |
| Total | | 39 | 84 | 61 | 27 | 211 |

| **Chi-Square Tests** | | | |
| --- | --- | --- | --- |
|  | Value | df | Asymptotic Significance (2-sided) |
| Pearson Chi-Square | 4.912^a^ | 6 | .555 |
| Likelihood Ratio | 5.324 | 6 | .503 |
| N of Valid Cases | 211 |  |  |
| a. 4 cells (33.3%) have expected count less than 5. The minimum expected count is .13. | | | |

**Eastern Africa * Industry competition: Decision to adopt M-Health is appropriate when the hospital is facing high level of competition for patients.**

| **Crosstab** | | | | | | |
| --- | --- | --- | --- | --- | --- | --- |
| Count | | | | | | |
|  | | Industry competition: Decision to adopt M-Health is appropriate when the hospital is facing high level of competition for patients. | | | | Total |
|  |  | Strongly disgree | Disagree | Agree | Strongly Agree |  |
| Eastern Africa | No | 22 | 57 | 38 | 15 | 132 |
|  | Yes | 17 | 27 | 23 | 12 | 79 |
| Total | | 39 | 84 | 61 | 27 | 211 |

| **Chi-Square Tests** | | | |
| --- | --- | --- | --- |
|  | Value | df | Asymptotic Significance (2-sided) |
| Pearson Chi-Square | 2.203^a^ | 3 | .531 |
| Likelihood Ratio | 2.202 | 3 | .532 |
| N of Valid Cases | 211 |  |  |
| a. 0 cells (0.0%) have expected count less than 5. The minimum expected count is 10.11. | | | |

| **Global Medical Tourism: The increased pace of medical tourism and borderless health care services requires strategic adoption of M-Health by hospitals. * Category of hospital classification Crosstabulation** | | | | | |
| --- | --- | --- | --- | --- | --- |
| Count | | | | | |
|  | | Category of hospital classification | | | Total |
|  |  | Public Hospital | Private Hospital | Faith-Based Hospital/NGO |  |
| Global Medical Tourism: The increased pace of medical tourism and borderless health care services requires strategic adoption of M-Health by hospitals. | Strongly disagree | 3 | 5 | 0 | 8 |
|  | Disagree | 4 | 7 | 0 | 11 |
|  | Agree | 55 | 40 | 22 | 117 |
|  | Strongly Agree | 40 | 24 | 11 | 75 |
| Total | | 102 | 76 | 33 | 211 |

| **Crosstab** | | | | | | |
| --- | --- | --- | --- | --- | --- | --- |
| Count | | | | | | |
|  | | Global Medical Tourism: The increased pace of medical tourism and borderless health care services requires strategic adoption of M-Health by hospitals. | | | | Total |
|  |  | Strongly disgree | Disagree | Agree | Strongly Agree |  |
| Classification level of facility | Level IV | 8 | 9 | 94 | 59 | 170 |
|  | Level V | 0 | 1 | 19 | 13 | 33 |
|  | Level VI | 0 | 1 | 4 | 3 | 8 |
| Total | | 8 | 11 | 117 | 75 | 211 |

| **Chi-Square Tests** | | | |
| --- | --- | --- | --- |
|  | Value | df | Asymptotic Significance (2-sided) |
| Pearson Chi-Square | 3.298^a^ | 6 | .771 |
| Likelihood Ratio | 4.645 | 6 | .590 |
| Linear-by-Linear Association | .861 | 1 | .353 |
| N of Valid Cases | 211 |  |  |
| a. 6 cells (50.0%) have expected count less than 5. The minimum expected count is .30. | | | |

**Category of hospital classification * Global Medical Tourism: The increased pace of medical tourism and borderless health care services requires strategic adoption of M-Health by hospitals.**

| **Crosstab** | | | | | | |
| --- | --- | --- | --- | --- | --- | --- |
| Count | | | | | | |
|  | | Global Medical Tourism: The increased pace of medical tourism and borderless health care services requires strategic adoption of M-Health by hospitals. | | | | Total |
|  |  | Strongly disgree | Disagree | Agree | Strongly Agree |  |
| Category of hospital classification | Public Hospital | 3 | 4 | 55 | 40 | 102 |
|  | Private Hospital | 5 | 7 | 40 | 24 | 76 |
|  | Faith-Based Hospital/NGO | 0 | 0 | 22 | 11 | 33 |
| Total | | 8 | 11 | 117 | 75 | 211 |

| **Chi-Square Tests** | | | |
| --- | --- | --- | --- |
|  | Value | df | Asymptotic Significance (2-sided) |
| Pearson Chi-Square | 9.048^a^ | 6 | .171 |
| Likelihood Ratio | 11.305 | 6 | .079 |
| Linear-by-Linear Association | .190 | 1 | .663 |
| N of Valid Cases | 211 |  |  |
| a. 5 cells (41.7%) have expected count less than 5. The minimum expected count is 1.25. | | | |

**Geographical category of facility classification * Global Medical Tourism: The increased pace of medical tourism and borderless health care services requires strategic adoption of M-Health by hospitals.**

| **Crosstab** | | | | | | |
| --- | --- | --- | --- | --- | --- | --- |
| Count | | | | | | |
|  | | Global Medical Tourism: The increased pace of medical tourism and borderless health care services requires strategic adoption of M-Health by hospitals. | | | | Total |
|  |  | Strongly disgree | Disagree | Agree | Strongly Agree |  |
| Geographical category of facility classification | Urban | 4 | 6 | 43 | 24 | 77 |
|  | Semi-urban | 4 | 3 | 43 | 30 | 80 |
|  | Rural | 0 | 2 | 31 | 21 | 54 |
| Total | | 8 | 11 | 117 | 75 | 211 |

| **Chi-Square Tests** | | | |
| --- | --- | --- | --- |
|  | Value | df | Asymptotic Significance (2-sided) |
| Pearson Chi-Square | 5.054^a^ | 6 | .537 |
| Likelihood Ratio | 6.975 | 6 | .323 |
| N of Valid Cases | 211 |  |  |
| a. 6 cells (50.0%) have expected count less than 5. The minimum expected count is 2.05. | | | |

**Current annual number of patients (outpatients and in-patients) * Global Medical Tourism: The increased pace of medical tourism and borderless health care services requires strategic adoption of M-Health by hospitals.**

| **Crosstab** | | | | | | |
| --- | --- | --- | --- | --- | --- | --- |
| Count | | | | | | |
|  | | Global Medical Tourism: The increased pace of medical tourism and borderless health care services requires strategic adoption of M-Health by hospitals. | | | | Total |
|  |  | Strongly disgree | Disagree | Agree | Strongly Agree |  |
| Current annual number of patients (outpatients and in-patients) | Less than 200 | 2 | 3 | 26 | 18 | 49 |
|  | 200-500 | 3 | 3 | 29 | 21 | 56 |
|  | 501-1000 | 0 | 2 | 13 | 9 | 24 |
|  | 1001-1500 | 2 | 0 | 9 | 2 | 13 |
|  | 1501-2000 | 0 | 2 | 6 | 7 | 15 |
|  | 2001 and above | 1 | 1 | 31 | 17 | 50 |
| Total | | 8 | 11 | 114 | 74 | 207 |

| **Chi-Square Tests** | | | |
| --- | --- | --- | --- |
|  | Value | df | Asymptotic Significance (2-sided) |
| Pearson Chi-Square | 14.571^a^ | 15 | .483 |
| Likelihood Ratio | 15.004 | 15 | .451 |
| Linear-by-Linear Association | .129 | 1 | .720 |
| N of Valid Cases | 207 |  |  |
| a. 13 cells (54.2%) have expected count less than 5. The minimum expected count is .50. | | | |

**Number of staff * Global Medical Tourism: The increased pace of medical tourism and borderless health care services requires strategic adoption of M-Health by hospitals.**

| **Crosstab** | | | | | | |
| --- | --- | --- | --- | --- | --- | --- |
| Count | | | | | | |
|  | | Global Medical Tourism: The increased pace of medical tourism and borderless health care services requires strategic adoption of M-Health by hospitals. | | | | Total |
|  |  | Strongly disgree | Disagree | Agree | Strongly Agree |  |
| Number of staff | Less than 100 | 5 | 6 | 74 | 40 | 125 |
|  | 100-200 | 2 | 2 | 27 | 20 | 51 |
|  | 201-300 | 1 | 1 | 5 | 6 | 13 |
|  | 301-500 | 0 | 1 | 6 | 5 | 12 |
|  | 501 and above | 0 | 1 | 5 | 4 | 10 |
| Total | | 8 | 11 | 117 | 75 | 211 |

| **Chi-Square Tests** | | | |
| --- | --- | --- | --- |
|  | Value | df | Asymptotic Significance (2-sided) |
| Pearson Chi-Square | 4.792^a^ | 12 | .965 |
| Likelihood Ratio | 5.418 | 12 | .943 |
| Linear-by-Linear Association | .637 | 1 | .425 |
| N of Valid Cases | 211 |  |  |
| a. 12 cells (60.0%) have expected count less than 5. The minimum expected count is .38. | | | |

**County * Global Medical Tourism: The increased pace of medical tourism and borderless health care services requires strategic adoption of M-Health by hospitals.**

| **Crosstab** | | | | | | |
| --- | --- | --- | --- | --- | --- | --- |
| Count | | | | | | |
|  | | Global Medical Tourism: The increased pace of medical tourism and borderless health care services requires strategic adoption of M-Health by hospitals. | | | | Total |
|  |  | Strongly disgree | Disagree | Agree | Strongly Agree |  |
| County | No | 3 | 4 | 16 | 13 | 36 |
|  | Yes | 5 | 7 | 101 | 62 | 175 |
| Total | | 8 | 11 | 117 | 75 | 211 |

| **Chi-Square Tests** | | | |
| --- | --- | --- | --- |
|  | Value | df | Asymptotic Significance (2-sided) |
| Pearson Chi-Square | 6.210^a^ | 3 | .102 |
| Likelihood Ratio | 5.249 | 3 | .154 |
| N of Valid Cases | 211 |  |  |
| a. 2 cells (25.0%) have expected count less than 5. The minimum expected count is 1.36. | | | |

**Country * Global Medical Tourism: The increased pace of medical tourism and borderless health care services requires strategic adoption of M-Health by hospitals.**

| **Crosstab** | | | | | | |
| --- | --- | --- | --- | --- | --- | --- |
| Count | | | | | | |
|  | | Global Medical Tourism: The increased pace of medical tourism and borderless health care services requires strategic adoption of M-Health by hospitals. | | | | Total |
|  |  | Strongly disgree | Disagree | Agree | Strongly Agree |  |
| Country | No | 3 | 3 | 33 | 22 | 61 |
|  | Yes | 5 | 8 | 83 | 53 | 149 |
|  | 2 | 0 | 0 | 1 | 0 | 1 |
| Total | | 8 | 11 | 117 | 75 | 211 |

| **Chi-Square Tests** | | | |
| --- | --- | --- | --- |
|  | Value | df | Asymptotic Significance (2-sided) |
| Pearson Chi-Square | 1.126^a^ | 6 | .980 |
| Likelihood Ratio | 1.487 | 6 | .960 |
| N of Valid Cases | 211 |  |  |
| a. 6 cells (50.0%) have expected count less than 5. The minimum expected count is .04. | | | |

**Eastern Africa * Global Medical Tourism: The increased pace of medical tourism and borderless health care services requires strategic adoption of M-Health by hospitals.**

| **Crosstab** | | | | | | |
| --- | --- | --- | --- | --- | --- | --- |
| Count | | | | | | |
|  | | Global Medical Tourism: The increased pace of medical tourism and borderless health care services requires strategic adoption of M-Health by hospitals. | | | | Total |
|  |  | Strongly disgree | Disagree | Agree | Strongly Agree |  |
| Eastern Africa | No | 5 | 7 | 71 | 49 | 132 |
|  | Yes | 3 | 4 | 46 | 26 | 79 |
| Total | | 8 | 11 | 117 | 75 | 211 |

| **Chi-Square Tests** | | | |
| --- | --- | --- | --- |
|  | Value | df | Asymptotic Significance (2-sided) |
| Pearson Chi-Square | .428^a^ | 3 | .934 |
| Likelihood Ratio | .429 | 3 | .934 |
| N of Valid Cases | 211 |  |  |
| a. 2 cells (25.0%) have expected count less than 5. The minimum expected count is 3.00. | | | |

| **Government support: Government and counties’ incentives (such as tax cuts) is important for adoption of M-Health . * Category of hospital classification Crosstabulation** | | | | | |
| --- | --- | --- | --- | --- | --- |
| Count | | | | | |
|  | | Category of hospital classification | | | Total |
|  |  | Public Hospital | Private Hospital | Faith-Based Hospital/NGO |  |
| Government support: Government and counties’ incentives (such as tax cuts) is important for adoption of M-Health . | Strongly disagree | 2 | 5 | 1 | 8 |
|  | Disagree | 11 | 11 | 5 | 27 |
|  | Agree | 47 | 32 | 13 | 92 |
|  | Strongly Agree | 42 | 27 | 12 | 81 |
| Total | | 102 | 75 | 31 | 208 |

| **Crosstab** | | | | | | |
| --- | --- | --- | --- | --- | --- | --- |
| Count | | | | | | |
|  | | Government support: Government and counties’ incentives (such as tax cuts) is important for adoption of M-Health . | | | | Total |
|  |  | Strongly disgree | Disagree | Agree | Strongly Agree |  |
| Classification level of facility | Level IV | 7 | 22 | 79 | 59 | 167 |
|  | Level V | 1 | 4 | 11 | 17 | 33 |
|  | Level VI | 0 | 1 | 2 | 5 | 8 |
| Total | | 8 | 27 | 92 | 81 | 208 |

| **Chi-Square Tests** | | | |
| --- | --- | --- | --- |
|  | Value | df | Asymptotic Significance (2-sided) |
| Pearson Chi-Square | 5.393^a^ | 6 | .494 |
| Likelihood Ratio | 5.620 | 6 | .467 |
| Linear-by-Linear Association | 2.938 | 1 | .087 |
| N of Valid Cases | 208 |  |  |
| a. 6 cells (50.0%) have expected count less than 5. The minimum expected count is .31. | | | |

**Category of hospital classification * Government support: Government and counties’ incentives (such as tax cuts) is important for adoption of M-Health .**

| **Crosstab** | | | | | | |
| --- | --- | --- | --- | --- | --- | --- |
| Count | | | | | | |
|  | | Government support: Government and counties’ incentives (such as tax cuts) is important for adoption of M-Health . | | | | Total |
|  |  | Strongly disgree | Disagree | Agree | Strongly Agree |  |
| Category of hospital classification | Public Hospital | 2 | 11 | 47 | 42 | 102 |
|  | Private Hospital | 5 | 11 | 32 | 27 | 75 |
|  | Faith-Based Hospital/NGO | 1 | 5 | 13 | 12 | 31 |
| Total | | 8 | 27 | 92 | 81 | 208 |

| **Chi-Square Tests** | | | |
| --- | --- | --- | --- |
|  | Value | df | Asymptotic Significance (2-sided) |
| Pearson Chi-Square | 3.760^a^ | 6 | .709 |
| Likelihood Ratio | 3.690 | 6 | .718 |
| Linear-by-Linear Association | 1.166 | 1 | .280 |
| N of Valid Cases | 208 |  |  |
| a. 4 cells (33.3%) have expected count less than 5. The minimum expected count is 1.19. | | | |

**Geographical category of facility classification * Government support: Government and counties’ incentives (such as tax cuts) is important for adoption of M-Health .**

| **Crosstab** | | | | | | |
| --- | --- | --- | --- | --- | --- | --- |
| Count | | | | | | |
|  | | Government support: Government and counties’ incentives (such as tax cuts) is important for adoption of M-Health . | | | | Total |
|  |  | Strongly disgree | Disagree | Agree | Strongly Agree |  |
| Geographical category of facility classification | Urban | 4 | 7 | 33 | 31 | 75 |
|  | Semi-urban | 4 | 16 | 32 | 28 | 80 |
|  | Rural | 0 | 4 | 27 | 22 | 53 |
| Total | | 8 | 27 | 92 | 81 | 208 |

| **Chi-Square Tests** | | | |
| --- | --- | --- | --- |
|  | Value | df | Asymptotic Significance (2-sided) |
| Pearson Chi-Square | 9.141^a^ | 6 | .166 |
| Likelihood Ratio | 10.966 | 6 | .089 |
| N of Valid Cases | 208 |  |  |
| a. 3 cells (25.0%) have expected count less than 5. The minimum expected count is 2.04. | | | |

**Current annual number of patients (outpatients and in-patients) * Government support: Government and counties’ incentives (such as tax cuts) is important for adoption of M-Health .**

| **Crosstab** | | | | | | |
| --- | --- | --- | --- | --- | --- | --- |
| Count | | | | | | |
|  | | Government support: Government and counties’ incentives (such as tax cuts) is important for adoption of M-Health . | | | | Total |
|  |  | Strongly disgree | Disagree | Agree | Strongly Agree |  |
| Current annual number of patients (outpatients and in-patients) | Less than 200 | 3 | 7 | 26 | 13 | 49 |
|  | 200-500 | 3 | 7 | 22 | 22 | 54 |
|  | 501-1000 | 0 | 4 | 7 | 13 | 24 |
|  | 1001-1500 | 1 | 0 | 5 | 6 | 12 |
|  | 1501-2000 | 0 | 2 | 8 | 5 | 15 |
|  | 2001 and above | 1 | 7 | 20 | 22 | 50 |
| Total | | 8 | 27 | 88 | 81 | 204 |

| **Chi-Square Tests** | | | |
| --- | --- | --- | --- |
|  | Value | df | Asymptotic Significance (2-sided) |
| Pearson Chi-Square | 12.321^a^ | 15 | .655 |
| Likelihood Ratio | 15.421 | 15 | .422 |
| Linear-by-Linear Association | 2.117 | 1 | .146 |
| N of Valid Cases | 204 |  |  |
| a. 10 cells (41.7%) have expected count less than 5. The minimum expected count is .47. | | | |

**Number of staff * Government support: Government and counties’ incentives (such as tax cuts) is important for adoption of M-Health .**

| **Crosstab** | | | | | | |
| --- | --- | --- | --- | --- | --- | --- |
| Count | | | | | | |
|  | | Government support: Government and counties’ incentives (such as tax cuts) is important for adoption of M-Health . | | | | Total |
|  |  | Strongly disgree | Disagree | Agree | Strongly Agree |  |
| Number of staff | Less than 100 | 6 | 16 | 59 | 41 | 122 |
|  | 100-200 | 2 | 7 | 18 | 24 | 51 |
|  | 201-300 | 0 | 3 | 5 | 5 | 13 |
|  | 301-500 | 0 | 1 | 4 | 7 | 12 |
|  | 501 and above | 0 | 0 | 6 | 4 | 10 |
| Total | | 8 | 27 | 92 | 81 | 208 |

| **Chi-Square Tests** | | | |
| --- | --- | --- | --- |
|  | Value | df | Asymptotic Significance (2-sided) |
| Pearson Chi-Square | 9.544^a^ | 12 | .656 |
| Likelihood Ratio | 11.861 | 12 | .457 |
| Linear-by-Linear Association | 3.198 | 1 | .074 |
| N of Valid Cases | 208 |  |  |
| a. 11 cells (55.0%) have expected count less than 5. The minimum expected count is .38. | | | |

**County * Government support: Government and counties’ incentives (such as tax cuts) is important for adoption of M-Health .**

| **Crosstab** | | | | | | |
| --- | --- | --- | --- | --- | --- | --- |
| Count | | | | | | |
|  | | Government support: Government and counties’ incentives (such as tax cuts) is important for adoption of M-Health . | | | | Total |
|  |  | Strongly disgree | Disagree | Agree | Strongly Agree |  |
| County | No | 2 | 3 | 17 | 12 | 34 |
|  | Yes | 6 | 24 | 75 | 69 | 174 |
| Total | | 8 | 27 | 92 | 81 | 208 |

| **Chi-Square Tests** | | | |
| --- | --- | --- | --- |
|  | Value | df | Asymptotic Significance (2-sided) |
| Pearson Chi-Square | 1.424^a^ | 3 | .700 |
| Likelihood Ratio | 1.425 | 3 | .700 |
| N of Valid Cases | 208 |  |  |
| a. 2 cells (25.0%) have expected count less than 5. The minimum expected count is 1.31. | | | |

**Country * Government support: Government and counties’ incentives (such as tax cuts) is important for adoption of M-Health .**

| **Crosstab** | | | | | | |
| --- | --- | --- | --- | --- | --- | --- |
| Count | | | | | | |
|  | | Government support: Government and counties’ incentives (such as tax cuts) is important for adoption of M-Health . | | | | Total |
|  |  | Strongly disgree | Disagree | Agree | Strongly Agree |  |
| Country | No | 3 | 8 | 30 | 20 | 61 |
|  | Yes | 5 | 19 | 61 | 61 | 146 |
|  | 2 | 0 | 0 | 1 | 0 | 1 |
| Total | | 8 | 27 | 92 | 81 | 208 |

| **Chi-Square Tests** | | | |
| --- | --- | --- | --- |
|  | Value | df | Asymptotic Significance (2-sided) |
| Pearson Chi-Square | 2.943^a^ | 6 | .816 |
| Likelihood Ratio | 3.320 | 6 | .768 |
| N of Valid Cases | 208 |  |  |
| a. 5 cells (41.7%) have expected count less than 5. The minimum expected count is .04. | | | |

**Eastern Africa * Government support: Government and counties’ incentives (such as tax cuts) is important for adoption of M-Health .**

| **Crosstab** | | | | | | |
| --- | --- | --- | --- | --- | --- | --- |
| Count | | | | | | |
|  | | Government support: Government and counties’ incentives (such as tax cuts) is important for adoption of M-Health . | | | | Total |
|  |  | Strongly disgree | Disagree | Agree | Strongly Agree |  |
| Eastern Africa | No | 6 | 12 | 65 | 47 | 130 |
|  | Yes | 2 | 15 | 27 | 34 | 78 |
| Total | | 8 | 27 | 92 | 81 | 208 |

| **Chi-Square Tests** | | | |
| --- | --- | --- | --- |
|  | Value | df | Asymptotic Significance (2-sided) |
| Pearson Chi-Square | 7.590^a^ | 3 | .055 |
| Likelihood Ratio | 7.559 | 3 | .056 |
| N of Valid Cases | 208 |  |  |
| a. 1 cells (12.5%) have expected count less than 5. The minimum expected count is 3.00. | | | |

| **Patients pressure: Decision to adopt M-Health was (or will be) driven by patients’ demand for M-health services * Category of hospital classification Crosstabulation** | | | | | |
| --- | --- | --- | --- | --- | --- |
| Count | | | | | |
|  | | Category of hospital classification | | | Total |
|  |  | Public Hospital | Private Hospital | Faith-Based Hospital/NGO |  |
| Patients pressure: Decision to adopt M-Health was (or will be) driven by patients’ demand for M-health services | Strongly disagree | 6 | 7 | 4 | 17 |
|  | Disagree | 34 | 24 | 10 | 68 |
|  | Agree | 41 | 27 | 9 | 77 |
|  | Strongly Agree | 21 | 18 | 10 | 49 |
| Total | | 102 | 76 | 33 | 211 |

| **Crosstab** | | | | | | |
| --- | --- | --- | --- | --- | --- | --- |
| Count | | | | | | |
|  | | Patients pressure: Decision to adopt M-Health was (or will be) driven by patients’ demand for M-health services | | | | Total |
|  |  | Strongly disgree | Disagree | Agree | Strongly Agree |  |
| Classification level of facility | Level IV | 14 | 56 | 62 | 38 | 170 |
|  | Level V | 3 | 10 | 12 | 8 | 33 |
|  | Level VI | 0 | 2 | 3 | 3 | 8 |
| Total | | 17 | 68 | 77 | 49 | 211 |

| **Chi-Square Tests** | | | |
| --- | --- | --- | --- |
|  | Value | df | Asymptotic Significance (2-sided) |
| Pearson Chi-Square | 1.664^a^ | 6 | .948 |
| Likelihood Ratio | 2.208 | 6 | .900 |
| Linear-by-Linear Association | .908 | 1 | .341 |
| N of Valid Cases | 211 |  |  |
| a. 5 cells (41.7%) have expected count less than 5. The minimum expected count is .64. | | | |

**Category of hospital classification * Patients pressure: Decision to adopt M-Health was (or will be) driven by patients’ demand for M-health services**

| **Crosstab** | | | | | | |
| --- | --- | --- | --- | --- | --- | --- |
| Count | | | | | | |
|  | | Patients pressure: Decision to adopt M-Health was (or will be) driven by patients’ demand for M-health services | | | | Total |
|  |  | Strongly disgree | Disagree | Agree | Strongly Agree |  |
| Category of hospital classification | Public Hospital | 6 | 34 | 41 | 21 | 102 |
|  | Private Hospital | 7 | 24 | 27 | 18 | 76 |
|  | Faith-Based Hospital/NGO | 4 | 10 | 9 | 10 | 33 |
| Total | | 17 | 68 | 77 | 49 | 211 |

| **Chi-Square Tests** | | | |
| --- | --- | --- | --- |
|  | Value | df | Asymptotic Significance (2-sided) |
| Pearson Chi-Square | 3.683^a^ | 6 | .719 |
| Likelihood Ratio | 3.660 | 6 | .723 |
| Linear-by-Linear Association | .001 | 1 | .972 |
| N of Valid Cases | 211 |  |  |
| a. 1 cells (8.3%) have expected count less than 5. The minimum expected count is 2.66. | | | |

**Geographical category of facility classification * Patients pressure: Decision to adopt M-Health was (or will be) driven by patients’ demand for M-health services**

| **Crosstab** | | | | | | |
| --- | --- | --- | --- | --- | --- | --- |
| Count | | | | | | |
|  | | Patients pressure: Decision to adopt M-Health was (or will be) driven by patients’ demand for M-health services | | | | Total |
|  |  | Strongly disgree | Disagree | Agree | Strongly Agree |  |
| Geographical category of facility classification | Urban | 7 | 23 | 27 | 20 | 77 |
|  | Semi-urban | 9 | 32 | 20 | 19 | 80 |
|  | Rural | 1 | 13 | 30 | 10 | 54 |
| Total | | 17 | 68 | 77 | 49 | 211 |

| **Chi-Square Tests** | | | |
| --- | --- | --- | --- |
|  | Value | df | Asymptotic Significance (2-sided) |
| Pearson Chi-Square | 15.532^a^ | 6 | .016 |
| Likelihood Ratio | 16.244 | 6 | .013 |
| N of Valid Cases | 211 |  |  |
| a. 1 cells (8.3%) have expected count less than 5. The minimum expected count is 4.35. | | | |

**Current annual number of patients (outpatients and in-patients) * Patients pressure: Decision to adopt M-Health was (or will be) driven by patients’ demand for M-health services**

| **Crosstab** | | | | | | |
| --- | --- | --- | --- | --- | --- | --- |
| Count | | | | | | |
|  | | Patients pressure: Decision to adopt M-Health was (or will be) driven by patients’ demand for M-health services | | | | Total |
|  |  | Strongly disgree | Disagree | Agree | Strongly Agree |  |
| Current annual number of patients (outpatients and in-patients) | Less than 200 | 7 | 7 | 24 | 11 | 49 |
|  | 200-500 | 5 | 21 | 14 | 16 | 56 |
|  | 501-1000 | 1 | 9 | 8 | 6 | 24 |
|  | 1001-1500 | 1 | 7 | 2 | 3 | 13 |
|  | 1501-2000 | 0 | 7 | 6 | 2 | 15 |
|  | 2001 and above | 3 | 17 | 19 | 11 | 50 |
| Total | | 17 | 68 | 73 | 49 | 207 |

| **Chi-Square Tests** | | | |
| --- | --- | --- | --- |
|  | Value | df | Asymptotic Significance (2-sided) |
| Pearson Chi-Square | 19.877^a^ | 15 | .177 |
| Likelihood Ratio | 22.216 | 15 | .102 |
| Linear-by-Linear Association | .077 | 1 | .781 |
| N of Valid Cases | 207 |  |  |
| a. 11 cells (45.8%) have expected count less than 5. The minimum expected count is 1.07. | | | |

**Number of staff * Patients pressure: Decision to adopt M-Health was (or will be) driven by patients’ demand for M-health services**

| **Crosstab** | | | | | | |
| --- | --- | --- | --- | --- | --- | --- |
| Count | | | | | | |
|  | | Patients pressure: Decision to adopt M-Health was (or will be) driven by patients’ demand for M-health services | | | | Total |
|  |  | Strongly disgree | Disagree | Agree | Strongly Agree |  |
| Number of staff | Less than 100 | 12 | 40 | 49 | 24 | 125 |
|  | 100-200 | 4 | 18 | 16 | 13 | 51 |
|  | 201-300 | 1 | 5 | 4 | 3 | 13 |
|  | 301-500 | 0 | 2 | 6 | 4 | 12 |
|  | 501 and above | 0 | 3 | 2 | 5 | 10 |
| Total | | 17 | 68 | 77 | 49 | 211 |

| **Chi-Square Tests** | | | |
| --- | --- | --- | --- |
|  | Value | df | Asymptotic Significance (2-sided) |
| Pearson Chi-Square | 10.051^a^ | 12 | .611 |
| Likelihood Ratio | 11.302 | 12 | .503 |
| Linear-by-Linear Association | 4.647 | 1 | .031 |
| N of Valid Cases | 211 |  |  |
| a. 13 cells (65.0%) have expected count less than 5. The minimum expected count is .81. | | | |

**County * Patients pressure: Decision to adopt M-Health was (or will be) driven by patients’ demand for M-health services**

| **Crosstab** | | | | | | |
| --- | --- | --- | --- | --- | --- | --- |
| Count | | | | | | |
|  | | Patients pressure: Decision to adopt M-Health was (or will be) driven by patients’ demand for M-health services | | | | Total |
|  |  | Strongly disgree | Disagree | Agree | Strongly Agree |  |
| County | No | 2 | 14 | 8 | 12 | 36 |
|  | Yes | 15 | 54 | 69 | 37 | 175 |
| Total | | 17 | 68 | 77 | 49 | 211 |

| **Chi-Square Tests** | | | |
| --- | --- | --- | --- |
|  | Value | df | Asymptotic Significance (2-sided) |
| Pearson Chi-Square | 5.268^a^ | 3 | .153 |
| Likelihood Ratio | 5.410 | 3 | .144 |
| N of Valid Cases | 211 |  |  |
| a. 1 cells (12.5%) have expected count less than 5. The minimum expected count is 2.90. | | | |

**Country * Patients pressure: Decision to adopt M-Health was (or will be) driven by patients’ demand for M-health services**

| **Crosstab** | | | | | | |
| --- | --- | --- | --- | --- | --- | --- |
| Count | | | | | | |
|  | | Patients pressure: Decision to adopt M-Health was (or will be) driven by patients’ demand for M-health services | | | | Total |
|  |  | Strongly disgree | Disagree | Agree | Strongly Agree |  |
| Country | No | 4 | 18 | 25 | 14 | 61 |
|  | Yes | 13 | 50 | 51 | 35 | 149 |
|  | 2 | 0 | 0 | 1 | 0 | 1 |
| Total | | 17 | 68 | 77 | 49 | 211 |

| **Chi-Square Tests** | | | |
| --- | --- | --- | --- |
|  | Value | df | Asymptotic Significance (2-sided) |
| Pearson Chi-Square | 2.768^a^ | 6 | .837 |
| Likelihood Ratio | 3.047 | 6 | .803 |
| N of Valid Cases | 211 |  |  |
| a. 5 cells (41.7%) have expected count less than 5. The minimum expected count is .08. | | | |

**Eastern Africa * Patients pressure: Decision to adopt M-Health was (or will be) driven by patients’ demand for M-health services**

| **Crosstab** | | | | | | |
| --- | --- | --- | --- | --- | --- | --- |
| Count | | | | | | |
|  | | Patients pressure: Decision to adopt M-Health was (or will be) driven by patients’ demand for M-health services | | | | Total |
|  |  | Strongly disgree | Disagree | Agree | Strongly Agree |  |
| Eastern Africa | No | 8 | 41 | 54 | 29 | 132 |
|  | Yes | 9 | 27 | 23 | 20 | 79 |
| Total | | 17 | 68 | 77 | 49 | 211 |

| **Chi-Square Tests** | | | |
| --- | --- | --- | --- |
|  | Value | df | Asymptotic Significance (2-sided) |
| Pearson Chi-Square | 4.015^a^ | 3 | .260 |
| Likelihood Ratio | 4.010 | 3 | .260 |
| N of Valid Cases | 211 |  |  |
| a. 0 cells (0.0%) have expected count less than 5. The minimum expected count is 6.36. | | | |

| **Support from professional associations: Decision to adopt M-health was (will be) dependent on support from medical professional associations as an accepted standard. * Category of hospital classification Crosstabulation** | | | | | |
| --- | --- | --- | --- | --- | --- |
| Count | | | | | |
|  | | Category of hospital classification | | | Total |
|  |  | Public Hospital | Private Hospital | Faith-Based Hospital/NGO |  |
| Support from professional associations: Decision to adopt M-health was (will be) dependent on support from medical professional associations as an accepted standard. | Strongly disgree | 5 | 6 | 1 | 12 |
|  | Disagree | 14 | 10 | 3 | 27 |
|  | Agree | 49 | 42 | 21 | 112 |
|  | Strongly Agree | 34 | 18 | 8 | 60 |
| Total | | 102 | 76 | 33 | 211 |

| **Crosstab** | | | | | | |
| --- | --- | --- | --- | --- | --- | --- |
| Count | | | | | | |
|  | | Support from professional associations: Decision to adopt M-health was (will be) dependent on support from medical professional associations as an accepted standard. | | | | Total |
|  |  | Strongly disgree | Disagree | Agree | Strongly Agree |  |
| Classification level of facility | Level IV | 10 | 18 | 97 | 45 | 170 |
|  | Level V | 2 | 7 | 10 | 14 | 33 |
|  | Level VI | 0 | 2 | 5 | 1 | 8 |
| Total | | 12 | 27 | 112 | 60 | 211 |

| **Chi-Square Tests** | | | |
| --- | --- | --- | --- |
|  | Value | df | Asymptotic Significance (2-sided) |
| Pearson Chi-Square | 10.962^a^ | 6 | .090 |
| Likelihood Ratio | 11.420 | 6 | .076 |
| Linear-by-Linear Association | .035 | 1 | .853 |
| N of Valid Cases | 211 |  |  |
| a. 6 cells (50.0%) have expected count less than 5. The minimum expected count is .45. | | | |

**Category of hospital classification * Support from professional associations: Decision to adopt M-health was (will be) dependent on support from medical professional associations as an accepted standard.**

| **Crosstab** | | | | | | |
| --- | --- | --- | --- | --- | --- | --- |
| Count | | | | | | |
|  | | Support from professional associations: Decision to adopt M-health was (will be) dependent on support from medical professional associations as an accepted standard. | | | | Total |
|  |  | Strongly disgree | Disagree | Agree | Strongly Agree |  |
| Category of hospital classification | Public Hospital | 5 | 14 | 49 | 34 | 102 |
|  | Private Hospital | 6 | 10 | 42 | 18 | 76 |
|  | Faith-Based Hospital/NGO | 1 | 3 | 21 | 8 | 33 |
| Total | | 12 | 27 | 112 | 60 | 211 |

| **Chi-Square Tests** | | | |
| --- | --- | --- | --- |
|  | Value | df | Asymptotic Significance (2-sided) |
| Pearson Chi-Square | 4.519^a^ | 6 | .607 |
| Likelihood Ratio | 4.553 | 6 | .602 |
| Linear-by-Linear Association | .228 | 1 | .633 |
| N of Valid Cases | 211 |  |  |
| a. 3 cells (25.0%) have expected count less than 5. The minimum expected count is 1.88. | | | |

**Geographical category of facility classification * Support from professional associations: Decision to adopt M-health was (will be) dependent on support from medical professional associations as an accepted standard.**

| **Crosstab** | | | | | | |
| --- | --- | --- | --- | --- | --- | --- |
| Count | | | | | | |
|  | | Support from professional associations: Decision to adopt M-health was (will be) dependent on support from medical professional associations as an accepted standard. | | | | Total |
|  |  | Strongly disgree | Disagree | Agree | Strongly Agree |  |
| Geographical category of facility classification | Urban | 4 | 13 | 39 | 21 | 77 |
|  | Semi-urban | 5 | 10 | 37 | 28 | 80 |
|  | Rural | 3 | 4 | 36 | 11 | 54 |
| Total | | 12 | 27 | 112 | 60 | 211 |

| **Chi-Square Tests** | | | |
| --- | --- | --- | --- |
|  | Value | df | Asymptotic Significance (2-sided) |
| Pearson Chi-Square | 7.466^a^ | 6 | .280 |
| Likelihood Ratio | 7.539 | 6 | .274 |
| N of Valid Cases | 211 |  |  |
| a. 3 cells (25.0%) have expected count less than 5. The minimum expected count is 3.07. | | | |

**Current annual number of patients (outpatients and in-patients) * Support from professional associations: Decision to adopt M-health was (will be) dependent on support from medical professional associations as an accepted standard.**

| **Crosstab** | | | | | | |
| --- | --- | --- | --- | --- | --- | --- |
| Count | | | | | | |
|  | | Support from professional associations: Decision to adopt M-health was (will be) dependent on support from medical professional associations as an accepted standard. | | | | Total |
|  |  | Strongly disgree | Disagree | Agree | Strongly Agree |  |
| Current annual number of patients (outpatients and in-patients) | Less than 200 | 2 | 2 | 35 | 10 | 49 |
|  | 200-500 | 3 | 8 | 26 | 19 | 56 |
|  | 501-1000 | 1 | 4 | 12 | 7 | 24 |
|  | 1001-1500 | 3 | 1 | 5 | 4 | 13 |
|  | 1501-2000 | 0 | 2 | 8 | 5 | 15 |
|  | 2001 and above | 3 | 10 | 22 | 15 | 50 |
| Total | | 12 | 27 | 108 | 60 | 207 |

| **Chi-Square Tests** | | | |
| --- | --- | --- | --- |
|  | Value | df | Asymptotic Significance (2-sided) |
| Pearson Chi-Square | 20.229^a^ | 15 | .163 |
| Likelihood Ratio | 18.974 | 15 | .215 |
| Linear-by-Linear Association | .431 | 1 | .512 |
| N of Valid Cases | 207 |  |  |
| a. 11 cells (45.8%) have expected count less than 5. The minimum expected count is .75. | | | |

**Number of staff * Support from professional associations: Decision to adopt M-health was (will be) dependent on support from medical professional associations as an accepted standard.**

| **Crosstab** | | | | | | |
| --- | --- | --- | --- | --- | --- | --- |
| Count | | | | | | |
|  | | Support from professional associations: Decision to adopt M-health was (will be) dependent on support from medical professional associations as an accepted standard. | | | | Total |
|  |  | Strongly disgree | Disagree | Agree | Strongly Agree |  |
| Number of staff | Less than 100 | 8 | 11 | 79 | 27 | 125 |
|  | 100-200 | 4 | 7 | 19 | 21 | 51 |
|  | 201-300 | 0 | 5 | 4 | 4 | 13 |
|  | 301-500 | 0 | 2 | 5 | 5 | 12 |
|  | 501 and above | 0 | 2 | 5 | 3 | 10 |
| Total | | 12 | 27 | 112 | 60 | 211 |

| **Chi-Square Tests** | | | |
| --- | --- | --- | --- |
|  | Value | df | Asymptotic Significance (2-sided) |
| Pearson Chi-Square | 23.438^a^ | 12 | .024 |
| Likelihood Ratio | 23.263 | 12 | .026 |
| Linear-by-Linear Association | .658 | 1 | .417 |
| N of Valid Cases | 211 |  |  |
| a. 10 cells (50.0%) have expected count less than 5. The minimum expected count is .57. | | | |

**County * Support from professional associations: Decision to adopt M-health was (will be) dependent on support from medical professional associations as an accepted standard.**

| **Crosstab** | | | | | | |
| --- | --- | --- | --- | --- | --- | --- |
| Count | | | | | | |
|  | | Support from professional associations: Decision to adopt M-health was (will be) dependent on support from medical professional associations as an accepted standard. | | | | Total |
|  |  | Strongly disgree | Disagree | Agree | Strongly Agree |  |
| County | No | 3 | 7 | 16 | 10 | 36 |
|  | Yes | 9 | 20 | 96 | 50 | 175 |
| Total | | 12 | 27 | 112 | 60 | 211 |

| **Chi-Square Tests** | | | |
| --- | --- | --- | --- |
|  | Value | df | Asymptotic Significance (2-sided) |
| Pearson Chi-Square | 2.650^a^ | 3 | .449 |
| Likelihood Ratio | 2.463 | 3 | .482 |
| N of Valid Cases | 211 |  |  |
| a. 2 cells (25.0%) have expected count less than 5. The minimum expected count is 2.05. | | | |

**Country * Support from professional associations: Decision to adopt M-health was (will be) dependent on support from medical professional associations as an accepted standard.**

| **Crosstab** | | | | | | |
| --- | --- | --- | --- | --- | --- | --- |
| Count | | | | | | |
|  | | Support from professional associations: Decision to adopt M-health was (will be) dependent on support from medical professional associations as an accepted standard. | | | | Total |
|  |  | Strongly disgree | Disagree | Agree | Strongly Agree |  |
| Country | No | 4 | 7 | 31 | 19 | 61 |
|  | Yes | 8 | 20 | 80 | 41 | 149 |
|  | 2 | 0 | 0 | 1 | 0 | 1 |
| Total | | 12 | 27 | 112 | 60 | 211 |

| **Chi-Square Tests** | | | |
| --- | --- | --- | --- |
|  | Value | df | Asymptotic Significance (2-sided) |
| Pearson Chi-Square | 1.392^a^ | 6 | .966 |
| Likelihood Ratio | 1.770 | 6 | .940 |
| N of Valid Cases | 211 |  |  |
| a. 5 cells (41.7%) have expected count less than 5. The minimum expected count is .06. | | | |

**Eastern Africa * Support from professional associations: Decision to adopt M-health was (will be) dependent on support from medical professional associations as an accepted standard.**

| **Crosstab** | | | | | | |
| --- | --- | --- | --- | --- | --- | --- |
| Count | | | | | | |
|  | | Support from professional associations: Decision to adopt M-health was (will be) dependent on support from medical professional associations as an accepted standard. | | | | Total |
|  |  | Strongly disgree | Disagree | Agree | Strongly Agree |  |
| Eastern Africa | No | 8 | 14 | 73 | 37 | 132 |
|  | Yes | 4 | 13 | 39 | 23 | 79 |
| Total | | 12 | 27 | 112 | 60 | 211 |

| **Chi-Square Tests** | | | |
| --- | --- | --- | --- |
|  | Value | df | Asymptotic Significance (2-sided) |
| Pearson Chi-Square | 1.756^a^ | 3 | .624 |
| Likelihood Ratio | 1.723 | 3 | .632 |
| N of Valid Cases | 211 |  |  |
| a. 1 cells (12.5%) have expected count less than 5. The minimum expected count is 4.49. | | | |

| **Support from medical health insurance: Adoption of M-Health was (will be) dependent on health insurance companies approval and payment of claims for services rendered through M-Health * Category of hospital classification Crosstabulation** | | | | | |
| --- | --- | --- | --- | --- | --- |
| Count | | | | | |
|  | | Category of hospital classification | | | Total |
|  |  | Public Hospital | Private Hospital | Faith-Based Hospital/NGO |  |
| Support from medical health insurance: Adoption of M-Health was (will be) dependent on health insurance companies approval and payment of claims for services rendered through M-Health | Strongly disgree | 8 | 5 | 1 | 14 |
|  | Disagree | 22 | 12 | 4 | 38 |
|  | Agree | 48 | 38 | 22 | 108 |
|  | Strongly Agree | 24 | 20 | 6 | 50 |
| Total | | 102 | 75 | 33 | 210 |

1. **Indicate the extent to which you agree with the following statements using 1=strongly disagree.....4= strongly agree.**

| **M-Health will disrupt the way healthcare is delivered in Kenya within the next 10 years * Category of hospital classification Crosstabulation** | | | | | |
| --- | --- | --- | --- | --- | --- |
| Count | | | | | |
|  | | Category of hospital classification | | | Total |
|  |  | Public Hospital | Private Hospital | Faith-Based Hospital/NGO |  |
| M-Health will disrupt the way healthcare is delivered in Kenya within the next 10 years | Strongly disagree | 37 | 27 | 12 | 76 |
|  | Disagree | 27 | 12 | 8 | 47 |
|  | Agree | 21 | 16 | 6 | 43 |
|  | Strongly Agree | 17 | 21 | 7 | 45 |
| Total | | 102 | 76 | 33 | 211 |

| **M-health will increase competition among Kenya hospitals and with international hospitals * Category of hospital classification Crosstabulation** | | | | | |
| --- | --- | --- | --- | --- | --- |
| Count | | | | | |
|  | | Category of hospital classification | | | Total |
|  |  | Public Hospital | Private Hospital | Faith-Based Hospital/NGO |  |
| M-health will increase competition among Kenya hospitals and with international hospitals | Strongly disagree | 7 | 4 | 2 | 13 |
|  | Disagree | 10 | 8 | 4 | 22 |
|  | Agree | 53 | 26 | 18 | 97 |
|  | Strongly Agree | 32 | 38 | 9 | 79 |
| Total | | 102 | 76 | 33 | 211 |

| **M-health has the potential to reduce the cost of health care to the patient * Category of hospital classification Crosstabulation** | | | | | |
| --- | --- | --- | --- | --- | --- |
| Count | | | | | |
|  | | Category of hospital classification | | | Total |
|  |  | Public Hospital | Private Hospital | Faith-Based Hospital/NGO |  |
| M-health has the potential to reduce the cost of health care to the patient | Strongly disagree | 6 | 6 | 2 | 14 |
|  | Disagree | 13 | 12 | 4 | 29 |
|  | Agree | 45 | 31 | 17 | 93 |
|  | Strongly Agree | 38 | 27 | 9 | 74 |
| Total | | 102 | 76 | 32 | 210 |

| **Kenya is well-positioned to become the leader in M-health as it did with Mobile Financing * Category of hospital classification Crosstabulation** | | | | | |
| --- | --- | --- | --- | --- | --- |
| Count | | | | | |
|  | | Category of hospital classification | | | Total |
|  |  | Public Hospital | Private Hospital | Faith-Based Hospital/NGO |  |
| Kenya is well-positioned to become the leader in M-health as it did with Mobile Financing | Strongly disagree | 5 | 1 | 1 | 7 |
|  | Disagree | 11 | 5 | 5 | 21 |
|  | Agree | 44 | 30 | 14 | 88 |
|  | Strongly Agree | 41 | 39 | 11 | 91 |
| Total | | 101 | 75 | 31 | 207 |

| **M-health will reduce the ability of hospitals to keep their customer-bases and to insulate themselves against the competition * Category of hospital classification Crosstabulation** | | | | | |
| --- | --- | --- | --- | --- | --- |
| Count | | | | | |
|  | | Category of hospital classification | | | Total |
|  |  | Public Hospital | Private Hospital | Faith-Based Hospital/NGO |  |
| M-health will reduce the ability of hospitals to keep their customer-bases and to insulate themselves against the competition | Strongly disagree | 19 | 17 | 6 | 42 |
|  | Disagree | 37 | 23 | 15 | 75 |
|  | Agree | 32 | 17 | 9 | 58 |
|  | Strongly Agree | 14 | 19 | 3 | 36 |
| Total | | 102 | 76 | 33 | 211 |

| **M-Health innovations present more risks than they offer opportunities * Category of hospital classification Crosstabulation** | | | | | |
| --- | --- | --- | --- | --- | --- |
| Count | | | | | |
|  | | Category of hospital classification | | | Total |
|  |  | Public Hospital | Private Hospital | Faith-Based Hospital/NGO |  |
| M-Health innovations present more risks than they offer opportunities | Strongly disagree | 44 | 38 | 10 | 92 |
|  | Disagree | 42 | 25 | 18 | 85 |
|  | Agree | 11 | 9 | 0 | 20 |
|  | Strongly Agree | 5 | 2 | 5 | 12 |
| Total | | 102 | 74 | 33 | 209 |

1. **This section provides statements that best describe the leadership style used by boards or oversight committees of hospitals. Please rate the extent to which your board or similar oversight bodies exhibit the following leadership styles/characteristics using a 7 point likert scale (1=never to 7= always)**

MODERATING FACTORS OF LEADERSHIP STYLE OF OVERSIGHT MANAGEMENT BODIES

| 1) Idealized influence – **The leadership of my board fosters trust, involvement and cooperation. * Category of hospital classification Crosstabulation** | | | | | |
| --- | --- | --- | --- | --- | --- |
| Count | | | | | |
|  | | Category of hospital classification | | | Total |
|  |  | Public Hospital | Private Hospital | Faith-Based Hospital/NGO |  |
| The leadership of my board fosters trust, involvement and cooperation. | Never | 3 | 0 | 0 | 3 |
|  | Almost Never | 2 | 2 | 0 | 4 |
|  | Moderately | 5 | 3 | 0 | 8 |
|  | Frequently | 13 | 3 | 3 | 19 |
|  | Very frequently | 19 | 11 | 6 | 36 |
|  | Usually | 21 | 13 | 8 | 42 |
|  | Always | 38 | 44 | 16 | 98 |
| Total | | 101 | 76 | 33 | 210 |

| **Crosstab** | | | | | | | |
| --- | --- | --- | --- | --- | --- | --- | --- |
| Count | | | | | | | |
|  | | The leadership of my board fosters trust, involvement and cooperation. | | | | | Total |
|  |  | Never | Rarely | Frequently | Very frequently | Always |  |
| Classification level of facility | Level IV | 3 | 10 | 14 | 30 | 113 | 170 |
|  | Level V | 1 | 2 | 2 | 6 | 22 | 33 |
|  | Level VI | 0 | 0 | 3 | 0 | 5 | 8 |
| Total | | 4 | 12 | 19 | 36 | 140 | 211 |

| **Chi-Square Tests** | | | |
| --- | --- | --- | --- |
|  | Value | df | Asymptotic Significance (2-sided) |
| Pearson Chi-Square | 9.951^a^ | 8 | .268 |
| Likelihood Ratio | 8.719 | 8 | .367 |
| Linear-by-Linear Association | .141 | 1 | .708 |
| N of Valid Cases | 211 |  |  |
| a. 8 cells (53.3%) have expected count less than 5. The minimum expected count is .15. | | | |

**Category of hospital classification * The leadership of my board fosters trust, involvement and cooperation.**

| **Crosstab** | | | | | | | |
| --- | --- | --- | --- | --- | --- | --- | --- |
| Count | | | | | | | |
|  | | The leadership of my board fosters trust, involvement and cooperation. | | | | | Total |
|  |  | Never | Rarely | Frequently | Very frequently | Always |  |
| Category of hospital classification | Public Hospital | 4 | 7 | 13 | 19 | 59 | 102 |
|  | Private Hospital | 0 | 5 | 3 | 11 | 57 | 76 |
|  | Faith-Based Hospital/NGO | 0 | 0 | 3 | 6 | 24 | 33 |
| Total | | 4 | 12 | 19 | 36 | 140 | 211 |

| **Chi-Square Tests** | | | |
| --- | --- | --- | --- |
|  | Value | df | Asymptotic Significance (2-sided) |
| Pearson Chi-Square | 12.890^a^ | 8 | .116 |
| Likelihood Ratio | 16.706 | 8 | .033 |
| Linear-by-Linear Association | 7.419 | 1 | .006 |
| N of Valid Cases | 211 |  |  |
| a. 6 cells (40.0%) have expected count less than 5. The minimum expected count is .63. | | | |

| **The leadership of my board instills pride and respect in others * Category of hospital classification Crosstabulation** | | | | | |
| --- | --- | --- | --- | --- | --- |
| Count | | | | | |
|  | | Category of hospital classification | | | Total |
|  |  | Public Hospital | Private Hospital | Faith-Based Hospital/NGO |  |
| The leadership of my board instills pride and respect in others | Never | 6 | 2 | 2 | 10 |
|  | Almost Never | 4 | 1 | 0 | 5 |
|  | Moderately | 6 | 2 | 0 | 8 |
|  | Frequently | 10 | 6 | 0 | 16 |
|  | Very frequently | 19 | 11 | 7 | 37 |
|  | Usually | 25 | 10 | 9 | 44 |
|  | Always | 30 | 44 | 15 | 89 |
| Total | | 100 | 76 | 33 | 209 |

| **Crosstab** | | | | | | | |
| --- | --- | --- | --- | --- | --- | --- | --- |
| Count | | | | | | | |
|  | | The leadership of my board instills pride and respect in others | | | | | Total |
|  |  | Never | Rarely | Frequently | Very frequently | Always |  |
| Classification level of facility | Level IV | 10 | 12 | 13 | 29 | 105 | 169 |
|  | Level V | 1 | 1 | 2 | 5 | 24 | 33 |
|  | Level VI | 0 | 0 | 1 | 3 | 4 | 8 |
| Total | | 11 | 13 | 16 | 37 | 133 | 210 |

| **Chi-Square Tests** | | | |
| --- | --- | --- | --- |
|  | Value | df | Asymptotic Significance (2-sided) |
| Pearson Chi-Square | 5.137^a^ | 8 | .743 |
| Likelihood Ratio | 5.741 | 8 | .676 |
| Linear-by-Linear Association | 1.231 | 1 | .267 |
| N of Valid Cases | 210 |  |  |
| a. 7 cells (46.7%) have expected count less than 5. The minimum expected count is .42. | | | |

**Category of hospital classification * The leadership of my board instills pride and respect in others**

| **Crosstab** | | | | | | | |
| --- | --- | --- | --- | --- | --- | --- | --- |
| Count | | | | | | | |
|  | | The leadership of my board instills pride and respect in others | | | | | Total |
|  |  | Never | Rarely | Frequently | Very frequently | Always |  |
| Category of hospital classification | Public Hospital | 7 | 10 | 10 | 19 | 55 | 101 |
|  | Private Hospital | 2 | 3 | 6 | 11 | 54 | 76 |
|  | Faith-Based Hospital/NGO | 2 | 0 | 0 | 7 | 24 | 33 |
| Total | | 11 | 13 | 16 | 37 | 133 | 210 |

| **Chi-Square Tests** | | | |
| --- | --- | --- | --- |
|  | Value | df | Asymptotic Significance (2-sided) |
| Pearson Chi-Square | 12.882^a^ | 8 | .116 |
| Likelihood Ratio | 17.317 | 8 | .027 |
| Linear-by-Linear Association | 7.062 | 1 | .008 |
| N of Valid Cases | 210 |  |  |
| a. 5 cells (33.3%) have expected count less than 5. The minimum expected count is 1.73. | | | |

1) Idealized influence –

| **The leadership of my board practices what it preaches * Category of hospital classification Crosstabulation** | | | | | |
| --- | --- | --- | --- | --- | --- |
| Count | | | | | |
|  | | Category of hospital classification | | | Total |
|  |  | Public Hospital | Private Hospital | Faith-Based Hospital/NGO |  |
| The leadership of my board practices what it preaches | Never | 4 | 1 | 0 | 5 |
|  | Almost Never | 2 | 3 | 0 | 5 |
|  | Moderately | 5 | 2 | 1 | 8 |
|  | Frequently | 13 | 5 | 3 | 21 |
|  | Very frequently | 15 | 3 | 5 | 23 |
|  | Usually | 27 | 15 | 9 | 51 |
|  | Always | 32 | 46 | 14 | 92 |
| Total | | 98 | 75 | 32 | 205 |

| **Crosstab** | | | | | | | |
| --- | --- | --- | --- | --- | --- | --- | --- |
| Count | | | | | | | |
|  | | The leadership of my board practices what it preaches | | | | | Total |
|  |  | Never | Rarely | Frequently | Very frequently | Always |  |
| Classification level of facility | Level IV | 5 | 11 | 17 | 19 | 114 | 166 |
|  | Level V | 1 | 2 | 1 | 4 | 24 | 32 |
|  | Level VI | 0 | 0 | 3 | 0 | 5 | 8 |
| Total | | 6 | 13 | 21 | 23 | 143 | 206 |

| **Chi-Square Tests** | | | |
| --- | --- | --- | --- |
|  | Value | df | Asymptotic Significance (2-sided) |
| Pearson Chi-Square | 9.363^a^ | 8 | .313 |
| Likelihood Ratio | 9.176 | 8 | .328 |
| Linear-by-Linear Association | .054 | 1 | .817 |
| N of Valid Cases | 206 |  |  |
| a. 9 cells (60.0%) have expected count less than 5. The minimum expected count is .23. | | | |

**Category of hospital classification * The leadership of my board practices what it preaches**

| **Crosstab** | | | | | | | |
| --- | --- | --- | --- | --- | --- | --- | --- |
| Count | | | | | | | |
|  | | The leadership of my board practices what it preaches | | | | | Total |
|  |  | Never | Rarely | Frequently | Very frequently | Always |  |
| Category of hospital classification | Public Hospital | 5 | 7 | 13 | 15 | 59 | 99 |
|  | Private Hospital | 1 | 5 | 5 | 3 | 61 | 75 |
|  | Faith-Based Hospital/NGO | 0 | 1 | 3 | 5 | 23 | 32 |
| Total | | 6 | 13 | 21 | 23 | 143 | 206 |

| **Chi-Square Tests** | | | |
| --- | --- | --- | --- |
|  | Value | df | Asymptotic Significance (2-sided) |
| Pearson Chi-Square | 13.888^a^ | 8 | .085 |
| Likelihood Ratio | 15.797 | 8 | .045 |
| Linear-by-Linear Association | 5.428 | 1 | .020 |
| N of Valid Cases | 206 |  |  |
| a. 7 cells (46.7%) have expected count less than 5. The minimum expected count is .93. | | | |

2) Inspirational motivation

| **The leadership of my board encourages thinking about problems in new ways and questions assumptions * Category of hospital classification Crosstabulation** | | | | | |
| --- | --- | --- | --- | --- | --- |
| Count | | | | | |
|  | | Category of hospital classification | | | Total |
|  |  | Public Hospital | Private Hospital | Faith-Based Hospital/NGO |  |
| The leadership of my board encourages thinking about problems in new ways and questions assumptions | Never | 3 | 1 | 0 | 4 |
|  | Almost Never | 2 | 2 | 0 | 4 |
|  | Moderately | 10 | 3 | 2 | 15 |
|  | Frequently | 12 | 2 | 0 | 14 |
|  | Very frequently | 12 | 6 | 2 | 20 |
|  | Usually | 26 | 22 | 12 | 60 |
|  | Always | 36 | 40 | 17 | 93 |
| Total | | 101 | 76 | 33 | 210 |

| **Crosstab** | | | | | | | |
| --- | --- | --- | --- | --- | --- | --- | --- |
| Count | | | | | | | |
|  | | The leadership of my board encourages thinking about problems in new ways and questions assumptions | | | | | Total |
|  |  | Never | Rarely | Frequently | Very frequently | Always |  |
| Classification level of facility | Level IV | 4 | 16 | 13 | 16 | 121 | 170 |
|  | Level V | 1 | 2 | 1 | 4 | 25 | 33 |
|  | Level VI | 0 | 1 | 0 | 0 | 7 | 8 |
| Total | | 5 | 19 | 14 | 20 | 153 | 211 |

| **Chi-Square Tests** | | | |
| --- | --- | --- | --- |
|  | Value | df | Asymptotic Significance (2-sided) |
| Pearson Chi-Square | 3.487^a^ | 8 | .900 |
| Likelihood Ratio | 5.108 | 8 | .746 |
| Linear-by-Linear Association | .765 | 1 | .382 |
| N of Valid Cases | 211 |  |  |
| a. 9 cells (60.0%) have expected count less than 5. The minimum expected count is .19. | | | |

**Category of hospital classification * The leadership of my board encourages thinking about problems in new ways and questions assumptions**

| **Crosstab** | | | | | | | |
| --- | --- | --- | --- | --- | --- | --- | --- |
| Count | | | | | | | |
|  | | The leadership of my board encourages thinking about problems in new ways and questions assumptions | | | | | Total |
|  |  | Never | Rarely | Frequently | Very frequently | Always |  |
| Category of hospital classification | Public Hospital | 4 | 12 | 12 | 12 | 62 | 102 |
|  | Private Hospital | 1 | 5 | 2 | 6 | 62 | 76 |
|  | Faith-Based Hospital/NGO | 0 | 2 | 0 | 2 | 29 | 33 |
| Total | | 5 | 19 | 14 | 20 | 153 | 211 |

| **Chi-Square Tests** | | | |
| --- | --- | --- | --- |
|  | Value | df | Asymptotic Significance (2-sided) |
| Pearson Chi-Square | 16.964^a^ | 8 | .030 |
| Likelihood Ratio | 19.678 | 8 | .012 |
| Linear-by-Linear Association | 11.302 | 1 | .001 |
| N of Valid Cases | 211 |  |  |
| a. 6 cells (40.0%) have expected count less than 5. The minimum expected count is .78. | | | |

3) Individualized considerations

| **The leadership of my board treats me as an individual, supports and encourages my development * Category of hospital classification Crosstabulation** | | | | | |
| --- | --- | --- | --- | --- | --- |
| Count | | | | | |
|  | | Category of hospital classification | | | Total |
|  |  | Public Hospital | Private Hospital | Faith-Based Hospital/NGO |  |
| The leadership of my board treats me as individuals, supports and encourages their development | Never | 4 | 0 | 0 | 4 |
|  | Almost Never | 2 | 0 | 1 | 3 |
|  | Moderately | 8 | 4 | 0 | 12 |
|  | Frequently | 11 | 4 | 0 | 15 |
|  | Very frequently | 12 | 4 | 3 | 19 |
|  | Usually | 20 | 22 | 11 | 53 |
|  | Always | 43 | 42 | 18 | 103 |
| Total | | 100 | 76 | 33 | 209 |

| **Crosstab** | | | | | | | |
| --- | --- | --- | --- | --- | --- | --- | --- |
| Count | | | | | | | |
|  | | The leadership of my board treats me as individuals, supports and encourages their development | | | | | Total |
|  |  | Never | Rarely | Frequently | Very frequently | Always |  |
| Classification level of facility | Level IV | 4 | 14 | 9 | 17 | 125 | 169 |
|  | Level V | 1 | 1 | 6 | 1 | 24 | 33 |
|  | Level VI | 0 | 0 | 0 | 1 | 7 | 8 |
| Total | | 5 | 15 | 15 | 19 | 156 | 210 |

| **Chi-Square Tests** | | | |
| --- | --- | --- | --- |
|  | Value | df | Asymptotic Significance (2-sided) |
| Pearson Chi-Square | 10.711^a^ | 8 | .219 |
| Likelihood Ratio | 11.050 | 8 | .199 |
| Linear-by-Linear Association | .356 | 1 | .551 |
| N of Valid Cases | 210 |  |  |
| a. 9 cells (60.0%) have expected count less than 5. The minimum expected count is .19. | | | |

**Category of hospital classification * The leadership of my board treats me as individuals, supports and encourages their development**

| **Crosstab** | | | | | | | |
| --- | --- | --- | --- | --- | --- | --- | --- |
| Count | | | | | | | |
|  | | The leadership of my board treats me as individuals, supports and encourages their development | | | | | Total |
|  |  | Never | Rarely | Frequently | Very frequently | Always |  |
| Category of hospital classification | Public Hospital | 5 | 10 | 11 | 12 | 63 | 101 |
|  | Private Hospital | 0 | 4 | 4 | 4 | 64 | 76 |
|  | Faith-Based Hospital/NGO | 0 | 1 | 0 | 3 | 29 | 33 |
| Total | | 5 | 15 | 15 | 19 | 156 | 210 |

| **Chi-Square Tests** | | | |
| --- | --- | --- | --- |
|  | Value | df | Asymptotic Significance (2-sided) |
| Pearson Chi-Square | 18.205^a^ | 8 | .020 |
| Likelihood Ratio | 22.544 | 8 | .004 |
| Linear-by-Linear Association | 13.573 | 1 | .000 |
| N of Valid Cases | 210 |  |  |
| a. 6 cells (40.0%) have expected count less than 5. The minimum expected count is .79. | | | |

| **The leadership of my board is clear about organizational values and communicates them to me and staff * Category of hospital classification Crosstabulation** | | | | | |
| --- | --- | --- | --- | --- | --- |
| Count | | | | | |
|  | | Category of hospital classification | | | Total |
|  |  | Public Hospital | Private Hospital | Faith-Based Hospital/NGO |  |
| The leadership of my board is clear about organizational values and communicates them to me and staff | Never | 3 | 0 | 0 | 3 |
|  | Almost Never | 1 | 0 | 0 | 1 |
|  | Moderately | 8 | 1 | 0 | 9 |
|  | Frequently | 13 | 5 | 0 | 18 |
|  | Very frequently | 17 | 5 | 7 | 29 |
|  | Usually | 19 | 15 | 13 | 47 |
|  | Always | 40 | 50 | 13 | 103 |
| Total | | 101 | 76 | 33 | 210 |

| **Crosstab** | | | | | | | |
| --- | --- | --- | --- | --- | --- | --- | --- |
| Count | | | | | | | |
|  | | The leadership of my board is clear about organizational values and communicates them to me and staff | | | | | Total |
|  |  | Never | Rarely | Frequently | Very frequently | Always |  |
| Classification level of facility | Level IV | 3 | 10 | 14 | 20 | 123 | 170 |
|  | Level V | 1 | 0 | 3 | 9 | 20 | 33 |
|  | Level VI | 0 | 0 | 1 | 0 | 7 | 8 |
| Total | | 4 | 10 | 18 | 29 | 150 | 211 |

| **Chi-Square Tests** | | | |
| --- | --- | --- | --- |
|  | Value | df | Asymptotic Significance (2-sided) |
| Pearson Chi-Square | 9.810^a^ | 8 | .279 |
| Likelihood Ratio | 11.960 | 8 | .153 |
| Linear-by-Linear Association | .154 | 1 | .695 |
| N of Valid Cases | 211 |  |  |
| a. 9 cells (60.0%) have expected count less than 5. The minimum expected count is .15. | | | |

**Category of hospital classification * The leadership of my board is clear about organizational values and communicates them to me and staff**

| **Crosstab** | | | | | | | |
| --- | --- | --- | --- | --- | --- | --- | --- |
| Count | | | | | | | |
|  | | The leadership of my board is clear about organizational values and communicates them to me and staff | | | | | Total |
|  |  | Never | Rarely | Frequently | Very frequently | Always |  |
| Category of hospital classification | Public Hospital | 4 | 9 | 13 | 17 | 59 | 102 |
|  | Private Hospital | 0 | 1 | 5 | 5 | 65 | 76 |
|  | Faith-Based Hospital/NGO | 0 | 0 | 0 | 7 | 26 | 33 |
| Total | | 4 | 10 | 18 | 29 | 150 | 211 |

| **Chi-Square Tests** | | | |
| --- | --- | --- | --- |
|  | Value | df | Asymptotic Significance (2-sided) |
| Pearson Chi-Square | 26.419^a^ | 8 | .001 |
| Likelihood Ratio | 32.590 | 8 | .000 |
| Linear-by-Linear Association | 17.322 | 1 | .000 |
| N of Valid Cases | 211 |  |  |
| a. 8 cells (53.3%) have expected count less than 5. The minimum expected count is .63. | | | |

| **The leadership of my board gives me encouragement and recognition * Category of hospital classification Crosstabulation** | | | | | |
| --- | --- | --- | --- | --- | --- |
| Count | | | | | |
|  | | Category of hospital classification | | | Total |
|  |  | Public Hospital | Private Hospital | Faith-Based Hospital/NGO |  |
| The leadership of my board gives me encouragement and recognition | Never | 4 | 0 | 0 | 4 |
|  | Almost Never | 2 | 3 | 0 | 5 |
|  | Moderately | 9 | 0 | 0 | 9 |
|  | Frequently | 12 | 5 | 3 | 20 |
|  | Very frequently | 13 | 4 | 2 | 19 |
|  | Usually | 18 | 20 | 10 | 48 |
|  | Always | 43 | 44 | 18 | 105 |
| Total | | 101 | 76 | 33 | 210 |

| **Crosstab** | | | | | | | |
| --- | --- | --- | --- | --- | --- | --- | --- |
| Count | | | | | | | |
|  | | The leadership of my board gives me encouragement and recognition | | | | | Total |
|  |  | Never | Rarely | Frequently | Very frequently | Always |  |
| Classification level of facility | Level IV | 4 | 10 | 18 | 14 | 124 | 170 |
|  | Level V | 1 | 4 | 2 | 2 | 24 | 33 |
|  | Level VI | 0 | 0 | 0 | 3 | 5 | 8 |
| Total | | 5 | 14 | 20 | 19 | 153 | 211 |

| **Chi-Square Tests** | | | |
| --- | --- | --- | --- |
|  | Value | df | Asymptotic Significance (2-sided) |
| Pearson Chi-Square | 11.568^a^ | 8 | .172 |
| Likelihood Ratio | 9.639 | 8 | .291 |
| Linear-by-Linear Association | .000 | 1 | .990 |
| N of Valid Cases | 211 |  |  |
| a. 9 cells (60.0%) have expected count less than 5. The minimum expected count is .19. | | | |

**Category of hospital classification * The leadership of my board gives me encouragement and recognition**

| **Crosstab** | | | | | | | |
| --- | --- | --- | --- | --- | --- | --- | --- |
| Count | | | | | | | |
|  | | The leadership of my board gives me encouragement and recognition | | | | | Total |
|  |  | Never | Rarely | Frequently | Very frequently | Always |  |
| Category of hospital classification | Public Hospital | 5 | 11 | 12 | 13 | 61 | 102 |
|  | Private Hospital | 0 | 3 | 5 | 4 | 64 | 76 |
|  | Faith-Based Hospital/NGO | 0 | 0 | 3 | 2 | 28 | 33 |
| Total | | 5 | 14 | 20 | 19 | 153 | 211 |

| **Chi-Square Tests** | | | |
| --- | --- | --- | --- |
|  | Value | df | Asymptotic Significance (2-sided) |
| Pearson Chi-Square | 19.731^a^ | 8 | .011 |
| Likelihood Ratio | 23.711 | 8 | .003 |
| Linear-by-Linear Association | 14.272 | 1 | .000 |
| N of Valid Cases | 211 |  |  |
| a. 6 cells (40.0%) have expected count less than 5. The minimum expected count is .78. | | | |

4) Intellectual stimulation

| **The leadership of my board communicates a clear and positive vision of the future * Category of hospital classification Crosstabulation** | | | | | |
| --- | --- | --- | --- | --- | --- |
| Count | | | | | |
|  | | Category of hospital classification | | | Total |
|  |  | Public Hospital | Private Hospital | Faith-Based Hospital/NGO |  |
| The leadership of my board communicates a clear and positive vision of the future | Never | 3 | 1 | 0 | 4 |
|  | Almost Never | 5 | 0 | 0 | 5 |
|  | Moderately | 6 | 1 | 0 | 7 |
|  | Frequently | 10 | 3 | 0 | 13 |
|  | Very frequently | 14 | 3 | 3 | 20 |
|  | Usually | 22 | 21 | 13 | 56 |
|  | Always | 41 | 46 | 17 | 104 |
| Total | | 101 | 75 | 33 | 209 |

| **Crosstab** | | | | | | | |
| --- | --- | --- | --- | --- | --- | --- | --- |
| Count | | | | | | | |
|  | | The leadership of my board communicates a clear and positive vision of the future | | | | | Total |
|  |  | Never | Rarely | Frequently | Very frequently | Always |  |
| Classification level of facility | Level IV | 4 | 10 | 11 | 16 | 128 | 169 |
|  | Level V | 1 | 2 | 1 | 4 | 25 | 33 |
|  | Level VI | 0 | 0 | 1 | 0 | 7 | 8 |
| Total | | 5 | 12 | 13 | 20 | 160 | 210 |

| **Chi-Square Tests** | | | |
| --- | --- | --- | --- |
|  | Value | df | Asymptotic Significance (2-sided) |
| Pearson Chi-Square | 2.936^a^ | 8 | .938 |
| Likelihood Ratio | 4.321 | 8 | .827 |
| Linear-by-Linear Association | .268 | 1 | .605 |
| N of Valid Cases | 210 |  |  |
| a. 9 cells (60.0%) have expected count less than 5. The minimum expected count is .19. | | | |

**Category of hospital classification * The leadership of my board communicates a clear and positive vision of the future**

| **Crosstab** | | | | | | | |
| --- | --- | --- | --- | --- | --- | --- | --- |
| Count | | | | | | | |
|  | | The leadership of my board communicates a clear and positive vision of the future | | | | | Total |
|  |  | Never | Rarely | Frequently | Very frequently | Always |  |
| Category of hospital classification | Public Hospital | 4 | 11 | 10 | 14 | 63 | 102 |
|  | Private Hospital | 1 | 1 | 3 | 3 | 67 | 75 |
|  | Faith-Based Hospital/NGO | 0 | 0 | 0 | 3 | 30 | 33 |
| Total | | 5 | 12 | 13 | 20 | 160 | 210 |

| **Chi-Square Tests** | | | |
| --- | --- | --- | --- |
|  | Value | df | Asymptotic Significance (2-sided) |
| Pearson Chi-Square | 25.642^a^ | 8 | .001 |
| Likelihood Ratio | 30.692 | 8 | .000 |
| Linear-by-Linear Association | 19.104 | 1 | .000 |
| N of Valid Cases | 210 |  |  |
| a. 8 cells (53.3%) have expected count less than 5. The minimum expected count is .79. | | | |

| **The leadership of my board inspires by being highly competent * Category of hospital classification Crosstabulation** | | | | | |
| --- | --- | --- | --- | --- | --- |
| Count | | | | | |
|  | | Category of hospital classification | | | Total |
|  |  | Public Hospital | Private Hospital | Faith-Based Hospital/NGO |  |
| The leadership of my board inspires by being highly competent | Never | 4 | 1 | 0 | 5 |
|  | Almost Never | 3 | 1 | 0 | 4 |
|  | Moderately | 5 | 0 | 0 | 5 |
|  | Frequently | 20 | 1 | 0 | 21 |
|  | Very frequently | 10 | 8 | 6 | 24 |
|  | Usually | 17 | 18 | 12 | 47 |
|  | Always | 42 | 46 | 15 | 103 |
| Total | | 101 | 75 | 33 | 209 |

| **Crosstab** | | | | | | | |
| --- | --- | --- | --- | --- | --- | --- | --- |
| Count | | | | | | | |
|  | | The leadership of my board inspires by being highly competent | | | | | Total |
|  |  | Never | Rarely | Frequently | Very frequently | Always |  |
| Classification level of facility | Level IV | 5 | 8 | 17 | 15 | 124 | 169 |
|  | Level V | 1 | 1 | 4 | 5 | 22 | 33 |
|  | Level VI | 0 | 0 | 0 | 4 | 4 | 8 |
| Total | | 6 | 9 | 21 | 24 | 150 | 210 |

| **Chi-Square Tests** | | | |
| --- | --- | --- | --- |
|  | Value | df | Asymptotic Significance (2-sided) |
| Pearson Chi-Square | 14.217^a^ | 8 | .076 |
| Likelihood Ratio | 10.918 | 8 | .206 |
| Linear-by-Linear Association | .009 | 1 | .926 |
| N of Valid Cases | 210 |  |  |
| a. 9 cells (60.0%) have expected count less than 5. The minimum expected count is .23. | | | |

**Category of hospital classification * The leadership of my board inspires by being highly competent**

| **Crosstab** | | | | | | | |
| --- | --- | --- | --- | --- | --- | --- | --- |
| Count | | | | | | | |
|  | | The leadership of my board inspires by being highly competent | | | | | Total |
|  |  | Never | Rarely | Frequently | Very frequently | Always |  |
| Category of hospital classification | Public Hospital | 5 | 8 | 20 | 10 | 59 | 102 |
|  | Private Hospital | 1 | 1 | 1 | 8 | 64 | 75 |
|  | Faith-Based Hospital/NGO | 0 | 0 | 0 | 6 | 27 | 33 |
| Total | | 6 | 9 | 21 | 24 | 150 | 210 |

| **Chi-Square Tests** | | | |
| --- | --- | --- | --- |
|  | Value | df | Asymptotic Significance (2-sided) |
| Pearson Chi-Square | 34.101^a^ | 8 | .000 |
| Likelihood Ratio | 40.720 | 8 | .000 |
| Linear-by-Linear Association | 20.788 | 1 | .000 |
| N of Valid Cases | 210 |  |  |
| a. 8 cells (53.3%) have expected count less than 5. The minimum expected count is .94. | | | |

KEY RECOMMENDATIONS

1. **What are your recommendations for ensuring adoption of m-health innovations in Kenya? (Please, be as specific and concise as possible)**

| **Training * Category of hospital classification Crosstabulation** | | | | | |
| --- | --- | --- | --- | --- | --- |
| Count | | | | | |
|  | | Category of hospital classification | | | Total |
|  |  | Public Hospital | Private Hospital | Faith-Based Hospital/NGO |  |
| Training | No | 87 | 67 | 32 | 186 |
|  | Yes | 11 | 7 | 1 | 19 |
| Total | | 98 | 74 | 33 | 205 |

| **Community/stakeholder mobilization * Category of hospital classification Crosstabulation** | | | | | |
| --- | --- | --- | --- | --- | --- |
| Count | | | | | |
|  | | Category of hospital classification | | | Total |
|  |  | Public Hospital | Private Hospital | Faith-Based Hospital/NGO |  |
| Community/stakeholder mobilization | No | 90 | 66 | 26 | 182 |
|  | Yes | 8 | 8 | 7 | 23 |
| Total | | 98 | 74 | 33 | 205 |

| **Strengthening of e-government government wide * Category of hospital classification Crosstabulation** | | | | | |
| --- | --- | --- | --- | --- | --- |
| Count | | | | | |
|  | | Category of hospital classification | | | Total |
|  |  | Public Hospital | Private Hospital | Faith-Based Hospital/NGO |  |
| Strengthening of e-government government wide | No | 90 | 74 | 33 | 197 |
|  | Yes | 8 | 0 | 0 | 8 |
| Total | | 98 | 74 | 33 | 205 |

| **Support of top leadership * Category of hospital classification Crosstabulation** | | | | | |
| --- | --- | --- | --- | --- | --- |
| Count | | | | | |
|  | | Category of hospital classification | | | Total |
|  |  | Public Hospital | Private Hospital | Faith-Based Hospital/NGO |  |
| Support of top leadership | No | 93 | 72 | 33 | 198 |
|  | Yes | 5 | 2 | 0 | 7 |
| Total | | 98 | 74 | 33 | 205 |

| **Establish m-health compliance regulation and guidelines * Category of hospital classification Crosstabulation** | | | | | |
| --- | --- | --- | --- | --- | --- |
| Count | | | | | |
|  | | Category of hospital classification | | | Total |
|  |  | Public Hospital | Private Hospital | Faith-Based Hospital/NGO |  |
| Establish m-health compliance regulation and guidelines | No | 95 | 71 | 33 | 199 |
|  | Yes | 3 | 3 | 0 | 6 |
| Total | | 98 | 74 | 33 | 205 |

| **Piloting m-health * Category of hospital classification Crosstabulation** | | | | | |
| --- | --- | --- | --- | --- | --- |
| Count | | | | | |
|  | | Category of hospital classification | | | Total |
|  |  | Public Hospital | Private Hospital | Faith-Based Hospital/NGO |  |
| Piloting m-health | No | 92 | 70 | 32 | 194 |
|  | Yes | 6 | 4 | 1 | 11 |
| Total | | 98 | 74 | 33 | 205 |

| **Confidentiality issues addressed * Category of hospital classification Crosstabulation** | | | | | |
| --- | --- | --- | --- | --- | --- |
| Count | | | | | |
|  | | Category of hospital classification | | | Total |
|  |  | Public Hospital | Private Hospital | Faith-Based Hospital/NGO |  |
| Confidentiality issues addressed | No | 97 | 71 | 31 | 199 |
|  | Yes | 1 | 3 | 2 | 6 |
| Total | | 98 | 74 | 33 | 205 |

| **GoK to envest more in e-health infrastructure /NHIF * Category of hospital classification Crosstabulation** | | | | | |
| --- | --- | --- | --- | --- | --- |
| Count | | | | | |
|  | | Category of hospital classification | | | Total |
|  |  | Public Hospital | Private Hospital | Faith-Based Hospital/NGO |  |
| GoK to envest more in e-health infrastructure /NHIF | No | 90 | 68 | 32 | 190 |
|  | Yes | 8 | 5 | 1 | 14 |
|  | 8 | 0 | 1 | 0 | 1 |
| Total | | 98 | 74 | 33 | 205 |

| **Implementation and adoption * Category of hospital classification Crosstabulation** | | | | | |
| --- | --- | --- | --- | --- | --- |
| Count | | | | | |
|  | | Category of hospital classification | | | Total |
|  |  | Public Hospital | Private Hospital | Faith-Based Hospital/NGO |  |
| Implementation and adoption | No | 73 | 67 | 25 | 165 |
|  | Yes | 25 | 7 | 8 | 40 |
| Total | | 98 | 74 | 33 | 205 |

| **Financial support' * Category of hospital classification Crosstabulation** | | | | | |
| --- | --- | --- | --- | --- | --- |
| Count | | | | | |
|  | | Category of hospital classification | | | Total |
|  |  | Public Hospital | Private Hospital | Faith-Based Hospital/NGO |  |
| Financial support' | No | 94 | 72 | 32 | 198 |
|  | Yes | 4 | 2 | 1 | 7 |
| Total | | 98 | 74 | 33 | 205 |

| **PPP strategy * Category of hospital classification Crosstabulation** | | | | | |
| --- | --- | --- | --- | --- | --- |
| Count | | | | | |
|  | | Category of hospital classification | | | Total |
|  |  | Public Hospital | Private Hospital | Faith-Based Hospital/NGO |  |
| PPP strategy | No | 97 | 74 | 33 | 204 |
|  | Yes | 1 | 0 | 0 | 1 |
| Total | | 98 | 74 | 33 | 205 |

| **Building of staff capacity at all levels * Category of hospital classification Crosstabulation** | | | | | |
| --- | --- | --- | --- | --- | --- |
| Count | | | | | |
|  | | Category of hospital classification | | | Total |
|  |  | Public Hospital | Private Hospital | Faith-Based Hospital/NGO |  |
| Building of staff capacity at all levels | No | 95 | 74 | 32 | 201 |
|  | Yes | 3 | 0 | 1 | 4 |
| Total | | 98 | 74 | 33 | 205 |
