# Supplementary material for: The technological, organizational and environmental determinants of adoption of mobile health applications (m-health) by hospitals in Kenya
Source: PLoS One. 2019 Dec 13;14(12):e0225167. doi: 10.1371/journal.pone.0225167 (PMC6910672; doi:10.1371/journal.pone.0225167)
Supplement: S3 File — (DOCX) [file pone.0225167.s003.docx]

**Technological determinants of M-health adoption by hospitals in Kenya**

1. *Health call centers/telephone help line and technological determinants*

**Logistic Regression**

| **Case Processing Summary** | | | |
| --- | --- | --- | --- |
| Unweighted Cases^a^ | | N | Percent |
| Selected Cases | Included in Analysis | 167 | 79.1 |
|  | Missing Cases | 44 | 20.9 |
|  | Total | 211 | 100.0 |
| Unselected Cases | | 0 | .0 |
| Total | | 211 | 100.0 |
| a. If weight is in effect, see classification table for the total number of cases. | | | |

| **Dependent Variable Encoding** | |
| --- | --- |
| Original Value | Internal Value |
| Not adopted | 0 |
| Adopted | 1 |

| **Categorical Variables Codings** | | | | | |
| --- | --- | --- | --- | --- | --- |
|  | | Frequency | Parameter coding | | |
|  |  |  | (1) | (2) | (3) |
| It is more strategic to adopt M-Health when the hospital fully owned the technology | Strongly disgree | 14 | 1.000 | .000 | .000 |
|  | Disagree | 24 | .000 | 1.000 | .000 |
|  | Agree | 61 | .000 | .000 | 1.000 |
|  | Strongly Agree | 68 | .000 | .000 | .000 |
| M-Health improves efficiency of hospital’s operations | Strongly disgree | 6 | 1.000 | .000 | .000 |
|  | Disagree | 8 | .000 | 1.000 | .000 |
|  | Agree | 63 | .000 | .000 | 1.000 |
|  | Strongly Agree | 90 | .000 | .000 | .000 |
| M-health will reduce the cost of health care to patients | Strongly disgree | 8 | 1.000 | .000 | .000 |
|  | Disagree | 24 | .000 | 1.000 | .000 |
|  | Agree | 57 | .000 | .000 | 1.000 |
|  | Strongly Agree | 78 | .000 | .000 | .000 |
| M-Health is compatible with current hospital’s health information system and consistent with its values and needs. | Strongly disgree | 16 | 1.000 | .000 | .000 |
|  | Disagree | 29 | .000 | 1.000 | .000 |
|  | Agree | 66 | .000 | .000 | 1.000 |
|  | Strongly Agree | 56 | .000 | .000 | .000 |
| M-Health can be trusted in terms of its security and confidentiality of patient information | Strongly disgree | 10 | 1.000 | .000 | .000 |
|  | Disagree | 30 | .000 | 1.000 | .000 |
|  | Agree | 83 | .000 | .000 | 1.000 |
|  | Strongly Agree | 44 | .000 | .000 | .000 |
| M-Health will make many key staff redundant | Strongly disgree | 44 | 1.000 | .000 | .000 |
|  | Disagree | 66 | .000 | 1.000 | .000 |
|  | Agree | 37 | .000 | .000 | 1.000 |
|  | Strongly Agree | 20 | .000 | .000 | .000 |
| M-Health is difficult to understand, use and integrate in the hospital systems and operations | Strongly disgree | 53 | 1.000 | .000 | .000 |
|  | Disagree | 76 | .000 | 1.000 | .000 |
|  | Agree | 31 | .000 | .000 | 1.000 |
|  | Strongly Agree | 7 | .000 | .000 | .000 |
| It is more strategic to adopt M-Health when the technology is leased to the hospital | Strongly disgree | 35 | 1.000 | .000 | .000 |
|  | Disagree | 65 | .000 | 1.000 | .000 |
|  | Agree | 41 | .000 | .000 | 1.000 |
|  | Strongly Agree | 26 | .000 | .000 | .000 |
| M-Health can be piloted without serious negative impact to patients and hospital operations | Strongly disgree | 6 | 1.000 | .000 | .000 |
|  | Disagree | 26 | .000 | 1.000 | .000 |
|  | Agree | 84 | .000 | .000 | 1.000 |
|  | Strongly Agree | 51 | .000 | .000 | .000 |
| M-Health needs to be piloted first in order to demonstrate that it is better than using current manual systems | Strongly disgree | 17 | 1.000 | .000 | .000 |
|  | Disagree | 20 | .000 | 1.000 | .000 |
|  | Agree | 59 | .000 | .000 | 1.000 |
|  | Strongly Agree | 71 | .000 | .000 | .000 |
| Adoption of M-Health innovations requires exorbitant cost in infrastructure and human resources development | Strongly disgree | 25 | 1.000 | .000 | .000 |
|  | Disagree | 57 | .000 | 1.000 | .000 |
|  | Agree | 60 | .000 | .000 | 1.000 |
|  | Strongly Agree | 25 | .000 | .000 | .000 |
| Use of M-health is superior and more advantageous than current manual practices. | Strongly disgree | 10 | 1.000 | .000 | .000 |
|  | Disagree | 14 | .000 | 1.000 | .000 |
|  | Agree | 66 | .000 | .000 | 1.000 |
|  | Strongly Agree | 77 | .000 | .000 | .000 |

**Block 0: Beginning Block**

| **Classification Table^a,b^** | | | | | |
| --- | --- | --- | --- | --- | --- |
|  | Observed | | Predicted | | |
|  |  |  | Health call centers/telephone help line | | Percentage Correct |
|  |  |  | Not adopted | Adopted |  |
| Step 0 | Health call centers/telephone help line | Not adopted | 0 | 29 | .0 |
|  |  | Adopted | 0 | 138 | 100.0 |
|  | Overall Percentage | |  |  | 82.6 |
| a. Constant is included in the model. | | | | | |
| b. The cut value is .500 | | | | | |

| **Variables in the Equation** | | | | | | | |
| --- | --- | --- | --- | --- | --- | --- | --- |
|  | | B | S.E. | Wald | df | Sig. | Exp(B) |
| Step 0 | Constant | 1.560 | .204 | 58.316 | 1 | .000 | 4.759 |

| **Variables not in the Equation** | | | | | |
| --- | --- | --- | --- | --- | --- |
|  | | | Score | df | Sig. |
| Step 0 | Variables | Use of M-health is superior and more advantageous than current manual practices. | 11.693 | 3 | .009 |
|  |  | Use of M-health is superior and more advantageous than current manual practices.(1) | .051 | 1 | .821 |
|  |  | Use of M-health is superior and more advantageous than current manual practices.(2) | 11.341 | 1 | .001 |
|  |  | Use of M-health is superior and more advantageous than current manual practices.(3) | .373 | 1 | .542 |
|  |  | M-Health improves efficiency of hospital’s operations | 6.649 | 3 | .084 |
|  |  | M-Health improves efficiency of hospital’s operations (1) | .002 | 1 | .963 |
|  |  | M-Health improves efficiency of hospital’s operations (2) | .139 | 1 | .710 |
|  |  | M-Health improves efficiency of hospital’s operations (3) | 6.523 | 1 | .011 |
|  |  | M-health will reduce the cost of health care to patients | 12.780 | 3 | .005 |
|  |  | M-health will reduce the cost of health care to patients(1) | 1.766 | 1 | .184 |
|  |  | M-health will reduce the cost of health care to patients(2) | 11.535 | 1 | .001 |
|  |  | M-health will reduce the cost of health care to patients(3) | .150 | 1 | .699 |
|  |  | M-Health is compatible with current hospital’s health information system and consistent with its values and needs. | 3.771 | 3 | .287 |
|  |  | M-Health is compatible with current hospital’s health information system and consistent with its values and needs.(1) | .719 | 1 | .397 |
|  |  | M-Health is compatible with current hospital’s health information system and consistent with its values and needs.(2) | 2.555 | 1 | .110 |
|  |  | M-Health is compatible with current hospital’s health information system and consistent with its values and needs.(3) | 1.057 | 1 | .304 |
|  |  | M-Health can be trusted in terms of its security and confidentiality of patient information | 2.090 | 3 | .554 |
|  |  | M-Health can be trusted in terms of its security and confidentiality of patient information(1) | 1.183 | 1 | .277 |
|  |  | M-Health can be trusted in terms of its security and confidentiality of patient information(2) | .414 | 1 | .520 |
|  |  | M-Health can be trusted in terms of its security and confidentiality of patient information(3) | .420 | 1 | .517 |
|  |  | M-Health will make many key staff redundant | 1.931 | 3 | .587 |
|  |  | M-Health will make many key staff redundant(1) | .579 | 1 | .447 |
|  |  | M-Health will make many key staff redundant(2) | .373 | 1 | .542 |
|  |  | M-Health will make many key staff redundant(3) | .600 | 1 | .439 |
|  |  | M-Health is difficult to understand, use and integrate in the hospital systems and operations | 5.880 | 3 | .118 |
|  |  | M-Health is difficult to understand, use and integrate in the hospital systems and operations(1) | 3.404 | 1 | .065 |
|  |  | M-Health is difficult to understand, use and integrate in the hospital systems and operations(2) | .007 | 1 | .935 |
|  |  | M-Health is difficult to understand, use and integrate in the hospital systems and operations(3) | 3.611 | 1 | .057 |
|  |  | Adoption of M-Health innovations requires exorbitant cost in infrastructure and human resources development | 2.394 | 3 | .495 |
|  |  | Adoption of M-Health innovations requires exorbitant cost in infrastructure and human resources development(1) | .142 | 1 | .706 |
|  |  | Adoption of M-Health innovations requires exorbitant cost in infrastructure and human resources development(2) | .225 | 1 | .635 |
|  |  | Adoption of M-Health innovations requires exorbitant cost in infrastructure and human resources development(3) | 2.119 | 1 | .145 |
|  |  | M-Health needs to be piloted first in order to demonstrate that it is better than using current manual systems | 3.168 | 3 | .366 |
|  |  | M-Health needs to be piloted first in order to demonstrate that it is better than using current manual systems(1) | .414 | 1 | .520 |
|  |  | M-Health needs to be piloted first in order to demonstrate that it is better than using current manual systems(2) | .923 | 1 | .337 |
|  |  | M-Health needs to be piloted first in order to demonstrate that it is better than using current manual systems(3) | 1.386 | 1 | .239 |
|  |  | M-Health can be piloted without serious negative impact to patients and hospital operations | 2.786 | 3 | .426 |
|  |  | M-Health can be piloted without serious negative impact to patients and hospital operations(1) | .002 | 1 | .963 |
|  |  | M-Health can be piloted without serious negative impact to patients and hospital operations(2) | 1.960 | 1 | .161 |
|  |  | M-Health can be piloted without serious negative impact to patients and hospital operations(3) | .028 | 1 | .866 |
|  |  | It is more strategic to adopt M-Health when the technology is leased to the hospital | 2.622 | 3 | .454 |
|  |  | It is more strategic to adopt M-Health when the technology is leased to the hospital(1) | 1.088 | 1 | .297 |
|  |  | It is more strategic to adopt M-Health when the technology is leased to the hospital(2) | 2.419 | 1 | .120 |
|  |  | It is more strategic to adopt M-Health when the technology is leased to the hospital(3) | .282 | 1 | .595 |
|  |  | It is more strategic to adopt M-Health when the hospital fully owned the technology | 7.045 | 3 | .070 |
|  |  | It is more strategic to adopt M-Health when the hospital fully owned the technology(1) | 1.113 | 1 | .291 |
|  |  | It is more strategic to adopt M-Health when the hospital fully owned the technology(2) | 2.720 | 1 | .099 |
|  |  | It is more strategic to adopt M-Health when the hospital fully owned the technology(3) | 2.089 | 1 | .148 |
|  | Overall Statistics | | 47.732 | 36 | .091 |

**Block 1: Method = Enter**

| **Omnibus Tests of Model Coefficients** | | | | |
| --- | --- | --- | --- | --- |
|  | | Chi-square | df | Sig. |
| Step 1 | Step | 62.279 | 36 | .004 |
|  | Block | 62.279 | 36 | .004 |
|  | Model | 62.279 | 36 | .004 |

| **Model Summary** | | | |
| --- | --- | --- | --- |
| Step | -2 Log likelihood | Cox & Snell R Square | Nagelkerke R Square |
| 1 | 91.905^a^ | .311 | .516 |
| a. Estimation terminated at iteration number 20 because maximum iterations has been reached. Final solution cannot be found. | | | |

| **Hosmer and Lemeshow Test** | | | |
| --- | --- | --- | --- |
| Step | Chi-square | df | Sig. |
| 1 | 4.247 | 8 | .834 |

| **Contingency Table for Hosmer and Lemeshow Test** | | | | | | |
| --- | --- | --- | --- | --- | --- | --- |
|  | | Health call centers/telephone help line = Not adopted | | Health call centers/telephone help line = Adopted | | Total |
|  |  | Observed | Expected | Observed | Expected |  |
| Step 1 | 1 | 12 | 12.489 | 5 | 4.511 | 17 |
|  | 2 | 7 | 7.107 | 10 | 9.893 | 17 |
|  | 3 | 5 | 4.685 | 12 | 12.315 | 17 |
|  | 4 | 3 | 2.544 | 14 | 14.456 | 17 |
|  | 5 | 1 | 1.399 | 16 | 15.601 | 17 |
|  | 6 | 0 | .532 | 17 | 16.468 | 17 |
|  | 7 | 1 | .196 | 16 | 16.804 | 17 |
|  | 8 | 0 | .043 | 17 | 16.957 | 17 |
|  | 9 | 0 | .005 | 17 | 16.995 | 17 |
|  | 10 | 0 | .000 | 14 | 14.000 | 14 |

| **Classification Table^a^** | | | | | |
| --- | --- | --- | --- | --- | --- |
|  | Observed | | Predicted | | |
|  |  |  | Health call centers/telephone help line | | Percentage Correct |
|  |  |  | Not adopted | Adopted |  |
| Step 1 | Health call centers/telephone help line | Not adopted | 12 | 17 | 41.4 |
|  |  | Adopted | 5 | 133 | 96.4 |
|  | Overall Percentage | |  |  | 86.8 |
| a. The cut value is .500 | | | | | |

| **Variables in the Equation** | | | | | | | | | |
| --- | --- | --- | --- | --- | --- | --- | --- | --- | --- |
|  | | B | S.E. | Wald | df | Sig. | Exp(B) | 95% C.I.for EXP(B) | |
|  |  |  |  |  |  |  |  | Lower | Upper |
| Step 1^a^ | Use of M-health is superior and more advantageous than current manual practices. |  |  | 5.362 | 3 | .147 |  |  |  |
|  | Use of M-health is superior and more advantageous than current manual practices.(1) | -3.318 | 1.822 | 3.315 | 1 | .069 | .036 | .001 | 1.289 |
|  | Use of M-health is superior and more advantageous than current manual practices.(2) | -1.897 | 1.675 | 1.283 | 1 | .257 | .150 | .006 | 3.999 |
|  | Use of M-health is superior and more advantageous than current manual practices.(3) | -.434 | 1.057 | .168 | 1 | .681 | .648 | .082 | 5.141 |
|  | M-Health improves efficiency of hospital’s operations |  |  | 5.109 | 3 | .164 |  |  |  |
|  | M-Health improves efficiency of hospital’s operations (1) | -5.577 | 3.577 | 2.432 | 1 | .119 | .004 | .000 | 4.191 |
|  | M-Health improves efficiency of hospital’s operations (2) | 2.801 | 2.483 | 1.273 | 1 | .259 | 16.463 | .127 | 2138.230 |
|  | M-Health improves efficiency of hospital’s operations (3) | -1.343 | 1.064 | 1.592 | 1 | .207 | .261 | .032 | 2.103 |
|  | M-health will reduce the cost of health care to patients |  |  | .661 | 3 | .882 |  |  |  |
|  | M-health will reduce the cost of health care to patients(1) | 27.065 | 9162.819 | .000 | 1 | .998 | 567657574526.095 | .000 | . |
|  | M-health will reduce the cost of health care to patients(2) | .621 | 1.285 | .233 | 1 | .629 | 1.861 | .150 | 23.096 |
|  | M-health will reduce the cost of health care to patients(3) | .861 | 1.062 | .658 | 1 | .417 | 2.366 | .295 | 18.955 |
|  | M-Health is compatible with current hospital’s health information system and consistent with its values and needs. |  |  | 6.277 | 3 | .099 |  |  |  |
|  | M-Health is compatible with current hospital’s health information system and consistent with its values and needs.(1) | -2.167 | 1.327 | 2.666 | 1 | .102 | .115 | .009 | 1.543 |
|  | M-Health is compatible with current hospital’s health information system and consistent with its values and needs.(2) | -1.073 | 1.136 | .893 | 1 | .345 | .342 | .037 | 3.166 |
|  | M-Health is compatible with current hospital’s health information system and consistent with its values and needs.(3) | 1.123 | 1.054 | 1.135 | 1 | .287 | 3.075 | .389 | 24.289 |
|  | M-Health can be trusted in terms of its security and confidentiality of patient information |  |  | 4.247 | 3 | .236 |  |  |  |
|  | M-Health can be trusted in terms of its security and confidentiality of patient information(1) | -.960 | 1.721 | .311 | 1 | .577 | .383 | .013 | 11.167 |
|  | M-Health can be trusted in terms of its security and confidentiality of patient information(2) | 1.558 | 1.347 | 1.339 | 1 | .247 | 4.752 | .339 | 66.586 |
|  | M-Health can be trusted in terms of its security and confidentiality of patient information(3) | -.234 | 1.155 | .041 | 1 | .839 | .791 | .082 | 7.616 |
|  | M-Health will make many key staff redundant |  |  | .488 | 3 | .922 |  |  |  |
|  | M-Health will make many key staff redundant(1) | .735 | 1.160 | .402 | 1 | .526 | 2.085 | .215 | 20.240 |
|  | M-Health will make many key staff redundant(2) | .677 | 1.301 | .271 | 1 | .603 | 1.969 | .154 | 25.238 |
|  | M-Health will make many key staff redundant(3) | .787 | 1.258 | .392 | 1 | .531 | 2.197 | .187 | 25.871 |
|  | M-Health is difficult to understand, use and integrate in the hospital systems and operations |  |  | 9.577 | 3 | .023 |  |  |  |
|  | M-Health is difficult to understand, use and integrate in the hospital systems and operations(1) | 6.931 | 2.486 | 7.771 | 1 | .005 | 1023.092 | 7.829 | 133698.834 |
|  | M-Health is difficult to understand, use and integrate in the hospital systems and operations(2) | 4.981 | 2.311 | 4.646 | 1 | .031 | 145.666 | 1.571 | 13505.183 |
|  | M-Health is difficult to understand, use and integrate in the hospital systems and operations(3) | 4.095 | 2.352 | 3.032 | 1 | .082 | 60.041 | .598 | 6028.787 |
|  | Adoption of M-Health innovations requires exorbitant cost in infrastructure and human resources development |  |  | 11.459 | 3 | .009 |  |  |  |
|  | Adoption of M-Health innovations requires exorbitant cost in infrastructure and human resources development(1) | -2.222 | 1.324 | 2.818 | 1 | .093 | .108 | .008 | 1.451 |
|  | Adoption of M-Health innovations requires exorbitant cost in infrastructure and human resources development(2) | 1.544 | 1.031 | 2.241 | 1 | .134 | 4.682 | .620 | 35.337 |
|  | Adoption of M-Health innovations requires exorbitant cost in infrastructure and human resources development(3) | 3.978 | 1.305 | 9.287 | 1 | .002 | 53.414 | 4.135 | 689.927 |
|  | M-Health needs to be piloted first in order to demonstrate that it is better than using current manual systems |  |  | 3.550 | 3 | .314 |  |  |  |
|  | M-Health needs to be piloted first in order to demonstrate that it is better than using current manual systems(1) | 1.230 | 1.864 | .435 | 1 | .509 | 3.421 | .089 | 132.066 |
|  | M-Health needs to be piloted first in order to demonstrate that it is better than using current manual systems(2) | -1.556 | 1.286 | 1.464 | 1 | .226 | .211 | .017 | 2.624 |
|  | M-Health needs to be piloted first in order to demonstrate that it is better than using current manual systems(3) | -1.308 | 1.063 | 1.513 | 1 | .219 | .270 | .034 | 2.173 |
|  | M-Health can be piloted without serious negative impact to patients and hospital operations |  |  | 3.653 | 3 | .301 |  |  |  |
|  | M-Health can be piloted without serious negative impact to patients and hospital operations(1) | -3.031 | 1.931 | 2.464 | 1 | .116 | .048 | .001 | 2.124 |
|  | M-Health can be piloted without serious negative impact to patients and hospital operations(2) | -1.869 | 1.228 | 2.316 | 1 | .128 | .154 | .014 | 1.712 |
|  | M-Health can be piloted without serious negative impact to patients and hospital operations(3) | -.978 | .953 | 1.055 | 1 | .304 | .376 | .058 | 2.432 |
|  | It is more strategic to adopt M-Health when the technology is leased to the hospital |  |  | 2.032 | 3 | .566 |  |  |  |
|  | It is more strategic to adopt M-Health when the technology is leased to the hospital(1) | 2.042 | 1.478 | 1.909 | 1 | .167 | 7.702 | .426 | 139.429 |
|  | It is more strategic to adopt M-Health when the technology is leased to the hospital(2) | 1.008 | 1.454 | .481 | 1 | .488 | 2.741 | .159 | 47.386 |
|  | It is more strategic to adopt M-Health when the technology is leased to the hospital(3) | .979 | 1.291 | .575 | 1 | .448 | 2.661 | .212 | 33.407 |
|  | It is more strategic to adopt M-Health when the hospital fully owned the technology |  |  | 7.666 | 3 | .053 |  |  |  |
|  | It is more strategic to adopt M-Health when the hospital fully owned the technology(1) | 7.374 | 3.632 | 4.123 | 1 | .042 | 1594.754 | 1.292 | 1968675.936 |
|  | It is more strategic to adopt M-Health when the hospital fully owned the technology(2) | -2.431 | 1.364 | 3.176 | 1 | .075 | .088 | .006 | 1.274 |
|  | It is more strategic to adopt M-Health when the hospital fully owned the technology(3) | -2.217 | 1.014 | 4.779 | 1 | .029 | .109 | .015 | .795 |
|  | Constant | -2.827 | 2.031 | 1.937 | 1 | .164 | .059 |  |  |
| a. Variable(s) entered on step 1: Use of M-health is superior and more advantageous than current manual practices., M-Health improves efficiency of hospital’s operations , M-health will reduce the cost of health care to patients, M-Health is compatible with current hospital’s health information system and consistent with its values and needs., M-Health can be trusted in terms of its security and confidentiality of patient information, M-Health will make many key staff redundant, M-Health is difficult to understand, use and integrate in the hospital systems and operations, Adoption of M-Health innovations requires exorbitant cost in infrastructure and human resources development, M-Health needs to be piloted first in order to demonstrate that it is better than using current manual systems, M-Health can be piloted without serious negative impact to patients and hospital operations, It is more strategic to adopt M-Health when the technology is leased to the hospital, It is more strategic to adopt M-Health when the hospital fully owned the technology. | | | | | | | | | |

1. *Emergency toll-free telephone services and technological determinants*

| **Case Processing Summary** | | | |
| --- | --- | --- | --- |
| Unweighted Cases^a^ | | N | Percent |
| Selected Cases | Included in Analysis | 156 | 73.9 |
|  | Missing Cases | 55 | 26.1 |
|  | Total | 211 | 100.0 |
| Unselected Cases | | 0 | .0 |
| Total | | 211 | 100.0 |
| a. If weight is in effect, see classification table for the total number of cases. | | | |

| **Dependent Variable Encoding** | |
| --- | --- |
| Original Value | Internal Value |
| Not adopted | 0 |
| Adopted | 1 |

| **Categorical Variables Codings** | | | | | |
| --- | --- | --- | --- | --- | --- |
|  | | Frequency | Parameter coding | | |
|  |  |  | (1) | (2) | (3) |
| It is more strategic to adopt M-Health when the hospital fully owned the technology | Strongly disgree | 14 | 1.000 | .000 | .000 |
|  | Disagree | 22 | .000 | 1.000 | .000 |
|  | Agree | 56 | .000 | .000 | 1.000 |
|  | Strongly Agree | 64 | .000 | .000 | .000 |
| M-Health improves efficiency of hospital’s operations | Strongly disgree | 6 | 1.000 | .000 | .000 |
|  | Disagree | 8 | .000 | 1.000 | .000 |
|  | Agree | 61 | .000 | .000 | 1.000 |
|  | Strongly Agree | 81 | .000 | .000 | .000 |
| M-health will reduce the cost of health care to patients | Strongly disgree | 8 | 1.000 | .000 | .000 |
|  | Disagree | 19 | .000 | 1.000 | .000 |
|  | Agree | 55 | .000 | .000 | 1.000 |
|  | Strongly Agree | 74 | .000 | .000 | .000 |
| M-Health is compatible with current hospital’s health information system and consistent with its values and needs. | Strongly disgree | 16 | 1.000 | .000 | .000 |
|  | Disagree | 29 | .000 | 1.000 | .000 |
|  | Agree | 60 | .000 | .000 | 1.000 |
|  | Strongly Agree | 51 | .000 | .000 | .000 |
| M-Health can be trusted in terms of its security and confidentiality of patient information | Strongly disgree | 10 | 1.000 | .000 | .000 |
|  | Disagree | 27 | .000 | 1.000 | .000 |
|  | Agree | 79 | .000 | .000 | 1.000 |
|  | Strongly Agree | 40 | .000 | .000 | .000 |
| M-Health will make many key staff redundant | Strongly disgree | 41 | 1.000 | .000 | .000 |
|  | Disagree | 62 | .000 | 1.000 | .000 |
|  | Agree | 36 | .000 | .000 | 1.000 |
|  | Strongly Agree | 17 | .000 | .000 | .000 |
| M-Health is difficult to understand, use and integrate in the hospital systems and operations | Strongly disgree | 48 | 1.000 | .000 | .000 |
|  | Disagree | 73 | .000 | 1.000 | .000 |
|  | Agree | 28 | .000 | .000 | 1.000 |
|  | Strongly Agree | 7 | .000 | .000 | .000 |
| It is more strategic to adopt M-Health when the technology is leased to the hospital | Strongly disgree | 34 | 1.000 | .000 | .000 |
|  | Disagree | 60 | .000 | 1.000 | .000 |
|  | Agree | 38 | .000 | .000 | 1.000 |
|  | Strongly Agree | 24 | .000 | .000 | .000 |
| M-Health can be piloted without serious negative impact to patients and hospital operations | Strongly disgree | 6 | 1.000 | .000 | .000 |
|  | Disagree | 24 | .000 | 1.000 | .000 |
|  | Agree | 81 | .000 | .000 | 1.000 |
|  | Strongly Agree | 45 | .000 | .000 | .000 |
| M-Health needs to be piloted first in order to demonstrate that it is better than using current manual systems | Strongly disgree | 16 | 1.000 | .000 | .000 |
|  | Disagree | 19 | .000 | 1.000 | .000 |
|  | Agree | 54 | .000 | .000 | 1.000 |
|  | Strongly Agree | 67 | .000 | .000 | .000 |
| Adoption of M-Health innovations requires exorbitant cost in infrastructure and human resources development | Strongly disgree | 23 | 1.000 | .000 | .000 |
|  | Disagree | 51 | .000 | 1.000 | .000 |
|  | Agree | 57 | .000 | .000 | 1.000 |
|  | Strongly Agree | 25 | .000 | .000 | .000 |
| Use of M-health is superior and more advantageous than current manual practices. | Strongly disgree | 10 | 1.000 | .000 | .000 |
|  | Disagree | 13 | .000 | 1.000 | .000 |
|  | Agree | 60 | .000 | .000 | 1.000 |
|  | Strongly Agree | 73 | .000 | .000 | .000 |

**Block 0: Beginning Block**

| **Classification Table^a,b^** | | | | | |
| --- | --- | --- | --- | --- | --- |
|  | Observed | | Predicted | | |
|  |  |  | Emergency toll-free telephone services | | Percentage Correct |
|  |  |  | Not adopted | Adopted |  |
| Step 0 | Emergency toll-free telephone services | Not adopted | 110 | 0 | 100.0 |
|  |  | Adopted | 46 | 0 | .0 |
|  | Overall Percentage | |  |  | 70.5 |
| a. Constant is included in the model. | | | | | |
| b. The cut value is .500 | | | | | |

| **Variables in the Equation** | | | | | | | |
| --- | --- | --- | --- | --- | --- | --- | --- |
|  | | B | S.E. | Wald | df | Sig. | Exp(B) |
| Step 0 | Constant | -.872 | .176 | 24.655 | 1 | .000 | .418 |

| **Variables not in the Equation** | | | | | |
| --- | --- | --- | --- | --- | --- |
|  | | | Score | df | Sig. |
| Step 0 | Variables | Use of M-health is superior and more advantageous than current manual practices. | 3.145 | 3 | .370 |
|  |  | Use of M-health is superior and more advantageous than current manual practices.(1) | .001 | 1 | .971 |
|  |  | Use of M-health is superior and more advantageous than current manual practices.(2) | 1.357 | 1 | .244 |
|  |  | Use of M-health is superior and more advantageous than current manual practices.(3) | .944 | 1 | .331 |
|  |  | M-Health improves efficiency of hospital’s operations | .697 | 3 | .874 |
|  |  | M-Health improves efficiency of hospital’s operations (1) | .044 | 1 | .833 |
|  |  | M-Health improves efficiency of hospital’s operations (2) | .082 | 1 | .775 |
|  |  | M-Health improves efficiency of hospital’s operations (3) | .511 | 1 | .475 |
|  |  | M-health will reduce the cost of health care to patients | 7.470 | 3 | .058 |
|  |  | M-health will reduce the cost of health care to patients(1) | 4.420 | 1 | .036 |
|  |  | M-health will reduce the cost of health care to patients(2) | 1.952 | 1 | .162 |
|  |  | M-health will reduce the cost of health care to patients(3) | 1.399 | 1 | .237 |
|  |  | M-Health is compatible with current hospital’s health information system and consistent with its values and needs. | 4.980 | 3 | .173 |
|  |  | M-Health is compatible with current hospital’s health information system and consistent with its values and needs.(1) | 2.474 | 1 | .116 |
|  |  | M-Health is compatible with current hospital’s health information system and consistent with its values and needs.(2) | .490 | 1 | .484 |
|  |  | M-Health is compatible with current hospital’s health information system and consistent with its values and needs.(3) | .062 | 1 | .803 |
|  |  | M-Health can be trusted in terms of its security and confidentiality of patient information | 4.697 | 3 | .195 |
|  |  | M-Health can be trusted in terms of its security and confidentiality of patient information(1) | .463 | 1 | .496 |
|  |  | M-Health can be trusted in terms of its security and confidentiality of patient information(2) | .829 | 1 | .363 |
|  |  | M-Health can be trusted in terms of its security and confidentiality of patient information(3) | .650 | 1 | .420 |
|  |  | M-Health will make many key staff redundant | 3.431 | 3 | .330 |
|  |  | M-Health will make many key staff redundant(1) | .132 | 1 | .717 |
|  |  | M-Health will make many key staff redundant(2) | .010 | 1 | .919 |
|  |  | M-Health will make many key staff redundant(3) | .988 | 1 | .320 |
|  |  | M-Health is difficult to understand, use and integrate in the hospital systems and operations | 3.944 | 3 | .268 |
|  |  | M-Health is difficult to understand, use and integrate in the hospital systems and operations(1) | .493 | 1 | .482 |
|  |  | M-Health is difficult to understand, use and integrate in the hospital systems and operations(2) | .288 | 1 | .591 |
|  |  | M-Health is difficult to understand, use and integrate in the hospital systems and operations(3) | 1.066 | 1 | .302 |
|  |  | Adoption of M-Health innovations requires exorbitant cost in infrastructure and human resources development | 2.131 | 3 | .546 |
|  |  | Adoption of M-Health innovations requires exorbitant cost in infrastructure and human resources development(1) | .364 | 1 | .546 |
|  |  | Adoption of M-Health innovations requires exorbitant cost in infrastructure and human resources development(2) | .130 | 1 | .719 |
|  |  | Adoption of M-Health innovations requires exorbitant cost in infrastructure and human resources development(3) | 1.928 | 1 | .165 |
|  |  | M-Health needs to be piloted first in order to demonstrate that it is better than using current manual systems | 7.016 | 3 | .071 |
|  |  | M-Health needs to be piloted first in order to demonstrate that it is better than using current manual systems(1) | .989 | 1 | .320 |
|  |  | M-Health needs to be piloted first in order to demonstrate that it is better than using current manual systems(2) | 1.657 | 1 | .198 |
|  |  | M-Health needs to be piloted first in order to demonstrate that it is better than using current manual systems(3) | 3.511 | 1 | .061 |
|  |  | M-Health can be piloted without serious negative impact to patients and hospital operations | 2.405 | 3 | .493 |
|  |  | M-Health can be piloted without serious negative impact to patients and hospital operations(1) | .493 | 1 | .482 |
|  |  | M-Health can be piloted without serious negative impact to patients and hospital operations(2) | 2.024 | 1 | .155 |
|  |  | M-Health can be piloted without serious negative impact to patients and hospital operations(3) | .439 | 1 | .508 |
|  |  | It is more strategic to adopt M-Health when the technology is leased to the hospital | 7.682 | 3 | .053 |
|  |  | It is more strategic to adopt M-Health when the technology is leased to the hospital(1) | 1.656 | 1 | .198 |
|  |  | It is more strategic to adopt M-Health when the technology is leased to the hospital(2) | 2.868 | 1 | .090 |
|  |  | It is more strategic to adopt M-Health when the technology is leased to the hospital(3) | 3.847 | 1 | .050 |
|  |  | It is more strategic to adopt M-Health when the hospital fully owned the technology | 4.450 | 3 | .217 |
|  |  | It is more strategic to adopt M-Health when the hospital fully owned the technology(1) | 1.322 | 1 | .250 |
|  |  | It is more strategic to adopt M-Health when the hospital fully owned the technology(2) | 3.095 | 1 | .079 |
|  |  | It is more strategic to adopt M-Health when the hospital fully owned the technology(3) | .829 | 1 | .363 |
|  | Overall Statistics | | 52.263 | 36 | .039 |

**Block 1: Method = Enter**

| **Omnibus Tests of Model Coefficients** | | | | |
| --- | --- | --- | --- | --- |
|  | | Chi-square | df | Sig. |
| Step 1 | Step | 60.674 | 36 | .006 |
|  | Block | 60.674 | 36 | .006 |
|  | Model | 60.674 | 36 | .006 |

| **Model Summary** | | | |
| --- | --- | --- | --- |
| Step | -2 Log likelihood | Cox & Snell R Square | Nagelkerke R Square |
| 1 | 128.541^a^ | .322 | .459 |
| a. Estimation terminated at iteration number 6 because parameter estimates changed by less than .001. | | | |

| **Hosmer and Lemeshow Test** | | | |
| --- | --- | --- | --- |
| Step | Chi-square | df | Sig. |
| 1 | 4.583 | 8 | .801 |

| **Contingency Table for Hosmer and Lemeshow Test** | | | | | | |
| --- | --- | --- | --- | --- | --- | --- |
|  | | Emergency toll-free telephone services = Not adopted | | Emergency toll-free telephone services = Adopted | | Total |
|  |  | Observed | Expected | Observed | Expected |  |
| Step 1 | 1 | 16 | 15.829 | 0 | .171 | 16 |
|  | 2 | 14 | 15.324 | 2 | .676 | 16 |
|  | 3 | 15 | 14.873 | 1 | 1.127 | 16 |
|  | 4 | 15 | 14.137 | 1 | 1.863 | 16 |
|  | 5 | 14 | 13.122 | 2 | 2.878 | 16 |
|  | 6 | 13 | 12.060 | 3 | 3.940 | 16 |
|  | 7 | 9 | 10.479 | 7 | 5.521 | 16 |
|  | 8 | 8 | 8.067 | 8 | 7.933 | 16 |
|  | 9 | 5 | 5.064 | 11 | 10.936 | 16 |
|  | 10 | 1 | 1.046 | 11 | 10.954 | 12 |

| **Classification Table^a^** | | | | | |
| --- | --- | --- | --- | --- | --- |
|  | Observed | | Predicted | | |
|  |  |  | Emergency toll-free telephone services | | Percentage Correct |
|  |  |  | Not adopted | Adopted |  |
| Step 1 | Emergency toll-free telephone services | Not adopted | 100 | 10 | 90.9 |
|  |  | Adopted | 19 | 27 | 58.7 |
|  | Overall Percentage | |  |  | 81.4 |
| a. The cut value is .500 | | | | | |

| **Variables in the Equation** | | | | | | | | | |
| --- | --- | --- | --- | --- | --- | --- | --- | --- | --- |
|  | | B | S.E. | Wald | df | Sig. | Exp(B) | 95% C.I.for EXP(B) | |
|  |  |  |  |  |  |  |  | Lower | Upper |
| Step 1^a^ | Use of M-health is superior and more advantageous than current manual practices. |  |  | 3.692 | 3 | .297 |  |  |  |
|  | Use of M-health is superior and more advantageous than current manual practices.(1) | .090 | 1.505 | .004 | 1 | .952 | 1.094 | .057 | 20.893 |
|  | Use of M-health is superior and more advantageous than current manual practices.(2) | -.793 | 1.298 | .373 | 1 | .541 | .452 | .036 | 5.760 |
|  | Use of M-health is superior and more advantageous than current manual practices.(3) | -1.308 | .696 | 3.536 | 1 | .060 | .270 | .069 | 1.057 |
|  | M-Health improves efficiency of hospital’s operations |  |  | 2.837 | 3 | .417 |  |  |  |
|  | M-Health improves efficiency of hospital’s operations (1) | -1.267 | 2.999 | .178 | 1 | .673 | .282 | .001 | 100.575 |
|  | M-Health improves efficiency of hospital’s operations (2) | -1.349 | 1.690 | .637 | 1 | .425 | .259 | .009 | 7.123 |
|  | M-Health improves efficiency of hospital’s operations (3) | .889 | .814 | 1.195 | 1 | .274 | 2.433 | .494 | 11.986 |
|  | M-health will reduce the cost of health care to patients |  |  | 3.602 | 3 | .308 |  |  |  |
|  | M-health will reduce the cost of health care to patients(1) | 1.673 | 1.565 | 1.143 | 1 | .285 | 5.327 | .248 | 114.410 |
|  | M-health will reduce the cost of health care to patients(2) | -1.421 | 1.063 | 1.790 | 1 | .181 | .241 | .030 | 1.937 |
|  | M-health will reduce the cost of health care to patients(3) | -.639 | .724 | .778 | 1 | .378 | .528 | .128 | 2.184 |
|  | M-Health is compatible with current hospital’s health information system and consistent with its values and needs. |  |  | 2.918 | 3 | .405 |  |  |  |
|  | M-Health is compatible with current hospital’s health information system and consistent with its values and needs.(1) | -2.272 | 1.347 | 2.844 | 1 | .092 | .103 | .007 | 1.445 |
|  | M-Health is compatible with current hospital’s health information system and consistent with its values and needs.(2) | -.528 | .884 | .357 | 1 | .550 | .590 | .104 | 3.334 |
|  | M-Health is compatible with current hospital’s health information system and consistent with its values and needs.(3) | -.456 | .657 | .480 | 1 | .488 | .634 | .175 | 2.300 |
|  | M-Health can be trusted in terms of its security and confidentiality of patient information |  |  | 3.000 | 3 | .392 |  |  |  |
|  | M-Health can be trusted in terms of its security and confidentiality of patient information(1) | .417 | 1.484 | .079 | 1 | .779 | 1.518 | .083 | 27.814 |
|  | M-Health can be trusted in terms of its security and confidentiality of patient information(2) | -.352 | .923 | .145 | 1 | .703 | .703 | .115 | 4.296 |
|  | M-Health can be trusted in terms of its security and confidentiality of patient information(3) | -1.046 | .665 | 2.475 | 1 | .116 | .351 | .095 | 1.293 |
|  | M-Health will make many key staff redundant |  |  | 4.486 | 3 | .214 |  |  |  |
|  | M-Health will make many key staff redundant(1) | 2.385 | 1.273 | 3.511 | 1 | .061 | 10.857 | .896 | 131.529 |
|  | M-Health will make many key staff redundant(2) | 2.691 | 1.307 | 4.239 | 1 | .040 | 14.750 | 1.138 | 191.198 |
|  | M-Health will make many key staff redundant(3) | 2.519 | 1.249 | 4.064 | 1 | .044 | 12.415 | 1.073 | 143.699 |
|  | M-Health is difficult to understand, use and integrate in the hospital systems and operations |  |  | 1.363 | 3 | .714 |  |  |  |
|  | M-Health is difficult to understand, use and integrate in the hospital systems and operations(1) | -1.500 | 1.367 | 1.205 | 1 | .272 | .223 | .015 | 3.251 |
|  | M-Health is difficult to understand, use and integrate in the hospital systems and operations(2) | -1.478 | 1.341 | 1.215 | 1 | .270 | .228 | .016 | 3.159 |
|  | M-Health is difficult to understand, use and integrate in the hospital systems and operations(3) | -1.188 | 1.453 | .669 | 1 | .413 | .305 | .018 | 5.253 |
|  | Adoption of M-Health innovations requires exorbitant cost in infrastructure and human resources development |  |  | 7.261 | 3 | .064 |  |  |  |
|  | Adoption of M-Health innovations requires exorbitant cost in infrastructure and human resources development(1) | -1.681 | .986 | 2.909 | 1 | .088 | .186 | .027 | 1.285 |
|  | Adoption of M-Health innovations requires exorbitant cost in infrastructure and human resources development(2) | .462 | .828 | .311 | 1 | .577 | 1.587 | .313 | 8.035 |
|  | Adoption of M-Health innovations requires exorbitant cost in infrastructure and human resources development(3) | -1.245 | .812 | 2.350 | 1 | .125 | .288 | .059 | 1.415 |
|  | M-Health needs to be piloted first in order to demonstrate that it is better than using current manual systems |  |  | 5.022 | 3 | .170 |  |  |  |
|  | M-Health needs to be piloted first in order to demonstrate that it is better than using current manual systems(1) | .474 | 1.061 | .199 | 1 | .655 | 1.606 | .201 | 12.841 |
|  | M-Health needs to be piloted first in order to demonstrate that it is better than using current manual systems(2) | 1.696 | .912 | 3.454 | 1 | .063 | 5.451 | .912 | 32.599 |
|  | M-Health needs to be piloted first in order to demonstrate that it is better than using current manual systems(3) | 1.347 | .726 | 3.442 | 1 | .064 | 3.846 | .927 | 15.964 |
|  | M-Health can be piloted without serious negative impact to patients and hospital operations |  |  | 6.945 | 3 | .074 |  |  |  |
|  | M-Health can be piloted without serious negative impact to patients and hospital operations(1) | -1.861 | 2.112 | .776 | 1 | .378 | .156 | .002 | 9.766 |
|  | M-Health can be piloted without serious negative impact to patients and hospital operations(2) | 2.253 | .963 | 5.481 | 1 | .019 | 9.521 | 1.443 | 62.807 |
|  | M-Health can be piloted without serious negative impact to patients and hospital operations(3) | .144 | .664 | .047 | 1 | .829 | 1.154 | .314 | 4.238 |
|  | It is more strategic to adopt M-Health when the technology is leased to the hospital |  |  | 10.470 | 3 | .015 |  |  |  |
|  | It is more strategic to adopt M-Health when the technology is leased to the hospital(1) | -.671 | .861 | .608 | 1 | .435 | .511 | .095 | 2.762 |
|  | It is more strategic to adopt M-Health when the technology is leased to the hospital(2) | -2.451 | .932 | 6.923 | 1 | .009 | .086 | .014 | .535 |
|  | It is more strategic to adopt M-Health when the technology is leased to the hospital(3) | .292 | .888 | .108 | 1 | .742 | 1.339 | .235 | 7.626 |
|  | It is more strategic to adopt M-Health when the hospital fully owned the technology |  |  | 9.955 | 3 | .019 |  |  |  |
|  | It is more strategic to adopt M-Health when the hospital fully owned the technology(1) | .210 | 1.092 | .037 | 1 | .848 | 1.233 | .145 | 10.492 |
|  | It is more strategic to adopt M-Health when the hospital fully owned the technology(2) | -2.186 | 1.086 | 4.054 | 1 | .044 | .112 | .013 | .944 |
|  | It is more strategic to adopt M-Health when the hospital fully owned the technology(3) | 1.246 | .691 | 3.247 | 1 | .072 | 3.476 | .897 | 13.474 |
|  | Constant | -.186 | 1.663 | .013 | 1 | .911 | .830 |  |  |
| a. Variable(s) entered on step 1: Use of M-health is superior and more advantageous than current manual practices., M-Health improves efficiency of hospital’s operations , M-health will reduce the cost of health care to patients, M-Health is compatible with current hospital’s health information system and consistent with its values and needs., M-Health can be trusted in terms of its security and confidentiality of patient information, M-Health will make many key staff redundant, M-Health is difficult to understand, use and integrate in the hospital systems and operations, Adoption of M-Health innovations requires exorbitant cost in infrastructure and human resources development, M-Health needs to be piloted first in order to demonstrate that it is better than using current manual systems, M-Health can be piloted without serious negative impact to patients and hospital operations, It is more strategic to adopt M-Health when the technology is leased to the hospital, It is more strategic to adopt M-Health when the hospital fully owned the technology. | | | | | | | | | |

1. *Treatment compliance and technological determinants*

| **Case Processing Summary** | | | |
| --- | --- | --- | --- |
| Unweighted Cases^a^ | | N | Percent |
| Selected Cases | Included in Analysis | 163 | 77.3 |
|  | Missing Cases | 48 | 22.7 |
|  | Total | 211 | 100.0 |
| Unselected Cases | | 0 | .0 |
| Total | | 211 | 100.0 |
| a. If weight is in effect, see classification table for the total number of cases. | | | |

| **Dependent Variable Encoding** | |
| --- | --- |
| Original Value | Internal Value |
| Not adopted | 0 |
| Adopted | 1 |

| **Categorical Variables Codings** | | | | | |
| --- | --- | --- | --- | --- | --- |
|  | | Frequency | Parameter coding | | |
|  |  |  | (1) | (2) | (3) |
| It is more strategic to adopt M-Health when the hospital fully owned the technology | Strongly disgree | 15 | 1.000 | .000 | .000 |
|  | Disagree | 22 | .000 | 1.000 | .000 |
|  | Agree | 60 | .000 | .000 | 1.000 |
|  | Strongly Agree | 66 | .000 | .000 | .000 |
| M-Health improves efficiency of hospital’s operations | Strongly disgree | 6 | 1.000 | .000 | .000 |
|  | Disagree | 8 | .000 | 1.000 | .000 |
|  | Agree | 61 | .000 | .000 | 1.000 |
|  | Strongly Agree | 88 | .000 | .000 | .000 |
| M-health will reduce the cost of health care to patients | Strongly disgree | 8 | 1.000 | .000 | .000 |
|  | Disagree | 21 | .000 | 1.000 | .000 |
|  | Agree | 59 | .000 | .000 | 1.000 |
|  | Strongly Agree | 75 | .000 | .000 | .000 |
| M-Health is compatible with current hospital’s health information system and consistent with its values and needs. | Strongly disgree | 15 | 1.000 | .000 | .000 |
|  | Disagree | 28 | .000 | 1.000 | .000 |
|  | Agree | 66 | .000 | .000 | 1.000 |
|  | Strongly Agree | 54 | .000 | .000 | .000 |
| M-Health can be trusted in terms of its security and confidentiality of patient information | Strongly disgree | 9 | 1.000 | .000 | .000 |
|  | Disagree | 29 | .000 | 1.000 | .000 |
|  | Agree | 83 | .000 | .000 | 1.000 |
|  | Strongly Agree | 42 | .000 | .000 | .000 |
| M-Health will make many key staff redundant | Strongly disgree | 44 | 1.000 | .000 | .000 |
|  | Disagree | 63 | .000 | 1.000 | .000 |
|  | Agree | 39 | .000 | .000 | 1.000 |
|  | Strongly Agree | 17 | .000 | .000 | .000 |
| M-Health is difficult to understand, use and integrate in the hospital systems and operations | Strongly disgree | 53 | 1.000 | .000 | .000 |
|  | Disagree | 75 | .000 | 1.000 | .000 |
|  | Agree | 28 | .000 | .000 | 1.000 |
|  | Strongly Agree | 7 | .000 | .000 | .000 |
| It is more strategic to adopt M-Health when the technology is leased to the hospital | Strongly disgree | 33 | 1.000 | .000 | .000 |
|  | Disagree | 61 | .000 | 1.000 | .000 |
|  | Agree | 43 | .000 | .000 | 1.000 |
|  | Strongly Agree | 26 | .000 | .000 | .000 |
| M-Health can be piloted without serious negative impact to patients and hospital operations | Strongly disgree | 4 | 1.000 | .000 | .000 |
|  | Disagree | 24 | .000 | 1.000 | .000 |
|  | Agree | 84 | .000 | .000 | 1.000 |
|  | Strongly Agree | 51 | .000 | .000 | .000 |
| M-Health needs to be piloted first in order to demonstrate that it is better than using current manual systems | Strongly disgree | 17 | 1.000 | .000 | .000 |
|  | Disagree | 20 | .000 | 1.000 | .000 |
|  | Agree | 56 | .000 | .000 | 1.000 |
|  | Strongly Agree | 70 | .000 | .000 | .000 |
| Adoption of M-Health innovations requires exorbitant cost in infrastructure and human resources development | Strongly disgree | 25 | 1.000 | .000 | .000 |
|  | Disagree | 54 | .000 | 1.000 | .000 |
|  | Agree | 59 | .000 | .000 | 1.000 |
|  | Strongly Agree | 25 | .000 | .000 | .000 |
| Use of M-health is superior and more advantageous than current manual practices. | Strongly disgree | 10 | 1.000 | .000 | .000 |
|  | Disagree | 12 | .000 | 1.000 | .000 |
|  | Agree | 65 | .000 | .000 | 1.000 |
|  | Strongly Agree | 76 | .000 | .000 | .000 |

**Block 0: Beginning Block**

| **Classification Table^a,b^** | | | | | |
| --- | --- | --- | --- | --- | --- |
|  | Observed | | Predicted | | |
|  |  |  | Treatment compliance | | Percentage Correct |
|  |  |  | Not adopted | Adopted |  |
| Step 0 | Treatment compliance | Not adopted | 0 | 49 | .0 |
|  |  | Adopted | 0 | 114 | 100.0 |
|  | Overall Percentage | |  |  | 69.9 |
| a. Constant is included in the model. | | | | | |
| b. The cut value is .500 | | | | | |

| **Variables in the Equation** | | | | | | | |
| --- | --- | --- | --- | --- | --- | --- | --- |
|  | | B | S.E. | Wald | df | Sig. | Exp(B) |
| Step 0 | Constant | .844 | .171 | 24.434 | 1 | .000 | 2.327 |

| **Variables not in the Equation** | | | | | |
| --- | --- | --- | --- | --- | --- |
|  | | | Score | df | Sig. |
| Step 0 | Variables | Use of M-health is superior and more advantageous than current manual practices. | 4.119 | 3 | .249 |
|  |  | Use of M-health is superior and more advantageous than current manual practices.(1) | .513 | 1 | .474 |
|  |  | Use of M-health is superior and more advantageous than current manual practices.(2) | 2.449 | 1 | .118 |
|  |  | Use of M-health is superior and more advantageous than current manual practices.(3) | .737 | 1 | .391 |
|  |  | M-Health improves efficiency of hospital’s operations | 2.895 | 3 | .408 |
|  |  | M-Health improves efficiency of hospital’s operations (1) | .032 | 1 | .859 |
|  |  | M-Health improves efficiency of hospital’s operations (2) | .103 | 1 | .749 |
|  |  | M-Health improves efficiency of hospital’s operations (3) | 2.709 | 1 | .100 |
|  |  | M-health will reduce the cost of health care to patients | 2.912 | 3 | .405 |
|  |  | M-health will reduce the cost of health care to patients(1) | .103 | 1 | .749 |
|  |  | M-health will reduce the cost of health care to patients(2) | .740 | 1 | .390 |
|  |  | M-health will reduce the cost of health care to patients(3) | 1.346 | 1 | .246 |
|  |  | M-Health is compatible with current hospital’s health information system and consistent with its values and needs. | 4.809 | 3 | .186 |
|  |  | M-Health is compatible with current hospital’s health information system and consistent with its values and needs.(1) | .084 | 1 | .772 |
|  |  | M-Health is compatible with current hospital’s health information system and consistent with its values and needs.(2) | 4.308 | 1 | .038 |
|  |  | M-Health is compatible with current hospital’s health information system and consistent with its values and needs.(3) | .410 | 1 | .522 |
|  |  | M-Health can be trusted in terms of its security and confidentiality of patient information | 7.331 | 3 | .062 |
|  |  | M-Health can be trusted in terms of its security and confidentiality of patient information(1) | .937 | 1 | .333 |
|  |  | M-Health can be trusted in terms of its security and confidentiality of patient information(2) | .016 | 1 | .900 |
|  |  | M-Health can be trusted in terms of its security and confidentiality of patient information(3) | 2.977 | 1 | .084 |
|  |  | M-Health will make many key staff redundant | .749 | 3 | .862 |
|  |  | M-Health will make many key staff redundant(1) | .465 | 1 | .495 |
|  |  | M-Health will make many key staff redundant(2) | .000 | 1 | .983 |
|  |  | M-Health will make many key staff redundant(3) | .084 | 1 | .772 |
|  |  | M-Health is difficult to understand, use and integrate in the hospital systems and operations | 4.696 | 3 | .195 |
|  |  | M-Health is difficult to understand, use and integrate in the hospital systems and operations(1) | 3.235 | 1 | .072 |
|  |  | M-Health is difficult to understand, use and integrate in the hospital systems and operations(2) | 2.330 | 1 | .127 |
|  |  | M-Health is difficult to understand, use and integrate in the hospital systems and operations(3) | .514 | 1 | .473 |
|  |  | Adoption of M-Health innovations requires exorbitant cost in infrastructure and human resources development | 4.073 | 3 | .254 |
|  |  | Adoption of M-Health innovations requires exorbitant cost in infrastructure and human resources development(1) | 1.387 | 1 | .239 |
|  |  | Adoption of M-Health innovations requires exorbitant cost in infrastructure and human resources development(2) | .200 | 1 | .654 |
|  |  | Adoption of M-Health innovations requires exorbitant cost in infrastructure and human resources development(3) | .648 | 1 | .421 |
|  |  | M-Health needs to be piloted first in order to demonstrate that it is better than using current manual systems | .569 | 3 | .903 |
|  |  | M-Health needs to be piloted first in order to demonstrate that it is better than using current manual systems(1) | .247 | 1 | .619 |
|  |  | M-Health needs to be piloted first in order to demonstrate that it is better than using current manual systems(2) | .000 | 1 | .995 |
|  |  | M-Health needs to be piloted first in order to demonstrate that it is better than using current manual systems(3) | .435 | 1 | .509 |
|  |  | M-Health can be piloted without serious negative impact to patients and hospital operations | 6.866 | 3 | .076 |
|  |  | M-Health can be piloted without serious negative impact to patients and hospital operations(1) | .775 | 1 | .379 |
|  |  | M-Health can be piloted without serious negative impact to patients and hospital operations(2) | 5.322 | 1 | .021 |
|  |  | M-Health can be piloted without serious negative impact to patients and hospital operations(3) | 3.222 | 1 | .073 |
|  |  | It is more strategic to adopt M-Health when the technology is leased to the hospital | 1.921 | 3 | .589 |
|  |  | It is more strategic to adopt M-Health when the technology is leased to the hospital(1) | .001 | 1 | .973 |
|  |  | It is more strategic to adopt M-Health when the technology is leased to the hospital(2) | 1.388 | 1 | .239 |
|  |  | It is more strategic to adopt M-Health when the technology is leased to the hospital(3) | 1.419 | 1 | .233 |
|  |  | It is more strategic to adopt M-Health when the hospital fully owned the technology | 4.815 | 3 | .186 |
|  |  | It is more strategic to adopt M-Health when the hospital fully owned the technology(1) | .091 | 1 | .763 |
|  |  | It is more strategic to adopt M-Health when the hospital fully owned the technology(2) | 4.809 | 1 | .028 |
|  |  | It is more strategic to adopt M-Health when the hospital fully owned the technology(3) | .520 | 1 | .471 |
|  | Overall Statistics | | 39.275 | 36 | .325 |

**Block 1: Method = Enter**

| **Omnibus Tests of Model Coefficients** | | | | |
| --- | --- | --- | --- | --- |
|  | | Chi-square | df | Sig. |
| Step 1 | Step | 43.724 | 36 | .176 |
|  | Block | 43.724 | 36 | .176 |
|  | Model | 43.724 | 36 | .176 |

| **Model Summary** | | | |
| --- | --- | --- | --- |
| Step | -2 Log likelihood | Cox & Snell R Square | Nagelkerke R Square |
| 1 | 155.586^a^ | .235 | .333 |
| a. Estimation terminated at iteration number 5 because parameter estimates changed by less than .001. | | | |

| **Hosmer and Lemeshow Test** | | | |
| --- | --- | --- | --- |
| Step | Chi-square | df | Sig. |
| 1 | 4.045 | 8 | .853 |

| **Contingency Table for Hosmer and Lemeshow Test** | | | | | | |
| --- | --- | --- | --- | --- | --- | --- |
|  | | Treatment compliance = Not adopted | | Treatment compliance = Adopted | | Total |
|  |  | Observed | Expected | Observed | Expected |  |
| Step 1 | 1 | 14 | 12.729 | 2 | 3.271 | 16 |
|  | 2 | 8 | 9.279 | 8 | 6.721 | 16 |
|  | 3 | 6 | 7.214 | 10 | 8.786 | 16 |
|  | 4 | 4 | 5.491 | 12 | 10.509 | 16 |
|  | 5 | 5 | 4.404 | 11 | 11.596 | 16 |
|  | 6 | 4 | 3.382 | 12 | 12.618 | 16 |
|  | 7 | 3 | 2.754 | 14 | 14.246 | 17 |
|  | 8 | 3 | 1.955 | 13 | 14.045 | 16 |
|  | 9 | 2 | 1.231 | 14 | 14.769 | 16 |
|  | 10 | 0 | .562 | 18 | 17.438 | 18 |

| **Classification Table^a^** | | | | | |
| --- | --- | --- | --- | --- | --- |
|  | Observed | | Predicted | | |
|  |  |  | Treatment compliance | | Percentage Correct |
|  |  |  | Not adopted | Adopted |  |
| Step 1 | Treatment compliance | Not adopted | 23 | 26 | 46.9 |
|  |  | Adopted | 10 | 104 | 91.2 |
|  | Overall Percentage | |  |  | 77.9 |
| a. The cut value is .500 | | | | | |

| **Variables in the Equation** | | | | | | | | | |
| --- | --- | --- | --- | --- | --- | --- | --- | --- | --- |
|  | | B | S.E. | Wald | df | Sig. | Exp(B) | 95% C.I.for EXP(B) | |
|  |  |  |  |  |  |  |  | Lower | Upper |
| Step 1^a^ | Use of M-health is superior and more advantageous than current manual practices. |  |  | .913 | 3 | .822 |  |  |  |
|  | Use of M-health is superior and more advantageous than current manual practices.(1) | .469 | 1.640 | .082 | 1 | .775 | 1.598 | .064 | 39.764 |
|  | Use of M-health is superior and more advantageous than current manual practices.(2) | -.862 | 1.092 | .624 | 1 | .430 | .422 | .050 | 3.588 |
|  | Use of M-health is superior and more advantageous than current manual practices.(3) | -.376 | .581 | .418 | 1 | .518 | .687 | .220 | 2.144 |
|  | M-Health improves efficiency of hospital’s operations |  |  | .460 | 3 | .928 |  |  |  |
|  | M-Health improves efficiency of hospital’s operations (1) | -.313 | 2.086 | .023 | 1 | .881 | .731 | .012 | 43.636 |
|  | M-Health improves efficiency of hospital’s operations (2) | .597 | 1.280 | .217 | 1 | .641 | 1.816 | .148 | 22.306 |
|  | M-Health improves efficiency of hospital’s operations (3) | .330 | .669 | .243 | 1 | .622 | 1.391 | .375 | 5.167 |
|  | M-health will reduce the cost of health care to patients |  |  | 2.809 | 3 | .422 |  |  |  |
|  | M-health will reduce the cost of health care to patients(1) | .425 | 1.499 | .081 | 1 | .777 | 1.530 | .081 | 28.858 |
|  | M-health will reduce the cost of health care to patients(2) | -.705 | .840 | .704 | 1 | .402 | .494 | .095 | 2.565 |
|  | M-health will reduce the cost of health care to patients(3) | -.959 | .621 | 2.380 | 1 | .123 | .383 | .113 | 1.296 |
|  | M-Health is compatible with current hospital’s health information system and consistent with its values and needs. |  |  | 4.007 | 3 | .261 |  |  |  |
|  | M-Health is compatible with current hospital’s health information system and consistent with its values and needs.(1) | .418 | .963 | .189 | 1 | .664 | 1.519 | .230 | 10.023 |
|  | M-Health is compatible with current hospital’s health information system and consistent with its values and needs.(2) | -1.006 | .756 | 1.770 | 1 | .183 | .366 | .083 | 1.610 |
|  | M-Health is compatible with current hospital’s health information system and consistent with its values and needs.(3) | .166 | .621 | .071 | 1 | .789 | 1.181 | .350 | 3.987 |
|  | M-Health can be trusted in terms of its security and confidentiality of patient information |  |  | 5.901 | 3 | .117 |  |  |  |
|  | M-Health can be trusted in terms of its security and confidentiality of patient information(1) | -1.603 | 1.131 | 2.008 | 1 | .156 | .201 | .022 | 1.848 |
|  | M-Health can be trusted in terms of its security and confidentiality of patient information(2) | -.058 | .820 | .005 | 1 | .944 | .944 | .189 | 4.711 |
|  | M-Health can be trusted in terms of its security and confidentiality of patient information(3) | -1.121 | .661 | 2.880 | 1 | .090 | .326 | .089 | 1.190 |
|  | M-Health will make many key staff redundant |  |  | 3.488 | 3 | .322 |  |  |  |
|  | M-Health will make many key staff redundant(1) | .005 | .897 | .000 | 1 | .995 | 1.005 | .173 | 5.836 |
|  | M-Health will make many key staff redundant(2) | .519 | .942 | .304 | 1 | .581 | 1.681 | .265 | 10.652 |
|  | M-Health will make many key staff redundant(3) | 1.333 | 1.015 | 1.724 | 1 | .189 | 3.794 | .519 | 27.760 |
|  | M-Health is difficult to understand, use and integrate in the hospital systems and operations |  |  | 6.523 | 3 | .089 |  |  |  |
|  | M-Health is difficult to understand, use and integrate in the hospital systems and operations(1) | .584 | 1.367 | .183 | 1 | .669 | 1.793 | .123 | 26.118 |
|  | M-Health is difficult to understand, use and integrate in the hospital systems and operations(2) | -1.066 | 1.339 | .634 | 1 | .426 | .344 | .025 | 4.752 |
|  | M-Health is difficult to understand, use and integrate in the hospital systems and operations(3) | -.591 | 1.381 | .183 | 1 | .669 | .554 | .037 | 8.294 |
|  | Adoption of M-Health innovations requires exorbitant cost in infrastructure and human resources development |  |  | 5.976 | 3 | .113 |  |  |  |
|  | Adoption of M-Health innovations requires exorbitant cost in infrastructure and human resources development(1) | -2.182 | .942 | 5.360 | 1 | .021 | .113 | .018 | .716 |
|  | Adoption of M-Health innovations requires exorbitant cost in infrastructure and human resources development(2) | -.714 | .832 | .736 | 1 | .391 | .490 | .096 | 2.502 |
|  | Adoption of M-Health innovations requires exorbitant cost in infrastructure and human resources development(3) | -.770 | .809 | .904 | 1 | .342 | .463 | .095 | 2.263 |
|  | M-Health needs to be piloted first in order to demonstrate that it is better than using current manual systems |  |  | .420 | 3 | .936 |  |  |  |
|  | M-Health needs to be piloted first in order to demonstrate that it is better than using current manual systems(1) | .246 | .817 | .091 | 1 | .763 | 1.279 | .258 | 6.346 |
|  | M-Health needs to be piloted first in order to demonstrate that it is better than using current manual systems(2) | .499 | .808 | .380 | 1 | .537 | 1.646 | .338 | 8.029 |
|  | M-Health needs to be piloted first in order to demonstrate that it is better than using current manual systems(3) | .085 | .584 | .021 | 1 | .884 | 1.089 | .347 | 3.421 |
|  | M-Health can be piloted without serious negative impact to patients and hospital operations |  |  | 7.287 | 3 | .063 |  |  |  |
|  | M-Health can be piloted without serious negative impact to patients and hospital operations(1) | -1.538 | 1.466 | 1.101 | 1 | .294 | .215 | .012 | 3.802 |
|  | M-Health can be piloted without serious negative impact to patients and hospital operations(2) | -1.436 | .749 | 3.675 | 1 | .055 | .238 | .055 | 1.033 |
|  | M-Health can be piloted without serious negative impact to patients and hospital operations(3) | .334 | .541 | .381 | 1 | .537 | 1.396 | .484 | 4.032 |
|  | It is more strategic to adopt M-Health when the technology is leased to the hospital |  |  | 1.650 | 3 | .648 |  |  |  |
|  | It is more strategic to adopt M-Health when the technology is leased to the hospital(1) | -.271 | .806 | .113 | 1 | .737 | .763 | .157 | 3.703 |
|  | It is more strategic to adopt M-Health when the technology is leased to the hospital(2) | .573 | .798 | .514 | 1 | .473 | 1.773 | .371 | 8.479 |
|  | It is more strategic to adopt M-Health when the technology is leased to the hospital(3) | -.137 | .753 | .033 | 1 | .856 | .872 | .199 | 3.816 |
|  | It is more strategic to adopt M-Health when the hospital fully owned the technology |  |  | 2.234 | 3 | .525 |  |  |  |
|  | It is more strategic to adopt M-Health when the hospital fully owned the technology(1) | .394 | .902 | .191 | 1 | .662 | 1.483 | .253 | 8.689 |
|  | It is more strategic to adopt M-Health when the hospital fully owned the technology(2) | -.578 | .800 | .523 | 1 | .470 | .561 | .117 | 2.690 |
|  | It is more strategic to adopt M-Health when the hospital fully owned the technology(3) | .524 | .611 | .736 | 1 | .391 | 1.689 | .510 | 5.593 |
|  | Constant | 2.761 | 1.594 | 3.001 | 1 | .083 | 15.813 |  |  |
| a. Variable(s) entered on step 1: Use of M-health is superior and more advantageous than current manual practices., M-Health improves efficiency of hospital’s operations , M-health will reduce the cost of health care to patients, M-Health is compatible with current hospital’s health information system and consistent with its values and needs., M-Health can be trusted in terms of its security and confidentiality of patient information, M-Health will make many key staff redundant, M-Health is difficult to understand, use and integrate in the hospital systems and operations, Adoption of M-Health innovations requires exorbitant cost in infrastructure and human resources development, M-Health needs to be piloted first in order to demonstrate that it is better than using current manual systems, M-Health can be piloted without serious negative impact to patients and hospital operations, It is more strategic to adopt M-Health when the technology is leased to the hospital, It is more strategic to adopt M-Health when the hospital fully owned the technology. | | | | | | | | | |

1. *Appointment reminders and technological determinants*

| **Case Processing Summary** | | | |
| --- | --- | --- | --- |
| Unweighted Cases^a^ | | N | Percent |
| Selected Cases | Included in Analysis | 171 | 81.0 |
|  | Missing Cases | 40 | 19.0 |
|  | Total | 211 | 100.0 |
| Unselected Cases | | 0 | .0 |
| Total | | 211 | 100.0 |
| a. If weight is in effect, see classification table for the total number of cases. | | | |

| **Dependent Variable Encoding** | |
| --- | --- |
| Original Value | Internal Value |
| Not adopted | 0 |
| Adopted | 1 |

| **Categorical Variables Codings** | | | | | |
| --- | --- | --- | --- | --- | --- |
|  | | Frequency | Parameter coding | | |
|  |  |  | (1) | (2) | (3) |
| It is more strategic to adopt M-Health when the hospital fully owned the technology | Strongly disgree | 15 | 1.000 | .000 | .000 |
|  | Disagree | 24 | .000 | 1.000 | .000 |
|  | Agree | 63 | .000 | .000 | 1.000 |
|  | Strongly Agree | 69 | .000 | .000 | .000 |
| M-Health improves efficiency of hospital’s operations | Strongly disgree | 6 | 1.000 | .000 | .000 |
|  | Disagree | 9 | .000 | 1.000 | .000 |
|  | Agree | 65 | .000 | .000 | 1.000 |
|  | Strongly Agree | 91 | .000 | .000 | .000 |
| M-health will reduce the cost of health care to patients | Strongly disgree | 8 | 1.000 | .000 | .000 |
|  | Disagree | 25 | .000 | 1.000 | .000 |
|  | Agree | 59 | .000 | .000 | 1.000 |
|  | Strongly Agree | 79 | .000 | .000 | .000 |
| M-Health is compatible with current hospital’s health information system and consistent with its values and needs. | Strongly disgree | 16 | 1.000 | .000 | .000 |
|  | Disagree | 31 | .000 | 1.000 | .000 |
|  | Agree | 68 | .000 | .000 | 1.000 |
|  | Strongly Agree | 56 | .000 | .000 | .000 |
| M-Health can be trusted in terms of its security and confidentiality of patient information | Strongly disgree | 10 | 1.000 | .000 | .000 |
|  | Disagree | 31 | .000 | 1.000 | .000 |
|  | Agree | 86 | .000 | .000 | 1.000 |
|  | Strongly Agree | 44 | .000 | .000 | .000 |
| M-Health will make many key staff redundant | Strongly disgree | 45 | 1.000 | .000 | .000 |
|  | Disagree | 67 | .000 | 1.000 | .000 |
|  | Agree | 39 | .000 | .000 | 1.000 |
|  | Strongly Agree | 20 | .000 | .000 | .000 |
| M-Health is difficult to understand, use and integrate in the hospital systems and operations | Strongly disgree | 55 | 1.000 | .000 | .000 |
|  | Disagree | 78 | .000 | 1.000 | .000 |
|  | Agree | 31 | .000 | .000 | 1.000 |
|  | Strongly Agree | 7 | .000 | .000 | .000 |
| It is more strategic to adopt M-Health when the technology is leased to the hospital | Strongly disgree | 35 | 1.000 | .000 | .000 |
|  | Disagree | 66 | .000 | 1.000 | .000 |
|  | Agree | 43 | .000 | .000 | 1.000 |
|  | Strongly Agree | 27 | .000 | .000 | .000 |
| M-Health can be piloted without serious negative impact to patients and hospital operations | Strongly disgree | 6 | 1.000 | .000 | .000 |
|  | Disagree | 26 | .000 | 1.000 | .000 |
|  | Agree | 87 | .000 | .000 | 1.000 |
|  | Strongly Agree | 52 | .000 | .000 | .000 |
| M-Health needs to be piloted first in order to demonstrate that it is better than using current manual systems | Strongly disgree | 17 | 1.000 | .000 | .000 |
|  | Disagree | 20 | .000 | 1.000 | .000 |
|  | Agree | 61 | .000 | .000 | 1.000 |
|  | Strongly Agree | 73 | .000 | .000 | .000 |
| Adoption of M-Health innovations requires exorbitant cost in infrastructure and human resources development | Strongly disgree | 27 | 1.000 | .000 | .000 |
|  | Disagree | 57 | .000 | 1.000 | .000 |
|  | Agree | 62 | .000 | .000 | 1.000 |
|  | Strongly Agree | 25 | .000 | .000 | .000 |
| Use of M-health is superior and more advantageous than current manual practices. | Strongly disgree | 10 | 1.000 | .000 | .000 |
|  | Disagree | 14 | .000 | 1.000 | .000 |
|  | Agree | 68 | .000 | .000 | 1.000 |
|  | Strongly Agree | 79 | .000 | .000 | .000 |

**Block 0: Beginning Block**

| **Classification Table^a,b^** | | | | | |
| --- | --- | --- | --- | --- | --- |
|  | Observed | | Predicted | | |
|  |  |  | Appointment reminders | | Percentage Correct |
|  |  |  | Not adopted | Adopted |  |
| Step 0 | Appointment reminders | Not adopted | 0 | 43 | .0 |
|  |  | Adopted | 0 | 128 | 100.0 |
|  | Overall Percentage | |  |  | 74.9 |
| a. Constant is included in the model. | | | | | |
| b. The cut value is .500 | | | | | |

| **Variables in the Equation** | | | | | | | |
| --- | --- | --- | --- | --- | --- | --- | --- |
|  | | B | S.E. | Wald | df | Sig. | Exp(B) |
| Step 0 | Constant | 1.091 | .176 | 38.300 | 1 | .000 | 2.977 |

| **Variables not in the Equation** | | | | | |
| --- | --- | --- | --- | --- | --- |
|  | | | Score | df | Sig. |
| Step 0 | Variables | Use of M-health is superior and more advantageous than current manual practices. | 2.311 | 3 | .510 |
|  |  | Use of M-health is superior and more advantageous than current manual practices.(1) | 1.294 | 1 | .255 |
|  |  | Use of M-health is superior and more advantageous than current manual practices.(2) | .095 | 1 | .758 |
|  |  | Use of M-health is superior and more advantageous than current manual practices.(3) | .572 | 1 | .450 |
|  |  | M-Health improves efficiency of hospital’s operations | .353 | 3 | .950 |
|  |  | M-Health improves efficiency of hospital’s operations (1) | .238 | 1 | .626 |
|  |  | M-Health improves efficiency of hospital’s operations (2) | .043 | 1 | .835 |
|  |  | M-Health improves efficiency of hospital’s operations (3) | .016 | 1 | .900 |
|  |  | M-health will reduce the cost of health care to patients | 5.226 | 3 | .156 |
|  |  | M-health will reduce the cost of health care to patients(1) | 2.819 | 1 | .093 |
|  |  | M-health will reduce the cost of health care to patients(2) | 1.833 | 1 | .176 |
|  |  | M-health will reduce the cost of health care to patients(3) | .644 | 1 | .422 |
|  |  | M-Health is compatible with current hospital’s health information system and consistent with its values and needs. | 3.231 | 3 | .357 |
|  |  | M-Health is compatible with current hospital’s health information system and consistent with its values and needs.(1) | 1.500 | 1 | .221 |
|  |  | M-Health is compatible with current hospital’s health information system and consistent with its values and needs.(2) | 2.150 | 1 | .143 |
|  |  | M-Health is compatible with current hospital’s health information system and consistent with its values and needs.(3) | .001 | 1 | .971 |
|  |  | M-Health can be trusted in terms of its security and confidentiality of patient information | .535 | 3 | .911 |
|  |  | M-Health can be trusted in terms of its security and confidentiality of patient information(1) | .133 | 1 | .715 |
|  |  | M-Health can be trusted in terms of its security and confidentiality of patient information(2) | .304 | 1 | .582 |
|  |  | M-Health can be trusted in terms of its security and confidentiality of patient information(3) | .049 | 1 | .825 |
|  |  | M-Health will make many key staff redundant | 1.605 | 3 | .658 |
|  |  | M-Health will make many key staff redundant(1) | .075 | 1 | .784 |
|  |  | M-Health will make many key staff redundant(2) | .604 | 1 | .437 |
|  |  | M-Health will make many key staff redundant(3) | .115 | 1 | .735 |
|  |  | M-Health is difficult to understand, use and integrate in the hospital systems and operations | 3.989 | 3 | .263 |
|  |  | M-Health is difficult to understand, use and integrate in the hospital systems and operations(1) | 2.089 | 1 | .148 |
|  |  | M-Health is difficult to understand, use and integrate in the hospital systems and operations(2) | 3.633 | 1 | .057 |
|  |  | M-Health is difficult to understand, use and integrate in the hospital systems and operations(3) | .675 | 1 | .411 |
|  |  | Adoption of M-Health innovations requires exorbitant cost in infrastructure and human resources development | 2.428 | 3 | .488 |
|  |  | Adoption of M-Health innovations requires exorbitant cost in infrastructure and human resources development(1) | 2.408 | 1 | .121 |
|  |  | Adoption of M-Health innovations requires exorbitant cost in infrastructure and human resources development(2) | .249 | 1 | .618 |
|  |  | Adoption of M-Health innovations requires exorbitant cost in infrastructure and human resources development(3) | .340 | 1 | .560 |
|  |  | M-Health needs to be piloted first in order to demonstrate that it is better than using current manual systems | .493 | 3 | .920 |
|  |  | M-Health needs to be piloted first in order to demonstrate that it is better than using current manual systems(1) | .182 | 1 | .669 |
|  |  | M-Health needs to be piloted first in order to demonstrate that it is better than using current manual systems(2) | .319 | 1 | .572 |
|  |  | M-Health needs to be piloted first in order to demonstrate that it is better than using current manual systems(3) | .059 | 1 | .808 |
|  |  | M-Health can be piloted without serious negative impact to patients and hospital operations | 5.046 | 3 | .168 |
|  |  | M-Health can be piloted without serious negative impact to patients and hospital operations(1) | 2.089 | 1 | .148 |
|  |  | M-Health can be piloted without serious negative impact to patients and hospital operations(2) | 1.461 | 1 | .227 |
|  |  | M-Health can be piloted without serious negative impact to patients and hospital operations(3) | 1.869 | 1 | .172 |
|  |  | It is more strategic to adopt M-Health when the technology is leased to the hospital | 2.026 | 3 | .567 |
|  |  | It is more strategic to adopt M-Health when the technology is leased to the hospital(1) | 1.498 | 1 | .221 |
|  |  | It is more strategic to adopt M-Health when the technology is leased to the hospital(2) | .757 | 1 | .384 |
|  |  | It is more strategic to adopt M-Health when the technology is leased to the hospital(3) | .109 | 1 | .741 |
|  |  | It is more strategic to adopt M-Health when the hospital fully owned the technology | 4.842 | 3 | .184 |
|  |  | It is more strategic to adopt M-Health when the hospital fully owned the technology(1) | 2.983 | 1 | .084 |
|  |  | It is more strategic to adopt M-Health when the hospital fully owned the technology(2) | .994 | 1 | .319 |
|  |  | It is more strategic to adopt M-Health when the hospital fully owned the technology(3) | 1.331 | 1 | .249 |
|  | Overall Statistics | | 33.195 | 36 | .603 |

**Block 1: Method = Enter**

| **Omnibus Tests of Model Coefficients** | | | | |
| --- | --- | --- | --- | --- |
|  | | Chi-square | df | Sig. |
| Step 1 | Step | 43.417 | 36 | .185 |
|  | Block | 43.417 | 36 | .185 |
|  | Model | 43.417 | 36 | .185 |

| **Model Summary** | | | |
| --- | --- | --- | --- |
| Step | -2 Log likelihood | Cox & Snell R Square | Nagelkerke R Square |
| 1 | 149.449^a^ | .224 | .332 |
| a. Estimation terminated at iteration number 20 because maximum iterations has been reached. Final solution cannot be found. | | | |

| **Hosmer and Lemeshow Test** | | | |
| --- | --- | --- | --- |
| Step | Chi-square | df | Sig. |
| 1 | 7.045 | 8 | .532 |

| **Contingency Table for Hosmer and Lemeshow Test** | | | | | | |
| --- | --- | --- | --- | --- | --- | --- |
|  | | Appointment reminders = Not adopted | | Appointment reminders = Adopted | | Total |
|  |  | Observed | Expected | Observed | Expected |  |
| Step 1 | 1 | 13 | 11.235 | 4 | 5.765 | 17 |
|  | 2 | 9 | 8.547 | 8 | 8.453 | 17 |
|  | 3 | 5 | 6.823 | 12 | 10.177 | 17 |
|  | 4 | 3 | 5.248 | 14 | 11.752 | 17 |
|  | 5 | 5 | 4.088 | 12 | 12.912 | 17 |
|  | 6 | 2 | 3.288 | 15 | 13.712 | 17 |
|  | 7 | 4 | 2.056 | 13 | 14.944 | 17 |
|  | 8 | 2 | 1.240 | 15 | 15.760 | 17 |
|  | 9 | 0 | .449 | 17 | 16.551 | 17 |
|  | 10 | 0 | .026 | 18 | 17.974 | 18 |

| **Classification Table^a^** | | | | | |
| --- | --- | --- | --- | --- | --- |
|  | Observed | | Predicted | | |
|  |  |  | Appointment reminders | | Percentage Correct |
|  |  |  | Not adopted | Adopted |  |
| Step 1 | Appointment reminders | Not adopted | 19 | 24 | 44.2 |
|  |  | Adopted | 8 | 120 | 93.8 |
|  | Overall Percentage | |  |  | 81.3 |
| a. The cut value is .500 | | | | | |

| **Variables in the Equation** | | | | | | | | | |
| --- | --- | --- | --- | --- | --- | --- | --- | --- | --- |
|  | | B | S.E. | Wald | df | Sig. | Exp(B) | 95% C.I.for EXP(B) | |
|  |  |  |  |  |  |  |  | Lower | Upper |
| Step 1^a^ | Use of M-health is superior and more advantageous than current manual practices. |  |  | 1.872 | 3 | .599 |  |  |  |
|  | Use of M-health is superior and more advantageous than current manual practices.(1) | .315 | 1.495 | .044 | 1 | .833 | 1.371 | .073 | 25.700 |
|  | Use of M-health is superior and more advantageous than current manual practices.(2) | 1.488 | 1.199 | 1.540 | 1 | .215 | 4.428 | .422 | 46.438 |
|  | Use of M-health is superior and more advantageous than current manual practices.(3) | .713 | .643 | 1.230 | 1 | .267 | 2.039 | .579 | 7.185 |
|  | M-Health improves efficiency of hospital’s operations |  |  | .508 | 3 | .917 |  |  |  |
|  | M-Health improves efficiency of hospital’s operations (1) | -1.197 | 2.378 | .253 | 1 | .615 | .302 | .003 | 31.956 |
|  | M-Health improves efficiency of hospital’s operations (2) | -.505 | 1.351 | .140 | 1 | .708 | .603 | .043 | 8.516 |
|  | M-Health improves efficiency of hospital’s operations (3) | .051 | .705 | .005 | 1 | .942 | 1.052 | .264 | 4.188 |
|  | M-health will reduce the cost of health care to patients |  |  | 1.718 | 3 | .633 |  |  |  |
|  | M-health will reduce the cost of health care to patients(1) | 20.950 | 11576.869 | .000 | 1 | .999 | 1254173572.900 | .000 | . |
|  | M-health will reduce the cost of health care to patients(2) | -1.097 | .843 | 1.694 | 1 | .193 | .334 | .064 | 1.742 |
|  | M-health will reduce the cost of health care to patients(3) | -.336 | .620 | .293 | 1 | .588 | .715 | .212 | 2.409 |
|  | M-Health is compatible with current hospital’s health information system and consistent with its values and needs. |  |  | 3.659 | 3 | .301 |  |  |  |
|  | M-Health is compatible with current hospital’s health information system and consistent with its values and needs.(1) | .748 | 1.131 | .438 | 1 | .508 | 2.112 | .230 | 19.367 |
|  | M-Health is compatible with current hospital’s health information system and consistent with its values and needs.(2) | -.875 | .807 | 1.175 | 1 | .278 | .417 | .086 | 2.028 |
|  | M-Health is compatible with current hospital’s health information system and consistent with its values and needs.(3) | .242 | .645 | .140 | 1 | .708 | 1.273 | .360 | 4.508 |
|  | M-Health can be trusted in terms of its security and confidentiality of patient information |  |  | 1.071 | 3 | .784 |  |  |  |
|  | M-Health can be trusted in terms of its security and confidentiality of patient information(1) | -.155 | 1.224 | .016 | 1 | .899 | .857 | .078 | 9.428 |
|  | M-Health can be trusted in terms of its security and confidentiality of patient information(2) | .722 | .786 | .845 | 1 | .358 | 2.059 | .441 | 9.611 |
|  | M-Health can be trusted in terms of its security and confidentiality of patient information(3) | .484 | .662 | .534 | 1 | .465 | 1.622 | .443 | 5.933 |
|  | M-Health will make many key staff redundant |  |  | 2.789 | 3 | .425 |  |  |  |
|  | M-Health will make many key staff redundant(1) | -1.092 | .946 | 1.331 | 1 | .249 | .336 | .053 | 2.145 |
|  | M-Health will make many key staff redundant(2) | -1.136 | .975 | 1.358 | 1 | .244 | .321 | .047 | 2.170 |
|  | M-Health will make many key staff redundant(3) | -.326 | .997 | .107 | 1 | .743 | .722 | .102 | 5.091 |
|  | M-Health is difficult to understand, use and integrate in the hospital systems and operations |  |  | 9.946 | 3 | .019 |  |  |  |
|  | M-Health is difficult to understand, use and integrate in the hospital systems and operations(1) | 2.952 | 1.398 | 4.456 | 1 | .035 | 19.136 | 1.235 | 296.492 |
|  | M-Health is difficult to understand, use and integrate in the hospital systems and operations(2) | .725 | 1.323 | .300 | 1 | .584 | 2.064 | .154 | 27.599 |
|  | M-Health is difficult to understand, use and integrate in the hospital systems and operations(3) | 1.478 | 1.436 | 1.060 | 1 | .303 | 4.385 | .263 | 73.099 |
|  | Adoption of M-Health innovations requires exorbitant cost in infrastructure and human resources development |  |  | 5.323 | 3 | .150 |  |  |  |
|  | Adoption of M-Health innovations requires exorbitant cost in infrastructure and human resources development(1) | -1.389 | .899 | 2.387 | 1 | .122 | .249 | .043 | 1.452 |
|  | Adoption of M-Health innovations requires exorbitant cost in infrastructure and human resources development(2) | .346 | .749 | .214 | 1 | .644 | 1.414 | .326 | 6.137 |
|  | Adoption of M-Health innovations requires exorbitant cost in infrastructure and human resources development(3) | .593 | .730 | .659 | 1 | .417 | 1.809 | .433 | 7.564 |
|  | M-Health needs to be piloted first in order to demonstrate that it is better than using current manual systems |  |  | .770 | 3 | .857 |  |  |  |
|  | M-Health needs to be piloted first in order to demonstrate that it is better than using current manual systems(1) | .065 | .870 | .006 | 1 | .940 | 1.067 | .194 | 5.874 |
|  | M-Health needs to be piloted first in order to demonstrate that it is better than using current manual systems(2) | .504 | .866 | .339 | 1 | .561 | 1.655 | .303 | 9.033 |
|  | M-Health needs to be piloted first in order to demonstrate that it is better than using current manual systems(3) | .458 | .592 | .599 | 1 | .439 | 1.581 | .496 | 5.041 |
|  | M-Health can be piloted without serious negative impact to patients and hospital operations |  |  | 4.254 | 3 | .235 |  |  |  |
|  | M-Health can be piloted without serious negative impact to patients and hospital operations(1) | 19.702 | 14287.918 | .000 | 1 | .999 | 360196098.454 | .000 | . |
|  | M-Health can be piloted without serious negative impact to patients and hospital operations(2) | -.831 | .768 | 1.169 | 1 | .280 | .436 | .097 | 1.964 |
|  | M-Health can be piloted without serious negative impact to patients and hospital operations(3) | .537 | .576 | .866 | 1 | .352 | 1.710 | .553 | 5.293 |
|  | It is more strategic to adopt M-Health when the technology is leased to the hospital |  |  | 1.402 | 3 | .705 |  |  |  |
|  | It is more strategic to adopt M-Health when the technology is leased to the hospital(1) | .738 | .851 | .753 | 1 | .386 | 2.092 | .395 | 11.092 |
|  | It is more strategic to adopt M-Health when the technology is leased to the hospital(2) | .611 | .795 | .589 | 1 | .443 | 1.842 | .387 | 8.755 |
|  | It is more strategic to adopt M-Health when the technology is leased to the hospital(3) | .889 | .784 | 1.287 | 1 | .257 | 2.433 | .523 | 11.310 |
|  | It is more strategic to adopt M-Health when the hospital fully owned the technology |  |  | 5.677 | 3 | .128 |  |  |  |
|  | It is more strategic to adopt M-Health when the hospital fully owned the technology(1) | 3.142 | 1.660 | 3.582 | 1 | .058 | 23.159 | .894 | 599.891 |
|  | It is more strategic to adopt M-Health when the hospital fully owned the technology(2) | -.623 | .813 | .588 | 1 | .443 | .536 | .109 | 2.637 |
|  | It is more strategic to adopt M-Health when the hospital fully owned the technology(3) | -.815 | .605 | 1.815 | 1 | .178 | .443 | .135 | 1.449 |
|  | Constant | -.769 | 1.484 | .268 | 1 | .604 | .464 |  |  |
| a. Variable(s) entered on step 1: Use of M-health is superior and more advantageous than current manual practices., M-Health improves efficiency of hospital’s operations , M-health will reduce the cost of health care to patients, M-Health is compatible with current hospital’s health information system and consistent with its values and needs., M-Health can be trusted in terms of its security and confidentiality of patient information, M-Health will make many key staff redundant, M-Health is difficult to understand, use and integrate in the hospital systems and operations, Adoption of M-Health innovations requires exorbitant cost in infrastructure and human resources development, M-Health needs to be piloted first in order to demonstrate that it is better than using current manual systems, M-Health can be piloted without serious negative impact to patients and hospital operations, It is more strategic to adopt M-Health when the technology is leased to the hospital, It is more strategic to adopt M-Health when the hospital fully owned the technology. | | | | | | | | | |

1. *Community mobilization and technological determinants*

| **Case Processing Summary** | | | |
| --- | --- | --- | --- |
| Unweighted Cases^a^ | | N | Percent |
| Selected Cases | Included in Analysis | 169 | 80.1 |
|  | Missing Cases | 42 | 19.9 |
|  | Total | 211 | 100.0 |
| Unselected Cases | | 0 | .0 |
| Total | | 211 | 100.0 |
| a. If weight is in effect, see classification table for the total number of cases. | | | |

| **Dependent Variable Encoding** | |
| --- | --- |
| Original Value | Internal Value |
| Not adopted | 0 |
| Adopted | 1 |

| **Categorical Variables Codings** | | | | | |
| --- | --- | --- | --- | --- | --- |
|  | | Frequency | Parameter coding | | |
|  |  |  | (1) | (2) | (3) |
| It is more strategic to adopt M-Health when the hospital fully owned the technology | Strongly disgree | 15 | 1.000 | .000 | .000 |
|  | Disagree | 24 | .000 | 1.000 | .000 |
|  | Agree | 62 | .000 | .000 | 1.000 |
|  | Strongly Agree | 68 | .000 | .000 | .000 |
| M-Health improves efficiency of hospital’s operations | Strongly disgree | 6 | 1.000 | .000 | .000 |
|  | Disagree | 9 | .000 | 1.000 | .000 |
|  | Agree | 65 | .000 | .000 | 1.000 |
|  | Strongly Agree | 89 | .000 | .000 | .000 |
| M-health will reduce the cost of health care to patients | Strongly disgree | 8 | 1.000 | .000 | .000 |
|  | Disagree | 24 | .000 | 1.000 | .000 |
|  | Agree | 58 | .000 | .000 | 1.000 |
|  | Strongly Agree | 79 | .000 | .000 | .000 |
| M-Health is compatible with current hospital’s health information system and consistent with its values and needs. | Strongly disgree | 16 | 1.000 | .000 | .000 |
|  | Disagree | 30 | .000 | 1.000 | .000 |
|  | Agree | 67 | .000 | .000 | 1.000 |
|  | Strongly Agree | 56 | .000 | .000 | .000 |
| M-Health can be trusted in terms of its security and confidentiality of patient information | Strongly disgree | 10 | 1.000 | .000 | .000 |
|  | Disagree | 29 | .000 | 1.000 | .000 |
|  | Agree | 86 | .000 | .000 | 1.000 |
|  | Strongly Agree | 44 | .000 | .000 | .000 |
| M-Health will make many key staff redundant | Strongly disgree | 45 | 1.000 | .000 | .000 |
|  | Disagree | 66 | .000 | 1.000 | .000 |
|  | Agree | 39 | .000 | .000 | 1.000 |
|  | Strongly Agree | 19 | .000 | .000 | .000 |
| M-Health is difficult to understand, use and integrate in the hospital systems and operations | Strongly disgree | 54 | 1.000 | .000 | .000 |
|  | Disagree | 77 | .000 | 1.000 | .000 |
|  | Agree | 31 | .000 | .000 | 1.000 |
|  | Strongly Agree | 7 | .000 | .000 | .000 |
| It is more strategic to adopt M-Health when the technology is leased to the hospital | Strongly disgree | 35 | 1.000 | .000 | .000 |
|  | Disagree | 66 | .000 | 1.000 | .000 |
|  | Agree | 42 | .000 | .000 | 1.000 |
|  | Strongly Agree | 26 | .000 | .000 | .000 |
| M-Health can be piloted without serious negative impact to patients and hospital operations | Strongly disgree | 6 | 1.000 | .000 | .000 |
|  | Disagree | 26 | .000 | 1.000 | .000 |
|  | Agree | 86 | .000 | .000 | 1.000 |
|  | Strongly Agree | 51 | .000 | .000 | .000 |
| M-Health needs to be piloted first in order to demonstrate that it is better than using current manual systems | Strongly disgree | 17 | 1.000 | .000 | .000 |
|  | Disagree | 20 | .000 | 1.000 | .000 |
|  | Agree | 60 | .000 | .000 | 1.000 |
|  | Strongly Agree | 72 | .000 | .000 | .000 |
| Adoption of M-Health innovations requires exorbitant cost in infrastructure and human resources development | Strongly disgree | 26 | 1.000 | .000 | .000 |
|  | Disagree | 56 | .000 | 1.000 | .000 |
|  | Agree | 62 | .000 | .000 | 1.000 |
|  | Strongly Agree | 25 | .000 | .000 | .000 |
| Use of M-health is superior and more advantageous than current manual practices. | Strongly disgree | 10 | 1.000 | .000 | .000 |
|  | Disagree | 14 | .000 | 1.000 | .000 |
|  | Agree | 68 | .000 | .000 | 1.000 |
|  | Strongly Agree | 77 | .000 | .000 | .000 |

**Block 0: Beginning Block**

| **Classification Table^a,b^** | | | | | |
| --- | --- | --- | --- | --- | --- |
|  | Observed | | Predicted | | |
|  |  |  | Community mobilization | | Percentage Correct |
|  |  |  | Not adopted | Adopted |  |
| Step 0 | Community mobilization | Not adopted | 0 | 59 | .0 |
|  |  | Adopted | 0 | 110 | 100.0 |
|  | Overall Percentage | |  |  | 65.1 |
| a. Constant is included in the model. | | | | | |
| b. The cut value is .500 | | | | | |

| **Variables in the Equation** | | | | | | | |
| --- | --- | --- | --- | --- | --- | --- | --- |
|  | | B | S.E. | Wald | df | Sig. | Exp(B) |
| Step 0 | Constant | .623 | .161 | 14.902 | 1 | .000 | 1.864 |

| **Variables not in the Equation** | | | | | |
| --- | --- | --- | --- | --- | --- |
|  | | | Score | df | Sig. |
| Step 0 | Variables | Use of M-health is superior and more advantageous than current manual practices. | 6.169 | 3 | .104 |
|  |  | Use of M-health is superior and more advantageous than current manual practices.(1) | 2.903 | 1 | .088 |
|  |  | Use of M-health is superior and more advantageous than current manual practices.(2) | 1.529 | 1 | .216 |
|  |  | Use of M-health is superior and more advantageous than current manual practices.(3) | 1.966 | 1 | .161 |
|  |  | M-Health improves efficiency of hospital’s operations | 2.985 | 3 | .394 |
|  |  | M-Health improves efficiency of hospital’s operations (1) | .911 | 1 | .340 |
|  |  | M-Health improves efficiency of hospital’s operations (2) | .674 | 1 | .412 |
|  |  | M-Health improves efficiency of hospital’s operations (3) | 2.042 | 1 | .153 |
|  |  | M-health will reduce the cost of health care to patients | 7.333 | 3 | .062 |
|  |  | M-health will reduce the cost of health care to patients(1) | 4.504 | 1 | .034 |
|  |  | M-health will reduce the cost of health care to patients(2) | 1.468 | 1 | .226 |
|  |  | M-health will reduce the cost of health care to patients(3) | 1.626 | 1 | .202 |
|  |  | M-Health is compatible with current hospital’s health information system and consistent with its values and needs. | 4.712 | 3 | .194 |
|  |  | M-Health is compatible with current hospital’s health information system and consistent with its values and needs.(1) | .764 | 1 | .382 |
|  |  | M-Health is compatible with current hospital’s health information system and consistent with its values and needs.(2) | 3.655 | 1 | .056 |
|  |  | M-Health is compatible with current hospital’s health information system and consistent with its values and needs.(3) | .040 | 1 | .841 |
|  |  | M-Health can be trusted in terms of its security and confidentiality of patient information | 5.184 | 3 | .159 |
|  |  | M-Health can be trusted in terms of its security and confidentiality of patient information(1) | .113 | 1 | .737 |
|  |  | M-Health can be trusted in terms of its security and confidentiality of patient information(2) | .827 | 1 | .363 |
|  |  | M-Health can be trusted in terms of its security and confidentiality of patient information(3) | 5.071 | 1 | .024 |
|  |  | M-Health will make many key staff redundant | 4.808 | 3 | .186 |
|  |  | M-Health will make many key staff redundant(1) | 1.835 | 1 | .176 |
|  |  | M-Health will make many key staff redundant(2) | 2.690 | 1 | .101 |
|  |  | M-Health will make many key staff redundant(3) | .281 | 1 | .596 |
|  |  | M-Health is difficult to understand, use and integrate in the hospital systems and operations | 3.384 | 3 | .336 |
|  |  | M-Health is difficult to understand, use and integrate in the hospital systems and operations(1) | 1.777 | 1 | .183 |
|  |  | M-Health is difficult to understand, use and integrate in the hospital systems and operations(2) | 1.021 | 1 | .312 |
|  |  | M-Health is difficult to understand, use and integrate in the hospital systems and operations(3) | .118 | 1 | .732 |
|  |  | Adoption of M-Health innovations requires exorbitant cost in infrastructure and human resources development | 4.556 | 3 | .207 |
|  |  | Adoption of M-Health innovations requires exorbitant cost in infrastructure and human resources development(1) | 3.079 | 1 | .079 |
|  |  | Adoption of M-Health innovations requires exorbitant cost in infrastructure and human resources development(2) | .764 | 1 | .382 |
|  |  | Adoption of M-Health innovations requires exorbitant cost in infrastructure and human resources development(3) | .206 | 1 | .650 |
|  |  | M-Health needs to be piloted first in order to demonstrate that it is better than using current manual systems | 5.296 | 3 | .151 |
|  |  | M-Health needs to be piloted first in order to demonstrate that it is better than using current manual systems(1) | .252 | 1 | .616 |
|  |  | M-Health needs to be piloted first in order to demonstrate that it is better than using current manual systems(2) | 1.016 | 1 | .313 |
|  |  | M-Health needs to be piloted first in order to demonstrate that it is better than using current manual systems(3) | 2.904 | 1 | .088 |
|  |  | M-Health can be piloted without serious negative impact to patients and hospital operations | 2.435 | 3 | .487 |
|  |  | M-Health can be piloted without serious negative impact to patients and hospital operations(1) | .911 | 1 | .340 |
|  |  | M-Health can be piloted without serious negative impact to patients and hospital operations(2) | 1.709 | 1 | .191 |
|  |  | M-Health can be piloted without serious negative impact to patients and hospital operations(3) | .109 | 1 | .741 |
|  |  | It is more strategic to adopt M-Health when the technology is leased to the hospital | 5.812 | 3 | .121 |
|  |  | It is more strategic to adopt M-Health when the technology is leased to the hospital(1) | .236 | 1 | .627 |
|  |  | It is more strategic to adopt M-Health when the technology is leased to the hospital(2) | 2.690 | 1 | .101 |
|  |  | It is more strategic to adopt M-Health when the technology is leased to the hospital(3) | 4.471 | 1 | .034 |
|  |  | It is more strategic to adopt M-Health when the hospital fully owned the technology | 5.133 | 3 | .162 |
|  |  | It is more strategic to adopt M-Health when the hospital fully owned the technology(1) | 1.001 | 1 | .317 |
|  |  | It is more strategic to adopt M-Health when the hospital fully owned the technology(2) | .562 | 1 | .454 |
|  |  | It is more strategic to adopt M-Health when the hospital fully owned the technology(3) | 1.262 | 1 | .261 |
|  | Overall Statistics | | 43.091 | 36 | .194 |

**Block 1: Method = Enter**

| **Omnibus Tests of Model Coefficients** | | | | |
| --- | --- | --- | --- | --- |
|  | | Chi-square | df | Sig. |
| Step 1 | Step | 52.323 | 36 | .039 |
|  | Block | 52.323 | 36 | .039 |
|  | Model | 52.323 | 36 | .039 |

| **Model Summary** | | | |
| --- | --- | --- | --- |
| Step | -2 Log likelihood | Cox & Snell R Square | Nagelkerke R Square |
| 1 | 166.328^a^ | .266 | .367 |
| a. Estimation terminated at iteration number 20 because maximum iterations has been reached. Final solution cannot be found. | | | |

| **Hosmer and Lemeshow Test** | | | |
| --- | --- | --- | --- |
| Step | Chi-square | df | Sig. |
| 1 | 5.690 | 8 | .682 |

| **Contingency Table for Hosmer and Lemeshow Test** | | | | | | |
| --- | --- | --- | --- | --- | --- | --- |
|  | | Community mobilization = Not adopted | | Community mobilization = Adopted | | Total |
|  |  | Observed | Expected | Observed | Expected |  |
| Step 1 | 1 | 14 | 14.122 | 3 | 2.878 | 17 |
|  | 2 | 12 | 11.124 | 5 | 5.876 | 17 |
|  | 3 | 7 | 8.673 | 10 | 8.327 | 17 |
|  | 4 | 8 | 7.264 | 9 | 9.736 | 17 |
|  | 5 | 5 | 5.777 | 12 | 11.223 | 17 |
|  | 6 | 5 | 4.583 | 12 | 12.417 | 17 |
|  | 7 | 3 | 3.628 | 14 | 13.372 | 17 |
|  | 8 | 5 | 2.500 | 12 | 14.500 | 17 |
|  | 9 | 0 | 1.155 | 17 | 15.845 | 17 |
|  | 10 | 0 | .174 | 16 | 15.826 | 16 |

| **Classification Table^a^** | | | | | |
| --- | --- | --- | --- | --- | --- |
|  | Observed | | Predicted | | |
|  |  |  | Community mobilization | | Percentage Correct |
|  |  |  | Not adopted | Adopted |  |
| Step 1 | Community mobilization | Not adopted | 30 | 29 | 50.8 |
|  |  | Adopted | 14 | 96 | 87.3 |
|  | Overall Percentage | |  |  | 74.6 |
| a. The cut value is .500 | | | | | |

| **Variables in the Equation** | | | | | | | | | |
| --- | --- | --- | --- | --- | --- | --- | --- | --- | --- |
|  | | B | S.E. | Wald | df | Sig. | Exp(B) | 95% C.I.for EXP(B) | |
|  |  |  |  |  |  |  |  | Lower | Upper |
| Step 1^a^ | Use of M-health is superior and more advantageous than current manual practices. |  |  | 3.181 | 3 | .364 |  |  |  |
|  | Use of M-health is superior and more advantageous than current manual practices.(1) | 1.011 | 1.820 | .309 | 1 | .578 | 2.749 | .078 | 97.307 |
|  | Use of M-health is superior and more advantageous than current manual practices.(2) | -.575 | 1.045 | .303 | 1 | .582 | .563 | .073 | 4.364 |
|  | Use of M-health is superior and more advantageous than current manual practices.(3) | -.920 | .596 | 2.379 | 1 | .123 | .399 | .124 | 1.283 |
|  | M-Health improves efficiency of hospital’s operations |  |  | 1.125 | 3 | .771 |  |  |  |
|  | M-Health improves efficiency of hospital’s operations (1) | .001 | 2.183 | .000 | 1 | 1.000 | 1.001 | .014 | 72.177 |
|  | M-Health improves efficiency of hospital’s operations (2) | 1.006 | 1.309 | .591 | 1 | .442 | 2.735 | .210 | 35.562 |
|  | M-Health improves efficiency of hospital’s operations (3) | -.208 | .656 | .101 | 1 | .751 | .812 | .225 | 2.937 |
|  | M-health will reduce the cost of health care to patients |  |  | .095 | 3 | .992 |  |  |  |
|  | M-health will reduce the cost of health care to patients(1) | 19.517 | 12894.261 | .000 | 1 | .999 | 299363524.439 | .000 | . |
|  | M-health will reduce the cost of health care to patients(2) | -.040 | .790 | .003 | 1 | .960 | .961 | .204 | 4.521 |
|  | M-health will reduce the cost of health care to patients(3) | .136 | .577 | .055 | 1 | .814 | 1.145 | .369 | 3.550 |
|  | M-Health is compatible with current hospital’s health information system and consistent with its values and needs. |  |  | 2.759 | 3 | .430 |  |  |  |
|  | M-Health is compatible with current hospital’s health information system and consistent with its values and needs.(1) | -.255 | 1.003 | .065 | 1 | .799 | .775 | .108 | 5.537 |
|  | M-Health is compatible with current hospital’s health information system and consistent with its values and needs.(2) | -1.102 | .769 | 2.055 | 1 | .152 | .332 | .074 | 1.499 |
|  | M-Health is compatible with current hospital’s health information system and consistent with its values and needs.(3) | -.147 | .622 | .056 | 1 | .814 | .864 | .255 | 2.923 |
|  | M-Health can be trusted in terms of its security and confidentiality of patient information |  |  | 2.447 | 3 | .485 |  |  |  |
|  | M-Health can be trusted in terms of its security and confidentiality of patient information(1) | -.618 | 1.126 | .301 | 1 | .583 | .539 | .059 | 4.899 |
|  | M-Health can be trusted in terms of its security and confidentiality of patient information(2) | .755 | .806 | .878 | 1 | .349 | 2.129 | .438 | 10.340 |
|  | M-Health can be trusted in terms of its security and confidentiality of patient information(3) | -.134 | .628 | .046 | 1 | .831 | .874 | .255 | 2.995 |
|  | M-Health will make many key staff redundant |  |  | 3.058 | 3 | .383 |  |  |  |
|  | M-Health will make many key staff redundant(1) | .289 | .914 | .100 | 1 | .751 | 1.336 | .223 | 8.007 |
|  | M-Health will make many key staff redundant(2) | -.726 | .916 | .629 | 1 | .428 | .484 | .080 | 2.911 |
|  | M-Health will make many key staff redundant(3) | -.194 | .941 | .042 | 1 | .837 | .824 | .130 | 5.215 |
|  | M-Health is difficult to understand, use and integrate in the hospital systems and operations |  |  | 8.106 | 3 | .044 |  |  |  |
|  | M-Health is difficult to understand, use and integrate in the hospital systems and operations(1) | 3.414 | 1.313 | 6.755 | 1 | .009 | 30.374 | 2.315 | 398.574 |
|  | M-Health is difficult to understand, use and integrate in the hospital systems and operations(2) | 2.339 | 1.276 | 3.363 | 1 | .067 | 10.371 | .851 | 126.347 |
|  | M-Health is difficult to understand, use and integrate in the hospital systems and operations(3) | 2.960 | 1.410 | 4.408 | 1 | .036 | 19.305 | 1.217 | 306.114 |
|  | Adoption of M-Health innovations requires exorbitant cost in infrastructure and human resources development |  |  | 10.857 | 3 | .013 |  |  |  |
|  | Adoption of M-Health innovations requires exorbitant cost in infrastructure and human resources development(1) | -2.274 | .842 | 7.294 | 1 | .007 | .103 | .020 | .536 |
|  | Adoption of M-Health innovations requires exorbitant cost in infrastructure and human resources development(2) | .478 | .765 | .391 | 1 | .532 | 1.613 | .360 | 7.227 |
|  | Adoption of M-Health innovations requires exorbitant cost in infrastructure and human resources development(3) | -.456 | .674 | .459 | 1 | .498 | .634 | .169 | 2.373 |
|  | M-Health needs to be piloted first in order to demonstrate that it is better than using current manual systems |  |  | 1.287 | 3 | .732 |  |  |  |
|  | M-Health needs to be piloted first in order to demonstrate that it is better than using current manual systems(1) | .621 | .833 | .555 | 1 | .456 | 1.860 | .363 | 9.523 |
|  | M-Health needs to be piloted first in order to demonstrate that it is better than using current manual systems(2) | -.467 | .761 | .377 | 1 | .539 | .627 | .141 | 2.784 |
|  | M-Health needs to be piloted first in order to demonstrate that it is better than using current manual systems(3) | .085 | .572 | .022 | 1 | .882 | 1.088 | .355 | 3.337 |
|  | M-Health can be piloted without serious negative impact to patients and hospital operations |  |  | .405 | 3 | .939 |  |  |  |
|  | M-Health can be piloted without serious negative impact to patients and hospital operations(1) | -.229 | 1.459 | .025 | 1 | .875 | .796 | .046 | 13.886 |
|  | M-Health can be piloted without serious negative impact to patients and hospital operations(2) | -.149 | .715 | .043 | 1 | .835 | .862 | .212 | 3.498 |
|  | M-Health can be piloted without serious negative impact to patients and hospital operations(3) | .212 | .525 | .163 | 1 | .686 | 1.237 | .442 | 3.461 |
|  | It is more strategic to adopt M-Health when the technology is leased to the hospital |  |  | 6.649 | 3 | .084 |  |  |  |
|  | It is more strategic to adopt M-Health when the technology is leased to the hospital(1) | .036 | .769 | .002 | 1 | .962 | 1.037 | .230 | 4.679 |
|  | It is more strategic to adopt M-Health when the technology is leased to the hospital(2) | -.226 | .754 | .090 | 1 | .765 | .798 | .182 | 3.497 |
|  | It is more strategic to adopt M-Health when the technology is leased to the hospital(3) | 1.402 | .774 | 3.283 | 1 | .070 | 4.063 | .892 | 18.508 |
|  | It is more strategic to adopt M-Health when the hospital fully owned the technology |  |  | 4.911 | 3 | .178 |  |  |  |
|  | It is more strategic to adopt M-Health when the hospital fully owned the technology(1) | -1.381 | .939 | 2.163 | 1 | .141 | .251 | .040 | 1.583 |
|  | It is more strategic to adopt M-Health when the hospital fully owned the technology(2) | -1.594 | .828 | 3.709 | 1 | .054 | .203 | .040 | 1.029 |
|  | It is more strategic to adopt M-Health when the hospital fully owned the technology(3) | -.787 | .566 | 1.935 | 1 | .164 | .455 | .150 | 1.380 |
|  | Constant | -.414 | 1.460 | .080 | 1 | .777 | .661 |  |  |
| a. Variable(s) entered on step 1: Use of M-health is superior and more advantageous than current manual practices., M-Health improves efficiency of hospital’s operations , M-health will reduce the cost of health care to patients, M-Health is compatible with current hospital’s health information system and consistent with its values and needs., M-Health can be trusted in terms of its security and confidentiality of patient information, M-Health will make many key staff redundant, M-Health is difficult to understand, use and integrate in the hospital systems and operations, Adoption of M-Health innovations requires exorbitant cost in infrastructure and human resources development, M-Health needs to be piloted first in order to demonstrate that it is better than using current manual systems, M-Health can be piloted without serious negative impact to patients and hospital operations, It is more strategic to adopt M-Health when the technology is leased to the hospital, It is more strategic to adopt M-Health when the hospital fully owned the technology. | | | | | | | | | |

1. *Awareness raising over health issues and technological determinants*

| **Case Processing Summary** | | | |
| --- | --- | --- | --- |
| Unweighted Cases^a^ | | N | Percent |
| Selected Cases | Included in Analysis | 168 | 79.6 |
|  | Missing Cases | 43 | 20.4 |
|  | Total | 211 | 100.0 |
| Unselected Cases | | 0 | .0 |
| Total | | 211 | 100.0 |
| a. If weight is in effect, see classification table for the total number of cases. | | | |

| **Dependent Variable Encoding** | |
| --- | --- |
| Original Value | Internal Value |
| Not adopted | 0 |
| Adopted | 1 |

| **Categorical Variables Codings** | | | | | |
| --- | --- | --- | --- | --- | --- |
|  | | Frequency | Parameter coding | | |
|  |  |  | (1) | (2) | (3) |
| It is more strategic to adopt M-Health when the hospital fully owned the technology | Strongly disgree | 14 | 1.000 | .000 | .000 |
|  | Disagree | 23 | .000 | 1.000 | .000 |
|  | Agree | 63 | .000 | .000 | 1.000 |
|  | Strongly Agree | 68 | .000 | .000 | .000 |
| M-Health improves efficiency of hospital’s operations | Strongly disgree | 6 | 1.000 | .000 | .000 |
|  | Disagree | 9 | .000 | 1.000 | .000 |
|  | Agree | 64 | .000 | .000 | 1.000 |
|  | Strongly Agree | 89 | .000 | .000 | .000 |
| M-health will reduce the cost of health care to patients | Strongly disgree | 8 | 1.000 | .000 | .000 |
|  | Disagree | 24 | .000 | 1.000 | .000 |
|  | Agree | 57 | .000 | .000 | 1.000 |
|  | Strongly Agree | 79 | .000 | .000 | .000 |
| M-Health is compatible with current hospital’s health information system and consistent with its values and needs. | Strongly disgree | 16 | 1.000 | .000 | .000 |
|  | Disagree | 30 | .000 | 1.000 | .000 |
|  | Agree | 67 | .000 | .000 | 1.000 |
|  | Strongly Agree | 55 | .000 | .000 | .000 |
| M-Health can be trusted in terms of its security and confidentiality of patient information | Strongly disgree | 10 | 1.000 | .000 | .000 |
|  | Disagree | 30 | .000 | 1.000 | .000 |
|  | Agree | 85 | .000 | .000 | 1.000 |
|  | Strongly Agree | 43 | .000 | .000 | .000 |
| M-Health will make many key staff redundant | Strongly disgree | 44 | 1.000 | .000 | .000 |
|  | Disagree | 67 | .000 | 1.000 | .000 |
|  | Agree | 39 | .000 | .000 | 1.000 |
|  | Strongly Agree | 18 | .000 | .000 | .000 |
| M-Health is difficult to understand, use and integrate in the hospital systems and operations | Strongly disgree | 53 | 1.000 | .000 | .000 |
|  | Disagree | 78 | .000 | 1.000 | .000 |
|  | Agree | 30 | .000 | .000 | 1.000 |
|  | Strongly Agree | 7 | .000 | .000 | .000 |
| It is more strategic to adopt M-Health when the technology is leased to the hospital | Strongly disgree | 35 | 1.000 | .000 | .000 |
|  | Disagree | 66 | .000 | 1.000 | .000 |
|  | Agree | 42 | .000 | .000 | 1.000 |
|  | Strongly Agree | 25 | .000 | .000 | .000 |
| M-Health can be piloted without serious negative impact to patients and hospital operations | Strongly disgree | 6 | 1.000 | .000 | .000 |
|  | Disagree | 26 | .000 | 1.000 | .000 |
|  | Agree | 85 | .000 | .000 | 1.000 |
|  | Strongly Agree | 51 | .000 | .000 | .000 |
| M-Health needs to be piloted first in order to demonstrate that it is better than using current manual systems | Strongly disgree | 17 | 1.000 | .000 | .000 |
|  | Disagree | 20 | .000 | 1.000 | .000 |
|  | Agree | 61 | .000 | .000 | 1.000 |
|  | Strongly Agree | 70 | .000 | .000 | .000 |
| Adoption of M-Health innovations requires exorbitant cost in infrastructure and human resources development | Strongly disgree | 26 | 1.000 | .000 | .000 |
|  | Disagree | 56 | .000 | 1.000 | .000 |
|  | Agree | 61 | .000 | .000 | 1.000 |
|  | Strongly Agree | 25 | .000 | .000 | .000 |
| Use of M-health is superior and more advantageous than current manual practices. | Strongly disgree | 10 | 1.000 | .000 | .000 |
|  | Disagree | 14 | .000 | 1.000 | .000 |
|  | Agree | 67 | .000 | .000 | 1.000 |
|  | Strongly Agree | 77 | .000 | .000 | .000 |

**Block 0: Beginning Block**

| **Classification Table^a,b^** | | | | | |
| --- | --- | --- | --- | --- | --- |
|  | Observed | | Predicted | | |
|  |  |  | Awareness raising over health issues | | Percentage Correct |
|  |  |  | Not adopted | Adopted |  |
| Step 0 | Awareness raising over health issues | Not adopted | 0 | 71 | .0 |
|  |  | Adopted | 0 | 97 | 100.0 |
|  | Overall Percentage | |  |  | 57.7 |
| a. Constant is included in the model. | | | | | |
| b. The cut value is .500 | | | | | |

| **Variables in the Equation** | | | | | | | |
| --- | --- | --- | --- | --- | --- | --- | --- |
|  | | B | S.E. | Wald | df | Sig. | Exp(B) |
| Step 0 | Constant | .312 | .156 | 3.991 | 1 | .046 | 1.366 |

| **Variables not in the Equation** | | | | | |
| --- | --- | --- | --- | --- | --- |
|  | | | Score | df | Sig. |
| Step 0 | Variables | Use of M-health is superior and more advantageous than current manual practices. | 2.995 | 3 | .392 |
|  |  | Use of M-health is superior and more advantageous than current manual practices.(1) | .655 | 1 | .418 |
|  |  | Use of M-health is superior and more advantageous than current manual practices.(2) | 1.386 | 1 | .239 |
|  |  | Use of M-health is superior and more advantageous than current manual practices.(3) | .733 | 1 | .392 |
|  |  | M-Health improves efficiency of hospital’s operations | 4.447 | 3 | .217 |
|  |  | M-Health improves efficiency of hospital’s operations (1) | .153 | 1 | .696 |
|  |  | M-Health improves efficiency of hospital’s operations (2) | .019 | 1 | .892 |
|  |  | M-Health improves efficiency of hospital’s operations (3) | 3.665 | 1 | .056 |
|  |  | M-health will reduce the cost of health care to patients | 3.210 | 3 | .360 |
|  |  | M-health will reduce the cost of health care to patients(1) | .078 | 1 | .780 |
|  |  | M-health will reduce the cost of health care to patients(2) | 2.964 | 1 | .085 |
|  |  | M-health will reduce the cost of health care to patients(3) | .001 | 1 | .977 |
|  |  | M-Health is compatible with current hospital’s health information system and consistent with its values and needs. | 7.055 | 3 | .070 |
|  |  | M-Health is compatible with current hospital’s health information system and consistent with its values and needs.(1) | .164 | 1 | .685 |
|  |  | M-Health is compatible with current hospital’s health information system and consistent with its values and needs.(2) | 6.646 | 1 | .010 |
|  |  | M-Health is compatible with current hospital’s health information system and consistent with its values and needs.(3) | .176 | 1 | .675 |
|  |  | M-Health can be trusted in terms of its security and confidentiality of patient information | 2.375 | 3 | .498 |
|  |  | M-Health can be trusted in terms of its security and confidentiality of patient information(1) | .022 | 1 | .881 |
|  |  | M-Health can be trusted in terms of its security and confidentiality of patient information(2) | .290 | 1 | .590 |
|  |  | M-Health can be trusted in terms of its security and confidentiality of patient information(3) | .924 | 1 | .336 |
|  |  | M-Health will make many key staff redundant | 1.934 | 3 | .586 |
|  |  | M-Health will make many key staff redundant(1) | 1.631 | 1 | .202 |
|  |  | M-Health will make many key staff redundant(2) | .048 | 1 | .827 |
|  |  | M-Health will make many key staff redundant(3) | .868 | 1 | .352 |
|  |  | M-Health is difficult to understand, use and integrate in the hospital systems and operations | 5.924 | 3 | .115 |
|  |  | M-Health is difficult to understand, use and integrate in the hospital systems and operations(1) | .650 | 1 | .420 |
|  |  | M-Health is difficult to understand, use and integrate in the hospital systems and operations(2) | .091 | 1 | .763 |
|  |  | M-Health is difficult to understand, use and integrate in the hospital systems and operations(3) | .017 | 1 | .896 |
|  |  | Adoption of M-Health innovations requires exorbitant cost in infrastructure and human resources development | .558 | 3 | .906 |
|  |  | Adoption of M-Health innovations requires exorbitant cost in infrastructure and human resources development(1) | .000 | 1 | .996 |
|  |  | Adoption of M-Health innovations requires exorbitant cost in infrastructure and human resources development(2) | .012 | 1 | .912 |
|  |  | Adoption of M-Health innovations requires exorbitant cost in infrastructure and human resources development(3) | .334 | 1 | .563 |
|  |  | M-Health needs to be piloted first in order to demonstrate that it is better than using current manual systems | 3.929 | 3 | .269 |
|  |  | M-Health needs to be piloted first in order to demonstrate that it is better than using current manual systems(1) | 1.280 | 1 | .258 |
|  |  | M-Health needs to be piloted first in order to demonstrate that it is better than using current manual systems(2) | 1.510 | 1 | .219 |
|  |  | M-Health needs to be piloted first in order to demonstrate that it is better than using current manual systems(3) | 1.094 | 1 | .296 |
|  |  | M-Health can be piloted without serious negative impact to patients and hospital operations | 10.847 | 3 | .013 |
|  |  | M-Health can be piloted without serious negative impact to patients and hospital operations(1) | 4.554 | 1 | .033 |
|  |  | M-Health can be piloted without serious negative impact to patients and hospital operations(2) | 6.740 | 1 | .009 |
|  |  | M-Health can be piloted without serious negative impact to patients and hospital operations(3) | 1.502 | 1 | .220 |
|  |  | It is more strategic to adopt M-Health when the technology is leased to the hospital | .774 | 3 | .856 |
|  |  | It is more strategic to adopt M-Health when the technology is leased to the hospital(1) | .006 | 1 | .936 |
|  |  | It is more strategic to adopt M-Health when the technology is leased to the hospital(2) | .366 | 1 | .545 |
|  |  | It is more strategic to adopt M-Health when the technology is leased to the hospital(3) | .659 | 1 | .417 |
|  |  | It is more strategic to adopt M-Health when the hospital fully owned the technology | 2.235 | 3 | .525 |
|  |  | It is more strategic to adopt M-Health when the hospital fully owned the technology(1) | 1.386 | 1 | .239 |
|  |  | It is more strategic to adopt M-Health when the hospital fully owned the technology(2) | .016 | 1 | .899 |
|  |  | It is more strategic to adopt M-Health when the hospital fully owned the technology(3) | 1.368 | 1 | .242 |
|  | Overall Statistics | | 40.379 | 36 | .283 |

**Block 1: Method = Enter**

| **Omnibus Tests of Model Coefficients** | | | | |
| --- | --- | --- | --- | --- |
|  | | Chi-square | df | Sig. |
| Step 1 | Step | 51.890 | 36 | .042 |
|  | Block | 51.890 | 36 | .042 |
|  | Model | 51.890 | 36 | .042 |

| **Model Summary** | | | |
| --- | --- | --- | --- |
| Step | -2 Log likelihood | Cox & Snell R Square | Nagelkerke R Square |
| 1 | 176.967^a^ | .266 | .357 |
| a. Estimation terminated at iteration number 20 because maximum iterations has been reached. Final solution cannot be found. | | | |

| **Hosmer and Lemeshow Test** | | | |
| --- | --- | --- | --- |
| Step | Chi-square | df | Sig. |
| 1 | 3.093 | 8 | .928 |

| **Contingency Table for Hosmer and Lemeshow Test** | | | | | | |
| --- | --- | --- | --- | --- | --- | --- |
|  | | Awareness raising over health issues = Not adopted | | Awareness raising over health issues = Adopted | | Total |
|  |  | Observed | Expected | Observed | Expected |  |
| Step 1 | 1 | 16 | 15.470 | 1 | 1.530 | 17 |
|  | 2 | 13 | 11.903 | 4 | 5.097 | 17 |
|  | 3 | 10 | 10.164 | 7 | 6.836 | 17 |
|  | 4 | 7 | 8.560 | 10 | 8.440 | 17 |
|  | 5 | 7 | 7.067 | 10 | 9.933 | 17 |
|  | 6 | 4 | 6.012 | 13 | 10.988 | 17 |
|  | 7 | 6 | 5.099 | 11 | 11.901 | 17 |
|  | 8 | 4 | 3.926 | 13 | 13.074 | 17 |
|  | 9 | 3 | 2.267 | 14 | 14.733 | 17 |
|  | 10 | 1 | .531 | 14 | 14.469 | 15 |

| **Classification Table^a^** | | | | | |
| --- | --- | --- | --- | --- | --- |
|  | Observed | | Predicted | | |
|  |  |  | Awareness raising over health issues | | Percentage Correct |
|  |  |  | Not adopted | Adopted |  |
| Step 1 | Awareness raising over health issues | Not adopted | 42 | 29 | 59.2 |
|  |  | Adopted | 17 | 80 | 82.5 |
|  | Overall Percentage | |  |  | 72.6 |
| a. The cut value is .500 | | | | | |

| **Variables in the Equation** | | | | | | | | | |
| --- | --- | --- | --- | --- | --- | --- | --- | --- | --- |
|  | | B | S.E. | Wald | df | Sig. | Exp(B) | 95% C.I.for EXP(B) | |
|  |  |  |  |  |  |  |  | Lower | Upper |
| Step 1^a^ | Use of M-health is superior and more advantageous than current manual practices. |  |  | .992 | 3 | .803 |  |  |  |
|  | Use of M-health is superior and more advantageous than current manual practices.(1) | 1.534 | 1.553 | .975 | 1 | .323 | 4.635 | .221 | 97.332 |
|  | Use of M-health is superior and more advantageous than current manual practices.(2) | .130 | 1.017 | .016 | 1 | .898 | 1.138 | .155 | 8.349 |
|  | Use of M-health is superior and more advantageous than current manual practices.(3) | .175 | .556 | .099 | 1 | .753 | 1.192 | .401 | 3.543 |
|  | M-Health improves efficiency of hospital’s operations |  |  | 3.115 | 3 | .374 |  |  |  |
|  | M-Health improves efficiency of hospital’s operations (1) | -1.734 | 2.181 | .632 | 1 | .427 | .177 | .002 | 12.691 |
|  | M-Health improves efficiency of hospital’s operations (2) | .580 | 1.187 | .239 | 1 | .625 | 1.787 | .175 | 18.290 |
|  | M-Health improves efficiency of hospital’s operations (3) | -.696 | .625 | 1.243 | 1 | .265 | .498 | .146 | 1.695 |
|  | M-health will reduce the cost of health care to patients |  |  | .486 | 3 | .922 |  |  |  |
|  | M-health will reduce the cost of health care to patients(1) | -.387 | 1.393 | .077 | 1 | .781 | .679 | .044 | 10.417 |
|  | M-health will reduce the cost of health care to patients(2) | -.218 | .750 | .085 | 1 | .771 | .804 | .185 | 3.495 |
|  | M-health will reduce the cost of health care to patients(3) | -.385 | .571 | .455 | 1 | .500 | .681 | .222 | 2.083 |
|  | M-Health is compatible with current hospital’s health information system and consistent with its values and needs. |  |  | 8.438 | 3 | .038 |  |  |  |
|  | M-Health is compatible with current hospital’s health information system and consistent with its values and needs.(1) | -.507 | .868 | .340 | 1 | .560 | .603 | .110 | 3.305 |
|  | M-Health is compatible with current hospital’s health information system and consistent with its values and needs.(2) | -2.057 | .755 | 7.427 | 1 | .006 | .128 | .029 | .561 |
|  | M-Health is compatible with current hospital’s health information system and consistent with its values and needs.(3) | -.495 | .567 | .763 | 1 | .382 | .609 | .200 | 1.852 |
|  | M-Health can be trusted in terms of its security and confidentiality of patient information |  |  | 2.443 | 3 | .486 |  |  |  |
|  | M-Health can be trusted in terms of its security and confidentiality of patient information(1) | -.939 | 1.140 | .679 | 1 | .410 | .391 | .042 | 3.654 |
|  | M-Health can be trusted in terms of its security and confidentiality of patient information(2) | .620 | .716 | .750 | 1 | .386 | 1.859 | .457 | 7.557 |
|  | M-Health can be trusted in terms of its security and confidentiality of patient information(3) | -.062 | .570 | .012 | 1 | .913 | .940 | .307 | 2.872 |
|  | M-Health will make many key staff redundant |  |  | .369 | 3 | .946 |  |  |  |
|  | M-Health will make many key staff redundant(1) | .444 | .835 | .283 | 1 | .595 | 1.559 | .304 | 8.010 |
|  | M-Health will make many key staff redundant(2) | .351 | .857 | .168 | 1 | .682 | 1.421 | .265 | 7.619 |
|  | M-Health will make many key staff redundant(3) | .498 | .883 | .317 | 1 | .573 | 1.645 | .291 | 9.289 |
|  | M-Health is difficult to understand, use and integrate in the hospital systems and operations |  |  | .782 | 3 | .854 |  |  |  |
|  | M-Health is difficult to understand, use and integrate in the hospital systems and operations(1) | 21.592 | 9802.240 | .000 | 1 | .998 | 2382798017.142 | .000 | . |
|  | M-Health is difficult to understand, use and integrate in the hospital systems and operations(2) | 22.068 | 9802.240 | .000 | 1 | .998 | 3837930347.536 | .000 | . |
|  | M-Health is difficult to understand, use and integrate in the hospital systems and operations(3) | 21.885 | 9802.240 | .000 | 1 | .998 | 3194632155.136 | .000 | . |
|  | Adoption of M-Health innovations requires exorbitant cost in infrastructure and human resources development |  |  | 1.032 | 3 | .793 |  |  |  |
|  | Adoption of M-Health innovations requires exorbitant cost in infrastructure and human resources development(1) | .028 | .757 | .001 | 1 | .971 | 1.028 | .233 | 4.530 |
|  | Adoption of M-Health innovations requires exorbitant cost in infrastructure and human resources development(2) | -.026 | .665 | .002 | 1 | .969 | .975 | .265 | 3.586 |
|  | Adoption of M-Health innovations requires exorbitant cost in infrastructure and human resources development(3) | .444 | .654 | .461 | 1 | .497 | 1.559 | .433 | 5.616 |
|  | M-Health needs to be piloted first in order to demonstrate that it is better than using current manual systems |  |  | 2.716 | 3 | .438 |  |  |  |
|  | M-Health needs to be piloted first in order to demonstrate that it is better than using current manual systems(1) | 1.130 | .866 | 1.702 | 1 | .192 | 3.095 | .567 | 16.901 |
|  | M-Health needs to be piloted first in order to demonstrate that it is better than using current manual systems(2) | -.231 | .720 | .103 | 1 | .749 | .794 | .194 | 3.256 |
|  | M-Health needs to be piloted first in order to demonstrate that it is better than using current manual systems(3) | -.262 | .526 | .247 | 1 | .619 | .770 | .274 | 2.159 |
|  | M-Health can be piloted without serious negative impact to patients and hospital operations |  |  | 6.782 | 3 | .079 |  |  |  |
|  | M-Health can be piloted without serious negative impact to patients and hospital operations(1) | 42.084 | 15639.691 | .000 | 1 | .998 | 1892543116364291580.000 | .000 | . |
|  | M-Health can be piloted without serious negative impact to patients and hospital operations(2) | -1.461 | .677 | 4.654 | 1 | .031 | .232 | .061 | .875 |
|  | M-Health can be piloted without serious negative impact to patients and hospital operations(3) | .161 | .496 | .105 | 1 | .745 | 1.175 | .444 | 3.105 |
|  | It is more strategic to adopt M-Health when the technology is leased to the hospital |  |  | 2.822 | 3 | .420 |  |  |  |
|  | It is more strategic to adopt M-Health when the technology is leased to the hospital(1) | -.728 | .795 | .840 | 1 | .360 | .483 | .102 | 2.293 |
|  | It is more strategic to adopt M-Health when the technology is leased to the hospital(2) | -.697 | .791 | .777 | 1 | .378 | .498 | .106 | 2.345 |
|  | It is more strategic to adopt M-Health when the technology is leased to the hospital(3) | -1.268 | .777 | 2.667 | 1 | .102 | .281 | .061 | 1.289 |
|  | It is more strategic to adopt M-Health when the hospital fully owned the technology |  |  | 4.653 | 3 | .199 |  |  |  |
|  | It is more strategic to adopt M-Health when the hospital fully owned the technology(1) | -1.072 | .981 | 1.193 | 1 | .275 | .342 | .050 | 2.343 |
|  | It is more strategic to adopt M-Health when the hospital fully owned the technology(2) | .910 | .804 | 1.281 | 1 | .258 | 2.485 | .514 | 12.023 |
|  | It is more strategic to adopt M-Health when the hospital fully owned the technology(3) | .751 | .530 | 2.006 | 1 | .157 | 2.120 | .750 | 5.994 |
|  | Constant | -20.495 | 9802.240 | .000 | 1 | .998 | .000 |  |  |
| a. Variable(s) entered on step 1: Use of M-health is superior and more advantageous than current manual practices., M-Health improves efficiency of hospital’s operations , M-health will reduce the cost of health care to patients, M-Health is compatible with current hospital’s health information system and consistent with its values and needs., M-Health can be trusted in terms of its security and confidentiality of patient information, M-Health will make many key staff redundant, M-Health is difficult to understand, use and integrate in the hospital systems and operations, Adoption of M-Health innovations requires exorbitant cost in infrastructure and human resources development, M-Health needs to be piloted first in order to demonstrate that it is better than using current manual systems, M-Health can be piloted without serious negative impact to patients and hospital operations, It is more strategic to adopt M-Health when the technology is leased to the hospital, It is more strategic to adopt M-Health when the hospital fully owned the technology. | | | | | | | | | |

1. *Mobile telemedicine and technological determinants*

LOGISTIC REGRESSION VARIABLES Q20.7

/METHOD=ENTER Q23.1 Q23.2 Q23.3 Q23.4 Q23.5 Q23.6 Q23.7 Q23.8 Q23.9 Q23.10 Q23.11 Q23.12

/CONTRAST (Q23.1)=Indicator

/CONTRAST (Q23.2)=Indicator

/CONTRAST (Q23.3)=Indicator

/CONTRAST (Q23.4)=Indicator

/CONTRAST (Q23.5)=Indicator

/CONTRAST (Q23.6)=Indicator

/CONTRAST (Q23.7)=Indicator

/CONTRAST (Q23.8)=Indicator

/CONTRAST (Q23.9)=Indicator

/CONTRAST (Q23.10)=Indicator

/CONTRAST (Q23.11)=Indicator

/CONTRAST (Q23.12)=Indicator

/SAVE=PGROUP

/PRINT=GOODFIT SUMMARY CI(95)

/CRITERIA=PIN(0.05) POUT(0.10) ITERATE(20) CUT(0.5).

**Logistic Regression**

| **Notes** | | |
| --- | --- | --- |
| Output Created | | 15-JUL-2018 13:34:26 |
| Comments | |  |
| Input | Data | C:\Users\Ocholap\Downloads\m-health data.- 13-JULY 2018.sav |
|  | Active Dataset | DataSet1 |
|  | Filter | <none> |
|  | Weight | <none> |
|  | Split File | <none> |
|  | N of Rows in Working Data File | 211 |
| Missing Value Handling | Definition of Missing | User-defined missing values are treated as missing |
| Syntax | | LOGISTIC REGRESSION VARIABLES Q20.7  /METHOD=ENTER Q23.1 Q23.2 Q23.3 Q23.4 Q23.5 Q23.6 Q23.7 Q23.8 Q23.9 Q23.10 Q23.11 Q23.12  /CONTRAST (Q23.1)=Indicator  /CONTRAST (Q23.2)=Indicator  /CONTRAST (Q23.3)=Indicator  /CONTRAST (Q23.4)=Indicator  /CONTRAST (Q23.5)=Indicator  /CONTRAST (Q23.6)=Indicator  /CONTRAST (Q23.7)=Indicator  /CONTRAST (Q23.8)=Indicator  /CONTRAST (Q23.9)=Indicator  /CONTRAST (Q23.10)=Indicator  /CONTRAST (Q23.11)=Indicator  /CONTRAST (Q23.12)=Indicator  /SAVE=PGROUP  /PRINT=GOODFIT SUMMARY CI(95)  /CRITERIA=PIN(0.05) POUT(0.10) ITERATE(20) CUT(0.5). |
| Resources | Processor Time | 00:00:00.06 |
|  | Elapsed Time | 00:00:00.28 |
| Variables Created or Modified | PGR_21 | Predicted group |

| **Case Processing Summary** | | | |
| --- | --- | --- | --- |
| Unweighted Cases^a^ | | N | Percent |
| Selected Cases | Included in Analysis | 170 | 80.6 |
|  | Missing Cases | 41 | 19.4 |
|  | Total | 211 | 100.0 |
| Unselected Cases | | 0 | .0 |
| Total | | 211 | 100.0 |
| a. If weight is in effect, see classification table for the total number of cases. | | | |

| **Dependent Variable Encoding** | |
| --- | --- |
| Original Value | Internal Value |
| Not adopted | 0 |
| Adopted | 1 |

| **Categorical Variables Codings** | | | | | |
| --- | --- | --- | --- | --- | --- |
|  | | Frequency | Parameter coding | | |
|  |  |  | (1) | (2) | (3) |
| It is more strategic to adopt M-Health when the hospital fully owned the technology | Strongly disgree | 15 | 1.000 | .000 | .000 |
|  | Disagree | 24 | .000 | 1.000 | .000 |
|  | Agree | 62 | .000 | .000 | 1.000 |
|  | Strongly Agree | 69 | .000 | .000 | .000 |
| M-Health improves efficiency of hospital’s operations | Strongly disgree | 6 | 1.000 | .000 | .000 |
|  | Disagree | 9 | .000 | 1.000 | .000 |
|  | Agree | 64 | .000 | .000 | 1.000 |
|  | Strongly Agree | 91 | .000 | .000 | .000 |
| M-health will reduce the cost of health care to patients | Strongly disgree | 8 | 1.000 | .000 | .000 |
|  | Disagree | 24 | .000 | 1.000 | .000 |
|  | Agree | 59 | .000 | .000 | 1.000 |
|  | Strongly Agree | 79 | .000 | .000 | .000 |
| M-Health is compatible with current hospital’s health information system and consistent with its values and needs. | Strongly disgree | 16 | 1.000 | .000 | .000 |
|  | Disagree | 30 | .000 | 1.000 | .000 |
|  | Agree | 68 | .000 | .000 | 1.000 |
|  | Strongly Agree | 56 | .000 | .000 | .000 |
| M-Health can be trusted in terms of its security and confidentiality of patient information | Strongly disgree | 10 | 1.000 | .000 | .000 |
|  | Disagree | 31 | .000 | 1.000 | .000 |
|  | Agree | 85 | .000 | .000 | 1.000 |
|  | Strongly Agree | 44 | .000 | .000 | .000 |
| M-Health will make many key staff redundant | Strongly disgree | 45 | 1.000 | .000 | .000 |
|  | Disagree | 66 | .000 | 1.000 | .000 |
|  | Agree | 39 | .000 | .000 | 1.000 |
|  | Strongly Agree | 20 | .000 | .000 | .000 |
| M-Health is difficult to understand, use and integrate in the hospital systems and operations | Strongly disgree | 55 | 1.000 | .000 | .000 |
|  | Disagree | 78 | .000 | 1.000 | .000 |
|  | Agree | 30 | .000 | .000 | 1.000 |
|  | Strongly Agree | 7 | .000 | .000 | .000 |
| It is more strategic to adopt M-Health when the technology is leased to the hospital | Strongly disgree | 34 | 1.000 | .000 | .000 |
|  | Disagree | 66 | .000 | 1.000 | .000 |
|  | Agree | 43 | .000 | .000 | 1.000 |
|  | Strongly Agree | 27 | .000 | .000 | .000 |
| M-Health can be piloted without serious negative impact to patients and hospital operations | Strongly disgree | 6 | 1.000 | .000 | .000 |
|  | Disagree | 26 | .000 | 1.000 | .000 |
|  | Agree | 86 | .000 | .000 | 1.000 |
|  | Strongly Agree | 52 | .000 | .000 | .000 |
| M-Health needs to be piloted first in order to demonstrate that it is better than using current manual systems | Strongly disgree | 17 | 1.000 | .000 | .000 |
|  | Disagree | 20 | .000 | 1.000 | .000 |
|  | Agree | 60 | .000 | .000 | 1.000 |
|  | Strongly Agree | 73 | .000 | .000 | .000 |
| Adoption of M-Health innovations requires exorbitant cost in infrastructure and human resources development | Strongly disgree | 27 | 1.000 | .000 | .000 |
|  | Disagree | 57 | .000 | 1.000 | .000 |
|  | Agree | 61 | .000 | .000 | 1.000 |
|  | Strongly Agree | 25 | .000 | .000 | .000 |
| Use of M-health is superior and more advantageous than current manual practices. | Strongly disgree | 10 | 1.000 | .000 | .000 |
|  | Disagree | 14 | .000 | 1.000 | .000 |
|  | Agree | 67 | .000 | .000 | 1.000 |
|  | Strongly Agree | 79 | .000 | .000 | .000 |

**Block 0: Beginning Block**

| **Classification Table^a,b^** | | | | | |
| --- | --- | --- | --- | --- | --- |
|  | Observed | | Predicted | | |
|  |  |  | Mobile telemedicine | | Percentage Correct |
|  |  |  | Not adopted | Adopted |  |
| Step 0 | Mobile telemedicine | Not adopted | 0 | 85 | .0 |
|  |  | Adopted | 0 | 85 | 100.0 |
|  | Overall Percentage | |  |  | 50.0 |
| a. Constant is included in the model. | | | | | |
| b. The cut value is .500 | | | | | |

| **Variables in the Equation** | | | | | | | |
| --- | --- | --- | --- | --- | --- | --- | --- |
|  | | B | S.E. | Wald | df | Sig. | Exp(B) |
| Step 0 | Constant | .000 | .153 | .000 | 1 | 1.000 | 1.000 |

| **Variables not in the Equation** | | | | | |
| --- | --- | --- | --- | --- | --- |
|  | | | Score | df | Sig. |
| Step 0 | Variables | Use of M-health is superior and more advantageous than current manual practices. | 4.861 | 3 | .182 |
|  |  | Use of M-health is superior and more advantageous than current manual practices.(1) | 1.700 | 1 | .192 |
|  |  | Use of M-health is superior and more advantageous than current manual practices.(2) | 2.802 | 1 | .094 |
|  |  | Use of M-health is superior and more advantageous than current manual practices.(3) | .616 | 1 | .433 |
|  |  | M-Health improves efficiency of hospital’s operations | 1.615 | 3 | .656 |
|  |  | M-Health improves efficiency of hospital’s operations (1) | .691 | 1 | .406 |
|  |  | M-Health improves efficiency of hospital’s operations (2) | .117 | 1 | .732 |
|  |  | M-Health improves efficiency of hospital’s operations (3) | .902 | 1 | .342 |
|  |  | M-health will reduce the cost of health care to patients | 3.338 | 3 | .342 |
|  |  | M-health will reduce the cost of health care to patients(1) | .000 | 1 | 1.000 |
|  |  | M-health will reduce the cost of health care to patients(2) | .776 | 1 | .378 |
|  |  | M-health will reduce the cost of health care to patients(3) | 3.141 | 1 | .076 |
|  |  | M-Health is compatible with current hospital’s health information system and consistent with its values and needs. | .440 | 3 | .932 |
|  |  | M-Health is compatible with current hospital’s health information system and consistent with its values and needs.(1) | .000 | 1 | 1.000 |
|  |  | M-Health is compatible with current hospital’s health information system and consistent with its values and needs.(2) | .162 | 1 | .687 |
|  |  | M-Health is compatible with current hospital’s health information system and consistent with its values and needs.(3) | .392 | 1 | .531 |
|  |  | M-Health can be trusted in terms of its security and confidentiality of patient information | .044 | 3 | .998 |
|  |  | M-Health can be trusted in terms of its security and confidentiality of patient information(1) | .000 | 1 | 1.000 |
|  |  | M-Health can be trusted in terms of its security and confidentiality of patient information(2) | .039 | 1 | .843 |
|  |  | M-Health can be trusted in terms of its security and confidentiality of patient information(3) | .024 | 1 | .878 |
|  |  | M-Health will make many key staff redundant | .998 | 3 | .802 |
|  |  | M-Health will make many key staff redundant(1) | .030 | 1 | .862 |
|  |  | M-Health will make many key staff redundant(2) | .892 | 1 | .345 |
|  |  | M-Health will make many key staff redundant(3) | .299 | 1 | .584 |
|  |  | M-Health is difficult to understand, use and integrate in the hospital systems and operations | 2.285 | 3 | .515 |
|  |  | M-Health is difficult to understand, use and integrate in the hospital systems and operations(1) | 1.317 | 1 | .251 |
|  |  | M-Health is difficult to understand, use and integrate in the hospital systems and operations(2) | .095 | 1 | .758 |
|  |  | M-Health is difficult to understand, use and integrate in the hospital systems and operations(3) | 1.457 | 1 | .227 |
|  |  | Adoption of M-Health innovations requires exorbitant cost in infrastructure and human resources development | 1.492 | 3 | .684 |
|  |  | Adoption of M-Health innovations requires exorbitant cost in infrastructure and human resources development(1) | .044 | 1 | .834 |
|  |  | Adoption of M-Health innovations requires exorbitant cost in infrastructure and human resources development(2) | .660 | 1 | .417 |
|  |  | Adoption of M-Health innovations requires exorbitant cost in infrastructure and human resources development(3) | .026 | 1 | .873 |
|  |  | M-Health needs to be piloted first in order to demonstrate that it is better than using current manual systems | 1.582 | 3 | .663 |
|  |  | M-Health needs to be piloted first in order to demonstrate that it is better than using current manual systems(1) | .065 | 1 | .798 |
|  |  | M-Health needs to be piloted first in order to demonstrate that it is better than using current manual systems(2) | .907 | 1 | .341 |
|  |  | M-Health needs to be piloted first in order to demonstrate that it is better than using current manual systems(3) | .927 | 1 | .336 |
|  |  | M-Health can be piloted without serious negative impact to patients and hospital operations | 10.592 | 3 | .014 |
|  |  | M-Health can be piloted without serious negative impact to patients and hospital operations(1) | .000 | 1 | 1.000 |
|  |  | M-Health can be piloted without serious negative impact to patients and hospital operations(2) | 8.900 | 1 | .003 |
|  |  | M-Health can be piloted without serious negative impact to patients and hospital operations(3) | 6.024 | 1 | .014 |
|  |  | It is more strategic to adopt M-Health when the technology is leased to the hospital | .778 | 3 | .855 |
|  |  | It is more strategic to adopt M-Health when the technology is leased to the hospital(1) | .588 | 1 | .443 |
|  |  | It is more strategic to adopt M-Health when the technology is leased to the hospital(2) | .099 | 1 | .753 |
|  |  | It is more strategic to adopt M-Health when the technology is leased to the hospital(3) | .280 | 1 | .597 |
|  |  | It is more strategic to adopt M-Health when the hospital fully owned the technology | 3.044 | 3 | .385 |
|  |  | It is more strategic to adopt M-Health when the hospital fully owned the technology(1) | 1.828 | 1 | .176 |
|  |  | It is more strategic to adopt M-Health when the hospital fully owned the technology(2) | .776 | 1 | .378 |
|  |  | It is more strategic to adopt M-Health when the hospital fully owned the technology(3) | .914 | 1 | .339 |
|  | Overall Statistics | | 32.928 | 36 | .615 |

**Block 1: Method = Enter**

| **Omnibus Tests of Model Coefficients** | | | | |
| --- | --- | --- | --- | --- |
|  | | Chi-square | df | Sig. |
| Step 1 | Step | 36.506 | 36 | .445 |
|  | Block | 36.506 | 36 | .445 |
|  | Model | 36.506 | 36 | .445 |

| **Model Summary** | | | |
| --- | --- | --- | --- |
| Step | -2 Log likelihood | Cox & Snell R Square | Nagelkerke R Square |
| 1 | 199.164^a^ | .193 | .258 |
| a. Estimation terminated at iteration number 4 because parameter estimates changed by less than .001. | | | |

| **Hosmer and Lemeshow Test** | | | |
| --- | --- | --- | --- |
| Step | Chi-square | df | Sig. |
| 1 | 9.000 | 8 | .342 |

| **Contingency Table for Hosmer and Lemeshow Test** | | | | | | |
| --- | --- | --- | --- | --- | --- | --- |
|  | | Mobile telemedicine = Not adopted | | Mobile telemedicine = Adopted | | Total |
|  |  | Observed | Expected | Observed | Expected |  |
| Step 1 | 1 | 15 | 15.122 | 2 | 1.878 | 17 |
|  | 2 | 13 | 12.881 | 4 | 4.119 | 17 |
|  | 3 | 10 | 11.697 | 7 | 5.303 | 17 |
|  | 4 | 12 | 10.191 | 5 | 6.809 | 17 |
|  | 5 | 12 | 8.668 | 5 | 8.332 | 17 |
|  | 6 | 7 | 7.458 | 10 | 9.542 | 17 |
|  | 7 | 5 | 6.404 | 12 | 10.596 | 17 |
|  | 8 | 6 | 5.276 | 11 | 11.724 | 17 |
|  | 9 | 1 | 4.419 | 16 | 12.581 | 17 |
|  | 10 | 4 | 2.884 | 13 | 14.116 | 17 |

| **Classification Table^a^** | | | | | |
| --- | --- | --- | --- | --- | --- |
|  | Observed | | Predicted | | |
|  |  |  | Mobile telemedicine | | Percentage Correct |
|  |  |  | Not adopted | Adopted |  |
| Step 1 | Mobile telemedicine | Not adopted | 58 | 27 | 68.2 |
|  |  | Adopted | 20 | 65 | 76.5 |
|  | Overall Percentage | |  |  | 72.4 |
| a. The cut value is .500 | | | | | |

| **Variables in the Equation** | | | | | | | | | |
| --- | --- | --- | --- | --- | --- | --- | --- | --- | --- |
|  | | B | S.E. | Wald | df | Sig. | Exp(B) | 95% C.I.for EXP(B) | |
|  |  |  |  |  |  |  |  | Lower | Upper |
| Step 1^a^ | Use of M-health is superior and more advantageous than current manual practices. |  |  | 5.424 | 3 | .143 |  |  |  |
|  | Use of M-health is superior and more advantageous than current manual practices.(1) | -1.511 | 1.146 | 1.740 | 1 | .187 | .221 | .023 | 2.084 |
|  | Use of M-health is superior and more advantageous than current manual practices.(2) | -1.942 | .959 | 4.099 | 1 | .043 | .143 | .022 | .940 |
|  | Use of M-health is superior and more advantageous than current manual practices.(3) | -.487 | .529 | .848 | 1 | .357 | .615 | .218 | 1.732 |
|  | M-Health improves efficiency of hospital’s operations |  |  | 4.067 | 3 | .254 |  |  |  |
|  | M-Health improves efficiency of hospital’s operations (1) | .243 | 1.835 | .017 | 1 | .895 | 1.274 | .035 | 46.493 |
|  | M-Health improves efficiency of hospital’s operations (2) | 1.233 | 1.032 | 1.427 | 1 | .232 | 3.430 | .454 | 25.913 |
|  | M-Health improves efficiency of hospital’s operations (3) | 1.144 | .596 | 3.692 | 1 | .055 | 3.140 | .977 | 10.091 |
|  | M-health will reduce the cost of health care to patients |  |  | 1.206 | 3 | .752 |  |  |  |
|  | M-health will reduce the cost of health care to patients(1) | -.102 | 1.207 | .007 | 1 | .932 | .903 | .085 | 9.620 |
|  | M-health will reduce the cost of health care to patients(2) | .202 | .696 | .084 | 1 | .772 | 1.223 | .312 | 4.790 |
|  | M-health will reduce the cost of health care to patients(3) | .537 | .512 | 1.101 | 1 | .294 | 1.710 | .628 | 4.661 |
|  | M-Health is compatible with current hospital’s health information system and consistent with its values and needs. |  |  | 3.116 | 3 | .374 |  |  |  |
|  | M-Health is compatible with current hospital’s health information system and consistent with its values and needs.(1) | .132 | .810 | .026 | 1 | .871 | 1.141 | .233 | 5.580 |
|  | M-Health is compatible with current hospital’s health information system and consistent with its values and needs.(2) | .063 | .669 | .009 | 1 | .925 | 1.065 | .287 | 3.948 |
|  | M-Health is compatible with current hospital’s health information system and consistent with its values and needs.(3) | -.743 | .514 | 2.087 | 1 | .149 | .476 | .174 | 1.303 |
|  | M-Health can be trusted in terms of its security and confidentiality of patient information |  |  | .920 | 3 | .821 |  |  |  |
|  | M-Health can be trusted in terms of its security and confidentiality of patient information(1) | .602 | 1.011 | .354 | 1 | .552 | 1.825 | .251 | 13.248 |
|  | M-Health can be trusted in terms of its security and confidentiality of patient information(2) | -.007 | .660 | .000 | 1 | .991 | .993 | .272 | 3.617 |
|  | M-Health can be trusted in terms of its security and confidentiality of patient information(3) | -.278 | .529 | .275 | 1 | .600 | .758 | .268 | 2.138 |
|  | M-Health will make many key staff redundant |  |  | .399 | 3 | .940 |  |  |  |
|  | M-Health will make many key staff redundant(1) | .152 | .667 | .052 | 1 | .820 | 1.164 | .315 | 4.301 |
|  | M-Health will make many key staff redundant(2) | .399 | .696 | .328 | 1 | .567 | 1.490 | .381 | 5.832 |
|  | M-Health will make many key staff redundant(3) | .321 | .712 | .203 | 1 | .652 | 1.379 | .341 | 5.572 |
|  | M-Health is difficult to understand, use and integrate in the hospital systems and operations |  |  | 5.352 | 3 | .148 |  |  |  |
|  | M-Health is difficult to understand, use and integrate in the hospital systems and operations(1) | -.377 | 1.079 | .122 | 1 | .727 | .686 | .083 | 5.684 |
|  | M-Health is difficult to understand, use and integrate in the hospital systems and operations(2) | -1.455 | 1.088 | 1.789 | 1 | .181 | .233 | .028 | 1.968 |
|  | M-Health is difficult to understand, use and integrate in the hospital systems and operations(3) | -1.357 | 1.134 | 1.432 | 1 | .231 | .258 | .028 | 2.376 |
|  | Adoption of M-Health innovations requires exorbitant cost in infrastructure and human resources development |  |  | 2.115 | 3 | .549 |  |  |  |
|  | Adoption of M-Health innovations requires exorbitant cost in infrastructure and human resources development(1) | .005 | .707 | .000 | 1 | .995 | 1.005 | .251 | 4.015 |
|  | Adoption of M-Health innovations requires exorbitant cost in infrastructure and human resources development(2) | .788 | .632 | 1.554 | 1 | .213 | 2.200 | .637 | 7.596 |
|  | Adoption of M-Health innovations requires exorbitant cost in infrastructure and human resources development(3) | .581 | .614 | .896 | 1 | .344 | 1.788 | .537 | 5.957 |
|  | M-Health needs to be piloted first in order to demonstrate that it is better than using current manual systems |  |  | .836 | 3 | .841 |  |  |  |
|  | M-Health needs to be piloted first in order to demonstrate that it is better than using current manual systems(1) | .178 | .726 | .060 | 1 | .806 | 1.195 | .288 | 4.955 |
|  | M-Health needs to be piloted first in order to demonstrate that it is better than using current manual systems(2) | -.512 | .711 | .518 | 1 | .472 | .600 | .149 | 2.414 |
|  | M-Health needs to be piloted first in order to demonstrate that it is better than using current manual systems(3) | .078 | .512 | .023 | 1 | .879 | 1.081 | .396 | 2.947 |
|  | M-Health can be piloted without serious negative impact to patients and hospital operations |  |  | 6.736 | 3 | .081 |  |  |  |
|  | M-Health can be piloted without serious negative impact to patients and hospital operations(1) | .451 | 1.043 | .187 | 1 | .666 | 1.569 | .203 | 12.123 |
|  | M-Health can be piloted without serious negative impact to patients and hospital operations(2) | -1.147 | .677 | 2.868 | 1 | .090 | .317 | .084 | 1.198 |
|  | M-Health can be piloted without serious negative impact to patients and hospital operations(3) | .419 | .458 | .835 | 1 | .361 | 1.520 | .619 | 3.734 |
|  | It is more strategic to adopt M-Health when the technology is leased to the hospital |  |  | 3.221 | 3 | .359 |  |  |  |
|  | It is more strategic to adopt M-Health when the technology is leased to the hospital(1) | -.517 | .671 | .592 | 1 | .442 | .597 | .160 | 2.223 |
|  | It is more strategic to adopt M-Health when the technology is leased to the hospital(2) | .505 | .677 | .558 | 1 | .455 | 1.658 | .440 | 6.246 |
|  | It is more strategic to adopt M-Health when the technology is leased to the hospital(3) | .445 | .631 | .496 | 1 | .481 | 1.560 | .453 | 5.379 |
|  | It is more strategic to adopt M-Health when the hospital fully owned the technology |  |  | 2.556 | 3 | .465 |  |  |  |
|  | It is more strategic to adopt M-Health when the hospital fully owned the technology(1) | -.983 | .815 | 1.453 | 1 | .228 | .374 | .076 | 1.850 |
|  | It is more strategic to adopt M-Health when the hospital fully owned the technology(2) | -.920 | .704 | 1.706 | 1 | .192 | .399 | .100 | 1.585 |
|  | It is more strategic to adopt M-Health when the hospital fully owned the technology(3) | -.378 | .498 | .575 | 1 | .448 | .685 | .258 | 1.820 |
|  | Constant | .469 | 1.248 | .141 | 1 | .707 | 1.598 |  |  |
| a. Variable(s) entered on step 1: Use of M-health is superior and more advantageous than current manual practices., M-Health improves efficiency of hospital’s operations , M-health will reduce the cost of health care to patients, M-Health is compatible with current hospital’s health information system and consistent with its values and needs., M-Health can be trusted in terms of its security and confidentiality of patient information, M-Health will make many key staff redundant, M-Health is difficult to understand, use and integrate in the hospital systems and operations, Adoption of M-Health innovations requires exorbitant cost in infrastructure and human resources development, M-Health needs to be piloted first in order to demonstrate that it is better than using current manual systems, M-Health can be piloted without serious negative impact to patients and hospital operations, It is more strategic to adopt M-Health when the technology is leased to the hospital, It is more strategic to adopt M-Health when the hospital fully owned the technology. | | | | | | | | | |

1. *Mobile surveys (surveys by mobile phone) and technological determinants*

LOGISTIC REGRESSION VARIABLES Q20.8

/METHOD=ENTER Q23.1 Q23.2 Q23.3 Q23.4 Q23.5 Q23.6 Q23.7 Q23.8 Q23.9 Q23.10 Q23.11 Q23.12

/CONTRAST (Q23.1)=Indicator

/CONTRAST (Q23.2)=Indicator

/CONTRAST (Q23.3)=Indicator

/CONTRAST (Q23.4)=Indicator

/CONTRAST (Q23.5)=Indicator

/CONTRAST (Q23.6)=Indicator

/CONTRAST (Q23.7)=Indicator

/CONTRAST (Q23.8)=Indicator

/CONTRAST (Q23.9)=Indicator

/CONTRAST (Q23.10)=Indicator

/CONTRAST (Q23.11)=Indicator

/CONTRAST (Q23.12)=Indicator

/SAVE=PGROUP

/PRINT=GOODFIT SUMMARY CI(95)

/CRITERIA=PIN(0.05) POUT(0.10) ITERATE(20) CUT(0.5).

**Logistic Regression**

| **Notes** | | |
| --- | --- | --- |
| Output Created | | 15-JUL-2018 13:34:46 |
| Comments | |  |
| Input | Data | C:\Users\Ocholap\Downloads\m-health data.- 13-JULY 2018.sav |
|  | Active Dataset | DataSet1 |
|  | Filter | <none> |
|  | Weight | <none> |
|  | Split File | <none> |
|  | N of Rows in Working Data File | 211 |
| Missing Value Handling | Definition of Missing | User-defined missing values are treated as missing |
| Syntax | | LOGISTIC REGRESSION VARIABLES Q20.8  /METHOD=ENTER Q23.1 Q23.2 Q23.3 Q23.4 Q23.5 Q23.6 Q23.7 Q23.8 Q23.9 Q23.10 Q23.11 Q23.12  /CONTRAST (Q23.1)=Indicator  /CONTRAST (Q23.2)=Indicator  /CONTRAST (Q23.3)=Indicator  /CONTRAST (Q23.4)=Indicator  /CONTRAST (Q23.5)=Indicator  /CONTRAST (Q23.6)=Indicator  /CONTRAST (Q23.7)=Indicator  /CONTRAST (Q23.8)=Indicator  /CONTRAST (Q23.9)=Indicator  /CONTRAST (Q23.10)=Indicator  /CONTRAST (Q23.11)=Indicator  /CONTRAST (Q23.12)=Indicator  /SAVE=PGROUP  /PRINT=GOODFIT SUMMARY CI(95)  /CRITERIA=PIN(0.05) POUT(0.10) ITERATE(20) CUT(0.5). |
| Resources | Processor Time | 00:00:00.06 |
|  | Elapsed Time | 00:00:00.17 |
| Variables Created or Modified | PGR_22 | Predicted group |

| **Case Processing Summary** | | | |
| --- | --- | --- | --- |
| Unweighted Cases^a^ | | N | Percent |
| Selected Cases | Included in Analysis | 164 | 77.7 |
|  | Missing Cases | 47 | 22.3 |
|  | Total | 211 | 100.0 |
| Unselected Cases | | 0 | .0 |
| Total | | 211 | 100.0 |
| a. If weight is in effect, see classification table for the total number of cases. | | | |

| **Dependent Variable Encoding** | |
| --- | --- |
| Original Value | Internal Value |
| Not adopted | 0 |
| Adopted | 1 |

| **Categorical Variables Codings** | | | | | |
| --- | --- | --- | --- | --- | --- |
|  | | Frequency | Parameter coding | | |
|  |  |  | (1) | (2) | (3) |
| It is more strategic to adopt M-Health when the hospital fully owned the technology | Strongly disgree | 14 | 1.000 | .000 | .000 |
|  | Disagree | 23 | .000 | 1.000 | .000 |
|  | Agree | 62 | .000 | .000 | 1.000 |
|  | Strongly Agree | 65 | .000 | .000 | .000 |
| M-Health improves efficiency of hospital’s operations | Strongly disgree | 6 | 1.000 | .000 | .000 |
|  | Disagree | 8 | .000 | 1.000 | .000 |
|  | Agree | 64 | .000 | .000 | 1.000 |
|  | Strongly Agree | 86 | .000 | .000 | .000 |
| M-health will reduce the cost of health care to patients | Strongly disgree | 8 | 1.000 | .000 | .000 |
|  | Disagree | 21 | .000 | 1.000 | .000 |
|  | Agree | 58 | .000 | .000 | 1.000 |
|  | Strongly Agree | 77 | .000 | .000 | .000 |
| M-Health is compatible with current hospital’s health information system and consistent with its values and needs. | Strongly disgree | 16 | 1.000 | .000 | .000 |
|  | Disagree | 30 | .000 | 1.000 | .000 |
|  | Agree | 64 | .000 | .000 | 1.000 |
|  | Strongly Agree | 54 | .000 | .000 | .000 |
| M-Health can be trusted in terms of its security and confidentiality of patient information | Strongly disgree | 9 | 1.000 | .000 | .000 |
|  | Disagree | 29 | .000 | 1.000 | .000 |
|  | Agree | 82 | .000 | .000 | 1.000 |
|  | Strongly Agree | 44 | .000 | .000 | .000 |
| M-Health will make many key staff redundant | Strongly disgree | 42 | 1.000 | .000 | .000 |
|  | Disagree | 64 | .000 | 1.000 | .000 |
|  | Agree | 39 | .000 | .000 | 1.000 |
|  | Strongly Agree | 19 | .000 | .000 | .000 |
| M-Health is difficult to understand, use and integrate in the hospital systems and operations | Strongly disgree | 50 | 1.000 | .000 | .000 |
|  | Disagree | 77 | .000 | 1.000 | .000 |
|  | Agree | 30 | .000 | .000 | 1.000 |
|  | Strongly Agree | 7 | .000 | .000 | .000 |
| It is more strategic to adopt M-Health when the technology is leased to the hospital | Strongly disgree | 34 | 1.000 | .000 | .000 |
|  | Disagree | 62 | .000 | 1.000 | .000 |
|  | Agree | 42 | .000 | .000 | 1.000 |
|  | Strongly Agree | 26 | .000 | .000 | .000 |
| M-Health can be piloted without serious negative impact to patients and hospital operations | Strongly disgree | 6 | 1.000 | .000 | .000 |
|  | Disagree | 26 | .000 | 1.000 | .000 |
|  | Agree | 85 | .000 | .000 | 1.000 |
|  | Strongly Agree | 47 | .000 | .000 | .000 |
| M-Health needs to be piloted first in order to demonstrate that it is better than using current manual systems | Strongly disgree | 17 | 1.000 | .000 | .000 |
|  | Disagree | 19 | .000 | 1.000 | .000 |
|  | Agree | 59 | .000 | .000 | 1.000 |
|  | Strongly Agree | 69 | .000 | .000 | .000 |
| Adoption of M-Health innovations requires exorbitant cost in infrastructure and human resources development | Strongly disgree | 26 | 1.000 | .000 | .000 |
|  | Disagree | 54 | .000 | 1.000 | .000 |
|  | Agree | 59 | .000 | .000 | 1.000 |
|  | Strongly Agree | 25 | .000 | .000 | .000 |
| Use of M-health is superior and more advantageous than current manual practices. | Strongly disgree | 10 | 1.000 | .000 | .000 |
|  | Disagree | 14 | .000 | 1.000 | .000 |
|  | Agree | 65 | .000 | .000 | 1.000 |
|  | Strongly Agree | 75 | .000 | .000 | .000 |

**Block 0: Beginning Block**

| **Classification Table^a,b^** | | | | | |
| --- | --- | --- | --- | --- | --- |
|  | Observed | | Predicted | | |
|  |  |  | Mobile surveys (surveys by mobile phone) | | Percentage Correct |
|  |  |  | Not adopted | Adopted |  |
| Step 0 | Mobile surveys (surveys by mobile phone) | Not adopted | 127 | 0 | 100.0 |
|  |  | Adopted | 37 | 0 | .0 |
|  | Overall Percentage | |  |  | 77.4 |
| a. Constant is included in the model. | | | | | |
| b. The cut value is .500 | | | | | |

| **Variables in the Equation** | | | | | | | |
| --- | --- | --- | --- | --- | --- | --- | --- |
|  | | B | S.E. | Wald | df | Sig. | Exp(B) |
| Step 0 | Constant | -1.233 | .187 | 43.579 | 1 | .000 | .291 |

| **Variables not in the Equation** | | | | | |
| --- | --- | --- | --- | --- | --- |
|  | | | Score | df | Sig. |
| Step 0 | Variables | Use of M-health is superior and more advantageous than current manual practices. | 1.458 | 3 | .692 |
|  |  | Use of M-health is superior and more advantageous than current manual practices.(1) | .337 | 1 | .561 |
|  |  | Use of M-health is superior and more advantageous than current manual practices.(2) | .011 | 1 | .916 |
|  |  | Use of M-health is superior and more advantageous than current manual practices.(3) | .796 | 1 | .372 |
|  |  | M-Health improves efficiency of hospital’s operations | 1.743 | 3 | .627 |
|  |  | M-Health improves efficiency of hospital’s operations (1) | .124 | 1 | .725 |
|  |  | M-Health improves efficiency of hospital’s operations (2) | 1.074 | 1 | .300 |
|  |  | M-Health improves efficiency of hospital’s operations (3) | .357 | 1 | .550 |
|  |  | M-health will reduce the cost of health care to patients | 4.054 | 3 | .256 |
|  |  | M-health will reduce the cost of health care to patients(1) | 3.624 | 1 | .057 |
|  |  | M-health will reduce the cost of health care to patients(2) | .170 | 1 | .680 |
|  |  | M-health will reduce the cost of health care to patients(3) | .664 | 1 | .415 |
|  |  | M-Health is compatible with current hospital’s health information system and consistent with its values and needs. | .788 | 3 | .852 |
|  |  | M-Health is compatible with current hospital’s health information system and consistent with its values and needs.(1) | .060 | 1 | .806 |
|  |  | M-Health is compatible with current hospital’s health information system and consistent with its values and needs.(2) | .013 | 1 | .911 |
|  |  | M-Health is compatible with current hospital’s health information system and consistent with its values and needs.(3) | .357 | 1 | .550 |
|  |  | M-Health can be trusted in terms of its security and confidentiality of patient information | 3.303 | 3 | .347 |
|  |  | M-Health can be trusted in terms of its security and confidentiality of patient information(1) | .715 | 1 | .398 |
|  |  | M-Health can be trusted in terms of its security and confidentiality of patient information(2) | 2.866 | 1 | .090 |
|  |  | M-Health can be trusted in terms of its security and confidentiality of patient information(3) | .314 | 1 | .575 |
|  |  | M-Health will make many key staff redundant | 5.229 | 3 | .156 |
|  |  | M-Health will make many key staff redundant(1) | 2.276 | 1 | .131 |
|  |  | M-Health will make many key staff redundant(2) | 4.339 | 1 | .037 |
|  |  | M-Health will make many key staff redundant(3) | .008 | 1 | .930 |
|  |  | M-Health is difficult to understand, use and integrate in the hospital systems and operations | .174 | 3 | .982 |
|  |  | M-Health is difficult to understand, use and integrate in the hospital systems and operations(1) | .013 | 1 | .909 |
|  |  | M-Health is difficult to understand, use and integrate in the hospital systems and operations(2) | .019 | 1 | .889 |
|  |  | M-Health is difficult to understand, use and integrate in the hospital systems and operations(3) | .013 | 1 | .911 |
|  |  | Adoption of M-Health innovations requires exorbitant cost in infrastructure and human resources development | 2.909 | 3 | .406 |
|  |  | Adoption of M-Health innovations requires exorbitant cost in infrastructure and human resources development(1) | .911 | 1 | .340 |
|  |  | Adoption of M-Health innovations requires exorbitant cost in infrastructure and human resources development(2) | .522 | 1 | .470 |
|  |  | Adoption of M-Health innovations requires exorbitant cost in infrastructure and human resources development(3) | .809 | 1 | .368 |
|  |  | M-Health needs to be piloted first in order to demonstrate that it is better than using current manual systems | 5.573 | 3 | .134 |
|  |  | M-Health needs to be piloted first in order to demonstrate that it is better than using current manual systems(1) | .262 | 1 | .609 |
|  |  | M-Health needs to be piloted first in order to demonstrate that it is better than using current manual systems(2) | .173 | 1 | .677 |
|  |  | M-Health needs to be piloted first in order to demonstrate that it is better than using current manual systems(3) | 4.274 | 1 | .039 |
|  |  | M-Health can be piloted without serious negative impact to patients and hospital operations | 3.559 | 3 | .313 |
|  |  | M-Health can be piloted without serious negative impact to patients and hospital operations(1) | .124 | 1 | .725 |
|  |  | M-Health can be piloted without serious negative impact to patients and hospital operations(2) | .911 | 1 | .340 |
|  |  | M-Health can be piloted without serious negative impact to patients and hospital operations(3) | .662 | 1 | .416 |
|  |  | It is more strategic to adopt M-Health when the technology is leased to the hospital | .704 | 3 | .872 |
|  |  | It is more strategic to adopt M-Health when the technology is leased to the hospital(1) | .023 | 1 | .879 |
|  |  | It is more strategic to adopt M-Health when the technology is leased to the hospital(2) | .587 | 1 | .444 |
|  |  | It is more strategic to adopt M-Health when the technology is leased to the hospital(3) | .426 | 1 | .514 |
|  |  | It is more strategic to adopt M-Health when the hospital fully owned the technology | 3.934 | 3 | .269 |
|  |  | It is more strategic to adopt M-Health when the hospital fully owned the technology(1) | 2.083 | 1 | .149 |
|  |  | It is more strategic to adopt M-Health when the hospital fully owned the technology(2) | .010 | 1 | .919 |
|  |  | It is more strategic to adopt M-Health when the hospital fully owned the technology(3) | .587 | 1 | .444 |
|  | Overall Statistics | | 32.012 | 36 | .659 |

**Block 1: Method = Enter**

| **Omnibus Tests of Model Coefficients** | | | | |
| --- | --- | --- | --- | --- |
|  | | Chi-square | df | Sig. |
| Step 1 | Step | 33.055 | 36 | .609 |
|  | Block | 33.055 | 36 | .609 |
|  | Model | 33.055 | 36 | .609 |

| **Model Summary** | | | |
| --- | --- | --- | --- |
| Step | -2 Log likelihood | Cox & Snell R Square | Nagelkerke R Square |
| 1 | 142.070^a^ | .183 | .278 |
| a. Estimation terminated at iteration number 6 because parameter estimates changed by less than .001. | | | |

| **Hosmer and Lemeshow Test** | | | |
| --- | --- | --- | --- |
| Step | Chi-square | df | Sig. |
| 1 | 8.153 | 8 | .419 |

| **Contingency Table for Hosmer and Lemeshow Test** | | | | | | |
| --- | --- | --- | --- | --- | --- | --- |
|  | | Mobile surveys (surveys by mobile phone) = Not adopted | | Mobile surveys (surveys by mobile phone) = Adopted | | Total |
|  |  | Observed | Expected | Observed | Expected |  |
| Step 1 | 1 | 16 | 15.578 | 0 | .422 | 16 |
|  | 2 | 15 | 14.968 | 1 | 1.032 | 16 |
|  | 3 | 13 | 14.567 | 3 | 1.433 | 16 |
|  | 4 | 15 | 14.199 | 1 | 1.801 | 16 |
|  | 5 | 14 | 13.681 | 2 | 2.319 | 16 |
|  | 6 | 15 | 13.225 | 1 | 2.775 | 16 |
|  | 7 | 10 | 12.548 | 6 | 3.452 | 16 |
|  | 8 | 11 | 11.256 | 5 | 4.744 | 16 |
|  | 9 | 12 | 9.790 | 4 | 6.210 | 16 |
|  | 10 | 6 | 7.187 | 14 | 12.813 | 20 |

| **Classification Table^a^** | | | | | |
| --- | --- | --- | --- | --- | --- |
|  | Observed | | Predicted | | |
|  |  |  | Mobile surveys (surveys by mobile phone) | | Percentage Correct |
|  |  |  | Not adopted | Adopted |  |
| Step 1 | Mobile surveys (surveys by mobile phone) | Not adopted | 121 | 6 | 95.3 |
|  |  | Adopted | 27 | 10 | 27.0 |
|  | Overall Percentage | |  |  | 79.9 |
| a. The cut value is .500 | | | | | |

| **Variables in the Equation** | | | | | | | | | |
| --- | --- | --- | --- | --- | --- | --- | --- | --- | --- |
|  | | B | S.E. | Wald | df | Sig. | Exp(B) | 95% C.I.for EXP(B) | |
|  |  |  |  |  |  |  |  | Lower | Upper |
| Step 1^a^ | Use of M-health is superior and more advantageous than current manual practices. |  |  | 1.962 | 3 | .580 |  |  |  |
|  | Use of M-health is superior and more advantageous than current manual practices.(1) | 1.001 | 1.239 | .653 | 1 | .419 | 2.722 | .240 | 30.877 |
|  | Use of M-health is superior and more advantageous than current manual practices.(2) | .616 | 1.145 | .289 | 1 | .591 | 1.852 | .196 | 17.480 |
|  | Use of M-health is superior and more advantageous than current manual practices.(3) | .833 | .657 | 1.610 | 1 | .204 | 2.301 | .635 | 8.336 |
|  | M-Health improves efficiency of hospital’s operations |  |  | 1.362 | 3 | .714 |  |  |  |
|  | M-Health improves efficiency of hospital’s operations (1) | -2.621 | 2.570 | 1.041 | 1 | .308 | .073 | .000 | 11.191 |
|  | M-Health improves efficiency of hospital’s operations (2) | .404 | 1.313 | .095 | 1 | .758 | 1.497 | .114 | 19.629 |
|  | M-Health improves efficiency of hospital’s operations (3) | .210 | .755 | .078 | 1 | .781 | 1.234 | .281 | 5.421 |
|  | M-health will reduce the cost of health care to patients |  |  | 3.446 | 3 | .328 |  |  |  |
|  | M-health will reduce the cost of health care to patients(1) | 2.409 | 1.548 | 2.423 | 1 | .120 | 11.124 | .536 | 231.093 |
|  | M-health will reduce the cost of health care to patients(2) | -.366 | .962 | .145 | 1 | .703 | .693 | .105 | 4.569 |
|  | M-health will reduce the cost of health care to patients(3) | -.472 | .688 | .471 | 1 | .492 | .624 | .162 | 2.400 |
|  | M-Health is compatible with current hospital’s health information system and consistent with its values and needs. |  |  | 1.625 | 3 | .654 |  |  |  |
|  | M-Health is compatible with current hospital’s health information system and consistent with its values and needs.(1) | .501 | .999 | .251 | 1 | .616 | 1.650 | .233 | 11.704 |
|  | M-Health is compatible with current hospital’s health information system and consistent with its values and needs.(2) | .703 | .880 | .639 | 1 | .424 | 2.021 | .360 | 11.338 |
|  | M-Health is compatible with current hospital’s health information system and consistent with its values and needs.(3) | .849 | .671 | 1.602 | 1 | .206 | 2.338 | .627 | 8.712 |
|  | M-Health can be trusted in terms of its security and confidentiality of patient information |  |  | 1.363 | 3 | .714 |  |  |  |
|  | M-Health can be trusted in terms of its security and confidentiality of patient information(1) | -.823 | 1.670 | .243 | 1 | .622 | .439 | .017 | 11.596 |
|  | M-Health can be trusted in terms of its security and confidentiality of patient information(2) | .400 | .808 | .246 | 1 | .620 | 1.492 | .306 | 7.272 |
|  | M-Health can be trusted in terms of its security and confidentiality of patient information(3) | -.266 | .685 | .151 | 1 | .697 | .766 | .200 | 2.931 |
|  | M-Health will make many key staff redundant |  |  | 1.862 | 3 | .601 |  |  |  |
|  | M-Health will make many key staff redundant(1) | .055 | .847 | .004 | 1 | .949 | 1.056 | .201 | 5.550 |
|  | M-Health will make many key staff redundant(2) | -.800 | .889 | .809 | 1 | .368 | .449 | .079 | 2.567 |
|  | M-Health will make many key staff redundant(3) | -.303 | .865 | .123 | 1 | .726 | .739 | .136 | 4.023 |
|  | M-Health is difficult to understand, use and integrate in the hospital systems and operations |  |  | 1.858 | 3 | .602 |  |  |  |
|  | M-Health is difficult to understand, use and integrate in the hospital systems and operations(1) | -1.450 | 1.323 | 1.201 | 1 | .273 | .235 | .018 | 3.137 |
|  | M-Health is difficult to understand, use and integrate in the hospital systems and operations(2) | -1.667 | 1.338 | 1.554 | 1 | .213 | .189 | .014 | 2.597 |
|  | M-Health is difficult to understand, use and integrate in the hospital systems and operations(3) | -1.878 | 1.408 | 1.779 | 1 | .182 | .153 | .010 | 2.415 |
|  | Adoption of M-Health innovations requires exorbitant cost in infrastructure and human resources development |  |  | 1.320 | 3 | .724 |  |  |  |
|  | Adoption of M-Health innovations requires exorbitant cost in infrastructure and human resources development(1) | -.962 | .874 | 1.213 | 1 | .271 | .382 | .069 | 2.117 |
|  | Adoption of M-Health innovations requires exorbitant cost in infrastructure and human resources development(2) | -.354 | .746 | .225 | 1 | .635 | .702 | .163 | 3.028 |
|  | Adoption of M-Health innovations requires exorbitant cost in infrastructure and human resources development(3) | -.488 | .715 | .466 | 1 | .495 | .614 | .151 | 2.493 |
|  | M-Health needs to be piloted first in order to demonstrate that it is better than using current manual systems |  |  | 2.207 | 3 | .530 |  |  |  |
|  | M-Health needs to be piloted first in order to demonstrate that it is better than using current manual systems(1) | -.128 | .970 | .017 | 1 | .895 | .880 | .131 | 5.889 |
|  | M-Health needs to be piloted first in order to demonstrate that it is better than using current manual systems(2) | .502 | .855 | .345 | 1 | .557 | 1.652 | .309 | 8.830 |
|  | M-Health needs to be piloted first in order to demonstrate that it is better than using current manual systems(3) | -.744 | .668 | 1.240 | 1 | .266 | .475 | .128 | 1.760 |
|  | M-Health can be piloted without serious negative impact to patients and hospital operations |  |  | 3.207 | 3 | .361 |  |  |  |
|  | M-Health can be piloted without serious negative impact to patients and hospital operations(1) | -.319 | 1.322 | .058 | 1 | .809 | .727 | .054 | 9.700 |
|  | M-Health can be piloted without serious negative impact to patients and hospital operations(2) | -1.329 | .836 | 2.526 | 1 | .112 | .265 | .051 | 1.363 |
|  | M-Health can be piloted without serious negative impact to patients and hospital operations(3) | -.869 | .598 | 2.113 | 1 | .146 | .420 | .130 | 1.353 |
|  | It is more strategic to adopt M-Health when the technology is leased to the hospital |  |  | .150 | 3 | .985 |  |  |  |
|  | It is more strategic to adopt M-Health when the technology is leased to the hospital(1) | .175 | .849 | .043 | 1 | .836 | 1.192 | .226 | 6.289 |
|  | It is more strategic to adopt M-Health when the technology is leased to the hospital(2) | .229 | .827 | .077 | 1 | .782 | 1.257 | .248 | 6.363 |
|  | It is more strategic to adopt M-Health when the technology is leased to the hospital(3) | .301 | .787 | .147 | 1 | .702 | 1.352 | .289 | 6.318 |
|  | It is more strategic to adopt M-Health when the hospital fully owned the technology |  |  | 2.400 | 3 | .494 |  |  |  |
|  | It is more strategic to adopt M-Health when the hospital fully owned the technology(1) | -2.099 | 1.379 | 2.315 | 1 | .128 | .123 | .008 | 1.830 |
|  | It is more strategic to adopt M-Health when the hospital fully owned the technology(2) | -.281 | .903 | .097 | 1 | .756 | .755 | .129 | 4.435 |
|  | It is more strategic to adopt M-Health when the hospital fully owned the technology(3) | -.344 | .655 | .277 | 1 | .599 | .709 | .196 | 2.558 |
|  | Constant | 1.118 | 1.510 | .548 | 1 | .459 | 3.059 |  |  |
| a. Variable(s) entered on step 1: Use of M-health is superior and more advantageous than current manual practices., M-Health improves efficiency of hospital’s operations , M-health will reduce the cost of health care to patients, M-Health is compatible with current hospital’s health information system and consistent with its values and needs., M-Health can be trusted in terms of its security and confidentiality of patient information, M-Health will make many key staff redundant, M-Health is difficult to understand, use and integrate in the hospital systems and operations, Adoption of M-Health innovations requires exorbitant cost in infrastructure and human resources development, M-Health needs to be piloted first in order to demonstrate that it is better than using current manual systems, M-Health can be piloted without serious negative impact to patients and hospital operations, It is more strategic to adopt M-Health when the technology is leased to the hospital, It is more strategic to adopt M-Health when the hospital fully owned the technology. | | | | | | | | | |

1. *Surveillance and technological determinants*

LOGISTIC REGRESSION VARIABLES Q20.9

/METHOD=ENTER Q23.1 Q23.2 Q23.3 Q23.4 Q23.5 Q23.6 Q23.7 Q23.8 Q23.9 Q23.10 Q23.11 Q23.12

/CONTRAST (Q23.1)=Indicator

/CONTRAST (Q23.2)=Indicator

/CONTRAST (Q23.3)=Indicator

/CONTRAST (Q23.4)=Indicator

/CONTRAST (Q23.5)=Indicator

/CONTRAST (Q23.6)=Indicator

/CONTRAST (Q23.7)=Indicator

/CONTRAST (Q23.8)=Indicator

/CONTRAST (Q23.9)=Indicator

/CONTRAST (Q23.10)=Indicator

/CONTRAST (Q23.11)=Indicator

/CONTRAST (Q23.12)=Indicator

/SAVE=PGROUP

/PRINT=GOODFIT SUMMARY CI(95)

/CRITERIA=PIN(0.05) POUT(0.10) ITERATE(20) CUT(0.5).

**Logistic Regression**

| **Notes** | | |
| --- | --- | --- |
| Output Created | | 15-JUL-2018 13:35:06 |
| Comments | |  |
| Input | Data | C:\Users\Ocholap\Downloads\m-health data.- 13-JULY 2018.sav |
|  | Active Dataset | DataSet1 |
|  | Filter | <none> |
|  | Weight | <none> |
|  | Split File | <none> |
|  | N of Rows in Working Data File | 211 |
| Missing Value Handling | Definition of Missing | User-defined missing values are treated as missing |
| Syntax | | LOGISTIC REGRESSION VARIABLES Q20.9  /METHOD=ENTER Q23.1 Q23.2 Q23.3 Q23.4 Q23.5 Q23.6 Q23.7 Q23.8 Q23.9 Q23.10 Q23.11 Q23.12  /CONTRAST (Q23.1)=Indicator  /CONTRAST (Q23.2)=Indicator  /CONTRAST (Q23.3)=Indicator  /CONTRAST (Q23.4)=Indicator  /CONTRAST (Q23.5)=Indicator  /CONTRAST (Q23.6)=Indicator  /CONTRAST (Q23.7)=Indicator  /CONTRAST (Q23.8)=Indicator  /CONTRAST (Q23.9)=Indicator  /CONTRAST (Q23.10)=Indicator  /CONTRAST (Q23.11)=Indicator  /CONTRAST (Q23.12)=Indicator  /SAVE=PGROUP  /PRINT=GOODFIT SUMMARY CI(95)  /CRITERIA=PIN(0.05) POUT(0.10) ITERATE(20) CUT(0.5). |
| Resources | Processor Time | 00:00:00.05 |
|  | Elapsed Time | 00:00:00.06 |
| Variables Created or Modified | PGR_23 | Predicted group |

| **Case Processing Summary** | | | |
| --- | --- | --- | --- |
| Unweighted Cases^a^ | | N | Percent |
| Selected Cases | Included in Analysis | 160 | 75.8 |
|  | Missing Cases | 51 | 24.2 |
|  | Total | 211 | 100.0 |
| Unselected Cases | | 0 | .0 |
| Total | | 211 | 100.0 |
| a. If weight is in effect, see classification table for the total number of cases. | | | |

| **Dependent Variable Encoding** | |
| --- | --- |
| Original Value | Internal Value |
| Not adopted | 0 |
| Adopted | 1 |

| **Categorical Variables Codings** | | | | | |
| --- | --- | --- | --- | --- | --- |
|  | | Frequency | Parameter coding | | |
|  |  |  | (1) | (2) | (3) |
| It is more strategic to adopt M-Health when the hospital fully owned the technology | Strongly disgree | 15 | 1.000 | .000 | .000 |
|  | Disagree | 23 | .000 | 1.000 | .000 |
|  | Agree | 59 | .000 | .000 | 1.000 |
|  | Strongly Agree | 63 | .000 | .000 | .000 |
| M-Health improves efficiency of hospital’s operations | Strongly disgree | 6 | 1.000 | .000 | .000 |
|  | Disagree | 9 | .000 | 1.000 | .000 |
|  | Agree | 57 | .000 | .000 | 1.000 |
|  | Strongly Agree | 88 | .000 | .000 | .000 |
| M-health will reduce the cost of health care to patients | Strongly disgree | 8 | 1.000 | .000 | .000 |
|  | Disagree | 21 | .000 | 1.000 | .000 |
|  | Agree | 55 | .000 | .000 | 1.000 |
|  | Strongly Agree | 76 | .000 | .000 | .000 |
| M-Health is compatible with current hospital’s health information system and consistent with its values and needs. | Strongly disgree | 15 | 1.000 | .000 | .000 |
|  | Disagree | 29 | .000 | 1.000 | .000 |
|  | Agree | 63 | .000 | .000 | 1.000 |
|  | Strongly Agree | 53 | .000 | .000 | .000 |
| M-Health can be trusted in terms of its security and confidentiality of patient information | Strongly disgree | 9 | 1.000 | .000 | .000 |
|  | Disagree | 26 | .000 | 1.000 | .000 |
|  | Agree | 84 | .000 | .000 | 1.000 |
|  | Strongly Agree | 41 | .000 | .000 | .000 |
| M-Health will make many key staff redundant | Strongly disgree | 43 | 1.000 | .000 | .000 |
|  | Disagree | 62 | .000 | 1.000 | .000 |
|  | Agree | 38 | .000 | .000 | 1.000 |
|  | Strongly Agree | 17 | .000 | .000 | .000 |
| M-Health is difficult to understand, use and integrate in the hospital systems and operations | Strongly disgree | 51 | 1.000 | .000 | .000 |
|  | Disagree | 72 | .000 | 1.000 | .000 |
|  | Agree | 30 | .000 | .000 | 1.000 |
|  | Strongly Agree | 7 | .000 | .000 | .000 |
| It is more strategic to adopt M-Health when the technology is leased to the hospital | Strongly disgree | 32 | 1.000 | .000 | .000 |
|  | Disagree | 63 | .000 | 1.000 | .000 |
|  | Agree | 40 | .000 | .000 | 1.000 |
|  | Strongly Agree | 25 | .000 | .000 | .000 |
| M-Health can be piloted without serious negative impact to patients and hospital operations | Strongly disgree | 6 | 1.000 | .000 | .000 |
|  | Disagree | 23 | .000 | 1.000 | .000 |
|  | Agree | 83 | .000 | .000 | 1.000 |
|  | Strongly Agree | 48 | .000 | .000 | .000 |
| M-Health needs to be piloted first in order to demonstrate that it is better than using current manual systems | Strongly disgree | 17 | 1.000 | .000 | .000 |
|  | Disagree | 19 | .000 | 1.000 | .000 |
|  | Agree | 55 | .000 | .000 | 1.000 |
|  | Strongly Agree | 69 | .000 | .000 | .000 |
| Adoption of M-Health innovations requires exorbitant cost in infrastructure and human resources development | Strongly disgree | 23 | 1.000 | .000 | .000 |
|  | Disagree | 55 | .000 | 1.000 | .000 |
|  | Agree | 58 | .000 | .000 | 1.000 |
|  | Strongly Agree | 24 | .000 | .000 | .000 |
| Use of M-health is superior and more advantageous than current manual practices. | Strongly disgree | 10 | 1.000 | .000 | .000 |
|  | Disagree | 13 | .000 | 1.000 | .000 |
|  | Agree | 61 | .000 | .000 | 1.000 |
|  | Strongly Agree | 76 | .000 | .000 | .000 |

**Block 0: Beginning Block**

| **Classification Table^a,b^** | | | | | |
| --- | --- | --- | --- | --- | --- |
|  | Observed | | Predicted | | |
|  |  |  | Surveillance | | Percentage Correct |
|  |  |  | Not adopted | Adopted |  |
| Step 0 | Surveillance | Not adopted | 102 | 0 | 100.0 |
|  |  | Adopted | 58 | 0 | .0 |
|  | Overall Percentage | |  |  | 63.7 |
| a. Constant is included in the model. | | | | | |
| b. The cut value is .500 | | | | | |

| **Variables in the Equation** | | | | | | | |
| --- | --- | --- | --- | --- | --- | --- | --- |
|  | | B | S.E. | Wald | df | Sig. | Exp(B) |
| Step 0 | Constant | -.565 | .164 | 11.784 | 1 | .001 | .569 |

| **Variables not in the Equation** | | | | | |
| --- | --- | --- | --- | --- | --- |
|  | | | Score | df | Sig. |
| Step 0 | Variables | Use of M-health is superior and more advantageous than current manual practices. | 3.516 | 3 | .319 |
|  |  | Use of M-health is superior and more advantageous than current manual practices.(1) | 2.604 | 1 | .107 |
|  |  | Use of M-health is superior and more advantageous than current manual practices.(2) | 1.063 | 1 | .303 |
|  |  | Use of M-health is superior and more advantageous than current manual practices.(3) | .142 | 1 | .706 |
|  |  | M-Health improves efficiency of hospital’s operations | .482 | 3 | .923 |
|  |  | M-Health improves efficiency of hospital’s operations (1) | .023 | 1 | .880 |
|  |  | M-Health improves efficiency of hospital’s operations (2) | .035 | 1 | .851 |
|  |  | M-Health improves efficiency of hospital’s operations (3) | .326 | 1 | .568 |
|  |  | M-health will reduce the cost of health care to patients | .749 | 3 | .862 |
|  |  | M-health will reduce the cost of health care to patients(1) | .689 | 1 | .407 |
|  |  | M-health will reduce the cost of health care to patients(2) | .089 | 1 | .765 |
|  |  | M-health will reduce the cost of health care to patients(3) | .000 | 1 | .983 |
|  |  | M-Health is compatible with current hospital’s health information system and consistent with its values and needs. | 3.115 | 3 | .374 |
|  |  | M-Health is compatible with current hospital’s health information system and consistent with its values and needs.(1) | 2.090 | 1 | .148 |
|  |  | M-Health is compatible with current hospital’s health information system and consistent with its values and needs.(2) | .403 | 1 | .525 |
|  |  | M-Health is compatible with current hospital’s health information system and consistent with its values and needs.(3) | .079 | 1 | .778 |
|  |  | M-Health can be trusted in terms of its security and confidentiality of patient information | 2.965 | 3 | .397 |
|  |  | M-Health can be trusted in terms of its security and confidentiality of patient information(1) | .812 | 1 | .368 |
|  |  | M-Health can be trusted in terms of its security and confidentiality of patient information(2) | 1.318 | 1 | .251 |
|  |  | M-Health can be trusted in terms of its security and confidentiality of patient information(3) | 1.291 | 1 | .256 |
|  |  | M-Health will make many key staff redundant | .386 | 3 | .943 |
|  |  | M-Health will make many key staff redundant(1) | .023 | 1 | .878 |
|  |  | M-Health will make many key staff redundant(2) | .031 | 1 | .859 |
|  |  | M-Health will make many key staff redundant(3) | .008 | 1 | .931 |
|  |  | M-Health is difficult to understand, use and integrate in the hospital systems and operations | 4.382 | 3 | .223 |
|  |  | M-Health is difficult to understand, use and integrate in the hospital systems and operations(1) | 2.536 | 1 | .111 |
|  |  | M-Health is difficult to understand, use and integrate in the hospital systems and operations(2) | 1.837 | 1 | .175 |
|  |  | M-Health is difficult to understand, use and integrate in the hospital systems and operations(3) | .225 | 1 | .635 |
|  |  | Adoption of M-Health innovations requires exorbitant cost in infrastructure and human resources development | 5.472 | 3 | .140 |
|  |  | Adoption of M-Health innovations requires exorbitant cost in infrastructure and human resources development(1) | 4.134 | 1 | .042 |
|  |  | Adoption of M-Health innovations requires exorbitant cost in infrastructure and human resources development(2) | .105 | 1 | .745 |
|  |  | Adoption of M-Health innovations requires exorbitant cost in infrastructure and human resources development(3) | 2.897 | 1 | .089 |
|  |  | M-Health needs to be piloted first in order to demonstrate that it is better than using current manual systems | 7.074 | 3 | .070 |
|  |  | M-Health needs to be piloted first in order to demonstrate that it is better than using current manual systems(1) | .962 | 1 | .327 |
|  |  | M-Health needs to be piloted first in order to demonstrate that it is better than using current manual systems(2) | .204 | 1 | .652 |
|  |  | M-Health needs to be piloted first in order to demonstrate that it is better than using current manual systems(3) | 5.770 | 1 | .016 |
|  |  | M-Health can be piloted without serious negative impact to patients and hospital operations | 8.454 | 3 | .038 |
|  |  | M-Health can be piloted without serious negative impact to patients and hospital operations(1) | 1.035 | 1 | .309 |
|  |  | M-Health can be piloted without serious negative impact to patients and hospital operations(2) | 6.260 | 1 | .012 |
|  |  | M-Health can be piloted without serious negative impact to patients and hospital operations(3) | .396 | 1 | .529 |
|  |  | It is more strategic to adopt M-Health when the technology is leased to the hospital | 1.220 | 3 | .748 |
|  |  | It is more strategic to adopt M-Health when the technology is leased to the hospital(1) | .433 | 1 | .511 |
|  |  | It is more strategic to adopt M-Health when the technology is leased to the hospital(2) | .003 | 1 | .956 |
|  |  | It is more strategic to adopt M-Health when the technology is leased to the hospital(3) | .902 | 1 | .342 |
|  |  | It is more strategic to adopt M-Health when the hospital fully owned the technology | 8.790 | 3 | .032 |
|  |  | It is more strategic to adopt M-Health when the hospital fully owned the technology(1) | 6.268 | 1 | .012 |
|  |  | It is more strategic to adopt M-Health when the hospital fully owned the technology(2) | .096 | 1 | .756 |
|  |  | It is more strategic to adopt M-Health when the hospital fully owned the technology(3) | .662 | 1 | .416 |
|  | Overall Statistics | | 52.322 | 36 | .039 |

**Block 1: Method = Enter**

| **Omnibus Tests of Model Coefficients** | | | | |
| --- | --- | --- | --- | --- |
|  | | Chi-square | df | Sig. |
| Step 1 | Step | 64.947 | 36 | .002 |
|  | Block | 64.947 | 36 | .002 |
|  | Model | 64.947 | 36 | .002 |

| **Model Summary** | | | |
| --- | --- | --- | --- |
| Step | -2 Log likelihood | Cox & Snell R Square | Nagelkerke R Square |
| 1 | 144.602^a^ | .334 | .457 |
| a. Estimation terminated at iteration number 6 because parameter estimates changed by less than .001. | | | |

| **Hosmer and Lemeshow Test** | | | |
| --- | --- | --- | --- |
| Step | Chi-square | df | Sig. |
| 1 | 9.773 | 8 | .281 |

| **Contingency Table for Hosmer and Lemeshow Test** | | | | | | |
| --- | --- | --- | --- | --- | --- | --- |
|  | | Surveillance = Not adopted | | Surveillance = Adopted | | Total |
|  |  | Observed | Expected | Observed | Expected |  |
| Step 1 | 1 | 15 | 15.784 | 1 | .216 | 16 |
|  | 2 | 15 | 14.232 | 0 | .768 | 15 |
|  | 3 | 13 | 15.028 | 4 | 1.972 | 17 |
|  | 4 | 14 | 13.153 | 2 | 2.847 | 16 |
|  | 5 | 13 | 12.724 | 4 | 4.276 | 17 |
|  | 6 | 12 | 10.635 | 4 | 5.365 | 16 |
|  | 7 | 10 | 8.767 | 6 | 7.233 | 16 |
|  | 8 | 5 | 6.306 | 11 | 9.694 | 16 |
|  | 9 | 5 | 3.907 | 11 | 12.093 | 16 |
|  | 10 | 0 | 1.464 | 15 | 13.536 | 15 |

| **Classification Table^a^** | | | | | |
| --- | --- | --- | --- | --- | --- |
|  | Observed | | Predicted | | |
|  |  |  | Surveillance | | Percentage Correct |
|  |  |  | Not adopted | Adopted |  |
| Step 1 | Surveillance | Not adopted | 90 | 12 | 88.2 |
|  |  | Adopted | 20 | 38 | 65.5 |
|  | Overall Percentage | |  |  | 80.0 |
| a. The cut value is .500 | | | | | |

| **Variables in the Equation** | | | | | | | | | |
| --- | --- | --- | --- | --- | --- | --- | --- | --- | --- |
|  | | B | S.E. | Wald | df | Sig. | Exp(B) | 95% C.I.for EXP(B) | |
|  |  |  |  |  |  |  |  | Lower | Upper |
| Step 1^a^ | Use of M-health is superior and more advantageous than current manual practices. |  |  | 5.110 | 3 | .164 |  |  |  |
|  | Use of M-health is superior and more advantageous than current manual practices.(1) | 2.583 | 1.526 | 2.865 | 1 | .091 | 13.235 | .665 | 263.420 |
|  | Use of M-health is superior and more advantageous than current manual practices.(2) | -1.578 | 1.114 | 2.006 | 1 | .157 | .206 | .023 | 1.833 |
|  | Use of M-health is superior and more advantageous than current manual practices.(3) | .222 | .599 | .137 | 1 | .711 | 1.248 | .386 | 4.040 |
|  | M-Health improves efficiency of hospital’s operations |  |  | .831 | 3 | .842 |  |  |  |
|  | M-Health improves efficiency of hospital’s operations (1) | -2.020 | 2.343 | .744 | 1 | .388 | .133 | .001 | 13.079 |
|  | M-Health improves efficiency of hospital’s operations (2) | -.426 | 1.176 | .131 | 1 | .717 | .653 | .065 | 6.541 |
|  | M-Health improves efficiency of hospital’s operations (3) | -.329 | .696 | .224 | 1 | .636 | .720 | .184 | 2.814 |
|  | M-health will reduce the cost of health care to patients |  |  | .832 | 3 | .842 |  |  |  |
|  | M-health will reduce the cost of health care to patients(1) | .732 | 1.302 | .316 | 1 | .574 | 2.079 | .162 | 26.689 |
|  | M-health will reduce the cost of health care to patients(2) | .760 | .930 | .668 | 1 | .414 | 2.138 | .346 | 13.223 |
|  | M-health will reduce the cost of health care to patients(3) | .377 | .651 | .337 | 1 | .562 | 1.459 | .407 | 5.222 |
|  | M-Health is compatible with current hospital’s health information system and consistent with its values and needs. |  |  | 3.291 | 3 | .349 |  |  |  |
|  | M-Health is compatible with current hospital’s health information system and consistent with its values and needs.(1) | 1.820 | 1.017 | 3.200 | 1 | .074 | 6.172 | .840 | 45.334 |
|  | M-Health is compatible with current hospital’s health information system and consistent with its values and needs.(2) | .801 | .786 | 1.038 | 1 | .308 | 2.228 | .477 | 10.401 |
|  | M-Health is compatible with current hospital’s health information system and consistent with its values and needs.(3) | .544 | .621 | .767 | 1 | .381 | 1.723 | .510 | 5.820 |
|  | M-Health can be trusted in terms of its security and confidentiality of patient information |  |  | 5.906 | 3 | .116 |  |  |  |
|  | M-Health can be trusted in terms of its security and confidentiality of patient information(1) | -1.444 | 1.678 | .741 | 1 | .389 | .236 | .009 | 6.325 |
|  | M-Health can be trusted in terms of its security and confidentiality of patient information(2) | .282 | .778 | .131 | 1 | .717 | 1.326 | .288 | 6.096 |
|  | M-Health can be trusted in terms of its security and confidentiality of patient information(3) | -1.119 | .654 | 2.931 | 1 | .087 | .327 | .091 | 1.176 |
|  | M-Health will make many key staff redundant |  |  | 3.264 | 3 | .353 |  |  |  |
|  | M-Health will make many key staff redundant(1) | 1.375 | .934 | 2.169 | 1 | .141 | 3.955 | .635 | 24.651 |
|  | M-Health will make many key staff redundant(2) | 1.204 | .982 | 1.504 | 1 | .220 | 3.334 | .487 | 22.839 |
|  | M-Health will make many key staff redundant(3) | 1.749 | .998 | 3.072 | 1 | .080 | 5.748 | .813 | 40.629 |
|  | M-Health is difficult to understand, use and integrate in the hospital systems and operations |  |  | 8.004 | 3 | .046 |  |  |  |
|  | M-Health is difficult to understand, use and integrate in the hospital systems and operations(1) | 2.213 | 1.459 | 2.302 | 1 | .129 | 9.144 | .524 | 159.492 |
|  | M-Health is difficult to understand, use and integrate in the hospital systems and operations(2) | .518 | 1.427 | .132 | 1 | .717 | 1.679 | .102 | 27.549 |
|  | M-Health is difficult to understand, use and integrate in the hospital systems and operations(3) | 1.235 | 1.496 | .682 | 1 | .409 | 3.439 | .183 | 64.493 |
|  | Adoption of M-Health innovations requires exorbitant cost in infrastructure and human resources development |  |  | 9.070 | 3 | .028 |  |  |  |
|  | Adoption of M-Health innovations requires exorbitant cost in infrastructure and human resources development(1) | -1.785 | .980 | 3.319 | 1 | .068 | .168 | .025 | 1.145 |
|  | Adoption of M-Health innovations requires exorbitant cost in infrastructure and human resources development(2) | .190 | .726 | .068 | 1 | .794 | 1.209 | .291 | 5.019 |
|  | Adoption of M-Health innovations requires exorbitant cost in infrastructure and human resources development(3) | .975 | .747 | 1.701 | 1 | .192 | 2.650 | .613 | 11.463 |
|  | M-Health needs to be piloted first in order to demonstrate that it is better than using current manual systems |  |  | 4.291 | 3 | .232 |  |  |  |
|  | M-Health needs to be piloted first in order to demonstrate that it is better than using current manual systems(1) | .729 | .901 | .655 | 1 | .418 | 2.074 | .355 | 12.133 |
|  | M-Health needs to be piloted first in order to demonstrate that it is better than using current manual systems(2) | .237 | .829 | .082 | 1 | .775 | 1.268 | .249 | 6.442 |
|  | M-Health needs to be piloted first in order to demonstrate that it is better than using current manual systems(3) | -.911 | .623 | 2.135 | 1 | .144 | .402 | .119 | 1.364 |
|  | M-Health can be piloted without serious negative impact to patients and hospital operations |  |  | 9.908 | 3 | .019 |  |  |  |
|  | M-Health can be piloted without serious negative impact to patients and hospital operations(1) | -1.276 | 1.410 | .819 | 1 | .365 | .279 | .018 | 4.424 |
|  | M-Health can be piloted without serious negative impact to patients and hospital operations(2) | -2.834 | .913 | 9.632 | 1 | .002 | .059 | .010 | .352 |
|  | M-Health can be piloted without serious negative impact to patients and hospital operations(3) | -.734 | .561 | 1.712 | 1 | .191 | .480 | .160 | 1.441 |
|  | It is more strategic to adopt M-Health when the technology is leased to the hospital |  |  | 4.764 | 3 | .190 |  |  |  |
|  | It is more strategic to adopt M-Health when the technology is leased to the hospital(1) | -.477 | .873 | .298 | 1 | .585 | .621 | .112 | 3.435 |
|  | It is more strategic to adopt M-Health when the technology is leased to the hospital(2) | .858 | .800 | 1.151 | 1 | .283 | 2.358 | .492 | 11.309 |
|  | It is more strategic to adopt M-Health when the technology is leased to the hospital(3) | 1.094 | .781 | 1.963 | 1 | .161 | 2.986 | .646 | 13.797 |
|  | It is more strategic to adopt M-Health when the hospital fully owned the technology |  |  | 5.848 | 3 | .119 |  |  |  |
|  | It is more strategic to adopt M-Health when the hospital fully owned the technology(1) | -2.898 | 1.413 | 4.207 | 1 | .040 | .055 | .003 | .879 |
|  | It is more strategic to adopt M-Health when the hospital fully owned the technology(2) | -1.040 | .874 | 1.414 | 1 | .234 | .354 | .064 | 1.961 |
|  | It is more strategic to adopt M-Health when the hospital fully owned the technology(3) | -1.032 | .615 | 2.811 | 1 | .094 | .356 | .107 | 1.191 |
|  | Constant | -2.257 | 1.739 | 1.685 | 1 | .194 | .105 |  |  |
| a. Variable(s) entered on step 1: Use of M-health is superior and more advantageous than current manual practices., M-Health improves efficiency of hospital’s operations , M-health will reduce the cost of health care to patients, M-Health is compatible with current hospital’s health information system and consistent with its values and needs., M-Health can be trusted in terms of its security and confidentiality of patient information, M-Health will make many key staff redundant, M-Health is difficult to understand, use and integrate in the hospital systems and operations, Adoption of M-Health innovations requires exorbitant cost in infrastructure and human resources development, M-Health needs to be piloted first in order to demonstrate that it is better than using current manual systems, M-Health can be piloted without serious negative impact to patients and hospital operations, It is more strategic to adopt M-Health when the technology is leased to the hospital, It is more strategic to adopt M-Health when the hospital fully owned the technology. | | | | | | | | | |

1. *Patient monitoring and technological determinants*

LOGISTIC REGRESSION VARIABLES Q20.10

/METHOD=ENTER Q23.1 Q23.2 Q23.3 Q23.4 Q23.5 Q23.6 Q23.7 Q23.8 Q23.9 Q23.10 Q23.11 Q23.12

/CONTRAST (Q23.1)=Indicator

/CONTRAST (Q23.2)=Indicator

/CONTRAST (Q23.3)=Indicator

/CONTRAST (Q23.4)=Indicator

/CONTRAST (Q23.5)=Indicator

/CONTRAST (Q23.6)=Indicator

/CONTRAST (Q23.7)=Indicator

/CONTRAST (Q23.8)=Indicator

/CONTRAST (Q23.9)=Indicator

/CONTRAST (Q23.10)=Indicator

/CONTRAST (Q23.11)=Indicator

/CONTRAST (Q23.12)=Indicator

/SAVE=PGROUP

/PRINT=GOODFIT SUMMARY CI(95)

/CRITERIA=PIN(0.05) POUT(0.10) ITERATE(20) CUT(0.5).

**Logistic Regression**

| **Notes** | | |
| --- | --- | --- |
| Output Created | | 15-JUL-2018 13:35:25 |
| Comments | |  |
| Input | Data | C:\Users\Ocholap\Downloads\m-health data.- 13-JULY 2018.sav |
|  | Active Dataset | DataSet1 |
|  | Filter | <none> |
|  | Weight | <none> |
|  | Split File | <none> |
|  | N of Rows in Working Data File | 211 |
| Missing Value Handling | Definition of Missing | User-defined missing values are treated as missing |
| Syntax | | LOGISTIC REGRESSION VARIABLES Q20.10  /METHOD=ENTER Q23.1 Q23.2 Q23.3 Q23.4 Q23.5 Q23.6 Q23.7 Q23.8 Q23.9 Q23.10 Q23.11 Q23.12  /CONTRAST (Q23.1)=Indicator  /CONTRAST (Q23.2)=Indicator  /CONTRAST (Q23.3)=Indicator  /CONTRAST (Q23.4)=Indicator  /CONTRAST (Q23.5)=Indicator  /CONTRAST (Q23.6)=Indicator  /CONTRAST (Q23.7)=Indicator  /CONTRAST (Q23.8)=Indicator  /CONTRAST (Q23.9)=Indicator  /CONTRAST (Q23.10)=Indicator  /CONTRAST (Q23.11)=Indicator  /CONTRAST (Q23.12)=Indicator  /SAVE=PGROUP  /PRINT=GOODFIT SUMMARY CI(95)  /CRITERIA=PIN(0.05) POUT(0.10) ITERATE(20) CUT(0.5). |
| Resources | Processor Time | 00:00:00.08 |
|  | Elapsed Time | 00:00:00.09 |
| Variables Created or Modified | PGR_24 | Predicted group |

| **Case Processing Summary** | | | |
| --- | --- | --- | --- |
| Unweighted Cases^a^ | | N | Percent |
| Selected Cases | Included in Analysis | 167 | 79.1 |
|  | Missing Cases | 44 | 20.9 |
|  | Total | 211 | 100.0 |
| Unselected Cases | | 0 | .0 |
| Total | | 211 | 100.0 |
| a. If weight is in effect, see classification table for the total number of cases. | | | |

| **Dependent Variable Encoding** | |
| --- | --- |
| Original Value | Internal Value |
| Not adopted | 0 |
| Adopted | 1 |

| **Categorical Variables Codings** | | | | | |
| --- | --- | --- | --- | --- | --- |
|  | | Frequency | Parameter coding | | |
|  |  |  | (1) | (2) | (3) |
| It is more strategic to adopt M-Health when the hospital fully owned the technology | Strongly disgree | 15 | 1.000 | .000 | .000 |
|  | Disagree | 23 | .000 | 1.000 | .000 |
|  | Agree | 62 | .000 | .000 | 1.000 |
|  | Strongly Agree | 67 | .000 | .000 | .000 |
| M-Health improves efficiency of hospital’s operations | Strongly disgree | 6 | 1.000 | .000 | .000 |
|  | Disagree | 9 | .000 | 1.000 | .000 |
|  | Agree | 64 | .000 | .000 | 1.000 |
|  | Strongly Agree | 88 | .000 | .000 | .000 |
| M-health will reduce the cost of health care to patients | Strongly disgree | 8 | 1.000 | .000 | .000 |
|  | Disagree | 24 | .000 | 1.000 | .000 |
|  | Agree | 58 | .000 | .000 | 1.000 |
|  | Strongly Agree | 77 | .000 | .000 | .000 |
| M-Health is compatible with current hospital’s health information system and consistent with its values and needs. | Strongly disgree | 16 | 1.000 | .000 | .000 |
|  | Disagree | 30 | .000 | 1.000 | .000 |
|  | Agree | 66 | .000 | .000 | 1.000 |
|  | Strongly Agree | 55 | .000 | .000 | .000 |
| M-Health can be trusted in terms of its security and confidentiality of patient information | Strongly disgree | 9 | 1.000 | .000 | .000 |
|  | Disagree | 30 | .000 | 1.000 | .000 |
|  | Agree | 84 | .000 | .000 | 1.000 |
|  | Strongly Agree | 44 | .000 | .000 | .000 |
| M-Health will make many key staff redundant | Strongly disgree | 44 | 1.000 | .000 | .000 |
|  | Disagree | 67 | .000 | 1.000 | .000 |
|  | Agree | 38 | .000 | .000 | 1.000 |
|  | Strongly Agree | 18 | .000 | .000 | .000 |
| M-Health is difficult to understand, use and integrate in the hospital systems and operations | Strongly disgree | 54 | 1.000 | .000 | .000 |
|  | Disagree | 75 | .000 | 1.000 | .000 |
|  | Agree | 31 | .000 | .000 | 1.000 |
|  | Strongly Agree | 7 | .000 | .000 | .000 |
| It is more strategic to adopt M-Health when the technology is leased to the hospital | Strongly disgree | 35 | 1.000 | .000 | .000 |
|  | Disagree | 63 | .000 | 1.000 | .000 |
|  | Agree | 43 | .000 | .000 | 1.000 |
|  | Strongly Agree | 26 | .000 | .000 | .000 |
| M-Health can be piloted without serious negative impact to patients and hospital operations | Strongly disgree | 6 | 1.000 | .000 | .000 |
|  | Disagree | 25 | .000 | 1.000 | .000 |
|  | Agree | 85 | .000 | .000 | 1.000 |
|  | Strongly Agree | 51 | .000 | .000 | .000 |
| M-Health needs to be piloted first in order to demonstrate that it is better than using current manual systems | Strongly disgree | 17 | 1.000 | .000 | .000 |
|  | Disagree | 19 | .000 | 1.000 | .000 |
|  | Agree | 60 | .000 | .000 | 1.000 |
|  | Strongly Agree | 71 | .000 | .000 | .000 |
| Adoption of M-Health innovations requires exorbitant cost in infrastructure and human resources development | Strongly disgree | 26 | 1.000 | .000 | .000 |
|  | Disagree | 55 | .000 | 1.000 | .000 |
|  | Agree | 62 | .000 | .000 | 1.000 |
|  | Strongly Agree | 24 | .000 | .000 | .000 |
| Use of M-health is superior and more advantageous than current manual practices. | Strongly disgree | 10 | 1.000 | .000 | .000 |
|  | Disagree | 14 | .000 | 1.000 | .000 |
|  | Agree | 66 | .000 | .000 | 1.000 |
|  | Strongly Agree | 77 | .000 | .000 | .000 |

**Block 0: Beginning Block**

| **Classification Table^a,b^** | | | | | |
| --- | --- | --- | --- | --- | --- |
|  | Observed | | Predicted | | |
|  |  |  | Patient monitoring | | Percentage Correct |
|  |  |  | Not adopted | Adopted |  |
| Step 0 | Patient monitoring | Not adopted | 0 | 78 | .0 |
|  |  | Adopted | 0 | 89 | 100.0 |
|  | Overall Percentage | |  |  | 53.3 |
| a. Constant is included in the model. | | | | | |
| b. The cut value is .500 | | | | | |

| **Variables in the Equation** | | | | | | | |
| --- | --- | --- | --- | --- | --- | --- | --- |
|  | | B | S.E. | Wald | df | Sig. | Exp(B) |
| Step 0 | Constant | .132 | .155 | .724 | 1 | .395 | 1.141 |

| **Variables not in the Equation** | | | | | |
| --- | --- | --- | --- | --- | --- |
|  | | | Score | df | Sig. |
| Step 0 | Variables | Use of M-health is superior and more advantageous than current manual practices. | 3.605 | 3 | .307 |
|  |  | Use of M-health is superior and more advantageous than current manual practices.(1) | 1.193 | 1 | .275 |
|  |  | Use of M-health is superior and more advantageous than current manual practices.(2) | 1.897 | 1 | .168 |
|  |  | Use of M-health is superior and more advantageous than current manual practices.(3) | .476 | 1 | .490 |
|  |  | M-Health improves efficiency of hospital’s operations | 2.514 | 3 | .473 |
|  |  | M-Health improves efficiency of hospital’s operations (1) | .027 | 1 | .869 |
|  |  | M-Health improves efficiency of hospital’s operations (2) | 1.523 | 1 | .217 |
|  |  | M-Health improves efficiency of hospital’s operations (3) | .452 | 1 | .501 |
|  |  | M-health will reduce the cost of health care to patients | 4.224 | 3 | .238 |
|  |  | M-health will reduce the cost of health care to patients(1) | 1.591 | 1 | .207 |
|  |  | M-health will reduce the cost of health care to patients(2) | 1.522 | 1 | .217 |
|  |  | M-health will reduce the cost of health care to patients(3) | .899 | 1 | .343 |
|  |  | M-Health is compatible with current hospital’s health information system and consistent with its values and needs. | 4.229 | 3 | .238 |
|  |  | M-Health is compatible with current hospital’s health information system and consistent with its values and needs.(1) | .647 | 1 | .421 |
|  |  | M-Health is compatible with current hospital’s health information system and consistent with its values and needs.(2) | 1.457 | 1 | .227 |
|  |  | M-Health is compatible with current hospital’s health information system and consistent with its values and needs.(3) | .139 | 1 | .710 |
|  |  | M-Health can be trusted in terms of its security and confidentiality of patient information | 5.244 | 3 | .155 |
|  |  | M-Health can be trusted in terms of its security and confidentiality of patient information(1) | 3.689 | 1 | .055 |
|  |  | M-Health can be trusted in terms of its security and confidentiality of patient information(2) | .645 | 1 | .422 |
|  |  | M-Health can be trusted in terms of its security and confidentiality of patient information(3) | .146 | 1 | .702 |
|  |  | M-Health will make many key staff redundant | 1.498 | 3 | .683 |
|  |  | M-Health will make many key staff redundant(1) | 1.475 | 1 | .225 |
|  |  | M-Health will make many key staff redundant(2) | .527 | 1 | .468 |
|  |  | M-Health will make many key staff redundant(3) | .077 | 1 | .782 |
|  |  | M-Health is difficult to understand, use and integrate in the hospital systems and operations | 1.461 | 3 | .691 |
|  |  | M-Health is difficult to understand, use and integrate in the hospital systems and operations(1) | .067 | 1 | .796 |
|  |  | M-Health is difficult to understand, use and integrate in the hospital systems and operations(2) | .377 | 1 | .539 |
|  |  | M-Health is difficult to understand, use and integrate in the hospital systems and operations(3) | .348 | 1 | .555 |
|  |  | Adoption of M-Health innovations requires exorbitant cost in infrastructure and human resources development | 1.081 | 3 | .782 |
|  |  | Adoption of M-Health innovations requires exorbitant cost in infrastructure and human resources development(1) | .239 | 1 | .625 |
|  |  | Adoption of M-Health innovations requires exorbitant cost in infrastructure and human resources development(2) | .052 | 1 | .820 |
|  |  | Adoption of M-Health innovations requires exorbitant cost in infrastructure and human resources development(3) | .954 | 1 | .329 |
|  |  | M-Health needs to be piloted first in order to demonstrate that it is better than using current manual systems | .942 | 3 | .815 |
|  |  | M-Health needs to be piloted first in order to demonstrate that it is better than using current manual systems(1) | .233 | 1 | .630 |
|  |  | M-Health needs to be piloted first in order to demonstrate that it is better than using current manual systems(2) | .302 | 1 | .582 |
|  |  | M-Health needs to be piloted first in order to demonstrate that it is better than using current manual systems(3) | .428 | 1 | .513 |
|  |  | M-Health can be piloted without serious negative impact to patients and hospital operations | .289 | 3 | .962 |
|  |  | M-Health can be piloted without serious negative impact to patients and hospital operations(1) | .027 | 1 | .869 |
|  |  | M-Health can be piloted without serious negative impact to patients and hospital operations(2) | .020 | 1 | .888 |
|  |  | M-Health can be piloted without serious negative impact to patients and hospital operations(3) | .278 | 1 | .598 |
|  |  | It is more strategic to adopt M-Health when the technology is leased to the hospital | 1.433 | 3 | .698 |
|  |  | It is more strategic to adopt M-Health when the technology is leased to the hospital(1) | .800 | 1 | .371 |
|  |  | It is more strategic to adopt M-Health when the technology is leased to the hospital(2) | .254 | 1 | .614 |
|  |  | It is more strategic to adopt M-Health when the technology is leased to the hospital(3) | .148 | 1 | .701 |
|  |  | It is more strategic to adopt M-Health when the hospital fully owned the technology | 10.516 | 3 | .015 |
|  |  | It is more strategic to adopt M-Health when the hospital fully owned the technology(1) | .298 | 1 | .585 |
|  |  | It is more strategic to adopt M-Health when the hospital fully owned the technology(2) | 2.150 | 1 | .143 |
|  |  | It is more strategic to adopt M-Health when the hospital fully owned the technology(3) | 5.111 | 1 | .024 |
|  | Overall Statistics | | 32.035 | 36 | .658 |

**Block 1: Method = Enter**

| **Omnibus Tests of Model Coefficients** | | | | |
| --- | --- | --- | --- | --- |
|  | | Chi-square | df | Sig. |
| Step 1 | Step | 36.977 | 36 | .424 |
|  | Block | 36.977 | 36 | .424 |
|  | Model | 36.977 | 36 | .424 |

| **Model Summary** | | | |
| --- | --- | --- | --- |
| Step | -2 Log likelihood | Cox & Snell R Square | Nagelkerke R Square |
| 1 | 193.809^a^ | .199 | .265 |
| a. Estimation terminated at iteration number 5 because parameter estimates changed by less than .001. | | | |

| **Hosmer and Lemeshow Test** | | | |
| --- | --- | --- | --- |
| Step | Chi-square | df | Sig. |
| 1 | 10.058 | 8 | .261 |

| **Contingency Table for Hosmer and Lemeshow Test** | | | | | | |
| --- | --- | --- | --- | --- | --- | --- |
|  | | Patient monitoring = Not adopted | | Patient monitoring = Adopted | | Total |
|  |  | Observed | Expected | Observed | Expected |  |
| Step 1 | 1 | 13 | 14.590 | 4 | 2.410 | 17 |
|  | 2 | 14 | 12.341 | 3 | 4.659 | 17 |
|  | 3 | 11 | 10.726 | 6 | 6.274 | 17 |
|  | 4 | 8 | 9.361 | 9 | 7.639 | 17 |
|  | 5 | 12 | 8.310 | 5 | 8.690 | 17 |
|  | 6 | 9 | 7.163 | 8 | 9.837 | 17 |
|  | 7 | 6 | 5.665 | 11 | 11.335 | 17 |
|  | 8 | 3 | 4.490 | 14 | 12.510 | 17 |
|  | 9 | 1 | 3.802 | 17 | 14.198 | 18 |
|  | 10 | 1 | 1.551 | 12 | 11.449 | 13 |

| **Classification Table^a^** | | | | | |
| --- | --- | --- | --- | --- | --- |
|  | Observed | | Predicted | | |
|  |  |  | Patient monitoring | | Percentage Correct |
|  |  |  | Not adopted | Adopted |  |
| Step 1 | Patient monitoring | Not adopted | 50 | 28 | 64.1 |
|  |  | Adopted | 23 | 66 | 74.2 |
|  | Overall Percentage | |  |  | 69.5 |
| a. The cut value is .500 | | | | | |

| **Variables in the Equation** | | | | | | | | | |
| --- | --- | --- | --- | --- | --- | --- | --- | --- | --- |
|  | | B | S.E. | Wald | df | Sig. | Exp(B) | 95% C.I.for EXP(B) | |
|  |  |  |  |  |  |  |  | Lower | Upper |
| Step 1^a^ | Use of M-health is superior and more advantageous than current manual practices. |  |  | 1.013 | 3 | .798 |  |  |  |
|  | Use of M-health is superior and more advantageous than current manual practices.(1) | 1.024 | 1.315 | .607 | 1 | .436 | 2.785 | .212 | 36.667 |
|  | Use of M-health is superior and more advantageous than current manual practices.(2) | -.178 | .965 | .034 | 1 | .854 | .837 | .126 | 5.544 |
|  | Use of M-health is superior and more advantageous than current manual practices.(3) | -.267 | .535 | .249 | 1 | .618 | .766 | .268 | 2.186 |
|  | M-Health improves efficiency of hospital’s operations |  |  | 2.152 | 3 | .541 |  |  |  |
|  | M-Health improves efficiency of hospital’s operations (1) | -2.412 | 2.375 | 1.031 | 1 | .310 | .090 | .001 | 9.423 |
|  | M-Health improves efficiency of hospital’s operations (2) | -1.414 | 1.167 | 1.470 | 1 | .225 | .243 | .025 | 2.391 |
|  | M-Health improves efficiency of hospital’s operations (3) | -.368 | .597 | .381 | 1 | .537 | .692 | .215 | 2.229 |
|  | M-health will reduce the cost of health care to patients |  |  | 4.073 | 3 | .254 |  |  |  |
|  | M-health will reduce the cost of health care to patients(1) | 3.166 | 1.617 | 3.834 | 1 | .050 | 23.718 | .997 | 564.322 |
|  | M-health will reduce the cost of health care to patients(2) | .292 | .702 | .173 | 1 | .678 | 1.339 | .338 | 5.300 |
|  | M-health will reduce the cost of health care to patients(3) | .429 | .529 | .656 | 1 | .418 | 1.535 | .544 | 4.333 |
|  | M-Health is compatible with current hospital’s health information system and consistent with its values and needs. |  |  | 2.266 | 3 | .519 |  |  |  |
|  | M-Health is compatible with current hospital’s health information system and consistent with its values and needs.(1) | -.366 | .843 | .188 | 1 | .664 | .694 | .133 | 3.619 |
|  | M-Health is compatible with current hospital’s health information system and consistent with its values and needs.(2) | -.891 | .692 | 1.656 | 1 | .198 | .410 | .106 | 1.593 |
|  | M-Health is compatible with current hospital’s health information system and consistent with its values and needs.(3) | -.698 | .541 | 1.665 | 1 | .197 | .497 | .172 | 1.437 |
|  | M-Health can be trusted in terms of its security and confidentiality of patient information |  |  | 4.533 | 3 | .209 |  |  |  |
|  | M-Health can be trusted in terms of its security and confidentiality of patient information(1) | -3.326 | 1.703 | 3.814 | 1 | .051 | .036 | .001 | 1.012 |
|  | M-Health can be trusted in terms of its security and confidentiality of patient information(2) | .043 | .660 | .004 | 1 | .947 | 1.044 | .287 | 3.806 |
|  | M-Health can be trusted in terms of its security and confidentiality of patient information(3) | .270 | .538 | .252 | 1 | .616 | 1.310 | .456 | 3.764 |
|  | M-Health will make many key staff redundant |  |  | 1.789 | 3 | .617 |  |  |  |
|  | M-Health will make many key staff redundant(1) | -.058 | .756 | .006 | 1 | .939 | .944 | .215 | 4.154 |
|  | M-Health will make many key staff redundant(2) | .376 | .764 | .243 | 1 | .622 | 1.457 | .326 | 6.508 |
|  | M-Health will make many key staff redundant(3) | .689 | .775 | .790 | 1 | .374 | 1.992 | .436 | 9.107 |
|  | M-Health is difficult to understand, use and integrate in the hospital systems and operations |  |  | .732 | 3 | .866 |  |  |  |
|  | M-Health is difficult to understand, use and integrate in the hospital systems and operations(1) | -.487 | 1.120 | .190 | 1 | .663 | .614 | .068 | 5.512 |
|  | M-Health is difficult to understand, use and integrate in the hospital systems and operations(2) | -.402 | 1.118 | .129 | 1 | .719 | .669 | .075 | 5.987 |
|  | M-Health is difficult to understand, use and integrate in the hospital systems and operations(3) | -.022 | 1.185 | .000 | 1 | .985 | .978 | .096 | 9.983 |
|  | Adoption of M-Health innovations requires exorbitant cost in infrastructure and human resources development |  |  | 5.018 | 3 | .170 |  |  |  |
|  | Adoption of M-Health innovations requires exorbitant cost in infrastructure and human resources development(1) | .388 | .709 | .299 | 1 | .584 | 1.474 | .367 | 5.916 |
|  | Adoption of M-Health innovations requires exorbitant cost in infrastructure and human resources development(2) | .707 | .638 | 1.226 | 1 | .268 | 2.028 | .580 | 7.088 |
|  | Adoption of M-Health innovations requires exorbitant cost in infrastructure and human resources development(3) | -.367 | .593 | .383 | 1 | .536 | .693 | .217 | 2.214 |
|  | M-Health needs to be piloted first in order to demonstrate that it is better than using current manual systems |  |  | 3.238 | 3 | .356 |  |  |  |
|  | M-Health needs to be piloted first in order to demonstrate that it is better than using current manual systems(1) | .922 | .775 | 1.416 | 1 | .234 | 2.515 | .551 | 11.484 |
|  | M-Health needs to be piloted first in order to demonstrate that it is better than using current manual systems(2) | -.550 | .721 | .581 | 1 | .446 | .577 | .140 | 2.372 |
|  | M-Health needs to be piloted first in order to demonstrate that it is better than using current manual systems(3) | .450 | .511 | .776 | 1 | .378 | 1.569 | .576 | 4.272 |
|  | M-Health can be piloted without serious negative impact to patients and hospital operations |  |  | .941 | 3 | .816 |  |  |  |
|  | M-Health can be piloted without serious negative impact to patients and hospital operations(1) | -.064 | 1.107 | .003 | 1 | .954 | .938 | .107 | 8.208 |
|  | M-Health can be piloted without serious negative impact to patients and hospital operations(2) | .585 | .658 | .789 | 1 | .374 | 1.794 | .494 | 6.516 |
|  | M-Health can be piloted without serious negative impact to patients and hospital operations(3) | .333 | .473 | .495 | 1 | .482 | 1.395 | .552 | 3.522 |
|  | It is more strategic to adopt M-Health when the technology is leased to the hospital |  |  | 1.158 | 3 | .763 |  |  |  |
|  | It is more strategic to adopt M-Health when the technology is leased to the hospital(1) | .642 | .722 | .790 | 1 | .374 | 1.899 | .462 | 7.815 |
|  | It is more strategic to adopt M-Health when the technology is leased to the hospital(2) | .306 | .684 | .201 | 1 | .654 | 1.359 | .356 | 5.189 |
|  | It is more strategic to adopt M-Health when the technology is leased to the hospital(3) | .575 | .644 | .799 | 1 | .371 | 1.778 | .504 | 6.276 |
|  | It is more strategic to adopt M-Health when the hospital fully owned the technology |  |  | 5.705 | 3 | .127 |  |  |  |
|  | It is more strategic to adopt M-Health when the hospital fully owned the technology(1) | .174 | .868 | .040 | 1 | .841 | 1.191 | .217 | 6.531 |
|  | It is more strategic to adopt M-Health when the hospital fully owned the technology(2) | -1.102 | .740 | 2.218 | 1 | .136 | .332 | .078 | 1.417 |
|  | It is more strategic to adopt M-Health when the hospital fully owned the technology(3) | -1.094 | .507 | 4.659 | 1 | .031 | .335 | .124 | .904 |
|  | Constant | .236 | 1.290 | .033 | 1 | .855 | 1.266 |  |  |
| a. Variable(s) entered on step 1: Use of M-health is superior and more advantageous than current manual practices., M-Health improves efficiency of hospital’s operations , M-health will reduce the cost of health care to patients, M-Health is compatible with current hospital’s health information system and consistent with its values and needs., M-Health can be trusted in terms of its security and confidentiality of patient information, M-Health will make many key staff redundant, M-Health is difficult to understand, use and integrate in the hospital systems and operations, Adoption of M-Health innovations requires exorbitant cost in infrastructure and human resources development, M-Health needs to be piloted first in order to demonstrate that it is better than using current manual systems, M-Health can be piloted without serious negative impact to patients and hospital operations, It is more strategic to adopt M-Health when the technology is leased to the hospital, It is more strategic to adopt M-Health when the hospital fully owned the technology. | | | | | | | | | |

1. *Information and decision support systems and technological determinants*

LOGISTIC REGRESSION VARIABLES Q20.11

/METHOD=ENTER Q23.1 Q23.2 Q23.3 Q23.4 Q23.5 Q23.6 Q23.7 Q23.8 Q23.9 Q23.10 Q23.11 Q23.12

/CONTRAST (Q23.1)=Indicator

/CONTRAST (Q23.2)=Indicator

/CONTRAST (Q23.3)=Indicator

/CONTRAST (Q23.4)=Indicator

/CONTRAST (Q23.5)=Indicator

/CONTRAST (Q23.6)=Indicator

/CONTRAST (Q23.7)=Indicator

/CONTRAST (Q23.8)=Indicator

/CONTRAST (Q23.9)=Indicator

/CONTRAST (Q23.10)=Indicator

/CONTRAST (Q23.11)=Indicator

/CONTRAST (Q23.12)=Indicator

/SAVE=PGROUP

/PRINT=GOODFIT SUMMARY CI(95)

/CRITERIA=PIN(0.05) POUT(0.10) ITERATE(20) CUT(0.5).

**Logistic Regression**

| **Notes** | | |
| --- | --- | --- |
| Output Created | | 15-JUL-2018 13:35:45 |
| Comments | |  |
| Input | Data | C:\Users\Ocholap\Downloads\m-health data.- 13-JULY 2018.sav |
|  | Active Dataset | DataSet1 |
|  | Filter | <none> |
|  | Weight | <none> |
|  | Split File | <none> |
|  | N of Rows in Working Data File | 211 |
| Missing Value Handling | Definition of Missing | User-defined missing values are treated as missing |
| Syntax | | LOGISTIC REGRESSION VARIABLES Q20.11  /METHOD=ENTER Q23.1 Q23.2 Q23.3 Q23.4 Q23.5 Q23.6 Q23.7 Q23.8 Q23.9 Q23.10 Q23.11 Q23.12  /CONTRAST (Q23.1)=Indicator  /CONTRAST (Q23.2)=Indicator  /CONTRAST (Q23.3)=Indicator  /CONTRAST (Q23.4)=Indicator  /CONTRAST (Q23.5)=Indicator  /CONTRAST (Q23.6)=Indicator  /CONTRAST (Q23.7)=Indicator  /CONTRAST (Q23.8)=Indicator  /CONTRAST (Q23.9)=Indicator  /CONTRAST (Q23.10)=Indicator  /CONTRAST (Q23.11)=Indicator  /CONTRAST (Q23.12)=Indicator  /SAVE=PGROUP  /PRINT=GOODFIT SUMMARY CI(95)  /CRITERIA=PIN(0.05) POUT(0.10) ITERATE(20) CUT(0.5). |
| Resources | Processor Time | 00:00:00.08 |
|  | Elapsed Time | 00:00:00.52 |
| Variables Created or Modified | PGR_25 | Predicted group |

| **Case Processing Summary** | | | |
| --- | --- | --- | --- |
| Unweighted Cases^a^ | | N | Percent |
| Selected Cases | Included in Analysis | 162 | 76.8 |
|  | Missing Cases | 49 | 23.2 |
|  | Total | 211 | 100.0 |
| Unselected Cases | | 0 | .0 |
| Total | | 211 | 100.0 |
| a. If weight is in effect, see classification table for the total number of cases. | | | |

| **Dependent Variable Encoding** | |
| --- | --- |
| Original Value | Internal Value |
| Not adopted | 0 |
| Adopted | 1 |

| **Categorical Variables Codings** | | | | | |
| --- | --- | --- | --- | --- | --- |
|  | | Frequency | Parameter coding | | |
|  |  |  | (1) | (2) | (3) |
| It is more strategic to adopt M-Health when the hospital fully owned the technology | Strongly disgree | 13 | 1.000 | .000 | .000 |
|  | Disagree | 23 | .000 | 1.000 | .000 |
|  | Agree | 58 | .000 | .000 | 1.000 |
|  | Strongly Agree | 68 | .000 | .000 | .000 |
| M-Health improves efficiency of hospital’s operations | Strongly disgree | 6 | 1.000 | .000 | .000 |
|  | Disagree | 8 | .000 | 1.000 | .000 |
|  | Agree | 61 | .000 | .000 | 1.000 |
|  | Strongly Agree | 87 | .000 | .000 | .000 |
| M-health will reduce the cost of health care to patients | Strongly disgree | 8 | 1.000 | .000 | .000 |
|  | Disagree | 22 | .000 | 1.000 | .000 |
|  | Agree | 54 | .000 | .000 | 1.000 |
|  | Strongly Agree | 78 | .000 | .000 | .000 |
| M-Health is compatible with current hospital’s health information system and consistent with its values and needs. | Strongly disgree | 16 | 1.000 | .000 | .000 |
|  | Disagree | 29 | .000 | 1.000 | .000 |
|  | Agree | 63 | .000 | .000 | 1.000 |
|  | Strongly Agree | 54 | .000 | .000 | .000 |
| M-Health can be trusted in terms of its security and confidentiality of patient information | Strongly disgree | 8 | 1.000 | .000 | .000 |
|  | Disagree | 27 | .000 | 1.000 | .000 |
|  | Agree | 84 | .000 | .000 | 1.000 |
|  | Strongly Agree | 43 | .000 | .000 | .000 |
| M-Health will make many key staff redundant | Strongly disgree | 43 | 1.000 | .000 | .000 |
|  | Disagree | 65 | .000 | 1.000 | .000 |
|  | Agree | 37 | .000 | .000 | 1.000 |
|  | Strongly Agree | 17 | .000 | .000 | .000 |
| M-Health is difficult to understand, use and integrate in the hospital systems and operations | Strongly disgree | 52 | 1.000 | .000 | .000 |
|  | Disagree | 74 | .000 | 1.000 | .000 |
|  | Agree | 29 | .000 | .000 | 1.000 |
|  | Strongly Agree | 7 | .000 | .000 | .000 |
| It is more strategic to adopt M-Health when the technology is leased to the hospital | Strongly disgree | 34 | 1.000 | .000 | .000 |
|  | Disagree | 62 | .000 | 1.000 | .000 |
|  | Agree | 40 | .000 | .000 | 1.000 |
|  | Strongly Agree | 26 | .000 | .000 | .000 |
| M-Health can be piloted without serious negative impact to patients and hospital operations | Strongly disgree | 6 | 1.000 | .000 | .000 |
|  | Disagree | 25 | .000 | 1.000 | .000 |
|  | Agree | 81 | .000 | .000 | 1.000 |
|  | Strongly Agree | 50 | .000 | .000 | .000 |
| M-Health needs to be piloted first in order to demonstrate that it is better than using current manual systems | Strongly disgree | 17 | 1.000 | .000 | .000 |
|  | Disagree | 18 | .000 | 1.000 | .000 |
|  | Agree | 58 | .000 | .000 | 1.000 |
|  | Strongly Agree | 69 | .000 | .000 | .000 |
| Adoption of M-Health innovations requires exorbitant cost in infrastructure and human resources development | Strongly disgree | 26 | 1.000 | .000 | .000 |
|  | Disagree | 52 | .000 | 1.000 | .000 |
|  | Agree | 59 | .000 | .000 | 1.000 |
|  | Strongly Agree | 25 | .000 | .000 | .000 |
| Use of M-health is superior and more advantageous than current manual practices. | Strongly disgree | 10 | 1.000 | .000 | .000 |
|  | Disagree | 14 | .000 | 1.000 | .000 |
|  | Agree | 62 | .000 | .000 | 1.000 |
|  | Strongly Agree | 76 | .000 | .000 | .000 |

**Block 0: Beginning Block**

| **Classification Table^a,b^** | | | | | |
| --- | --- | --- | --- | --- | --- |
|  | Observed | | Predicted | | |
|  |  |  | Information and decision support systems | | Percentage Correct |
|  |  |  | Not adopted | Adopted |  |
| Step 0 | Information and decision support systems | Not adopted | 0 | 75 | .0 |
|  |  | Adopted | 0 | 87 | 100.0 |
|  | Overall Percentage | |  |  | 53.7 |
| a. Constant is included in the model. | | | | | |
| b. The cut value is .500 | | | | | |

| **Variables in the Equation** | | | | | | | |
| --- | --- | --- | --- | --- | --- | --- | --- |
|  | | B | S.E. | Wald | df | Sig. | Exp(B) |
| Step 0 | Constant | .148 | .158 | .887 | 1 | .346 | 1.160 |

| **Variables not in the Equation** | | | | | |
| --- | --- | --- | --- | --- | --- |
|  | | | Score | df | Sig. |
| Step 0 | Variables | Use of M-health is superior and more advantageous than current manual practices. | 3.251 | 3 | .355 |
|  |  | Use of M-health is superior and more advantageous than current manual practices.(1) | .170 | 1 | .680 |
|  |  | Use of M-health is superior and more advantageous than current manual practices.(2) | 1.995 | 1 | .158 |
|  |  | Use of M-health is superior and more advantageous than current manual practices.(3) | .554 | 1 | .457 |
|  |  | M-Health improves efficiency of hospital’s operations | 2.858 | 3 | .414 |
|  |  | M-Health improves efficiency of hospital’s operations (1) | .034 | 1 | .853 |
|  |  | M-Health improves efficiency of hospital’s operations (2) | .046 | 1 | .829 |
|  |  | M-Health improves efficiency of hospital’s operations (3) | 2.395 | 1 | .122 |
|  |  | M-health will reduce the cost of health care to patients | 7.744 | 3 | .052 |
|  |  | M-health will reduce the cost of health care to patients(1) | 1.535 | 1 | .215 |
|  |  | M-health will reduce the cost of health care to patients(2) | 4.904 | 1 | .027 |
|  |  | M-health will reduce the cost of health care to patients(3) | 2.793 | 1 | .095 |
|  |  | M-Health is compatible with current hospital’s health information system and consistent with its values and needs. | 16.942 | 3 | .001 |
|  |  | M-Health is compatible with current hospital’s health information system and consistent with its values and needs.(1) | .552 | 1 | .457 |
|  |  | M-Health is compatible with current hospital’s health information system and consistent with its values and needs.(2) | 12.419 | 1 | .000 |
|  |  | M-Health is compatible with current hospital’s health information system and consistent with its values and needs.(3) | .351 | 1 | .553 |
|  |  | M-Health can be trusted in terms of its security and confidentiality of patient information | 3.646 | 3 | .302 |
|  |  | M-Health can be trusted in terms of its security and confidentiality of patient information(1) | .262 | 1 | .609 |
|  |  | M-Health can be trusted in terms of its security and confidentiality of patient information(2) | .402 | 1 | .526 |
|  |  | M-Health can be trusted in terms of its security and confidentiality of patient information(3) | 1.681 | 1 | .195 |
|  |  | M-Health will make many key staff redundant | 8.613 | 3 | .035 |
|  |  | M-Health will make many key staff redundant(1) | 6.075 | 1 | .014 |
|  |  | M-Health will make many key staff redundant(2) | .376 | 1 | .540 |
|  |  | M-Health will make many key staff redundant(3) | 4.855 | 1 | .028 |
|  |  | M-Health is difficult to understand, use and integrate in the hospital systems and operations | 7.465 | 3 | .058 |
|  |  | M-Health is difficult to understand, use and integrate in the hospital systems and operations(1) | 5.700 | 1 | .017 |
|  |  | M-Health is difficult to understand, use and integrate in the hospital systems and operations(2) | 3.298 | 1 | .069 |
|  |  | M-Health is difficult to understand, use and integrate in the hospital systems and operations(3) | 1.119 | 1 | .290 |
|  |  | Adoption of M-Health innovations requires exorbitant cost in infrastructure and human resources development | .999 | 3 | .801 |
|  |  | Adoption of M-Health innovations requires exorbitant cost in infrastructure and human resources development(1) | .198 | 1 | .656 |
|  |  | Adoption of M-Health innovations requires exorbitant cost in infrastructure and human resources development(2) | .975 | 1 | .323 |
|  |  | Adoption of M-Health innovations requires exorbitant cost in infrastructure and human resources development(3) | .185 | 1 | .667 |
|  |  | M-Health needs to be piloted first in order to demonstrate that it is better than using current manual systems | 1.173 | 3 | .760 |
|  |  | M-Health needs to be piloted first in order to demonstrate that it is better than using current manual systems(1) | .925 | 1 | .336 |
|  |  | M-Health needs to be piloted first in order to demonstrate that it is better than using current manual systems(2) | .028 | 1 | .867 |
|  |  | M-Health needs to be piloted first in order to demonstrate that it is better than using current manual systems(3) | .498 | 1 | .480 |
|  |  | M-Health can be piloted without serious negative impact to patients and hospital operations | 16.189 | 3 | .001 |
|  |  | M-Health can be piloted without serious negative impact to patients and hospital operations(1) | 5.371 | 1 | .020 |
|  |  | M-Health can be piloted without serious negative impact to patients and hospital operations(2) | 10.491 | 1 | .001 |
|  |  | M-Health can be piloted without serious negative impact to patients and hospital operations(3) | .025 | 1 | .875 |
|  |  | It is more strategic to adopt M-Health when the technology is leased to the hospital | 1.783 | 3 | .619 |
|  |  | It is more strategic to adopt M-Health when the technology is leased to the hospital(1) | 1.125 | 1 | .289 |
|  |  | It is more strategic to adopt M-Health when the technology is leased to the hospital(2) | .177 | 1 | .674 |
|  |  | It is more strategic to adopt M-Health when the technology is leased to the hospital(3) | .822 | 1 | .365 |
|  |  | It is more strategic to adopt M-Health when the hospital fully owned the technology | 2.294 | 3 | .514 |
|  |  | It is more strategic to adopt M-Health when the hospital fully owned the technology(1) | 1.371 | 1 | .242 |
|  |  | It is more strategic to adopt M-Health when the hospital fully owned the technology(2) | 1.127 | 1 | .288 |
|  |  | It is more strategic to adopt M-Health when the hospital fully owned the technology(3) | .078 | 1 | .779 |
|  | Overall Statistics | | 56.012 | 36 | .018 |

**Block 1: Method = Enter**

| **Omnibus Tests of Model Coefficients** | | | | |
| --- | --- | --- | --- | --- |
|  | | Chi-square | df | Sig. |
| Step 1 | Step | 68.257 | 36 | .001 |
|  | Block | 68.257 | 36 | .001 |
|  | Model | 68.257 | 36 | .001 |

| **Model Summary** | | | |
| --- | --- | --- | --- |
| Step | -2 Log likelihood | Cox & Snell R Square | Nagelkerke R Square |
| 1 | 155.433^a^ | .344 | .459 |
| a. Estimation terminated at iteration number 20 because maximum iterations has been reached. Final solution cannot be found. | | | |

| **Hosmer and Lemeshow Test** | | | |
| --- | --- | --- | --- |
| Step | Chi-square | df | Sig. |
| 1 | 6.977 | 8 | .539 |

| **Contingency Table for Hosmer and Lemeshow Test** | | | | | | |
| --- | --- | --- | --- | --- | --- | --- |
|  | | Information and decision support systems = Not adopted | | Information and decision support systems = Adopted | | Total |
|  |  | Observed | Expected | Observed | Expected |  |
| Step 1 | 1 | 15 | 14.851 | 1 | 1.149 | 16 |
|  | 2 | 13 | 13.554 | 3 | 2.446 | 16 |
|  | 3 | 13 | 11.706 | 3 | 4.294 | 16 |
|  | 4 | 10 | 9.881 | 6 | 6.119 | 16 |
|  | 5 | 11 | 8.344 | 5 | 7.656 | 16 |
|  | 6 | 3 | 6.684 | 13 | 9.316 | 16 |
|  | 7 | 5 | 4.361 | 11 | 11.639 | 16 |
|  | 8 | 3 | 3.165 | 13 | 12.835 | 16 |
|  | 9 | 1 | 1.908 | 15 | 14.092 | 16 |
|  | 10 | 1 | .547 | 17 | 17.453 | 18 |

| **Classification Table^a^** | | | | | |
| --- | --- | --- | --- | --- | --- |
|  | Observed | | Predicted | | |
|  |  |  | Information and decision support systems | | Percentage Correct |
|  |  |  | Not adopted | Adopted |  |
| Step 1 | Information and decision support systems | Not adopted | 57 | 18 | 76.0 |
|  |  | Adopted | 16 | 71 | 81.6 |
|  | Overall Percentage | |  |  | 79.0 |
| a. The cut value is .500 | | | | | |

| **Variables in the Equation** | | | | | | | | | |
| --- | --- | --- | --- | --- | --- | --- | --- | --- | --- |
|  | | B | S.E. | Wald | df | Sig. | Exp(B) | 95% C.I.for EXP(B) | |
|  |  |  |  |  |  |  |  | Lower | Upper |
| Step 1^a^ | Use of M-health is superior and more advantageous than current manual practices. |  |  | 1.081 | 3 | .782 |  |  |  |
|  | Use of M-health is superior and more advantageous than current manual practices.(1) | .799 | 1.208 | .438 | 1 | .508 | 2.224 | .208 | 23.724 |
|  | Use of M-health is superior and more advantageous than current manual practices.(2) | .946 | 1.123 | .709 | 1 | .400 | 2.576 | .285 | 23.289 |
|  | Use of M-health is superior and more advantageous than current manual practices.(3) | .290 | .647 | .201 | 1 | .654 | 1.336 | .376 | 4.751 |
|  | M-Health improves efficiency of hospital’s operations |  |  | 3.698 | 3 | .296 |  |  |  |
|  | M-Health improves efficiency of hospital’s operations (1) | -4.753 | 2.485 | 3.659 | 1 | .056 | .009 | .000 | 1.124 |
|  | M-Health improves efficiency of hospital’s operations (2) | -.702 | 1.289 | .296 | 1 | .586 | .496 | .040 | 6.199 |
|  | M-Health improves efficiency of hospital’s operations (3) | -.300 | .731 | .168 | 1 | .682 | .741 | .177 | 3.107 |
|  | M-health will reduce the cost of health care to patients |  |  | 11.236 | 3 | .011 |  |  |  |
|  | M-health will reduce the cost of health care to patients(1) | 3.694 | 1.889 | 3.825 | 1 | .050 | 40.186 | .992 | 1627.799 |
|  | M-health will reduce the cost of health care to patients(2) | .739 | .893 | .686 | 1 | .408 | 2.095 | .364 | 12.050 |
|  | M-health will reduce the cost of health care to patients(3) | 2.001 | .674 | 8.803 | 1 | .003 | 7.393 | 1.972 | 27.720 |
|  | M-Health is compatible with current hospital’s health information system and consistent with its values and needs. |  |  | 9.883 | 3 | .020 |  |  |  |
|  | M-Health is compatible with current hospital’s health information system and consistent with its values and needs.(1) | -.249 | .973 | .065 | 1 | .798 | .780 | .116 | 5.251 |
|  | M-Health is compatible with current hospital’s health information system and consistent with its values and needs.(2) | -2.245 | .778 | 8.329 | 1 | .004 | .106 | .023 | .487 |
|  | M-Health is compatible with current hospital’s health information system and consistent with its values and needs.(3) | -.518 | .586 | .782 | 1 | .377 | .596 | .189 | 1.878 |
|  | M-Health can be trusted in terms of its security and confidentiality of patient information |  |  | 2.548 | 3 | .467 |  |  |  |
|  | M-Health can be trusted in terms of its security and confidentiality of patient information(1) | -.047 | 1.735 | .001 | 1 | .978 | .954 | .032 | 28.604 |
|  | M-Health can be trusted in terms of its security and confidentiality of patient information(2) | .450 | .784 | .329 | 1 | .566 | 1.568 | .337 | 7.285 |
|  | M-Health can be trusted in terms of its security and confidentiality of patient information(3) | -.579 | .628 | .851 | 1 | .356 | .560 | .164 | 1.918 |
|  | M-Health will make many key staff redundant |  |  | 3.111 | 3 | .375 |  |  |  |
|  | M-Health will make many key staff redundant(1) | .786 | .900 | .762 | 1 | .383 | 2.194 | .376 | 12.799 |
|  | M-Health will make many key staff redundant(2) | .184 | .911 | .041 | 1 | .840 | 1.202 | .201 | 7.171 |
|  | M-Health will make many key staff redundant(3) | -.390 | .940 | .172 | 1 | .678 | .677 | .107 | 4.277 |
|  | M-Health is difficult to understand, use and integrate in the hospital systems and operations |  |  | 7.488 | 3 | .058 |  |  |  |
|  | M-Health is difficult to understand, use and integrate in the hospital systems and operations(1) | .117 | 1.313 | .008 | 1 | .929 | 1.124 | .086 | 14.754 |
|  | M-Health is difficult to understand, use and integrate in the hospital systems and operations(2) | -1.610 | 1.360 | 1.401 | 1 | .236 | .200 | .014 | 2.873 |
|  | M-Health is difficult to understand, use and integrate in the hospital systems and operations(3) | -1.181 | 1.403 | .708 | 1 | .400 | .307 | .020 | 4.805 |
|  | Adoption of M-Health innovations requires exorbitant cost in infrastructure and human resources development |  |  | 3.621 | 3 | .305 |  |  |  |
|  | Adoption of M-Health innovations requires exorbitant cost in infrastructure and human resources development(1) | -.917 | .851 | 1.162 | 1 | .281 | .400 | .075 | 2.118 |
|  | Adoption of M-Health innovations requires exorbitant cost in infrastructure and human resources development(2) | -.623 | .731 | .725 | 1 | .395 | .537 | .128 | 2.249 |
|  | Adoption of M-Health innovations requires exorbitant cost in infrastructure and human resources development(3) | .233 | .727 | .103 | 1 | .748 | 1.263 | .304 | 5.252 |
|  | M-Health needs to be piloted first in order to demonstrate that it is better than using current manual systems |  |  | 4.494 | 3 | .213 |  |  |  |
|  | M-Health needs to be piloted first in order to demonstrate that it is better than using current manual systems(1) | 1.515 | .856 | 3.135 | 1 | .077 | 4.549 | .850 | 24.330 |
|  | M-Health needs to be piloted first in order to demonstrate that it is better than using current manual systems(2) | 1.055 | .879 | 1.440 | 1 | .230 | 2.873 | .513 | 16.104 |
|  | M-Health needs to be piloted first in order to demonstrate that it is better than using current manual systems(3) | .019 | .609 | .001 | 1 | .975 | 1.019 | .309 | 3.362 |
|  | M-Health can be piloted without serious negative impact to patients and hospital operations |  |  | 5.531 | 3 | .137 |  |  |  |
|  | M-Health can be piloted without serious negative impact to patients and hospital operations(1) | 20.477 | 16123.570 | .000 | 1 | .999 | 781696702.524 | .000 | . |
|  | M-Health can be piloted without serious negative impact to patients and hospital operations(2) | -1.804 | .768 | 5.519 | 1 | .019 | .165 | .037 | .742 |
|  | M-Health can be piloted without serious negative impact to patients and hospital operations(3) | -.469 | .533 | .775 | 1 | .379 | .626 | .220 | 1.777 |
|  | It is more strategic to adopt M-Health when the technology is leased to the hospital |  |  | 1.036 | 3 | .793 |  |  |  |
|  | It is more strategic to adopt M-Health when the technology is leased to the hospital(1) | .421 | .800 | .278 | 1 | .598 | 1.524 | .318 | 7.307 |
|  | It is more strategic to adopt M-Health when the technology is leased to the hospital(2) | .243 | .813 | .089 | 1 | .765 | 1.275 | .259 | 6.276 |
|  | It is more strategic to adopt M-Health when the technology is leased to the hospital(3) | -.247 | .776 | .102 | 1 | .750 | .781 | .171 | 3.572 |
|  | It is more strategic to adopt M-Health when the hospital fully owned the technology |  |  | 1.261 | 3 | .738 |  |  |  |
|  | It is more strategic to adopt M-Health when the hospital fully owned the technology(1) | .906 | 1.090 | .690 | 1 | .406 | 2.474 | .292 | 20.960 |
|  | It is more strategic to adopt M-Health when the hospital fully owned the technology(2) | -.115 | .811 | .020 | 1 | .887 | .891 | .182 | 4.374 |
|  | It is more strategic to adopt M-Health when the hospital fully owned the technology(3) | .386 | .580 | .444 | 1 | .505 | 1.472 | .472 | 4.585 |
|  | Constant | 1.024 | 1.484 | .477 | 1 | .490 | 2.785 |  |  |
| a. Variable(s) entered on step 1: Use of M-health is superior and more advantageous than current manual practices., M-Health improves efficiency of hospital’s operations , M-health will reduce the cost of health care to patients, M-Health is compatible with current hospital’s health information system and consistent with its values and needs., M-Health can be trusted in terms of its security and confidentiality of patient information, M-Health will make many key staff redundant, M-Health is difficult to understand, use and integrate in the hospital systems and operations, Adoption of M-Health innovations requires exorbitant cost in infrastructure and human resources development, M-Health needs to be piloted first in order to demonstrate that it is better than using current manual systems, M-Health can be piloted without serious negative impact to patients and hospital operations, It is more strategic to adopt M-Health when the technology is leased to the hospital, It is more strategic to adopt M-Health when the hospital fully owned the technology. | | | | | | | | | |

1. *Patient records and technological determinants*

LOGISTIC REGRESSION VARIABLES Q20.12

/METHOD=ENTER Q23.1 Q23.2 Q23.3 Q23.4 Q23.5 Q23.6 Q23.7 Q23.8 Q23.9 Q23.10 Q23.11 Q23.12

/CONTRAST (Q23.1)=Indicator

/CONTRAST (Q23.2)=Indicator

/CONTRAST (Q23.3)=Indicator

/CONTRAST (Q23.4)=Indicator

/CONTRAST (Q23.5)=Indicator

/CONTRAST (Q23.6)=Indicator

/CONTRAST (Q23.7)=Indicator

/CONTRAST (Q23.8)=Indicator

/CONTRAST (Q23.9)=Indicator

/CONTRAST (Q23.10)=Indicator

/CONTRAST (Q23.11)=Indicator

/CONTRAST (Q23.12)=Indicator

/SAVE=PGROUP

/PRINT=GOODFIT SUMMARY CI(95)

/CRITERIA=PIN(0.05) POUT(0.10) ITERATE(20) CUT(0.5).

**Logistic Regression**

| **Notes** | | |
| --- | --- | --- |
| Output Created | | 15-JUL-2018 13:36:08 |
| Comments | |  |
| Input | Data | C:\Users\Ocholap\Downloads\m-health data.- 13-JULY 2018.sav |
|  | Active Dataset | DataSet1 |
|  | Filter | <none> |
|  | Weight | <none> |
|  | Split File | <none> |
|  | N of Rows in Working Data File | 211 |
| Missing Value Handling | Definition of Missing | User-defined missing values are treated as missing |
| Syntax | | LOGISTIC REGRESSION VARIABLES Q20.12  /METHOD=ENTER Q23.1 Q23.2 Q23.3 Q23.4 Q23.5 Q23.6 Q23.7 Q23.8 Q23.9 Q23.10 Q23.11 Q23.12  /CONTRAST (Q23.1)=Indicator  /CONTRAST (Q23.2)=Indicator  /CONTRAST (Q23.3)=Indicator  /CONTRAST (Q23.4)=Indicator  /CONTRAST (Q23.5)=Indicator  /CONTRAST (Q23.6)=Indicator  /CONTRAST (Q23.7)=Indicator  /CONTRAST (Q23.8)=Indicator  /CONTRAST (Q23.9)=Indicator  /CONTRAST (Q23.10)=Indicator  /CONTRAST (Q23.11)=Indicator  /CONTRAST (Q23.12)=Indicator  /SAVE=PGROUP  /PRINT=GOODFIT SUMMARY CI(95)  /CRITERIA=PIN(0.05) POUT(0.10) ITERATE(20) CUT(0.5). |
| Resources | Processor Time | 00:00:00.06 |
|  | Elapsed Time | 00:00:00.06 |
| Variables Created or Modified | PGR_26 | Predicted group |

| **Case Processing Summary** | | | |
| --- | --- | --- | --- |
| Unweighted Cases^a^ | | N | Percent |
| Selected Cases | Included in Analysis | 167 | 79.1 |
|  | Missing Cases | 44 | 20.9 |
|  | Total | 211 | 100.0 |
| Unselected Cases | | 0 | .0 |
| Total | | 211 | 100.0 |
| a. If weight is in effect, see classification table for the total number of cases. | | | |

| **Dependent Variable Encoding** | |
| --- | --- |
| Original Value | Internal Value |
| Not adopted | 0 |
| Adopted | 1 |

| **Categorical Variables Codings** | | | | | |
| --- | --- | --- | --- | --- | --- |
|  | | Frequency | Parameter coding | | |
|  |  |  | (1) | (2) | (3) |
| It is more strategic to adopt M-Health when the hospital fully owned the technology | Strongly disgree | 14 | 1.000 | .000 | .000 |
|  | Disagree | 24 | .000 | 1.000 | .000 |
|  | Agree | 61 | .000 | .000 | 1.000 |
|  | Strongly Agree | 68 | .000 | .000 | .000 |
| M-Health improves efficiency of hospital’s operations | Strongly disgree | 6 | 1.000 | .000 | .000 |
|  | Disagree | 8 | .000 | 1.000 | .000 |
|  | Agree | 63 | .000 | .000 | 1.000 |
|  | Strongly Agree | 90 | .000 | .000 | .000 |
| M-health will reduce the cost of health care to patients | Strongly disgree | 8 | 1.000 | .000 | .000 |
|  | Disagree | 23 | .000 | 1.000 | .000 |
|  | Agree | 57 | .000 | .000 | 1.000 |
|  | Strongly Agree | 79 | .000 | .000 | .000 |
| M-Health is compatible with current hospital’s health information system and consistent with its values and needs. | Strongly disgree | 16 | 1.000 | .000 | .000 |
|  | Disagree | 29 | .000 | 1.000 | .000 |
|  | Agree | 66 | .000 | .000 | 1.000 |
|  | Strongly Agree | 56 | .000 | .000 | .000 |
| M-Health can be trusted in terms of its security and confidentiality of patient information | Strongly disgree | 9 | 1.000 | .000 | .000 |
|  | Disagree | 30 | .000 | 1.000 | .000 |
|  | Agree | 84 | .000 | .000 | 1.000 |
|  | Strongly Agree | 44 | .000 | .000 | .000 |
| M-Health will make many key staff redundant | Strongly disgree | 43 | 1.000 | .000 | .000 |
|  | Disagree | 66 | .000 | 1.000 | .000 |
|  | Agree | 39 | .000 | .000 | 1.000 |
|  | Strongly Agree | 19 | .000 | .000 | .000 |
| M-Health is difficult to understand, use and integrate in the hospital systems and operations | Strongly disgree | 53 | 1.000 | .000 | .000 |
|  | Disagree | 76 | .000 | 1.000 | .000 |
|  | Agree | 31 | .000 | .000 | 1.000 |
|  | Strongly Agree | 7 | .000 | .000 | .000 |
| It is more strategic to adopt M-Health when the technology is leased to the hospital | Strongly disgree | 34 | 1.000 | .000 | .000 |
|  | Disagree | 64 | .000 | 1.000 | .000 |
|  | Agree | 43 | .000 | .000 | 1.000 |
|  | Strongly Agree | 26 | .000 | .000 | .000 |
| M-Health can be piloted without serious negative impact to patients and hospital operations | Strongly disgree | 6 | 1.000 | .000 | .000 |
|  | Disagree | 26 | .000 | 1.000 | .000 |
|  | Agree | 85 | .000 | .000 | 1.000 |
|  | Strongly Agree | 50 | .000 | .000 | .000 |
| M-Health needs to be piloted first in order to demonstrate that it is better than using current manual systems | Strongly disgree | 17 | 1.000 | .000 | .000 |
|  | Disagree | 19 | .000 | 1.000 | .000 |
|  | Agree | 61 | .000 | .000 | 1.000 |
|  | Strongly Agree | 70 | .000 | .000 | .000 |
| Adoption of M-Health innovations requires exorbitant cost in infrastructure and human resources development | Strongly disgree | 26 | 1.000 | .000 | .000 |
|  | Disagree | 55 | .000 | 1.000 | .000 |
|  | Agree | 61 | .000 | .000 | 1.000 |
|  | Strongly Agree | 25 | .000 | .000 | .000 |
| Use of M-health is superior and more advantageous than current manual practices. | Strongly disgree | 10 | 1.000 | .000 | .000 |
|  | Disagree | 14 | .000 | 1.000 | .000 |
|  | Agree | 65 | .000 | .000 | 1.000 |
|  | Strongly Agree | 78 | .000 | .000 | .000 |

**Block 0: Beginning Block**

| **Classification Table^a,b^** | | | | | |
| --- | --- | --- | --- | --- | --- |
|  | Observed | | Predicted | | |
|  |  |  | Patient records | | Percentage Correct |
|  |  |  | Not adopted | Adopted |  |
| Step 0 | Patient records | Not adopted | 86 | 0 | 100.0 |
|  |  | Adopted | 81 | 0 | .0 |
|  | Overall Percentage | |  |  | 51.5 |
| a. Constant is included in the model. | | | | | |
| b. The cut value is .500 | | | | | |

| **Variables in the Equation** | | | | | | | |
| --- | --- | --- | --- | --- | --- | --- | --- |
|  | | B | S.E. | Wald | df | Sig. | Exp(B) |
| Step 0 | Constant | -.060 | .155 | .150 | 1 | .699 | .942 |

| **Variables not in the Equation** | | | | | |
| --- | --- | --- | --- | --- | --- |
|  | | | Score | df | Sig. |
| Step 0 | Variables | Use of M-health is superior and more advantageous than current manual practices. | 4.921 | 3 | .178 |
|  |  | Use of M-health is superior and more advantageous than current manual practices.(1) | 1.968 | 1 | .161 |
|  |  | Use of M-health is superior and more advantageous than current manual practices.(2) | 1.001 | 1 | .317 |
|  |  | Use of M-health is superior and more advantageous than current manual practices.(3) | 2.067 | 1 | .151 |
|  |  | M-Health improves efficiency of hospital’s operations | 2.205 | 3 | .531 |
|  |  | M-Health improves efficiency of hospital’s operations (1) | .573 | 1 | .449 |
|  |  | M-Health improves efficiency of hospital’s operations (2) | .008 | 1 | .931 |
|  |  | M-Health improves efficiency of hospital’s operations (3) | 1.291 | 1 | .256 |
|  |  | M-health will reduce the cost of health care to patients | 2.755 | 3 | .431 |
|  |  | M-health will reduce the cost of health care to patients(1) | .659 | 1 | .417 |
|  |  | M-health will reduce the cost of health care to patients(2) | 2.010 | 1 | .156 |
|  |  | M-health will reduce the cost of health care to patients(3) | .045 | 1 | .833 |
|  |  | M-Health is compatible with current hospital’s health information system and consistent with its values and needs. | 13.021 | 3 | .005 |
|  |  | M-Health is compatible with current hospital’s health information system and consistent with its values and needs.(1) | .858 | 1 | .354 |
|  |  | M-Health is compatible with current hospital’s health information system and consistent with its values and needs.(2) | 6.147 | 1 | .013 |
|  |  | M-Health is compatible with current hospital’s health information system and consistent with its values and needs.(3) | .406 | 1 | .524 |
|  |  | M-Health can be trusted in terms of its security and confidentiality of patient information | 8.938 | 3 | .030 |
|  |  | M-Health can be trusted in terms of its security and confidentiality of patient information(1) | 2.630 | 1 | .105 |
|  |  | M-Health can be trusted in terms of its security and confidentiality of patient information(2) | .049 | 1 | .824 |
|  |  | M-Health can be trusted in terms of its security and confidentiality of patient information(3) | 2.157 | 1 | .142 |
|  |  | M-Health will make many key staff redundant | 5.545 | 3 | .136 |
|  |  | M-Health will make many key staff redundant(1) | 4.733 | 1 | .030 |
|  |  | M-Health will make many key staff redundant(2) | 2.520 | 1 | .112 |
|  |  | M-Health will make many key staff redundant(3) | .492 | 1 | .483 |
|  |  | M-Health is difficult to understand, use and integrate in the hospital systems and operations | 3.854 | 3 | .278 |
|  |  | M-Health is difficult to understand, use and integrate in the hospital systems and operations(1) | 1.200 | 1 | .273 |
|  |  | M-Health is difficult to understand, use and integrate in the hospital systems and operations(2) | 3.322 | 1 | .068 |
|  |  | M-Health is difficult to understand, use and integrate in the hospital systems and operations(3) | 1.393 | 1 | .238 |
|  |  | Adoption of M-Health innovations requires exorbitant cost in infrastructure and human resources development | 1.119 | 3 | .773 |
|  |  | Adoption of M-Health innovations requires exorbitant cost in infrastructure and human resources development(1) | .028 | 1 | .868 |
|  |  | Adoption of M-Health innovations requires exorbitant cost in infrastructure and human resources development(2) | .778 | 1 | .378 |
|  |  | Adoption of M-Health innovations requires exorbitant cost in infrastructure and human resources development(3) | .018 | 1 | .894 |
|  |  | M-Health needs to be piloted first in order to demonstrate that it is better than using current manual systems | .964 | 3 | .810 |
|  |  | M-Health needs to be piloted first in order to demonstrate that it is better than using current manual systems(1) | .807 | 1 | .369 |
|  |  | M-Health needs to be piloted first in order to demonstrate that it is better than using current manual systems(2) | .011 | 1 | .916 |
|  |  | M-Health needs to be piloted first in order to demonstrate that it is better than using current manual systems(3) | .018 | 1 | .894 |
|  |  | M-Health can be piloted without serious negative impact to patients and hospital operations | 3.868 | 3 | .276 |
|  |  | M-Health can be piloted without serious negative impact to patients and hospital operations(1) | 3.023 | 1 | .082 |
|  |  | M-Health can be piloted without serious negative impact to patients and hospital operations(2) | .473 | 1 | .492 |
|  |  | M-Health can be piloted without serious negative impact to patients and hospital operations(3) | .301 | 1 | .583 |
|  |  | It is more strategic to adopt M-Health when the technology is leased to the hospital | 3.583 | 3 | .310 |
|  |  | It is more strategic to adopt M-Health when the technology is leased to the hospital(1) | 1.821 | 1 | .177 |
|  |  | It is more strategic to adopt M-Health when the technology is leased to the hospital(2) | .093 | 1 | .760 |
|  |  | It is more strategic to adopt M-Health when the technology is leased to the hospital(3) | .092 | 1 | .762 |
|  |  | It is more strategic to adopt M-Health when the hospital fully owned the technology | .319 | 3 | .956 |
|  |  | It is more strategic to adopt M-Health when the hospital fully owned the technology(1) | .195 | 1 | .659 |
|  |  | It is more strategic to adopt M-Health when the hospital fully owned the technology(2) | .080 | 1 | .777 |
|  |  | It is more strategic to adopt M-Health when the hospital fully owned the technology(3) | .018 | 1 | .894 |
|  | Overall Statistics | | 46.924 | 36 | .105 |

**Block 1: Method = Enter**

| **Omnibus Tests of Model Coefficients** | | | | |
| --- | --- | --- | --- | --- |
|  | | Chi-square | df | Sig. |
| Step 1 | Step | 59.702 | 36 | .008 |
|  | Block | 59.702 | 36 | .008 |
|  | Model | 59.702 | 36 | .008 |

| **Model Summary** | | | |
| --- | --- | --- | --- |
| Step | -2 Log likelihood | Cox & Snell R Square | Nagelkerke R Square |
| 1 | 171.659^a^ | .301 | .401 |
| a. Estimation terminated at iteration number 20 because maximum iterations has been reached. Final solution cannot be found. | | | |

| **Hosmer and Lemeshow Test** | | | |
| --- | --- | --- | --- |
| Step | Chi-square | df | Sig. |
| 1 | 14.301 | 8 | .074 |

| **Contingency Table for Hosmer and Lemeshow Test** | | | | | | |
| --- | --- | --- | --- | --- | --- | --- |
|  | | Patient records = Not adopted | | Patient records = Adopted | | Total |
|  |  | Observed | Expected | Observed | Expected |  |
| Step 1 | 1 | 14 | 15.847 | 3 | 1.153 | 17 |
|  | 2 | 15 | 13.778 | 2 | 3.222 | 17 |
|  | 3 | 12 | 12.647 | 5 | 4.353 | 17 |
|  | 4 | 14 | 11.892 | 4 | 6.108 | 18 |
|  | 5 | 9 | 9.904 | 8 | 7.096 | 17 |
|  | 6 | 10 | 8.208 | 7 | 8.792 | 17 |
|  | 7 | 7 | 6.367 | 10 | 10.633 | 17 |
|  | 8 | 0 | 4.342 | 17 | 12.658 | 17 |
|  | 9 | 5 | 2.758 | 12 | 14.242 | 17 |
|  | 10 | 0 | .258 | 13 | 12.742 | 13 |

| **Classification Table^a^** | | | | | |
| --- | --- | --- | --- | --- | --- |
|  | Observed | | Predicted | | |
|  |  |  | Patient records | | Percentage Correct |
|  |  |  | Not adopted | Adopted |  |
| Step 1 | Patient records | Not adopted | 69 | 17 | 80.2 |
|  |  | Adopted | 23 | 58 | 71.6 |
|  | Overall Percentage | |  |  | 76.0 |
| a. The cut value is .500 | | | | | |

| **Variables in the Equation** | | | | | | | | | |
| --- | --- | --- | --- | --- | --- | --- | --- | --- | --- |
|  | | B | S.E. | Wald | df | Sig. | Exp(B) | 95% C.I.for EXP(B) | |
|  |  |  |  |  |  |  |  | Lower | Upper |
| Step 1^a^ | Use of M-health is superior and more advantageous than current manual practices. |  |  | .214 | 3 | .975 |  |  |  |
|  | Use of M-health is superior and more advantageous than current manual practices.(1) | 21.978 | 13981.546 | .000 | 1 | .999 | 3507013116.212 | .000 | . |
|  | Use of M-health is superior and more advantageous than current manual practices.(2) | -.135 | .992 | .019 | 1 | .892 | .873 | .125 | 6.103 |
|  | Use of M-health is superior and more advantageous than current manual practices.(3) | -.253 | .556 | .208 | 1 | .648 | .776 | .261 | 2.306 |
|  | M-Health improves efficiency of hospital’s operations |  |  | .417 | 3 | .937 |  |  |  |
|  | M-Health improves efficiency of hospital’s operations (1) | -23.532 | 13981.546 | .000 | 1 | .999 | .000 | .000 | . |
|  | M-Health improves efficiency of hospital’s operations (2) | -.426 | 1.117 | .145 | 1 | .703 | .653 | .073 | 5.832 |
|  | M-Health improves efficiency of hospital’s operations (3) | -.411 | .643 | .408 | 1 | .523 | .663 | .188 | 2.338 |
|  | M-health will reduce the cost of health care to patients |  |  | 2.461 | 3 | .482 |  |  |  |
|  | M-health will reduce the cost of health care to patients(1) | 1.927 | 1.493 | 1.664 | 1 | .197 | 6.866 | .368 | 128.213 |
|  | M-health will reduce the cost of health care to patients(2) | .214 | .782 | .074 | 1 | .785 | 1.238 | .267 | 5.736 |
|  | M-health will reduce the cost of health care to patients(3) | .606 | .589 | 1.058 | 1 | .304 | 1.833 | .578 | 5.818 |
|  | M-Health is compatible with current hospital’s health information system and consistent with its values and needs. |  |  | 7.660 | 3 | .054 |  |  |  |
|  | M-Health is compatible with current hospital’s health information system and consistent with its values and needs.(1) | -.788 | .914 | .744 | 1 | .388 | .455 | .076 | 2.726 |
|  | M-Health is compatible with current hospital’s health information system and consistent with its values and needs.(2) | -2.078 | .756 | 7.544 | 1 | .006 | .125 | .028 | .552 |
|  | M-Health is compatible with current hospital’s health information system and consistent with its values and needs.(3) | -.634 | .564 | 1.266 | 1 | .261 | .530 | .176 | 1.601 |
|  | M-Health can be trusted in terms of its security and confidentiality of patient information |  |  | 8.810 | 3 | .032 |  |  |  |
|  | M-Health can be trusted in terms of its security and confidentiality of patient information(1) | -4.144 | 1.637 | 6.410 | 1 | .011 | .016 | .001 | .392 |
|  | M-Health can be trusted in terms of its security and confidentiality of patient information(2) | .195 | .708 | .076 | 1 | .783 | 1.216 | .304 | 4.868 |
|  | M-Health can be trusted in terms of its security and confidentiality of patient information(3) | -.848 | .586 | 2.091 | 1 | .148 | .428 | .136 | 1.352 |
|  | M-Health will make many key staff redundant |  |  | 5.434 | 3 | .143 |  |  |  |
|  | M-Health will make many key staff redundant(1) | .764 | .887 | .742 | 1 | .389 | 2.147 | .377 | 12.220 |
|  | M-Health will make many key staff redundant(2) | -.604 | .904 | .446 | 1 | .504 | .547 | .093 | 3.214 |
|  | M-Health will make many key staff redundant(3) | -.134 | .935 | .021 | 1 | .886 | .874 | .140 | 5.460 |
|  | M-Health is difficult to understand, use and integrate in the hospital systems and operations |  |  | 2.260 | 3 | .520 |  |  |  |
|  | M-Health is difficult to understand, use and integrate in the hospital systems and operations(1) | .903 | 1.159 | .607 | 1 | .436 | 2.466 | .255 | 23.886 |
|  | M-Health is difficult to understand, use and integrate in the hospital systems and operations(2) | .378 | 1.167 | .105 | 1 | .746 | 1.459 | .148 | 14.368 |
|  | M-Health is difficult to understand, use and integrate in the hospital systems and operations(3) | 1.213 | 1.248 | .945 | 1 | .331 | 3.365 | .292 | 38.844 |
|  | Adoption of M-Health innovations requires exorbitant cost in infrastructure and human resources development |  |  | 1.056 | 3 | .788 |  |  |  |
|  | Adoption of M-Health innovations requires exorbitant cost in infrastructure and human resources development(1) | -.739 | .792 | .871 | 1 | .351 | .477 | .101 | 2.255 |
|  | Adoption of M-Health innovations requires exorbitant cost in infrastructure and human resources development(2) | -.160 | .666 | .058 | 1 | .810 | .852 | .231 | 3.143 |
|  | Adoption of M-Health innovations requires exorbitant cost in infrastructure and human resources development(3) | -.427 | .667 | .411 | 1 | .522 | .652 | .176 | 2.411 |
|  | M-Health needs to be piloted first in order to demonstrate that it is better than using current manual systems |  |  | 9.801 | 3 | .020 |  |  |  |
|  | M-Health needs to be piloted first in order to demonstrate that it is better than using current manual systems(1) | 2.633 | .889 | 8.773 | 1 | .003 | 13.921 | 2.437 | 79.517 |
|  | M-Health needs to be piloted first in order to demonstrate that it is better than using current manual systems(2) | 1.340 | .778 | 2.971 | 1 | .085 | 3.820 | .832 | 17.536 |
|  | M-Health needs to be piloted first in order to demonstrate that it is better than using current manual systems(3) | 1.228 | .581 | 4.460 | 1 | .035 | 3.413 | 1.092 | 10.665 |
|  | M-Health can be piloted without serious negative impact to patients and hospital operations |  |  | 3.255 | 3 | .354 |  |  |  |
|  | M-Health can be piloted without serious negative impact to patients and hospital operations(1) | 4.303 | 2.452 | 3.079 | 1 | .079 | 73.944 | .605 | 9042.586 |
|  | M-Health can be piloted without serious negative impact to patients and hospital operations(2) | .390 | .686 | .322 | 1 | .570 | 1.476 | .385 | 5.666 |
|  | M-Health can be piloted without serious negative impact to patients and hospital operations(3) | .190 | .509 | .140 | 1 | .709 | 1.210 | .446 | 3.281 |
|  | It is more strategic to adopt M-Health when the technology is leased to the hospital |  |  | 4.473 | 3 | .215 |  |  |  |
|  | It is more strategic to adopt M-Health when the technology is leased to the hospital(1) | 1.655 | .808 | 4.201 | 1 | .040 | 5.234 | 1.075 | 25.479 |
|  | It is more strategic to adopt M-Health when the technology is leased to the hospital(2) | .785 | .762 | 1.063 | 1 | .303 | 2.193 | .493 | 9.762 |
|  | It is more strategic to adopt M-Health when the technology is leased to the hospital(3) | .526 | .708 | .553 | 1 | .457 | 1.693 | .423 | 6.776 |
|  | It is more strategic to adopt M-Health when the hospital fully owned the technology |  |  | 1.649 | 3 | .648 |  |  |  |
|  | It is more strategic to adopt M-Health when the hospital fully owned the technology(1) | -.362 | 1.024 | .125 | 1 | .724 | .696 | .094 | 5.178 |
|  | It is more strategic to adopt M-Health when the hospital fully owned the technology(2) | .596 | .762 | .612 | 1 | .434 | 1.814 | .408 | 8.073 |
|  | It is more strategic to adopt M-Health when the hospital fully owned the technology(3) | .558 | .543 | 1.056 | 1 | .304 | 1.748 | .603 | 5.067 |
|  | Constant | -1.311 | 1.406 | .870 | 1 | .351 | .269 |  |  |
| a. Variable(s) entered on step 1: Use of M-health is superior and more advantageous than current manual practices., M-Health improves efficiency of hospital’s operations , M-health will reduce the cost of health care to patients, M-Health is compatible with current hospital’s health information system and consistent with its values and needs., M-Health can be trusted in terms of its security and confidentiality of patient information, M-Health will make many key staff redundant, M-Health is difficult to understand, use and integrate in the hospital systems and operations, Adoption of M-Health innovations requires exorbitant cost in infrastructure and human resources development, M-Health needs to be piloted first in order to demonstrate that it is better than using current manual systems, M-Health can be piloted without serious negative impact to patients and hospital operations, It is more strategic to adopt M-Health when the technology is leased to the hospital, It is more strategic to adopt M-Health when the hospital fully owned the technology. | | | | | | | | | |

**Organizational determinants of M-health adoption by hospitals in Kenya.**

1. **Health call centers/telephone help line adoption and organizational determinants**

**Logistic Regression**

| **Notes** | | |
| --- | --- | --- |
| Output Created | | 15-JUL-2018 14:24:51 |
| Comments | |  |
| Input | Data | C:\Users\Ocholap\Downloads\m-health data.- 13-JULY 2018.sav |
|  | Active Dataset | DataSet1 |
|  | Filter | <none> |
|  | Weight | <none> |
|  | Split File | <none> |
|  | N of Rows in Working Data File | 211 |
| Missing Value Handling | Definition of Missing | User-defined missing values are treated as missing |
| Syntax | | LOGISTIC REGRESSION VARIABLES Q20.1  /METHOD=ENTER Q24.1 Q24.2 Q24.3 Q24.4 Q24.5 Q24.6 Q24.7  /CONTRAST (Q24.1)=Indicator  /CONTRAST (Q24.2)=Indicator  /CONTRAST (Q24.3)=Indicator  /CONTRAST (Q24.4)=Indicator  /CONTRAST (Q24.5)=Indicator  /CONTRAST (Q24.6)=Indicator  /CONTRAST (Q24.7)=Indicator  /SAVE=PGROUP  /PRINT=GOODFIT SUMMARY CI(95)  /CRITERIA=PIN(0.05) POUT(0.10) ITERATE(20) CUT(0.5). |
| Resources | Processor Time | 00:00:00.03 |
|  | Elapsed Time | 00:00:00.05 |
| Variables Created or Modified | PGR_28 | Predicted group |

| **Case Processing Summary** | | | |
| --- | --- | --- | --- |
| Unweighted Cases^a^ | | N | Percent |
| Selected Cases | Included in Analysis | 203 | 96.2 |
|  | Missing Cases | 8 | 3.8 |
|  | Total | 211 | 100.0 |
| Unselected Cases | | 0 | .0 |
| Total | | 211 | 100.0 |
| a. If weight is in effect, see classification table for the total number of cases. | | | |

| **Dependent Variable Encoding** | |
| --- | --- |
| Original Value | Internal Value |
| Not adopted | 0 |
| Adopted | 1 |

| **Categorical Variables Codings** | | | | | |
| --- | --- | --- | --- | --- | --- |
|  | | Frequency | Parameter coding | | |
|  |  |  | (1) | (2) | (3) |
| Technology leadership: M-Health is appropriate for hospitals that pursue market growth through technology leadership | Strongly disgree | 29 | 1.000 | .000 | .000 |
|  | Disagree | 49 | .000 | 1.000 | .000 |
|  | Agree | 73 | .000 | .000 | 1.000 |
|  | Strongly Agree | 52 | .000 | .000 | .000 |
| Size: M-Health adoption is appropriate only when the hospital has substantial volume of patients and staff to justify its adoption | Strongly disgree | 58 | 1.000 | .000 | .000 |
|  | Disagree | 78 | .000 | 1.000 | .000 |
|  | Agree | 49 | .000 | .000 | 1.000 |
|  | Strongly Agree | 18 | .000 | .000 | .000 |
| ICT capacity: M-Health adoption is appropriate when hospitals have a very complex ICT infrastructure | Strongly disgree | 62 | 1.000 | .000 | .000 |
|  | Disagree | 92 | .000 | 1.000 | .000 |
|  | Agree | 26 | .000 | .000 | 1.000 |
|  | Strongly Agree | 23 | .000 | .000 | .000 |
| ICT staff : M-Health adoption is appropriate when hospitals have very knowledgeable and adequate number of staff in ICT | Strongly disgree | 26 | 1.000 | .000 | .000 |
|  | Disagree | 67 | .000 | 1.000 | .000 |
|  | Agree | 72 | .000 | .000 | 1.000 |
|  | Strongly Agree | 38 | .000 | .000 | .000 |
| Scope of the Market: M-Health adoption is appropriate for hospitals with larger market scopes such as national, regional or global markets. | Strongly disgree | 55 | 1.000 | .000 | .000 |
|  | Disagree | 86 | .000 | 1.000 | .000 |
|  | Agree | 37 | .000 | .000 | 1.000 |
|  | Strongly Agree | 25 | .000 | .000 | .000 |
| Slack/Financial Resources: M-health adoption is appropriate for hospitals that have excess budgets to invest in new IT technologies (soft and hard ware) such as M-Health | Strongly disgree | 72 | 1.000 | .000 | .000 |
|  | Disagree | 74 | .000 | 1.000 | .000 |
|  | Agree | 44 | .000 | .000 | 1.000 |
|  | Strongly Agree | 13 | .000 | .000 | .000 |
| Decision making structure: decision to adopt or not to adopt M-Health adoption is the prerogative of the hospital’s top management only. | Strongly disgree | 58 | 1.000 | .000 | .000 |
|  | Disagree | 71 | .000 | 1.000 | .000 |
|  | Agree | 44 | .000 | .000 | 1.000 |
|  | Strongly Agree | 30 | .000 | .000 | .000 |

**Block 0: Beginning Block**

| **Classification Table^a,b^** | | | | | |
| --- | --- | --- | --- | --- | --- |
|  | Observed | | Predicted | | |
|  |  |  | Health call centers/telephone help line | | Percentage Correct |
|  |  |  | Not adopted | Adopted |  |
| Step 0 | Health call centers/telephone help line | Not adopted | 0 | 36 | .0 |
|  |  | Adopted | 0 | 167 | 100.0 |
|  | Overall Percentage | |  |  | 82.3 |
| a. Constant is included in the model. | | | | | |
| b. The cut value is .500 | | | | | |

| **Variables in the Equation** | | | | | | | |
| --- | --- | --- | --- | --- | --- | --- | --- |
|  | | B | S.E. | Wald | df | Sig. | Exp(B) |
| Step 0 | Constant | 1.534 | .184 | 69.734 | 1 | .000 | 4.639 |

| **Variables not in the Equation** | | | | | |
| --- | --- | --- | --- | --- | --- |
|  | | | Score | df | Sig. |
| Step 0 | Variables | Decision making structure: decision to adopt or not to adopt M-Health adoption is the prerogative of the hospital’s top management only. | 4.689 | 3 | .196 |
|  |  | Decision making structure: decision to adopt or not to adopt M-Health adoption is the prerogative of the hospital’s top management only.(1) | .864 | 1 | .353 |
|  |  | Decision making structure: decision to adopt or not to adopt M-Health adoption is the prerogative of the hospital’s top management only.(2) | 4.344 | 1 | .037 |
|  |  | Decision making structure: decision to adopt or not to adopt M-Health adoption is the prerogative of the hospital’s top management only.(3) | 1.563 | 1 | .211 |
|  |  | Size: M-Health adoption is appropriate only when the hospital has substantial volume of patients and staff to justify its adoption | 2.048 | 3 | .562 |
|  |  | Size: M-Health adoption is appropriate only when the hospital has substantial volume of patients and staff to justify its adoption(1) | .864 | 1 | .353 |
|  |  | Size: M-Health adoption is appropriate only when the hospital has substantial volume of patients and staff to justify its adoption(2) | .195 | 1 | .659 |
|  |  | Size: M-Health adoption is appropriate only when the hospital has substantial volume of patients and staff to justify its adoption(3) | .088 | 1 | .767 |
|  |  | ICT capacity: M-Health adoption is appropriate when hospitals have a very complex ICT infrastructure | 4.057 | 3 | .255 |
|  |  | ICT capacity: M-Health adoption is appropriate when hospitals have a very complex ICT infrastructure(1) | 3.971 | 1 | .046 |
|  |  | ICT capacity: M-Health adoption is appropriate when hospitals have a very complex ICT infrastructure(2) | .982 | 1 | .322 |
|  |  | ICT capacity: M-Health adoption is appropriate when hospitals have a very complex ICT infrastructure(3) | .583 | 1 | .445 |
|  |  | ICT staff : M-Health adoption is appropriate when hospitals have very knowledgeable and adequate number of staff in ICT | 4.618 | 3 | .202 |
|  |  | ICT staff : M-Health adoption is appropriate when hospitals have very knowledgeable and adequate number of staff in ICT (1) | .785 | 1 | .376 |
|  |  | ICT staff : M-Health adoption is appropriate when hospitals have very knowledgeable and adequate number of staff in ICT (2) | 2.301 | 1 | .129 |
|  |  | ICT staff : M-Health adoption is appropriate when hospitals have very knowledgeable and adequate number of staff in ICT (3) | .735 | 1 | .391 |
|  |  | Scope of the Market: M-Health adoption is appropriate for hospitals with larger market scopes such as national, regional or global markets. | 4.753 | 3 | .191 |
|  |  | Scope of the Market: M-Health adoption is appropriate for hospitals with larger market scopes such as national, regional or global markets.(1) | 2.409 | 1 | .121 |
|  |  | Scope of the Market: M-Health adoption is appropriate for hospitals with larger market scopes such as national, regional or global markets.(2) | .423 | 1 | .515 |
|  |  | Scope of the Market: M-Health adoption is appropriate for hospitals with larger market scopes such as national, regional or global markets.(3) | 2.678 | 1 | .102 |
|  |  | Slack/Financial Resources: M-health adoption is appropriate for hospitals that have excess budgets to invest in new IT technologies (soft and hard ware) such as M-Health | 4.083 | 3 | .253 |
|  |  | Slack/Financial Resources: M-health adoption is appropriate for hospitals that have excess budgets to invest in new IT technologies (soft and hard ware) such as M-Health(1) | 3.354 | 1 | .067 |
|  |  | Slack/Financial Resources: M-health adoption is appropriate for hospitals that have excess budgets to invest in new IT technologies (soft and hard ware) such as M-Health(2) | .112 | 1 | .738 |
|  |  | Slack/Financial Resources: M-health adoption is appropriate for hospitals that have excess budgets to invest in new IT technologies (soft and hard ware) such as M-Health(3) | 2.033 | 1 | .154 |
|  |  | Technology leadership: M-Health is appropriate for hospitals that pursue market growth through technology leadership | 2.258 | 3 | .521 |
|  |  | Technology leadership: M-Health is appropriate for hospitals that pursue market growth through technology leadership (1) | .203 | 1 | .653 |
|  |  | Technology leadership: M-Health is appropriate for hospitals that pursue market growth through technology leadership (2) | 1.334 | 1 | .248 |
|  |  | Technology leadership: M-Health is appropriate for hospitals that pursue market growth through technology leadership (3) | 1.368 | 1 | .242 |
|  | Overall Statistics | | 19.382 | 21 | .561 |

**Block 1: Method = Enter**

| **Omnibus Tests of Model Coefficients** | | | | |
| --- | --- | --- | --- | --- |
|  | | Chi-square | df | Sig. |
| Step 1 | Step | 21.142 | 21 | .450 |
|  | Block | 21.142 | 21 | .450 |
|  | Model | 21.142 | 21 | .450 |

| **Model Summary** | | | |
| --- | --- | --- | --- |
| Step | -2 Log likelihood | Cox & Snell R Square | Nagelkerke R Square |
| 1 | 168.597^a^ | .099 | .163 |
| a. Estimation terminated at iteration number 5 because parameter estimates changed by less than .001. | | | |

| **Hosmer and Lemeshow Test** | | | |
| --- | --- | --- | --- |
| Step | Chi-square | df | Sig. |
| 1 | 5.151 | 8 | .741 |

| **Contingency Table for Hosmer and Lemeshow Test** | | | | | | |
| --- | --- | --- | --- | --- | --- | --- |
|  | | Health call centers/telephone help line = Not adopted | | Health call centers/telephone help line = Adopted | | Total |
|  |  | Observed | Expected | Observed | Expected |  |
| Step 1 | 1 | 8 | 8.682 | 12 | 11.318 | 20 |
|  | 2 | 7 | 6.258 | 12 | 12.742 | 19 |
|  | 3 | 5 | 5.111 | 15 | 14.889 | 20 |
|  | 4 | 4 | 4.160 | 16 | 15.840 | 20 |
|  | 5 | 5 | 3.286 | 15 | 16.714 | 20 |
|  | 6 | 1 | 2.762 | 19 | 17.238 | 20 |
|  | 7 | 2 | 2.226 | 18 | 17.774 | 20 |
|  | 8 | 3 | 1.666 | 17 | 18.334 | 20 |
|  | 9 | 0 | 1.147 | 20 | 18.853 | 20 |
|  | 10 | 1 | .701 | 23 | 23.299 | 24 |

| **Classification Table^a^** | | | | | |
| --- | --- | --- | --- | --- | --- |
|  | Observed | | Predicted | | |
|  |  |  | Health call centers/telephone help line | | Percentage Correct |
|  |  |  | Not adopted | Adopted |  |
| Step 1 | Health call centers/telephone help line | Not adopted | 1 | 35 | 2.8 |
|  |  | Adopted | 1 | 166 | 99.4 |
|  | Overall Percentage | |  |  | 82.3 |
| a. The cut value is .500 | | | | | |

| **Variables in the Equation** | | | | | | | | | |
| --- | --- | --- | --- | --- | --- | --- | --- | --- | --- |
|  | | B | S.E. | Wald | df | Sig. | Exp(B) | 95% C.I.for EXP(B) | |
|  |  |  |  |  |  |  |  | Lower | Upper |
| Step 1^a^ | Decision making structure: decision to adopt or not to adopt M-Health adoption is the prerogative of the hospital’s top management only. |  |  | 5.204 | 3 | .157 |  |  |  |
|  | Decision making structure: decision to adopt or not to adopt M-Health adoption is the prerogative of the hospital’s top management only.(1) | -.527 | .833 | .399 | 1 | .527 | .591 | .115 | 3.025 |
|  | Decision making structure: decision to adopt or not to adopt M-Health adoption is the prerogative of the hospital’s top management only.(2) | -1.048 | .731 | 2.055 | 1 | .152 | .351 | .084 | 1.469 |
|  | Decision making structure: decision to adopt or not to adopt M-Health adoption is the prerogative of the hospital’s top management only.(3) | .189 | .841 | .050 | 1 | .822 | 1.208 | .232 | 6.273 |
|  | Size: M-Health adoption is appropriate only when the hospital has substantial volume of patients and staff to justify its adoption |  |  | 1.687 | 3 | .640 |  |  |  |
|  | Size: M-Health adoption is appropriate only when the hospital has substantial volume of patients and staff to justify its adoption(1) | .704 | .886 | .632 | 1 | .426 | 2.022 | .356 | 11.473 |
|  | Size: M-Health adoption is appropriate only when the hospital has substantial volume of patients and staff to justify its adoption(2) | .864 | .866 | .995 | 1 | .319 | 2.372 | .434 | 12.951 |
|  | Size: M-Health adoption is appropriate only when the hospital has substantial volume of patients and staff to justify its adoption(3) | 1.072 | .831 | 1.666 | 1 | .197 | 2.922 | .574 | 14.892 |
|  | ICT capacity: M-Health adoption is appropriate when hospitals have a very complex ICT infrastructure |  |  | 2.046 | 3 | .563 |  |  |  |
|  | ICT capacity: M-Health adoption is appropriate when hospitals have a very complex ICT infrastructure(1) | .409 | .937 | .191 | 1 | .662 | 1.505 | .240 | 9.452 |
|  | ICT capacity: M-Health adoption is appropriate when hospitals have a very complex ICT infrastructure(2) | -.430 | .857 | .252 | 1 | .616 | .651 | .121 | 3.492 |
|  | ICT capacity: M-Health adoption is appropriate when hospitals have a very complex ICT infrastructure(3) | -.257 | .889 | .083 | 1 | .773 | .773 | .135 | 4.419 |
|  | ICT staff : M-Health adoption is appropriate when hospitals have very knowledgeable and adequate number of staff in ICT |  |  | 2.728 | 3 | .436 |  |  |  |
|  | ICT staff : M-Health adoption is appropriate when hospitals have very knowledgeable and adequate number of staff in ICT (1) | .875 | .926 | .893 | 1 | .345 | 2.399 | .391 | 14.739 |
|  | ICT staff : M-Health adoption is appropriate when hospitals have very knowledgeable and adequate number of staff in ICT (2) | 1.161 | .713 | 2.654 | 1 | .103 | 3.194 | .790 | 12.914 |
|  | ICT staff : M-Health adoption is appropriate when hospitals have very knowledgeable and adequate number of staff in ICT (3) | .682 | .635 | 1.151 | 1 | .283 | 1.977 | .569 | 6.867 |
|  | Scope of the Market: M-Health adoption is appropriate for hospitals with larger market scopes such as national, regional or global markets. |  |  | .907 | 3 | .824 |  |  |  |
|  | Scope of the Market: M-Health adoption is appropriate for hospitals with larger market scopes such as national, regional or global markets.(1) | -.094 | .984 | .009 | 1 | .924 | .910 | .132 | 6.266 |
|  | Scope of the Market: M-Health adoption is appropriate for hospitals with larger market scopes such as national, regional or global markets.(2) | -.450 | .880 | .261 | 1 | .609 | .638 | .114 | 3.580 |
|  | Scope of the Market: M-Health adoption is appropriate for hospitals with larger market scopes such as national, regional or global markets.(3) | -.673 | .871 | .596 | 1 | .440 | .510 | .093 | 2.815 |
|  | Slack/Financial Resources: M-health adoption is appropriate for hospitals that have excess budgets to invest in new IT technologies (soft and hard ware) such as M-Health |  |  | 2.137 | 3 | .545 |  |  |  |
|  | Slack/Financial Resources: M-health adoption is appropriate for hospitals that have excess budgets to invest in new IT technologies (soft and hard ware) such as M-Health(1) | .890 | .925 | .927 | 1 | .336 | 2.436 | .398 | 14.920 |
|  | Slack/Financial Resources: M-health adoption is appropriate for hospitals that have excess budgets to invest in new IT technologies (soft and hard ware) such as M-Health(2) | .077 | .879 | .008 | 1 | .930 | 1.080 | .193 | 6.047 |
|  | Slack/Financial Resources: M-health adoption is appropriate for hospitals that have excess budgets to invest in new IT technologies (soft and hard ware) such as M-Health(3) | .089 | .850 | .011 | 1 | .917 | 1.093 | .207 | 5.783 |
|  | Technology leadership: M-Health is appropriate for hospitals that pursue market growth through technology leadership |  |  | 2.817 | 3 | .421 |  |  |  |
|  | Technology leadership: M-Health is appropriate for hospitals that pursue market growth through technology leadership (1) | -1.135 | .793 | 2.046 | 1 | .153 | .322 | .068 | 1.522 |
|  | Technology leadership: M-Health is appropriate for hospitals that pursue market growth through technology leadership (2) | -.037 | .756 | .002 | 1 | .961 | .964 | .219 | 4.243 |
|  | Technology leadership: M-Health is appropriate for hospitals that pursue market growth through technology leadership (3) | -.427 | .645 | .439 | 1 | .508 | .652 | .184 | 2.308 |
|  | Constant | 1.160 | .938 | 1.528 | 1 | .216 | 3.189 |  |  |
| a. Variable(s) entered on step 1: Decision making structure: decision to adopt or not to adopt M-Health adoption is the prerogative of the hospital’s top management only., Size: M-Health adoption is appropriate only when the hospital has substantial volume of patients and staff to justify its adoption, ICT capacity: M-Health adoption is appropriate when hospitals have a very complex ICT infrastructure, ICT staff : M-Health adoption is appropriate when hospitals have very knowledgeable and adequate number of staff in ICT , Scope of the Market: M-Health adoption is appropriate for hospitals with larger market scopes such as national, regional or global markets., Slack/Financial Resources: M-health adoption is appropriate for hospitals that have excess budgets to invest in new IT technologies (soft and hard ware) such as M-Health, Technology leadership: M-Health is appropriate for hospitals that pursue market growth through technology leadership . | | | | | | | | | |

1. **Emergency toll-free telephone services adoption and organizational determinants**

**Logistic Regression**

| **Notes** | | |
| --- | --- | --- |
| Output Created | | 15-JUL-2018 14:37:06 |
| Comments | |  |
| Input | Data | C:\Users\Ocholap\Downloads\m-health data.- 13-JULY 2018.sav |
|  | Active Dataset | DataSet1 |
|  | Filter | <none> |
|  | Weight | <none> |
|  | Split File | <none> |
|  | N of Rows in Working Data File | 211 |
| Missing Value Handling | Definition of Missing | User-defined missing values are treated as missing |
| Syntax | | LOGISTIC REGRESSION VARIABLES Q20.2  /METHOD=ENTER Q24.1 Q24.2 Q24.3 Q24.4 Q24.5 Q24.6 Q24.7  /CONTRAST (Q24.1)=Indicator  /CONTRAST (Q24.2)=Indicator  /CONTRAST (Q24.3)=Indicator  /CONTRAST (Q24.4)=Indicator  /CONTRAST (Q24.5)=Indicator  /CONTRAST (Q24.6)=Indicator  /CONTRAST (Q24.7)=Indicator  /SAVE=PGROUP  /PRINT=GOODFIT SUMMARY CI(95)  /CRITERIA=PIN(0.05) POUT(0.10) ITERATE(20) CUT(0.5). |
| Resources | Processor Time | 00:00:00.03 |
|  | Elapsed Time | 00:00:00.03 |
| Variables Created or Modified | PGR_29 | Predicted group |

| **Case Processing Summary** | | | |
| --- | --- | --- | --- |
| Unweighted Cases^a^ | | N | Percent |
| Selected Cases | Included in Analysis | 192 | 91.0 |
|  | Missing Cases | 19 | 9.0 |
|  | Total | 211 | 100.0 |
| Unselected Cases | | 0 | .0 |
| Total | | 211 | 100.0 |
| a. If weight is in effect, see classification table for the total number of cases. | | | |

| **Dependent Variable Encoding** | |
| --- | --- |
| Original Value | Internal Value |
| Not adopted | 0 |
| Adopted | 1 |

| **Categorical Variables Codings** | | | | | |
| --- | --- | --- | --- | --- | --- |
|  | | Frequency | Parameter coding | | |
|  |  |  | (1) | (2) | (3) |
| Technology leadership: M-Health is appropriate for hospitals that pursue market growth through technology leadership | Strongly disgree | 26 | 1.000 | .000 | .000 |
|  | Disagree | 46 | .000 | 1.000 | .000 |
|  | Agree | 71 | .000 | .000 | 1.000 |
|  | Strongly Agree | 49 | .000 | .000 | .000 |
| Size: M-Health adoption is appropriate only when the hospital has substantial volume of patients and staff to justify its adoption | Strongly disgree | 52 | 1.000 | .000 | .000 |
|  | Disagree | 74 | .000 | 1.000 | .000 |
|  | Agree | 48 | .000 | .000 | 1.000 |
|  | Strongly Agree | 18 | .000 | .000 | .000 |
| ICT capacity: M-Health adoption is appropriate when hospitals have a very complex ICT infrastructure | Strongly disgree | 57 | 1.000 | .000 | .000 |
|  | Disagree | 86 | .000 | 1.000 | .000 |
|  | Agree | 26 | .000 | .000 | 1.000 |
|  | Strongly Agree | 23 | .000 | .000 | .000 |
| ICT staff : M-Health adoption is appropriate when hospitals have very knowledgeable and adequate number of staff in ICT | Strongly disgree | 24 | 1.000 | .000 | .000 |
|  | Disagree | 59 | .000 | 1.000 | .000 |
|  | Agree | 72 | .000 | .000 | 1.000 |
|  | Strongly Agree | 37 | .000 | .000 | .000 |
| Scope of the Market: M-Health adoption is appropriate for hospitals with larger market scopes such as national, regional or global markets. | Strongly disgree | 50 | 1.000 | .000 | .000 |
|  | Disagree | 79 | .000 | 1.000 | .000 |
|  | Agree | 37 | .000 | .000 | 1.000 |
|  | Strongly Agree | 26 | .000 | .000 | .000 |
| Slack/Financial Resources: M-health adoption is appropriate for hospitals that have excess budgets to invest in new IT technologies (soft and hard ware) such as M-Health | Strongly disgree | 66 | 1.000 | .000 | .000 |
|  | Disagree | 69 | .000 | 1.000 | .000 |
|  | Agree | 43 | .000 | .000 | 1.000 |
|  | Strongly Agree | 14 | .000 | .000 | .000 |
| Decision making structure: decision to adopt or not to adopt M-Health adoption is the prerogative of the hospital’s top management only. | Strongly disgree | 50 | 1.000 | .000 | .000 |
|  | Disagree | 71 | .000 | 1.000 | .000 |
|  | Agree | 42 | .000 | .000 | 1.000 |
|  | Strongly Agree | 29 | .000 | .000 | .000 |

**Block 0: Beginning Block**

| **Classification Table^a,b^** | | | | | |
| --- | --- | --- | --- | --- | --- |
|  | Observed | | Predicted | | |
|  |  |  | Emergency toll-free telephone services | | Percentage Correct |
|  |  |  | Not adopted | Adopted |  |
| Step 0 | Emergency toll-free telephone services | Not adopted | 137 | 0 | 100.0 |
|  |  | Adopted | 55 | 0 | .0 |
|  | Overall Percentage | |  |  | 71.4 |
| a. Constant is included in the model. | | | | | |
| b. The cut value is .500 | | | | | |

| **Variables in the Equation** | | | | | | | |
| --- | --- | --- | --- | --- | --- | --- | --- |
|  | | B | S.E. | Wald | df | Sig. | Exp(B) |
| Step 0 | Constant | -.913 | .160 | 32.688 | 1 | .000 | .401 |

| **Variables not in the Equation** | | | | | |
| --- | --- | --- | --- | --- | --- |
|  | | | Score | df | Sig. |
| Step 0 | Variables | Decision making structure: decision to adopt or not to adopt M-Health adoption is the prerogative of the hospital’s top management only. | 2.399 | 3 | .494 |
|  |  | Decision making structure: decision to adopt or not to adopt M-Health adoption is the prerogative of the hospital’s top management only.(1) | .232 | 1 | .630 |
|  |  | Decision making structure: decision to adopt or not to adopt M-Health adoption is the prerogative of the hospital’s top management only.(2) | .598 | 1 | .439 |
|  |  | Decision making structure: decision to adopt or not to adopt M-Health adoption is the prerogative of the hospital’s top management only.(3) | 2.348 | 1 | .125 |
|  |  | Size: M-Health adoption is appropriate only when the hospital has substantial volume of patients and staff to justify its adoption | 4.468 | 3 | .215 |
|  |  | Size: M-Health adoption is appropriate only when the hospital has substantial volume of patients and staff to justify its adoption(1) | .104 | 1 | .748 |
|  |  | Size: M-Health adoption is appropriate only when the hospital has substantial volume of patients and staff to justify its adoption(2) | .520 | 1 | .471 |
|  |  | Size: M-Health adoption is appropriate only when the hospital has substantial volume of patients and staff to justify its adoption(3) | .076 | 1 | .782 |
|  |  | ICT capacity: M-Health adoption is appropriate when hospitals have a very complex ICT infrastructure | 3.294 | 3 | .348 |
|  |  | ICT capacity: M-Health adoption is appropriate when hospitals have a very complex ICT infrastructure(1) | .013 | 1 | .909 |
|  |  | ICT capacity: M-Health adoption is appropriate when hospitals have a very complex ICT infrastructure(2) | 1.362 | 1 | .243 |
|  |  | ICT capacity: M-Health adoption is appropriate when hospitals have a very complex ICT infrastructure(3) | .066 | 1 | .797 |
|  |  | ICT staff : M-Health adoption is appropriate when hospitals have very knowledgeable and adequate number of staff in ICT | 4.462 | 3 | .216 |
|  |  | ICT staff : M-Health adoption is appropriate when hospitals have very knowledgeable and adequate number of staff in ICT (1) | .178 | 1 | .673 |
|  |  | ICT staff : M-Health adoption is appropriate when hospitals have very knowledgeable and adequate number of staff in ICT (2) | 2.011 | 1 | .156 |
|  |  | ICT staff : M-Health adoption is appropriate when hospitals have very knowledgeable and adequate number of staff in ICT (3) | 3.440 | 1 | .064 |
|  |  | Scope of the Market: M-Health adoption is appropriate for hospitals with larger market scopes such as national, regional or global markets. | 5.759 | 3 | .124 |
|  |  | Scope of the Market: M-Health adoption is appropriate for hospitals with larger market scopes such as national, regional or global markets.(1) | .714 | 1 | .398 |
|  |  | Scope of the Market: M-Health adoption is appropriate for hospitals with larger market scopes such as national, regional or global markets.(2) | 2.256 | 1 | .133 |
|  |  | Scope of the Market: M-Health adoption is appropriate for hospitals with larger market scopes such as national, regional or global markets.(3) | 1.895 | 1 | .169 |
|  |  | Slack/Financial Resources: M-health adoption is appropriate for hospitals that have excess budgets to invest in new IT technologies (soft and hard ware) such as M-Health | 6.071 | 3 | .108 |
|  |  | Slack/Financial Resources: M-health adoption is appropriate for hospitals that have excess budgets to invest in new IT technologies (soft and hard ware) such as M-Health(1) | .410 | 1 | .522 |
|  |  | Slack/Financial Resources: M-health adoption is appropriate for hospitals that have excess budgets to invest in new IT technologies (soft and hard ware) such as M-Health(2) | .065 | 1 | .799 |
|  |  | Slack/Financial Resources: M-health adoption is appropriate for hospitals that have excess budgets to invest in new IT technologies (soft and hard ware) such as M-Health(3) | .255 | 1 | .614 |
|  |  | Technology leadership: M-Health is appropriate for hospitals that pursue market growth through technology leadership | 1.806 | 3 | .614 |
|  |  | Technology leadership: M-Health is appropriate for hospitals that pursue market growth through technology leadership (1) | .456 | 1 | .499 |
|  |  | Technology leadership: M-Health is appropriate for hospitals that pursue market growth through technology leadership (2) | .663 | 1 | .416 |
|  |  | Technology leadership: M-Health is appropriate for hospitals that pursue market growth through technology leadership (3) | .048 | 1 | .827 |
|  | Overall Statistics | | 26.071 | 21 | .204 |

**Block 1: Method = Enter**

| **Omnibus Tests of Model Coefficients** | | | | |
| --- | --- | --- | --- | --- |
|  | | Chi-square | df | Sig. |
| Step 1 | Step | 27.264 | 21 | .162 |
|  | Block | 27.264 | 21 | .162 |
|  | Model | 27.264 | 21 | .162 |

| **Model Summary** | | | |
| --- | --- | --- | --- |
| Step | -2 Log likelihood | Cox & Snell R Square | Nagelkerke R Square |
| 1 | 202.733^a^ | .132 | .190 |
| a. Estimation terminated at iteration number 5 because parameter estimates changed by less than .001. | | | |

| **Hosmer and Lemeshow Test** | | | |
| --- | --- | --- | --- |
| Step | Chi-square | df | Sig. |
| 1 | 10.792 | 8 | .214 |

| **Contingency Table for Hosmer and Lemeshow Test** | | | | | | |
| --- | --- | --- | --- | --- | --- | --- |
|  | | Emergency toll-free telephone services = Not adopted | | Emergency toll-free telephone services = Adopted | | Total |
|  |  | Observed | Expected | Observed | Expected |  |
| Step 1 | 1 | 19 | 17.658 | 0 | 1.342 | 19 |
|  | 2 | 15 | 16.945 | 4 | 2.055 | 19 |
|  | 3 | 18 | 16.146 | 1 | 2.854 | 19 |
|  | 4 | 13 | 15.300 | 6 | 3.700 | 19 |
|  | 5 | 14 | 15.269 | 6 | 4.731 | 20 |
|  | 6 | 14 | 13.520 | 5 | 5.480 | 19 |
|  | 7 | 13 | 12.844 | 6 | 6.156 | 19 |
|  | 8 | 11 | 11.850 | 8 | 7.150 | 19 |
|  | 9 | 14 | 10.193 | 5 | 8.807 | 19 |
|  | 10 | 6 | 7.274 | 14 | 12.726 | 20 |

| **Classification Table^a^** | | | | | |
| --- | --- | --- | --- | --- | --- |
|  | Observed | | Predicted | | |
|  |  |  | Emergency toll-free telephone services | | Percentage Correct |
|  |  |  | Not adopted | Adopted |  |
| Step 1 | Emergency toll-free telephone services | Not adopted | 129 | 8 | 94.2 |
|  |  | Adopted | 40 | 15 | 27.3 |
|  | Overall Percentage | |  |  | 75.0 |
| a. The cut value is .500 | | | | | |

| **Variables in the Equation** | | | | | | | | | |
| --- | --- | --- | --- | --- | --- | --- | --- | --- | --- |
|  | | B | S.E. | Wald | df | Sig. | Exp(B) | 95% C.I.for EXP(B) | |
|  |  |  |  |  |  |  |  | Lower | Upper |
| Step 1^a^ | Decision making structure: decision to adopt or not to adopt M-Health adoption is the prerogative of the hospital’s top management only. |  |  | 4.663 | 3 | .198 |  |  |  |
|  | Decision making structure: decision to adopt or not to adopt M-Health adoption is the prerogative of the hospital’s top management only.(1) | .545 | .706 | .596 | 1 | .440 | 1.724 | .432 | 6.875 |
|  | Decision making structure: decision to adopt or not to adopt M-Health adoption is the prerogative of the hospital’s top management only.(2) | .759 | .653 | 1.354 | 1 | .245 | 2.137 | .595 | 7.681 |
|  | Decision making structure: decision to adopt or not to adopt M-Health adoption is the prerogative of the hospital’s top management only.(3) | 1.410 | .693 | 4.141 | 1 | .042 | 4.095 | 1.053 | 15.922 |
|  | Size: M-Health adoption is appropriate only when the hospital has substantial volume of patients and staff to justify its adoption |  |  | 3.815 | 3 | .282 |  |  |  |
|  | Size: M-Health adoption is appropriate only when the hospital has substantial volume of patients and staff to justify its adoption(1) | -.872 | .743 | 1.377 | 1 | .241 | .418 | .098 | 1.794 |
|  | Size: M-Health adoption is appropriate only when the hospital has substantial volume of patients and staff to justify its adoption(2) | -.586 | .742 | .622 | 1 | .430 | .557 | .130 | 2.385 |
|  | Size: M-Health adoption is appropriate only when the hospital has substantial volume of patients and staff to justify its adoption(3) | -1.314 | .731 | 3.228 | 1 | .072 | .269 | .064 | 1.127 |
|  | ICT capacity: M-Health adoption is appropriate when hospitals have a very complex ICT infrastructure |  |  | 1.748 | 3 | .626 |  |  |  |
|  | ICT capacity: M-Health adoption is appropriate when hospitals have a very complex ICT infrastructure(1) | -.643 | .808 | .633 | 1 | .426 | .526 | .108 | 2.562 |
|  | ICT capacity: M-Health adoption is appropriate when hospitals have a very complex ICT infrastructure(2) | -.956 | .774 | 1.528 | 1 | .216 | .384 | .084 | 1.750 |
|  | ICT capacity: M-Health adoption is appropriate when hospitals have a very complex ICT infrastructure(3) | -.462 | .800 | .334 | 1 | .564 | .630 | .131 | 3.022 |
|  | ICT staff : M-Health adoption is appropriate when hospitals have very knowledgeable and adequate number of staff in ICT |  |  | 10.567 | 3 | .014 |  |  |  |
|  | ICT staff : M-Health adoption is appropriate when hospitals have very knowledgeable and adequate number of staff in ICT (1) | .328 | .813 | .163 | 1 | .686 | 1.389 | .282 | 6.828 |
|  | ICT staff : M-Health adoption is appropriate when hospitals have very knowledgeable and adequate number of staff in ICT (2) | 1.138 | .670 | 2.887 | 1 | .089 | 3.122 | .840 | 11.607 |
|  | ICT staff : M-Health adoption is appropriate when hospitals have very knowledgeable and adequate number of staff in ICT (3) | -.591 | .640 | .851 | 1 | .356 | .554 | .158 | 1.944 |
|  | Scope of the Market: M-Health adoption is appropriate for hospitals with larger market scopes such as national, regional or global markets. |  |  | 5.602 | 3 | .133 |  |  |  |
|  | Scope of the Market: M-Health adoption is appropriate for hospitals with larger market scopes such as national, regional or global markets.(1) | -.620 | .739 | .704 | 1 | .401 | .538 | .126 | 2.289 |
|  | Scope of the Market: M-Health adoption is appropriate for hospitals with larger market scopes such as national, regional or global markets.(2) | -.852 | .675 | 1.595 | 1 | .207 | .426 | .114 | 1.601 |
|  | Scope of the Market: M-Health adoption is appropriate for hospitals with larger market scopes such as national, regional or global markets.(3) | .512 | .689 | .551 | 1 | .458 | 1.668 | .432 | 6.441 |
|  | Slack/Financial Resources: M-health adoption is appropriate for hospitals that have excess budgets to invest in new IT technologies (soft and hard ware) such as M-Health |  |  | 3.136 | 3 | .371 |  |  |  |
|  | Slack/Financial Resources: M-health adoption is appropriate for hospitals that have excess budgets to invest in new IT technologies (soft and hard ware) such as M-Health(1) | -1.096 | .775 | 2.001 | 1 | .157 | .334 | .073 | 1.526 |
|  | Slack/Financial Resources: M-health adoption is appropriate for hospitals that have excess budgets to invest in new IT technologies (soft and hard ware) such as M-Health(2) | -.943 | .766 | 1.513 | 1 | .219 | .390 | .087 | 1.749 |
|  | Slack/Financial Resources: M-health adoption is appropriate for hospitals that have excess budgets to invest in new IT technologies (soft and hard ware) such as M-Health(3) | -1.270 | .740 | 2.941 | 1 | .086 | .281 | .066 | 1.199 |
|  | Technology leadership: M-Health is appropriate for hospitals that pursue market growth through technology leadership |  |  | .516 | 3 | .915 |  |  |  |
|  | Technology leadership: M-Health is appropriate for hospitals that pursue market growth through technology leadership (1) | .119 | .701 | .029 | 1 | .865 | 1.127 | .285 | 4.448 |
|  | Technology leadership: M-Health is appropriate for hospitals that pursue market growth through technology leadership (2) | -.208 | .610 | .116 | 1 | .733 | .812 | .246 | 2.683 |
|  | Technology leadership: M-Health is appropriate for hospitals that pursue market growth through technology leadership (3) | .140 | .553 | .064 | 1 | .800 | 1.150 | .389 | 3.397 |
|  | Constant | .899 | .782 | 1.322 | 1 | .250 | 2.458 |  |  |
| a. Variable(s) entered on step 1: Decision making structure: decision to adopt or not to adopt M-Health adoption is the prerogative of the hospital’s top management only., Size: M-Health adoption is appropriate only when the hospital has substantial volume of patients and staff to justify its adoption, ICT capacity: M-Health adoption is appropriate when hospitals have a very complex ICT infrastructure, ICT staff : M-Health adoption is appropriate when hospitals have very knowledgeable and adequate number of staff in ICT , Scope of the Market: M-Health adoption is appropriate for hospitals with larger market scopes such as national, regional or global markets., Slack/Financial Resources: M-health adoption is appropriate for hospitals that have excess budgets to invest in new IT technologies (soft and hard ware) such as M-Health, Technology leadership: M-Health is appropriate for hospitals that pursue market growth through technology leadership . | | | | | | | | | |

1. **Treatment compliance technology Adoption and organizational determinants**

**Logistic Regression**

| **Notes** | | |
| --- | --- | --- |
| Output Created | | 15-JUL-2018 14:48:15 |
| Comments | |  |
| Input | Data | C:\Users\Ocholap\Downloads\m-health data.- 13-JULY 2018.sav |
|  | Active Dataset | DataSet1 |
|  | Filter | <none> |
|  | Weight | <none> |
|  | Split File | <none> |
|  | N of Rows in Working Data File | 211 |
| Missing Value Handling | Definition of Missing | User-defined missing values are treated as missing |
| Syntax | | LOGISTIC REGRESSION VARIABLES Q20.3  /METHOD=ENTER Q24.1 Q24.2 Q24.3 Q24.4 Q24.5 Q24.6 Q24.7  /CONTRAST (Q24.1)=Indicator  /CONTRAST (Q24.2)=Indicator  /CONTRAST (Q24.3)=Indicator  /CONTRAST (Q24.4)=Indicator  /CONTRAST (Q24.5)=Indicator  /CONTRAST (Q24.6)=Indicator  /CONTRAST (Q24.7)=Indicator  /SAVE=PGROUP  /PRINT=GOODFIT SUMMARY CI(95)  /CRITERIA=PIN(0.05) POUT(0.10) ITERATE(20) CUT(0.5). |
| Resources | Processor Time | 00:00:00.05 |
|  | Elapsed Time | 00:00:00.05 |
| Variables Created or Modified | PGR_30 | Predicted group |

| **Case Processing Summary** | | | |
| --- | --- | --- | --- |
| Unweighted Cases^a^ | | N | Percent |
| Selected Cases | Included in Analysis | 199 | 94.3 |
|  | Missing Cases | 12 | 5.7 |
|  | Total | 211 | 100.0 |
| Unselected Cases | | 0 | .0 |
| Total | | 211 | 100.0 |
| a. If weight is in effect, see classification table for the total number of cases. | | | |

| **Dependent Variable Encoding** | |
| --- | --- |
| Original Value | Internal Value |
| Not adopted | 0 |
| Adopted | 1 |

| **Categorical Variables Codings** | | | | | |
| --- | --- | --- | --- | --- | --- |
|  | | Frequency | Parameter coding | | |
|  |  |  | (1) | (2) | (3) |
| Technology leadership: M-Health is appropriate for hospitals that pursue market growth through technology leadership | Strongly disgree | 25 | 1.000 | .000 | .000 |
|  | Disagree | 48 | .000 | 1.000 | .000 |
|  | Agree | 73 | .000 | .000 | 1.000 |
|  | Strongly Agree | 53 | .000 | .000 | .000 |
| Size: M-Health adoption is appropriate only when the hospital has substantial volume of patients and staff to justify its adoption | Strongly disgree | 56 | 1.000 | .000 | .000 |
|  | Disagree | 78 | .000 | 1.000 | .000 |
|  | Agree | 46 | .000 | .000 | 1.000 |
|  | Strongly Agree | 19 | .000 | .000 | .000 |
| ICT capacity: M-Health adoption is appropriate when hospitals have a very complex ICT infrastructure | Strongly disgree | 60 | 1.000 | .000 | .000 |
|  | Disagree | 89 | .000 | 1.000 | .000 |
|  | Agree | 26 | .000 | .000 | 1.000 |
|  | Strongly Agree | 24 | .000 | .000 | .000 |
| ICT staff : M-Health adoption is appropriate when hospitals have very knowledgeable and adequate number of staff in ICT | Strongly disgree | 23 | 1.000 | .000 | .000 |
|  | Disagree | 67 | .000 | 1.000 | .000 |
|  | Agree | 70 | .000 | .000 | 1.000 |
|  | Strongly Agree | 39 | .000 | .000 | .000 |
| Scope of the Market: M-Health adoption is appropriate for hospitals with larger market scopes such as national, regional or global markets. | Strongly disgree | 52 | 1.000 | .000 | .000 |
|  | Disagree | 87 | .000 | 1.000 | .000 |
|  | Agree | 34 | .000 | .000 | 1.000 |
|  | Strongly Agree | 26 | .000 | .000 | .000 |
| Slack/Financial Resources: M-health adoption is appropriate for hospitals that have excess budgets to invest in new IT technologies (soft and hard ware) such as M-Health | Strongly disgree | 71 | 1.000 | .000 | .000 |
|  | Disagree | 73 | .000 | 1.000 | .000 |
|  | Agree | 41 | .000 | .000 | 1.000 |
|  | Strongly Agree | 14 | .000 | .000 | .000 |
| Decision making structure: decision to adopt or not to adopt M-Health adoption is the prerogative of the hospital’s top management only. | Strongly disgree | 55 | 1.000 | .000 | .000 |
|  | Disagree | 72 | .000 | 1.000 | .000 |
|  | Agree | 43 | .000 | .000 | 1.000 |
|  | Strongly Agree | 29 | .000 | .000 | .000 |

**Block 0: Beginning Block**

| **Classification Table^a,b^** | | | | | |
| --- | --- | --- | --- | --- | --- |
|  | Observed | | Predicted | | |
|  |  |  | Treatment compliance | | Percentage Correct |
|  |  |  | Not adopted | Adopted |  |
| Step 0 | Treatment compliance | Not adopted | 0 | 57 | .0 |
|  |  | Adopted | 0 | 142 | 100.0 |
|  | Overall Percentage | |  |  | 71.4 |
| a. Constant is included in the model. | | | | | |
| b. The cut value is .500 | | | | | |

| **Variables in the Equation** | | | | | | | |
| --- | --- | --- | --- | --- | --- | --- | --- |
|  | | B | S.E. | Wald | df | Sig. | Exp(B) |
| Step 0 | Constant | .913 | .157 | 33.887 | 1 | .000 | 2.491 |

| **Variables not in the Equation** | | | | | |
| --- | --- | --- | --- | --- | --- |
|  | | | Score | df | Sig. |
| Step 0 | Variables | Decision making structure: decision to adopt or not to adopt M-Health adoption is the prerogative of the hospital’s top management only. | 1.978 | 3 | .577 |
|  |  | Decision making structure: decision to adopt or not to adopt M-Health adoption is the prerogative of the hospital’s top management only.(1) | .932 | 1 | .334 |
|  |  | Decision making structure: decision to adopt or not to adopt M-Health adoption is the prerogative of the hospital’s top management only.(2) | .041 | 1 | .839 |
|  |  | Decision making structure: decision to adopt or not to adopt M-Health adoption is the prerogative of the hospital’s top management only.(3) | .068 | 1 | .795 |
|  |  | Size: M-Health adoption is appropriate only when the hospital has substantial volume of patients and staff to justify its adoption | 1.270 | 3 | .736 |
|  |  | Size: M-Health adoption is appropriate only when the hospital has substantial volume of patients and staff to justify its adoption(1) | .506 | 1 | .477 |
|  |  | Size: M-Health adoption is appropriate only when the hospital has substantial volume of patients and staff to justify its adoption(2) | .012 | 1 | .913 |
|  |  | Size: M-Health adoption is appropriate only when the hospital has substantial volume of patients and staff to justify its adoption(3) | 1.103 | 1 | .294 |
|  |  | ICT capacity: M-Health adoption is appropriate when hospitals have a very complex ICT infrastructure | 1.341 | 3 | .719 |
|  |  | ICT capacity: M-Health adoption is appropriate when hospitals have a very complex ICT infrastructure(1) | .164 | 1 | .685 |
|  |  | ICT capacity: M-Health adoption is appropriate when hospitals have a very complex ICT infrastructure(2) | 1.224 | 1 | .269 |
|  |  | ICT capacity: M-Health adoption is appropriate when hospitals have a very complex ICT infrastructure(3) | .453 | 1 | .501 |
|  |  | ICT staff : M-Health adoption is appropriate when hospitals have very knowledgeable and adequate number of staff in ICT | .978 | 3 | .807 |
|  |  | ICT staff : M-Health adoption is appropriate when hospitals have very knowledgeable and adequate number of staff in ICT (1) | .083 | 1 | .773 |
|  |  | ICT staff : M-Health adoption is appropriate when hospitals have very knowledgeable and adequate number of staff in ICT (2) | .072 | 1 | .788 |
|  |  | ICT staff : M-Health adoption is appropriate when hospitals have very knowledgeable and adequate number of staff in ICT (3) | .410 | 1 | .522 |
|  |  | Scope of the Market: M-Health adoption is appropriate for hospitals with larger market scopes such as national, regional or global markets. | 5.689 | 3 | .128 |
|  |  | Scope of the Market: M-Health adoption is appropriate for hospitals with larger market scopes such as national, regional or global markets.(1) | 3.051 | 1 | .081 |
|  |  | Scope of the Market: M-Health adoption is appropriate for hospitals with larger market scopes such as national, regional or global markets.(2) | 3.694 | 1 | .055 |
|  |  | Scope of the Market: M-Health adoption is appropriate for hospitals with larger market scopes such as national, regional or global markets.(3) | .276 | 1 | .599 |
|  |  | Slack/Financial Resources: M-health adoption is appropriate for hospitals that have excess budgets to invest in new IT technologies (soft and hard ware) such as M-Health | .664 | 3 | .882 |
|  |  | Slack/Financial Resources: M-health adoption is appropriate for hospitals that have excess budgets to invest in new IT technologies (soft and hard ware) such as M-Health(1) | .012 | 1 | .912 |
|  |  | Slack/Financial Resources: M-health adoption is appropriate for hospitals that have excess budgets to invest in new IT technologies (soft and hard ware) such as M-Health(2) | .463 | 1 | .496 |
|  |  | Slack/Financial Resources: M-health adoption is appropriate for hospitals that have excess budgets to invest in new IT technologies (soft and hard ware) such as M-Health(3) | .457 | 1 | .499 |
|  |  | Technology leadership: M-Health is appropriate for hospitals that pursue market growth through technology leadership | 1.349 | 3 | .717 |
|  |  | Technology leadership: M-Health is appropriate for hospitals that pursue market growth through technology leadership (1) | .302 | 1 | .583 |
|  |  | Technology leadership: M-Health is appropriate for hospitals that pursue market growth through technology leadership (2) | .008 | 1 | .927 |
|  |  | Technology leadership: M-Health is appropriate for hospitals that pursue market growth through technology leadership (3) | 1.011 | 1 | .315 |
|  | Overall Statistics | | 14.372 | 21 | .853 |

**Block 1: Method = Enter**

| **Omnibus Tests of Model Coefficients** | | | | |
| --- | --- | --- | --- | --- |
|  | | Chi-square | df | Sig. |
| Step 1 | Step | 14.925 | 21 | .827 |
|  | Block | 14.925 | 21 | .827 |
|  | Model | 14.925 | 21 | .827 |

| **Model Summary** | | | |
| --- | --- | --- | --- |
| Step | -2 Log likelihood | Cox & Snell R Square | Nagelkerke R Square |
| 1 | 223.447^a^ | .072 | .103 |
| a. Estimation terminated at iteration number 4 because parameter estimates changed by less than .001. | | | |

| **Hosmer and Lemeshow Test** | | | |
| --- | --- | --- | --- |
| Step | Chi-square | df | Sig. |
| 1 | 7.132 | 8 | .522 |

| **Contingency Table for Hosmer and Lemeshow Test** | | | | | | |
| --- | --- | --- | --- | --- | --- | --- |
|  | | Treatment compliance = Not adopted | | Treatment compliance = Adopted | | Total |
|  |  | Observed | Expected | Observed | Expected |  |
| Step 1 | 1 | 12 | 10.651 | 8 | 9.349 | 20 |
|  | 2 | 6 | 8.216 | 14 | 11.784 | 20 |
|  | 3 | 8 | 7.069 | 12 | 12.931 | 20 |
|  | 4 | 6 | 6.388 | 14 | 13.612 | 20 |
|  | 5 | 7 | 5.998 | 13 | 14.002 | 20 |
|  | 6 | 4 | 5.223 | 16 | 14.777 | 20 |
|  | 7 | 3 | 5.115 | 20 | 17.885 | 23 |
|  | 8 | 7 | 3.708 | 13 | 16.292 | 20 |
|  | 9 | 3 | 3.138 | 18 | 17.862 | 21 |
|  | 10 | 1 | 1.494 | 14 | 13.506 | 15 |

| **Classification Table^a^** | | | | | |
| --- | --- | --- | --- | --- | --- |
|  | Observed | | Predicted | | |
|  |  |  | Treatment compliance | | Percentage Correct |
|  |  |  | Not adopted | Adopted |  |
| Step 1 | Treatment compliance | Not adopted | 5 | 52 | 8.8 |
|  |  | Adopted | 4 | 138 | 97.2 |
|  | Overall Percentage | |  |  | 71.9 |
| a. The cut value is .500 | | | | | |

| **Variables in the Equation** | | | | | | | | | |
| --- | --- | --- | --- | --- | --- | --- | --- | --- | --- |
|  | | B | S.E. | Wald | df | Sig. | Exp(B) | 95% C.I.for EXP(B) | |
|  |  |  |  |  |  |  |  | Lower | Upper |
| Step 1^a^ | Decision making structure: decision to adopt or not to adopt M-Health adoption is the prerogative of the hospital’s top management only. |  |  | 3.671 | 3 | .299 |  |  |  |
|  | Decision making structure: decision to adopt or not to adopt M-Health adoption is the prerogative of the hospital’s top management only.(1) | 1.049 | .621 | 2.855 | 1 | .091 | 2.856 | .845 | 9.649 |
|  | Decision making structure: decision to adopt or not to adopt M-Health adoption is the prerogative of the hospital’s top management only.(2) | 1.038 | .592 | 3.068 | 1 | .080 | 2.823 | .884 | 9.016 |
|  | Decision making structure: decision to adopt or not to adopt M-Health adoption is the prerogative of the hospital’s top management only.(3) | .979 | .618 | 2.507 | 1 | .113 | 2.661 | .792 | 8.937 |
|  | Size: M-Health adoption is appropriate only when the hospital has substantial volume of patients and staff to justify its adoption |  |  | 1.973 | 3 | .578 |  |  |  |
|  | Size: M-Health adoption is appropriate only when the hospital has substantial volume of patients and staff to justify its adoption(1) | -.136 | .767 | .031 | 1 | .859 | .873 | .194 | 3.926 |
|  | Size: M-Health adoption is appropriate only when the hospital has substantial volume of patients and staff to justify its adoption(2) | .069 | .770 | .008 | 1 | .929 | 1.071 | .237 | 4.849 |
|  | Size: M-Health adoption is appropriate only when the hospital has substantial volume of patients and staff to justify its adoption(3) | -.610 | .727 | .703 | 1 | .402 | .543 | .131 | 2.260 |
|  | ICT capacity: M-Health adoption is appropriate when hospitals have a very complex ICT infrastructure |  |  | 1.301 | 3 | .729 |  |  |  |
|  | ICT capacity: M-Health adoption is appropriate when hospitals have a very complex ICT infrastructure(1) | -.391 | .844 | .215 | 1 | .643 | .676 | .129 | 3.538 |
|  | ICT capacity: M-Health adoption is appropriate when hospitals have a very complex ICT infrastructure(2) | -.324 | .791 | .168 | 1 | .682 | .723 | .153 | 3.412 |
|  | ICT capacity: M-Health adoption is appropriate when hospitals have a very complex ICT infrastructure(3) | .340 | .861 | .156 | 1 | .693 | 1.405 | .260 | 7.591 |
|  | ICT staff : M-Health adoption is appropriate when hospitals have very knowledgeable and adequate number of staff in ICT |  |  | .459 | 3 | .928 |  |  |  |
|  | ICT staff : M-Health adoption is appropriate when hospitals have very knowledgeable and adequate number of staff in ICT (1) | -.272 | .797 | .116 | 1 | .733 | .762 | .160 | 3.635 |
|  | ICT staff : M-Health adoption is appropriate when hospitals have very knowledgeable and adequate number of staff in ICT (2) | -.233 | .641 | .133 | 1 | .715 | .792 | .226 | 2.779 |
|  | ICT staff : M-Health adoption is appropriate when hospitals have very knowledgeable and adequate number of staff in ICT (3) | -.398 | .610 | .424 | 1 | .515 | .672 | .203 | 2.222 |
|  | Scope of the Market: M-Health adoption is appropriate for hospitals with larger market scopes such as national, regional or global markets. |  |  | 5.828 | 3 | .120 |  |  |  |
|  | Scope of the Market: M-Health adoption is appropriate for hospitals with larger market scopes such as national, regional or global markets.(1) | .404 | .780 | .268 | 1 | .605 | 1.497 | .325 | 6.907 |
|  | Scope of the Market: M-Health adoption is appropriate for hospitals with larger market scopes such as national, regional or global markets.(2) | -.835 | .692 | 1.458 | 1 | .227 | .434 | .112 | 1.683 |
|  | Scope of the Market: M-Health adoption is appropriate for hospitals with larger market scopes such as national, regional or global markets.(3) | -.601 | .746 | .648 | 1 | .421 | .548 | .127 | 2.367 |
|  | Slack/Financial Resources: M-health adoption is appropriate for hospitals that have excess budgets to invest in new IT technologies (soft and hard ware) such as M-Health |  |  | 1.919 | 3 | .589 |  |  |  |
|  | Slack/Financial Resources: M-health adoption is appropriate for hospitals that have excess budgets to invest in new IT technologies (soft and hard ware) such as M-Health(1) | -.122 | .785 | .024 | 1 | .876 | .885 | .190 | 4.123 |
|  | Slack/Financial Resources: M-health adoption is appropriate for hospitals that have excess budgets to invest in new IT technologies (soft and hard ware) such as M-Health(2) | .317 | .781 | .165 | 1 | .685 | 1.373 | .297 | 6.352 |
|  | Slack/Financial Resources: M-health adoption is appropriate for hospitals that have excess budgets to invest in new IT technologies (soft and hard ware) such as M-Health(3) | .590 | .775 | .579 | 1 | .447 | 1.803 | .395 | 8.236 |
|  | Technology leadership: M-Health is appropriate for hospitals that pursue market growth through technology leadership |  |  | .703 | 3 | .873 |  |  |  |
|  | Technology leadership: M-Health is appropriate for hospitals that pursue market growth through technology leadership (1) | -.122 | .670 | .033 | 1 | .856 | .886 | .238 | 3.292 |
|  | Technology leadership: M-Health is appropriate for hospitals that pursue market growth through technology leadership (2) | -.380 | .564 | .455 | 1 | .500 | .684 | .226 | 2.064 |
|  | Technology leadership: M-Health is appropriate for hospitals that pursue market growth through technology leadership (3) | -.384 | .513 | .561 | 1 | .454 | .681 | .249 | 1.860 |
|  | Constant | 1.155 | .827 | 1.947 | 1 | .163 | 3.173 |  |  |
| a. Variable(s) entered on step 1: Decision making structure: decision to adopt or not to adopt M-Health adoption is the prerogative of the hospital’s top management only., Size: M-Health adoption is appropriate only when the hospital has substantial volume of patients and staff to justify its adoption, ICT capacity: M-Health adoption is appropriate when hospitals have a very complex ICT infrastructure, ICT staff : M-Health adoption is appropriate when hospitals have very knowledgeable and adequate number of staff in ICT , Scope of the Market: M-Health adoption is appropriate for hospitals with larger market scopes such as national, regional or global markets., Slack/Financial Resources: M-health adoption is appropriate for hospitals that have excess budgets to invest in new IT technologies (soft and hard ware) such as M-Health, Technology leadership: M-Health is appropriate for hospitals that pursue market growth through technology leadership . | | | | | | | | | |

1. **Appointment reminders technology Adoption and organizational determinants**

**Logistic Regression**

| **Notes** | | |
| --- | --- | --- |
| Output Created | | 15-JUL-2018 14:56:01 |
| Comments | |  |
| Input | Data | C:\Users\Ocholap\Downloads\m-health data.- 13-JULY 2018.sav |
|  | Active Dataset | DataSet1 |
|  | Filter | <none> |
|  | Weight | <none> |
|  | Split File | <none> |
|  | N of Rows in Working Data File | 211 |
| Missing Value Handling | Definition of Missing | User-defined missing values are treated as missing |
| Syntax | | LOGISTIC REGRESSION VARIABLES Q20.4  /METHOD=ENTER Q24.1 Q24.2 Q24.3 Q24.4 Q24.5 Q24.6 Q24.7  /CONTRAST (Q24.1)=Indicator  /CONTRAST (Q24.2)=Indicator  /CONTRAST (Q24.3)=Indicator  /CONTRAST (Q24.4)=Indicator  /CONTRAST (Q24.5)=Indicator  /CONTRAST (Q24.6)=Indicator  /CONTRAST (Q24.7)=Indicator  /SAVE=PGROUP  /PRINT=GOODFIT SUMMARY CI(95)  /CRITERIA=PIN(0.05) POUT(0.10) ITERATE(20) CUT(0.5). |
| Resources | Processor Time | 00:00:00.06 |
|  | Elapsed Time | 00:00:00.05 |
| Variables Created or Modified | PGR_31 | Predicted group |

| **Case Processing Summary** | | | |
| --- | --- | --- | --- |
| Unweighted Cases^a^ | | N | Percent |
| Selected Cases | Included in Analysis | 207 | 98.1 |
|  | Missing Cases | 4 | 1.9 |
|  | Total | 211 | 100.0 |
| Unselected Cases | | 0 | .0 |
| Total | | 211 | 100.0 |
| a. If weight is in effect, see classification table for the total number of cases. | | | |

| **Dependent Variable Encoding** | |
| --- | --- |
| Original Value | Internal Value |
| Not adopted | 0 |
| Adopted | 1 |

| **Categorical Variables Codings** | | | | | |
| --- | --- | --- | --- | --- | --- |
|  | | Frequency | Parameter coding | | |
|  |  |  | (1) | (2) | (3) |
| Technology leadership: M-Health is appropriate for hospitals that pursue market growth through technology leadership | Strongly disgree | 30 | 1.000 | .000 | .000 |
|  | Disagree | 48 | .000 | 1.000 | .000 |
|  | Agree | 76 | .000 | .000 | 1.000 |
|  | Strongly Agree | 53 | .000 | .000 | .000 |
| Size: M-Health adoption is appropriate only when the hospital has substantial volume of patients and staff to justify its adoption | Strongly disgree | 59 | 1.000 | .000 | .000 |
|  | Disagree | 81 | .000 | 1.000 | .000 |
|  | Agree | 48 | .000 | .000 | 1.000 |
|  | Strongly Agree | 19 | .000 | .000 | .000 |
| ICT capacity: M-Health adoption is appropriate when hospitals have a very complex ICT infrastructure | Strongly disgree | 64 | 1.000 | .000 | .000 |
|  | Disagree | 93 | .000 | 1.000 | .000 |
|  | Agree | 26 | .000 | .000 | 1.000 |
|  | Strongly Agree | 24 | .000 | .000 | .000 |
| ICT staff : M-Health adoption is appropriate when hospitals have very knowledgeable and adequate number of staff in ICT | Strongly disgree | 27 | 1.000 | .000 | .000 |
|  | Disagree | 67 | .000 | 1.000 | .000 |
|  | Agree | 74 | .000 | .000 | 1.000 |
|  | Strongly Agree | 39 | .000 | .000 | .000 |
| Scope of the Market: M-Health adoption is appropriate for hospitals with larger market scopes such as national, regional or global markets. | Strongly disgree | 56 | 1.000 | .000 | .000 |
|  | Disagree | 87 | .000 | 1.000 | .000 |
|  | Agree | 38 | .000 | .000 | 1.000 |
|  | Strongly Agree | 26 | .000 | .000 | .000 |
| Slack/Financial Resources: M-health adoption is appropriate for hospitals that have excess budgets to invest in new IT technologies (soft and hard ware) such as M-Health | Strongly disgree | 74 | 1.000 | .000 | .000 |
|  | Disagree | 75 | .000 | 1.000 | .000 |
|  | Agree | 44 | .000 | .000 | 1.000 |
|  | Strongly Agree | 14 | .000 | .000 | .000 |
| Decision making structure: decision to adopt or not to adopt M-Health adoption is the prerogative of the hospital’s top management only. | Strongly disgree | 57 | 1.000 | .000 | .000 |
|  | Disagree | 76 | .000 | 1.000 | .000 |
|  | Agree | 43 | .000 | .000 | 1.000 |
|  | Strongly Agree | 31 | .000 | .000 | .000 |

**Block 0: Beginning Block**

| **Classification Table^a,b^** | | | | | |
| --- | --- | --- | --- | --- | --- |
|  | Observed | | Predicted | | |
|  |  |  | Appointment reminders | | Percentage Correct |
|  |  |  | Not adopted | Adopted |  |
| Step 0 | Appointment reminders | Not adopted | 0 | 55 | .0 |
|  |  | Adopted | 0 | 152 | 100.0 |
|  | Overall Percentage | |  |  | 73.4 |
| a. Constant is included in the model. | | | | | |
| b. The cut value is .500 | | | | | |

| **Variables in the Equation** | | | | | | | |
| --- | --- | --- | --- | --- | --- | --- | --- |
|  | | B | S.E. | Wald | df | Sig. | Exp(B) |
| Step 0 | Constant | 1.017 | .157 | 41.734 | 1 | .000 | 2.764 |

| **Variables not in the Equation** | | | | | |
| --- | --- | --- | --- | --- | --- |
|  | | | Score | df | Sig. |
| Step 0 | Variables | Decision making structure: decision to adopt or not to adopt M-Health adoption is the prerogative of the hospital’s top management only. | 2.944 | 3 | .400 |
|  |  | Decision making structure: decision to adopt or not to adopt M-Health adoption is the prerogative of the hospital’s top management only.(1) | 1.012 | 1 | .315 |
|  |  | Decision making structure: decision to adopt or not to adopt M-Health adoption is the prerogative of the hospital’s top management only.(2) | 2.874 | 1 | .090 |
|  |  | Decision making structure: decision to adopt or not to adopt M-Health adoption is the prerogative of the hospital’s top management only.(3) | .373 | 1 | .541 |
|  |  | Size: M-Health adoption is appropriate only when the hospital has substantial volume of patients and staff to justify its adoption | 2.746 | 3 | .432 |
|  |  | Size: M-Health adoption is appropriate only when the hospital has substantial volume of patients and staff to justify its adoption(1) | 1.642 | 1 | .200 |
|  |  | Size: M-Health adoption is appropriate only when the hospital has substantial volume of patients and staff to justify its adoption(2) | 2.085 | 1 | .149 |
|  |  | Size: M-Health adoption is appropriate only when the hospital has substantial volume of patients and staff to justify its adoption(3) | .008 | 1 | .927 |
|  |  | ICT capacity: M-Health adoption is appropriate when hospitals have a very complex ICT infrastructure | 6.685 | 3 | .083 |
|  |  | ICT capacity: M-Health adoption is appropriate when hospitals have a very complex ICT infrastructure(1) | 2.904 | 1 | .088 |
|  |  | ICT capacity: M-Health adoption is appropriate when hospitals have a very complex ICT infrastructure(2) | 5.318 | 1 | .021 |
|  |  | ICT capacity: M-Health adoption is appropriate when hospitals have a very complex ICT infrastructure(3) | 1.907 | 1 | .167 |
|  |  | ICT staff : M-Health adoption is appropriate when hospitals have very knowledgeable and adequate number of staff in ICT | .034 | 3 | .998 |
|  |  | ICT staff : M-Health adoption is appropriate when hospitals have very knowledgeable and adequate number of staff in ICT (1) | .007 | 1 | .935 |
|  |  | ICT staff : M-Health adoption is appropriate when hospitals have very knowledgeable and adequate number of staff in ICT (2) | .004 | 1 | .947 |
|  |  | ICT staff : M-Health adoption is appropriate when hospitals have very knowledgeable and adequate number of staff in ICT (3) | .012 | 1 | .912 |
|  |  | Scope of the Market: M-Health adoption is appropriate for hospitals with larger market scopes such as national, regional or global markets. | 3.803 | 3 | .283 |
|  |  | Scope of the Market: M-Health adoption is appropriate for hospitals with larger market scopes such as national, regional or global markets.(1) | 1.040 | 1 | .308 |
|  |  | Scope of the Market: M-Health adoption is appropriate for hospitals with larger market scopes such as national, regional or global markets.(2) | 1.533 | 1 | .216 |
|  |  | Scope of the Market: M-Health adoption is appropriate for hospitals with larger market scopes such as national, regional or global markets.(3) | .599 | 1 | .439 |
|  |  | Slack/Financial Resources: M-health adoption is appropriate for hospitals that have excess budgets to invest in new IT technologies (soft and hard ware) such as M-Health | 9.452 | 3 | .024 |
|  |  | Slack/Financial Resources: M-health adoption is appropriate for hospitals that have excess budgets to invest in new IT technologies (soft and hard ware) such as M-Health(1) | 6.328 | 1 | .012 |
|  |  | Slack/Financial Resources: M-health adoption is appropriate for hospitals that have excess budgets to invest in new IT technologies (soft and hard ware) such as M-Health(2) | 6.984 | 1 | .008 |
|  |  | Slack/Financial Resources: M-health adoption is appropriate for hospitals that have excess budgets to invest in new IT technologies (soft and hard ware) such as M-Health(3) | .423 | 1 | .515 |
|  |  | Technology leadership: M-Health is appropriate for hospitals that pursue market growth through technology leadership | 2.726 | 3 | .436 |
|  |  | Technology leadership: M-Health is appropriate for hospitals that pursue market growth through technology leadership (1) | .776 | 1 | .378 |
|  |  | Technology leadership: M-Health is appropriate for hospitals that pursue market growth through technology leadership (2) | .216 | 1 | .642 |
|  |  | Technology leadership: M-Health is appropriate for hospitals that pursue market growth through technology leadership (3) | 1.544 | 1 | .214 |
|  | Overall Statistics | | 25.677 | 21 | .219 |

**Block 1: Method = Enter**

| **Omnibus Tests of Model Coefficients** | | | | |
| --- | --- | --- | --- | --- |
|  | | Chi-square | df | Sig. |
| Step 1 | Step | 28.084 | 21 | .138 |
|  | Block | 28.084 | 21 | .138 |
|  | Model | 28.084 | 21 | .138 |

| **Model Summary** | | | |
| --- | --- | --- | --- |
| Step | -2 Log likelihood | Cox & Snell R Square | Nagelkerke R Square |
| 1 | 211.596^a^ | .127 | .185 |
| a. Estimation terminated at iteration number 5 because parameter estimates changed by less than .001. | | | |

| **Hosmer and Lemeshow Test** | | | |
| --- | --- | --- | --- |
| Step | Chi-square | df | Sig. |
| 1 | 8.649 | 8 | .373 |

| **Contingency Table for Hosmer and Lemeshow Test** | | | | | | |
| --- | --- | --- | --- | --- | --- | --- |
|  | | Appointment reminders = Not adopted | | Appointment reminders = Adopted | | Total |
|  |  | Observed | Expected | Observed | Expected |  |
| Step 1 | 1 | 13 | 12.285 | 8 | 8.715 | 21 |
|  | 2 | 8 | 9.369 | 13 | 11.631 | 21 |
|  | 3 | 4 | 7.818 | 17 | 13.182 | 21 |
|  | 4 | 8 | 6.161 | 12 | 13.839 | 20 |
|  | 5 | 9 | 5.454 | 12 | 15.546 | 21 |
|  | 6 | 5 | 4.866 | 18 | 18.134 | 23 |
|  | 7 | 4 | 3.630 | 18 | 18.370 | 22 |
|  | 8 | 3 | 2.773 | 18 | 18.227 | 21 |
|  | 9 | 1 | 1.935 | 20 | 19.065 | 21 |
|  | 10 | 0 | .708 | 16 | 15.292 | 16 |

| **Classification Table^a^** | | | | | |
| --- | --- | --- | --- | --- | --- |
|  | Observed | | Predicted | | |
|  |  |  | Appointment reminders | | Percentage Correct |
|  |  |  | Not adopted | Adopted |  |
| Step 1 | Appointment reminders | Not adopted | 14 | 41 | 25.5 |
|  |  | Adopted | 8 | 144 | 94.7 |
|  | Overall Percentage | |  |  | 76.3 |
| a. The cut value is .500 | | | | | |

| **Variables in the Equation** | | | | | | | | | |
| --- | --- | --- | --- | --- | --- | --- | --- | --- | --- |
|  | | B | S.E. | Wald | df | Sig. | Exp(B) | 95% C.I.for EXP(B) | |
|  |  |  |  |  |  |  |  | Lower | Upper |
| Step 1^a^ | Decision making structure: decision to adopt or not to adopt M-Health adoption is the prerogative of the hospital’s top management only. |  |  | 7.623 | 3 | .054 |  |  |  |
|  | Decision making structure: decision to adopt or not to adopt M-Health adoption is the prerogative of the hospital’s top management only.(1) | -.781 | .661 | 1.396 | 1 | .237 | .458 | .125 | 1.673 |
|  | Decision making structure: decision to adopt or not to adopt M-Health adoption is the prerogative of the hospital’s top management only.(2) | .718 | .607 | 1.397 | 1 | .237 | 2.049 | .624 | 6.735 |
|  | Decision making structure: decision to adopt or not to adopt M-Health adoption is the prerogative of the hospital’s top management only.(3) | .147 | .639 | .053 | 1 | .818 | 1.159 | .331 | 4.057 |
|  | Size: M-Health adoption is appropriate only when the hospital has substantial volume of patients and staff to justify its adoption |  |  | 2.755 | 3 | .431 |  |  |  |
|  | Size: M-Health adoption is appropriate only when the hospital has substantial volume of patients and staff to justify its adoption(1) | -.203 | .869 | .055 | 1 | .815 | .816 | .149 | 4.483 |
|  | Size: M-Health adoption is appropriate only when the hospital has substantial volume of patients and staff to justify its adoption(2) | -.996 | .852 | 1.368 | 1 | .242 | .369 | .070 | 1.960 |
|  | Size: M-Health adoption is appropriate only when the hospital has substantial volume of patients and staff to justify its adoption(3) | -.479 | .814 | .347 | 1 | .556 | .619 | .126 | 3.051 |
|  | ICT capacity: M-Health adoption is appropriate when hospitals have a very complex ICT infrastructure |  |  | 4.596 | 3 | .204 |  |  |  |
|  | ICT capacity: M-Health adoption is appropriate when hospitals have a very complex ICT infrastructure(1) | 1.288 | .900 | 2.049 | 1 | .152 | 3.627 | .622 | 21.163 |
|  | ICT capacity: M-Health adoption is appropriate when hospitals have a very complex ICT infrastructure(2) | .572 | .821 | .485 | 1 | .486 | 1.771 | .354 | 8.852 |
|  | ICT capacity: M-Health adoption is appropriate when hospitals have a very complex ICT infrastructure(3) | 1.369 | .915 | 2.240 | 1 | .134 | 3.933 | .654 | 23.638 |
|  | ICT staff : M-Health adoption is appropriate when hospitals have very knowledgeable and adequate number of staff in ICT |  |  | 1.945 | 3 | .584 |  |  |  |
|  | ICT staff : M-Health adoption is appropriate when hospitals have very knowledgeable and adequate number of staff in ICT (1) | -.907 | .845 | 1.152 | 1 | .283 | .404 | .077 | 2.115 |
|  | ICT staff : M-Health adoption is appropriate when hospitals have very knowledgeable and adequate number of staff in ICT (2) | -.053 | .681 | .006 | 1 | .938 | .948 | .250 | 3.601 |
|  | ICT staff : M-Health adoption is appropriate when hospitals have very knowledgeable and adequate number of staff in ICT (3) | -.367 | .658 | .310 | 1 | .578 | .693 | .191 | 2.520 |
|  | Scope of the Market: M-Health adoption is appropriate for hospitals with larger market scopes such as national, regional or global markets. |  |  | 3.083 | 3 | .379 |  |  |  |
|  | Scope of the Market: M-Health adoption is appropriate for hospitals with larger market scopes such as national, regional or global markets.(1) | -.934 | .859 | 1.182 | 1 | .277 | .393 | .073 | 2.117 |
|  | Scope of the Market: M-Health adoption is appropriate for hospitals with larger market scopes such as national, regional or global markets.(2) | -1.147 | .793 | 2.091 | 1 | .148 | .318 | .067 | 1.503 |
|  | Scope of the Market: M-Health adoption is appropriate for hospitals with larger market scopes such as national, regional or global markets.(3) | -1.421 | .822 | 2.985 | 1 | .084 | .241 | .048 | 1.211 |
|  | Slack/Financial Resources: M-health adoption is appropriate for hospitals that have excess budgets to invest in new IT technologies (soft and hard ware) such as M-Health |  |  | 7.308 | 3 | .063 |  |  |  |
|  | Slack/Financial Resources: M-health adoption is appropriate for hospitals that have excess budgets to invest in new IT technologies (soft and hard ware) such as M-Health(1) | 1.782 | .850 | 4.395 | 1 | .036 | 5.943 | 1.123 | 31.454 |
|  | Slack/Financial Resources: M-health adoption is appropriate for hospitals that have excess budgets to invest in new IT technologies (soft and hard ware) such as M-Health(2) | .830 | .803 | 1.069 | 1 | .301 | 2.294 | .475 | 11.069 |
|  | Slack/Financial Resources: M-health adoption is appropriate for hospitals that have excess budgets to invest in new IT technologies (soft and hard ware) such as M-Health(3) | 1.443 | .810 | 3.173 | 1 | .075 | 4.233 | .865 | 20.712 |
|  | Technology leadership: M-Health is appropriate for hospitals that pursue market growth through technology leadership |  |  | .901 | 3 | .825 |  |  |  |
|  | Technology leadership: M-Health is appropriate for hospitals that pursue market growth through technology leadership (1) | .256 | .718 | .127 | 1 | .722 | 1.292 | .316 | 5.279 |
|  | Technology leadership: M-Health is appropriate for hospitals that pursue market growth through technology leadership (2) | -.330 | .601 | .302 | 1 | .583 | .719 | .222 | 2.333 |
|  | Technology leadership: M-Health is appropriate for hospitals that pursue market growth through technology leadership (3) | -.025 | .553 | .002 | 1 | .964 | .976 | .330 | 2.884 |
|  | Constant | .928 | .836 | 1.234 | 1 | .267 | 2.530 |  |  |
| a. Variable(s) entered on step 1: Decision making structure: decision to adopt or not to adopt M-Health adoption is the prerogative of the hospital’s top management only., Size: M-Health adoption is appropriate only when the hospital has substantial volume of patients and staff to justify its adoption, ICT capacity: M-Health adoption is appropriate when hospitals have a very complex ICT infrastructure, ICT staff : M-Health adoption is appropriate when hospitals have very knowledgeable and adequate number of staff in ICT , Scope of the Market: M-Health adoption is appropriate for hospitals with larger market scopes such as national, regional or global markets., Slack/Financial Resources: M-health adoption is appropriate for hospitals that have excess budgets to invest in new IT technologies (soft and hard ware) such as M-Health, Technology leadership: M-Health is appropriate for hospitals that pursue market growth through technology leadership . | | | | | | | | | |

1. **Community mobilization technology Adoption and organizational determinants**

**Logistic Regression**

| **Notes** | | |
| --- | --- | --- |
| Output Created | | 15-JUL-2018 14:58:39 |
| Comments | |  |
| Input | Data | C:\Users\Ocholap\Downloads\m-health data.- 13-JULY 2018.sav |
|  | Active Dataset | DataSet1 |
|  | Filter | <none> |
|  | Weight | <none> |
|  | Split File | <none> |
|  | N of Rows in Working Data File | 211 |
| Missing Value Handling | Definition of Missing | User-defined missing values are treated as missing |
| Syntax | | LOGISTIC REGRESSION VARIABLES Q20.5  /METHOD=ENTER Q24.1 Q24.2 Q24.3 Q24.4 Q24.5 Q24.6 Q24.7  /CONTRAST (Q24.1)=Indicator  /CONTRAST (Q24.2)=Indicator  /CONTRAST (Q24.3)=Indicator  /CONTRAST (Q24.4)=Indicator  /CONTRAST (Q24.5)=Indicator  /CONTRAST (Q24.6)=Indicator  /CONTRAST (Q24.7)=Indicator  /SAVE=PGROUP  /PRINT=GOODFIT SUMMARY CI(95)  /CRITERIA=PIN(0.05) POUT(0.10) ITERATE(20) CUT(0.5). |
| Resources | Processor Time | 00:00:00.05 |
|  | Elapsed Time | 00:00:00.05 |
| Variables Created or Modified | PGR_32 | Predicted group |

| **Case Processing Summary** | | | |
| --- | --- | --- | --- |
| Unweighted Cases^a^ | | N | Percent |
| Selected Cases | Included in Analysis | 204 | 96.7 |
|  | Missing Cases | 7 | 3.3 |
|  | Total | 211 | 100.0 |
| Unselected Cases | | 0 | .0 |
| Total | | 211 | 100.0 |
| a. If weight is in effect, see classification table for the total number of cases. | | | |

| **Dependent Variable Encoding** | |
| --- | --- |
| Original Value | Internal Value |
| Not adopted | 0 |
| Adopted | 1 |

| **Categorical Variables Codings** | | | | | |
| --- | --- | --- | --- | --- | --- |
|  | | Frequency | Parameter coding | | |
|  |  |  | (1) | (2) | (3) |
| Technology leadership: M-Health is appropriate for hospitals that pursue market growth through technology leadership | Strongly disgree | 29 | 1.000 | .000 | .000 |
|  | Disagree | 48 | .000 | 1.000 | .000 |
|  | Agree | 75 | .000 | .000 | 1.000 |
|  | Strongly Agree | 52 | .000 | .000 | .000 |
| Size: M-Health adoption is appropriate only when the hospital has substantial volume of patients and staff to justify its adoption | Strongly disgree | 58 | 1.000 | .000 | .000 |
|  | Disagree | 81 | .000 | 1.000 | .000 |
|  | Agree | 47 | .000 | .000 | 1.000 |
|  | Strongly Agree | 18 | .000 | .000 | .000 |
| ICT capacity: M-Health adoption is appropriate when hospitals have a very complex ICT infrastructure | Strongly disgree | 61 | 1.000 | .000 | .000 |
|  | Disagree | 93 | .000 | 1.000 | .000 |
|  | Agree | 26 | .000 | .000 | 1.000 |
|  | Strongly Agree | 24 | .000 | .000 | .000 |
| ICT staff : M-Health adoption is appropriate when hospitals have very knowledgeable and adequate number of staff in ICT | Strongly disgree | 25 | 1.000 | .000 | .000 |
|  | Disagree | 65 | .000 | 1.000 | .000 |
|  | Agree | 75 | .000 | .000 | 1.000 |
|  | Strongly Agree | 39 | .000 | .000 | .000 |
| Scope of the Market: M-Health adoption is appropriate for hospitals with larger market scopes such as national, regional or global markets. | Strongly disgree | 54 | 1.000 | .000 | .000 |
|  | Disagree | 87 | .000 | 1.000 | .000 |
|  | Agree | 38 | .000 | .000 | 1.000 |
|  | Strongly Agree | 25 | .000 | .000 | .000 |
| Slack/Financial Resources: M-health adoption is appropriate for hospitals that have excess budgets to invest in new IT technologies (soft and hard ware) such as M-Health | Strongly disgree | 72 | 1.000 | .000 | .000 |
|  | Disagree | 74 | .000 | 1.000 | .000 |
|  | Agree | 44 | .000 | .000 | 1.000 |
|  | Strongly Agree | 14 | .000 | .000 | .000 |
| Decision making structure: decision to adopt or not to adopt M-Health adoption is the prerogative of the hospital’s top management only. | Strongly disgree | 56 | 1.000 | .000 | .000 |
|  | Disagree | 76 | .000 | 1.000 | .000 |
|  | Agree | 42 | .000 | .000 | 1.000 |
|  | Strongly Agree | 30 | .000 | .000 | .000 |

**Block 0: Beginning Block**

| **Classification Table^a,b^** | | | | | |
| --- | --- | --- | --- | --- | --- |
|  | Observed | | Predicted | | |
|  |  |  | Community mobilization | | Percentage Correct |
|  |  |  | Not adopted | Adopted |  |
| Step 0 | Community mobilization | Not adopted | 0 | 77 | .0 |
|  |  | Adopted | 0 | 127 | 100.0 |
|  | Overall Percentage | |  |  | 62.3 |
| a. Constant is included in the model. | | | | | |
| b. The cut value is .500 | | | | | |

| **Variables in the Equation** | | | | | | | |
| --- | --- | --- | --- | --- | --- | --- | --- |
|  | | B | S.E. | Wald | df | Sig. | Exp(B) |
| Step 0 | Constant | .500 | .144 | 12.002 | 1 | .001 | 1.649 |

| **Variables not in the Equation** | | | | | |
| --- | --- | --- | --- | --- | --- |
|  | | | Score | df | Sig. |
| Step 0 | Variables | Decision making structure: decision to adopt or not to adopt M-Health adoption is the prerogative of the hospital’s top management only. | 2.631 | 3 | .452 |
|  |  | Decision making structure: decision to adopt or not to adopt M-Health adoption is the prerogative of the hospital’s top management only.(1) | .363 | 1 | .547 |
|  |  | Decision making structure: decision to adopt or not to adopt M-Health adoption is the prerogative of the hospital’s top management only.(2) | .980 | 1 | .322 |
|  |  | Decision making structure: decision to adopt or not to adopt M-Health adoption is the prerogative of the hospital’s top management only.(3) | 1.894 | 1 | .169 |
|  |  | Size: M-Health adoption is appropriate only when the hospital has substantial volume of patients and staff to justify its adoption | 5.318 | 3 | .150 |
|  |  | Size: M-Health adoption is appropriate only when the hospital has substantial volume of patients and staff to justify its adoption(1) | 3.559 | 1 | .059 |
|  |  | Size: M-Health adoption is appropriate only when the hospital has substantial volume of patients and staff to justify its adoption(2) | 2.566 | 1 | .109 |
|  |  | Size: M-Health adoption is appropriate only when the hospital has substantial volume of patients and staff to justify its adoption(3) | .601 | 1 | .438 |
|  |  | ICT capacity: M-Health adoption is appropriate when hospitals have a very complex ICT infrastructure | 5.323 | 3 | .150 |
|  |  | ICT capacity: M-Health adoption is appropriate when hospitals have a very complex ICT infrastructure(1) | 4.911 | 1 | .027 |
|  |  | ICT capacity: M-Health adoption is appropriate when hospitals have a very complex ICT infrastructure(2) | 2.017 | 1 | .156 |
|  |  | ICT capacity: M-Health adoption is appropriate when hospitals have a very complex ICT infrastructure(3) | .897 | 1 | .344 |
|  |  | ICT staff : M-Health adoption is appropriate when hospitals have very knowledgeable and adequate number of staff in ICT | 1.099 | 3 | .777 |
|  |  | ICT staff : M-Health adoption is appropriate when hospitals have very knowledgeable and adequate number of staff in ICT (1) | .400 | 1 | .527 |
|  |  | ICT staff : M-Health adoption is appropriate when hospitals have very knowledgeable and adequate number of staff in ICT (2) | .021 | 1 | .885 |
|  |  | ICT staff : M-Health adoption is appropriate when hospitals have very knowledgeable and adequate number of staff in ICT (3) | .650 | 1 | .420 |
|  |  | Scope of the Market: M-Health adoption is appropriate for hospitals with larger market scopes such as national, regional or global markets. | 2.747 | 3 | .432 |
|  |  | Scope of the Market: M-Health adoption is appropriate for hospitals with larger market scopes such as national, regional or global markets.(1) | 2.058 | 1 | .151 |
|  |  | Scope of the Market: M-Health adoption is appropriate for hospitals with larger market scopes such as national, regional or global markets.(2) | .115 | 1 | .734 |
|  |  | Scope of the Market: M-Health adoption is appropriate for hospitals with larger market scopes such as national, regional or global markets.(3) | .059 | 1 | .807 |
|  |  | Slack/Financial Resources: M-health adoption is appropriate for hospitals that have excess budgets to invest in new IT technologies (soft and hard ware) such as M-Health | 2.448 | 3 | .485 |
|  |  | Slack/Financial Resources: M-health adoption is appropriate for hospitals that have excess budgets to invest in new IT technologies (soft and hard ware) such as M-Health(1) | .126 | 1 | .722 |
|  |  | Slack/Financial Resources: M-health adoption is appropriate for hospitals that have excess budgets to invest in new IT technologies (soft and hard ware) such as M-Health(2) | 1.494 | 1 | .222 |
|  |  | Slack/Financial Resources: M-health adoption is appropriate for hospitals that have excess budgets to invest in new IT technologies (soft and hard ware) such as M-Health(3) | 1.605 | 1 | .205 |
|  |  | Technology leadership: M-Health is appropriate for hospitals that pursue market growth through technology leadership | 2.543 | 3 | .468 |
|  |  | Technology leadership: M-Health is appropriate for hospitals that pursue market growth through technology leadership (1) | 1.485 | 1 | .223 |
|  |  | Technology leadership: M-Health is appropriate for hospitals that pursue market growth through technology leadership (2) | .520 | 1 | .471 |
|  |  | Technology leadership: M-Health is appropriate for hospitals that pursue market growth through technology leadership (3) | .650 | 1 | .420 |
|  | Overall Statistics | | 24.399 | 21 | .274 |

**Block 1: Method = Enter**

| **Omnibus Tests of Model Coefficients** | | | | |
| --- | --- | --- | --- | --- |
|  | | Chi-square | df | Sig. |
| Step 1 | Step | 26.052 | 21 | .204 |
|  | Block | 26.052 | 21 | .204 |
|  | Model | 26.052 | 21 | .204 |

| **Model Summary** | | | |
| --- | --- | --- | --- |
| Step | -2 Log likelihood | Cox & Snell R Square | Nagelkerke R Square |
| 1 | 244.372^a^ | .120 | .163 |
| a. Estimation terminated at iteration number 4 because parameter estimates changed by less than .001. | | | |

| **Hosmer and Lemeshow Test** | | | |
| --- | --- | --- | --- |
| Step | Chi-square | df | Sig. |
| 1 | 4.297 | 8 | .829 |

| **Contingency Table for Hosmer and Lemeshow Test** | | | | | | |
| --- | --- | --- | --- | --- | --- | --- |
|  | | Community mobilization = Not adopted | | Community mobilization = Adopted | | Total |
|  |  | Observed | Expected | Observed | Expected |  |
| Step 1 | 1 | 14 | 13.672 | 6 | 6.328 | 20 |
|  | 2 | 12 | 11.399 | 8 | 8.601 | 20 |
|  | 3 | 9 | 10.000 | 11 | 10.000 | 20 |
|  | 4 | 8 | 9.189 | 12 | 10.811 | 20 |
|  | 5 | 9 | 7.961 | 11 | 12.039 | 20 |
|  | 6 | 7 | 6.884 | 13 | 13.116 | 20 |
|  | 7 | 5 | 5.761 | 15 | 14.239 | 20 |
|  | 8 | 8 | 4.936 | 12 | 15.064 | 20 |
|  | 9 | 3 | 4.099 | 17 | 15.901 | 20 |
|  | 10 | 2 | 3.100 | 22 | 20.900 | 24 |

| **Classification Table^a^** | | | | | |
| --- | --- | --- | --- | --- | --- |
|  | Observed | | Predicted | | |
|  |  |  | Community mobilization | | Percentage Correct |
|  |  |  | Not adopted | Adopted |  |
| Step 1 | Community mobilization | Not adopted | 29 | 48 | 37.7 |
|  |  | Adopted | 19 | 108 | 85.0 |
|  | Overall Percentage | |  |  | 67.2 |
| a. The cut value is .500 | | | | | |

| **Variables in the Equation** | | | | | | | | | |
| --- | --- | --- | --- | --- | --- | --- | --- | --- | --- |
|  | | B | S.E. | Wald | df | Sig. | Exp(B) | 95% C.I.for EXP(B) | |
|  |  |  |  |  |  |  |  | Lower | Upper |
| Step 1^a^ | Decision making structure: decision to adopt or not to adopt M-Health adoption is the prerogative of the hospital’s top management only. |  |  | 8.064 | 3 | .045 |  |  |  |
|  | Decision making structure: decision to adopt or not to adopt M-Health adoption is the prerogative of the hospital’s top management only.(1) | -.965 | .637 | 2.299 | 1 | .129 | .381 | .109 | 1.326 |
|  | Decision making structure: decision to adopt or not to adopt M-Health adoption is the prerogative of the hospital’s top management only.(2) | -.305 | .567 | .289 | 1 | .591 | .737 | .243 | 2.239 |
|  | Decision making structure: decision to adopt or not to adopt M-Health adoption is the prerogative of the hospital’s top management only.(3) | .641 | .630 | 1.035 | 1 | .309 | 1.898 | .552 | 6.523 |
|  | Size: M-Health adoption is appropriate only when the hospital has substantial volume of patients and staff to justify its adoption |  |  | 5.329 | 3 | .149 |  |  |  |
|  | Size: M-Health adoption is appropriate only when the hospital has substantial volume of patients and staff to justify its adoption(1) | -.124 | .777 | .026 | 1 | .873 | .883 | .192 | 4.052 |
|  | Size: M-Health adoption is appropriate only when the hospital has substantial volume of patients and staff to justify its adoption(2) | -1.098 | .765 | 2.057 | 1 | .151 | .334 | .074 | 1.495 |
|  | Size: M-Health adoption is appropriate only when the hospital has substantial volume of patients and staff to justify its adoption(3) | -.929 | .725 | 1.642 | 1 | .200 | .395 | .095 | 1.635 |
|  | ICT capacity: M-Health adoption is appropriate when hospitals have a very complex ICT infrastructure |  |  | 4.943 | 3 | .176 |  |  |  |
|  | ICT capacity: M-Health adoption is appropriate when hospitals have a very complex ICT infrastructure(1) | 1.494 | .778 | 3.691 | 1 | .055 | 4.457 | .970 | 20.470 |
|  | ICT capacity: M-Health adoption is appropriate when hospitals have a very complex ICT infrastructure(2) | .790 | .716 | 1.216 | 1 | .270 | 2.203 | .541 | 8.970 |
|  | ICT capacity: M-Health adoption is appropriate when hospitals have a very complex ICT infrastructure(3) | .428 | .740 | .334 | 1 | .563 | 1.534 | .360 | 6.546 |
|  | ICT staff : M-Health adoption is appropriate when hospitals have very knowledgeable and adequate number of staff in ICT |  |  | 1.924 | 3 | .588 |  |  |  |
|  | ICT staff : M-Health adoption is appropriate when hospitals have very knowledgeable and adequate number of staff in ICT (1) | -.950 | .755 | 1.583 | 1 | .208 | .387 | .088 | 1.698 |
|  | ICT staff : M-Health adoption is appropriate when hospitals have very knowledgeable and adequate number of staff in ICT (2) | -.644 | .593 | 1.179 | 1 | .278 | .525 | .164 | 1.679 |
|  | ICT staff : M-Health adoption is appropriate when hospitals have very knowledgeable and adequate number of staff in ICT (3) | -.672 | .567 | 1.404 | 1 | .236 | .511 | .168 | 1.552 |
|  | Scope of the Market: M-Health adoption is appropriate for hospitals with larger market scopes such as national, regional or global markets. |  |  | 2.261 | 3 | .520 |  |  |  |
|  | Scope of the Market: M-Health adoption is appropriate for hospitals with larger market scopes such as national, regional or global markets.(1) | .951 | .699 | 1.851 | 1 | .174 | 2.587 | .658 | 10.176 |
|  | Scope of the Market: M-Health adoption is appropriate for hospitals with larger market scopes such as national, regional or global markets.(2) | .705 | .628 | 1.261 | 1 | .261 | 2.024 | .591 | 6.928 |
|  | Scope of the Market: M-Health adoption is appropriate for hospitals with larger market scopes such as national, regional or global markets.(3) | .270 | .660 | .168 | 1 | .682 | 1.310 | .360 | 4.772 |
|  | Slack/Financial Resources: M-health adoption is appropriate for hospitals that have excess budgets to invest in new IT technologies (soft and hard ware) such as M-Health |  |  | 4.484 | 3 | .214 |  |  |  |
|  | Slack/Financial Resources: M-health adoption is appropriate for hospitals that have excess budgets to invest in new IT technologies (soft and hard ware) such as M-Health(1) | -.331 | .768 | .186 | 1 | .667 | .718 | .160 | 3.235 |
|  | Slack/Financial Resources: M-health adoption is appropriate for hospitals that have excess budgets to invest in new IT technologies (soft and hard ware) such as M-Health(2) | -.415 | .749 | .307 | 1 | .580 | .661 | .152 | 2.865 |
|  | Slack/Financial Resources: M-health adoption is appropriate for hospitals that have excess budgets to invest in new IT technologies (soft and hard ware) such as M-Health(3) | .569 | .729 | .610 | 1 | .435 | 1.767 | .424 | 7.369 |
|  | Technology leadership: M-Health is appropriate for hospitals that pursue market growth through technology leadership |  |  | 1.660 | 3 | .646 |  |  |  |
|  | Technology leadership: M-Health is appropriate for hospitals that pursue market growth through technology leadership (1) | .651 | .625 | 1.083 | 1 | .298 | 1.917 | .563 | 6.533 |
|  | Technology leadership: M-Health is appropriate for hospitals that pursue market growth through technology leadership (2) | .579 | .524 | 1.223 | 1 | .269 | 1.784 | .639 | 4.978 |
|  | Technology leadership: M-Health is appropriate for hospitals that pursue market growth through technology leadership (3) | .284 | .477 | .356 | 1 | .551 | 1.329 | .522 | 3.383 |
|  | Constant | .421 | .763 | .305 | 1 | .581 | 1.524 |  |  |
| a. Variable(s) entered on step 1: Decision making structure: decision to adopt or not to adopt M-Health adoption is the prerogative of the hospital’s top management only., Size: M-Health adoption is appropriate only when the hospital has substantial volume of patients and staff to justify its adoption, ICT capacity: M-Health adoption is appropriate when hospitals have a very complex ICT infrastructure, ICT staff : M-Health adoption is appropriate when hospitals have very knowledgeable and adequate number of staff in ICT , Scope of the Market: M-Health adoption is appropriate for hospitals with larger market scopes such as national, regional or global markets., Slack/Financial Resources: M-health adoption is appropriate for hospitals that have excess budgets to invest in new IT technologies (soft and hard ware) such as M-Health, Technology leadership: M-Health is appropriate for hospitals that pursue market growth through technology leadership . | | | | | | | | | |

1. **Awareness raising over health issues technology Adoption and organizational determinants**

**Logistic Regression**

| **Notes** | | |
| --- | --- | --- |
| Output Created | | 15-JUL-2018 15:01:08 |
| Comments | |  |
| Input | Data | C:\Users\Ocholap\Downloads\m-health data.- 13-JULY 2018.sav |
|  | Active Dataset | DataSet1 |
|  | Filter | <none> |
|  | Weight | <none> |
|  | Split File | <none> |
|  | N of Rows in Working Data File | 211 |
| Missing Value Handling | Definition of Missing | User-defined missing values are treated as missing |
| Syntax | | LOGISTIC REGRESSION VARIABLES Q20.6  /METHOD=ENTER Q24.1 Q24.2 Q24.3 Q24.4 Q24.5 Q24.6 Q24.7  /CONTRAST (Q24.1)=Indicator  /CONTRAST (Q24.2)=Indicator  /CONTRAST (Q24.3)=Indicator  /CONTRAST (Q24.4)=Indicator  /CONTRAST (Q24.5)=Indicator  /CONTRAST (Q24.6)=Indicator  /CONTRAST (Q24.7)=Indicator  /SAVE=PGROUP  /PRINT=GOODFIT SUMMARY CI(95)  /CRITERIA=PIN(0.05) POUT(0.10) ITERATE(20) CUT(0.5). |
| Resources | Processor Time | 00:00:00.05 |
|  | Elapsed Time | 00:00:00.05 |
| Variables Created or Modified | PGR_33 | Predicted group |

| **Case Processing Summary** | | | |
| --- | --- | --- | --- |
| Unweighted Cases^a^ | | N | Percent |
| Selected Cases | Included in Analysis | 202 | 95.7 |
|  | Missing Cases | 9 | 4.3 |
|  | Total | 211 | 100.0 |
| Unselected Cases | | 0 | .0 |
| Total | | 211 | 100.0 |
| a. If weight is in effect, see classification table for the total number of cases. | | | |

| **Dependent Variable Encoding** | |
| --- | --- |
| Original Value | Internal Value |
| Not adopted | 0 |
| Adopted | 1 |

| **Categorical Variables Codings** | | | | | |
| --- | --- | --- | --- | --- | --- |
|  | | Frequency | Parameter coding | | |
|  |  |  | (1) | (2) | (3) |
| Technology leadership: M-Health is appropriate for hospitals that pursue market growth through technology leadership | Strongly disgree | 29 | 1.000 | .000 | .000 |
|  | Disagree | 46 | .000 | 1.000 | .000 |
|  | Agree | 75 | .000 | .000 | 1.000 |
|  | Strongly Agree | 52 | .000 | .000 | .000 |
| Size: M-Health adoption is appropriate only when the hospital has substantial volume of patients and staff to justify its adoption | Strongly disgree | 57 | 1.000 | .000 | .000 |
|  | Disagree | 79 | .000 | 1.000 | .000 |
|  | Agree | 48 | .000 | .000 | 1.000 |
|  | Strongly Agree | 18 | .000 | .000 | .000 |
| ICT capacity: M-Health adoption is appropriate when hospitals have a very complex ICT infrastructure | Strongly disgree | 60 | 1.000 | .000 | .000 |
|  | Disagree | 92 | .000 | 1.000 | .000 |
|  | Agree | 26 | .000 | .000 | 1.000 |
|  | Strongly Agree | 24 | .000 | .000 | .000 |
| ICT staff : M-Health adoption is appropriate when hospitals have very knowledgeable and adequate number of staff in ICT | Strongly disgree | 25 | 1.000 | .000 | .000 |
|  | Disagree | 67 | .000 | 1.000 | .000 |
|  | Agree | 71 | .000 | .000 | 1.000 |
|  | Strongly Agree | 39 | .000 | .000 | .000 |
| Scope of the Market: M-Health adoption is appropriate for hospitals with larger market scopes such as national, regional or global markets. | Strongly disgree | 53 | 1.000 | .000 | .000 |
|  | Disagree | 86 | .000 | 1.000 | .000 |
|  | Agree | 38 | .000 | .000 | 1.000 |
|  | Strongly Agree | 25 | .000 | .000 | .000 |
| Slack/Financial Resources: M-health adoption is appropriate for hospitals that have excess budgets to invest in new IT technologies (soft and hard ware) such as M-Health | Strongly disgree | 70 | 1.000 | .000 | .000 |
|  | Disagree | 74 | .000 | 1.000 | .000 |
|  | Agree | 44 | .000 | .000 | 1.000 |
|  | Strongly Agree | 14 | .000 | .000 | .000 |
| Decision making structure: decision to adopt or not to adopt M-Health adoption is the prerogative of the hospital’s top management only. | Strongly disgree | 55 | 1.000 | .000 | .000 |
|  | Disagree | 74 | .000 | 1.000 | .000 |
|  | Agree | 42 | .000 | .000 | 1.000 |
|  | Strongly Agree | 31 | .000 | .000 | .000 |

**Block 0: Beginning Block**

| **Classification Table^a,b^** | | | | | |
| --- | --- | --- | --- | --- | --- |
|  | Observed | | Predicted | | |
|  |  |  | Awareness raising over health issues | | Percentage Correct |
|  |  |  | Not adopted | Adopted |  |
| Step 0 | Awareness raising over health issues | Not adopted | 0 | 89 | .0 |
|  |  | Adopted | 0 | 113 | 100.0 |
|  | Overall Percentage | |  |  | 55.9 |
| a. Constant is included in the model. | | | | | |
| b. The cut value is .500 | | | | | |

| **Variables in the Equation** | | | | | | | |
| --- | --- | --- | --- | --- | --- | --- | --- |
|  | | B | S.E. | Wald | df | Sig. | Exp(B) |
| Step 0 | Constant | .239 | .142 | 2.838 | 1 | .092 | 1.270 |

| **Variables not in the Equation** | | | | | |
| --- | --- | --- | --- | --- | --- |
|  | | | Score | df | Sig. |
| Step 0 | Variables | Decision making structure: decision to adopt or not to adopt M-Health adoption is the prerogative of the hospital’s top management only. | 4.003 | 3 | .261 |
|  |  | Decision making structure: decision to adopt or not to adopt M-Health adoption is the prerogative of the hospital’s top management only.(1) | .060 | 1 | .807 |
|  |  | Decision making structure: decision to adopt or not to adopt M-Health adoption is the prerogative of the hospital’s top management only.(2) | .497 | 1 | .481 |
|  |  | Decision making structure: decision to adopt or not to adopt M-Health adoption is the prerogative of the hospital’s top management only.(3) | 3.696 | 1 | .055 |
|  |  | Size: M-Health adoption is appropriate only when the hospital has substantial volume of patients and staff to justify its adoption | 8.871 | 3 | .031 |
|  |  | Size: M-Health adoption is appropriate only when the hospital has substantial volume of patients and staff to justify its adoption(1) | 6.528 | 1 | .011 |
|  |  | Size: M-Health adoption is appropriate only when the hospital has substantial volume of patients and staff to justify its adoption(2) | 4.364 | 1 | .037 |
|  |  | Size: M-Health adoption is appropriate only when the hospital has substantial volume of patients and staff to justify its adoption(3) | .902 | 1 | .342 |
|  |  | ICT capacity: M-Health adoption is appropriate when hospitals have a very complex ICT infrastructure | 2.496 | 3 | .476 |
|  |  | ICT capacity: M-Health adoption is appropriate when hospitals have a very complex ICT infrastructure(1) | 1.135 | 1 | .287 |
|  |  | ICT capacity: M-Health adoption is appropriate when hospitals have a very complex ICT infrastructure(2) | .492 | 1 | .483 |
|  |  | ICT capacity: M-Health adoption is appropriate when hospitals have a very complex ICT infrastructure(3) | 1.160 | 1 | .282 |
|  |  | ICT staff : M-Health adoption is appropriate when hospitals have very knowledgeable and adequate number of staff in ICT | 2.841 | 3 | .417 |
|  |  | ICT staff : M-Health adoption is appropriate when hospitals have very knowledgeable and adequate number of staff in ICT (1) | 1.683 | 1 | .194 |
|  |  | ICT staff : M-Health adoption is appropriate when hospitals have very knowledgeable and adequate number of staff in ICT (2) | 1.819 | 1 | .177 |
|  |  | ICT staff : M-Health adoption is appropriate when hospitals have very knowledgeable and adequate number of staff in ICT (3) | .007 | 1 | .933 |
|  |  | Scope of the Market: M-Health adoption is appropriate for hospitals with larger market scopes such as national, regional or global markets. | .839 | 3 | .840 |
|  |  | Scope of the Market: M-Health adoption is appropriate for hospitals with larger market scopes such as national, regional or global markets.(1) | .190 | 1 | .663 |
|  |  | Scope of the Market: M-Health adoption is appropriate for hospitals with larger market scopes such as national, regional or global markets.(2) | .001 | 1 | .975 |
|  |  | Scope of the Market: M-Health adoption is appropriate for hospitals with larger market scopes such as national, regional or global markets.(3) | .073 | 1 | .788 |
|  |  | Slack/Financial Resources: M-health adoption is appropriate for hospitals that have excess budgets to invest in new IT technologies (soft and hard ware) such as M-Health | .718 | 3 | .869 |
|  |  | Slack/Financial Resources: M-health adoption is appropriate for hospitals that have excess budgets to invest in new IT technologies (soft and hard ware) such as M-Health(1) | .119 | 1 | .730 |
|  |  | Slack/Financial Resources: M-health adoption is appropriate for hospitals that have excess budgets to invest in new IT technologies (soft and hard ware) such as M-Health(2) | .169 | 1 | .681 |
|  |  | Slack/Financial Resources: M-health adoption is appropriate for hospitals that have excess budgets to invest in new IT technologies (soft and hard ware) such as M-Health(3) | .671 | 1 | .413 |
|  |  | Technology leadership: M-Health is appropriate for hospitals that pursue market growth through technology leadership | 2.015 | 3 | .569 |
|  |  | Technology leadership: M-Health is appropriate for hospitals that pursue market growth through technology leadership (1) | 1.260 | 1 | .262 |
|  |  | Technology leadership: M-Health is appropriate for hospitals that pursue market growth through technology leadership (2) | .183 | 1 | .668 |
|  |  | Technology leadership: M-Health is appropriate for hospitals that pursue market growth through technology leadership (3) | .079 | 1 | .779 |
|  | Overall Statistics | | 26.987 | 21 | .171 |

**Block 1: Method = Enter**

| **Omnibus Tests of Model Coefficients** | | | | |
| --- | --- | --- | --- | --- |
|  | | Chi-square | df | Sig. |
| Step 1 | Step | 28.817 | 21 | .118 |
|  | Block | 28.817 | 21 | .118 |
|  | Model | 28.817 | 21 | .118 |

| **Model Summary** | | | |
| --- | --- | --- | --- |
| Step | -2 Log likelihood | Cox & Snell R Square | Nagelkerke R Square |
| 1 | 248.356^a^ | .133 | .178 |
| a. Estimation terminated at iteration number 4 because parameter estimates changed by less than .001. | | | |

| **Hosmer and Lemeshow Test** | | | |
| --- | --- | --- | --- |
| Step | Chi-square | df | Sig. |
| 1 | 8.757 | 8 | .363 |

| **Contingency Table for Hosmer and Lemeshow Test** | | | | | | |
| --- | --- | --- | --- | --- | --- | --- |
|  | | Awareness raising over health issues = Not adopted | | Awareness raising over health issues = Adopted | | Total |
|  |  | Observed | Expected | Observed | Expected |  |
| Step 1 | 1 | 16 | 14.984 | 4 | 5.016 | 20 |
|  | 2 | 12 | 13.152 | 8 | 6.848 | 20 |
|  | 3 | 9 | 11.915 | 11 | 8.085 | 20 |
|  | 4 | 11 | 10.377 | 9 | 9.623 | 20 |
|  | 5 | 11 | 9.309 | 9 | 10.691 | 20 |
|  | 6 | 9 | 6.795 | 8 | 10.205 | 17 |
|  | 7 | 7 | 7.215 | 13 | 12.785 | 20 |
|  | 8 | 4 | 6.063 | 16 | 13.937 | 20 |
|  | 9 | 8 | 5.074 | 12 | 14.926 | 20 |
|  | 10 | 2 | 4.116 | 23 | 20.884 | 25 |

| **Classification Table^a^** | | | | | |
| --- | --- | --- | --- | --- | --- |
|  | Observed | | Predicted | | |
|  |  |  | Awareness raising over health issues | | Percentage Correct |
|  |  |  | Not adopted | Adopted |  |
| Step 1 | Awareness raising over health issues | Not adopted | 50 | 39 | 56.2 |
|  |  | Adopted | 33 | 80 | 70.8 |
|  | Overall Percentage | |  |  | 64.4 |
| a. The cut value is .500 | | | | | |

| **Variables in the Equation** | | | | | | | | | |
| --- | --- | --- | --- | --- | --- | --- | --- | --- | --- |
|  | | B | S.E. | Wald | df | Sig. | Exp(B) | 95% C.I.for EXP(B) | |
|  |  |  |  |  |  |  |  | Lower | Upper |
| Step 1^a^ | Decision making structure: decision to adopt or not to adopt M-Health adoption is the prerogative of the hospital’s top management only. |  |  | 8.914 | 3 | .030 |  |  |  |
|  | Decision making structure: decision to adopt or not to adopt M-Health adoption is the prerogative of the hospital’s top management only.(1) | .101 | .600 | .028 | 1 | .866 | 1.106 | .342 | 3.583 |
|  | Decision making structure: decision to adopt or not to adopt M-Health adoption is the prerogative of the hospital’s top management only.(2) | .661 | .542 | 1.485 | 1 | .223 | 1.936 | .669 | 5.604 |
|  | Decision making structure: decision to adopt or not to adopt M-Health adoption is the prerogative of the hospital’s top management only.(3) | 1.597 | .618 | 6.684 | 1 | .010 | 4.939 | 1.472 | 16.578 |
|  | Size: M-Health adoption is appropriate only when the hospital has substantial volume of patients and staff to justify its adoption |  |  | 11.555 | 3 | .009 |  |  |  |
|  | Size: M-Health adoption is appropriate only when the hospital has substantial volume of patients and staff to justify its adoption(1) | .193 | .753 | .066 | 1 | .797 | 1.213 | .277 | 5.311 |
|  | Size: M-Health adoption is appropriate only when the hospital has substantial volume of patients and staff to justify its adoption(2) | -1.287 | .750 | 2.949 | 1 | .086 | .276 | .064 | 1.199 |
|  | Size: M-Health adoption is appropriate only when the hospital has substantial volume of patients and staff to justify its adoption(3) | -1.254 | .713 | 3.091 | 1 | .079 | .285 | .070 | 1.155 |
|  | ICT capacity: M-Health adoption is appropriate when hospitals have a very complex ICT infrastructure |  |  | 2.361 | 3 | .501 |  |  |  |
|  | ICT capacity: M-Health adoption is appropriate when hospitals have a very complex ICT infrastructure(1) | .132 | .753 | .031 | 1 | .861 | 1.141 | .261 | 4.991 |
|  | ICT capacity: M-Health adoption is appropriate when hospitals have a very complex ICT infrastructure(2) | .095 | .714 | .018 | 1 | .894 | 1.099 | .271 | 4.457 |
|  | ICT capacity: M-Health adoption is appropriate when hospitals have a very complex ICT infrastructure(3) | -.690 | .740 | .870 | 1 | .351 | .501 | .118 | 2.139 |
|  | ICT staff : M-Health adoption is appropriate when hospitals have very knowledgeable and adequate number of staff in ICT |  |  | 1.597 | 3 | .660 |  |  |  |
|  | ICT staff : M-Health adoption is appropriate when hospitals have very knowledgeable and adequate number of staff in ICT (1) | -.043 | .742 | .003 | 1 | .954 | .958 | .224 | 4.099 |
|  | ICT staff : M-Health adoption is appropriate when hospitals have very knowledgeable and adequate number of staff in ICT (2) | -.559 | .575 | .943 | 1 | .331 | .572 | .185 | 1.766 |
|  | ICT staff : M-Health adoption is appropriate when hospitals have very knowledgeable and adequate number of staff in ICT (3) | -.138 | .551 | .063 | 1 | .802 | .871 | .296 | 2.563 |
|  | Scope of the Market: M-Health adoption is appropriate for hospitals with larger market scopes such as national, regional or global markets. |  |  | .807 | 3 | .848 |  |  |  |
|  | Scope of the Market: M-Health adoption is appropriate for hospitals with larger market scopes such as national, regional or global markets.(1) | .416 | .686 | .368 | 1 | .544 | 1.516 | .395 | 5.810 |
|  | Scope of the Market: M-Health adoption is appropriate for hospitals with larger market scopes such as national, regional or global markets.(2) | .542 | .621 | .761 | 1 | .383 | 1.719 | .509 | 5.807 |
|  | Scope of the Market: M-Health adoption is appropriate for hospitals with larger market scopes such as national, regional or global markets.(3) | .298 | .655 | .207 | 1 | .649 | 1.347 | .373 | 4.859 |
|  | Slack/Financial Resources: M-health adoption is appropriate for hospitals that have excess budgets to invest in new IT technologies (soft and hard ware) such as M-Health |  |  | 3.075 | 3 | .380 |  |  |  |
|  | Slack/Financial Resources: M-health adoption is appropriate for hospitals that have excess budgets to invest in new IT technologies (soft and hard ware) such as M-Health(1) | -.662 | .759 | .762 | 1 | .383 | .516 | .117 | 2.281 |
|  | Slack/Financial Resources: M-health adoption is appropriate for hospitals that have excess budgets to invest in new IT technologies (soft and hard ware) such as M-Health(2) | -.389 | .740 | .277 | 1 | .599 | .678 | .159 | 2.891 |
|  | Slack/Financial Resources: M-health adoption is appropriate for hospitals that have excess budgets to invest in new IT technologies (soft and hard ware) such as M-Health(3) | .220 | .723 | .093 | 1 | .761 | 1.246 | .302 | 5.136 |
|  | Technology leadership: M-Health is appropriate for hospitals that pursue market growth through technology leadership |  |  | 2.111 | 3 | .550 |  |  |  |
|  | Technology leadership: M-Health is appropriate for hospitals that pursue market growth through technology leadership (1) | .650 | .610 | 1.136 | 1 | .287 | 1.915 | .580 | 6.327 |
|  | Technology leadership: M-Health is appropriate for hospitals that pursue market growth through technology leadership (2) | .589 | .514 | 1.310 | 1 | .252 | 1.801 | .658 | 4.935 |
|  | Technology leadership: M-Health is appropriate for hospitals that pursue market growth through technology leadership (3) | .156 | .469 | .110 | 1 | .740 | 1.168 | .466 | 2.930 |
|  | Constant | .315 | .749 | .177 | 1 | .674 | 1.371 |  |  |
| a. Variable(s) entered on step 1: Decision making structure: decision to adopt or not to adopt M-Health adoption is the prerogative of the hospital’s top management only., Size: M-Health adoption is appropriate only when the hospital has substantial volume of patients and staff to justify its adoption, ICT capacity: M-Health adoption is appropriate when hospitals have a very complex ICT infrastructure, ICT staff : M-Health adoption is appropriate when hospitals have very knowledgeable and adequate number of staff in ICT , Scope of the Market: M-Health adoption is appropriate for hospitals with larger market scopes such as national, regional or global markets., Slack/Financial Resources: M-health adoption is appropriate for hospitals that have excess budgets to invest in new IT technologies (soft and hard ware) such as M-Health, Technology leadership: M-Health is appropriate for hospitals that pursue market growth through technology leadership . | | | | | | | | | |

1. **Mobile telemedicine technology Adoption and organizational determinants**

**Logistic Regression**

| **Notes** | | |
| --- | --- | --- |
| Output Created | | 15-JUL-2018 15:03:37 |
| Comments | |  |
| Input | Data | C:\Users\Ocholap\Downloads\m-health data.- 13-JULY 2018.sav |
|  | Active Dataset | DataSet1 |
|  | Filter | <none> |
|  | Weight | <none> |
|  | Split File | <none> |
|  | N of Rows in Working Data File | 211 |
| Missing Value Handling | Definition of Missing | User-defined missing values are treated as missing |
| Syntax | | LOGISTIC REGRESSION VARIABLES Q20.7  /METHOD=ENTER Q24.1 Q24.2 Q24.3 Q24.4 Q24.5 Q24.6 Q24.7  /CONTRAST (Q24.1)=Indicator  /CONTRAST (Q24.2)=Indicator  /CONTRAST (Q24.3)=Indicator  /CONTRAST (Q24.4)=Indicator  /CONTRAST (Q24.5)=Indicator  /CONTRAST (Q24.6)=Indicator  /CONTRAST (Q24.7)=Indicator  /SAVE=PGROUP  /PRINT=GOODFIT SUMMARY CI(95)  /CRITERIA=PIN(0.05) POUT(0.10) ITERATE(20) CUT(0.5). |
| Resources | Processor Time | 00:00:00.03 |
|  | Elapsed Time | 00:00:00.05 |
| Variables Created or Modified | PGR_34 | Predicted group |

| **Case Processing Summary** | | | |
| --- | --- | --- | --- |
| Unweighted Cases^a^ | | N | Percent |
| Selected Cases | Included in Analysis | 208 | 98.6 |
|  | Missing Cases | 3 | 1.4 |
|  | Total | 211 | 100.0 |
| Unselected Cases | | 0 | .0 |
| Total | | 211 | 100.0 |
| a. If weight is in effect, see classification table for the total number of cases. | | | |

| **Dependent Variable Encoding** | |
| --- | --- |
| Original Value | Internal Value |
| Not adopted | 0 |
| Adopted | 1 |

| **Categorical Variables Codings** | | | | | |
| --- | --- | --- | --- | --- | --- |
|  | | Frequency | Parameter coding | | |
|  |  |  | (1) | (2) | (3) |
| Technology leadership: M-Health is appropriate for hospitals that pursue market growth through technology leadership | Strongly disgree | 30 | 1.000 | .000 | .000 |
|  | Disagree | 49 | .000 | 1.000 | .000 |
|  | Agree | 76 | .000 | .000 | 1.000 |
|  | Strongly Agree | 53 | .000 | .000 | .000 |
| Size: M-Health adoption is appropriate only when the hospital has substantial volume of patients and staff to justify its adoption | Strongly disgree | 59 | 1.000 | .000 | .000 |
|  | Disagree | 81 | .000 | 1.000 | .000 |
|  | Agree | 49 | .000 | .000 | 1.000 |
|  | Strongly Agree | 19 | .000 | .000 | .000 |
| ICT capacity: M-Health adoption is appropriate when hospitals have a very complex ICT infrastructure | Strongly disgree | 64 | 1.000 | .000 | .000 |
|  | Disagree | 93 | .000 | 1.000 | .000 |
|  | Agree | 27 | .000 | .000 | 1.000 |
|  | Strongly Agree | 24 | .000 | .000 | .000 |
| ICT staff : M-Health adoption is appropriate when hospitals have very knowledgeable and adequate number of staff in ICT | Strongly disgree | 27 | 1.000 | .000 | .000 |
|  | Disagree | 67 | .000 | 1.000 | .000 |
|  | Agree | 75 | .000 | .000 | 1.000 |
|  | Strongly Agree | 39 | .000 | .000 | .000 |
| Scope of the Market: M-Health adoption is appropriate for hospitals with larger market scopes such as national, regional or global markets. | Strongly disgree | 56 | 1.000 | .000 | .000 |
|  | Disagree | 89 | .000 | 1.000 | .000 |
|  | Agree | 37 | .000 | .000 | 1.000 |
|  | Strongly Agree | 26 | .000 | .000 | .000 |
| Slack/Financial Resources: M-health adoption is appropriate for hospitals that have excess budgets to invest in new IT technologies (soft and hard ware) such as M-Health | Strongly disgree | 75 | 1.000 | .000 | .000 |
|  | Disagree | 76 | .000 | 1.000 | .000 |
|  | Agree | 43 | .000 | .000 | 1.000 |
|  | Strongly Agree | 14 | .000 | .000 | .000 |
| Decision making structure: decision to adopt or not to adopt M-Health adoption is the prerogative of the hospital’s top management only. | Strongly disgree | 58 | 1.000 | .000 | .000 |
|  | Disagree | 75 | .000 | 1.000 | .000 |
|  | Agree | 44 | .000 | .000 | 1.000 |
|  | Strongly Agree | 31 | .000 | .000 | .000 |

**Block 0: Beginning Block**

| **Classification Table^a,b^** | | | | | |
| --- | --- | --- | --- | --- | --- |
|  | Observed | | Predicted | | |
|  |  |  | Mobile telemedicine | | Percentage Correct |
|  |  |  | Not adopted | Adopted |  |
| Step 0 | Mobile telemedicine | Not adopted | 0 | 101 | .0 |
|  |  | Adopted | 0 | 107 | 100.0 |
|  | Overall Percentage | |  |  | 51.4 |
| a. Constant is included in the model. | | | | | |
| b. The cut value is .500 | | | | | |

| **Variables in the Equation** | | | | | | | |
| --- | --- | --- | --- | --- | --- | --- | --- |
|  | | B | S.E. | Wald | df | Sig. | Exp(B) |
| Step 0 | Constant | .058 | .139 | .173 | 1 | .677 | 1.059 |

| **Variables not in the Equation** | | | | | |
| --- | --- | --- | --- | --- | --- |
|  | | | Score | df | Sig. |
| Step 0 | Variables | Decision making structure: decision to adopt or not to adopt M-Health adoption is the prerogative of the hospital’s top management only. | 1.655 | 3 | .647 |
|  |  | Decision making structure: decision to adopt or not to adopt M-Health adoption is the prerogative of the hospital’s top management only.(1) | .770 | 1 | .380 |
|  |  | Decision making structure: decision to adopt or not to adopt M-Health adoption is the prerogative of the hospital’s top management only.(2) | .488 | 1 | .485 |
|  |  | Decision making structure: decision to adopt or not to adopt M-Health adoption is the prerogative of the hospital’s top management only.(3) | .308 | 1 | .579 |
|  |  | Size: M-Health adoption is appropriate only when the hospital has substantial volume of patients and staff to justify its adoption | .486 | 3 | .922 |
|  |  | Size: M-Health adoption is appropriate only when the hospital has substantial volume of patients and staff to justify its adoption(1) | .040 | 1 | .842 |
|  |  | Size: M-Health adoption is appropriate only when the hospital has substantial volume of patients and staff to justify its adoption(2) | .225 | 1 | .635 |
|  |  | Size: M-Health adoption is appropriate only when the hospital has substantial volume of patients and staff to justify its adoption(3) | .005 | 1 | .946 |
|  |  | ICT capacity: M-Health adoption is appropriate when hospitals have a very complex ICT infrastructure | .454 | 3 | .929 |
|  |  | ICT capacity: M-Health adoption is appropriate when hospitals have a very complex ICT infrastructure(1) | .334 | 1 | .563 |
|  |  | ICT capacity: M-Health adoption is appropriate when hospitals have a very complex ICT infrastructure(2) | .363 | 1 | .547 |
|  |  | ICT capacity: M-Health adoption is appropriate when hospitals have a very complex ICT infrastructure(3) | .002 | 1 | .964 |
|  |  | ICT staff : M-Health adoption is appropriate when hospitals have very knowledgeable and adequate number of staff in ICT | 5.997 | 3 | .112 |
|  |  | ICT staff : M-Health adoption is appropriate when hospitals have very knowledgeable and adequate number of staff in ICT (1) | 5.910 | 1 | .015 |
|  |  | ICT staff : M-Health adoption is appropriate when hospitals have very knowledgeable and adequate number of staff in ICT (2) | .207 | 1 | .649 |
|  |  | ICT staff : M-Health adoption is appropriate when hospitals have very knowledgeable and adequate number of staff in ICT (3) | .975 | 1 | .323 |
|  |  | Scope of the Market: M-Health adoption is appropriate for hospitals with larger market scopes such as national, regional or global markets. | 4.994 | 3 | .172 |
|  |  | Scope of the Market: M-Health adoption is appropriate for hospitals with larger market scopes such as national, regional or global markets.(1) | 1.719 | 1 | .190 |
|  |  | Scope of the Market: M-Health adoption is appropriate for hospitals with larger market scopes such as national, regional or global markets.(2) | 4.763 | 1 | .029 |
|  |  | Scope of the Market: M-Health adoption is appropriate for hospitals with larger market scopes such as national, regional or global markets.(3) | 1.158 | 1 | .282 |
|  |  | Slack/Financial Resources: M-health adoption is appropriate for hospitals that have excess budgets to invest in new IT technologies (soft and hard ware) such as M-Health | 6.380 | 3 | .095 |
|  |  | Slack/Financial Resources: M-health adoption is appropriate for hospitals that have excess budgets to invest in new IT technologies (soft and hard ware) such as M-Health(1) | .556 | 1 | .456 |
|  |  | Slack/Financial Resources: M-health adoption is appropriate for hospitals that have excess budgets to invest in new IT technologies (soft and hard ware) such as M-Health(2) | .365 | 1 | .546 |
|  |  | Slack/Financial Resources: M-health adoption is appropriate for hospitals that have excess budgets to invest in new IT technologies (soft and hard ware) such as M-Health(3) | 5.555 | 1 | .018 |
|  |  | Technology leadership: M-Health is appropriate for hospitals that pursue market growth through technology leadership | 5.262 | 3 | .154 |
|  |  | Technology leadership: M-Health is appropriate for hospitals that pursue market growth through technology leadership (1) | 4.602 | 1 | .032 |
|  |  | Technology leadership: M-Health is appropriate for hospitals that pursue market growth through technology leadership (2) | .067 | 1 | .795 |
|  |  | Technology leadership: M-Health is appropriate for hospitals that pursue market growth through technology leadership (3) | 1.996 | 1 | .158 |
|  | Overall Statistics | | 30.848 | 21 | .076 |

**Block 1: Method = Enter**

| **Omnibus Tests of Model Coefficients** | | | | |
| --- | --- | --- | --- | --- |
|  | | Chi-square | df | Sig. |
| Step 1 | Step | 33.330 | 21 | .043 |
|  | Block | 33.330 | 21 | .043 |
|  | Model | 33.330 | 21 | .043 |

| **Model Summary** | | | |
| --- | --- | --- | --- |
| Step | -2 Log likelihood | Cox & Snell R Square | Nagelkerke R Square |
| 1 | 254.846^a^ | .148 | .197 |
| a. Estimation terminated at iteration number 4 because parameter estimates changed by less than .001. | | | |

| **Hosmer and Lemeshow Test** | | | |
| --- | --- | --- | --- |
| Step | Chi-square | df | Sig. |
| 1 | 7.017 | 8 | .535 |

| **Contingency Table for Hosmer and Lemeshow Test** | | | | | | |
| --- | --- | --- | --- | --- | --- | --- |
|  | | Mobile telemedicine = Not adopted | | Mobile telemedicine = Adopted | | Total |
|  |  | Observed | Expected | Observed | Expected |  |
| Step 1 | 1 | 15 | 16.943 | 6 | 4.057 | 21 |
|  | 2 | 18 | 15.325 | 3 | 5.675 | 21 |
|  | 3 | 14 | 13.360 | 7 | 7.640 | 21 |
|  | 4 | 11 | 12.342 | 11 | 9.658 | 22 |
|  | 5 | 9 | 10.665 | 13 | 11.335 | 22 |
|  | 6 | 9 | 8.974 | 12 | 12.026 | 21 |
|  | 7 | 11 | 8.196 | 10 | 12.804 | 21 |
|  | 8 | 5 | 7.019 | 16 | 13.981 | 21 |
|  | 9 | 7 | 5.490 | 14 | 15.510 | 21 |
|  | 10 | 2 | 2.685 | 15 | 14.315 | 17 |

| **Classification Table^a^** | | | | | |
| --- | --- | --- | --- | --- | --- |
|  | Observed | | Predicted | | |
|  |  |  | Mobile telemedicine | | Percentage Correct |
|  |  |  | Not adopted | Adopted |  |
| Step 1 | Mobile telemedicine | Not adopted | 60 | 41 | 59.4 |
|  |  | Adopted | 31 | 76 | 71.0 |
|  | Overall Percentage | |  |  | 65.4 |
| a. The cut value is .500 | | | | | |

| **Variables in the Equation** | | | | | | | | | |
| --- | --- | --- | --- | --- | --- | --- | --- | --- | --- |
|  | | B | S.E. | Wald | df | Sig. | Exp(B) | 95% C.I.for EXP(B) | |
|  |  |  |  |  |  |  |  | Lower | Upper |
| Step 1^a^ | Decision making structure: decision to adopt or not to adopt M-Health adoption is the prerogative of the hospital’s top management only. |  |  | 2.634 | 3 | .452 |  |  |  |
|  | Decision making structure: decision to adopt or not to adopt M-Health adoption is the prerogative of the hospital’s top management only.(1) | -.726 | .599 | 1.470 | 1 | .225 | .484 | .150 | 1.565 |
|  | Decision making structure: decision to adopt or not to adopt M-Health adoption is the prerogative of the hospital’s top management only.(2) | -.082 | .549 | .022 | 1 | .882 | .922 | .314 | 2.705 |
|  | Decision making structure: decision to adopt or not to adopt M-Health adoption is the prerogative of the hospital’s top management only.(3) | -.428 | .593 | .522 | 1 | .470 | .652 | .204 | 2.083 |
|  | Size: M-Health adoption is appropriate only when the hospital has substantial volume of patients and staff to justify its adoption |  |  | 2.530 | 3 | .470 |  |  |  |
|  | Size: M-Health adoption is appropriate only when the hospital has substantial volume of patients and staff to justify its adoption(1) | -.410 | .723 | .322 | 1 | .570 | .663 | .161 | 2.738 |
|  | Size: M-Health adoption is appropriate only when the hospital has substantial volume of patients and staff to justify its adoption(2) | -.983 | .734 | 1.795 | 1 | .180 | .374 | .089 | 1.577 |
|  | Size: M-Health adoption is appropriate only when the hospital has substantial volume of patients and staff to justify its adoption(3) | -.765 | .688 | 1.236 | 1 | .266 | .465 | .121 | 1.792 |
|  | ICT capacity: M-Health adoption is appropriate when hospitals have a very complex ICT infrastructure |  |  | 2.906 | 3 | .406 |  |  |  |
|  | ICT capacity: M-Health adoption is appropriate when hospitals have a very complex ICT infrastructure(1) | 1.041 | .752 | 1.918 | 1 | .166 | 2.832 | .649 | 12.360 |
|  | ICT capacity: M-Health adoption is appropriate when hospitals have a very complex ICT infrastructure(2) | 1.226 | .726 | 2.853 | 1 | .091 | 3.408 | .821 | 14.144 |
|  | ICT capacity: M-Health adoption is appropriate when hospitals have a very complex ICT infrastructure(3) | .825 | .754 | 1.197 | 1 | .274 | 2.282 | .521 | 9.998 |
|  | ICT staff : M-Health adoption is appropriate when hospitals have very knowledgeable and adequate number of staff in ICT |  |  | 7.776 | 3 | .051 |  |  |  |
|  | ICT staff : M-Health adoption is appropriate when hospitals have very knowledgeable and adequate number of staff in ICT (1) | -1.827 | .728 | 6.308 | 1 | .012 | .161 | .039 | .669 |
|  | ICT staff : M-Health adoption is appropriate when hospitals have very knowledgeable and adequate number of staff in ICT (2) | -.438 | .587 | .558 | 1 | .455 | .645 | .204 | 2.037 |
|  | ICT staff : M-Health adoption is appropriate when hospitals have very knowledgeable and adequate number of staff in ICT (3) | -.280 | .558 | .252 | 1 | .616 | .756 | .253 | 2.257 |
|  | Scope of the Market: M-Health adoption is appropriate for hospitals with larger market scopes such as national, regional or global markets. |  |  | 12.587 | 3 | .006 |  |  |  |
|  | Scope of the Market: M-Health adoption is appropriate for hospitals with larger market scopes such as national, regional or global markets.(1) | .508 | .675 | .566 | 1 | .452 | 1.661 | .443 | 6.236 |
|  | Scope of the Market: M-Health adoption is appropriate for hospitals with larger market scopes such as national, regional or global markets.(2) | -1.201 | .621 | 3.743 | 1 | .053 | .301 | .089 | 1.016 |
|  | Scope of the Market: M-Health adoption is appropriate for hospitals with larger market scopes such as national, regional or global markets.(3) | -.971 | .667 | 2.120 | 1 | .145 | .379 | .102 | 1.399 |
|  | Slack/Financial Resources: M-health adoption is appropriate for hospitals that have excess budgets to invest in new IT technologies (soft and hard ware) such as M-Health |  |  | 6.092 | 3 | .107 |  |  |  |
|  | Slack/Financial Resources: M-health adoption is appropriate for hospitals that have excess budgets to invest in new IT technologies (soft and hard ware) such as M-Health(1) | .650 | .748 | .755 | 1 | .385 | 1.915 | .442 | 8.293 |
|  | Slack/Financial Resources: M-health adoption is appropriate for hospitals that have excess budgets to invest in new IT technologies (soft and hard ware) such as M-Health(2) | .984 | .743 | 1.755 | 1 | .185 | 2.675 | .624 | 11.471 |
|  | Slack/Financial Resources: M-health adoption is appropriate for hospitals that have excess budgets to invest in new IT technologies (soft and hard ware) such as M-Health(3) | 1.601 | .738 | 4.712 | 1 | .030 | 4.960 | 1.168 | 21.060 |
|  | Technology leadership: M-Health is appropriate for hospitals that pursue market growth through technology leadership |  |  | 5.644 | 3 | .130 |  |  |  |
|  | Technology leadership: M-Health is appropriate for hospitals that pursue market growth through technology leadership (1) | -.401 | .604 | .440 | 1 | .507 | .670 | .205 | 2.189 |
|  | Technology leadership: M-Health is appropriate for hospitals that pursue market growth through technology leadership (2) | .263 | .517 | .257 | 1 | .612 | 1.300 | .472 | 3.585 |
|  | Technology leadership: M-Health is appropriate for hospitals that pursue market growth through technology leadership (3) | .800 | .482 | 2.753 | 1 | .097 | 2.226 | .865 | 5.731 |
|  | Constant | -.102 | .743 | .019 | 1 | .891 | .903 |  |  |
| a. Variable(s) entered on step 1: Decision making structure: decision to adopt or not to adopt M-Health adoption is the prerogative of the hospital’s top management only., Size: M-Health adoption is appropriate only when the hospital has substantial volume of patients and staff to justify its adoption, ICT capacity: M-Health adoption is appropriate when hospitals have a very complex ICT infrastructure, ICT staff : M-Health adoption is appropriate when hospitals have very knowledgeable and adequate number of staff in ICT , Scope of the Market: M-Health adoption is appropriate for hospitals with larger market scopes such as national, regional or global markets., Slack/Financial Resources: M-health adoption is appropriate for hospitals that have excess budgets to invest in new IT technologies (soft and hard ware) such as M-Health, Technology leadership: M-Health is appropriate for hospitals that pursue market growth through technology leadership . | | | | | | | | | |

1. **Mobile health surveys (surveys by mobile phone) technology Adoption and organizational determinants**

**Logistic Regression**

| **Notes** | | |
| --- | --- | --- |
| Output Created | | 15-JUL-2018 15:05:32 |
| Comments | |  |
| Input | Data | C:\Users\Ocholap\Downloads\m-health data.- 13-JULY 2018.sav |
|  | Active Dataset | DataSet1 |
|  | Filter | <none> |
|  | Weight | <none> |
|  | Split File | <none> |
|  | N of Rows in Working Data File | 211 |
| Missing Value Handling | Definition of Missing | User-defined missing values are treated as missing |
| Syntax | | LOGISTIC REGRESSION VARIABLES Q20.8  /METHOD=ENTER Q24.1 Q24.2 Q24.3 Q24.4 Q24.5 Q24.6 Q24.7  /CONTRAST (Q24.1)=Indicator  /CONTRAST (Q24.2)=Indicator  /CONTRAST (Q24.3)=Indicator  /CONTRAST (Q24.4)=Indicator  /CONTRAST (Q24.5)=Indicator  /CONTRAST (Q24.6)=Indicator  /CONTRAST (Q24.7)=Indicator  /SAVE=PGROUP  /PRINT=GOODFIT SUMMARY CI(95)  /CRITERIA=PIN(0.05) POUT(0.10) ITERATE(20) CUT(0.5). |
| Resources | Processor Time | 00:00:00.05 |
|  | Elapsed Time | 00:00:00.05 |
| Variables Created or Modified | PGR_35 | Predicted group |

| **Case Processing Summary** | | | |
| --- | --- | --- | --- |
| Unweighted Cases^a^ | | N | Percent |
| Selected Cases | Included in Analysis | 197 | 93.4 |
|  | Missing Cases | 14 | 6.6 |
|  | Total | 211 | 100.0 |
| Unselected Cases | | 0 | .0 |
| Total | | 211 | 100.0 |
| a. If weight is in effect, see classification table for the total number of cases. | | | |

| **Dependent Variable Encoding** | |
| --- | --- |
| Original Value | Internal Value |
| Not adopted | 0 |
| Adopted | 1 |

| **Categorical Variables Codings** | | | | | |
| --- | --- | --- | --- | --- | --- |
|  | | Frequency | Parameter coding | | |
|  |  |  | (1) | (2) | (3) |
| Technology leadership: M-Health is appropriate for hospitals that pursue market growth through technology leadership | Strongly disgree | 28 | 1.000 | .000 | .000 |
|  | Disagree | 45 | .000 | 1.000 | .000 |
|  | Agree | 74 | .000 | .000 | 1.000 |
|  | Strongly Agree | 50 | .000 | .000 | .000 |
| Size: M-Health adoption is appropriate only when the hospital has substantial volume of patients and staff to justify its adoption | Strongly disgree | 55 | 1.000 | .000 | .000 |
|  | Disagree | 74 | .000 | 1.000 | .000 |
|  | Agree | 49 | .000 | .000 | 1.000 |
|  | Strongly Agree | 19 | .000 | .000 | .000 |
| ICT capacity: M-Health adoption is appropriate when hospitals have a very complex ICT infrastructure | Strongly disgree | 58 | 1.000 | .000 | .000 |
|  | Disagree | 89 | .000 | 1.000 | .000 |
|  | Agree | 26 | .000 | .000 | 1.000 |
|  | Strongly Agree | 24 | .000 | .000 | .000 |
| ICT staff : M-Health adoption is appropriate when hospitals have very knowledgeable and adequate number of staff in ICT | Strongly disgree | 25 | 1.000 | .000 | .000 |
|  | Disagree | 64 | .000 | 1.000 | .000 |
|  | Agree | 70 | .000 | .000 | 1.000 |
|  | Strongly Agree | 38 | .000 | .000 | .000 |
| Scope of the Market: M-Health adoption is appropriate for hospitals with larger market scopes such as national, regional or global markets. | Strongly disgree | 51 | 1.000 | .000 | .000 |
|  | Disagree | 85 | .000 | 1.000 | .000 |
|  | Agree | 38 | .000 | .000 | 1.000 |
|  | Strongly Agree | 23 | .000 | .000 | .000 |
| Slack/Financial Resources: M-health adoption is appropriate for hospitals that have excess budgets to invest in new IT technologies (soft and hard ware) such as M-Health | Strongly disgree | 67 | 1.000 | .000 | .000 |
|  | Disagree | 73 | .000 | 1.000 | .000 |
|  | Agree | 43 | .000 | .000 | 1.000 |
|  | Strongly Agree | 14 | .000 | .000 | .000 |
| Decision making structure: decision to adopt or not to adopt M-Health adoption is the prerogative of the hospital’s top management only. | Strongly disgree | 54 | 1.000 | .000 | .000 |
|  | Disagree | 70 | .000 | 1.000 | .000 |
|  | Agree | 42 | .000 | .000 | 1.000 |
|  | Strongly Agree | 31 | .000 | .000 | .000 |

**Block 0: Beginning Block**

| **Classification Table^a,b^** | | | | | |
| --- | --- | --- | --- | --- | --- |
|  | Observed | | Predicted | | |
|  |  |  | Mobile surveys (surveys by mobile phone) | | Percentage Correct |
|  |  |  | Not adopted | Adopted |  |
| Step 0 | Mobile surveys (surveys by mobile phone) | Not adopted | 154 | 0 | 100.0 |
|  |  | Adopted | 43 | 0 | .0 |
|  | Overall Percentage | |  |  | 78.2 |
| a. Constant is included in the model. | | | | | |
| b. The cut value is .500 | | | | | |

| **Variables in the Equation** | | | | | | | |
| --- | --- | --- | --- | --- | --- | --- | --- |
|  | | B | S.E. | Wald | df | Sig. | Exp(B) |
| Step 0 | Constant | -1.276 | .172 | 54.709 | 1 | .000 | .279 |

| **Variables not in the Equation** | | | | | |
| --- | --- | --- | --- | --- | --- |
|  | | | Score | df | Sig. |
| Step 0 | Variables | Decision making structure: decision to adopt or not to adopt M-Health adoption is the prerogative of the hospital’s top management only. | .869 | 3 | .833 |
|  |  | Decision making structure: decision to adopt or not to adopt M-Health adoption is the prerogative of the hospital’s top management only.(1) | .220 | 1 | .639 |
|  |  | Decision making structure: decision to adopt or not to adopt M-Health adoption is the prerogative of the hospital’s top management only.(2) | .067 | 1 | .795 |
|  |  | Decision making structure: decision to adopt or not to adopt M-Health adoption is the prerogative of the hospital’s top management only.(3) | .833 | 1 | .361 |
|  |  | Size: M-Health adoption is appropriate only when the hospital has substantial volume of patients and staff to justify its adoption | 6.136 | 3 | .105 |
|  |  | Size: M-Health adoption is appropriate only when the hospital has substantial volume of patients and staff to justify its adoption(1) | .588 | 1 | .443 |
|  |  | Size: M-Health adoption is appropriate only when the hospital has substantial volume of patients and staff to justify its adoption(2) | 4.801 | 1 | .028 |
|  |  | Size: M-Health adoption is appropriate only when the hospital has substantial volume of patients and staff to justify its adoption(3) | .271 | 1 | .603 |
|  |  | ICT capacity: M-Health adoption is appropriate when hospitals have a very complex ICT infrastructure | 2.919 | 3 | .404 |
|  |  | ICT capacity: M-Health adoption is appropriate when hospitals have a very complex ICT infrastructure(1) | .257 | 1 | .612 |
|  |  | ICT capacity: M-Health adoption is appropriate when hospitals have a very complex ICT infrastructure(2) | 1.410 | 1 | .235 |
|  |  | ICT capacity: M-Health adoption is appropriate when hospitals have a very complex ICT infrastructure(3) | .118 | 1 | .731 |
|  |  | ICT staff : M-Health adoption is appropriate when hospitals have very knowledgeable and adequate number of staff in ICT | 9.382 | 3 | .025 |
|  |  | ICT staff : M-Health adoption is appropriate when hospitals have very knowledgeable and adequate number of staff in ICT (1) | .056 | 1 | .813 |
|  |  | ICT staff : M-Health adoption is appropriate when hospitals have very knowledgeable and adequate number of staff in ICT (2) | 3.350 | 1 | .067 |
|  |  | ICT staff : M-Health adoption is appropriate when hospitals have very knowledgeable and adequate number of staff in ICT (3) | .213 | 1 | .645 |
|  |  | Scope of the Market: M-Health adoption is appropriate for hospitals with larger market scopes such as national, regional or global markets. | 6.164 | 3 | .104 |
|  |  | Scope of the Market: M-Health adoption is appropriate for hospitals with larger market scopes such as national, regional or global markets.(1) | .117 | 1 | .733 |
|  |  | Scope of the Market: M-Health adoption is appropriate for hospitals with larger market scopes such as national, regional or global markets.(2) | 5.208 | 1 | .022 |
|  |  | Scope of the Market: M-Health adoption is appropriate for hospitals with larger market scopes such as national, regional or global markets.(3) | 2.624 | 1 | .105 |
|  |  | Slack/Financial Resources: M-health adoption is appropriate for hospitals that have excess budgets to invest in new IT technologies (soft and hard ware) such as M-Health | 3.430 | 3 | .330 |
|  |  | Slack/Financial Resources: M-health adoption is appropriate for hospitals that have excess budgets to invest in new IT technologies (soft and hard ware) such as M-Health(1) | .052 | 1 | .820 |
|  |  | Slack/Financial Resources: M-health adoption is appropriate for hospitals that have excess budgets to invest in new IT technologies (soft and hard ware) such as M-Health(2) | 1.974 | 1 | .160 |
|  |  | Slack/Financial Resources: M-health adoption is appropriate for hospitals that have excess budgets to invest in new IT technologies (soft and hard ware) such as M-Health(3) | 2.277 | 1 | .131 |
|  |  | Technology leadership: M-Health is appropriate for hospitals that pursue market growth through technology leadership | 1.924 | 3 | .588 |
|  |  | Technology leadership: M-Health is appropriate for hospitals that pursue market growth through technology leadership (1) | .870 | 1 | .351 |
|  |  | Technology leadership: M-Health is appropriate for hospitals that pursue market growth through technology leadership (2) | 1.345 | 1 | .246 |
|  |  | Technology leadership: M-Health is appropriate for hospitals that pursue market growth through technology leadership (3) | .003 | 1 | .957 |
|  | Overall Statistics | | 21.779 | 21 | .412 |

**Block 1: Method = Enter**

| **Omnibus Tests of Model Coefficients** | | | | |
| --- | --- | --- | --- | --- |
|  | | Chi-square | df | Sig. |
| Step 1 | Step | 21.979 | 21 | .401 |
|  | Block | 21.979 | 21 | .401 |
|  | Model | 21.979 | 21 | .401 |

| **Model Summary** | | | |
| --- | --- | --- | --- |
| Step | -2 Log likelihood | Cox & Snell R Square | Nagelkerke R Square |
| 1 | 184.758^a^ | .106 | .162 |
| a. Estimation terminated at iteration number 5 because parameter estimates changed by less than .001. | | | |

| **Hosmer and Lemeshow Test** | | | |
| --- | --- | --- | --- |
| Step | Chi-square | df | Sig. |
| 1 | 8.949 | 8 | .347 |

| **Contingency Table for Hosmer and Lemeshow Test** | | | | | | |
| --- | --- | --- | --- | --- | --- | --- |
|  | | Mobile surveys (surveys by mobile phone) = Not adopted | | Mobile surveys (surveys by mobile phone) = Adopted | | Total |
|  |  | Observed | Expected | Observed | Expected |  |
| Step 1 | 1 | 17 | 18.872 | 3 | 1.128 | 20 |
|  | 2 | 17 | 17.312 | 2 | 1.688 | 19 |
|  | 3 | 18 | 17.792 | 2 | 2.208 | 20 |
|  | 4 | 17 | 17.249 | 3 | 2.751 | 20 |
|  | 5 | 18 | 16.659 | 2 | 3.341 | 20 |
|  | 6 | 19 | 16.128 | 1 | 3.872 | 20 |
|  | 7 | 17 | 15.961 | 4 | 5.039 | 21 |
|  | 8 | 13 | 13.907 | 7 | 6.093 | 20 |
|  | 9 | 9 | 11.751 | 10 | 7.249 | 19 |
|  | 10 | 9 | 8.368 | 9 | 9.632 | 18 |

| **Classification Table^a^** | | | | | |
| --- | --- | --- | --- | --- | --- |
|  | Observed | | Predicted | | |
|  |  |  | Mobile surveys (surveys by mobile phone) | | Percentage Correct |
|  |  |  | Not adopted | Adopted |  |
| Step 1 | Mobile surveys (surveys by mobile phone) | Not adopted | 149 | 5 | 96.8 |
|  |  | Adopted | 36 | 7 | 16.3 |
|  | Overall Percentage | |  |  | 79.2 |
| a. The cut value is .500 | | | | | |

| **Variables in the Equation** | | | | | | | | | |
| --- | --- | --- | --- | --- | --- | --- | --- | --- | --- |
|  | | B | S.E. | Wald | df | Sig. | Exp(B) | 95% C.I.for EXP(B) | |
|  |  |  |  |  |  |  |  | Lower | Upper |
| Step 1^a^ | Decision making structure: decision to adopt or not to adopt M-Health adoption is the prerogative of the hospital’s top management only. |  |  | 3.273 | 3 | .351 |  |  |  |
|  | Decision making structure: decision to adopt or not to adopt M-Health adoption is the prerogative of the hospital’s top management only.(1) | .485 | .739 | .431 | 1 | .512 | 1.624 | .382 | 6.907 |
|  | Decision making structure: decision to adopt or not to adopt M-Health adoption is the prerogative of the hospital’s top management only.(2) | .985 | .677 | 2.116 | 1 | .146 | 2.677 | .710 | 10.092 |
|  | Decision making structure: decision to adopt or not to adopt M-Health adoption is the prerogative of the hospital’s top management only.(3) | .142 | .752 | .035 | 1 | .851 | 1.152 | .264 | 5.034 |
|  | Size: M-Health adoption is appropriate only when the hospital has substantial volume of patients and staff to justify its adoption |  |  | 3.313 | 3 | .346 |  |  |  |
|  | Size: M-Health adoption is appropriate only when the hospital has substantial volume of patients and staff to justify its adoption(1) | -.523 | .770 | .461 | 1 | .497 | .593 | .131 | 2.683 |
|  | Size: M-Health adoption is appropriate only when the hospital has substantial volume of patients and staff to justify its adoption(2) | -1.357 | .814 | 2.780 | 1 | .095 | .257 | .052 | 1.269 |
|  | Size: M-Health adoption is appropriate only when the hospital has substantial volume of patients and staff to justify its adoption(3) | -.556 | .738 | .567 | 1 | .451 | .574 | .135 | 2.436 |
|  | ICT capacity: M-Health adoption is appropriate when hospitals have a very complex ICT infrastructure |  |  | 2.746 | 3 | .433 |  |  |  |
|  | ICT capacity: M-Health adoption is appropriate when hospitals have a very complex ICT infrastructure(1) | .971 | .829 | 1.372 | 1 | .241 | 2.640 | .520 | 13.402 |
|  | ICT capacity: M-Health adoption is appropriate when hospitals have a very complex ICT infrastructure(2) | .683 | .787 | .754 | 1 | .385 | 1.980 | .424 | 9.258 |
|  | ICT capacity: M-Health adoption is appropriate when hospitals have a very complex ICT infrastructure(3) | -.212 | .847 | .063 | 1 | .802 | .809 | .154 | 4.257 |
|  | ICT staff : M-Health adoption is appropriate when hospitals have very knowledgeable and adequate number of staff in ICT |  |  | 5.019 | 3 | .170 |  |  |  |
|  | ICT staff : M-Health adoption is appropriate when hospitals have very knowledgeable and adequate number of staff in ICT (1) | -1.483 | .794 | 3.485 | 1 | .062 | .227 | .048 | 1.077 |
|  | ICT staff : M-Health adoption is appropriate when hospitals have very knowledgeable and adequate number of staff in ICT (2) | -1.232 | .656 | 3.534 | 1 | .060 | .292 | .081 | 1.054 |
|  | ICT staff : M-Health adoption is appropriate when hospitals have very knowledgeable and adequate number of staff in ICT (3) | -1.085 | .599 | 3.284 | 1 | .070 | .338 | .105 | 1.093 |
|  | Scope of the Market: M-Health adoption is appropriate for hospitals with larger market scopes such as national, regional or global markets. |  |  | 2.478 | 3 | .479 |  |  |  |
|  | Scope of the Market: M-Health adoption is appropriate for hospitals with larger market scopes such as national, regional or global markets.(1) | -.698 | .826 | .714 | 1 | .398 | .498 | .099 | 2.513 |
|  | Scope of the Market: M-Health adoption is appropriate for hospitals with larger market scopes such as national, regional or global markets.(2) | -1.065 | .769 | 1.914 | 1 | .167 | .345 | .076 | 1.558 |
|  | Scope of the Market: M-Health adoption is appropriate for hospitals with larger market scopes such as national, regional or global markets.(3) | -.317 | .740 | .184 | 1 | .668 | .728 | .171 | 3.108 |
|  | Slack/Financial Resources: M-health adoption is appropriate for hospitals that have excess budgets to invest in new IT technologies (soft and hard ware) such as M-Health |  |  | 2.048 | 3 | .562 |  |  |  |
|  | Slack/Financial Resources: M-health adoption is appropriate for hospitals that have excess budgets to invest in new IT technologies (soft and hard ware) such as M-Health(1) | -.244 | .845 | .084 | 1 | .773 | .783 | .150 | 4.102 |
|  | Slack/Financial Resources: M-health adoption is appropriate for hospitals that have excess budgets to invest in new IT technologies (soft and hard ware) such as M-Health(2) | .138 | .844 | .027 | 1 | .870 | 1.148 | .220 | 6.002 |
|  | Slack/Financial Resources: M-health adoption is appropriate for hospitals that have excess budgets to invest in new IT technologies (soft and hard ware) such as M-Health(3) | .596 | .777 | .590 | 1 | .443 | 1.816 | .396 | 8.318 |
|  | Technology leadership: M-Health is appropriate for hospitals that pursue market growth through technology leadership |  |  | 2.345 | 3 | .504 |  |  |  |
|  | Technology leadership: M-Health is appropriate for hospitals that pursue market growth through technology leadership (1) | 1.051 | .748 | 1.972 | 1 | .160 | 2.860 | .660 | 12.394 |
|  | Technology leadership: M-Health is appropriate for hospitals that pursue market growth through technology leadership (2) | .265 | .690 | .147 | 1 | .701 | 1.303 | .337 | 5.037 |
|  | Technology leadership: M-Health is appropriate for hospitals that pursue market growth through technology leadership (3) | .531 | .590 | .810 | 1 | .368 | 1.701 | .535 | 5.407 |
|  | Constant | -.580 | .793 | .535 | 1 | .465 | .560 |  |  |
| a. Variable(s) entered on step 1: Decision making structure: decision to adopt or not to adopt M-Health adoption is the prerogative of the hospital’s top management only., Size: M-Health adoption is appropriate only when the hospital has substantial volume of patients and staff to justify its adoption, ICT capacity: M-Health adoption is appropriate when hospitals have a very complex ICT infrastructure, ICT staff : M-Health adoption is appropriate when hospitals have very knowledgeable and adequate number of staff in ICT , Scope of the Market: M-Health adoption is appropriate for hospitals with larger market scopes such as national, regional or global markets., Slack/Financial Resources: M-health adoption is appropriate for hospitals that have excess budgets to invest in new IT technologies (soft and hard ware) such as M-Health, Technology leadership: M-Health is appropriate for hospitals that pursue market growth through technology leadership . | | | | | | | | | |

1. **Surveillance technology Adoption and organizational determinants**

**Logistic Regression**

| **Notes** | | |
| --- | --- | --- |
| Output Created | | 15-JUL-2018 15:07:45 |
| Comments | |  |
| Input | Data | C:\Users\Ocholap\Downloads\m-health data.- 13-JULY 2018.sav |
|  | Active Dataset | DataSet1 |
|  | Filter | <none> |
|  | Weight | <none> |
|  | Split File | <none> |
|  | N of Rows in Working Data File | 211 |
| Missing Value Handling | Definition of Missing | User-defined missing values are treated as missing |
| Syntax | | LOGISTIC REGRESSION VARIABLES Q20.9  /METHOD=ENTER Q24.1 Q24.2 Q24.3 Q24.4 Q24.5 Q24.6 Q24.7  /CONTRAST (Q24.1)=Indicator  /CONTRAST (Q24.2)=Indicator  /CONTRAST (Q24.3)=Indicator  /CONTRAST (Q24.4)=Indicator  /CONTRAST (Q24.5)=Indicator  /CONTRAST (Q24.6)=Indicator  /CONTRAST (Q24.7)=Indicator  /SAVE=PGROUP  /PRINT=GOODFIT SUMMARY CI(95)  /CRITERIA=PIN(0.05) POUT(0.10) ITERATE(20) CUT(0.5). |
| Resources | Processor Time | 00:00:00.03 |
|  | Elapsed Time | 00:00:00.03 |
| Variables Created or Modified | PGR_36 | Predicted group |

| **Case Processing Summary** | | | |
| --- | --- | --- | --- |
| Unweighted Cases^a^ | | N | Percent |
| Selected Cases | Included in Analysis | 192 | 91.0 |
|  | Missing Cases | 19 | 9.0 |
|  | Total | 211 | 100.0 |
| Unselected Cases | | 0 | .0 |
| Total | | 211 | 100.0 |
| a. If weight is in effect, see classification table for the total number of cases. | | | |

| **Dependent Variable Encoding** | |
| --- | --- |
| Original Value | Internal Value |
| Not adopted | 0 |
| Adopted | 1 |

| **Categorical Variables Codings** | | | | | |
| --- | --- | --- | --- | --- | --- |
|  | | Frequency | Parameter coding | | |
|  |  |  | (1) | (2) | (3) |
| Technology leadership: M-Health is appropriate for hospitals that pursue market growth through technology leadership | Strongly disgree | 28 | 1.000 | .000 | .000 |
|  | Disagree | 45 | .000 | 1.000 | .000 |
|  | Agree | 69 | .000 | .000 | 1.000 |
|  | Strongly Agree | 50 | .000 | .000 | .000 |
| Size: M-Health adoption is appropriate only when the hospital has substantial volume of patients and staff to justify its adoption | Strongly disgree | 55 | 1.000 | .000 | .000 |
|  | Disagree | 73 | .000 | 1.000 | .000 |
|  | Agree | 46 | .000 | .000 | 1.000 |
|  | Strongly Agree | 18 | .000 | .000 | .000 |
| ICT capacity: M-Health adoption is appropriate when hospitals have a very complex ICT infrastructure | Strongly disgree | 58 | 1.000 | .000 | .000 |
|  | Disagree | 86 | .000 | 1.000 | .000 |
|  | Agree | 26 | .000 | .000 | 1.000 |
|  | Strongly Agree | 22 | .000 | .000 | .000 |
| ICT staff : M-Health adoption is appropriate when hospitals have very knowledgeable and adequate number of staff in ICT | Strongly disgree | 24 | 1.000 | .000 | .000 |
|  | Disagree | 62 | .000 | 1.000 | .000 |
|  | Agree | 69 | .000 | .000 | 1.000 |
|  | Strongly Agree | 37 | .000 | .000 | .000 |
| Scope of the Market: M-Health adoption is appropriate for hospitals with larger market scopes such as national, regional or global markets. | Strongly disgree | 52 | 1.000 | .000 | .000 |
|  | Disagree | 80 | .000 | 1.000 | .000 |
|  | Agree | 36 | .000 | .000 | 1.000 |
|  | Strongly Agree | 24 | .000 | .000 | .000 |
| Slack/Financial Resources: M-health adoption is appropriate for hospitals that have excess budgets to invest in new IT technologies (soft and hard ware) such as M-Health | Strongly disgree | 71 | 1.000 | .000 | .000 |
|  | Disagree | 68 | .000 | 1.000 | .000 |
|  | Agree | 39 | .000 | .000 | 1.000 |
|  | Strongly Agree | 14 | .000 | .000 | .000 |
| Decision making structure: decision to adopt or not to adopt M-Health adoption is the prerogative of the hospital’s top management only. | Strongly disgree | 54 | 1.000 | .000 | .000 |
|  | Disagree | 68 | .000 | 1.000 | .000 |
|  | Agree | 41 | .000 | .000 | 1.000 |
|  | Strongly Agree | 29 | .000 | .000 | .000 |

**Block 0: Beginning Block**

| **Classification Table^a,b^** | | | | | |
| --- | --- | --- | --- | --- | --- |
|  | Observed | | Predicted | | |
|  |  |  | Surveillance | | Percentage Correct |
|  |  |  | Not adopted | Adopted |  |
| Step 0 | Surveillance | Not adopted | 124 | 0 | 100.0 |
|  |  | Adopted | 68 | 0 | .0 |
|  | Overall Percentage | |  |  | 64.6 |
| a. Constant is included in the model. | | | | | |
| b. The cut value is .500 | | | | | |

| **Variables in the Equation** | | | | | | | |
| --- | --- | --- | --- | --- | --- | --- | --- |
|  | | B | S.E. | Wald | df | Sig. | Exp(B) |
| Step 0 | Constant | -.601 | .151 | 15.851 | 1 | .000 | .548 |

| **Variables not in the Equation** | | | | | |
| --- | --- | --- | --- | --- | --- |
|  | | | Score | df | Sig. |
| Step 0 | Variables | Decision making structure: decision to adopt or not to adopt M-Health adoption is the prerogative of the hospital’s top management only. | 3.036 | 3 | .386 |
|  |  | Decision making structure: decision to adopt or not to adopt M-Health adoption is the prerogative of the hospital’s top management only.(1) | 2.677 | 1 | .102 |
|  |  | Decision making structure: decision to adopt or not to adopt M-Health adoption is the prerogative of the hospital’s top management only.(2) | 1.660 | 1 | .198 |
|  |  | Decision making structure: decision to adopt or not to adopt M-Health adoption is the prerogative of the hospital’s top management only.(3) | .037 | 1 | .848 |
|  |  | Size: M-Health adoption is appropriate only when the hospital has substantial volume of patients and staff to justify its adoption | 8.136 | 3 | .043 |
|  |  | Size: M-Health adoption is appropriate only when the hospital has substantial volume of patients and staff to justify its adoption(1) | 4.737 | 1 | .030 |
|  |  | Size: M-Health adoption is appropriate only when the hospital has substantial volume of patients and staff to justify its adoption(2) | 3.312 | 1 | .069 |
|  |  | Size: M-Health adoption is appropriate only when the hospital has substantial volume of patients and staff to justify its adoption(3) | 1.354 | 1 | .245 |
|  |  | ICT capacity: M-Health adoption is appropriate when hospitals have a very complex ICT infrastructure | 8.102 | 3 | .044 |
|  |  | ICT capacity: M-Health adoption is appropriate when hospitals have a very complex ICT infrastructure(1) | 4.505 | 1 | .034 |
|  |  | ICT capacity: M-Health adoption is appropriate when hospitals have a very complex ICT infrastructure(2) | 1.830 | 1 | .176 |
|  |  | ICT capacity: M-Health adoption is appropriate when hospitals have a very complex ICT infrastructure(3) | 3.444 | 1 | .063 |
|  |  | ICT staff : M-Health adoption is appropriate when hospitals have very knowledgeable and adequate number of staff in ICT | 1.652 | 3 | .648 |
|  |  | ICT staff : M-Health adoption is appropriate when hospitals have very knowledgeable and adequate number of staff in ICT (1) | 1.301 | 1 | .254 |
|  |  | ICT staff : M-Health adoption is appropriate when hospitals have very knowledgeable and adequate number of staff in ICT (2) | .113 | 1 | .737 |
|  |  | ICT staff : M-Health adoption is appropriate when hospitals have very knowledgeable and adequate number of staff in ICT (3) | .019 | 1 | .891 |
|  |  | Scope of the Market: M-Health adoption is appropriate for hospitals with larger market scopes such as national, regional or global markets. | 5.305 | 3 | .151 |
|  |  | Scope of the Market: M-Health adoption is appropriate for hospitals with larger market scopes such as national, regional or global markets.(1) | 2.422 | 1 | .120 |
|  |  | Scope of the Market: M-Health adoption is appropriate for hospitals with larger market scopes such as national, regional or global markets.(2) | 5.038 | 1 | .025 |
|  |  | Scope of the Market: M-Health adoption is appropriate for hospitals with larger market scopes such as national, regional or global markets.(3) | .234 | 1 | .629 |
|  |  | Slack/Financial Resources: M-health adoption is appropriate for hospitals that have excess budgets to invest in new IT technologies (soft and hard ware) such as M-Health | 5.679 | 3 | .128 |
|  |  | Slack/Financial Resources: M-health adoption is appropriate for hospitals that have excess budgets to invest in new IT technologies (soft and hard ware) such as M-Health(1) | 3.349 | 1 | .067 |
|  |  | Slack/Financial Resources: M-health adoption is appropriate for hospitals that have excess budgets to invest in new IT technologies (soft and hard ware) such as M-Health(2) | 4.995 | 1 | .025 |
|  |  | Slack/Financial Resources: M-health adoption is appropriate for hospitals that have excess budgets to invest in new IT technologies (soft and hard ware) such as M-Health(3) | .005 | 1 | .944 |
|  |  | Technology leadership: M-Health is appropriate for hospitals that pursue market growth through technology leadership | 1.544 | 3 | .672 |
|  |  | Technology leadership: M-Health is appropriate for hospitals that pursue market growth through technology leadership (1) | .793 | 1 | .373 |
|  |  | Technology leadership: M-Health is appropriate for hospitals that pursue market growth through technology leadership (2) | 1.095 | 1 | .295 |
|  |  | Technology leadership: M-Health is appropriate for hospitals that pursue market growth through technology leadership (3) | .031 | 1 | .860 |
|  | Overall Statistics | | 27.067 | 21 | .169 |

**Block 1: Method = Enter**

| **Omnibus Tests of Model Coefficients** | | | | |
| --- | --- | --- | --- | --- |
|  | | Chi-square | df | Sig. |
| Step 1 | Step | 28.928 | 21 | .116 |
|  | Block | 28.928 | 21 | .116 |
|  | Model | 28.928 | 21 | .116 |

| **Model Summary** | | | |
| --- | --- | --- | --- |
| Step | -2 Log likelihood | Cox & Snell R Square | Nagelkerke R Square |
| 1 | 220.667^a^ | .140 | .192 |
| a. Estimation terminated at iteration number 4 because parameter estimates changed by less than .001. | | | |

| **Hosmer and Lemeshow Test** | | | |
| --- | --- | --- | --- |
| Step | Chi-square | df | Sig. |
| 1 | 12.741 | 8 | .121 |

| **Contingency Table for Hosmer and Lemeshow Test** | | | | | | |
| --- | --- | --- | --- | --- | --- | --- |
|  | | Surveillance = Not adopted | | Surveillance = Adopted | | Total |
|  |  | Observed | Expected | Observed | Expected |  |
| Step 1 | 1 | 16 | 17.250 | 3 | 1.750 | 19 |
|  | 2 | 18 | 16.803 | 2 | 3.197 | 20 |
|  | 3 | 15 | 15.068 | 4 | 3.932 | 19 |
|  | 4 | 16 | 14.128 | 3 | 4.872 | 19 |
|  | 5 | 15 | 13.101 | 4 | 5.899 | 19 |
|  | 6 | 9 | 11.981 | 10 | 7.019 | 19 |
|  | 7 | 13 | 11.224 | 6 | 7.776 | 19 |
|  | 8 | 5 | 10.129 | 14 | 8.871 | 19 |
|  | 9 | 10 | 7.845 | 8 | 10.155 | 18 |
|  | 10 | 7 | 6.471 | 14 | 14.529 | 21 |

| **Classification Table^a^** | | | | | |
| --- | --- | --- | --- | --- | --- |
|  | Observed | | Predicted | | |
|  |  |  | Surveillance | | Percentage Correct |
|  |  |  | Not adopted | Adopted |  |
| Step 1 | Surveillance | Not adopted | 107 | 17 | 86.3 |
|  |  | Adopted | 43 | 25 | 36.8 |
|  | Overall Percentage | |  |  | 68.8 |
| a. The cut value is .500 | | | | | |

| **Variables in the Equation** | | | | | | | | | |
| --- | --- | --- | --- | --- | --- | --- | --- | --- | --- |
|  | | B | S.E. | Wald | df | Sig. | Exp(B) | 95% C.I.for EXP(B) | |
| Lower | Upper |
| Step 1^a^ | Decision making structure: decision to adopt or not to adopt M-Health adoption is the prerogative of the hospital’s top management only. |  |  | 1.382 | 3 | .710 |  |  |  |
| Decision making structure: decision to adopt or not to adopt M-Health adoption is the prerogative of the hospital’s top management only.(1) | .476 | .634 | .564 | 1 | .453 | 1.610 | .465 | 5.577 |
| Decision making structure: decision to adopt or not to adopt M-Health adoption is the prerogative of the hospital’s top management only.(2) | .447 | .613 | .532 | 1 | .466 | 1.563 | .470 | 5.192 |
| Decision making structure: decision to adopt or not to adopt M-Health adoption is the prerogative of the hospital’s top management only.(3) | .759 | .652 | 1.357 | 1 | .244 | 2.136 | .596 | 7.661 |
| Size: M-Health adoption is appropriate only when the hospital has substantial volume of patients and staff to justify its adoption |  |  | 2.739 | 3 | .434 |  |  |  |
| Size: M-Health adoption is appropriate only when the hospital has substantial volume of patients and staff to justify its adoption(1) | -.420 | .732 | .330 | 1 | .566 | .657 | .157 | 2.757 |
| Size: M-Health adoption is appropriate only when the hospital has substantial volume of patients and staff to justify its adoption(2) | -.975 | .749 | 1.692 | 1 | .193 | .377 | .087 | 1.639 |
| Size: M-Health adoption is appropriate only when the hospital has substantial volume of patients and staff to justify its adoption(3) | -.949 | .728 | 1.700 | 1 | .192 | .387 | .093 | 1.612 |
| ICT capacity: M-Health adoption is appropriate when hospitals have a very complex ICT infrastructure |  |  | 5.811 | 3 | .121 |  |  |  |
| ICT capacity: M-Health adoption is appropriate when hospitals have a very complex ICT infrastructure(1) | .463 | .765 | .366 | 1 | .545 | 1.589 | .354 | 7.123 |
| ICT capacity: M-Health adoption is appropriate when hospitals have a very complex ICT infrastructure(2) | -.094 | .728 | .017 | 1 | .897 | .910 | .218 | 3.793 |
| ICT capacity: M-Health adoption is appropriate when hospitals have a very complex ICT infrastructure(3) | -1.241 | .827 | 2.252 | 1 | .133 | .289 | .057 | 1.462 |
| ICT staff : M-Health adoption is appropriate when hospitals have very knowledgeable and adequate number of staff in ICT |  |  | 8.079 | 3 | .044 |  |  |  |
| ICT staff : M-Health adoption is appropriate when hospitals have very knowledgeable and adequate number of staff in ICT (1) | -1.647 | .771 | 4.565 | 1 | .033 | .193 | .043 | .873 |
| ICT staff : M-Health adoption is appropriate when hospitals have very knowledgeable and adequate number of staff in ICT (2) | .273 | .600 | .208 | 1 | .649 | 1.315 | .406 | 4.260 |
| ICT staff : M-Health adoption is appropriate when hospitals have very knowledgeable and adequate number of staff in ICT (3) | -.022 | .575 | .001 | 1 | .970 | .979 | .317 | 3.019 |
| Scope of the Market: M-Health adoption is appropriate for hospitals with larger market scopes such as national, regional or global markets. |  |  | 3.251 | 3 | .355 |  |  |  |
| Scope of the Market: M-Health adoption is appropriate for hospitals with larger market scopes such as national, regional or global markets.(1) | -.338 | .713 | .225 | 1 | .635 | .713 | .176 | 2.884 |
| Scope of the Market: M-Health adoption is appropriate for hospitals with larger market scopes such as national, regional or global markets.(2) | -1.017 | .664 | 2.348 | 1 | .125 | .362 | .098 | 1.328 |
| Scope of the Market: M-Health adoption is appropriate for hospitals with larger market scopes such as national, regional or global markets.(3) | -.448 | .663 | .456 | 1 | .499 | .639 | .174 | 2.343 |
[truncated: 724,961 more chars]
